# Behavioural Risk Factors in Mid-Life Associated with Successful Ageing, Disability, Dementia and Frailty in Later Life: A Rapid Systematic Review

**DOI:** 10.1371/journal.pone.0144405

**Published:** 2016-02-04

**Authors:** Louise Lafortune, Steven Martin, Sarah Kelly, Isla Kuhn, Olivia Remes, Andy Cowan, Carol Brayne

**Affiliations:** 1 Institute of Public Health, Forvie Site, University of Cambridge School of Clinical Medicine, Cambridge Biomedical Campus, Cambridge, United Kingdom; 2 University of Cambridge Medical Library, University of Cambridge School of Clinical Medicine, Cambridge Biomedical Campus, Cambridge, United Kingdom; National Institute for Viral Disease Control and Prevention, CDC, China, CHINA

## Abstract

**Background:**

Smoking, alcohol consumption, poor diet and low levels of physical activity significantly contribute to the burden of illness in developed countries. Whilst the links between specific and multiple risk behaviours and individual chronic conditions are well documented, the impact of these behaviours in mid-life across a range of later life outcomes has yet to be comprehensively assessed. This review aimed to provide an overview of behavioural risk factors in mid-life that are associated with successful ageing and the primary prevention or delay of disability, dementia, frailty and non-communicable chronic conditions.

**Methods:**

A literature search was conducted to identify cohort studies published in English since 2000 up to Dec 2014. Multivariate analyses and a minimum follow-up of five years were required for inclusion. Two reviewers screened titles, abstracts and papers independently. Studies were assessed for quality. Evidence was synthesised by mid-life behavioural risk for a range of late life outcomes.

**Findings:**

This search located 10,338 individual references, of which 164 are included in this review. Follow-up data ranged from five years to 36 years. Outcomes include dementia, frailty, disability and cardiovascular disease. There is consistent evidence of beneficial associations between mid-life physical activity, healthy ageing and disease outcomes. Across all populations studied there is consistent evidence that mid-life smoking has a detrimental effect on health. Evidence specific to alcohol consumption was mixed. Limited, but supportive, evidence was available relating specifically to mid-life diet, leisure and social activities or health inequalities.

**Conclusions:**

There is consistent evidence of associations between mid-life behaviours and a range of late life outcomes. The promotion of physical activity, healthy diet and smoking cessation in all mid-life populations should be encouraged for successful ageing and the prevention of disability and chronic disease.

## Background

Non-communicable chronic conditions and disabilities that manifest in later life are heavily influenced by physical and social exposures, including behaviours, across the life course [1,2], leading to an accumulation of risks in older age. The four main behavioural risk factors (smoking, excessive consumption of alcohol, poor diet and low levels of physical activity) have been estimated to contribute to close to half of the burden of illness in developed countries [3,4] and are known to be unequally distributed in the population particularly affecting the most vulnerable in society [5]. Importantly there is a contingent and temporal nature of these behavioural exposures in mid-life [6–8] and epidemiological data suggests that it ought to be possible to prevent or delay morbidity and mortality [9] whilst promoting successful aging and quality of life [10–12], if adoption of healthy behaviours is encouraged across life course and society.

Although many systematic reviews have looked at the links between individual behavioural risk factors and health outcomes [13–19], a comprehensive overview across behavioural risk factors and outcomes has yet to be undertaken. There remain uncertainties regarding associations between the relative contributions of behavioural and lifestyle factors, in mid-life specifically, to prevalence, risk and outcomes of non-communicable chronic conditions (for example, dementia, cancer and cardiovascular diseases), disabilities and frailty (including quality of life, and mortality). That is particularly true for the relationship between behavioural risk factors and frailty, where the operational definition of this complex syndrome is still controversial [20,21]; and for dementia where the aetiology and natural history of disease is still uncertain [22,23].

This systematic review was one of a series of reviews conducted to inform the development of UK national public health guidance on mid-life approaches to prevent dementia, disability and frailty in later life [24]. The aim of the review was to assess the behavioural risk factors in mid-life that are associated with successful ageing and the primary prevention or delay of disability, dementia, frailty (DDF) and non-communicable chronic conditions. The goal was not to summarise the whole of the epidemiological evidence on behavioural risk in adult life. Rather, we aimed to identify associations specifically derived from people in mid-life to inform the development of well-targeted interventions that will minimise the impact of ill health in later life. With a similar focus, the other two reviews in the series looked at the effectiveness of mid-life interventions on behavioural risks and late life outcomes, and at key issues for people in mid-life that prevent or limit, or which help or motivate them to take up and maintain healthy behaviours.

## Methods

The review was conducted as a rapid systematic review to provide best available evidence within limited timescales. The scope of the review was defined by the funders (National Institute for Health and Care Excellence—NICE), after open consultation with stakeholders and the protocol (S1 Text) was agreed prior to the start of work. Established systematic review methods of NICE (NICE 2015) were broadly followed, except as described below.

### Searches

The following electronic sources were searched for peer-reviewed studies published in the English language since the year 2000: MEDLINE, EMBASE, PsycINFO, CINAHL, Health Management Information Consortium, Social Science Citation Index, the HTA database, Cochrane Database of Systematic Reviews, and Cochrane Database of Abstracts of Reviews of Effectiveness as well as relevant websites (S2 Text).

Primary longitudinal cohort studies were identified using an observational study search filter [25]. To enable a manageable number of search hits, the searches were limited to studies in which terms related to mid-life/middle-aged were indexed in the title/abstract or MeSH term. Time constraints precluded hand searches or contact of authors for additional data.

### Inclusion and exclusion criteria

#### Populations

The populations covered by this review included 1) mid-life adults (aged 40–64 years), 2) adults aged 39 and younger in populations at higher risk of health inequalities, which refer to people from disadvantaged and minority groups. For this review ‘disadvantaged and minority groups’ includes (but is not limited to) people of low socioeconomic status; ethnic minority groups; LGBT community groups; travellers; and other groups with protected characteristics under the equality and diversity legislation.

Studies in populations with mid-life dementia or pre-existing cognitive impairment, and non-communicable chronic conditions were excluded, as were studies of specific disabilities associated with modifiable behavioural risk factors

#### Exposure

Behavioural risk factors in the populations described above include (but are not limited to): physical activity; diet and nutrition; smoking; alcohol consumption; and social activity. Studies that reported mid-life weight change/cycling (as a health behaviour) were included whereas studies focusing on mid-life obesity (as a risk factor) were excluded.

#### Outcomes

Outcomes included (but were not limited to): dementia, disability (i.e. activities of daily living (ADL), instrumental activities of daily living (IADL), independence, mobility), frailty, quality of life, cardiovascular diseases and stroke, renal disease, cancer, chronic obstructive pulmonary disease, type 2 diabetes, osteoporosis and bone health, mental health.

#### Study design

Primary longitudinal cohort studies that reported multivariate analyses and provided information on the association between behavioural risk factors at mid-life and health or ageing outcomes in later life (>/ = 5 years follow-up) as listed above were eligible for inclusion. Cross-sectional and univariate analyses were excluded. Abstracts, letters and editorials were also excluded.

#### Identification of relevant studies

**Fig 1:**
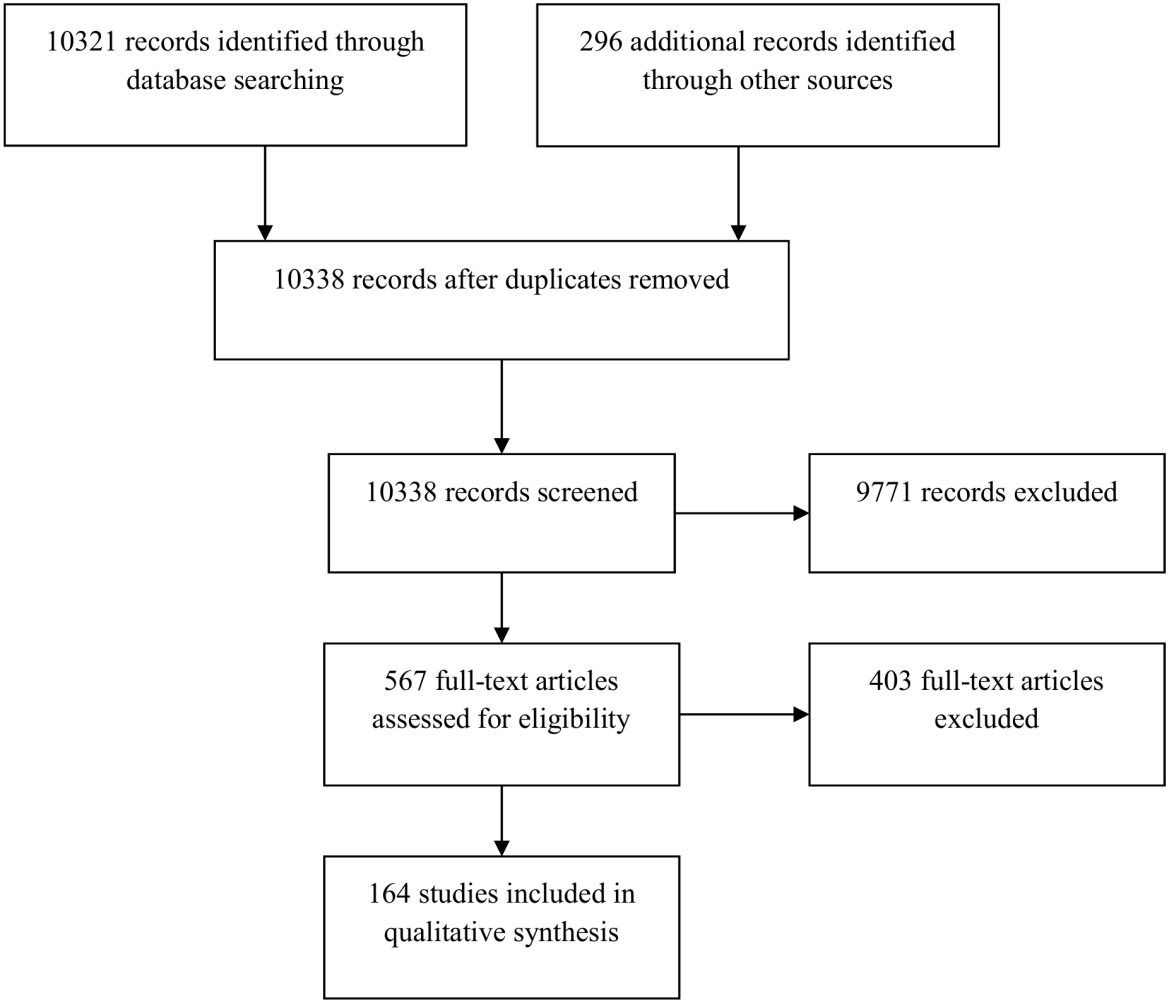
Search Results for Primary Studies.

Titles, abstracts and papers were screened for inclusion by two reviewers. Differences between reviewers’ results were resolved by discussion and when necessary in consultation with a third reviewer. Fig 1 illustrates the flow chart for the study selection process. Studies excluded at the full paper screening stage are listed in S1 Table along with the reason for exclusion.

### Quality Assessment

Quality appraisal of cohort studies was done using a validated quality appraisal checklist [24]. Each full paper was assessed by one reviewer and checked for accuracy by another. A minimum of 10% of the studies was assessed twice and discrepancies resolved by discussion. No studies were excluded on the basis of quality.

### Data extraction and evidence synthesis

Data was extracted on study detail, population and setting, study design, outcomes and method of analysis, and results. To ensure accurate reporting, the data extraction pro-forma was piloted against a selection of papers. A full summary of studies, exposures, outcomes and details of effect sizes are reported in Table 1.

**Table 1:** Overview of Included Studies.

Study	Country	n	Age at baseline	Length of follow-up	Quality
**Physical Activity (PA)**					
Andel 2008 (Case control study)	Sweden	264 dementia 2870 controls (90 twin pairs)	Mean 48.1 (SD 4.9)	31.4 years	+
Britton 2008	UK (England)	5823	35–55	17 years	+
Carlson 2008	US	147 twin pairs	45 (SD 3)	20–40 years	++
Chang 2010	Iceland	4761	51	26 years	-
Chang 2013 (Same study as Chang 2010, different outcomes)	Iceland	4753	51 (SD 7)	25 years	-
Debette 2011	USA	1352	61±9	10yrs	+
Ekelund 2005	UK (England)	605	53 (mean)	5.6 years	+
Elwood 2013 (Caerphilly cohort study)	UK (Wales)	2235	45–59	30 years	++
Englund 2011 (Case-control study)	Sweden	81 cases/ 156 controls	57 (SD 5)	11 years	-
Englund 2013 (Case-control study)	Sweden	376 cases/402 controls	54 (SD 6)	11 years	-
Friedland 2001 (Case-control study)	US	193 cases/358 controls (for total study, not reported for 40–59 year olds)	40–59	>12 (not fully reported)	-
Harmsen 2006	Sweden	6193	47–55	28 years	+
Hamer 2013	UK (England)	3454	63.7 (SD 8.9)	8 years	++
Holtermann 2009	Denmark	4952	40–59	30 years	+
Holme 2007 (Oslo study)	Norway	6382	40–49	28 years	-
Hu 2003	Finland	13290	35–64	12 years	+
Hu 2004	Finland	18892	25–74 (mean age 42–48)	9.8 years	+
Hu 2005	Finland	47212	25–64 (mean age 41–46)	17.7 years	+
Hu 2007	Finland		25–64 (mean age 42–49)	18.9 years	+
Knopman 2001	USA	10,963	47–70	6yrs	+
Lahti 2010	Finland	5437 women, 1257 men	49–51	5–7 years	+
Lang 2007	UK [England (ELSA study), and US]	8702 (US)& 1507 (UK) (from 2 studies	50–69 (mean 60.2 & 58)	6 years	+
Malmberg 2006	Finland	1791	40–64	16 years	+
Meisinger 2007	Germany	3501 men, 3475 women	45–74	8.6 years	+
Menotti 2006	Italy	1712	40–49	5 years	+
Morgan 2012 (Caerphilly cohort study)	UK (Wales)	1005	45–59	16 years	+
Nokes 2012	US	244	35–45	6 years	+
Ostbye 2002	US	7845 (HRS study) 5037	51–61	5–6 yrs	-
Patel 2006	Italy	1001	40–60	7 years	+
Pitsavos 2004	Greece (Corfu)	529	49 ±6	40 years	+
Riserus 2007	Sweden	770	50	20 yrs (70–73 to 91–95)	+
Rovio 2005	Sweden	2000	50	21 years	+
Rovio 2007 (Same study as Rovio 2005)	Sweden	1449	50	21 years	+
Sabia 2009	England	5123	44 (mean)	17 years (85–88 to 02–04)	+
Stevens 2009	England and Scotland	1.29 million women	50–64 (mean 56)	96–01 to 05–07 Mean yrs of follow-up: 7.2 for cancer incidence; 8.9 for mortality	+
Sun 2010	US	13535	60 (mean age)	14 years	++
Szoeke 2006	US	224	50 (mean)	11 years	+
Wannamethee 2001	UK (England)	7630	40–59	18.8 years	+
Wiles 2007 (Caerphilly cohort study)	UK (Wales)	2512	45–59	10 years	++
Xu 2010	South East Queensland, Australia	564	45–60 yrs (mean 55)	2001–06	**-**
Yu 2003 (Caerphilly cohort study)	UK (Wales)	1975	45–59	10.5 years	+
**Physical Inactivity**					
Christensen 2006	Denmark	376	50, 60, 70	25 years	-
Haapanen-Niemi 2000	Finland	2212 (295 PA)	35–63	16 years	+
**Diet**					
Akbaraly 2013	UK	8815	35–55 years	18 years	+
Britton 2008	UK	5823	35–55 years (mean: 44)	20 years	+
Elwood 2013	UK (Caerphilly)	2235	45–59 years	30 years	+
Eskelinen 2008	Finland	1449	SD: 50.2	21 years	+
Eskelinen 2009 (CAIDE study)	Finland	1409	SD: 50.4	21 years	+
He 2004	United States	74063	38–63 years	12 years	+
Hodge 2013	Australia	8660	50–69 years	12 years	+
Hughes 2010	Sweden	3779 (3424 non-demented, 355 dementia cases)	Mean age 48	30 years	+
Hu 2007	Finland	29335	25–64 years	18.9 years	+
Kesse-Guyot 2012	France	3054	SD: 52.1	13.4 years	-
Laitala 2009	Finland	2606	Mean age 46–52	28 years	++
Laitinen 2006	Finland	1449	SD: 50.4	21 years	++
Laurin 2004	US (Hawaii)	2459	45–68 years (Mean: 51.2)	30.2 years	+
Lehto 2013	Finland	2600	42–61 years	20.1 years	+
Liu 2003	United States	74091	38–63 years	12 years	+
Masaki 2003	Japan	5644	40–69	10 years	+
Miura 2004	United States	1710	40–55 years (Mean 48.5)	39 years	+
Nakamura 2009	Japan	2316	47–60 years	19 years	+
Nooyens 2011 (Doetinchem Cohort Study)	Netherlands	2613	43–70 years	10 years	+
Osler 2003	Denmark	7540	30–70 years	36 years	+
Ross 2000	US Hawaii	8004	45–68 years	30 years	+
Ruder 2011	United States	292797	40–61 years	10 years	++
Ruusanen 2010	Finland	2232	42–60 years	17.5 years	+
Sabia 2009	UK	5123	35–55 years	17 years	+
Samieri 2013	United States	10670	Upper 50s, lower 60s (SD: 59)	15.2 years	+
Seccareccia 2003	Italy	1536	45–65 years	30 years	+
Song 2006	United States	28349	45+ years	9.8 years	+
Strandhagen 2000	Sweden	792	Age 54	26 years	++
Tsugane 2004	Japan	39065	40–59 years	11 years	+
Walda 2002	Finland, Italy and The Netherlands	2917	50–69 years	20 years	+
Wang 2009	United States	38408	45+ years	11.5 years	+
Wang 2012	United States	28082	39+	12.9 years	+
Wang 2008	United States	28766	45+ years (SD 53.8)	10 years	+
Xu 2010	Australia	564	45–60 years	5 years	-
**Smoking**					
Agahi 2013	Sweden	1060	30–50	Up to 34yrs	-
Alonso 2009	USA	11,151	45–64	Up to 10yrs	+
Baba 2006	Japan	41,307	40-59yrs	11yrs	+
Blanco-Cedres 2002	USA	8,816	40-59yrs	25yrs	+
Boudik 2006	Prague	926 men	Mean 46.1 (Middle aged men)	21yrs	-
Britton 2008	England	5823 (civil servant)	35–55	17yrs	+
Dubas 2007	Sweden	7388	47–55	28yrs (‘70-‘98)	++
Englund 2013	Sweden	778	54±5.9	11.2±2.6	+
Fogelholm 2000	Finland	1143	36–88	10 yrs (‘85-‘95)	+
Gerber 2012	Israel	4633	50.1±6.5	Median 26yrs (quartiles 1–3: 16–35)	++
Halperin 2008	USA	13,529	52.4±8.9	Med 14.5yrs Max 20.5yrs	+
Hara 2002	Japan	41,484	40–59	Never: 64,986 PA Former: 42,798 PA Current: 103,537 PA	+
Harmsen 2006	Sweden	7457	Middle-age men	28yrs	+
Holme 2007	Norway	6382(M)	40–49	28yrs	+
Holmberg 2006	Sweden	22444 (M) 10902(W)	Men: 27–61 yrs. Women: 28–58 yrs	19yrs (M) 15yrs (W)	+
Humphries 2001	UK	3052 men	55.7±3.2	11yrs	+
Inoue 2004	Japan	92,792	40–69 (mean 53)	10 yrs	+
Janzon 2004	Sweden	10619	49 yrs (28.3–57.6)	14.0±4.5yrs (range 0.5–21.9 years)	+
Khalili 2002	Sweden	22 444 –(not clear)	Mean 42.2	17yrs	+
Kimm 2011	Korea	3252	Men 51.9 ±8.7 Women 53.6 ±9.9	14yrs	+
Lim 2013	Singapore	48,251	45–74	93–98 to 2009	-
Mannami 2004	Japan	19,782 men and 21,500 women	40–59	90–92 to 01 (total of 461,761 person-year follow-up)	++
Moayyeri 2009	UK	25,311	W: 64.7 (8.4) M: 61.9 (9.7) (40-75yrs)	11.3yrs (SD = 1.5; range 9.2–14.1)	+
Nakayama 2000	Japan	998	40-64yrs	20yrs	+
Noborisaka 2013	Japan	6998	Men 84.3% bt 30-59yrs Women 86.3% bt 30-59yrs	6 yrs	+
Nooyens 2008	Netherlands	1964	56.0 (7.0)	5yrs	+
Nafziger 2007	Sweden	82927	30–60	10 years	++
Östenson 2012	Sweden	2382	47.2 (46.9–47.4)	10 years	+
Ostbye 2002	US	7,845	51–61 years	HRS: 6yrs	-
Otani 2003	Japan	19,862 (cohort 1). 10,212 (cohort 2)	48.9 (6.0) (cohort 1). 53.4 (8.2) (cohort 2)	10yrs (cohort 1). 7yrs (cohort 2)	+
Patja 2005	Finland	41 372	25–64	Mean follow-up 21 years	-
Pelkonen 2000	Finland	1582	Not reported	30yrs	++
Qiao 2000	Finland	1673	Not reported	35yrs	+
Qiu 2003	China	50,069	55.3±11.8	6yrs	+
Räikkönen 2001	USA	541	48.0±1.5	9.2yrs; SD, 3.4 years	+
Riserus 2007	Sweden	770	50	20 yrs (70–73 to 91–95)	+
Rusanen 2011	USA	21,123	50–60	17yrs	+
Sabia 2008	England	5388	35–55	85–88 to 97–99	+
Sabia 2009	England	5123	Mean 56yrs	5yrs	+
Satoh 2006	Japan	2,764	35–44	10yrs	+
Sairenchi 2004	Japan	39,528 men and 88,613 women	40–79 (sub group: 40–59)	93–02	+
Shaper 2003	Britain	7735	40–59	22yrs (‘78-‘00)	++
Sobue 2002	Japan	91,738	40–69	9yrs	++
Stevens 2009	England Scotland	1.3 million	50–60 (women)	5-9yrs	++
Strand 2013	Norway	48,793	35–50	35yrs	++
Strandberg 2008	Finland	1658	40–55	26yrs	+
Szoeke 2006	Australia	438	46–52	11 years	+
Tyas 2003	Hawaii	3734	(mid-life)	(‘65–‘71) and (‘91–’96)	+
Whitmer 2005	USA	8,845	40–44	27yrs (‘64-‘03)	+
Wilcox 2006	USA	5820 Japanese American men	Mean 54yrs (45–68)	Up to 40yrs	++
Wannamethe 2001	UK	7735	40–59	16.8 yrs	+
**Alcohol**					
Anttila 2004	Finland	632 women and 386 men	Mean age 48.3 yrs	1972–77 to 1998	+
Beulens 2007	Netherlands	1,417	49–70 yrs	1993–97 to 2005	++
Elwood 2013	Caerphilly, UK	1,320 men	45–59	1979–04	+
Emberson 2005	England,Wales, and Scotland	7,735	40–59	1978/1980 to 1998/2000	++
Englund 2013	Sweden	778	49–61	85–08	+
Flood 2008	USA	49238	Older than 50 y	1995–1996 to 2000	++
Iso 2004	Japan	19 544	40–59	1990–2000	+
Lin 2005	Japan	110,792	40 to 79 years	1988–1990 to 1999	++
Moayyeri 2009	UK	25311	40–75 years	1993–1997 to 2007	+
Ostbye 2002	US	HRS study: 7,845 people	HRS study: ages 51–61 yrs	HRS: 92–98	-
Otani 2003	Japan	90004	40–59 Cohort 1. 40–69 Cohort 2	Cohort I. After January 1, 1990–1999. Cohort II. January 1, 1993–1994–1999	+
Qiu 2003	China	50069	40–80+	1994/1996-2000	+
Sabia 2009	England	5,123	Mean age 56 yrs	97–99 to 02–04	+
Sabia 2011	France	4073 men	Ages 40–50 for men and 35–50 for women	10 yrs (1992 to 2002–04)	+
Stevens 2009	England and Scotland	1.29 million women		96–01 to 05–07 Mean yrs of follow-up: 7.2 for cancer incidence; 8.9 for mortality	+
Sun 2011	US	13,894	70+	84–00	++
Tabak 2001	Finland, Italy, Netherlands	2,953 men (Finland: 1,186 men; Italy: 1,183; Netherlands: 667)	40–59	20 yrs (1965–70 to 1990)	-
Virta 2010	Finland	1,486	Mean age in 1981: 51.7 yrs (SD: 6.1)	1975–81 to 1999–07 (mean follow-up: 22.8 yrs.)	++
Wannamethee 2002	England,Wales, and Scotland	7157	40-59yrs	(‘78–’80)-(2000)	+
Waki 2005	Japan	28,893	40–59 yrs	10 yrs (baseline: 1990)	+
Wannamethee 2003	England, Wales, Scotland	7,608 men	40–59 yrs	1978–80 to 1983–85	+
Wang 2010	US	19,220	38–89 yrs	12.9 yrs. follow-up (baseline: 1992–95)	+
Willcox 2006	Island of Oahu	5820	45-68yrs	Not reported	++
Xu 2010	South East Queensland, Australia	564	45–60 yrs	2001–06	-
**Weight Change/Weight Cycling**					
Field 2009	United States	44842	30–55	16 years	+
Langlois 2001	United States	2180	50–74	22 years	++
Ravona-Springer 2013	Israel	10000	40–70	36 years	+
Waring 2010	United States	1577	40–50	11 years	++
Agrigoroaei 2011	United States	4995	33–84	9–10 years	++
Elwood 2013	Caerphilly, UK	1,320 men	45–59	1979–04	+
King 2007	United States	15708	45–64	11–13 years	++
**Leisure/Cognitive Activity/Social Networks**					
Bielak 2012	Australia	7152	20–24: 2404, 40–44: 2530, 60:64: 2551	7 years	++
Britton 2008	UK (England)	5823	35–55	17 years	+
Friedland 2001	United States	193 cases/358 controls (for total study, not reported for 40–59 year olds)	40–59	>12 (not fully reported)	-
Holtzman 2004	United States	354	50+	12.4 years	++
Kareholt 2011	Sweden	1643	57.4	20+ years	++
Raikkonen 2001	United States	541	42–50	9.2 years	++

**Guide: i**. Data is from multivariate models. Where multiple models have been reported data from the most adjusted (or most relevant) model has been used. **ii. Results**: + = significant positive association, – = significant inverse association, 0 = no significant association; √ = study that shows improved outcomes; X = study that shows poorer outcomes. **iii. Quality of study [++/+/-]**. ++ = high quality; + = moderate quality; - = low quality.

Note 1: A positive association (+) with PA is the better outcome

Note: 2 Physical inactivity where multiple outcomes have reported the most adjusted data in this table; sig = p</ = 0.05; ns = not significant (p>0.05)

Note 3: A positive association (+) with smoking is a worst outcome

Note 4: A positive association (+) with alcohol is the worst outcome

Longitudinal associations have been tabulated and synthesised narratively. Data specific to health inequalities has been summarised separately. Due to the methodological and statistical heterogeneity it was not appropriate to conduct a meta-analysis.

## Results

### Search Results

The searches for primary studies and the grey literature located 10,338 articles after removing duplicates, 567 of which had relevant titles and abstracts. In total, 164 observational longitudinal cohort studies were included in the review (Fig 1). The behavioural risk factors for which we found published data in mid-life with relevant outcomes in later life were: physical activity and inactivity; diet; tobacco consumption; alcohol; weight change or weight cycling; leisure, cognitive activity or social networks; and combinations of the above. Studies of behaviour related to hearing or vision were sought, but none met the inclusion criteria.

### Quality

Overall, the quality of studies is good (most studies were rated as high or moderate quality). Summary quality scores can be found in Table 1. Full quality assessments can be found in S2 Table.

### Characteristics of included studies

Summary characteristics for studies by health behaviours are listed in Table 1. Forty-five papers were found relating to mid-life physical activity (PA) or inactivity, 48 for diet, 57 for smoking and 24 for alcohol. Four studies were included that reported an association between weight change patterns in mid-life and later outcomes. Three studies reported data for combinations of lifestyle behaviours. Four studies were found that examined the relationship between mid-life leisure/social activities and DDF outcomes. The data relating studies found and health behaviour is reported in Table 2.

**Table 2:** Exposure and Outcomes.

	Physical activity and physical inactivity	Diet	Smoking	Alcohol	Weight change and cycling	Combined	Leisure/cognitive activity/social networks
Healthy Ageing / Quality of Life / Well-being	[30]; [39]; [46]; [59]; [31]; [38]; [42]; [37]; [32]	[71]; [30]; [115]; [112]; [116]; [72]	[120]; [30]; [163]; [121]; [147]; [122]; [148]; [207]; [209]; [37]; [118]	[46]; [32]; [173]; [174]	No evidence was identified	No evidence was identified	[30]
Disability / Frailty	[34]; [41]; [40]; [49]; [33]; [35]; [37]; [36]; [43]	[60]; [73]; [117]	[120]; [40]; [59]; [43]	[46]; [40]; [122]; [37];	[177]	[46]	[181]
Dementia & Cognition	[44]; [48]; [47]; [133]; [46]; [131]; [50]; [45]; [51]; [52]	[46]; [74]; [80]; [76]; [79]; [78]; [81]; [52]	[26]; [126]; [134]; [125]; [129]; [52]; [132]; [127]; [124]	[165]; [46]; [52]; [166]; [164];	[178]	[184]; [46]	[181]; [49]; [182]; [183]
Overall mortality	[46]; [57]; [54]; [53]; [55]; [161]; [56]	[71]; [46]; [82]; [75]; [85]; [84]; [83]	[140]; [137]; [152]; [139]; [134]; [132]; [118]; [119]	[46]; [167]; [168]; [119];	[179]	[46]	No evidence was identified
Cardiovascular (CVD) Outcomes	[46]; [57]; [62]; [53]; [60]; [28]; [61]; [56]	[71]; [97]; [98]; [96]; [111]; [100];	[27]; [141]; [142]; [153]; [140]; [150]; [137]; [154]; [149]; [152]; [139]; [147]; [138]; [135]; [89]; [151]; [146];	[93]; [46]; [167]; [171]; [89]; [169]; [170];	No evidence was identified	[46]; [186]	[151]
Diabetes / Metabolic Syndrome	[65]; [63]; [64]; [66]	[46]; [106]; [66]; [104]; [105]; [102];	[63]; [149]; [158]; [155]; [66]; [156]; [68]	[46]; [172]	[180]	[46]	No evidence was identified
Cancer	[46]; [68]	[46]; [107]; [108]; [83]; [109]	[162]; [159]; [160]; [161];	[46]; [176]; [159]; [161];	No evidence was identified	[46]	No evidence was identified
Mental health	[69]; [70];	[114]; [113]; [70]	[120]	[70]	No evidence was identified	No evidence was identified	No evidence was identified

Exposures were measured a number of different ways in the included studies. For physical activity all studies used self-reports of activity except one, which used an accelerometer. Dietary data was also self-reported through interview and food questionnaires. For smoking, many studies used self-reports with questionnaires and interviews with a number of studies using biochemical testing to confirm smoking status. All studies assessing the impact of alcohol, weight change/weight cycling and leisure and social activities used self-administered lifestyle questionnaires and interviews. The outcome and exposure measures are comprehensively reported in S4 Table.

Only four studies explicitly examining health inequalities were identified: three looking at gender or ethnicity [26–28] and one at lower socioeconomic status groups [29].

#### Summary of health behaviours and outcomes

A full summary of studies, exposures and outcomes are reported in S3 Table. An overview of reported outcomes in relation to health behaviours is shown in Tables 3 and 4. Due to the large volume of data found only the main findings are reported below.

**Table 3:** Overall Summary of Studies.

Results:								
Successful ageing	Disability and frailty	Dementia	Total mortality	CVD outcomes (events and mortality)	Diabetes (MetS)	Cancer (and cancer mortality)	Other chronic diseases	Mental health
**Physical Activity**								
√√√	√√√√√ 0	√√√√√√ 00	√√√√√	√√√√√√√√√	√√√√√	√√ X 000		√ 0
**Physical Inactivity**								
	0		0	X0				
**Tobacco**								
XXX	XX (mobility) 0X0X (fract)	XXXXXX (dementia) 00XXX (cognition)	X(XXX)XXX √√√√√√ (Ex-smokers)	XXXXXX0 (mortality) XXXXXXXXXXX0 (CVD)	XXX0 (Dia) X0 (MetS)	XXXXXX (lung, pancreatic,colorectal,cancer)	X (kidney disease) 0 (ex smoker)	No studies
**Smokeless Tobacco**								
					√ (insulin, weight)			
**Alcohol**								
√X	X (ADL)0X (fract)	X0 (dementia APOE4) 0 (cognition) XX (Abstainers (compared to mod or infreq) XXX Heavy drinkers (APOE4 compared to non-drinkers)	X	000 XX (Heavy cf occasional) √ (Reg cf occasional)	X (Diabetes—mod or high) X0√ (MetS)	000X	0 (COPD)	√
**Weight Change**								
	X (hip fracture) Weight loss of > 10% from max	X Weight variability	0 Weight cycling		X Weight cycling when OW at midlife			
**Leisure, Cognitive Activities and Social Network**								
0	0	√ (dementia) √√√ (cognition)						
**Combined Lifestyle**								
		√0 (cog)	√ √	√0	0	0		

Key: 0 = no significant association; √ = study that shows improved outcomes; X = study that shows poorer outcomes

**Table 4:** Overall Summary of Studies of Diet and Dietary Component.

Results:									
Diet or components of diet	Successful ageing	Disability & frailty	Dementia	Total mortality	CVD outcomes	Diabetes (MetS)	Cancer	Other chronic diseases	Mental health
Healthy dietary pattern	√√	√				√	0		√
Mediterranean diet	√				√√				√
Western diet	X								
Fruit and vegetables		√ 0 0	√ 0	√√ 0	√ 0	0	0 0	√	
Fat (saturated)		X	X		0	X			
Fat (polyunsaturated)			√		0				
Fat (monounsat)					0				
Fat (total)					0		X		
Fish				X	√ 0	√ women 0 men		√	
Meat		√ (>1 per 2 d)			√ (1-2/wk)				
Red and processed meat					X	X	X X		
Coffee		0	√ 0		X (heavy)	√√		√√	0
Tea								√	0
Caffeine								√	0
Milk							√		
Salt							X		
Glycaemic index/GL					√0				
Protein					0		0		
Chocolate					√ (1–3 times/month only)				
Fibre							0		
Micronutrients			0				√		0
Flavonoids			0		√		0		

Key: 0 = no significant association; √ = study that shows improved outcomes; X = study that shows poorer outcomes

### The impact of physical activity (PA) and physical inactivity (PI)

Summary data for physical activity, physical inactivity and sedentary behaviour studies, including study location, duration, period of follow-up, effect sizes, and quality scores are reported in Table 3. Note that we found no studies specifically looking at the consequences of physical inactivity or sedentary behaviour for dementia and cognition outcomes, diabetes/metabolic syndrome, or mental health.

#### Healthy ageing and well-being

There is consistent evidence [30–32] that PA in mid-life is positively associated with healthy and successful ageing outcomes. Healthy ageing or successful survival was defined in the three included studies as having no history of major chronic diseases and no cognitive impairment, physical impairment, or mental health limitations. No studies specifically aimed to assess physical inactivity; most studies reported physical activity in quintiles so reported lower levels of PA indirectly. No studies we found assessed quality of life.

#### Disability/frailty

There is consistent evidence that PA in mid-life is related to more positive outcomes in terms of disability and frailty in later life. Five of the six studies reported beneficial associations between mid-life PA and physical mobility [33] or physical functioning [34] or disability [35–37]. One study [38] reported no significant association with disability. Another study [39] found no association between inactivity in leisure time PA in mid-life and disability at age 75.

Three studies reported on the association between mid-life PA and bone fractures or bone health. One study reported less risk of hip or wrist fractures [40,41]. One reported improved bone mineral density in those who took part in PA in mid-life [42], and another reported no significant association with the risk of osteoarthritis [43].

#### Dementia & cognition

There is consistent evidence that PA in mid-life is associated with lower risk of dementia or better cognitive function in later life. Of the six prospective studies reporting on dementia or Alzheimer’s disease, four studies reported a significant beneficial association [44–47] and four studies non-significant associations [48–51]. Two studies found a significant inverse association between light and regular PA, but not for heavy PA [47,49]. Two studies found a positive association between mid-life PA and improved cognitive function in later life [34,52].

#### Overall mortality

There is consistent evidence [46,53–56] that regular, moderate and high intensity PA in mid-life is related to lower mortality in later life. One study [46,56] reports lower all-cause mortality but another [57] found no significant relationship between leisure time inactivity in mid-life and all-cause mortality.

#### Cardiovascular outcomes

There is strong evidence [28,46,53,54,58–62] of the beneficial effect of mid-life PA on CVD events or mortality. Six papers reported a significant association between mid-life PA and stroke [59], CVD risk [56,62], coronary heart disease (CHD) events [54,56,60], myocardial infarction [28], ischaemic heart disease [54] and three papers reported lower CVD-related mortality from stroke [61], CHD [56] and CVD [53] related to PA in mid-life.

Another [57] found a significant positive relationship between a single item measure of leisure time inactivity in mid-life and CVD mortality. However, no significant association was found when an index measure of leisure time physical activity was used.

#### Diabetes/metabolic syndrome

There is some consistent evidence that PA in mid-life is related to lower incidence of diabetes in later life [46,60,63,64]. In addition, three studies reported a beneficial association between mid-life PA and diabetes preconditions, two reported metabolic syndrome [63,65] and one reported insulin sensitivity [66].

#### Impact on cancer

The evidence relating to the associations between PA in mid-life and cancer is mixed. Four studies [46,53,66,67] reported longitudinal associations between mid-life physical activity and cancer or cancer mortality; however no significant relationship between mid-life PA and incident and fatal pancreatic cancer [67], lung, stomach, colorectal, lymphatic/hematopoietic cancers [68], or cancer mortality [53,68] was observed. One study [68] found lower rates of total cancer, upper digestive tract cancers (oral, oesophagus, stomach cancer) in those who participated in moderate or vigorous PA at mid-life, and increased risk of bladder cancer in those who participated in vigorous exercise compared to those who did not. Conversely, total cancer mortality was lower in those who took part in moderate or high levels of PA [46].

#### Impact on mental health

The evidence for an association between mid-life PA and mental health is inconclusive. One prospective cohort study [69] reported less risk of anxiety and/or depression for heavy PA at five-year follow-up but not at 10 years. Another study [70] found no significant association between mental well-being, including anxiety and depression and mid-life PA.

### The impact of overall diet and dietary patterns

Summary data, including study location, duration, period of follow-up, effect sizes, and quality scores for overall diet and dietary patterns is reported in Table 4.

#### Healthy ageing/quality-of-life/well-being

There is consistent evidence [30,71,72] that a ‘healthy’ diet in mid-life is related to healthy and successful ageing. A Mediterranean type diet is associated with more successful ageing [72]. A Western dietary pattern (characterised by high intakes of fried and sweet food, processed food and red meat, refined grains, and high-fat dairy products) was associated with less successful ageing. In these studies healthy ageing is defined as no major chronic diseases or major impairments in cognitive or physical function or mental health. No studies we found assessed quality of life or wellbeing.

#### Disability/frailty

There is limited evidence that ‘healthy’ diet or dietary patterns in mid-life is related to better functioning. One study reported associations between mid-life diet and ADL [73], in which men who ate meat at least once every two days or more were less likely to have impairment in ADL.

#### Dementia & cognition

There is limited evidence that ‘healthy’ diet or dietary patterns in mid-life is related to dementia and cognitive functioning in later life. Two studies reported beneficial associations between fruit and vegetable intake in mid-life and dementia [46,74]. Two studies examined relationships between coffee consumption in mid-life and dementia [75–77]. One study reported that moderate coffee consumption was associated with lower risk of dementia, but not tea drinking [75,77]. Another found no significant association with dementia [76]. One reported a non-significant relationship between mid-life dietary antioxidant intake, flavonoids and dementia [78]. A greater risk of dementia was also reported in those consuming moderate compared to low amounts of saturated fat compared to those consuming moderate compared to low amounts of polyunsaturated fat [79].

One study reported better cognitive outcomes for those consuming a ‘healthy pattern’ diet [80], characterised as consumption of fruit, whole grains, vegetables, and negatively correlated with meat and poultry, refined grains, animal fat, and processed meat. More than two portions of fruit and vegetables a day was associated with better cognitive performance [52]; however, two studies reported no significant association with cognitive function [46,81]. Higher levels of total or saturated fat were associated with greater cognitive impairment in later life [82].

#### Overall mortality

The evidence on the impact of diet/dietary patterns on mortality is mixed. Three studies reported associations between fruit and/or vegetable intake and total mortality. One reported significantly lower risk of death in people consuming higher levels of fruit and vegetables at mid-life [83], another reported significantly lower overall mortality for each increase of 20g/day in vegetable intake [84]. Associations between >3 portions fruit and vegetables/day were not significant in one study [46]. There is limited evidence for increased mortality with greater fish consumption in those at high risk of CHD [85].

#### Cardiovascular outcomes

Evidence suggests a healthy diet has a positive impact on cardiovascular outcomes. Two studies reported beneficial effects of a Mediterranean diet pattern with lower risk of CHD events and mortality [86,87]. One reported fruit and vegetable intake was associated with lower CVD mortality [83]; however another [46] reported non-significant associations. Findings from one study [88] suggest that high intakes of flavonoids may be associated with decreased risk of ischaemic stroke and possibly with reduced CVD mortality.

One study [89] reported lower risk of cerebrovascular disease in those consuming meat one-two times a month compared to those consuming no meat or those who ate meat more than once a week. A lower risk of CHD events and mortality was found in women when meat was replaced with fish [90]. Another study reported no significant association between fatty fish consumption and heart failure but lower risk of heart failure in those consuming marine omega 3 fatty acids once a week [91]. Higher intakes of marine omega 3 fatty acids were not significantly associated with heart failure.

Heavier coffee drinkers showed a higher risk of CHD events and mortality compared to moderate coffee drinkers [92]. Associations were not significant for light or no coffee drinking compared to moderate intake.

A higher risk of CVD was reported for those consuming diets with the highest compared to the lowest dietary glycaemic index and glycaemic load [93]. In another study associations between glycaemic index and glycaemic load, and CVD events were not significant [91]. One study found no significant associations between mid-life protein intake and ischemic heart disease [94].

One study reported no significant associations between total, saturated, monounsaturated or polyunsaturated fat and fatal or non-fatal cardiovascular events [95].

Fruit and possibly vitamin E intake may also have protective effects against COPD [96]. In men, diets higher in fruits and vegetables and lower in meats (except fish) may reduce the risk of developing high blood pressure [97]. In women results of one study [98] suggest that higher intake of dietary magnesium may have a modest effect on the development of hypertension. Intakes of low-fat dairy products, calcium, and vitamin D were each inversely associated with risk of hypertension in middle-aged and older women [99]. While higher intake of all fruits (but not all vegetables) was significantly associated with reduced risk of hypertension in women [100]; higher intake of saturated fats, monounsaturated fats, and trans fats were each associated with increased risk of hypertension among middle-aged and older women [101].

#### Diabetes/metabolic syndrome

The evidence for the impact of diet on diabetes and metabolic syndrome is mixed. One study reported that a dietary pattern low in staples and high in milk was associated with lower risk of diabetes [102]. Another reported no statistically significant association between fruit and vegetables and diabetes [46]. Higher saturated fat intake at mid-life was associated with lower insulin sensitivity [66]. One study [103] reported lower risk of diabetes in women eating moderate and high amounts of fish and shellfish with a significant trend with greater fish and shellfish intake. One study found increased risk of diabetes in those consuming higher levels of red and processed meat with a significant trend from lower to higher intake [104].

Two studies reported lower risk of diabetes with coffee intake. One conducted on men and women found a significant trend towards lower risk for diabetes with increasing coffee consumption [105]. The other [106] reported a significant inverse relationship for women consuming three or more cups of coffee a day with a significant trend. In men only one-two cups/day was significantly associated with lower risk of diabetes but there was also a significant inverse trend between coffee consumption and diabetes.

#### Impact on cancer

There is inconsistent evidence regarding the impact of diet/dietary patterns on cancers. One study [107] found no clear associations between four dietary patterns and cancer. Two studies reported no significant associations between fruit and vegetables and cancer incidence or mortality [46,83]. One study reported lower risk of colorectal cancer with consumption of fruit, dietary calcium, vitamin A and vitamin C; but higher risk of colorectal cancer with consumption of red and processed meat [108]. The study reported lower incidence of colorectal and rectal cancer in those consuming high volumes of milk but found no significant associations between protein and fibre intake with colorectal cancer. Another study [109] found a significant association between high salt intake and higher risk of gastric cancer in men but not women. One [110] reported lower risk of heart failure when chocolate was consumed 1–3 times month compared to no chocolate consumption. There is no significant relationship reported between flavonoids and total cancer or site-specific cancers [111].

#### Impact on mental health

There is limited evidence regarding the impact on mental health. One study [112] reported less psychological distress in those with the highest compared to lowest adherence to the Mediterranean diet. Light or heavy coffee consumption was also associated with lower risk of severe depression [113]. However, one [70] reported no significant association between coffee drinking and anxiety, depression or psychological symptoms, but reported lower scores on the mental health scale on the SF-36 general health questionnaire. There was no association with tea or caffeine intake. One study [114] found no association between dietary zinc intake and depression.

#### Impact on other conditions

There is limited evidence regarding the impact of diet on other outcomes. One study reported intake of fruits and vegetables may reduce long-term risk of weight gain in middle-aged women [115]. Another study in women reported that weight gain was inversely associated with the intake of high-fibre, wholegrain foods but positively related to the intake of refined-grain foods [116]. Higher coffee and caffeine intake was also associated with a significantly lower incidence of Parkinson’s Disease [117].

### The impact of tobacco

Summary data, including study location, duration, period of follow-up, effect sizes, and quality scores for tobacco is reported in Table 3.

#### Healthy ageing/quality-of-life/well-being

There is consistent evidence demonstrating a detrimental association between smoking and healthy ageing, quality of life or well-being outcomes. One study [30] showed that not smoking was related to a favourable older life; another [118] found that never-smokers lived longer than heavy smokers, and their extra years were of better quality. Health-related quality of life deteriorated with an increase in daily cigarettes smoked in a dose-dependent manner. The third [119] suggests that ‘ever smoking’ is associated with overall survival and a borderline association with exceptional survival (i.e. free of a set of major diseases and impairments).

#### Disability/frailty

There is consistent evidence demonstrating the dose-response relationship between smoking and impaired mobility. One study [120] suggests that a history of smoking, especially heavy smoking, with or without quitting, is associated with an earlier onset, and more rapid development, of mobility problems during the transition from middle age to old age. Another [37] showed a consistent adverse dose-response relationship between smoking and ill-health (i.e. disability, impaired mobility, health care utilisation, self-reported health).

There is inconsistent evidence demonstrating associations between smoking and low energy fractures. One [121] showed that among women, smokers had a higher risk for vertebral fractures than non-smokers. Among men, smokers had a greater risk for low energy fractures, vertebral fractures, proximal humerus fractures, and hip fractures. Other papers showed no association between smoking and wrist fractures [40] or osteoporotic fractures [122]. One study [43,123] found a positive association between smoking and risk of osteoarthritis. No studies we found assessed frailty.

#### Dementia & cognition

There is strong evidence demonstrating the association between smoking in mid-life and dementia, or cognitive decline, in later life. The association between smoking and specific types of dementia is less clear. In most studies smoking was strongly associated with dementia [26,124–127], subsequent risk of hospitalisation with dementia [26], and with being diagnosed with dementia [128]. Two studies [52,129] showed an association between smoking and cognition. One [51] showed that smoking in middle age is associated with memory deficit and decline in reasoning abilities; long-term ex-smokers (compared to current smokers and recent ex-smokers) are less likely to have cognitive deficits in memory, vocabulary, and verbal fluency. Another [130] reported greater decline in memory function, cognitive flexibility, and cognitive function among smokers. The declines in all cognitive domains were larger with increasing number of pack-years smoked. Only one study [131] found that smoking at baseline was not associated with change in cognitive decline whilst another [132] looking at dementia death failed to demonstrate an association with smoking. In another study [133], mid-life smoking was associated with an increased rate of progression of vascular brain injury, global and hippocampal atrophy.

#### Overall mortality

There is strong evidence demonstrating a dose-response relationship between smoking in mid-life and total mortality. Compared to never smokers, smokers are at increased risk of mortality. Five studies [118,134–137] showed that current smokers showed the highest risks of total mortality with a dose-response relationship with increasing number of cigarettes smoked. One [135] showed that men smoking persistently were most at risk, while those who persisted in quitting had no increased risk of death compared with non-smokers. Another [138] concluded that smokers across the entire range of pulmonary function may increase their expectation of lifespan by giving up smoking. Finally, one study [139] showed that compared to current smokers, never smokers, long-term quitters and new quitters had a decreased risk of all-cause mortality; the same association was observed for never smokers and long-term quitters for other non-lung cancer mortality. Compared to those who maintained their smoking habit, individuals that reduced or quit smoking had a decreased risk of all-cause mortality [140].

#### Cardiovascular outcomes

Smoking or having smoked is consistently associated with increased risk of cardiovascular mortality and CVD. Six studies provide evidence of a strong association between smoking and cardiovascular mortality [137,139–143], with current smokers being more likely to die from cardiovascular events compared to non-smokers. Only one study didn’t support this association [144]. Other studies provide evidence of a strong association between smoking and cardiovascular events and outcomes [59,134,145–153]. The highest risks for both CHD events and stroke were seen in heavy current smokers [134]. Smoking also increases the risk of CHD in men of all APOE genotypes but particularly in men carrying the e4 allele [145,154]. Only one showed no association between smoking and CHD [146]; all other studies have shown an association between smoking and stroke [59,146,147] and myocardial infarction [148].

#### Diabetes/metabolic syndrome

Cigarette smoking is an independent and modifiable risk factor for type 2 diabetes; however, the evidence for insulin sensitivity and metabolic syndrome is not sufficient to conclude. Three studies [68,155,156], demonstrated cigarette smoking is an independent and modifiable risk factor for type 2 diabetes and one did not [157]. One [68] reported the risk of diabetes in those who switched from smoking cigarettes to pipe or cigars remained equal to the risk in continuing cigarette smokers. Other studies showed that smoking is a risk factor for type 2 diabetes independently of BMI and physical activity [155]; however another [157] found that smoking was associated with the metabolic syndrome but not diabetes. Finally, one study [66] found no significant association with insulin sensitivity and smoking in men.

There is some evidence to suggest that the use of smokeless tobacco in mid-life is related to type 2 diabetes [158]. The use of smokeless tobacco was associated with low insulin response but not low insulin sensitivity.

#### Impact on cancer

Evidence showed a consistent association between smoking and cancer with a dose-response effect. The dose-response and exposure association seems to depend on the type of cancer considered [134,159,160]; current cigarette smokers showed the highest risk of total cancer with a strong dose-response effect. In a study [161] looking specifically at pancreatic cancer in women, pancreatic cancer incidence was greater in current smokers, with the risk increasing with the number of cigarettes smoked. One [159] showed that smoking was significantly associated with colorectal cancer in men and not significantly in women. Current cigarette smoking is associated with an elevated lung cancer risk approximately 10- to 20-fold higher for squamous cell and small cell carcinoma and 2- to 3-fold higher for adenocarcinoma in both men and women [160]. Another [162] confirmed the strong association between smoking and cancer, and cancer-related mortality. Compared to current smokers, never smokers, long-term quitters and new quitters also had a decreased risk of lung cancer mortality [139].

#### Impact on other conditions

One study reported that being a smoker was significantly associated with weight loss [163].

No studies were identified which assessed the impact on mental health.

### The impact of alcohol

Summary data, including study location, duration, period of follow-up, effect sizes, and quality scores for alcohol is reported in Table 3.

#### Disability/frailty

Two studies reported higher odds for ill-health and osteoporotic fractures among alcohol drinkers compared to non-drinkers, while one study found no link between alcohol intake and wrist fractures [40]. Conversely, the risk for any incident osteoporotic fracture was reported to be higher among male alcohol users compared to male teetotallers [122]. A large study [37] showed that respondents with a past drinking problem had the highest odds for ill-health in terms of ADL dependence, difficulty climbing stairs, difficulty walking, poor health, and hospitalization. No studies we found assessed frailty.

#### Dementia & cognition

There is consistent evidence demonstrating an association between alcohol abstinence and/or heavy drinking and cognitive impairment [52,164–166]. Compared to moderate alcohol intake, alcohol abstinence was associated with a higher risk of poor executive function and poor memory [52]. One study reported no association with impairment cognition or dementia [46].

#### Overall mortality

Evidence was mixed for associations between alcohol consumption and all-cause mortality. One study [119] reported excessive alcohol consumption was associated with overall and exceptional survival at age 85 years. One [167] showed that alcohol intake was associated with a higher risk for all-cause mortality. Another [168] showed that excessive mortality was significantly associated with heavy drinking among men diagnosed with cancer and cardiovascular disease in this time period. One reported no association between ethanol consumption and chronic obstructive pulmonary disease mortality [169].

#### Cardiovascular outcomes

The evidence regarding alcohol use and cardiovascular outcomes is inconsistent. Three studies [167,170,171] showed a significant association between alcohol intake and cardiovascular outcomes. Regular drinkers had a significantly lower risk of major CHD events, CHD deaths, and CVD deaths in comparison with occasional drinkers after full adjustment for lifestyle characteristics and pre-existing disease [170]. Heavy drinkers had a higher risk of both major CHD and stroke compared to occasional drinkers [167,171]. No significant associations were observed between alcohol intake and cardiovascular outcomes in three studies (e.g., disease development, death) [46,93,144].

#### Diabetes/metabolic syndrome

Findings were consistent with respect to the influence of alcohol intake on diabetes/metabolic syndrome. Three studies [172–174] found significant positive associations between alcohol intake levels and diabetes/metabolic syndrome, while one study [46] did not find an association.

#### Impact on cancer

There is mixed evidence demonstrating the absence of an association between alcohol intake and cancer. Three studies [46,175,176] did not find significant associations between alcohol intake and incident and fatal pancreatic cancer; cancer in general; and colorectal cancer, respectively. In contrast, another study [159] showed a significantly higher risk for colorectal cancer among men who drink alcohol compared to non-drinkers. That study [159] reported that approximately half of the reported colorectal cancer cases may be preventable by tobacco and alcohol controls in middle-aged Japanese men; however the portion attributable to alcohol is not known.

#### Impact on mental health

Evidence on the impact on mental health is limited. One study [70] of women indicated that past alcohol drinkers had lower anxiety than non-drinkers.

No studies were identified which assessed the impact on healthy ageing, quality of life, well-being.

### The impact of weight change/weight cycling

Summary data, including study location, duration, period of follow-up, effect sizes, and quality scores for weight change/weight cycling is reported in Table 3.

#### Disability/frailty

There is limited evidence that weight loss of more than 10% of max body weight in mid-life is related to hip fractures [177]. The impact was statistically significant for those aged 50–64 and 65–74 years who were weight cycling (as defined by study authors) in mid-life.

#### Dementia and cognition

There is limited evidence to suggest that weight change in mid-life is related to dementia [178]. Those in the highest two quartiles of weight change had a significantly higher risk of dementia, independent of the direction of weight change.

#### Overall mortality

One study reported no association between weight cycling in mid-life and mortality [179].

#### Diabetes/metabolic syndrome

One study [180] reported longitudinal associations between weight change/weight cycling and diabetes. The impact was not significant for weight change or weight cycling but overall weight status was more important in those who were overweight or obese at increased risk of diabetes.

No studies were identified which assessed the impact on healthy ageing, quality of life, well-being, cardiovascular outcomes, cancer or mental health.

### The impact of leisure, cognitive activities and social network

Summary data, including study location, duration, period of follow-up, effect sizes, and quality scores from leisure, cognitive activities and social network studies is reported in Table 3.

#### Healthy ageing/quality-of-life/well-being

There is little evidence to determine if leisure and cognitive activities, and an individual’s social network in mid-life are related to successful aging. One study [30] reported longitudinal associations between leisure, cognitive activities, social network and successful aging in both men and women. While there was a beneficial association these were non-significant. No studies we found assessed quality of life or well-being.

#### Dementia and cognition

There is some evidence that leisure and cognitive activities, and an individual’s social network in mid-life is related to a lower risk of cognitive decline in later life. One study [49] reported an association between diversity and intensity of participation in intellectual, physical and passive activities and lower risk of AD. Three studies [181–183] reported longitudinal associations between leisure, cognitive activities, social network and dementia or cognitive impairment. All studies reported a beneficial association between mid-life factors and dementia or cognitive decline in later life; however in one study the associations were non-significant for some activities including social, organisational and physical activities [183]. There is limited evidence for a prospective association between mid-life mental distress and dementia [184].

#### Cardiovascular outcomes

We found no evidence on the association between leisure activity, cognitive activity or social networks in mid-life and late life cardiovascular outcomes apart from one study which found no correlation with reduced hypertension in women [151].

No studies were identified which assessed the impact of these behaviours on disability, frailty, overall mortality, diabetes, cancer or mental health.

### Combined behavioural risks

Summary data, including study location, duration, period of follow-up, effect size, and quality scores from combined lifestyle studies are reported in Table 3.

#### Dementia & cognition

There is limited evidence to suggest that combined healthy behaviours in mid-life is related to less risk of dementia in later life. Three studies [46,185,186] reported longitudinal associations between healthy behaviours and dementia or cognitive impairment; one study [185] reported that modifiable psychosocial and behavioural factors have protective effects on cognition and another [164] reported that higher levels of consumption was associated with higher risk of cognitive impairment, in particular that binge drinking was found to be an independent risk factor for cognitive impairment; however in one study the associations were non-significant[46].

#### Overall mortality

There is evidence to suggest that reducing unhealthy behaviours or adopting a healthier lifestyle in mid-life is related to reduced death. Two cohort studies [46,187] reported a significant negative association between number of unhealthy behaviours and mortality.

#### Cardiovascular outcomes

There is inconsistent evidence that reducing unhealthy behaviours or adopting healthier behaviours in mid-life is related to reduced CVD. One study reported a significant negative association between number of unhealthy behaviours and CVD [187] while the other [46] reported no association. Importantly the combined behaviours in these two studies vary.

#### Diabetes/metabolic syndrome

There is limited evidence that association exists between reducing unhealthy combined lifestyle behaviours in mid-life are related to diabetes. One study examined the impact of combined lifestyle on diabetes; while there was a negative correlation this was non-significant [46].

#### Cancer

There is no evidence that association exists between reducing unhealthy combined lifestyle behaviours and cancer. One study examined the impact of combined lifestyle on cancer and there was no correlation [46].

No studies were identified which assessed the impact on healthy ageing, quality of life, well-being, disability, frailty or mental health.

### Disadvantaged and minority groups

For this review ‘disadvantaged and minority groups’ includes (but is not limited to) people of low socioeconomic status; ethnic minority groups; LGBT community groups; travellers; and other groups with protected characteristics under the equality and diversity legislation. One study examined associations between mid-life smoking and reported no difference in the risk of developing dementia by gender or ethnicity [26] or the risk of cardiovascular disease, total CHD or myocardial infarction by gender [143]. One [28] reported less risk of myocardial infarction in women participating in leisure time sports, however this result was based on a small sample size. High alcohol intake in lower SES groups is related to poorer cognitive performance[29]. No association between alcohol consumption and cognitive performance was found in those in intermediate or high socioeconomic groups. There is limited evidence and where evidence is available this is generally weak. Most of these groups also have substantially lower life expectancy than the general population, with fewer surviving into age groups of the highest risk.

## Discussion

This review provides an overview of the available evidence from high quality observational studies reporting on the association between specific behavioural risk factors, in mid-life, and successful ageing and the primary prevention or delay of DDF and non-communicable chronic conditions. The conclusions of individual cohort studies are consistent with existing knowledge and best practice for a range of specific non communicable disorders (for example, reducing alcohol and tobacco consumption, and increasing physical activity and intake of fruit and vegetables).

The findings that consistently emerge as being of societal importance in their range of potential impact are physical activity, which is beneficial, and smoking is consistently found to be associated with DDF and non-communicable chronic conditions. Other factors for which the evidence base is not as strong are diet, alcohol, weight change and social/leisure activities.

For Physical Activity there was consistent evidence within the 45 studies identified that mid-life PA is associated with better late life DDF and NCC outcomes. Only two studies specifically focused on physical inactivity. This finding is in alignment with previous research that suggests regular PA can reduce the risk of chronic disease and improve one’s health and well-being whereas a lack of physical activity leads to poorer health [188–190].

The review found a wealth of evidence from longitudinal cohort studies relating to the association between mid-life smoking and poorer DDF and NCC outcomes. The impact of tobacco is well known as previous research demonstrates that tobacco consumption is an important risk factor for CVD, cancer and hypertension, and is a major cause of premature mortality [191,192]. Whilst smoking cessation is associated with weight gain and a subsequent increase in risk of diabetes, the long-term benefits of giving up smoking outweigh the adverse effects of early weight gain.

There is some consistent evidence from 48 studies that healthy diets have beneficial effects on DDF and NCC outcomes and higher consumption of saturated fat or processed and red meat is associated with poorer ageing, DDF and NCC outcomes. Previous research has also demonstrated a strong relationship between diet, dietary patterns or nutrient intakes, and prevention and management of chronic diseases [193,194] including diabetes [195] and CVD [196].

Evidence from 24 studies specific to mid-life alcohol consumption was mixed and inconsistent with smaller effect sizes than for smoking and physical activity. Some studies reported negative outcomes (e.g. for dementia, mortality and cancer) and some more positive outcomes (e.g. for ageing and mental health). Previous research suggests that alcohol is a significant cause of mortality, morbidity and social problems, both in developed and in developing countries [197], causing an estimated 4% of the global disease burden including chronic diseases and certain cancers, as well as poor health in general [198–200].

Four studies were included that reported an association between weight change patterns in mid-life and later outcomes, including increased risk of hip fracture, mortality, diabetes and dementia. For issues specific to weight change or weight cycling it is known that being overweight increases the risks for many chronic medical conditions, including diabetes, heart disease, hypertension and stroke [201]. Modest reductions in weight can lead to important health benefits [67,202], while fatigue and unintentional weight loss can affect an individual’s physical, psychological, social and cognitive functioning [203,204]. Combinations of lifestyle behaviours were also protective factors in relation to cognitive function, diabetes, vascular disease, cancer, dementia and mortality.

There is little evidence on the impact of leisure, cognitive activities and social network and combined lifestyles. Studies that focused on disadvantaged groups have tended to be few in number and of lower quality indicating that this is a neglected research area.

## Limitations of the Evidence, Gaps

Limited evidence was found specifically relating to mid-life behaviours for leisure activities including cognitive activities and social networks, weight change and weight cycling and smokeless tobacco. While many diet-related studies were found they covered a broad range of diets and dietary components. There were some diets or dietary components for which studies specifically pertaining to mid-life was not found.

For the following exposures only one study was identified:

- Evidence of the impact of PA on stroke [61]; CHD [56,205]; CVD [53]; bone mineral density [42]; insulin sensitivity [66]; and risk of anxiety and/or depression [69].
- Evidence of the impact of diet, consumption of dietary calcium, vitamin A, vitamin C, zinc and flavonoids on CVD mortality [83], overall mortality [84], and psychological distress [206].
- Evidence on the impact of tobacco on total cancer [134]; pancreatic cancer [175]; colorectal cancer [159]; risks of lung cancer [207] and lung cancer mortality [139]; cancer related mortality [162]; quality of life [118]; psychological distress [37]; impaired mobility [120]; risk of chronic kidney condition [208]; and smokeless tobacco on type 2 diabetes [209]; weight and weight maintenance [210].
- Evidence on the impact of alcohol on major coronary heart disease and stroke [167]; ADL dependence, difficulty climbing stairs, difficulty walking, poor health, and hospitalization [37]; wrist fractures [40]; dementia [46]; COPD mortality [169]; incident and fatal pancreatic cancer [175,211]; cancer in general[46]; and colorectal cancer [176].

No studies were found which reported: physical inactivity and healthy ageing/quality of life/well-being, dementia, diabetes/metabolic syndrome, cancer or mental health; the relationship between alcohol consumption and healthy ageing/quality of life/well-being; weight change/weight cycling with healthy ageing/quality of life/well-being, CVD outcomes, cancer or mental health; impacts on disability/frailty, diabetes/metabolic syndrome, cancer, overall mortality, mental health or other chronic diseases; or the impact of combined lifestyle behaviours and healthy ageing/quality of life/well-being, disability/frailty, mental health or other chronic diseases; studies of behaviour related to hearing or vision. The challenge in assessing the impact of these exposures is a pronounced lack of data.

While no included studies reported relationship between mid-life physical activity and dementia, a recent meta-analysis [212] of prospective studies was published subsequently to the completion of the review and reported a fixed effects risk ratio of 0.61 (95% CI 0.52–0.73) indicating an overall reduction in risk of Alzheimer’s disease in physically active older adults.

There is also substantial heterogeneity in the measurement of exposures and outcomes included in this review making precise comparisons between studies problematic. While the review only includes longitudinal observational studies, which can show an association between mid-life risk factors and later life outcomes, associations from this type of study are not necessarily causal.

There was a paucity of data relating to disadvantaged groups covered by the equality and diversity legislation. While we found evidence assessing the risks of developing: dementia by gender or ethnicity [26]; CVD, total CHD or MI by gender [143], and in women [28] only, also alcohol intake in lower SES groups [29]. No longitudinal data was found relevant to other groups covered by the equality and diversity legislation such as LGBT groups or travellers. The absence of large-scale, high-quality research in these communities is problematic as there is a need for a more detailed and accurate picture of the disparities these communities face. Disadvantaged groups are significantly more likely to experience pervasive discrimination [213] and without clear evidence, the challenges faced are exacerbated. Whilst there have been some gains for visibility and equality for these communities, many disparities remain, particularly for women and the poorest (most vulnerable) in our society. Poorer health and well-being will persist, if high rates of discrimination are allowed to continue resulting in the experience of deeper inequality. Solutions to the disparities addressed above must focus both on reducing discrimination in general and improving conditions of living. Given that discrimination has a cumulative negative effect on health, there are plausible reasons for anticipating differences for disadvantaged groups or settings. These are the important considerations that should be made to ensure that inequities are reduced. There is therefore very little information about how mid-life behaviours in disadvantaged communities’ impact on population-level health outcomes in later life. More data is needed to understand how the behavioural risks are differentially modulated in these disadvantaged groups; with the aim of designing interventions that are fit for purpose.

## Limitations of the Review

The remit of this rapid review was specifically to identify mid-life behavioural risk factors for DDF outcomes and common NCCs in later life. Determinants of DDF over the whole life course were not included. Pragmatically, due to the wide scope of the review, the large amount of literature covering behavioural risk factors and the outcomes of interest, and the timescales for the review, the searches were focused on studies with mid-life-related terms in the title, abstract or related MeSH indexing to identify those studies that specifically aimed to report on mid-life exposure. The implication is that cohort studies that have followed individuals from mid- to late life and reported associations of interest without specifying mid-life terms in the title or abstract were not identified by the searches. This might explain some of the gaps in evidence and further work is on-going to address this limitation.

Although we conducted a rapid systematic review it is unlikely that our overall conclusions would vary greatly had a systematic review been conducted over a longer period of time [199]. Another strength is that unlike other rapid reviews a quality assessment of the included studies was undertaken reducing the limitations associated with the evidence synthesis process and results [200].

Also, only OECD countries are included, which may impact on the implications of findings within the broader global health context. However, few studies from non-OECD countries were found in the searches.

## Conclusion

The long-term impact of beneficial behavioural factors in middle to older age adults was greater chance of successful ageing and the primary prevention or delay of disability, dementia, frailty, and non-communicable chronic conditions. The exposures and health behaviours in mid-life identified in this review are important considerations both in identifying possible differential mechanisms for action (in order to reduce ill-health in the population) and the design of interventions to improve healthy behaviours.

## Supporting Information

S1 Table Excluded Studies. (DOCX)

S2 Table Quality Assessment. (DOCX)

S3 Table Included Study Overview. (DOCX)

S4 Table Data Extraction. (DOCX)

S5 Table PRISMA Checklist. (DOC)

S1 Text Study Protocol. (DOCX)

S2 Text Search Strategies. (DOCX)
