# Supplementary material for: Behavioural Risk Factors in Mid-Life Associated with Successful Ageing, Disability, Dementia and Frailty in Later Life: A Rapid Systematic Review
Source: PLoS One. 2016 Feb 4;11(2):e0144405. doi: 10.1371/journal.pone.0144405 (PMC4742275; doi:10.1371/journal.pone.0144405)
Supplement: S1 Table — (DOCX) [file pone.0144405.s001.docx]

Excluded Studies and Reason for Exclusion

| **Study** | **Reason excluded** |
| --- | --- |
| Abramson JL, Vaccarino V. (2002). Relationship between physical activity and inflammation among apparently healthy middle-aged and older US adults. Archives of Internal Medicine 162(11): 1286-1292. | X-sect, outcome is inflammation |
| Agardh EE, Ahlbom A, Andersson T, Efendic S, Grill V, Hallqvist J, et al. (2007). Socio-economic position at three points in life in association with type 2 diabetes and impaired glucose tolerance in middle-aged Swedish men and women. International Journal of Epidemiology 36(1): 84-92. | X-sect |
| Akbaraly TN, Kivimaki M, Shipley MJ, Tabak AG, Jokela M, Virtanen M, et al. (2010). Metabolic syndrome over 10 years and cognitive functioning in late midlife: the Whitehall II study. Diabetes Care 33(1): 84-89. | Not health behaviours (HB) |
| Akbaraly TN, Portet F, et al. (2009). Leisure activities and the risk of dementia in the elderly. Results from the Three-City Study. Neurology  73(11): 854-861. | Not midlife, analyses in older people |
| Albanese E, Hardy R, Wills A, Kuh D, Guralnik J, Richards M. (2012). No association between gain in body mass index across the life course and midlife cognitive function and cognitive reserve--the 1946 British Birth Cohort study. Alzheimer's & Dementia 8(6): 470-482. | Obesity is exposure, outcome cog fn at age 53 (<55y) |
| Alfred T, Ben-Shlomo Y, Cooper R, Hardy R, Deary IJ, Elliott J, et al. (2013). Genetic variants influencing biomarkers of nutrition are not associated with cognitive capability in middle-aged and older adults. Journal of Nutrition 143(5): 606-612. | Exposure not HB |
| Almeida, OP, Hulse GK, et al. (2002). Smoking as a risk factor for Alzheimer’s disease: contrasting evidence from a systematic review of case–control and cohort studies. Addiction 97: 15–28. | SR, not specifically midlife, look at again with SRs |
| Alter DA, Wijeysundera HC, Franklin B, Austin PC, Chong A, Oh PI, et al. (2012). Obesity, lifestyle risk-factors, and health service outcomes among healthy middle-aged adults in Canada. BMC Health Services Research 12: 238. | Exposure obesity, outcome HC costs |
| Anderson R, Anderson D, Hurst C. (2010). Modeling factors that influence exercise and dietary change among midlife Australian women: results from the Healthy Aging of Women Study. Maturitas 67(2): 151-158. | Not longitudinal, survey of midlife - review 1? |
| Anonymous (2003). Summaries for patients. Overweight and obesity by middle age are associated with shortened lifespan.[Original report in Ann Intern Med. 2003 Jan 7;138(1):24-32; PMID: 12513041]. Annals of Internal Medicine 138(1): I44. | Summary of Peeters paper |
| Anonymous (2013). Summaries for patients. The association between physical fitness and dementia.[Original report in Ann Intern Med. 2013 Feb 5;158(3):162-8; PMID: 23381040]. Annals of Internal Medicine 158(3): I-36. | Summary of DeFina 2013 paper |
| Ansari RM. (2009). Effect of physical activity and obesity on type 2 diabetes in a middle-aged population. Journal Of Environmental & Public Health 195285. | X-sect |
| Anstey KJ, Cherbuin N, Budge M, Young J. (2011). Body mass index in midlife and late-life as a risk factor for dementia: a meta-analysis of prospective studies. Obesity Reviews 12(5): e426-437. | SR, BMI as exposure |
| Anttila T, Helkala EL, Kivipelto M, Hallikainen M, Alhainen K, Heinonen H, et al. (2002). Midlife income, occupation, APOE status, and dementia: a population-based study. Neurology 59(6): 887-893. | Not HB |
| Arnlov J, Ingelsson E, Sundstrom J, Lind L. (2010). Impact of body mass index and the metabolic syndrome on the risk of cardiovascular disease and death in middle-aged men. Circulation 121(2): 230-236. | Obesity as exposure |
| Arnlov J, Sundstrom J, Ingelsson E, Lind L. (2011). Impact of BMI and the metabolic syndrome on the risk of diabetes in middle-aged men. Diabetes Care 34(1): 61-65. | Obesity as exposure |
| Arvanitakis Z, Wilson RS, et al. (2006).Diabetes mellitus and risk of Alzheimer disease and decline in cognitive function. Archives of Neurology 61(5):661-6. | Diabetes is exposure |
| Asia Pacific Cohort Studies Collaboration. (2003). Cholesterol, coronary heart disease, and stroke in the Asia Pacific region. International Journal of Epidemiology 32:563-72. | Not HB, cholesterol as exposure |
| Baker DW, Sudano JJ, Albert JM, Borawski EA, Dor A. (2001). Lack of health insurance and decline in overall health in late middle age. New England Journal of Medicine 345(15): 1106-1112. | Exposure is health insurance, outcome overall health, follow up 2 years |
| Barengo NC, Hu G, Lakka TA, Pekkarinen H, Nissinen A, Tuomilehto J. (2004). Low physical activity as a predictor for total and cardiovascular disease mortality in middle-aged men and women in Finland. European Heart Journal 25(24): 2204-2211. | Not cohort study, sequential X-sect |
| Barnes D, Yaffe K. (2011). The projected impact of risk factor reduction on alzheimer's disease prevalence. Alzheimer's and Dementia 1): S511. | Not specifically midlife but important paper. Include as SR? |
| Barnes DE, Yaffe K, Byers AL, McCormick M, Schaefer C, Whitmer RA. (2012). Midlife vs late-life depressive symptoms and risk of dementia: differential effects for Alzheimer disease and vascular dementia. Archives of General Psychiatry 69(5): 493-498. | Exposure is depression |
| Baron-Epel O, Azizi E. (2003). The association between counseling, sun protection, and early detection of skin cancer in middle-aged Israelis. Cancer Detection & Prevention 27(5): 338-344. | X-sect |
| Beeri MS, Goldbourt U. (2011). Late-life dementia predicts mortality beyond established midlife risk factors. American Journal of Geriatric Psychiatry 19(1): 79-87. | Exposure is dementia in late life, outcome is mortality |
| Behre CJ, Bergstrom G, Schmidt CB. (2011). Increasing leisure time physical activity is associated with less prevalence of the metabolic syndrome in healthy middle-aged men. Angiology 62(6): 509-512. | X-sect |
| Berentzen TL, Jakobsen MU, Halkjaer J, Tjonneland A, Sorensen TI, Overvad K. (2011). Changes in waist circumference and the incidence of diabetes in middle-aged men and women. PLoS ONE [Electronic Resource] 6(8): e23104. | Exposure is waist circumference (same paper) |
| Berentzen TL, Jakobsen MU, Stegger JG, Halkjaer J, Tjonneland A, Sorensen TI, et al. (2011). Changes in waist circumference and the incidence of acute myocardial infarction in middle-aged men and women. PLoS ONE [Electronic Resource] 6(10): e26849.. | Exposure is waist circumference |
| Bertrais S, Beyeme-Ondoua JP, Czernichow S, Galan P, Hercberg S, Oppert JM. (2005). Sedentary behaviors, physical activity, and metabolic syndrome in middle-aged French subjects. Obesity Research 13(5): 936-944. | X-sect |
| Beydoun, MA, Wang YF. (2010).Pathways linking socioeconomic status to obesity through depression and lifestyle factors among young US adults. Journal of Affective Disorders 123(1-3): 52-63. | Not midlife - young adults |
| Biggs ML, Mukamal KJ, Luchsinger JA, Ix JH, Carnethon MR, Newman AB, et al. (2010). Association between adiposity in midlife and older age and risk of diabetes in older adults. JAMA 303(24): 2504-2512. | Exposure is obesity, outcome diabetes |
| Bjorkelund C, Bondyr-Carlsson D, Lapidus L, Lissner L, Mansson J, Skoog I, et al. (2005). Sleep disturbances in midlife unrelated to 32-year diabetes incidence: the prospective population study of women in Gothenburg. Diabetes Care 28(11): 2739-2744. | Sleep duration and problems as exposure |
| Bjornholt JV, Erikssen G, Liestol K, Jervell J, Erikssen J, Thaulow E. (2001). Prediction of Type 2 diabetes in healthy middle-aged men with special emphasis on glucose homeostasis. Results from 22.5 years' follow-up. Diabetic Medicine 18(4): 261-267. | Not HB |
| Bodegard J, Erikssen G, Bjornholt JV, Gjesdal K, Thelle D, Erikssen J. (2004). Symptom-limited exercise testing, ST depressions and long-term coronary heart disease mortality in apparently healthy middle-aged men. European Journal of Cardiovascular Prevention & Rehabilitation 11(4): 320-327. | Exposure not HB |
| Boone-Heinonen J, Gordon-Larsen P, Kiefe CI, Shikany JM, Lewis CE, Popkin BM. (2011). Fast food restaurants and food stores: longitudinal associations with diet in young to middle-aged adults: the CARDIA study. Archives of Internal Medicine 171(13): 1162-1170. | Not diagnosed health outcomes (diet quality) |
| Bowling A, Dieppe P. (2005). What is successful ageing and who should define it? BMJ 331 24-31. | SR of definitions of healthy ageing |
| Breeze E, Clarke R, Shipley MJ, Marmot MG, Fletcher AE. (2006). Cause-specific mortality in old age in relation to body mass index in middle age and in old age: follow-up of the Whitehall cohort of male civil servants. International Journal of Epidemiology 35(1): 169-178. | Exposure is BMI, outcome is mortality |
| Briggs JE, McKeown PP, Crawford VL, Woodside JV, Stout RW, Evans A, et al. (2006). Angiographically confirmed coronary heart disease and periodontal disease in middle-aged males. Journal of Periodontology 77(1): 95-102. |  |
| Brown WJ, Mishra G, et al. (2000). Leisure time physical activity in Australian women: Relationship with well being and symptoms. Research Quarterly for Exercise and Sport 71(3): 206-216. | X-sect |
| Buckley J, Tucker G, Hugo G, Wittert G, Adams RJ, Wilson DH. (2013). The Australian baby boomer population--factors influencing changes to health-related quality of life over time. Journal of Aging & Health 25(1): 29-55. | Sedentary behaviour exposure but follow up is 4 years |
| Burazeri G, Kark JD. (2010). Prevalence and determinants of binge drinking in middle age in a transitional post-communist country: a population-based study in Tirana, Albania. Alcohol & Alcoholism 45(2): 180-187. | X-sect |
| Busetto L, Romanato G, Zambon S, Calo E, Zanoni S, Corti MC, et al. (2009). The effects of weight changes after middle age on the rate of disability in an elderly population sample. Journal of the American Geriatrics Society 57(6): 1015-1021. | Exposure is weight loss/weight gain |
| Calton BA, Lacey JV Jr, Schatzkin A, Schairer C, Colbert LH, Albanes D, Leitzmann MF. (2006). Physical activity and the risk of colon cancer among women: A prospective cohort study (United States). International Journal of Cancer 15;119(2):385-91. | Mean age at baseline 61 y and >92% postmenopausal. But not specifically midlife, includes >65 years and not separated in analysis. |
| Carlsson S, Hammar N, Efendic S, Persson PG, Ostenson CG, Grill V. (2000). Alcohol consumption, Type 2 diabetes mellitus and impaired glucose tolerance in middle-aged Swedish men. Diabetic Medicine 17(11): 776-781. | X-sect |
| Carroll D, Phillips AC, Ring C, Der G, Hunt K. (2005). Life events and hemodynamic stress reactivity in the middle-aged and elderly. Psychophysiology 42(3): 269-276. | X-sect |
| Carroll S, Cooke CB, Butterly RJ, Gately P. (2001). Associations of leisure-time physical activity and obesity with atherogenic lipoprotein-lipid markers among non-smoking middle-aged men. Scandinavian Journal of Medicine & Science in Sports 11(1): 38-46. | X-sect |
| Carroll S, Cooke CB, Butterly RJ. (2000). Leisure time physical activity, cardiorespiratory fitness, and plasma fibrinogen concentrations in nonsmoking middle-aged men. Medicine & Science in Sports & Exercise 32(3): 620-626. | X-sect |
| Carroll S, Cooke CB, Butterly RJ. (2000). Metabolic clustering, physical activity and fitness in nonsmoking, middle-aged men. Medicine & Science in Sports & Exercise 32(12): 2079-2086. | X-sect |
| Caspers K, Arndt S, Yucuis R, McKirgan L, Spinks R. (2010). Effects of alcohol- and cigarette-use disorders on global and specific measures of cognition in middle-age adults. Journal of Studies on Alcohol & Drugs 71(2): 192-200. | Outcomes midlife |
| Caspers KM, Yucuis R, McKirgan LM, Spinks R, Arndt S. (2009). Lifetime substance misuse and 5-year incidence rates of emergent health problems among middle-aged adults. Journal of Addictive Diseases 28(4): 320-331. | Outcomes midlife - X-sect? |
| Cassidy A, Mukamal KJ, Liu L, Franz M, Eliassen AH, Rimm EB. (2013). High anthocyanin intake is associated with a reduced risk of myocardial infarction in young and middle-aged women. Circulation 127(2): 188-196. | Mean age at baseline is 25-42 (mean 36) |
| Castelo-Branco C, Blumel JE, Roncagliolo ME, Haya J, Bolf D, Binfa L, et al. (2003). Age, menopause and hormone replacement therapy influences on cardiovascular risk factors in a cohort of middle-aged Chilean women. Maturitas 45(3): 205-212. | Exposures measured include smoking and sedentary behaviour but no analysis of associations with DDF, just follow up of same measures 5 years later. |
| Ceria-Ulep CD, Grove J, Chen R, Masaki KH, Rodriguez BL, Donlon TA, et al. (2010). Physical aspects of healthy aging: assessments of three measures of balance for studies in middle-aged and older adults. Current Gerontology & Geriatrics Research 2010: 849761. | X-sect |
| Chen M, He M, Min X, Pan A, Zhang X, Yao P, et al. (2013). Different physical activity subtypes and risk of metabolic syndrome in middle-aged and older Chinese people. PLoS ONE [Electronic Resource] 8(1): e53258. | X-sect |
| Cheung YB, Machin D, Karlberg J, Khoo KS. (2004). A longitudinal study of pediatric body mass index values predicted health in middle age. Journal of Clinical Epidemiology 57(12): 1316-1322. | Exposure in childhood, outcomes midlife |
| Chi D, Nakano M, Yamamoto K. (2003). Correlates of serum high-density lipoprotein cholesterol: a community-based study of middle-aged and older men and women in Japan. Asia-Pacific Journal of Public Health 15(1): 17-22. | X-sectional |
| Chiang CJ, Yip PK, Wu SC, Lu CS, Liou CW, Liu HC, et al. (2007). Midlife risk factors for subtypes of dementia: a nested case-control study in Taiwan. American Journal of Geriatric Psychiatry 15(9): 762-771. |  |
| Choi JK, Kim MY, Kim JK, Park JK, Oh SS, Koh SB, et al. (2011). Association between short sleep duration and high incidence of metabolic syndrome in midlife women. Tohoku Journal of Experimental Medicine 225(3): 187-193. | Midlife outcomes |
| Cholesterol Treatment Trialists’ (CTT) Collaboration. (2010). Effi cacy and safety of more intensive lowering of LDL cholesterol: a meta-analysis of data from 170 000 participants in 26 randomised trials. Lancet 2010; 376: 1670–81. | Statin therapy, drugs |
| Chou KL, Liang K, Mackenzie CS. (2011). Binge drinking and Axis I psychiatric disorders in community-dwelling middle-aged and older adults: results from the National Epidemiologic Survey on Alcohol and Related Conditions (NESARC). Journal of Clinical Psychiatry 72(5): 640-647. | Binge drinking and psychiatric disorders 3 yr follow up |
| Cosgrove MC, Franco OH, Granger SP, Murray PG, Mayes AE. (2007). Dietary nutrient intakes and skin-aging appearance among middle-aged American women.[Erratum appears in Am J Clin Nutr. 2008 Aug;88(2):480]. American Journal of Clinical Nutrition 86(4): 1225-1231. | X-sect |
| Cournot M, Marquie JC, Ansiau D, Martinaud C, Fonds H, Ferrieres J, et al. (2006). Relation between body mass index and cognitive function in healthy middle-aged men and women. Neurology 67(7): 1208-1214. | Relation between BMI and cognition |
| Covinsky KE, Yaffe K, Lindquist K, Cherkasova E, Yelin E, Blazer DG (2010). Depressive symptoms in middle age and the development of later-life functional limitations: the long-term effect of depressive symptoms. Journal of the American Geriatrics Society 58(3): 551-556. | Exposure is depression |
| Crane PK, Gibbons LE, Arani K, Nguyen V, Rhoads K, McCurry SM, et al. (2009). Midlife use of written Japanese and protection from late life dementia. Epidemiology 20(5): 766-774. | Midlife use of Japanese but could have been learnt in childhood, not a midlife behaviour |
| Crichton GE, Murphy KJ, Bryan J. (2010). Dairy intake and cognitive health in middle-aged South Australians. Asia Pacific Journal of Clinical Nutrition 19(2): 161-171. | X-sectional |
| Czernichow S, Bruckert E, Bertrais S, Galan P, Hercberg S, Oppert JM. (2007). Hypertriglyceridemic waist and 7.5-year prospective risk of cardiovascular disease in asymptomatic middle-aged men. International Journal of Obesity 31(5): 791-796. | Weight at midlife, 2 year follow up |
| Czernichow S, Mennen L, Bertrais S, Preziosi P, Hercberg S, Oppert JM. (2002). Relationships between changes in weight and changes in cardiovascular risk factors in middle-aged French subjects: effect of dieting. International Journal of Obesity & Related Metabolic Disorders: Journal of the International Association for the Study of Obesity 26(8): 1138-1143. | Exposure is WC/TG |
| Dahl A, Hassing LB, Fransson E, Berg S, Gatz M, Reynolds CA, et al. (2010). Being overweight in midlife is associated with lower cognitive ability and steeper cognitive decline in late life. Journals of Gerontology Series A-Biological Sciences & Medical Sciences 65(1): 57-62. | Exposure is OW, not health behaviour |
| Dahl AK, Hassing LB, Fransson EI, Gatz M, Reynolds CA, Pedersen NL (2013). Body mass index across midlife and cognitive change in late life. International Journal of Obesity 37(2): 296-302. | Exposure is BMI |
| Dai Q, Borenstein AR, et al. (2006).Fruit and Vegetable Juices and Alzheimer’s Disease: The Kame Project. The American Journal of Medicine 119, 751-759. | >65y at baseline |
| Danesh J, Saracci R, Berglund G, Feskens E, Overvad K, Panico S, et al. (2007). EPIC-Heart: the cardiovascular component of a prospective study of nutritional, lifestyle and biological factors in 520,000 middle-aged participants from 10 European countries. European Journal of Epidemiology 22(2): 129-141. | No results, protocol |
| Danforth KN, Townsend MK, Lifford K, Curhan GC, Resnick NM, Grodstein F. (2006). Risk factors for urinary incontinence among middle-aged women. American Journal of Obstetrics & Gynecology 194(2): 339-345. | X-sect |
| Daroszewski EB. (2004). Dietary fat consumption, readiness to change, and ethnocultural association in midlife African American women. Journal of Community Health Nursing 21(2): 63-75. | Not cohort study |
| Daviglus ML, Liu K, Pirzada A, Yan LL, Garside DB, Feinglass J, et al. (2003). Favorable cardiovascular risk profile in middle age and health-related quality of life in older age. Archives of Internal Medicine 163(20): 2460-2468. | Smoking but cannot separate smoking from other risk factors |
| Daviglus ML, Liu K, Pirzada A, Yan LL, Garside DB, Wang R, et al. (2005). Relationship of fruit and vegetable consumption in middle-aged men to medicare expenditures in older age: the Chicago Western Electric Study. Journal of the American Dietetic Association 105(11): 1735-1744. | Diet, outcome is health costs in older age |
| Daviglus ML, Liu K, Yan LL, Pirzada A, Garside DB, Schiffer L, et al. (2003). Body mass index in middle age and health-related quality of life in older age: the Chicago heart association detection project in industry study. Archives of Internal Medicine 163(20): 2448-2455. | Midlife exposure is BMI |
| Daviglus ML, Liu K, Yan LL, Pirzada A, Manheim L, Manning W, et al. (2004). Relation of body mass index in young adulthood and middle age to Medicare expenditures in older age. JAMA 292(22): 2743-2749. | Midlife exposure is BMI |
| Davis NC, Friedrich D. (2010). Age stereotypes in middle-aged through old-old adults. International Journal of Aging & Human Development 70(3): 199-212. |  |
| de Lauzon-Guillain B, Balkau B, Charles MA, Romieu I, Boutron-Ruault MC, Clavel-Chapelon F. (2010). Birth weight, body silhouette over the life course, and incident diabetes in 91,453 middle-aged women from the French Etude Epidemiologique de Femmes de la Mutuelle Generale de l'Education Nationale (E3N) Cohort. Diabetes Care 33(2): 298-303. | Exposure is body silhouette |
| Deary IJ, Allerhand M, Der G. (2009). Smarter in middle age, faster in old age: a cross-lagged panel analysis of reaction time and cognitive ability over 13 years in the West of Scotland Twenty-07 Study. Psychology & Aging 24(1): 40-47. | Exposure is processing speed |
| Defina LF, Willis BL, Radford NB, Gao A, Leonard D, Haskell WL, et al. (2013). The association between midlife cardiorespiratory fitness levels and later-life dementia: a cohort study.[Summary for patients in Ann Intern Med. 2013 Feb 5;158(3):I-36; PMID: 23381057]. Annals of Internal Medicine 158(3): 162-168. | Exposure is physical fitness |
| Delavar M, Lye M, Hassan S, Khor G, Hanachi P. (2011). Physical activity, nutrition, and dyslipidemia in middle-aged women. Iranian Journal of Public Health 40(4): 89-98. | X-sect |
| Demakakos P, Pierce MB, Hardy R. (2010). Depressive symptoms and risk of type 2 diabetes in a national sample of middle-aged and older adults: the English longitudinal study of aging. Diabetes Care 33(4): 792-797. | Exposure is depression |
| den Ouden ME, Schuurmans MJ, Brand JS, Arts IE, Mueller-Schotte S, van der Schouw YT. (2013). Physical functioning is related to both an impaired physical ability and ADL disability: a ten year follow-up study in middle-aged and older persons. Maturitas 74(1): 89-94. | Physical function measured at baseline |
| Denollet J, Maas K, Knottnerus A, Keyzer JJ, Pop VJ. (2009). Anxiety predicted premature all-cause and cardiovascular death in a 10-year follow-up of middle-aged women. Journal of Clinical Epidemiology 62(4): 452-456. | Exposure is anxiety |
| Deshpande N, Metter EJ, Guralnik J, Bandinelli S, Ferrucci L. (2013). Predicting 3-year incident mobility disability in middle-aged and older adults using physical performance tests. Archives of Physical Medicine & Rehabilitation 94(5): 994-997. | Exposure is physical performance, only 3 year follow up. |
| Dhingra R, Sullivan L, Jacques PF, Wang TJ, Fox CS, Meigs JB, et al. (2007). Soft drink consumption and risk of developing cardiometabolic risk factors and the metabolic syndrome in middle-aged adults in the community.[Erratum appears in Circulation. 2007 Dec 4;116(23):e557]. Circulation 116(5): 480-488. | Exposure is soft drink consumption, 4 year follow up, outcome is metabolic syndrome |
| Driscoll I, Beydoun MA, An Y, Davatzikos C, Ferrucci L, Zonderman AB, et al. (2012). Midlife obesity and trajectories of brain volume changes in older adults. Human Brain Mapping 33(9): 2204-2210. | Exposure is obesity |
| Du ., van der A DL, et al. (2009). Dietary glycaemic index, glycaemic load and subsequent changes of weight and waist circumference in European men and women. International Journal of Obesity 33(11): 1280-1288. | Not specifically midlife, age range is 20-78 years |
| Ecob R, Sutton G, Rudnicka A, Smith P, Power C, Strachan D, et al. (2008). Is the relation of social class to change in hearing threshold levels from childhood to middle age explained by noise, smoking, and drinking behaviour? International Journal of Audiology 47(3): 100-108. | Exposure in childhood, outcomes in middle age. |
| Ekelund U, Besson H, et al. (2011). Physical activity and gain in abdominal adiposity and body weight: prospective cohort study in 288,498 men and women. American Journal of Clinical Nutrition. 93:4;826-835. | Not specifically midlife, broader age range. Results stratified by > or <50 y so may not be that useful for assessing midlife population specifically. |
| Elbaz A, Sabia S, Brunner E, Shipley M, Marmot M, Kivimaki M, et al. (2013). Association of walking speed in late midlife with mortality: results from the Whitehall II cohort study. Age 35(3): 943-952. | Exposure is walking speed |
| Elovainio M, Kivimaki M, Ferrie JE, Gimeno D, De Vogli R, Virtanen M, et al. (2009). Physical and cognitive function in midlife: reciprocal effects? A 5-year follow-up of the Whitehall II study. Journal of Epidemiology & Community Health 63(6): 468-473. | Exposure is physical function in midlife |
| Emberson JR, Whincup PH, Morris RW, Walker M. (2004). Social class differences in coronary heart disease in middle-aged British men: implications for prevention. International Journal of Epidemiology 33(2): 289-296. | Exposure is social class |
| Eriksson M, Udden J, Hemmingsson E, Agewall S. (2010). Impact of physical activity and body composition on heart function and morphology in middle-aged, abdominally obese women. Clinical Physiology and Functional Imaging Sep;30(5):354-9. | Intervention study 6 month follow up |
| Etgen T, Sander D, et al. (2010).Physical Activity and Incident Cognitive Impairment in Elderly Persons. Archives of Internal Medicine 170(2):186-193. | Relevant PA - cognition but follow-up 2 years, mean age at baseline >65 y. |
| Falba T. (2005). Health events and the smoking cessation of middle aged Americans. Journal of Behavioral Medicine 28(1): 21-33. | Exposure is serious health events, outcome is smoking. |
| Farzadfar F, MM Finucane, et al. (2011). National, regional, and global trends in serum total cholesterol since 1980: systematic analysis of health examination surveys and epidemiological studies with 321 country-years and 3.0 million participants. Lancet 377:578-86. | Cholesterol trends data not HB |
| Feinglass J, Lin S, Thompson J, Sudano J, Dunlop D, Song J, et al. (2007). Baseline health, socioeconomic status, and 10-year mortality among older middle-aged Americans: findings from the Health and Retirement Study, 1992 2002. Journals of Gerontology Series B-Psychological Sciences & Social Sciences 62(4): S209-217. |  |
| Fernandez-Alonso AM, Trabalon-Pastor M, Vara C, Chedraui P, Perez-Lopez FR, MenopAuse RARG. (2012). Life satisfaction, loneliness and related factors during female midlife. Maturitas 72(1): 88-92. | X-sect |
| Field AE, Wing RR, Manson JE, Spiegelman DL, Willett WC. (2001). Relationship of a large weight loss to long-term weight change among young and middle-aged US women. International Journal of Obesity & Related Metabolic Disorders: Journal of the International Association for the Study of Obesity 25(8): 1113-1121. | Weight as exposure |
| Fitzpatrick AL, Kuller LH, Lopez OL, Diehr P, O'Meara ES, Longstreth WT, Jr., et al. (2009). Midlife and late-life obesity and the risk of dementia: cardiovascular health study. Archives of Neurology 66(3): 336-342. | Exposure is obesity, outcome is dementia |
| Flugsrud GB, Nordsletten L, Espehaug B, Havelin LI, Meyer HE. (2007). The effect of middle-age body weight and physical activity on the risk of early revision hip arthroplasty: a cohort study of 1,535 individuals. Acta Orthopaedica 78(1): 99-107. | Not primary incidence of hip arthroplasty but subsequent loosening of hip replacements |
| Franco, M, Ordunez P, Caballero B, Granados JAT, Lazo M, Bernal JL, Guallar E, Cooper RS. (2007). Impact of energy intake, physical activity, and population-wide weight loss on cardiovascular disease and diabetes mortality in Cuba, 1980-2005. American Journal of Epidemiology 166:12;1374-1380. | Not specifically midlife, age 15-74 |
| Fratiglioni L , Paillard-Borg S, Winblad B. (2004). An active and socially integrated lifestyle in late life might protect against dementia. Lancet Neurology Jun;3(6):343-53. | Review, not SR, social networks and dementia. Only 1 study in midlife (Hulsch 1999), rest all mean age >65y. |
| Freedman VA, Martin LG, Schoeni RF, Cornman JC. (2008). Declines in late-life disability: the role of early- and mid-life factors. Social Science & Medicine 66(7): 1588-1602. | No HB at midlife |
| Gallo LC, Jimenez JA, Shivpuri S, Espinosa de los Monteros K, Mills PJ. (2011). Domains of chronic stress, lifestyle factors, and allostatic load in middle-aged Mexican-American women. Annals of Behavioral Medicine 41(1): 21-31. | Exposure is stress, outcome is allostatic load. Age 40-65. X-sect |
| Gallo LC, Troxel WM, Matthews KA, Kuller LH. (2003). Marital status and quality in middle-aged women: Associations with levels and trajectories of cardiovascular risk factors. Health Psychology 22(5): 453-463. | Exposure is marital status/quality |
| Gautam P, Cherbuin N, Sachdev PS, Wen W, Anstey KJ. (2011). Relationships between cognitive function and frontal grey matter volumes and thickness in middle aged and early old-aged adults: the PATH Through Life Study. Neuroimage 55(3): 845-855. | X-sect. Exposure is grey matter volume. Outcome is cog fn. |
| George ES, Rosenkranz RR, Kolt GS. (2013). Chronic disease and sitting time in middle-aged Australian males: findings from the 45 and Up Study. International Journal of Behavioral Nutrition & Physical Activity 10: 20. | X-sect analysis. |
| Ginty AT, Carroll D, Roseboom TJ, Phillips AC, de Rooij SR. (2013). Depression and anxiety are associated with a diagnosis of hypertension 5 years later in a cohort of late middle-aged men and women. Journal of Human Hypertension 27(3): 187-190. | Exposure is depression and anxiety. |
| Goon JA, Aini AH, Musalmah M, Anum MY, Nazaimoon WM, Ngah WZ. (2009). Effect of Tai Chi exercise on DNA damage, antioxidant enzymes, and oxidative stress in middle-age adults. Journal of Physical Activity & Health 6(1): 43-54. | Exposure is Tai Chi, sedentary behaviour. Outcome is DNA damage but not specific health conditions. |
| Gray L, Hart CL, Smith GD, Batty GD. (2010). What is the predictive value of established risk factors for total and cardiovascular disease mortality when measured before middle age? Pooled analyses of two prospective cohort studies from Scotland. European Journal of Cardiovascular Prevention & Rehabilitation 17(1): 106-112. | Age 15-35 at baseline |
| Gray L, Lee IM, Sesso HD, Batty GD. (2011). Blood pressure in early adulthood, hypertension in middle age, and future cardiovascular disease mortality: HAHS (Harvard Alumni Health Study). Journal of the American College of Cardiology 58(23): 2396-2403. | Links between blood pressure and later CVD, mortality - pre-conditions |
| Guan JW, Huang CH, et al. (2011). No Association Between Hypertension and Risk for Alzheimer’s Disease: A Meta-Analysis of Longitudinal Studies. Journal of Alzheimer’s Disease 27 (2011) 799–807. | Exposure is hypertension |
| Guo X, Pantoni L, Simoni M, Gustafson D, Bengtsson C, Palmertz B, et al. (2006). Midlife respiratory function related to white matter lesions and lacunar infarcts in late life: the Prospective Population Study of Women in Gothenburg, Sweden. Stroke 37(7): 1658-1662. | Exposure is respiratory function, age 70-92 at baseline. |
| Gureje O, Ogunniyi A, et al. (2011). Incidence of and Risk Factors for Dementia in the Ibadan Study of Aging” Journal of American Geriatric Society 59(5):869-74. | Age >65 at baseline |
| Gustafsson PE, Janlert U, Theorell T, Westerlund H, Hammarstrom A. (2012). Do peer relations in adolescence influence health in adulthood? Peer problems in the school setting and the metabolic syndrome in middle-age. PLoS ONE [Electronic Resource] 7(6): e39385. | Exposure is peer problems in adolescence (16y). Outcome is metabolic syndrome in midlife. |
| Guthrie JR, Dennerstein L, Taffe JR, Lehert P, Burger HG. (2004). The menopausal transition: a 9-year prospective population-based study. The Melbourne Women's Midlife Health Project. Climacteric 7(4): 375-389. |  |
| Hall MH, MF Muldoon, et al. (2008). Self-Reported Sleep Duration is Associated with the Metabolic Syndrome in Midlife Adults. Sleep 31(5) 635-643. | X-sect |
| Hall MH, Michele L, et al. (2012). Sleep Is Associated with the Metabolic Syndrome in a Multi-Ethnic Cohort of Midlife Women: The SWAN Sleep Study. Sleep 35(6):783-90. | X-sect |
| Ham E, Choi H, et al. (2009). Risk Factors for Female Urinary Incontinence among Middle-Aged Korean Women. Journal of Women’s Health 18(11):1801-6. | X-sect |
| Hamer M, Chida Y. (2009). Physical activity and risk of neurodegenerative disease: a systematic review of prospective evidence. Psychological Medicine 39, 03-11. | Exposure is fitness, 3 yr follow-up |
| Hamer M, Steptoe A. (2009). Prospective study of physical fitness, adiposity, and inflammatory markers in healthy middle-aged men and women. American Journal of Clinical Nutrition 89(1): 85-89. | SR, most studies in people age >65. 3 studies <65 - check primary for inclusion - Chen 2005, Rovio 2005, Yamada 2003. |
| Hart CL, Hole DJ, Lawlor DA, Davey Smith G. (2007). How many cases of Type 2 diabetes mellitus are due to being overweight in middle age? Evidence from the Midspan prospective cohort studies using mention of diabetes mellitus on hospital discharge or death records. Diabetic Medicine 24(1):73-80. | Exposure is BMI |
| Hartman-Stein PE, Potkanowicz ES. (2003). Behavioral determinants of healthy aging: good news for the baby boomer generation. Online Journal of Issues in Nursing 8(2): 6. | Review but not SR |
| Haseli-Mashhadi N, Pan A, Ye X, et al. (2009). Self-Rated Health in middle-aged and elderly Chinese: distribution, determinants and associations with cardio-metabolic risk factors. BMC Public Health 9:368. | X-sect, exposure is self-rated health |
| Hassing LB, Dahl AK, Pedersen NL, Johansson B. (2010). Overweight in Midlife Is Related to Lower Cognitive Function 30 Years Later: A Prospective Study with Longitudinal Assessments. Dement and Geriatric Cognitive Disorders 29:543–552. | Exposure is overweight in midlife |
| Hassing LB, Dahl AK, Thorvaldsson D, et al. (2009). Overweight in Midlife and Risk of Dementia: A 40-Year Follow-up Study. International Journal of Obesity (8):893-8. | Exposure is overweight in midlife |
| Hatch SL, Feinstein L, Link BG, Wadsworth MEJ, Richards M. (2007). The Continuing Benefits of Education: Adult Education and Midlife Cognitive Ability in the British 1946 Birth Cohort. Journal of Gerontology: Social Sciences Vol. 62B, No. 6, S404–S414. | Outcomes at midlife |
| Haveman-Nies A, De Groot LCPGM, van Stavern WA. (2003). Dietary quality, lifestyle factors and healthy ageing in Europe: the SENECA study. Age and Ageing 32: 427–434. | Age 70-75 at baseline. |
| Hawkley LC, Thisted RA, Masi CM, Cacioppo JT. (2010). Loneliness Predicts Increased Blood Pressure: Five-Year Cross-Lagged Analyses in Middle-Aged and Older Adults. Psychology and Aging 25(1):132-41. | Exposure is loneliness (poss relevant?) but follow up is 4 yrs. |
| Heir T, T. Erikssen J, Sandvik L. (2011). Overweight as predictor of long-term mortality among healthy, middle-aged men: A prospective cohort study. Preventive Medicine 52 223–226. | Exposure is overweight |
| Henriksson K, Lindblad U, Gullberg B, et al. (2002). Development of hypertension over 6 years in a birth cohort of young middle-aged men: the Cardiovascular Risk Factor Study in southern Sweden (CRISS). Journal of Internal Medicine 252: 21–26. | Baseline ages 37,40,43, follow up 6 years but only to 49 years mas (<55 y) |
| Henriksson KM, Lindblad U, Agren B, Nilsson-Ehle P, Rastam L. (2001). Associations between body height, body composition and cholesterol levels in middle-aged men. The coronary risk factor study in southern Sweden (CRISS). European Journal of Epidemiology 17: 521–526, 2001. | Baseline ages 37,40,43, follow up 6 years but only to 49 years mas (<55 y) |
| Henriksson KM, Lindblad U, Gullberg B, Agren B, Nilsson-Ehle P, Rastam L. (2003). Body composition, ethnicity and alcohol consumption as determinants for the development of blood pressure in a birth cohort of young middle-aged men. European Journal of Epidemiology 18: 955–963, 2003. | Baseline ages 37,40,43, follow up 6 years but only to 49 years mas (<55 y) |
| Heraclides A, Chandola T, Witte DR, Brunner EJ. (2009). Psychosocial stress at work doubles the risk of type 2 diabetes in middle-aged women: evidence from the Whitehall II study. Diabetes Care 32(12):2230-5. | Psychosocial stress as risk factor |
| Hernelahti M, Kujala UM, Kaprio J, Sarna S. (2002). Long-term vigorous training in young adulthood and later physical activity as predictors of hypertension in middle-aged and older men. International Journal of Sports Medicine 23(3):178-82. | Population is elite athletes, includes young adulthood, not midlife specifically |
| Hirokawa W, Nakamura K, Sakurai M, et al. (2010). Mild metabolic abnormalities, abdominal obesity and the risk of cardiovascular diseases in middle-aged Japanese men. Journal of Atherosclerosis and Thrombosis 17(9):934-43. | Exposure is BP, lipids, glucose, obesity. |
| Hjerkinn EM, Sandvik L, Hjermann I, Arnesen H. (2004). Effect of diet intervention on long-term mortality in healthy middle-aged men with combined hyperlipidaemia. Journal of Internal Medicine 255(1):68-73. | Intervention study - review 3 |
| Ho SH, Li CS, Liu CC. (2009). The influence of chronic disease, physical function, and lifestyle on health transition among the middle-aged and older persons in Taiwan. Journal of Nursing Research 17(2):136-43. | Relevant lifestyle behaviours but 4 yr follow up (Taiwan) |
| Hoffman BM, Blumenthal JA, Babyak MA, Smith PJ, Rogers SD, Doraiswamy PM, Sherwood A. (2008). Exercise Fails to Improve Neurocognition in Depressed Middle- Aged and Older Adults. Medicine & Science in Sports & Exercise 40(7): 1344–1352. | Intervention study - review 3? |
| Hoffman GJ, Lee J, Mendez-Luck CA. (2012). Health Behaviors Among Baby Boomer Informal Caregivers. The Gerontologist Vol. 52, No. 2, 219–230. | X-sect analysis |
| Holahan CK. (2003). Stability and change in positive self-appraisal from midlife to later aging. International Journal of Aging and Human Development 56(3):247-67. | Exposure is self-appraisal of having lived up to one's abilities' |
| Holm K, Dan A, Wilbur J, Li S, Walker J. (2002). A longitudinal study of bone density in midlife women. Health Care For Women International 23(6-7):678-91. | 2 year follow up but relevant exposures and outcomes |
| Holmberg AH, Nilsson PM, Nilsson J-Å., Åkesson K. (2008). The Association between Hyperglycemia and Fracture Risk in Middle Age. A Prospective, Population-Based Study of 22,444 Men and 10,902 Women. The Journal of Clinical Endocrinology & Metabolism 93(3):815-22. | Exposure is fasting glucose |
| Holtermann A, Mortensen OS, Burr H, Søgaard K, Gyntelberg F, Suadicani P. (2010). Long work hours and physical fitness: 30-year risk of ischaemic heart disease and all-cause mortality among middle-aged Caucasian men. Heart 96(20):1638-44. | Exposure is long worl hours/fitness |
| Horsten M, Mittleman MA, Wamala SP, Schenck-Gustafsson K, Orth-GomeK. (2000). Depressive symptoms and lack of social integration in relation to prognosis of CHD in middle-aged women. The Stockholm Female Coronary Risk Study. European Heart Journal 21(13):1072-80 | In women with existing CHD |
| Houston DK, Cai J, Stevens J. (2009). Overweight and Obesity in Young and Middle Age and Early Retirement: The ARIC Study. Obesity 17, 143–149. | Exposure is OW/obesity, outcome is early retirement |
| Hu Y, Block G, Sternfeld B, Sowers M. (2009). Dietary glycemic load, glycemic index, and associated factors in a multiethnic cohort of midlife women. The Journal of the American College of Nutrition 28(6):636-47. | X-sect analysis |
| Hultsch DF, Hertzog C, Small BJ, Dixon RA. (1999). Use it or lose it: engaged lifestyle as a buffer of cognitive decline in aging? Psychology and Aging 14(2), 245. | Social networks and dementia - only midlife paper identified from Fratiglioni review but 1999 so pre-2000. |
| Huuskonen J, Väisänen SB, Kröger H, Jurvelin C, Bouchard C, Alhava E, Rauramaa R. (2000). Determinants of bone mineral density in middle aged men: a population-based study. Osteoporos International 11(8):702-8. | Exposure is BMI |
| Huuskonen J, Väisänen SB, Kröger H, Jurvelin JS, Alhava E, Rauramaa R. (2001). Regular physical exercise and bone mineral density: a four-year controlled randomized trial in middle-aged men. The DNASCO study. Osteoporos International 12(5):349-55. | Intervention study - review 3? |
| Hwang GS, Choi JW, Choi SH, Lee SG, Kim KH, Cho YM, Yoon C. (2012). Effects of a tailored health promotion program to reduce cardiovascular disease risk factors among middle-aged and advanced-age bus drivers. Asia Pac Journal of Public Health 24(1):117-27. | Intervention - rev 3? |
| Hwang LC, Chen SC, Chen CJ. (2011). Increased risk of mortality from overweight and obesity in middle-aged individuals from six communities in Taiwan. Journal of the Formosan Medical Association 110(5):290-8. | Exposure is BMI |
| Imagama S, Ito Z, Wakao N, Seki T, Hirano K, Muramoto A, Sakai Y, Matsuyama Y, Hamajima N, Ishiguro N, Hasegawa Y. (2013). Influence of sagittal balance and physical ability associated with exercise on quality of life in middle-aged and elderly people. Archives of Osteoporos 6:13–20 | X-sect analysis |
| Imano H, Kitamura A, Sato S, Kiyama M, Ohira T, Yamagishi K, Noda H, Tanigawa T, Iso H, Shimamoto T. (2009). Trends for blood pressure and its contribution to stroke incidence in the middle-aged Japanese population: the Circulatory Risk in Communities Study (CIRCS). Stroke 40(5):1571-7. | BP exposure |
| Inoue M, Hanaoka T, Sasazuki S, Sobue T, Tsugane S; JPHC Study Group. (2004). Impact of tobacco smoking on subsequent cancer risk among middle-aged Japanese men and women: data from a large-scale population-based cohort study in Japan--the JPHC study. Preventive Medicine 38(5):516-22. | X-sect analysis |
| Iso H, Imano H, Nakagawa Y, Kiyama M, Kitamura A, Sato S, Naito Y, Shimamoto T, Iida M. (2002). One-year community-based education program for hypercholesterolemia in middle-aged Japanese: a long-term outcome at 8-year follow-up. Atherosclerosis 164(1):195-202. | Intervention study - review 3? |
| Jain P, Jain P, Bhandari S, Siddhu A. (2008). A case-control study of risk factors for coronary heart disease in urban Indian middle-aged males. Indian Heart Journal 60(3):233-40. | X-sectional analysis |
| Jakovljević B, Stojanov V, Lović D, Paunović K, Radosavljević V, Tutić I. (2011). Obesity and fat distribution as predictors of aortoiliac peripheral arterial disease in middle-aged men. European Journal of Internal Medicine 22(1):84-8. | Exposure is obesity |
| Jang SY, Ju EY, Choi S, Seo S, Kim DE, Kim DK, Park SW. (2012). Prehypertension and obesity in middle-aged Korean men and women: the third Korea national health and nutrition examination survey (KNHANES III) study. Journal Of Public Health 34(4):562-9. | Exposure is obesity |
| Jayalath VH, de Souza RJ, et al. (2013). Effect of Dietary Pulses on Blood Pressure: A Systematic Review and Meta-analysis of Controlled Feeding Trials. American Journal of Hypertension Sep 7. [Epub ahead of print]. | Review of intervention studies - consider for rev 3 |
| Jeong JY, Lee SK, Kang YW, Jang SN, Choi YJ, Kim DH. (2011). Relationship between ED and depression among middle-aged and elderly men in Korea: Hallym aging study. International Journal of Impotence Research 23(5):227-34. | Exposure is erectile dysfunction |
| Jin L, Huang Y, Bi Y, Zhao L, Xu M, Xu Y, Chen Y, Gu L, Dai M, Wu Y, Hou J, Li X, Ning G. (2011). Association between alcohol consumption and metabolic syndrome in 19,215 middle-aged and elderly Chinese. Diabetes Research and Clinical Practice 92(3):386-92. | X-sect |
| Johansson E, Leijon O, Falkstedt D, Farah A, Hemmingsson T. (2012). Educational differences in disability pension among Swedish middle-aged men: role of factors in late adolescence and work characteristics in adulthood. Journal of Epidemiology and Community Health 66(10):901-7. | X-sect in midlife, exposure in adolescence |
| Johansson L, Guo X, Hällström T, Norton MC, Waern M, Ostling S, Bengtsson C, Skoog I. (2013). Common psychosocial stressors in middle-aged women related to longstanding distress and increased risk of Alzheimer’s disease: a 38 year longitudinal population study. BMJ Open 3(9):e003142. | Exposure is psychosocial stressors |
| Johansson S, Wilhelmsen L, Welin C, Eriksson H, Welin L, Rosengren A. (2010). Obesity, smoking and secular trends in cardiovascular risk factors in middle-aged women: data from population studies in Göteborg from 1980 to 2003. Journal of Internal Medicine 268(6):594-603. | Prevalence/trends |
| Jood K, Jern C, Wilhelmsen L, Rosengren A. (2004). Body mass index in mid-life is associated with a first stroke in men: a prospective population study over 28 years. Stroke 35(12):2764-9. | Exposure is BMI |
| Joosten H, van Eersel ME, Gansevoort RT, Bilo HJ, Slaets JP, Izaks GJ. (2013). Cardiovascular risk profile and cognitive function in young, middle-aged, and elderly subjects. Stroke 44(6):1543-9. | X-sect |
| Jovanovic GK, Zezelj SP, et al. (2010).Diet quality of middle age and older women from Primorsko-Goranska County evaluated by healthy eating index and association with body mass index. Collegium Antropologicum 34 Suppl 2: 155-160. | X-sect |
| Kaffashian S, Dugravot A, Brunner EJ, Sabia S, Ankri J, Kivimäki M, Singh-Manoux A. (2013). Midlife stroke risk and cognitive decline: A 10-year follow-up of the Whitehall II cohort study. Alzheimers & Dementia 9(5):572-9. | Exposure is stroke risk - includes smoking but cannot be separated from other stroke risk factors |
| Kalmijn S, van Boxtel MP, Ocké M, Verschuren WM, Kromhout D, Launer LJ. (2004). Dietary intake of fatty acids and fish in relation to cognitive performance at middle age. Neurology. 27;62(2):275-80. | X-sect, midlife outcomes |
| Kalmijn S, van Boxtel MP, Verschuren MW, Jolles J, Launer LJ. (2002). Cigarette Smoking and Alcohol Consumption in Relation to Cognitive Performance in Middle Age. American Journal of Epidemiology156(10):936-44. | Outcomes are midlife |
| Kamijo T, Murakami M. (2009). Regular Physical Exercise Improves Physical Motor Functions and Biochemical Markers in Middle-Age and Elderly Women. Journal of Physical Activity and Health 6(1):55-62. | Intervention study review 3? |
| Karp A, Andel R, Parker MG, Wang HX, Winblad B, Fratiglioni L. (2009). Regular Physical Exercise Improves Physical Motor Functions and Biochemical Markers in Middle-Age and Elderly Women. American Journal of Geriatric Psychiatry 17(3):227-36. | Exposure is mentally stimulating work |
| Karp A, Kåreholt I. (2004).Relation of Education and Occupation-based Socioeconomic Status to Incident Alzheimer’s Disease. American Journal of Epidemiology 159:175–183. | Exposure is level of education/SES |
| Karp A, Paillard-Borg S, et al. (2006). Mental, Physical and Social Components in Leisure Activities Equally Contribute to Decrease Dementia Risk. Dementia and Geriatric Cognitive Disorders 21:65–73. | Exposure is in over 75 years age |
| Karpansalo M, Manninen P, Lakka TA, Kauhanen J, Rauramaa R, Salonen JT. (2002). Physical Workload and Risk of Early Retirement: Prospective Population-Based Study Among Middle-Aged Men. Journal of Occupational and Environmental Medicine 44(10):930-9. | Outcome is early retirement |
| Karvonen-Gutierrez CA, Ylitalo KR. (2013). Prevalence and Correlates of Disability in a Late Middle-Aged Population of Women. Journal of Aging and Health 25(4):701-17. | X-sect |
| Kim J, Chu SK, Kim K, Moon JR. (2011). Alcohol use behaviors and risk of metabolic syndrome in South Korean middle-aged men. BMC Public Health 22;11:489. | X-sect |
| Kim JW, Lee DY, et al. (2012). Alcohol and Cognition in the Elderly: A Review. Psychiatry Investigation 9:8-16. | Review, not SR, in elderly |
| Kivimäki M, Lawlor DA, et al. (2007).Socioeconomic Position, Co-Occurrence of Behavior-Related Risk Factors, and Coronary Heart Disease: the Finnish Public Sector Study. American Journal of Public Health 97(5):874-9. | Exposure is SES |
| Kivimaki M, Nyberg ST, et al. (2012). Job strain as a risk factor for coronary heart disease: A collaborative meta-analysis of individual participant data. The Lancet 380(9852): 1491-1497. | Exposure is job strain |
| Kivipelto K, T Ngandu, et al. (2006). Risk score for the prediction of dementia risk in 20 years among middle aged people: a longitudinal, population based study. Lancet Neurology 5(9):735-41. | Review, not SR, not midlife. |
| Kivipelto M, Helkala EL, et al. (2002). Apolipoprotein E _4 Allele, Elevated Midlife Total Cholesterol Level, and High Midlife Systolic Blood Pressure Are Independent Risk Factors for Late-Life Alzheimer Disease. Annals of Internal Medicine 137:149-155. | Exposure is BP, serum cholesterol |
| Kivipelto M, Helkala EL, Laakso MP, Hanninen T, Hallikainen M, Alhainen K, et al. (2001). Midlife vascular risk factors and Alzheimer's disease in later life: longitudinal, population based study. BMJ 322(7300): 1447-1451. | Exposure is BP, serum cholesterol |
| Kivipelto M, Solomon A (2006). Cholesterol as a risk factor for Alzheimer’s disease – epidemiological evidence. Acta Neurologica Scandinavica 114 (Suppl. 185): 50–57 | Exposure is a general dementia risk score (includes obesity) |
| Kloppenborg RP, van den Berg E, et al. (2008). Diabetes and other vascular risk factors for dementia: Which factor matters most? A systematic review. European Journal of Pharmacology 585 97–108. | SR, not midlife specifically, exposures are diabetes, BP, lipids, obesity |
| Knopman D, Boland LL, Mosley T, Howard G, Liao D, Szklo M, McGovern P, Folsom AR; Atherosclerosis Risk in Communities (ARIC) Study Investigators. (2001). Cardiovascular risk factors and cognitive decline in middle-aged adults. Neurology 9;56(1):42-8. | For smoking, mean age at baseline 57, follow up 6 years |
| Kozakova M, Palombo C, Mhamdi L, Konrad T, Nilsson P, Staehr PB, Paterni M, Balkau B; RISC Investigators. (2007). Habitual Physical Activity and Vascular Aging in a Young to Middle-Age Population at Low Cardiovascular Risk. Stroke 38(9):2549-55. | Outcome is carotid wall stiffness |
| Kremen WS, Vinogradov S, Poole JH, Schaefer CA, Deicken RF, Factor-Litvak P, Brown AS. (2010). Cognitive Decline in Schizophrenia from Childhood to Midlife: A 33-Year Longitudinal Birth Cohort Study, Schizophrenia Research 118(1-3):1-5. | Cognitive deficit before and after schizophrenia onset |
| Kuh D, Cooper R, Hardy R, Guralnik J, Richards M; Musculoskeletal Study Team. (2009). Lifetime cognitive performance is associated with midlife physical performance in a prospective national birth cohort study. Psychosomatic Medicine 71(1):38-48. | Exposure is cognitive performance, outcome is phys perf at age 53 |
| Kuh D, Hardy R, Butterworth S, Okell L, Richards M, Wadsworth M, Cooper C, Sayer AA. (2006). Developmental Origins of Midlife Physical Performance: Evidence from a British Birth Cohort. American Journal of Epidemiology 15;164(2):110-21. | Exposure is developmental performance from childhood, outcomes age 53 |
| Kukuljan S, Nowson CA, Sanders K, Daly RM. (2009). Effects of resistance exercise and fortified milk on skeletal muscle mass, muscle size, and functional performance in middle-aged and older men: an 18-mo randomized controlled trial. Journal of Applied Physiology 107(6):1864-73 | Intervention - review 3? |
| Kumari M, Marmot M. (2005). Diabetes and cognitive function in a middle-aged cohort: Findings from the Whitehall II study. Neurology 22;65(10):1597-603. | X-sect. Exposure is diabetes |
| Kuo CW, Chang TH, Chi WL, Chu TC. (2008). Effect of Cigarette Smoking on Bone Mineral Density in Healthy Taiwanese Middle-Aged Men. Journal of Clinical Densitometry 11(4):518-24. | X-sect. Exposure is diabetes |
| Kuper H, Adami HO, Theorell T, Weiderpass E. (2006). Psychosocial Determinants of Coronary Heart Disease in Middle-Aged Women: A Prospective Study in Sweden. American Journal of Epidemiology 15;164(4):349-57. | Exposure is subjective rate of aging |
| Kurishima K, Satoh H, Ishikawa H, Yamashita YT, Kamma H, Ohtsuka M, Sekizawa K. (2001). Lung cancer in middle-aged patients. Oncology Reports 8(4):851-3.1. | Comparison of incidence between younger and older case-control patients. |
| Kurl S, Sivenius J, Mäkikallio TH, Rauramaa R, Laukkanen JA. (2008). Exercise workload, cardiovascular risk factor evaluation and the risk of stroke in middle-aged men. Journal of Internal Medicine 265(2):229-37. | Exposure is physical performance (max exercise workload) |
| Laaksonen DE, Niskanen L, Punnonen K, Nyyssönen K, Tuomainen TP, Valkonen VP, Salonen JT. (2005). The Metabolic Syndrome and Smoking in Relation to Hypogonadism in Middle-Aged Men: A Prospective Cohort Study. Journal of Clinical Endocrinolgy & Metabolism 90(2):712-9. | Exposure is MS- outcome hypogonadism |
| Lachman ME, Agrigoroaei S.Lahti. (2010). Promoting Functional Health in Midlife and Old Age: Long-Term Protective Effects of Control Beliefs, Social Support, and Physical Exercise. PLoS One 11;5(10):e13297. | Age 24-75 at baseline, 32-84 at FU, mean age 47 |
| Lallukka T, Chandola T, Hemingway H, Marmot M, Lahelma E, Rahkonen O. (2009). Job strain and symptoms of angina pectoris among British and Finnish middle-aged employees. Journal of Epidemiology & Community Health 63(12):980-5. | X-sect, exp job strain |
| Lamb SE, Bartlett HP, Ashley A, Bird W. (2002). Can lay-led walking programmes increase physical activity in middle aged adults? A randomised controlled trial. Journal of Epidemiology & Community Health 56(4):246-52. | Intervention - rev 3? |
| Laukkanen JA, Kurl S, Lakka TA, Tuomainen TP, Rauramaa R, Salonen R, Eränen J, Salonen JT. (2001). Exercise-Induced Silent Myocardial Ischemia and Coronary Morbidity and Mortality in Middle-Aged Men. Journal of the American College of Cardiology 38(1):72-9. | Exposure is ischaemia |
| Laukkanen JA, Rauramaa R, Kurl S. (2008). Exercise workload, coronary risk evaluation and the risk of cardiovascular and all-cause death in middle-aged men. European Journal of Cardiovascular Prevention & Rehabilitation 15(3):285-92. | Exposure is physical performance (not PA) |
| Launer LJ, Hughes T, Yu B, Masaki K, Petrovitch H, Ross GW, White LR. (2010). Lowering mid-life levels of systolic blood pressure as a public health strategy to reduce late-life dementia: Perspective from the Honolulu Heart Program/Honolulu Asia Aging Study. Hypertension 55(6):1352-9. | Exposure is BP |
| Launer LJ, Ross GW, Petrovitch H, Masaki K, Foley D, White LR, Havlik RJ. (2000). Midlife blood pressure and dementia: the Honolulu–Asia aging study. Neurobiology of Aging 21(1):49-55. | Exposure is BP |
| Laurin D, Verreault R, et al. (2001). Physical activity and risk of cognitive impairment and dementia in elderly persons. Archives of Neurolgy 58(3):498-504. | >65 y at baseline |
| Lee DM, Rutter MK, O'Neill TW, Boonen S, Vanderschueren D, Bouillon R, Bartfai G, Casanueva FF, Finn JD, Forti G, Giwercman A, Han TS, Huhtaniemi IT, Kula K, Lean ME, Pendleton N, Punab M, Silman AJ, Wu FC; European Male Ageing Study Group. (2009). Vitamin D, parathyroid hormone and the metabolic syndrome in middle-aged and older European men. European Journal of Endocrinology 161(6):947-54. | X-sect |
| Lee JS, Kawakubo K, Kobayashi Y, Mori K, Kasihara H, Tamura M. (2001). Effects of ten year body weight variability on cardiovascular risk factors in Japanese middle-aged men and women. International Journal of Obesity & Related Metabolic Disorders: Journal of the International Association for the Study of Obesity 25(7): 1063-1067. | X-sect |
| Lee PG, Cigolle CT, Ha J, Min L, Murphy SL, Blaum CS, Herman WH. (2013). Physical Function Limitations Among Middle-Aged and Older Adults With Prediabetes. Diabetes Care 36(10):3076-83. | X-sect |
| Lee SA, Cai H, Yang G, Xu WH, Zheng W, Li H, Gao YT, Xiang YB, Shu XO. (2010). Dietary patterns and blood pressure among middle-aged and elderly Chinese men in Shanghai. British Journal of Nutrition 104(2):265-75. | Exposure is adiposity |
| Lee WC, Ory MG. (2013). The Engagement in Physical Activity for Middle-Aged and Older Adults with Multiple Chronic Conditions: Findings from a Community Health Assessment. Journal of Aging Research 2013:152868. | X-sect |
| Lee YH, Lee SH, Jung ES, Kim JS, Shim CY, Ko YG, et al. (2010). Visceral adiposity and the severity of coronary artery disease in middle-aged subjects with normal waist circumference and its relation with lipocalin-2 and MCP-1. Atherosclerosis 213(2): 592-597. | X-sect |
| Lêng CH, Wang JD. (2013). Long term determinants of functional decline of mobility: An 11-year follow-up of 5464 adults of late middle aged and elderly. Archives of Gerontology & Geriatrics 57(2):215-20. | 50-97 at baseline, mean age >65 at baseline |
| Leskinen T, Sipilä S, Kaprio J, Kainulainen H, Alen M, Kujala UM. (2013). Physically active vs. inactive lifestyle, muscle properties, and glucose homeostasis in middle-aged and older twins. Age (Dordr) 35(5):1917-26. | Outcome is physical composition performance rather than illness/frailty related |
| Letenneur L, Larrieu S, Barberger-Gateau P. (2004). Alcohol and tobacco consumption as risk factors of dementia: a review of epidemiological studies. Biomedicine & Pharmacotherapy 58 95–99. | Review but not systematic review |
| Levinger I, Howlett KF, Peake J, Garnham A, Hare DL, Jerums G, Selig S, Goodman C. (2010). Akt, AS160, metabolic risk factors and aerobic fitness in middle-aged women. Exercise Immunology Review 16:98-104. | X-sect, metabolic inflamm markers, fitness |
| Lewis TT, Everson-Rose SA, Karavolos K, Janssen I, Wesley D, Powell LH. (2009). Hostility is associated with Visceral, but not Subcutaneous, Fat in Middle-Aged African-American and White women. Psychosomatic Medicine 71(7):733-40. | Exposure is hostility |
| Lewis TT, Kravitz HM, Janssen I, Powell LH.(2011). Self-reported Experiences of Discrimination and Visceral Fat in Middle-aged African-American and Caucasian Women. American Journal of Epidemiology 1;173(11):1223-31. | Exposure is discrimination |
| Li F, Harmer P, Cardinal BJ, Vongjaturapat N. (2009). Built Environment and Changes in Blood Pressure in Middle Aged and Older Adults. Preventive Medicine 48(3):237-41. | Relevant but 1 year follow up |
| Li Y, Yatsuya H, Iso H, Tamakoshi K, Toyoshima H. (2010). Incidence of metabolic syndrome according to combinations of lifestyle factors among middle-aged Japanese male workers. Preventive Medicine 51(2):118-22. | Relevant but 3 year follow up |
| Lida T, Ikeda H, Shiokawa M, Aoi S, Ishizaki F, Harada T, Ono Y. (2012). Longitudinal study on physical fitness parameters influencing bone mineral density reduction in middle-aged and elderly women: bone mineral density in the lumbar spine, femoral neck, and femur. Hiroshima Journal of Medical Science 61(2):23-8. | 1 year follow-up, mainly physical fitness but reports mean amount of exercise |
| Lida T, Ikeda H, Shiokawa M, Aoi S, Ishizaki F, Harada T, Ono Y. (2012). Longitudinal study on physical fitness parameters influencing bone mineral density reduction in middle-aged and elderly women: bone mineral density in the lumbar spine, femoral neck, and femur. Hiroshima Journal of Medical Science. 2012 Jun;61(2):23-8. | 1 year follow-up, mainly physical fitness but reports mean amount of exercise |
| Lidfeldt J, Nyberg P, Nerbrand C, Ojehagen A, Samsioe G, Scherstén B, Agardh CD. (2002). Biological Factors are More Important than Socio-demographic and Psychosocial Conditions in Relation to Hypertension in Middle-aged Women. The Women’s Health in the Lund Area (WHILA) Study. Blood Pressure 11(5):270-8. | X-sect |
| Lim NK, Park SH, Choi SJ, Lee KS, Park HY. (2012). A Risk Score for Predicting the Incidence of Type 2 Diabetes in a Middle-Aged Korean Cohort – The Korean Genome and Epidemiology Study. Circulation Journal 76(8):1904-10. | Overall risk score rather than individual behaviours |
| Lin YC, Chen JD, Chen PC. (2011). Excessive 5-year weight gain predicts metabolic syndrome development in healthy middle-aged adults. World Journal of Diabetes 2(1): 8-15. | Mean age 32 |
| Lin YC, Hsiao TJ, Chen PC. (2009). Persistent rotating shift-work exposure accelerates development of metabolic syndrome among middle-aged female employees: a five-year follow-up. Chronobiology International 26(4):740-55. | Mean age 32 |
| Lin YC, Hsiao TJ, Chen PC. (2009). Shift work aggravates metabolic syndrome development among early-middle-aged males with elevated ALT. World Journal of Gastroenterology 7;15(45):5654-61 | Mean age 32 |
| Lindsay J, Laurin D, et al. (2002). Risk Factors for Alzheimer’s Disease: A Prospective Analysis from the Canadian Study of Health and Aging. American Journal of Epidemiology 156(5):445-53. | >65years at baseline |
| Lindström I, Pallasaho P, Luukkonen R, Suojalehto H, Karjalainen J, Lauerma A, Karjalainen A (2011). Reduced work ability in middle-aged men with asthma from youth- a 20-year follow-up. Respiratory Medicine 105(6):950-5. | Exposure is childhood asthma |
| Lindström M, Hanson BS, Brunner E, Wirfält E, Elmståhl S, Mattisson I, Ostergren PO. (2000). Socioeconomic differences in fat intake in a middle-aged population: report from the Malmö Diet and Cancer Study. International Journal of Epidemiology 29(3):438-48. | X-sect, SES in fat intake |
| Liu C, Yu Z, Li H, Wang J, Sun L, Qi Q, Lin X. (2010). Associations of alcohol consumption with diabetes mellitus and impaired fasting glycemia among middle-aged and elderly Chinese. BMC Public Health 19;10:713. | X-sect |
| Liu K, Daviglus ML, Loria CM, Colangelo LA, Spring B, Moller AC, Lloyd-Jones DM. (2012). Healthy Lifestyle through Young Adulthood and Presence of Low Cardiovascular Disease Risk Profile in Middle Age: The Coronary Artery Risk Development in (Young) Adults (CARDIA) Study. Circulation 28;125(8):996-1004. | X-sect |
| Liu-Ambrose T, Donaldson MG (2009). Exercise and cognition in older adults: is there a role for resistance training programmes? British Journal of Sports Medicine 43:25–27. | Review of resistance training programmes, >65y at baseline |
| Loef M, Walach H. (2012). Fruit, vegetables and prevention of cognitive decline or dementia: a systematic review of cohort studies. The Journal of Nutrition, Health & Aging 16(7): 626-30. | SR on midlife obesity and dementia |
| Lu Y, Lu J, Wang S, Li C, Liu L, Zheng R, Tian H, Wang X, Yang L, Zhang Y, Pan C. (2012). Cognitive function with glucose tolerance status and obesity in Chinese middle-aged and aged adults. Aging & Mental Health 16(7):911-4. | Exposures in young adulthood, outcomes in middle-age |
| Ma E, Sasazuki S, Iwasaki M, Sawada N, Inoue M; Shoichiro Tsugane; Japan Public Health Center-based Prospective Study Group. (2010). 10-Year risk of colorectal cancer: Development and validation of a prediction model in middle-aged Japanese men. Cancer Epidemiology 34(5):534-41. | Prediction model for colorectal cancer |
| Maatouk I, Wild B, Herzog W, Wesche D, Schellberg D, Schöttker B, Müller H, Rothenbacher D, Stegmaier C, Brenner H. (2012). Longitudinal predictors of health-related quality of life in middle-aged and older adults with hypertension: results of a population-based study. Journal of Hypertension 30(7):1364-72. | Patients with existing hypertension |
| Mahamat A, Richard F, Arveiler D, Bongard V, Yarnell J, Ducimetière P, Ruidavets JB, Haas B, Bingham A, Evans A, Amouyel P, Dallongeville J. (2003). Body mass index, hypertension and 5-year coronary heart disease incidence in middle aged men: the PRIME study. Journal of Hypertension 21(3):519-24. | Exposure is hypertension and BMI |
| Malhotra A. (2013). Saturated fat is not the major issue. BMJ 201w3 347:f6340. | Letter |
| Malmberg J. Improved functional status in 16 years of follow-up of middle aged and elderly men and women in north eastern Finland. J Epidemiol. Communiy Health. | Data captured in Malberg 2006. |
| Mann J, McLean R, Te Morenga L. (2013). Evidence favours an association between saturated fat intake and coronary heart disease. BMJ 2013; 347. | Letter |
| Marmot MG, Syme SL, Kagan A, Kato H, Cohen JB, Belsky J. (1975). Epidemiologic studies of coronary heart disease and stroke in Japanese men living in Japan, Hawaii and California: prevalence of coronary and hypertensive heart disease and associated risk factors. American Journal of Epidemiology 102(6):514-25. | Background paper for methodology |
| Marques-Vidal P, Arveiler D, Evans A, Amouyel P, Ferrieres J, Luc G, Ducimetière P; PRIME Study Group. (2002). Awareness, treatment and control of hyperlipidaemia in middle-aged men in France and northern ireland in 1991-1993: the PRIME study. Prospective epidemiological study of myocardial infarction. Acta Cardiologica 57(2):117-23. | X-sect analysis |
| Marques-Vidal P, Arveiler D, Evans A, Montaye M, Bingham A, Ruidavets JB, McMaster D, Haas B, Amouyel P, Ducimetière P. (2000). Patterns of alcohol consumption in middle-aged men from France and Northern Ireland. The PRIME Study. European Journal of Clinical Nutrition 54(4):321-8. | X-sect |
| Marques-Vidal P, Montaye M, Haas B, Bingham A, Evans A, Juhan-Vague I, Ferrières J, Luc G, Amouyel P, Arveiler D, Yarnell J, Ruidavets JB, Scarabin PY, Ducimetière P. (2001). Relationships between alcoholic beverages and cardiovascular risk factor levels in middle-aged men, the PRIME study. Atherosclerosis 157(2):431-40. | X-sect |
| Martínez-González MA, Guillén-Grima F, De Irala J, Ruíz-Canela M, Bes-Rastrollo M, Beunza JJ, López del Burgo C, Toledo E, Carlos S, Sánchez-Villegas A. (2012). The Mediterranean Diet Is Associated with a Reduction in Premature Mortality among Middle-Aged Adults. Journal of Nutrition 142(9):1672-8. | Relevant but mean age at baseline 38, FU 7 years so outcomes at < 55 years |
| Masel MC, Raji M, Peek MK. (2010). Education and physical activity mediate the relationship between ethnicity and cognitive function in late middle-aged adults. Ethnicity and Health 15(3):283-302. | X-sect |
| Matthews KA, Abrams B, et al. (2001).Body mass index in mid-life women: relative influence of menopause, hormone use, and ethnicity.[Erratum appears in Int J Obes Relat Metab Disord 2002 Aug;26(8):1150]. International Journal of Obesity & Related Metabolic Disorders: Journal of the International Association for the Study of Obesity 25(6): 863-873. | X-sect analysis, BMI exposure |
| Matthews KA, Räikkönen K, Sutton-Tyrrell K, Kuller LH. (2004). Optimistic Attitudes Protect Against Progression of Carotid Atherosclerosis in Healthy Middle-Aged Women. Psychosomatic Medicine 66(5):640-4. | Exposure is optimism/pessimism |
| Medraś M, Słowińska-Lisowska M, Jóźków P. (2005). Impact of recreational physical activity on bone mineral density in middle-aged men. Aging Male 8(3-4):162-5. | X-sect analysis, retrospective identification of cases, controls. |
| Michikawa T, Inoue M, Sawada N, Iwasaki M, Tanaka Y, Shimazu T, Sasazuki S, Yamaji T, Mizokami M, Tsugane S; Japan Public Health Center-based Prospective Study Group. (2012). Development of a prediction model for 10-year risk of hepatocellular carcinoma in middle-aged Japanese: The Japan Public Health Center-based Prospective Study Cohort II. Preventive Medicine 55(2):137-43. | Prediction model |
| Mielke MM, Zandi PP, et l.(2010). The 32-year relationship between cholesterol and dementia from midlife to late life. Neurology 75: 1888–1895. | Exposure is cholesterol |
| Missault L, Witters N, Imschoot J. (2010). High cardiovascular risk and poor adherence to guidelines in 11 069 patients of middle age and older in primary care centres. European Journal of Cardiovascular Prevention & Rehabilitation 17(5):593-8. | X-sect, outcome is CV risk prediction rather than actual events |
| Mitnitski A, Song X, Rockwood K. (2007). Improvement and decline in health status from late middle age: Modeling age-related changes in deficit accumulation. Experimental Gerontology 42(11):1109-15. | Time trends for changes in health states |
| Miyake Y. (2000). Risk factors for non-fatal acute myocardial infarction in middle-aged and older Japanese. Fukuoka Heart Study Group. Japanese Circulation Journal 64(2):103-9. | X-sectional |
| Mohamed S, Bondi MW, Kasckow JW, Golshan S, Jeste DV. (2006). Neurocognitive functioning in dually diagnosed middle aged and elderly patients with alcoholism and schizophrenia. International Journal of Geriatric Psychiatry 21(8):711-8. | Participants had existing schizophrenia |
| Morgan GS, Gallacher J, Bayer A, Fish M, Ebrahim S, Ben-Shlomo Y. (2012). Physical activity in middle-age and dementia in later life: findings from a prospective cohort of men in Caerphilly, South Wales and a meta-analysis. Journal of Alzheimer's Disease 31(3): 569-580. | PA - dementia 16 yr Fup of Caerphilly cohort study |
| Mozaffarian D, Micha R, Wallace S. Effects on coronary heart disease of increasing polyunsaturated fat in place of saturated fat: a systematic review and meta-analysis of randomized controlled trials. PLoS Medicine 7:e1000252. | SR includes a number of studies in middle-aged people - check cited references |
| Murray ET, Hardy R, Strand BH, Cooper R, Guralnik JM, Kuh D. (2011). Gender and Life Course Occupational Social Class Differences in Trajectories of Functional Limitations in Midlife: Findings From the 1946 British Birth Cohort. Journals of Gerontology Series A: Biological Sciences and Medical Sciences 66(12):1350-9. | Prevalence study of functional limitation at midlife |
| Nakanishi N, Nakamura K, Matsuo Y, Suzuki K, Tatara K. (2000). Cigarette Smoking and Risk for Impaired Fasting Glucose and Type 2 Diabetes in Middle-Aged Japanese Men. Annals of Internal Medicine 1;133(3):183-91. | Age 46-47 at baseline, followed for 5 years, so outcomes at age <55 years. |
| Nakanishi N, Nakamura K, Suzuki K., Matsuo Y, Tatara K. (2000). Relation of body weight change to changes in atherogenic traits; a study of middle-aged Japanese obese male office workers. Industrial Health-Kawasaki *38*(2), 233-238. | Mean age 44-47 at baseline, 1 year follow up, intentional weight reduction |
| Nakanishi N, Suzuki K, Tatara K. (2003). Alcohol Consumption and Risk for Development of Impaired Fasting Glucose or Type 2 Diabetes in Middle-Aged Japanese Men. Diabetes Care 26(1):48-54 | Age 45-47 at baseline, 7 years of FU so outcomes at age <55years |
| Nakanishi N, Suzuki K. (2005). Daily Life Activity and the Risk of Developing Hypertension in Middle-aged Japanese Men. Archives of Internal Medicine 24;165(2):214-20. | Mean age 47-48 at baseline, range 35-59, 7 yr FU. Outcomes at age <55 years. Have excluded other papers from this cohort as outcomes <55 y. This one is borderline but just under age 55 yr cut off. |
| Nakanishi N, Takatorige T, Suzuki K. (2005). Cigarette smoking and the risk of the metabolic syndrome in middle-aged Japanese male office workers. Industrial Health 43(2):295-301. | Age 46-47 at baseline, followed for 7 years, so outcomes at age <55 years. |
| Nakanishi N, Yoshida H, Nakamura K, Suzuki K, Tatara K. (2001). Alcohol consumption and risk for hypertension in middle-aged Japanese men. Journal of Hypertension 19(5):851-5. | Age 42-45 at baseline, 9 years of FU so outcomes <55 years. |
| Nakanishi N, Kawashimo H, Nakamura K, Suzuki K, Yoshida, H, Uzura S, Tatara, K. (2001). Association of alcohol consumption with increase in aortic stiffness: a 9-year longitudinal study in middle-aged Japanese men.Industrial Health 39(1), 24-28. | Outcome is aortic stiffness |
| Naya M, Morita K, Yoshinaga K, Manabe O, Goto D, Hirata K, Katoh C, Tamaki N, Tsutsui H. (2011). Long-term smoking causes more advanced coronary endothelial dysfunction in middle-aged smokers compared to young smokers. European Journal of Nuclear Medicine and Molecular Imaging 38(3):491-8. | Comparison of smoking cessation between young and midlife smokers |
| Novak M, Björck L, Giang KW, Heden-Ståhl C, Wilhelmsen L, Rosengren A. (2012). Perceived stress and incidence of Type 2 diabetes: a 35-year follow-up study of middle-aged Swedish men. Diabetic Medicine 30(1):e8-16. | Exposure is perceived stress |
| Okazaki T, Himeno E, Nanri H, Ikeda M. (2001). Effects of a community-based lifestyle-modification program on cardiovascular risk factors in middle-aged women. Hypertension Research 24(6):647-53. | Intervention - review 3 |
| Opree SJ. (2012). Exploring casual effects of combining work and intergenerational support on depressive symptoms among middle-aged women. Ageing and Society, 1(1), 1-17. | X-sect |
| Otsuka R, Imai T, Kato Y, Ando F, Shimokata H. (2010). Relationship between number of metabolic syndrome components and dietary factors in middle-aged and elderly Japanese subjects. Hypertension Research 33(6):548-54. | X-sect |
| Owen CG, Whincup PH, Orfei L, Chou QA, Rudnicka AR, Wathern AK, Kaye SJ, Eriksson JG, Osmond C, Cook DG. (2009). Is body mass index before middle age related to coronary heart disease risk in later life? Evidence from observational studies. International Journal of Obesity 33(8):866-77. | Exposure is BMI |
| Pajak A, Kawalec E. (2005). Lifestyle characteristics and hypertension in the middle-aged population of Kraków. Blood Pressure Supplement 2:17-21. | X-sect |
| Pan A, Malik VS, Schulze MB, Manson JE, Willett WC, Hu FB. (2011). Plain-water intake and risk of type 2 diabetes in young and middle-aged women. American Journal of Clinical Nutrition 95(6):1454-60. | Mean age at baseline 36, range 25-42 |
| Paterson DH, Warburton DER. (2010). Physical activity and functional limitations in older adults: a systematic review related to Canada's Physical Activity Guidelines. International Journal of Behavioral Nutrition and Physical Activity 7:38. | SR in adults >65 yrs |
| Peacock JM, Folsom AR, Knopman DS, Mosley TH, Goff DC Jr, Szklo M. (2000). Dietary antioxidant intake and cognitive performance in middle-aged adults. Public Health Nutrition 3(3):337-43. | X-sect |
| Peila R, White LR, Petrovich H, Masaki K, Ross GW, Havlik RJ, Launer LJ. (2001). Joint Effect of the APOE Gene and Midlife Systolic Blood Pressure on Late-Life Cognitive Impairment: The Honolulu-Asia Aging Study. Stroke 32(12):2882-9. | Exposure is BP/APOE |
| Pereira MA, Schreiner PJ, Pankow JS, Williams RR, Higgins M, Province MA, Rao DC. (2000). The Family Risk Score for Coronary Heart Disease: Associations with Lipids, Lipoproteins, and Body Habitus in a Middle-Aged Bi-Racial Cohort: The ARIC Study. Annals of Epidemiology 10(4):239-45. | Combined risk factor score, includes non behavioural RF. |
| Pereira SMP, Ki M, Power C. (2012). Sedentary behaviour and biomarkers for cardiovascular disease and diabetes in mid-life: the role of television-viewing and sitting at work. PLoS One, 7(2), e31132. | X-sect |
| Peters R, Forette F, et al. (2008). Incident dementia and blood pressure lowering in the Hypertension in the Very Elderly Trial cognitive function assessment (HYVET-COG): a double-blind, placebo controlled trial. Lancet Neurology 7: 683–89. | Exposure is hypertension |
| Peters R, Peters J, et al. (2008). Alcohol, dementia and cognitive decline in the elderly: a systematic review. Age and Ageing 37: 505–512. | SR in those >65 yrs |
| Peters R, Poulter R, et al. (2008). Smoking, dementia and cognitive decline in the elderly, a systematic review. BMC Geriatrics 8:36. | SR in those >65 yrs |
| Peters R. (2012). Blood pressure, smoking and alcohol use, association with vascular dementia. Experimental Gerontology 47 865–872. | Review, not SR, not midlife |
| Piazza-Gardner AK, Gaffud TJB, Barry AE. (2013). The impact of alcohol on Alzheimer's disease: A systematic review. Aging & Mental Health 17:2, 133-146. | Alcohol SR, not midlife |
| Plassman, BL, Williams JW. (2010). Systematic Review: Factors Associated With Risk for and Possible Prevention of Cognitive Decline in Later Life. Annals of Internal Medicine 153:182-193. | Older adults, not midlife, I yr FU |
| Platz EA, Willett WC, Colditz GA, Rimm EB, Spiegelman D, Giovannucci E.(2000). Proportion of colon cancer risk that might be preventable in a cohort of middle-aged US men. Cancer Causes Control 11(7):579-88. | Combined risk factor score - includes non-behavioural risk factors |
| Podewils LJ, Guallar E, et al. (2005). Physical Activity, APOE Genotype, and Dementia Risk: Findings from the Cardiovascular Health Cognition Study. American Journal of Epidemiology 161:639–651. | >65 at baseline |
| Pope SK, Sowers M. (2005). Functional status and hearing impairments in women at midlife. Journals of Gerontology Series B: Psychological Sciences and Social Sciences 55(3):S190-4. | X-sect |
| Pope SK, Sowers MF, Welch GW, Albrecht G. (2001). Functional Limitations in Women at Midlife: The Role of Health Conditions, Behavioral and Environmental Factors. Womens Health Issues 11(6):494-502. | X-sect |
| Power MC, Weuve J, et al. (2011). The association between blood pressure and incident Alzheimer disease: a systematic review and meta-analysis. Epidemiology 22(5): 646–659. | Exposure is BP |
| Profenno LA, Porsteinsson AP, Faraone SV. (2010). Meta-Analysis of Alzheimer’s Disease Risk with Obesity, Diabetes, and Related Disorders. Biological Psychiatry 67:505–512 507. | Exposure is BMI, diabetes, MetS |
| Prospective Studies Collaboration. (2007). Blood cholesterol and vascular mortality by age, sex, and blood pressure: a meta-analysis of individual data from 61 prospective studies with 55 000 vascular deaths. Lancet 370:1829-39. | Exposure is BP and cholesterol |
| Pullen C, Noble Walker S. (2002). Midlife and Older Rural Women’s Adherence to U.S. Dietary Guidelines Across Stages of Change in Healthy Eating. Public Health Nursing 19(3):170-8. | X-sect |
| Qin L, Corpeleijn E, Jiang C, Thomas GN, Schooling CM, Zhang W, Cheng KK, Leung GM, Stolk RP, Lam TH. (2010). Physical Activity, Adiposity, and Diabetes Risk in Middle-Aged and Older Chinese Population. Diabetes Care 33(11):2342-8 | X-sect |
| Rantakömi SH, Laukkanen JA, Sivenius J, Kauhanen J, Kurl S. (2013). Hangover and the risk of stroke in middle-aged men. Acta Neurologic Scandinavica 127(3):186-91. | Exclude - drinking patterns - atherosclerosis as measured by ultrasound, not specific health conditions |
| Rantanen T. (2013). Midlife Fitness Predicts Less Burden of Chronic Disease in Later Life. Clinical Journal of Sports Medicine 23(6):499-500. | Exposure is physical fitness |
| Rasmussen M, Holstein BE, Due P. (2012) Tracking of overweight from mid-adolescence into adulthood: consistent patterns across socio-economic groups European Journal of Public Health 22 (6): 885–887. | Exposure is overweight in adolescence |
| Ravaglia G, Forti P, et al. (2008). Physical activity and dementia risk in the elderly. Findings from a prospective Italian study. Neurology. 70(19 Pt 2):1786-94. | Age >65 at baseline |
| Reis JP, Hankinson AL, Loria CM, Lewis CE, Powell-Wiley T, Wei GS, Liu K. (2013). Duration of Abdominal Obesity Beginning in Young Adulthood and Incident Diabetes Through Middle Age: the CARDIA study. Diabetes Care 36(5):1241-7. | Exposure is obesity |
| Rhee EJ, Oh KW, Lee WY, Kim SW, Oh ES, Baek KH, Kang MI, Park CY, Choi MG, Yoo HJ, Park SW. (2004). Age, body mass index, current smoking history, and serum insulin-like growth factor-I levels associated with bone mineral density in middle-aged Korean meen. Journal of Bone and Mineral Metabolism 22(4):392-8. | X-sect |
| Richards M, Hardy R, Wadsworth ME. (2005). Alcohol consumption and midlife cognitive change in the British 1946 birth cohort study. Alcohol and Alcoholism 40(2):112-7. | Outcomes in midlife |
| Richards M, Jarvis MJ, Thompson N, Wadsworth ME. (2003). Cigarette Smoking and Cognitive Decline in Midlife: Evidence From a Prospective Birth Cohort Study. American Journal of Public Health 93(6):994-8 | Outcomes in midlife |
| Ridley NJ, Draper B, Withall A. (2013). Alcohol-related dementia: an update of the evidence. Alzheimer’s Research & Therapy 5:3. | Review but not SR |
| Ritchie K, Carrie`re I, et al. (2007). The neuroprotective effects of caffeine. A prospective population study (the Three City Study). Neurology 69(6):536-45. | >65 y at baseline |
| Rohr G, Støvring H, Christensen K, Gaist D, Nybo H, Kragstrup J. (2005). Characteristics of middle-aged and elderly women with urinary incontinence. Scandinavian Journal of Primary Health Care 23(4):203-8. | X-sect |
| Rönnlund M, Sundström A, Sörman DE, Nilsson LG. (2013). Effects of Perceived Long- Term Stress on Subjective and Objective Aspects of Memory and Cognitive Functioning in a Middle-Aged Population-Based Sample. Journal of Genetic Psychology 174(1):25-41. | Exposure is perceived stress |
| Rundberg J, Lidfeldt J, Nerbrand C, Samsioe G, Romelsjö A, Ojehagen A. (2008). Abstinence, occasional drinking and binge drinking in middle-aged women. The Women’s Health in Lund Area (WHILA) Study. Nordic Journal of Psychiatry 62(3):186-91. | X-sect |
| Sabia S, Guéguen A, Marmot MG, Shipley MJ, Ankri J, Singh-Manoux A. (2010). Does cognition predict mortality in midlife? Results from the Whitehall II cohort study. Neurobiology of Aging 31(4):688-95 | Exposure is cognition |
| Sakurai M, Nakamura K, Miura K, Takamura T, Yoshita K, Nagasawa SY, Morikawa Y, Ishizaki M, Kido T, Naruse Y, Suwazono Y, Sasaki S, Nakagawa H. (2012). Self-reported speed of eating and 7-year risk of type 2 diabetes mellitus in middle-aged Japanese men. Metabolism 61(11):1566-71. | Age 46 at baseline, 7 y FU so outcomes at age <55y |
| Santos-Eggimann B, Cuénoud P, Spagnoli J, Junod J. (2009). Prevalence of Frailty in Middle-Aged and Older Community-Dwelling Europeans Living in 10 Countries. Journals of Gerontology Series A Biological Sciences & Medical Sciences 64(6):675-81. | X-sect |
| Savonen KP, Kiviniemi V, Laaksonen DE, Lakka TA, Laukkanen JA, Tuomainen TP, Rauramaa R. (2011). Two-minute heart rate recovery after cycle ergometer exercise and all-cause mortality in middle-aged men Journal of Internal Medicine 270(6):589-96. | Exposure is fitness related |
| Savonen KP, Lakka TA, Laukkanen JA, Halonen PM, Rauramaa TH, Salonen JT, Rauramaa R. (2006). Heart rate response during exercise test and cardiovascular mortality in middle-aged men. European Heart Journal 27(5):582-8. | Exposure is fitness related |
| Savva GM, Blossom CM, et al. (2010). Epidemiological Studies of the Effect of Stroke on Incident Dementia: A Systematic Review. Stroke 41:e41-e46. | Exposure is stroke risk - includes smoking but cannot be separated from other stroke risk factors |
| Scarmeas N, Luchsinger JA, et al. (2009). Physical Activity, Diet, and Risk of Alzheimer Disease. JAMA 302 (6):627-37. | Mean age 76-82 at baseline |
| Scarmeas N, Stern Y, et al. (2006). Mediterranean Diet, Alzheimer Disease, and Vascular Mediation. Archives of Neurology 63(12):1709-17. | X-sect analysis |
| Schuit AJ, Feskins EJM, et al. (2001). Physical activity and cognitive decline, the role of the apolipoprotein e4 allele. Medicine & Science in Sports & Exercise 33(5):772-7. | Mean age 74 at baseline |
| Schult A, Eriksson H, Wallerstedt S, Kaczynski J. (2011). Overweight and hypertriglyceridemia are risk factors for liver cirrhosis in middle-aged Swedish men. Scandinavian Journal of Gastroenterology 46(6):738-44. | OW/LTG as exposures |
| Schulze MB, Manson JE, Ludwig DS, Colditz GA, Stampfer MJ, Willett WC, Hu FB. (2004). Sugar-Sweetened Beverages, Weight Gain, and Incidence of Type 2 Diabetes in Young and Middle-Aged Women. JAMA. 292(8):927-34. | Mean age 36 at baseline |
| Seki A, Takigawa T, Ito T, Fukuoka E, Takahashi K, Kira S. (2002). Obesity and the risk of diabetes mellitus in middle-aged Japanese men. Acta Medica Okayama 56(5):255-60. | Exposure is obesity |
| Shaper AG, Wannamethee SG. (2000). Alcohol intake and mortality in middle-aged men with diagnosed coronary heart disease. Heart 83(4):394-9. | Study in those with existing CHD |
| Sharp SI, Aarsland D, et al. (2011).Hypertension is a potential risk factor for vascular dementia: systematic review. International Journal of Geriatric Psychiatry 26: 661–669. | Exposure is BP |
| Shay CM, Stamler J, Dyer AR, Brown IJ, Chan Q, Elliott P, Zhao L, Okuda N, Miura K, Daviglus ML, Van Horn L. (2012). Nutrient and food intakes of middle-aged adults at low risk of cardiovascular disease: the international study of macro-/ micronutrients and blood pressure (INTERMAP). European Journal of Nutrition 51(8):917-26. | X-sect |
| Shepherd JP, Shepherd I, Newcombe RG, Farrington D. (2009). Impact of antisocial lifestyle on health: chronic disability and death by middle age. Journal of Public Health 31(4):506-11. | Outcomes at 48 y (<55y), antisocial lifestyle |
| Sheu WH, Chuang SY, Lee WJ, Tsai ST, Chou P, Chen CH. (2006). Predictors of incident diabetes, metabolic syndrome in middle-aged adults: A 10-year follow-up study from Kinmen, Taiwan. Diabetes Research & Clinical Practice 74(2):162-8. | Exposure is baseline components of MetS |
| Shi J, Song X, Yu P, Tang Z, Mitnitski A, Fang X, Rockwood K. (2011). Analysis of frailty and survival from late middle age in the Beijing Longitudinal Study of Aging. BMC Geriatrics 20;11:17. | Exposure is frailty - mortality outcomes |
| Shimizu S, Kawata Y, Kawakami N, Aoyama H. (2001). Effects of Changes in Obesity and Exercise on the Development of Diabetes and Return to Normal Fasting Plasma Glucose Levels at One-Year Follow-up in Middle-Aged Subjects with Impaired Fasting Glucose. Environ Health & Preventive Medicine 6(2):127-31. | Intervention, consider review 3 |
| Shlomo YB, Kuh D. (2002). A life course approach to chronic disease epidemiology: conceptual models, empirical challenges and interdisciplinary perspectives. International Journal of Epidemiology 31: 285-193. | Model, not a primary study |
| Siervo M, Nasti G, Stephan BC, Papa A, Muscariello E, Wells JC, Prado CM, Colantuoni A. (2012). Effects of Intentional Weight Loss on Physical and Cognitive Function in Middle-Aged and Older Obese Participants: A Pilot Study. Journal of the American College of Nutrition 31(2):79-86. | Int - rev 3? Not midlife realtionship with older outcomes |
| Singh-Manoux A, Marmot M. (2005). High blood pressure was associated with cognitive function in middle-age in the Whitehall II study. Journal of Clinical Epidemiology 58(12):1308-15. | Exposure is BP |
| Skretteberg PT, Grundvold I, Kjeldsen SE, Engeseth K, Liestøl K, Erikssen G, Erikssen J, Gjesdal K, Bodegard J .(2013). Seven-Year Increase in Exercise Systolic Blood Pressure at Moderate Workload Predicts Long-Term Risk of Coronary Heart Disease and Mortality in Healthy Middle-Aged Men. Hypertension 61(5):1134-40. | Exposure is BP |
| Sluijs I, Beulens JW, Grobbee DE, van der Schouw YT. (2009). Dietary Carotenoid Intake Is Associated with Lower Prevalence of Metabolic Syndrome in Middle-Aged and Elderly Men. Journal of Nutrition 139(5):987-92. | X-sect |
| Smith ML, Honoré Goltz H, Ahn S, Dickerson JB, Ory MG. (2012). Correlates of chronic disease and patient–provider discussions among middle-aged and older adult males: Implications for successful aging and sexuality. The Aging Male 15(3):115-23. | X-sect |
| Smith-DiJulio K, Anderson D. (2009). Sustainability of a Multimodal Intervention to Promote Lifestyle Factors Associated with the Prevention of Cardiovascular Disease in Midlife Australian Women: A 5-year Follow-Up. Health Care for Women International 30(12):1111-30 | Int - rev 3? |
| Sofi F, Ceasri F et al. (2008). Adherence to Mediterranean diet and health status: meta-analysis. BMJ c337:a1344. | SR Med diet and health, not spec midlife, not spec follow up to older age. Checked ref list for midlife studies - Check Lagiou 2006, Fung 2006, Gao 2007, Mitrou 2007 |
| Sofi F, Valecchi D, et al (2011). Physical activity and risk of cognitive decline: a meta-analysis of prospective studies. Journal of Internal Medicine 269(1):107-17. | SR, not spec midlife, checked included studies list for midlife papers. |
| Soloman A, Kivipelto M, et al. (2009). Midlife Serum Cholesterol and Increased Risk of Alzheimer’s and Vascular Dementia Three Decades Later. Dementia and Geriatric Cognitive Disorders 28:75–80. |  |
| Solomon A, Kåreholt I, Ngandu T, Winblad B, Nissinen A, Tuomilehto J, Soininen H, Kivipelto M. (2007). Serum cholesterol changes after midlife and late-life cognition: twenty-one-year follow-up study. Neurology 6;68(10):751-6. | Exposure is cholesterol |
| Song Y, Ridker PM, Manson JE, Cook NR, Buring JE, Liu S. (2005). Magnesium Intake, C-Reactive Protein, and the Prevalence of Metabolic Syndrome in Middle-Aged and Older U.S. Women. Diabetes Care 28(6):1438-4. | X-sect |
| Sowers M, Zheng H, Tomey K, Karvonen-Gutierrez C, Jannausch M, Li X, Yosef M, Symons J. (2007). 6-year changes in body composition in women at mid-life: ovarian and chronological aging. Journal of Clinical Endocrinology & Metabolism 92(3):895-901. | Age- body composition |
| Stavem K, Aaser E, Sandvik L, Bjørnholt JV, Erikssen G, Thaulow E, Erikssen J. (2005). Lung function, smoking and mortality in a 26-year follow-up of healthy middle-aged males. European Respiratory Journal 25(4):618-25. | Exposure is baseline lung fn |
| Steptoe A, Owen N, Kunz-Ebrecht SR, Brydon L. (2004). Loneliness and neuroendocrine, cardiovascular, and inflammatory stress responses in middle-aged men and women. Psychoneuroendocrinology 29(5):593-611. | Not DDF outcomes |
| Stewart R, White LR, et al. (2007). Twenty-six–Year Change in Total Cholesterol Levels and Incident Dementia” Archives Neurology 64:103-107. | Exposure is cholesterol |
| Strandberg A, Strandberg TE, Salomaa VV, Pitkälä K, Häppölä O, Miettinen TA. (2004). A follow-up study found that cardiovascular risk in middle age predicted mortality and quality of life in old age. Journal of Clinical Epidemiology 57(4):415-21. | High vs low risk combined score includes non- behavioural RF |
| Strandberg TE, Sirola J, Pitkälä KH, Tilvis RS, Strandberg AY, Stenholm S. (2012). Association of midlife obesity and cardiovascular risk with old age frailty: a 26-year follow-up of initially healthy men. International Journal of Obesity 36(9):1153-7. | Exposure is midlife obesity/CVD risk |
| StStrandberg TE, Saijonmaa O, Tilvis RS, Pitkälä KH, Strandberg AY, Miettinen TA, Fyhrquist F. (2011). Association of telomere length in older men with mortality and midlife body mass index and smoking. Journals of Gerontology Series A: Biological Sciences and Medical Sciences 66(7):815-20. | Outcome is telomere length, not directly DDF outcomes |
| Sun Q, Townsend MK, Okereke OI, Franco OH, Hu FB, Grodstein F. (2009). Adiposity and weight change in mid-life in relation to healthy survival after age 70 in women: prospective cohort study. BMJ 29;339:b3796. | Exposure is BMI or weight change from 18 to midlife |
| Swanepoel de W, Eikelboom RH, Hunter ML, Friedland PL, Atlas MD. (2013). Self-reported hearing loss in baby boomers from the Busselton Healthy Ageing Study: audiometric correspondence and predictive value. Journal of the American Academy of Audiology 24(6):514-21. | Not a prospective study, comparison of self-reported hearing loss with objectively measured hearing loss. |
| Takwoingi Y, Hopewell S, Tovey D, Sutton AJ. (2013). A multicomponent decision tool for prioritising the updating of systematic reviews. BMJ 347:f7191. | Not relevant topic |
| Tanno K, Sakata K, Ohsawa M, Onoda T, Itai K, Yaegashi Y, Tamakoshi A; JACC Study Group. (2009). Associations of ikigai as a positive psychological factor with all-cause mortality and cause-specific mortality among middle-aged and elderly Japanese people: Findings from the Japan Collaborative Cohort Study. Journal of Psychosomatic Research 67(1):67-75. | Ikigai' psychological factor as exposure |
| Tatsuno I, Terano T, Nakamura M, Suzuki K, Kubota K, Yamaguchi J, Yoshida T, Suzuki S, Tanaka T, Shozu M. (2013). Lifestyle and osteoporosis in middle-aged and elderly women: Chiba bone survey. Endocrinology Journal 60(5):643-50. | X-sect |
| Thom DH, Brown JS, Schembri M, Ragins AI, Subak LL, Van Den Eeden SK. (2010). Incidence of and risk factors for change in urinary incontinence status in a prospective cohort of middle-aged and older women: The Reproductive Risk of Incontinence Study in Kaiser (RRISK). Journal of Urology 184(4):1394-401. | Not behavioural risk factors |
| Thornton EW, Sykes KS, Tang WK. (2004). Health benefits of Tai Chi exercise: improved balance and blood pressure in middle-aged women. Health Promotion International 19(1):33-8. | Intervention - rev 3? |
| Tice JA, Kanaya A, Hue T, Rubin S, Buist DS, Lacroix A, Lacey JV Jr, Cauley JA, Litwack S, Brinton LA, Bauer DC. (2006). Risk Factors for Mortality in Middle-aged Women. Archives of Intern Medicine 11-25;166(22):2469-77. | Smoking, 9y FU, mean age 68 at baseline |
| Tourlouki E, Matalas AL, Panagiotakos DB. (2009). Dietary habits and cardiovascular disease risk in middle-aged and elderly populations: a review of evidence. Clinical Interventions in Aging 4:319-30. | Review, not SR, >65 yrs |
| Tsai CC, Hsieh MH, Li AH, Chen PL, Jeng C. (2013). Dietary supplementation and engaging in physical activity as predictors of coronary artery disease among middle-aged women. Journal of Clinical Nursing 22(17-18):2487-98. | X-sect |
| Tsai SP, Donnelly RP, Wendt JK. (2006). Obesity and Mortality in a Prospective Study of a Middle-Aged Industrial Population. Journal of Occupational & Environmental Medicine 48(1):22-7. | Exposure is obesity |
| Tsuboi S, Hayakawa T, Kanda H, Fukushima T. (2009). The relationship between clustering health-promoting components of lifestyle and bone status among middle-aged women in a general population. Environmental Health & Preventive Medicine 14(5): 292-298. | X-sect |
| Tunstall-Pedoe H. (2013). The decline in coronary heart disease; did it fall or was it pushed? BMJ 344:d7809. | Editorial |
| van Dam RM, Willett WC, Manson JE, Hu FB. (2006). Coffee, Caffeine, and Risk of Type 2 Diabetes: a prospective cohort study in younger and middle-aged U.S. women. Diabetes Care 29(2):398-403. | Age 26-46 at baseline |
| van Gelder BM, Buijsse B, et al. (2007). Coffee consumption is inversely associated with cognitive decline in elderly European men: the FINE Study. European Journal of Clinical Nutrition 61, 226–232. | >65 at baseline |
| van Gelder BM, Tijhuis MAR, et al. (2004). Physical activity in relation to cognitive decline in elderly men. The FINE Study. Neurology 63(12):2316-21. | >65 at baseline |
| van Gool CH, Kempen GI, Penninx BW, Deeg DJ, Beekman AT, van Eijk JT. (2005). Impact of depression on disablement in late middle aged and older persons: results from the Longitudinal Aging Study Amsterdam. Social Science & Medicine 60(1):25-36. | Exposure is depression |
| van Vliet P. (2012). Cholesterol and Late-Life Cognitive Decline. Journal of Alzheimer’s Disease 30 S147–S162. | Exposure is cholesterol |
| Verghese J, Lipton RB, et al. (2003). Leisure Activities and the Risk of Dementia in the Elderly. The New England Journal of Medicine 348:2508-16.. | >75y at baseline |
| Verghese J, Wang C, et al (2009).Leisure Activities and Risk of Vascular Cognitive Impairment in Older Adults. Journal of Geriatric Psychiatry and Neuroogy 22(2): 110–118. | Age 75-85 y at baseline |
| Villegas R, Liu S, Gao YT, Yang G, Li H, Zheng W, Shu XO. (2007). Prospective Study of Dietary Carbohydrates, Glycemic Index, Glycemic Load, and Incidence of Type 2 Diabetes Mellitus in Middle-aged Chinese Women. Archives of Intern Medicine 26;167(21):2310-6. | FU 4.7 y (<5 y) |
| Virtanen JK, Voutilainen S, Rissanen TH, Happonen P, Mursu J, Laukkanen JA, Poulsen H, Lakka TA, Salonen JT. (2006). High dietary methionine intake increases the risk of acute coronary events in middle-aged men. Nutrition, Metabolism & Cardiovascular Diseases 16(2):113-20. | Exposure is dietary methionine |
| Voss R, Cullen P, Schulte H, Assmann G. (2002). Prediction of risk of coronary events in middle-aged men in the Prospective Cardiovascular Münster Study (PROCAM) using neural networks. International Journal of Epidemiology 31(6):1253-62. | Neural network modelling |
| Vuorinen M, Solomon A, Rovio S, Nieminen L, Kåreholt I, Tuomilehto J, Soininen H, Kivipelto M. (2011). Changes in vascular risk factors from midlife to late life and white matter lesions: a 20-year follow-up study. Dement and Geriatric Cognitive Disorders 31(2):119-25. | Outcome is white matter lesions but not specific health conditions. Exposures are BP, TC, BMI, ApoE. |
| Waetjen LE, Liao S, Johnson WO, Sampselle CM, Sternfield B, Harlow SD, Gold EB. (2007). Factors Associated with Prevalent and Incident Urinary Incontinence in a Cohort of Midlife Women: A Longitudinal Analysis of Data Study of Women’s Health Across the Nation. American Journal of Epidemiology 165(3):309-18. | Social support is one exposure - relevant?, Mean age 45 (range 42-52), 5y follow-up so outcomes at <55y |
| Weirich G, Bemben DA, Bemben MG. (2010). Predictors of balance in young, middle-aged, and late middle-aged women. Journal of Geriatric Physical Therapy 33(3): 110-117. | No behaviour |
| Weng LC, Steffen LM, Szklo M, Nettleton J, Chambless L, Folsom AR (2013). A diet pattern with more dairy and nuts, but less meat is related to lower risk of developing hypertension in middle-aged adults: the Atherosclerosis Risk in Communities (ARIC) study. Nutrients 5(5): 1719-1733. | The exposure we are interested in is diet and the outcome is hypertension/blood pressure (as a precondition for dementia, disability and frailty). Baseline measurements were taken at 1987-89 and follow-up at 1990-92 (Exam 2), 1993-95 (Exam 3) and 1996-98 (Exam 4). From the tables (3 and 4) the data seems to be reported for 1987-1998. So the outcome hypertension data was taken from exam 4 (1996-98). So between baseline measurements and Exam 4 there is about 9 years of follow-up. However, between exam 3 and exam 4 there is only 3 years of follow-up and it is not clear from the data reported how much of the analysis was based on 9 year follow-up and how much on 3 year follow up data. |
| Wennberg P, Andersson T, et al. (2000). Associations between different aspects of alcohol habits in adolescence, early adulthood, and early middle age: a prospective longitudinal study of a representative cohort of men and women. Psychology of Addictive Behaviors 14(3): 303-307. | Adolescent exposure - midlife outcomes |
| Whalley LJ, Dick FD, McNeil G. (2006). A life-course approach to the aetiology of late-onset dementias. Lancet Neurology 5: 87-96. | Review and model |
| Whisman MA. (2010). Loneliness and the Metabolic Syndrome in a Population-Based Sample of Middle-Aged and Older Adults. Health Psychology 29 (5): 550–554. | X-sect |
| White L. (2010). Educational attainment and mid-life stress as risk factors for dementia in late life. Brain 133; 2180–2184. | 18 months |
| White SM, Wójcicki TR, McAuley E. (2012). Social Cognitive Influences on Physical Activity Behavior in Middle-Aged and Older Adults. Journals of Gerontology Series A: Biological Sciences and Medical Sciences 64(5):543-50. | Model |
| Whitley E, Lee IM, Sesso HD, Batty GD. (2012). Association of cigarette smoking from adolescence to middle-age with later total and cardiovascular disease mortality: theHarvard Alumni Health Study. Journal of the American College of Cardiology 60(18): 1839-1840. | Letter not primary study |
| Whitmer RA, Gunderson EP, Barrett-Connor E, Quesenberry CP, Jr., Yaffe K. (2005). Obesity in middle age and future risk of dementia: a 27 year longitudinal population based study. BMJ 330(7504): 1360. | Exposure is obesity |
| Whitmer RA, Gunderson EP, Quesenberry CP, Jr., Zhou J, Yaffe K. (2007). Body mass index in midlife and risk of Alzheimer disease and vascular dementia. Current Alzheimer Research 4(2): 103-109. | Exposure is BMI |
| Whitmer RA, Karter AJ, Yaffe K, Quesenberry CP Jr, Selby JV. (2009). Hypoglycemic episodes and risk of dementia in older patients with type 2 diabetes mellitus. JAMA. 2009 Apr 15;301(15):1565-72 |  |
| WHO Ageing Website (accessed 26.11.13) | Information sheet only, not primary study |
| Wilbur J, A Vassalo, et al. (2005). Midlife women's adherence to home-based walking during maintenance. Nursing Research 54(1): 33-40. | Possible for rev 1 - check again |
| Wilson D, R. Peters, K. Ritchie, C. W. Ritchie. (2011). Latest advances on interventions that may prevent, delay or ameliorate dementia. Therapeutic Advances in Chronic Disease 2(3) 161-173. | Review but not SR, check refs |
| Wolinsky FD, Malmstrom TK, Miller JP, Andresen EM, Schootman M, Miller DK (2009). Antecedents of global decline in health-related quality of life among middle-aged African Americans. Journals of Gerontology Series B-Psychological Sciences & Social Sciences 64(2): 290-295. | No behaviour |
| Woodside JV, Yarnell JW, Patterson CC, Arveiler D, Amouyel P, Ferrieres J, et al. (2012). Do lifestyle behaviours explain socioeconomic differences in all-cause mortality, and fatal and non-fatal cardiovascular events? Evidence from middle aged men in France and Northern Ireland in the PRIME Study. Preventive Medicine 54(3-4): 247-253. | No behaviour |
| World Cancer Research Fund / American Institute for Cancer Research. (2007). Food, Nutrition, Physical Activity, and the Prevention of Cancer: a Global Perspective. Washington DC: AICR. |  |
| Wray LA, Alwin DF, McCammon RJ, Manning T, Best LE. (2006). Social status, risky health behaviors, and diabetes in middle-aged and older adults. Journals of Gerontology Series B-Psychological Sciences & Social Sciences 61(6): S290-298. | Clearly relevant exposures and outcomes but cannot tell which data is longitudinal > 5 years or cross-sectional so excluded on that basis. Contact authors? |
| Wright JL, Sherriff JL, Dhaliwal SS, Mamo JC. (2011). Tailored, iterative, printed dietary feedback is as effective as group education in improving dietary behaviours: results from a randomised control trial in middle-aged adults with cardiovascular risk factors. International Journal of Behavioral Nutrition & Physical Activity 8: 43. | Int - rev 3? |
| Xu WL, Atti AR, Gatz M, Pedersen NL, Johansson B, Fratiglioni L (2011). Midlife overweight and obesity increase late-life dementia risk: a population-based twin study. Neurology 76(18): 1568-1574. | Exposure is OW/Obesity |
| Yaffe K, Barnes D, et al. (2001). A prospective study of physical activity and cognitive decline in elderly women. Archives of Internal Medicine 296(19)-2343. | Exclude >65 at baseline |
| Yagci N, Cavlak U, Aslan UB, Akdag B. (2007). Relationship between balance performance and musculoskeletal pain in lower body comparison healthy middle aged and older adults. Archives of Gerontology & Geriatrics 45(1): 109-119. | Balance not behaviour |
| Yamada M, Kasagi F, Sasaki H, Masunari N, Mimori Y, Suzuki G. (2003). Association between dementia and midlife risk factors: the Radiation Effects Research Foundation Adult Health Study. Journal of the American Geriatrics Society 51(3): 410-414. | Mean age <40 y |
| Yan LL, Daviglus ML, Liu K, Stamler J, Wang R, Pirzada A, et al. (2006). Midlife body mass index and hospitalization and mortality in older age. JAMA 295(2): 190-198. | Cannot separate health behaviour data from other risk factors |
| Yang G, Shu XO, Gao YT, Zhang X, Li H, Zheng W. (2007). Impacts of weight change on prehypertension in middle-aged and elderly women. International Journal of Obesity 31(12): 1818-1825. | X-sect |
| Yang L, Kuper H, Sandin S, Margolis KL, Chen Z, Adami HO, et al. (2009). Reproductive history, oral contraceptive use, and the risk of ischemic and hemorrhagic stoke in a cohort study of middle-aged Swedish women. Stroke 40(4): 1050-1058. | Exposure is oral contraceptive use. |
| Yarnell JW, Patterson CC, Thomas HF, Sweetnam PM. (2000). Comparison of weight in middle age, weight at 18 years, and weight change between, in predicting subsequent 14 year mortality and coronary events: Caerphilly Prospective Study. Journal of Epidemiology & Community Health 54(5): 344-348. | Smoking - BMI relationship but smoking appears to be assessed at age 18 so not midlife. |
| Ye X, Gao X, Scott T, Tucker KL. (2011). Habitual sugar intake and cognitive function among middle-aged and older Puerto Ricans without diabetes. British Journal of Nutrition 106(9): 1423-1432. | X-sect analysis |
| Yoshida M, Ishikawa M, Kokaze A, Sekine Y, Matsunaga N, Uchida Y, Takashima Y. (2003). Association of life-style with intraocular pressure in middle-aged and older Japanese residents. Japanese Journal of Ophthalmology 47(2):191-8. | X-sect analysis of lifestyle, intraocukar pressure. Age range 29-79 so not midlife specifically |
| Zhang X, Zhang S, Li Y, Detrano RC, Chen K, Li X, et al. (2009). Association of obesity and atrial fibrillation among middle-aged and elderly Chinese. International Journal of Obesity 33(11): 1318-1325. | X-sect |
