# Supplementary material for: Behavioural Risk Factors in Mid-Life Associated with Successful Ageing, Disability, Dementia and Frailty in Later Life: A Rapid Systematic Review
Source: PLoS One. 2016 Feb 4;11(2):e0144405. doi: 10.1371/journal.pone.0144405 (PMC4742275; doi:10.1371/journal.pone.0144405)
Supplement: S2 Table — (DOCX) [file pone.0144405.s002.docx]

Quality Assessment of Included Studies

1. Quality Summary of Cohort Studies

Key to headings – Section 1: Population; 1.1 source population; 1.2 eligible population; 1.3 selected participants or areas. Section 2: Methods of Selection; 2.1 comparison group; explanatory variables; 2.3 contamination; 2.4 confounding factors; 2.5 setting applicability to the UK. Section 3: Outcomes; 3.1 reliable outcome measures; 3.2 outcome measurement; 3.3 important outcomes assessed; 3.4 follow-up time in exposure;. NA: not applicable; NR: not reported.

|  | **Population** | | | | | | | | **Method of selection of exposure**  **(or comparison) group** | | | | | | | **Outcomes** | | | | | | | |
| --- | --- | --- | --- | --- | --- | --- | --- | --- | --- | --- | --- | --- | --- | --- | --- | --- | --- | --- | --- | --- | --- | --- | --- |
| **Author (Year)** | **1** | | **1.2** | **1.2b** | **1.3.** | **1.3b** | **1.3c** | **1.3d** | **2.1** | **2.2** | **2.3** | **2.3b** | **2.4a** | **2.4b** | **2.5** | **3.1** | **3.1b** | **3.1c** | **3.2** | **3.3** | **3.3b** | **3.4** | **3.4b** |
| Agahi (2013) | - | | - | - | - | - | - | - | + | + | NA | NA | - | - | + | - | NR | NR | + | NA | NA | NA | NA |
| Agrigoroarei 2011 | + | | ++ | + | ++ | + | + | ++ | + | ++ | NA | NA | + | - | - | + | NR | NR | - | NA | NA | NA | NA |
| Akbaraly (2013) | + | | + | - | + | + | - | + | - | ++ | NA | NA | - | - | ++ | ++ | NR | + | ++ | NA | NA | ++ | ++ |
| Alonso (2009) | - | | - | - | - | ++ | - | + | + | ++ | NA | NA | + | + | - | ++ | NR | NR | ++ | NA | NA | ++ | ++ |
| Andel (2008) | - | | + | - | + | + | - | - | + | + | NA | NA | + | + | - | + | + | + | + | + | NA | NA | - |
| Anttila (2004) | - | | - | - | - | ++ | + | - | + | + | NA | NA | + | + | - | ++ | NR | NR | + | NA | NA | ++ | ++ |
| Ascherio (2001) | + | | ++ | - | - | + | - | + | + | + | NA | NA | - | - | - | ++ | NR | NR | ++ | NA | NA | NA | NA |
| Baba (2006) | - | | - | - | - | ++ | NR | ++ | ++ | ++ | NA | NA | - | + | - | ++ | NR | NR | ++ | NA | NA | ++ | ++ |
| Beulens (2007) | + | | + | NR | + | ++ | ++ | ++ | ++ | ++ | NA | NA | ++ | ++ | + | ++ | NR | NR | + | NA | NA | ++ | ++ |
| Beulens (2008) | - | | - | - | - | NR | + | + | ++ | ++ | NA | NA | + | + | - | - | NR | ++ | + | NA | NA | ++ | ++ |
| Bielak (2012) | + | | ++ | + | ++ | ++ | + | ++ | ++ | ++ | NA | NA | + | + | - | + | + | + | + | + | NA | NA | - |
| Blanco-Cedres 2002 | + | | ++ | + | + | + | + | + | - | NR | NA | NA | - | - | - | ++ | + | - | + | NA | NA | NA | ++ |
| Boudik (2004) | - | | - | - | + | - | NR | - | NR | - | NA | NA | - | - | - | ++ | + | - | - | NA | NA | NA | NA |
| Britton (2008) | - | | + | - | + | ++ | + | ++ | NR | NR | NA | NA | - | - | ++ | + | + | - | ++ | NA | NA | NA | NA |
| Carlson (2008) | + | | ++ | + | + | ++ | + | ++ | NR | ++ | NA | NA | ++ | ++ | - | + | NR | ++ | + | NA | NA | NA | NA |
| Chang (2010) | - | | - | - | - | NR | - | - | NR | NR | NA | NA | - | - | - | ++ | ++ | NR | ++ | NA | NA | NA | NA |
| Chang (2013) | - | | - | - | - | + | + | + | NR | NR | NA | NA | - | - | - | ++ | + | - | ++ | NA | NA | ++ | NA |
| Christensen (2006) | - | | - | - | - | ++ | NR | NR | - | ++ | NA | NA | - | - | + | - | NR | NR | + | NA | NA | NA | NA |
| Debette (2011) | . | | ++ | - | ++ | + | - | ++ | + | + | NA | NA | + | - | - | + | NR | NR | - | NA | NA | NA | - |
| Dudas (2007) | - | | ++ | + | ++ | ++ | ++ | ++ | + | + | NA | NA | + | - | + | - | - | - | ++ | ++ | NA | NA | - |
| Ekelund (2005) | - | | ++ | + | - | - | + | - | + | ++ | NA | NA | + | + | ++ | ++ | + | + | + | + | NA | + | ++ |
| Elwood (2013) | + | | + | + | ++ | ++ | + | ++ | NR | ++ | NA | NA | ++ | ++ | ++ | ++ | + | ++ | ++ | + | NA | NA | NA |
| Emberson (2005) | - | | ++ | + | + | ++ | - | - | - | + | NA | NA | - | - | ++ | - | - | - | - | + | NA | NA | ++ |
| Englund (2011) | - | | - | - | - | - | - | + | - | + | NA | NA | - | - | + | - | - | - | - | + | NA | NA | - |
| Englund (2013) | - | | - | - | - | - | - | + | - | + | NA | NA | - | - | + | - | - | - | - | + | NA | NA | - |
| Eskelinen (2008) | - | | + | - | - | + | - | - | - | + | NA | NA | + | + | + | - | + | + | + | + | NA | NA | - |
| Eskelinen (2009) | + | | + | - | - | ++ | + | - | NR | + | NA | NA | - | - | - | ++ | + | - | + | NA | NA | NA | NA |
| Field (2009) | - | | + | + | - | + | + | + | + | + | NA | NA | + | + | - | + | + | + | + | + | NA | NA | ++ |
| Fogelholm (2000) | - | | + | - | + | - | + | + | + | + | NA | NA | - | - | ++ | - | NR | NR | + | NA | NA | + | + |
| Friedland (2001) | - | | + | + | + | + | - | + | + | + | NA | NA | - | - | - | ++ | ++ | ++ | + | + | NA | NA | - |
| Gerber (2012) | ++ | | + | + | + | + | + | ++ | ++ | ++ | NA | NA | ++ | ++ | + | ++ | ++ | ++ | ++ | ++ | NA | NA | NA |
| Guallar-Castillon (2012) | + | | + | + | - | + | + | - | - | + | NA | NA | + | + | - | ++ | ++ | + | ++ | + | NA | + | + |
| Haaponen-Niemi (2000) | - | | ++ | ++ | + | ++ | + | + | + | + | NA | NA | + | + | - | ++ | ++ | + | ++ | + | NA | + | + |
| Halperin (2008) | - | | - | - | - | - | + | - | - | + | - | - | + | + | - | + | - | - | + | + | - | + | + |
| Hamer (2013) | - | | ++ | + | ++ | ++ | + | ++ | + | ++ | NA | NA | ++ | ++ | ++ | - | - | + | + | + | NA | + | - |
| Happonen (2004) | - | | - | - | - | - | - | - | - | + | NA | NA | + | + | + | ++ | + | + | ++ | + | NA | + | + |
| Hara (2002) | - | | - | - | - | - | + | - | - | + | NA | NA | + | + | - | - | + | + | ++ | + | NA | + | ++ |
| Harmsen (2006) | - | | + | - | - | + | + | - | + | + | NA | NA | - | - | ++ | + | - | + | ++ | + | NA | + | ++ |
| He (2004) | + | | + | NR | - | NR | - | + | + | ++ | NA | NA | - | - | - | - | NR | NR | + | NA | NA | ++ | + |
| Hodge (2013) | - | | + | ++ | + | + | + | + | + | + | NA | NA | + | + | - | + | + | + | + | + | NA | NA | - |
| Holmberg (2006) | - | | - | NR | - | + | + | - | ++ | - | NA | NA | + | + | + | ++ | NR | NR | ++ | NA | NA | - | - |
| Holme (2007) | + | | + | + | + | - | - | + | - | ++ | NA | NA | - | - | ++ | ++ | ++ | NR | ++ | NA | NA | ++ | ++ |
| Holtermann (2009) | - | | - | NR | - | ++ | + | + | + | ++ | NA | NA | - | - | + | ++ | NR | NR | + | NA | NA | ++ | NA |
| Holtzman (2004) | + | | + | - | + | ++ | + | + | + | ++ | NA | NA | ++ | ++ | - | + | NR | NR | + | NA | NA | NA | NA |
| Hu (2003) | - | | - | - | - | + | + | + | + | + | NA | NA | + | + | - | - | + | - | + | + | NA | NA | ++ |
| Hu (2004) | - | | + | + | + | + | + | + | + | + | NA | NA | + | + | - | ++ | ++ | ++ | ++ | + | NA | NA | ++ |
| Hu (2005) | - | | + | - | + | + | + | + | + | + | NA | NA | + | + | - | ++ | ++ | ++ | ++ | + | NA | NA | ++ |
| Hu (2007) Mov Disord | - | | - | - | - | + | + | + | + | + | NA | NA | + | + | - | + | + | + | + | + | NA | NA | ++ |
| Hughes (2010) | - | | - | - | - | - | - | + | + | + | NA | NA | ++ | + | - | + | + | + | + | + | NA | NA | - |
| Humphries (2001) | - | | - | - | - | + | + | + | - | + | NA | NA | + | + | ++ | ++ | + | ++ | + | + | NA | + | + |
| Inoue (2004) | + | | + | + | + | ++ | + | + | + | + | NA | NA | + | + | - | - | - | - | + | + | NA | + | + |
| Iso (2004) | - | | - | + | + | ++ | + | + | + | + | NA | NA | + | + | - | - | - | - | + | + | NA | + | + |
| Jakobsen (2009) | - | | + | + | - | - | + | + | + | ++ | NA | NA | ++ | + | - | + | + | + | + | + | NA | NA | ++ |
| Janzon (2004) | - | | - | - | - | + | - | - | - | + | NA | NA | + | + | - | + | + | + | + | + | NA | NA | + |
| Johnsen (2006) | - | | - | + | - | - | - | - | - | + | NA | NA | + | - | - | + | + | + | + | + | NA | NA | + |
| Kareholt (2011) | - | | - | - | + | + | + | - | + | + | NA | NA | + | + | - | + | - | ++ | - | - | NA | NA | + |
| Kato (2009) | + | | - | + | - | + | + | - | + | + | NA | NA | + | + | - | + | + | - | - | + | NA | - | - |
| Kesse-Guyot (2012) | - | | - | - | - | - | - | - | - | + | + | + | + | + | - | + | - | + | - | + | - | + | + |
| Khalili (2002) | + | | - | - | - | + | + | - | - | + | NA | NA | - | - | - | + | + | + | + | + | NA | NA | ++ |
| Kesse-Guyot (2012) | - | | - | - | - | - | - | - | - | + | + | + | + | + | - | + | - | + | - | + | - | + | + |
| Khalili (2002) | + | | - | - | - | + | + | - | - | + | NA | NA | - | - | - | + | + | + | + | + | NA | NA | ++ |
| Kimm (2011) | - | | + | + | - | ++ | + | + | + | + | NA | NA | - | - | - | + | - | - | + | + | NA | + | + |
| King (2007) | - | | + | + | + | - | - | - | + | + | NA | NA | + | + | - | + | + | + | + | + | NA | NA | - |
| Knopman (2001) | + | | ++ | + | ++ | - | + | ++ | + | + | NA | NA | + | + | - | ++ | + | + | + | NA | NA | ++ | + |
| Lahti (2010) | - | | + | + | + | + | + | + | ++ | + | NA | NA | - | + | - | - | + | + | - | + | NA | NA | - |
| Laitala (2009) | + | | ++ | + | + | ++ | ++ | ++ | ++ | ++ | NA | NA | ++ | ++ | ++ | ++ | ++ | NR | + | NA | NA | ++ | + |
| Laitinen (2006) | - | | + | - | - | ++ | + | + | + | + | NA | NA | - | + | - | ++ | + | NR | ++ | NA | NA | NA | NA |
| Lajous (2013) | + | | - | + | + | NR | + | ++ | ++ | ++ | NA | NA | ++ | ++ | - | ++ | + | + | + | NA | NA | ++ | + |
| Lang (2007) | + | | + | + | + | + | + | + | + | + | NA | NA | - | - | - | + | + | NR | + | NA | NA | + | + |
| Langlois (2001) | + | | - | + | - | ++ | - | + | + | ++ | NA | NA | + | + | - | + | ++ | NR | ++ | NA | NA | ++ | ++ |
| Laurin (2004) | + | | - | + | - | ++ | + | ++ | ++ | + | NA | NA | + | + | - | ++ | ++ | NR | + | NA | NA | ++ | ++ |
| Lehto (2013) | - | | - | - | - | - | - | - | + | + | NA | NA | + | + | - | + | + | + | - | - | NA | NA | - |
| Lesodottir 2007 | + | | ++ | + | + | - | + | + | + | ++ | NA | NA | + | + | + | ++ | + | + | + | NA | NA | NA | + |
| Levitan (2007) | - | | + |  | - | - | + | + | + | + | NA | NA | + | + | - | ++ | ++ | ++ | ++ | + | NA | NA | - |
| Levitan (2009) | - | | + |  | - | - | + | + | + | + | NA | NA | + | + | - | ++ | ++ | ++ | ++ | + | NA | NA | ++ |
| Levitan (2010) | - | | + | + | - | - | + | + | + | + | NA | NA | + | + | - | ++ | ++ | ++ | ++ | + | NA | NA | ++ |
| Lim (2013) | + | | - | + | + | ++ | + | + | + | + | NA | NA | + | + | - | + | + | + | + | NA | NA | ++ | ++ |
| Lin (2003) | ++ | | ++ | ++ | + | - | + | + | + | + | NA | NA | + | + | - | + | + | + | + | ++ | NA | NA | ++ |
| Liu (2003) | + | | + | + | + | - | + | ++ | ++ | ++ | NA | NA | + | + | - | - | NR | ++ | + | NA | NA | ++ | + |
| Liu (2004) | + | | ++ | ++ | ++ | ++ | + | ++ | ++ | + | NA | NA | + | - | - | ++ | + | + | ++ | NA | NA | + | ++ |
| Malmberg (2006) | + | | + | + | - | ++ | + | - | - | + | NA | NA | + | + | - | - | - | - | - | - | NA | NA | + |
| Mannami (2004) | + | | - | + | - | - | + | + | + | + | NA | NA | + | + | - | + | ++ | + | + | + | NA | NA | ++ |
| Masaki (2003) | - | | + | - | + | - | + | + | + | + | NA | NA | + | + | - | + | + | + | + | + | NA | NA | ++ |
| Meisinger (2007) | - | | + | + | + | + | + | + | + | + | NA | NA | - | - | - | ++ | + | + | + | + | NA | NA | ++ |
| Menotti (2000) | + | | - | + | - | + | + | - | - | + | NA | NA | + | + | - | + | + | + | ++ | + | NA | NA | - |
| Menotti (2006) | + | | - | - | - | + | + | - | - | ++ | NA | NA | ++ | ++ | - | + | + | + | ++ | + | NA | NA | - |
| Menotti (2012) | - | | - | - | - | + | + | + | + | + | NA | NA | + | + | + | + | NR | NR | - | NA | NA | + | + |
| Meyer (2011) | - | | - | - | - | + | + | + | + | + | NA | NA | + | + | + | + | NR | NR | - | NA | NA | + | + |
| Miura (2004) | + | | - | + | - | - | + | - | ++ | ++ | NA | NA | + | + | ++ | ++ | + | ++ | + | NA | NA | ++ | ++ |
| Moayyeri (2009) | - | | + | + | + | + | + | - | + | + | NA | NA | + | + | - | ++ | ++ | ++ | + | + | NA | NA | - |
| Morgan (2012) | - | | + | + | + | ++ | + | + | + | + | NA | NA | + | + | ++ | + | + | + | + | + | NA | NA | + |
| Mursu (2008) | - | | - | - | - | ++ | + | - | - | + | NA | NA | + | + | - | ++ | ++ | ++ | ++ | + | NA | NA | ++ |
| Nafziger (2007) | + | | - | - | - | + | + | + | - | - | - | - | + | + | - | ++ | + | + | ++ | + | NA | NA | - |
| Nakamura (2009) | + | | + | + | ++ | + | + | + | + | + | NA | NA | - | - | - | + | + | + | ++ | + | - | + | - |
| Nakayama (2000) | + | | - | + | - | + | - | + | - | + | NA | NA | + | + | - | + | + | + | + | + | NA | NA | - |
| Noborisaka (2013) | - | | + | + | - | + | + | - | + | + | NA | NA | + | + | - | + | + | + | + | + | NA | NA | - |
| Nokes (2012) | + | | ++ | + | + | + | + | + | + | ++ | NA | NA | + | + | + | ++ | + | ++ | ++ | + | NA | NA | NA |
| Nooyens (2008) | - | | + | + | + | + | + | + | + | + | NA | NA | + | + | - | + | + | + | + | + | NA | NA | - |
| Nooyens (2011) | - | | + | - | + | + | + | - | + | + | NA | NA | + | + | - | + | + | ++ | - | + | NA | NA | - |
| Osler (2003) | + | | + | + | + | + | + | ++ | ++ | + | NA | NA | + | + | ++ | ++ | + | NR | + | NA | NA | + | ++ |
| Ostbye (2002) | - | | - | + | - | NR | + | + | + | + | NA | NA | + | + | - | - | NR | NR | + | NA | NA | ++ | + |
| Ostenson (2012) | - | | - | ++ | - | + | + | + | + | + | NA | NA | + | + | - | ++ | ++ | ++ | ++ | + | NA | NA | + |
| Otani (2003) | + | | - | + | + | - | + | - | + | + | NA | NA | + | + | - | ++ | ++ | ++ | ++ | + | NA | NA | ++ |
| Patel (2006) | - | | + | + | + | ++ | + | - | + | + | NA | NA | + | + | - | ++ | + | + | + | + | NA | NA | + |
| Patja (2005) | ++ | | - | + | + | + | - | - | + | + | NA | NA | ++ | ++ | + | ++ | ++ | ++ | ++ | + | NA | NA | NA |
| Pelkonen (2000) | - | | - | + | - | ++ | + | + | + | + | NA | NA | - | - | - | ++ | ++ | ++ | ++ | + | NA | NA | + |
| Pitsavos (2004) | - | | - | ++ | - | ++ | + | + | + | + | NA | NA | + | + | - | ++ | ++ | ++ | ++ | + | NA | NA | ++ |
| Preis (2010) | - | | - | - | - | - | - | + | + | + | NA | NA | ++ | ++ | - | ++ | ++ | ++ | ++ | + | NA | NA | ++ |
| Qiao (2000) | - | | - | + | - | - | - | + | - | + | NA | NA | - | - | - | ++ | ++ | ++ | ++ | + | NA | NA | ++ |
| Qiu (2003) | - | | + | + | + | ++ | + | + | ++ | + | NA | NA | + | + | - | ++ | ++ | ++ | ++ | + | NA | NA | ++ |
| Raikkonen (2001) | - | | + | + | - | - | + | + | + | + | NA | NA | + | + | - | + | + | + | + | + | NA | NA | - |
| Rantakomi (2009) | + | | + | + | + | - | + | + | + | + | NA | NA | + | + | - | ++ | ++ | ++ | ++ | + | NA | NA | - |
| Ravona-Springer (2013) | - | | + | + | + | + | + | + | + | + | NA | NA | + | + | + | NA | NA | - | + | + | + | NA | NA |
| Riserus (2007) | + | | + | + | + | ++ | + | ++ | + | ++ | NA | NA | + | + | NA | NA | ++ | + | + | + | NA | NA | ++ |
| Ross (2000) | - | | - | + | - | - | - | - | + | + | NA | NA | + | + | + | NA | NA | ++ | + | + | + | NA | NA |
| Rovio (2005) | - | | + | + | + | + | + | ++ | ++ | + | NA | NA | + | + | + | NA | NA | - | + | + | + | NA | NA |
| Rovio (2007) | - | | + | + | + | + | + | ++ | ++ | + | NA | NA | + | + | + | NA | NA | - | + | + | + | NA | NA |
| Ruder (2011) | + | | ++ | ++ | ++ | ++ | ++ | ++ | ++ | ++ | NA | NA | + | ++ | NA | NA | NA | NA | + | ++ | NA | NA | NA |
| Rusanen (2011) | - | | + | + | + | + |  | + | + | ++ | NA | NA | + | + | + | NA | NA | - | + | + | + | NA | NA |
| Ruusunen (2010) | - | | - | + | - | - | - | ++ | + | + | NA | NA | + | + | + | NA | NA | - | + | + | + | NA | NA |
| Sabia (2008) | + | | ++ | + | ++ | + | + | + | + | ++ | NA | NA | + | + | NA | NA | ++ | + | + | + | NA | NA | ++ |
| Sabia (2009) | + | | ++ | + | ++ | - | + | + | + | ++ | NA | NA | + | + | NA | NA | ++ | + | + | + | NA | NA | ++ |
| Sabia (2011) | ++ | | ++ | + | ++ | - | + | ++ | + | + | NA | NA | + | + | NA | NA | ++ | + | + | + | NA | NA | ++ |
| Sairenchi (2004) | + | | + | + | + | - | + | ++ | ++ | - | NA | NA | + | ++ | NA | NA | ++ | ++ | + | ++ | NA | NA | ++ |
| Samieri (2013) | - | | + | + | - | - | + | + | + | + | NA | NA | + | + | + | NA | NA | - | + | + | + | NA | NA |
| Satoh (2006) | + | | - | + | - | - | - | + | + | + | NA | NA | ++ | ++ | + | NA | NA | - | ++ | ++ | + | NA | NA |
| Seccareccia (2003) | + | | + | + | + | ++ | + | + | + | + | NA | NA | ++ | ++ | + | NA | NA | - | ++ | ++ | + | NA | NA |
| Shaper (2003) | ++ | | ++ | ++ | ++ | + | + | ++ | + | + | NA | NA | + | + | + | NA | NA | ++ | + | + | + | NA | NA |
| Sobue (2002) | ++ | | + | + | - | + | + | ++ | + | + | NA | NA | ++ | ++ | + | NA | NA | ++ | ++ | ++ | + | NA | NA |
| Song (2004) | - | | - | + | - | ++ | - | + | + | + | - | - | + | + | + | - | - | ++ | + | + | + | - | - |
| Song (2006) | - | | - | + | - | ++ | - | + | + | + | - | + | + | + | - | - | + | ++ | + | + | - | - | + |
| Stevens (2009) | - | | ++ | ++ | ++ | ++ | + | ++ | + | + | NA | NA | ++ | ++ | + | NA | NA | ++ | ++ | ++ | + | NA | NA |
| Strand (2013) | - | | - | + | ++ | ++ | + | ++ | + | + | NA | NA | + | ++ | + | NA | NA | ++ | + | ++ | + | NA | NA |
| Strandberg (2008) | - | | + | - | + | ++ | + | - | + | + | NA | NA | + | ++ | + | NA | NA | ++ | + | ++ | + | NA | NA |
| Strandhagen (2000) | - | | ++ | ++ | ++ | + | + | + | + | + | NA | NA | + | ++ | + | NA | NA | - | + | ++ | + | NA | NA |
| Sun (2010) | - | | - | + | - | ++ | + | - | + | ++ | NA | NA | ++ | ++ | + | NA | NA | - | ++ | ++ | + | NA | NA |
| Sun (2011) | + | | + | + | - | ++ | + | + | + | ++ | NA | NA | ++ | ++ | NA | NA | ++ | + | ++ | ++ | NA | NA | ++ |
| Szoeke (2006) | - | | + | + | ++ | + | + | + | + | + | NA | NA | ++ | ++ | + | NA | NA | - | ++ | ++ | + | NA | NA |
| Tabak (2001) | - | | - | + | - | NR | + | - | + | + | NA | NA | + | + | NA | NA | + | ++ | + | + | NA | NA | + |
| Tsugane (2004) | - | | + | + | - | - | - | + | + | + | NA | NA | + | + | + | NA | NA | ++ | + | + | + | NA | NA |
| Tuomilehto (2004) | - | | - | - | - | + | - | + | + | + | NA | NA | + | + | + | NA | NA | ++ | + | + | + | NA | NA |
| Tyas (2003) | - | | + | ++ | + | + | + | + | + | + | NA | NA | + | + | + | NA | NA | - | + | + | + | NA | NA |
| Valtonen (2002) | + | | + | + | - | + | + | + | + | + | NA | NA | + | + | + | + | + | - | + | + | NA | NA | NA |
| Villegas (2010) | ++ | | ++ | ++ | ++ | ++ | ++ | ++ | ++ | + | NA | NA | + | + | + | NA | NA | ++ | + | + | + | NA | NA |
| Villegas (2011) | ++ | | ++ | ++ | ++ | ++ | ++ | ++ | ++ | + | NA | NA | + | + | + | NA | NA | ++ | + | + | + | NA | NA |
| Virta (2010) | + | | + | ++ | + | ++ | ++ | + | ++ | ++ | NA | NA | + | + | + | ++ | ++ | ++ | ++ | NA | NA | NA | ++ |
| Waki (2005) | + | | - | + | - | + | + | ++ | + | - | NA | NA | + | - | NA | NA | NA | NA | + | - | NA | NA | NA |
| Walda (2002) | + | | - | ++ | - | ++ | + | + | + | + | NA | NA | + | + | + | NA | NA |  | + | + | + | NA | NA |
| Wang (2008) | - | | - | - | - | + | + | + | + | ++ | NA | NA | + | + | NA | NA | NA | NA | + | + | NA | NA | NA |
| Wang (2009) | + | | NR | NR | NR | NR | NR | + | NR | ++ | NA | NA | ++ | ++ | ++ | ++ | ++ | ++ | ++ | + | NA | NA | NA |
| Wang (2012) | + | | NR | NR | NR | NR | NR | + | NR | ++ | NA | NA | ++ | ++ | ++ | + | + | + | + | + | NA | + | + |
| Wannamethee (2001a) Br.J. Canc. | ++ | | ++ | + | ++ | ++ | + | ++ | + | ++ | NA | NA | + | + | ++ | ++ | ++ | ++ | ++ | + | + | NA | NA |
| Wannamethee (2001b) Diab. Care | ++ | | ++ | + | ++ | ++ | + | ++ | + | ++ | NA | NA | + | + | ++ | ++ | ++ | ++ | ++ | + | + | NA | NA |
| Wannamethee (2002) | - | | + | + | + | ++ | + | + | + | + | NA | NA | - | + | + | NA | NA | ++ | - | + | + | NA | NA |
| Wannamethee (2003) | - | | + | - | - | + | - | - | + | - | NA | NA | NR | + | NA | NA | NA | NA | NR | + | NA | NA | NA |
| Waring (2010) | ++ | | ++ | ++ | ++ | - | + | + | ++ | ++ | NA | NA | ++ | ++ | + | NA | NA | ++ | ++ | ++ | + | NA | NA |
| Whitmer (2005) | - | - | | + | - | - | - | - | - | + | NA | NA | + | + | + | NA | NA | + | + | + | + | NA | NA |
| Willcox (2006) | ++ | ++ | | + | + | NR | + | ++ | NR | ++ | NA | NA | + | + | ++ | ++ | ++ | + | ++ | + | NA | NA | ++ |
| Wiles (2007) | + | + | | + | ++ | ++ | + | ++ | + | ++ | NA | NA | ++ | ++ | ++ | + | ++ | ++ | ++ | ++ | NA | NA | NA |
| Xu (2010) | + | + | | - | - | - | - | - | NR | + | NA | NA | - | - | - | + | + | NR | + | NA | NA | NA | NA |
| Yaffe (2001) | + | - | | - | + | + | - | + | ++ | + | NA | NA | - | - | - | + | NR | NR | + | NA | NA | NA | NA |
| Yu (2003) | - | - | | + | - | + | - | ++ | ++ | + | NA | NA | - | - | ++ | - | NR | NR | ++ | NA | NA | NA | NA |

Key to headings – Section 3.5 follow-up time meaningful; 4: Analyses; 4.1 powered to; 4.2 multiple explanatory variables; 4.3 analytical methods; 4.4 precision. Section 5: summary; 5.1 internally validity; 5.2 externally validity. NA: not applicable; NR: not reported.

|  |  | | **Analyses** | | | | | | | **Summary** | | |  |
| --- | --- | --- | --- | --- | --- | --- | --- | --- | --- | --- | --- | --- | --- |
| **Author (Year)** | **3.5** | **3.5b** | **4.1** | **4.1b** | **4.2** | **4.3** | **4.4** | **4.4b** | **4.4c** | **5.1** | **5.1b** | **5.2** | **Ranking** |
| Agahi (2013) | NA | - | NR | NR | + | - | ++ | + | ++ | - | + | + | - |
| Agrigoroarei (2011) | NA | NA | NR | NR | + | + | ++ | ++ | ++ | + | + | + | + |
| Akbaraly (2013) | NA | ++ | NR | NR | + | - | ++ | ++ | ++ | - | + | + | + |
| Alonso (2009) | NA | NR | NR | NR | + | ++ | ++ | ++ | ++ | ++ | + | + | + |
| Andel (2008) | ++ | + | - | + | + | + | + | + | + | + | + | + | + |
| Anttila (2004) | ++ | + | NR | NR | - | + | ++ | - | + | + | + | - | + |
| Ascherio (2001) | ++ | ++ | NR | NR | ++ | + | ++ | + | ++ | + | + | + | ++ |
| Baba (2006) | NA | ++ | NR | NR | ++ | + | ++ | + | ++ | + | + | + | + |
| Beulens (2007) | ++ | NR | NR | NR | ++ | ++ | ++ | ++ | ++ | ++ | ++ | - | ++ |
| Beulens (2008) | ++ | NR | NR | NR | ++ | ++ | ++ | ++ | ++ | + | ++ | - | + |
| Bielak (2012) | + | + | - | + | + | + | ++ | ++ | + | + | + | + | + |
| Blanco-Cedres (2002) | NA | NR | NR | NR | - | + | ++ | ++ | ++ | - | ++ | + | + |
| Boudik (2004) | ++ | NR | NR | NR | - | - | ++ | ++ | ++ | - | + | - | - |
| Britton (2008) | ++ | NA | NR | NR | - | - | ++ | ++ | ++ | - | + | - | + |
| Carlson (2008) | NA | NA | NR | NR | ++ | ++ | ++ | ++ | ++ | ++ | ++ | + | ++ |
| Chang (2010) | ++ | + | NR | NR | - | - | ++ | ++ | NR | - | + | - | - |
| Chang (2013) | NA | NA | NR | NR | - | - | ++ | ++ | ++ | - | + | - | - |
| Christensen (2006) | ++ | NA | NR | NR | - | - | ++ | - | + | - | - | - | - |
| Debette (2011) | ++ | ++ | - | + | + | - | ++ | ++ | ++ | + | - | + | + |
| Dudas (2007) | ++ | ++ | - | ++ | + | - | ++ | ++ | ++ | - | + | ++ | ++ |
| Ekelund (2005) | + | + | - | + | + | ++ | + | + | + | + | + | + | + |
| Elwood (2013) | ++ | ++ | NR | NR | ++ | ++ | + | ++ | + | + | ++ | ++ | ++ |
| Emberson (2005) | ++ | ++ | - | ++ | + | - | ++ | ++ | + | + | + | ++ | ++ |
| Englund (2011) | + | ++ | - | - | + | + | + | + | - | - | - | + | - |
| Englund (2013) | + | + | - | - | + | + | + | + | - | - | - | + | + |
| Eskelinen (2008) | ++ | ++ | - | ++ | ++ | + | + | + | ++ | - | - | + | + |
| Eskelinen (2009) | NA | NA | NR | NR | + | + | ++ | ++ | ++ | + | + | - | + |
| Field (2009) | ++ | + | - | + | + | ++ | + | + | + | + | + | + | + |
| Fogelholm (2000) | NA | + | NR | NR | + | + | ++ | + | + | + | + | + | + |
| Friedland (2001) | - | + | - | + | - | - | + | + | + | + | + | + | + |
| Gerber (2012) | ++ | ++ | NR | NR | ++ | ++ | ++ | + | NR | ++ | ++ | ++ | ++ |
| Guallar-Castillon (2012) | + | + | - | ++ | + | + | + | ++ | + | + | + | ++ | + |
| Haaponen-Niemi (2000) | + | + | - | ++ | + | + | ++ | ++ | + | + | + | + | + |
| Halperin (2008) | ++ | ++ | - | + | + | + | ++ | ++ | ++ | ++ | + | + | + |
| Hamer (2013) | ++ | + | - | + | + | + | + | + | + | ++ | + | + | ++ |
| Happonen (2004) | + | + | - | + | + | + | + | + | + | + | + | + | + |
| Hara (2002) | ++ | + | - | + | + | + | + | + | + | - | + | + | + |
| Harmsen (2006) | ++ | ++ | - | + | + | + | + | - | + | + | + | + | + |
| He (2004) | NA | NR | NR | NR | ++ | + | ++ | ++ | ++ | + | + | + | + |
| Hodge (2013) | + | + | - | + | + | - | ++ | ++ | + | + | + | + |  |
| Holmberg (2006) | + | NR | NR | NR | ++ | + | ++ | + | ++ | + | + | + | + |
| Holme (2007) | ++ | NR | NR | NR | + | - | ++ | ++ | ++ | - | + | + | + |
| Holtermann (2009) | ++ | ++ | NR | NR | ++ | + | ++ | ++ | ++ | + | + | - | + |
| Holtzman (2004) | ++ | ++ | NR | NR | ++ | ++ | ++ | + | ++ | ++ | ++ | - | ++ |
| Hu (2003) | + | + | - | + | + | + | + | + | + | + | + | + | + |
| Hu (2004) | + | + | - | + | + | + | + | ++ | + | + | + | + | + |
| Hu (2005) | + | + | - | + | + | + | ++ | ++ | + | + | + | + | + |
| Hu (2007) Mov Disord | + | + | - | ++ | + | + | + | ++ | + | + | + | + | + |
| Hughes (2010) | ++ | + | - | + | ++ | + | ++ | ++ | + | + | + | + | + |
| Humphries (2001) | + | + | - | + | + | + | ++ | - | + | + | + | + | + |
| Inoue (2004) | ++ | ++ | - | ++ | + | ++ | + | ++ | ++ | + | + | + | + |
| Iso (2004) | ++ | ++ | - | ++ | + | ++ | + | ++ | ++ | + | + | + | + |
| Jakobsen (2009) | ++ | ++ | - | ++ | ++ | ++ | ++ | + | ++ | + | + | + |  |
| Janzon (2004) | + | + | - | + | + | + | + | + | + | + | + | + | + |
| Johnsen (2006) | + | + | - | + | ++ | - | + | + | + | + | + | + | - |
| Kareholt (2011) | ++ | + | - | + | + | + | + | - | + | + | + | + | + |
| Kato (2009) | + | + | - | + | + | + | + | + | + | + | + | + | + |
| Kesse-Guyot (2012) | + | + | - | + | + | + | + | + | + | + | + | + | - |
| Khalili (2002) | ++ | + | - | + | + | + | ++ | ++ | + | + | + | + | + |
| Kimm (2011) | ++ | + | - | + | + | + | + | + | + | + | + | + | + |
| King (2007) | + | + | - | + | + | - | + | ++ | + | + | + | + | + |
| Knopman (2001) | + | + | NR | NR | + | + | ++ | + | + | + | + | + | + |
| Lahti (2010) | + | - | - | + | - | - | + | + | + | + | + | + | + |
| Laitala (2009) | ++ | + | NR | NR | ++ | + | ++ | + | + | + | + | + | ++ |
| Laitinen (2006) | NA | NA | NR | NR | ++ | + | ++ | ++ | ++ | + | ++ | + | ++ |
| Lajous (2013) | ++ | ++ | NR | NR | ++ | ++ | ++ | ++ | ++ | ++ | ++ | ++ | ++ |
| Lang (2007) | NA | NA | NR | NR | ++ | + | ++ | ++ | ++ | + | + | + | + |
| Langlois (2001) | ++ | ++ | NR | NR | + | ++ | ++ | + | ++ | + | + | + | + |
| Laurin (2004) | ++ | ++ | NR | NR | ++ | + | ++ | ++ | + | + | + | + | + |
| Lehto (2013) | ++ | ++ | - | + | + | + | + | + | + | + | + | + | + |
| Lesodottir (2007) | + | + | + | - | + | + | ++ | + | - | + | - | + | + |
| Levitan (2007) | + | + | - | ++ | + | + | + | + | + | + | + | + | + |
| Levitan (2009) | + | + | - | ++ | ++ | + | + | + | + | + | + | + | + |
| Levitan (2010) | + | + | - | ++ | ++ | + | + | + | + | + | + | + | + |
| Lim (2013) | + | + | NR | NR | + | ++ | ++ | ++ | ++ | + | + | + | - |
| Lin (2003) | + | + | - | ++ | + | + | ++ | ++ | ++ | + | + | + | ++ |
| Liu (2003) | NA | NR | NR | NR | ++ | + | ++ | ++ | ++ | + | + | + | + |
| Liu (2004) | ++ | + | NR | NR | + | + | ++ | + | ++ | + | + | + | + |
| Malmberg (2006) | ++ | ++ | - | + | + | + | + | + | + | + | + | + | + |
| Mannami (2004) | + | + | - | ++ | + | + | + | + | + | + | + | + | + |
| Masaki (2003) | + | + | - | + | + | + | + | + | + | + | + | + | + |
| Meisinger (2007) | ++ | + | - | - | - | - | + | + | + | + | + | + | + |
| Menotti (2000) | ++ | ++ | - | + | + | + | + | + | + | + | + | + | + |
| Menotti (2006) | ++ | ++ | - | + | ++ | + | + | + | + | + | + | + | + |
| Menotti (2012) | NA | NA | NR | NR | ++ | + | ++ | ++ | + | + | + | - | - |
| Meyer (2011) | NA | NA | NR | NR | ++ | + | ++ | ++ | + | + | + | - | - |
| Miura (2004) | ++ | + | NR | NR | + | + | ++ | ++ | + | + | + | + | + |
| Moayyeri (2009) | + | + | - | + | + | + | ++ | + | + | + | + | + | + |
| Morgan (2012) | ++ | ++ | - | + | + | + | + | + | + | + | + | + | + |
| Mursu (2008) | + | + | - | + | + | + | + | + | + | + | + | + | + |
| Nafziger (2007) | ++ | ++ | - | + | - | - | + | + | + | - | - | + | - |
| Nakamura (2009) | + | + | - | + | + | + | + | + | + | + | + | + | + |
| Nakayama (2000) | ++ | ++ | - | + | + | - | + | + | + | + | - | - | + |
| Noborisaka (2013) | ++ | ++ | - | + | + | + | ++ | ++ | + | + | + | + | + |
| Nokes (2012) | + | + | ++ | ++ | + | ++ | ++ | + | + | ++ | ++ | + | ++ |
| Nooyens (2008) | + | + | - | + | + | + | + | + | + | + | + | + | + |
| Nooyens (2011) | + | + | - | + | + | + | + | + | + | + | + | + | + |
| Osler (2003) | + | + | NR | NR | + | + | ++ | ++ | ++ | + | + | + | + |
| Ostbye (2002) | ++ | + | NR | NR | + | + | ++ | ++ | ++ | + | + | + | - |
| Ostenson (2012) | ++ | ++ | - | + | + | + | + | ++ | + | + | + | + | + |
| Otani (2003) | + | ++ | - | + | + | + | ++ | ++ | + | + | + | + | + |
| Patel (2006) | + | + | - | + | ++ | + | + | + | + | + | + | + | + |
| Patja (2005) | ++ | + | NA | NA | ++ | NA | ++ | + | + | + | + | + | + |
| Pelkonen (2000) | ++ | ++ | - | + | - | + | ++ | ++ | + | + | + | + | ++ |
| Pitsavos (2004) | ++ | ++ | - | ++ | ++ | + | ++ | ++ | + | + | + | + | ++ |
| Preis (2010) | ++ | ++ | - | + | ++ | ++ | ++ | ++ | ++ | + | + | + | ++ |
| Qiao (2000) | ++ | ++ | - | + | - | + | + | + | + | + | + | + | + |
| Qiu (2003) | + | ++ | - | + | + | + | ++ | ++ | + | + | + | + | + |
| Raikkonen (2001) | + | + | - | + | + | + | + | + | + | + | + | + | + |
| Rantakomi (2009) | + | + | - | + | + | - | + | + | + | + | + | + | + |
| Ravona-Springer (2013) | ++ | ++ | - | + | + | - | + | + | + | + | + | + | + |
| Riserus (2007) | ++ | - | NR | NR | + | + | ++ | + | + | + | + | + | + |
| Ross (2000) | ++ | + | - | + | - | + | + | + | + | + | + | + | + |
| Rovio (2005) | ++ | + | - | + | + | + | + | + | + | + | + | + | + |
| Rovio (2007) | ++ | + | - | + | + | + | + | + | + | + | + | + | + |
| Ruder (2011) | NA | NA | NR | NR | ++ | ++ | ++ | ++ | ++ | ++ | ++ | ++ | ++ |
| Rusanen (2011) | + | + | - | + | + | - | + | + | + | + | + | + | + |
| Ruusunen (2010) | ++ | + | - | + | + | + | + | + | + | + | + | + | + |
| Sabia (2008) | ++ | + | NR | NR | + | + | ++ | ++ | ++ | + | + | + | + |
| Sabia (2009) | ++ | - | NR | NR | + | + | ++ | + | ++ | + | + | + | + |
| Sabia (2011) | + | + | NR | NR | + | + | ++ | - | + | + | + | ++ | + |
| Sairenchi (2004) | + | - | NR | NR | + | + | ++ | ++ | ++ | + | + | + | + |
| Samieri (2013) | ++ | ++ | - | ++ | + | - | ++ | ++ | ++ | + | + | + | ++ |
| Satoh (2006) | + | + | - | + | + | - | + | ++ | + | + | + | + | + |
| Seccareccia (2003) | ++ | + | - | ++ | + | - | + | + | + | + | + | + | + |
| Shaper (2003) | ++ | + | - | ++ | + | + | + | + | + | + | + | + | ++ |
| Sobue (2002) | + | + | - | ++ | - | - | + | + | + | + | + | + | ++ |
| Song (2004) | + | + | - | ++ | - | - | ++ | ++ | ++ | - | - | + | ++ |
| Song (2006) | + | + | - | ++ | + | + | ++ | ++ | ++ | + | + | + | ++ |
| Stevens (2009) | + | + | - | ++ | - | - | + | ++ | ++ | - | + | ++ | ++ |
| Strand (2013) | ++ | ++ | - | ++ | + | + | ++ | ++ | + | + | + | + | ++ |
| Strandberg (2008) | ++ | ++ | - | + | - | + | + | + | + | + | + | + | + |
| Strandhagen (2000) | ++ | + | - | ++ | - | - | ++ | ++ | + | + | + | + |  |
| Sun (2010) | + | + | - | + | ++ | + | + | + | + | + | + | + | ++ |
| Sun (2011) | ++ | + | NR | NR | ++ | + | ++ | ++ | ++ | + | + | + | ++ |
| Szoeke (2006) | + | + | - | + | + | + | + | + | + | + | + | + | + |
| Tabak (2001) | ++ | + | NR | NR | + | + | ++ | - | + | + | + | + | - |
| Tsugane (2004) | + | - | - | ++ | - | + | ++ | ++ | ++ | + | + | + | ++ |
| Tuomilehto (2004) | + | ++ | - | + | - | + | ++ | ++ | + | + | + | + | + |
| Tyas (2003) | ++ | + | - | + | + | + | + | ++ | + | + | + | + | + |
| Valtonen (2010) | ++ | ++ | NR | + | + | + | ++ | + | + | + | + | + | + |
| Villegas (2010) | + | ++ | - | ++ | + | + | ++ | ++ | ++ | ++ | + | ++ | ++ |
| Villegas (2011) | + | ++ | - | ++ | + | + | ++ | ++ | ++ | + | + | ++ | ++ |
| Virta (2010) | ++ | ++ | NR | NR | ++ | ++ | ++ | + | NR | ++ | ++ | + | ++ |
| Waki (2005) | NA | NA | NR | NR | ++ | + | ++ | ++ | + | - | + | + | + |
| Walda (2002) | ++ | + | - | + | + | - | ++ | ++ | ++ | + | + | + | + |
| Wang (2008) | NA | NA | NR | NR | ++ | + | ++ | ++ | ++ | + | + | + | + |
| Wang (2009) | + | + | NR | NR | + | + | ++ | ++ | ++ | ++ | + | + | + |
| Wang (2012) | + | + | NR | NR | + | + | ++ | ++ | ++ | ++ | + | + | + |
| Wannamethee (2001a) Br.J. Canc | ++ | ++ | + | + | + | + | ++ | + | + | + | + | + | + |
| Wannamethee (2001b) Diab. Care | ++ | ++ | + | + | + | + | ++ | + | + | + | + | + | + |
| Wannamethee (2002) | ++ | ++ | - | ++ | + | ++ | + | + | ++ | ++ | + | + | + |
| Wannamethee (2003) | NA | NA | NR | NR | ++ | - | ++ | ++ | ++ | - | + | - | + |
| Waring (2010) | ++ | ++ | - | + | + | + | ++ | ++ | + | + | + | + | ++ |
| Whitmer (2005) | + | + | - | + | + | + | + | + | + | + | + | + | + |
| Willcox (2006) | ++ | ++ | NR | NR | ++ | ++ | ++ | + | + | NA | ++ | + | ++ |
| Wiles (2007) | + | + | NR | NR | ++ | ++ | ++ | + | + | ++ | ++ | ++ | ++ |
| Xu (2010) | ++ | - | NR | NR | + | - | ++ | NR | + | - | + | - | - |
| Yaffe (2001) | NA | NA | NR | NR | + | + | ++ | ++ | ++ | - | + | - | + |
| Yu (2003) | NA | NA | NR | NR | + | - | ++ | ++ | + | - | + | - | + |
