# Supplementary material for: Behavioural Risk Factors in Mid-Life Associated with Successful Ageing, Disability, Dementia and Frailty in Later Life: A Rapid Systematic Review
Source: PLoS One. 2016 Feb 4;11(2):e0144405. doi: 10.1371/journal.pone.0144405 (PMC4742275; doi:10.1371/journal.pone.0144405)
Supplement: S3 Table — (DOCX) [file pone.0144405.s003.docx]

Overview of Included Studies

| **Guide**   1. Data is from multivariate models. Where multiple models have been reported data from the most adjusted (or most relevant) model has been used. 2. **Results**: + = significant positive association, – = significant inverse association, 0 = no significant association; √ = study that shows improved outcomes; X = study that shows poorer outcomes 3. **Quality of study [++/+/-].** ++ = high quality; + = moderate quality; - = low quality | | | | | | | |
| --- | --- | --- | --- | --- | --- | --- | --- |
| **Study** | **Country** | **n** | **Age at baseline** | **Length of**  **follow-up** | **Results association**  **(-/+/0)** | | **Quality** |
| ***Physical Activity (PA)***  Note: A positive association (+) with PA is the better outcome | | | | | | | |
| Andel 2008  (Case control study) | Sweden | 264 dementia  2870 controls (90 twin pairs) | Mean 48.1 (SD 4.9) | 31.4 years | Dementia  Hardly any: 1  Light:  **–**  Regular: −  Heavy: 0  p for Trend .178 | Alz Dis  Hardly any: 1  Light:  **–**  Regular: −  Heavy: 0  p for Trend .339 | + |
|  |  |  |  |  |  |  |  |
| Britton 2008 | UK (England) | 5823 | 35-55 | 17 years | Successful aging  Men: +  Women: + | Odds higher in men and women with higher levels of PA [OR=1.9, (1.2, 3.1); OR=1.7 (1.1, 2.6), respectively] | + |
| Carlson 2008 | US | 147 twin pairs | 45 (SD 3) | 20-40 years | Dementia  Dementia risk: 0 | Risk for dementia by PA scores in discordant twin-pairs: OR=0.99 (0.73-1.33). | ++ |
| Chang 2010 | Iceland | 4761 | 51 | 26 years | Better cognitive function  None 1.00  </= 5h/wk: +  >5h/wk: + | Dementia  None: 1  </= 5h/wk: **─**  >5h/wk: 0  The odds of dementia were lower among those who exercised <=5 hours/week compared to those who never exercised (OR=0.59, [0.40, 0.88]) | - |
|  |  |  |  |  | Those who exercised <=5 hours/week and >5 hours/week at midlife had significantly faster speed of processing (p<0.0001), better memory (p<0.0001), and higher executive function (p<0.0001) compared to those who never exercised at midlife |  |  |
| Chang 2013  (Same study as Chang 2010, different outcomes) | Iceland | 4753 | 51 (SD 7) | 25 years | Better lower extremity function (LEF)  Those who were active in mid-life had better LEF  Inactive: 1  Active: **+** |  | - |
| Debette 2011 | USA | 1352 | 61±9 | 10yrs | White matter, total brain, and LV temporal horn volume  White matter vol.: 0  Total brain vol.: -  Temp. horn vol +  Estimate± SE p. White matter hyperintensity volume -0.03±0.07 p.0.694 Total brain volume -0.15±0.07 p.0.025 Temporal horn volume -0.19±0.07 p.0.008 | Verbal memory, visuospatial memory and exec. function  Verbal memory: 0  Visual memory: 0  Exec function: 0  Estimate± SE p. Logical Memory, delayed recall -0.08±0.08 p.0.316 Delayed recall component of the Visual Reproductions test -0.14±0.08 p.0.070 Trail-Making Test -0.04± 0.08 p.0.563 | + |
| Ekelund 2005 | UK (England) | 605 | 53 (mean) | 5.6 years | PA energy expenditure  Metabolic syndrome summary score: **–** | Baseline PA energy expenditure significantly predicted fasting insulin at follow-up after adjustment for baseline age, BMI, WHR, sex, smoking, SES, fasting insulin, baseline percentage body fat, aerobic fitness, and duration of follow-up (standardized β 0.0012, P 0.006). | + |
| Elwood 2013  (Caerphilly cohort study) | UK (Wales) | 2235 | 45-59 | 30 years | Death: ─  Cog impairment: **─**  Dementia: ─  The odds of cognitive impairment were lower for those who regularly exercised (OR=0.62, [0.41, 0.92]); similar findings reported for dementia. | Vascular disease**:** 0  Diabetes: ─  Cancer: 0  The odds of diabetes were lower among those who regularly exercised (OR=0.63, [0.46, 0.85]). | ++ |
| Englund 2011  (Case-control study) | Sweden | 81 cases/ 156 controls | 57 (SD 5) | 11 years | Walking:  Low: 1  Mod: **─**  High: 0  Never 1 1 time/week 0.14 (0.04–0.53) ≥2 times/week 0.33 (0.10–1.01) | Spare time activity:  Low: 1  Mod: **─**  High: ─  Low 1 Moderate 0.19 (0.08–0.46) High 0.17 (0.05–0.64) | - |
| Englund 2013  (Case-control study) | Sweden | 376 cases/402 controls | 54 (SD 6) | 11 years | Commuting activity:  Low: 1  Mod: 0  High: ─  Low 1.00 Moderate 0.98 (0.60–1.59) High 0.48 (0.27–0.88)  Occupational activity:  Low: 1  Mod: 0  High: 0  Low 1.00 Moderate 0.80 (0.53–1.19) High 0.96 (0.39–2.38) | Training activity: 0  Training activity 1.14 (0.75–1.74)  Cycling:  Low: 1  Mod: 0  High: 0  Low 1.00 Moderate 1.01 (0.54–1.92) High 1.13 (0.70–1.82) | - |
| Friedland 2001  (Case-control study) | US | 193 cases/358 controls  (for total study, not reported for 40-59 year olds) | 40-59 | >12  (not fully reported) | Phys intensity**:** 0  Physical diversity F(1, 544)= 0.96 |  | - |
| Harmsen 2006 | Sweden | 6193 | 47-55 | 28 years | Risk of stroke:  Low leisure PA: 0  Low PA 1.11 (0.90–1.36) |  | + |
| Hamer 2013 | UK (England) | 3454 | 63.7 (SD 8.9) | 8 years | Healthy ageing  **Baseline PA:**  Inactive: 1  Moderate: +  Vigorous: +  P-trend <0.001 | **Change in PA**  Remained inactive: 1.00  Became active: +  Remained active: +  P-trend <0.001 | ++ |
| Holtermann 2009 | Denmark | 4952 | 40-59 | 30 years | All cause mortality  Low Leisure PA : 1  Mod Leisure PA: **–**  High Leisure PA : **–**  Risk significantly lower for those with a moderate and high level of PA during leisure time (HR=0.82, [0.71, 0.94]; and HR=0.64, [0.50, 0.81], respectively) compared to those with a low level of PA during leisure and lower for those with moderate and high (combined) PA during leisure time (HR=0.80, [0.70, 0.92]) compared to those with low levels of PA reported | IHD mortality  Low Leisure PA: 1  High Leisure PA: **–**  The risk of IHD mortality was significantly lower for those with a high level of PA during leisure time (HR=0.37, [0.19, 0.70]) compared to those with a low level of PA | + |
| Holme 2007  (Oslo study) | Norway | 6382 | 40-49 | 28 years | Metabolic syndrome  sedentary/light PA: ref:  moderate:  **–**  mod vig:  **–**  vigorous:  **–**  Odds of metabolic syndrome were lower for those who reported vigorous, moderately vigorous, or moderate levels of LTPA in comparison with those who reported sedentary/light levels (OR=0.46, [0.28, 0.74]; OR=0.65, [0.54, 0.80]; OR=0.83, [0.71, 0.98]) | Diabetes  sedentary/light PA: ref:  moderate:  **–**  mod vig:  **–**  vigorous:  **–**  Odds of diabetes were lower for those who reported vigorous, moderately vigorous, or moderate levels of LTPA in comparison with those who reported sedentary/light levels (OR=0.28, [0.11, 0.71]; OR=0.68, [0.52, 0.91]; OR=0.75, [0.60, 0.94]) | - |
| Hu 2003 | Finland | 13290 | 35-64 | 12 years | Type 2 Diabetes  **Occupational physical activity**  Men 0 Women 0 *Men and women combined*  Light 1.00 Moderate: **–**  Active: **–**  p value for trend 0.020  **Commuting physical activity** Men 0 Women 0 *Men and women combined*  ≥30 min **–**  p value for trend 0.048 | **Leisure-time physical activity**  Men: 0  Women: 0 *Men and women combined*  Low 1.00 Moderate: 0  High: 0  p value for trend 0.186 | + |
| Hu 2004 | Finland | 18892 | 25-74 (mean age 42-48) | 9.8 years | Risk of CVD **Men** Low PA: 1.00 Moderate PA: **–**  High PA: **–** P trend 0.007 | Risk of CVD  **Women** Low PA: 1.00 Moderate PA: **–**  High PA: **–** P trend 0.02 | + |
| Hu 2005 | Finland | 47212 | 25-64 (mean age 41-46) | 17.7 years | **Men** Total mortality Low 1.00 Moderate: **–**  High: **–**  P-value for trend <0.001  **Women** Total mortality Low 1.00 Moderate: **–**  High: **–** P-value for trend <0.001 | **Men** Cardiovascular mortality Low 1.00 Moderate **–**  High **–**  P-value for trend <0.001  **Women** Cardiovascular mortality Low 1.00 Moderate: **–**  High: **–** P-value for trend <0.001  Cancer mortality Low 1.00 Moderate: **–**  High: **–**  P-value for trend 0.005 | + |
| Hu 2007 | Finland |  | 25-64  (mean age 42-49) | 18.9 years | CHD events **Men** Occupational PA  Low: 1 Moderate: **–**  High: **–**  P trend 0.007 | **Women** Physical activity Low: 1 Moderate: **–**  High: **–** P trend 0.02 | + |
| Knopman 2001 | USA | 10,963 | 47-70 | 6yrs | Cognitive change  Never: 0  Former: 0  Current: 0 |  | + |
| Lahti 2010 | Finland | 5437 women, 1257 men | 49-51 | 5-7 years | Better physical function  **Women:**  Inactive vs Conditioning PA: **+**  Inactive vs active mod, active vig, very active mod, very active vig PA: 0  Lowest percentage of poor physical health functioning at follow-up was reported for the active vigorous group (22%, [95% CI: 19%, 25]); highest percentage of good physical health functioning at follow-up was reported for the very active moderate group (27%, [24%, 30%]) | **Men:**  Inactive vs active mod, active vig, very active mod, very active vig, conditioning PA: 0  Lowest percentage of poor physical health functioning was reported for the active moderate and active vigorous group (22%, [17%, 27%], and 22%, [16%, 28%], respectively; highest percentage of good physical health functioning at follow-up was reported for the conditioning group (29%, [24%, 35%]) | + |
| Lang 2007 | UK [England (ELSA study), and US] | 8702 (US)& 1507 (UK) (from 2 studies | 50-69  (mean 60.2 & 58) | 6 years | Incidence of impaired physical mobility  UK study (ELSA): **–**  US study:  **–** |  | + |
| Malmberg 2006 | Finland | 1791 | 40-64 | 16 years | Difficulty walking  **Men:**  Fitness activity: P= .029  >3 times /wk: 1  2 times/wk: +  Once/wk: +  <once/wk: +  None: +  Global LTPA: 0  p = .023  LTPA energy ex 0  p = .531  LTPA freq – int 0  p = .393  Commuting 0  p = .532 | **Women:**  Fitness activity: 0  p = .690  Global LTPA: 0  p = 0.34  LTPA energy ex 0  p =.935  LTPA freq – int 0  Commuting 0  p = .873  Difficulty In Walking  p = .371  Commuting P= .363 | + |
| Meisinger 2007 | Germany | 3501 men, 3475 women | 45-74 | 8.6 years | Myocardial infarction  **Men:**  None: 1  Low 0  Med: 0  High: 0  None1.0  Low 1.01 (0.73–1.40)  Moderate 0.78 (0.56–1.10)  High 0.84 (0.59–1.18) | **Women:**  None: 1  Low 0  Med: –  High: –  None1.0  Low 1.00 (0.56–1.78)  Moderate 0.49 (0.24–1.00)  High 0.21 (0.05–0.87) | + |
| Menotti 2006 | Italy | 1712 | 40-49 | 5 years | All cause mortality: **–** |  | + |
| Morgan 2012  (Caerphilly cohort study) | UK (Wales) | 1005 | 45-59 | 16 years | Dementia  Leisure time PA: 0  Occupational PA: 0 |  | + |
| Nokes 2012 | US | 244 | 35-45 | 6 years | Gain in bone mineral density:  Low PA volume: 1  Moderate: +  Moderate-high: +  PA intensity: 0 | Women with moderate to high PA volume levels were more likely to have hip BMD gains compared to women in the low PA volume group (RR=2.01, [1.05, 3.81]); women with moderate PA volume levels were more likely to gain BMD at the hip than women with low PA volume (RR=1.97, [1.02, 3.79]) | + |
| Ostbye 2002 | US | 7845 (HRS study)  5037 | 51-61 | 5-6 yrs | Disability  Light: **–**  Mod:  **–**  Heavy:  **–**  ADL & IADL  Light: **–**  Mod:  **–**  Heavy:  **–**  Stairs/Blocks  Light: **–**  Mod:  **–**  Heavy:  **–** | Poor health  Light: **–**  Mod:  **–**  Heavy:  **–**  Hospitalised  Light: **–**  Mod:  **–**  Heavy:  **–**  Compared to those with BMI 18.5-30, those with BMI 30 or greater generally had the highest odds for ill health in terms of ADL dependence (OR=1.66, [1.45, 1.89]), disability (OR=1.48, [1.28, 1.72]), difficulty climbing stairs (OR=2.37, [2.16, 2.60]), difficulty walking (OR=2.10, [1.89, 2.34]), poor health (OR=1.70, [1.53, 1.88]), hospitalization (OR=1.38, [1.26, 1.51]); further, the odds for ill health were generally greater for people with BMI less than 18.5 | - |
| Patel 2006 | Italy | 1001 | 40-60 | 7 years | Unable to walk 400 meters:  **Men** Low Reference Moderate 0 Vigorous –  p for trend p = <0.001  **Women** Low Reference Moderate 0 Vigorous 0  p for trend p = 0.620 | Better physical performance **Men b weight (SE)** Low 1 Moderate 0 Vigorous + p for trend p = 0.023  **Women b weight (SE)** Low 1 Moderate 0 Vigorous + p for trend p = 0.024 | + |
| Pitsavos 2004 | Greece (Corfu) | 529 | 49 ±6 | 40 years | Presence of LVH  Sedentary: 1  Moderate –  Hard 0  Sedentary (ref. group) 1.00 —  Moderate 0.70 (0.50–0.97)  Hard 0.75 (0.52–1.09) | Absence of LVH Sedentary) 1 Moderate –  Hard 0  Sedentary (ref. group) 1.00 —  Moderate 0.64 (0.45–0.91)  Hard 0.72 (0.51–1.02) | + |
| Riserus 2007 | Sweden | 770 | 50 | 20 yrs (70-73 to 91-95) | Insulin sensitivity  Leisure PA:  **–**  beta=0.25, [0.08, 0.42] |  | + |
| Rovio 2005 | Sweden | 2000 | 50 | 21 years | Dementia  Sedentary: 1  PA at least 2/wk: **–**  Dementia 0·47 (0·25–0·90) | Alzheimer’s disease  Sedentary: 1  PA at least 2/wk: **–**  Alzheimer’s disease 0·35 (0·16–0·80) | + |
| Rovio 2007  (Same study as Rovio 2005) | Sweden | 1449 | 50 | 21 years | Dementia  Sedentary 1  PA at least 2/wk **0**  Dementia 1.45 (0.66–3.17) | Alzheimer’s disease  Sedentary 1  PA at least 2/wk 0  Alzheimer’s 1.90 (0.73–4.95) | + |
| Sabia 2009 | England | 5123 | 44 (mean) | 17 years  (85-88 to 02-04) | Short-term association (5y)  Low PA: 1  High PA: + | Long-term association (17y)  Low PA: 1  High PA: 0  The odds for poor executive function were higher among those with high levels of PA compared to low levels (OR=1.19, [1.01, 1.39]) | + |
| Stevens 2009 | England and Scotland | 1.29 million women | 50-64 (mean 56) | 96-01 to 05-07  Mean yrs of follow-up: 7.2 for cancer incidence; 8.9 for mortality | Mortality  <1 PA per week: 1.00  1 PA per week: 0  2-3 PA per week: 0  >/= 4 PA per week: 0 |  | + |
| Sun 2010 | US | 13535 | 60 (mean age) | 14 years | Successful survival  (Mean)  METS 0.9h/wk 1  3.6 0  7.9 +  16.2 +  37.1 + | Total Physical Activity  P for trend <0.0001  Walking  P for trend 0.0003 | ++ |
| Szoeke 2006 | US | 224 | 50 (mean) | 11 years | Osteoarthritis  PA 0 |  | + |
| Wannamethee 2001 | UK (England) | 7630 | 40-59 | 18.8 years | Total cancer:  No PA 1.00  Occasional PA **–**  Vigorous PA **–** p for trend <0.0001  Upper digestive tract cancer (oral, esophagus, stomach cancer)  No PA 1.00  Moderate-vigorous PA **–** | Bladder cancer  No PA 1.00  Vigorous PA **+**  Prostate cancer  No PA 1.00  Vigorous PA **–**  No sig association found for lung, stomach, colorectal, lymphatic/haematopoetic cancers. Vigorous exercise was associated with a significantly increased risk of bladder cancer. | + |
| Wiles 2007  (Caerphilly cohort study) | UK (Wales) | 2512 | 45-59 | 10 years | *5-year follow-up: Mental disorders  Total leisure PA  Low: 1  Med: 0  High: 0  % time in heavy PA Low: 1  Med: **–**  High: **–**  The odds of a common mental disorder at phase III were lower among those with low and high percentage of leisure time spent in heavy-intensity PA at phase II (OR=0.61, [0.40, 0.93], and OR=0.54, [0.35, 0.83], respectively) | *10-year follow-up: Total leisure PA  Low: 1  Med: 0  High: 0  % time in heavy PA Low: 1  Med: 0  High: 0 | ++ |
| Xu 2010 | South East Queensland, Australia | 564 | 45-60 yrs (mean 55) | 2001-06 | Anxiety  None ref  1-2/wk 0  3-4/wk 0  5-6/wk 0  Depression  None ref  1-2/wk 0  3-4/wk 0  5-6/wk 0  Women who exercise 5-6 times per week scored 1 point lower on the anxiety scale (p=0.013), 1 point lower on the depression scale (p=0.001) compared to women who did not exercise | Psychological symptoms  None ref  1-2/wk 0  3-4/wk 0  5-6/wk 0  SF-36 mental health:  None ref  1-2/wk 0  3-4/wk 0  5-6/wk 0  Women who exercise 5-6 times per week scored 2 points lower on the psychological scale (p=0.002), and 9 points higher on the mental well-being scale (p=0.001) compared to women who did not exercise | **-** |
| Yu 2003  (Caerphilly cohort study) | UK (Wales) | 1975 | 45-59 | 10.5 years | Heavy intensity activity  All-cause death: **–**  The greater the energy expenditure of heavy intensity activity during leisure at baseline, the smaller the risk of all-cause death, CVD death, and CHD death during follow-up (trend p-values: 0.006, 0.001, and 0.009, respectively) | Total activity:  CHD death: **–**  (trend p-value=0.039)  Heavy intensity activity  CVD death: **–**  CHD death: **–**  People expending 23.9-2142.9 kcal/day had a lower risk of all-cause death, CVD death, and CHD death than those expending 0.0-0.6 kcal/day (HR=0.61, [0.43, 0.86]; HR=0.38, [0.21, 0.67]; HR=0.36, [0.18, 0.73], respectively) | + |
| ***Physical Inactivity***  Note: Physical inactivity where multiple outcomes have reported the most adjusted data in this table; sig = p</=0.05; ns = not significant (p>0.05) | | | | | | | |
| Christensen 2006 | Denmark | 376 | 50, 60, 70 | 25 years | Disability at age 75  At age 50: 0  At age 60:  </= 7 y school: 0  > 7 y school: 0 | Physical inactivity at age 60 to 70 was related to disability for the sub-group with more than 7 years of education (OR=8.62, [1.08, 68.54]) | - |
| Haapanen-Niemi 2000 | Finland | 2212 (295 PA) | 35-63 | 16 years | CVD mortality  1) LTPA index:  High: 1.00  Mod: 0  Low: 0  2) Single item LTPA  Vigorous: 1.00  None: + | All cause mortality  1) LTPA index:  High: 1.00  Mod: 0  Low: 0  2) Single item LTPA  Vigorous: 1.00  None: 0 | + |
| ***Diet*** | | | | | | | |
| Akbaraly 2013 | UK | 8815 | 35-55 years | 18 years | Ideal aging  Western-type diet  Top tertile compared to the bottom tertile: **─**  Healthy diet pattern:  Top tertile compared to the bottom tertile: 0  Ideal aging were lower for participants in the top tertile of the Western-type diet compared to the bottom tertile (OR=0.58, [0.36, 0.93]) | CVD and non-CVD deaths  Healthy eating High adherence to the AEHI: **─**  High adherence to the AEHI CVD deaths  OR=0.60, [0.39, 0.92]  non-CVD deaths  OR=0.75, [0.57, 0.98] | + |
| Britton 2008 | UK | 5823 | 35-55 years  (mean: 44) | 20 years | Men  Good diet vs poor diet: +  [OR=1.4, (1.1,1.7)] |  | + |
| Elwood 2013 | UK (Caerphilly) | 2235 | 45-59 years | 30 years | Cog impairment: 0  Dementia: 0  Death: 0  Cancer: 0 |  | + |
| Eskelinen 2008 | Finland | 1449 | SD: 50.2 | 21 years | Cognitive impairment:  Total fat  Low (0-38 g/d): 1  High(>38 g/d) +  Low (0–38.0 g) 1  High (>38.0 g) 1.69 (1.00–2.87) | Sat fat  Low (0-21.6 g/d): 1  High(>21.6 g/d) +  Low (0–21.6 g) 1  High (>21.6 g) 2.36 (1.17–4.74) | + |
| Eskelinen 2009  (CAIDE study) | Finland | 1409 | SD: 50.4 | 21 years | Dementia risk:  Coffee: **─** lower for those consuming moderate amounts of coffee (3-5 cups/day) compared to low amounts (0-2 cups/day). | Tea 0  (all associations)  ***for APOE4 carriers:  Coffee:  **─** | + |
| He 2004 | United States | 74063 | 38-63 years | 12 years | Obesity  Change in fruit and veg (servings/day):  -2.36 1.00 (ref)  -0.49 **─**  +0.64 ─  +1.83 ─  +3.99 ─  P trend <0.0001 | Weight gain >/+25kg:  Change in fruit and veg (servings/day):  -2.36 1.00 (ref)  -0.49 0  +0.64 0  +1.83 ─  +3.99 ─  P trend 0.01 | + |
| Hodge 2013 | Australia | 8660 | 50-69 years | 12 years | Highest vs lowest adherence to Mediterranean Diet (MDS) **─** | Highest vs lowest adherence to traditional Australian Diet **─** | + |
| Hughes 2010 | Sweden | 3779  (3424 non-demented, 355 dementia cases) | Mean age 48 | 30 years | Dementia  No/small fruit & veg intake: 1.00 (ref)  Medium or great fruit & veg intake: **─** |  | + |
| Hu 2007 | Finland | 29335 | 25-64 years | 18.9 years | Risk of Parkinson’s Disease  Volume of coffee consumption (cups/d)  **Men**  0 1  1–4 0  5 or > ─  P-trend 0.063  **Women**  0 1  1–4 0  5 or > **─**  P-trend 0.073  **Men and Women**  0 1  1–4 **─**  5 **─**  P-trend .005 | Volume of tea consumption (cups/d)  **Men**  0 1  1–4 0  5 0  P-trend 0.31  **Women**  0 1  1–4 0  5 **─**  P-trend 0.11  **Men and Women**  0 1  1–4 0  5 **─**  P trend 0.038 | + |
| Kesse-Guyot 2012 | France | 3054 | SD: 52.1 | 13.4 years | Better cognitive performance  High score  ‘healthy pattern’ (Q4 vs Q1) **+**  P trend 0.001  (Executive fn) | High score ‘traditional pattern’  (Q4 vs Q1)  P trend 0.06  (Global cog fn) **─** | - |
| Laitala 2009 | Finland | 2606 | Mean age 46-52 | 28 years | MCI  Coffee (cups/d)  0-3 (ref) 1  3.5-8 0  >8 0 | Dementia  Coffee (cups/d)  0-3 (ref) 1  3.5-8 0  >8 0 | ++ |
| Laitinen 2006 | Finland | 1449 | SD: 50.4 | 21 years | Dementia  Moderate vs Low PUFA: **─**  Moderate vs Low sat fat: **+**  Odds of developing dementia were lower for those consuming moderate amounts of PUFA from spreads compared to those consuming low amounts [OR=0.40, 95% CI: 0.17-0.94; and 2]. Odds of developing dementia and AD were higher for those consuming moderate amounts of saturated fats from spreads in comparison with those consuming low amounts [OR=2.45, (1.10-5.47), and OR=3.82, (1.48-9.87), respectively] | APOE4 carriers  Moderate vs Low PUFA: **─**  Moderate vs Low sat fat: **+**  Among APOE4 carriers, the odds of developing dementia was lower for those with moderate PUFA intake compared to those with low PUFA intake [OR=0.29, 95% CI: 0.09-0.89]; the reverse was observed for SFA | ++ |
| Laurin 2004 | US (Hawaii) | 2459 | 45-68 years  (Mean: 51.2) | 30.2 years | Dementia  β-carotene 0  vitamin C 0  flavonoids 0  vitamin E  Q1 1.00 ref  Q2 **+**  Q3 0  Q4 0  Compared to the first quartile of vit. E intake, the risk for dementia was greater for those in the second quartile of vit. E intake (RR=1.47, [1.01, 2.14]  Alzheimer’s disease  β-carotene 0  vitamin C 0  flavonoids 0  vitamin E  Q1 1.00 ref  Q2 **+**  Q3 0  Q4 0  Compared to the first quartile of vit. E intake, the risk for Alzheimer’s disease was greater for those in the second quartile of vit. E intake [1.04, 3.25] | Alzheimer’s disease w/w’out cerebrovascular disease  β-carotene 0  vitamin C 0  flavonoids 0  vitamin E  Q1 1.00 ref  Q2 +  Q3 0  Q4 +  Vascular dementia: **0**  Compared to the first quartile of vit. E intake, the risk for Alzheimer’s disease with and without cerebrovascular disease was greater for those in the second and fourth quartiles of vit. E intake (RR=1.92, [1.16, 3.18], RR=1.78, [1.06, 2.98], respectively)  Significant association between high intake of antioxidants and risk for AD with and without contributing cerebrovascular disease (RR=1.82, [1.04, 3.21]) | + |
| Lehto 2013 | Finland | 2600 | 42-61 years | 20.1 years | Depression  Energy adjusted zinc intake: 0 |  | + |
| Liu 2003 | United States | 74091 | 38-63 years | 12 years | Obesity (BMI >/=30)  Wholegrains (change in intake)  Q1 (ref) 1.00  Q2 **─**  Q3 ─  Q4 ─  Q5 (high intake) ─  P trend 0.0002  Refined grains (change in intake)  Q1 (ref) 1.00  Q2 0  Q3 0  Q4 0  Q5 (high. intake) 0  Dietary fibre (change in intake)  Q1 (ref) 1.00  Q2 **─**  Q3 ─  Q4 ─  Q5 (highest intake) **─**  P trend <0.0001 | Weight gain >/= 25 kg  Wholegrains (change in intake)  Q1 (ref) 1.00  Q2 0  Q3 0  Q4 0  Q5 (highest intake) 0  Refined grains (change in intake)  Q1 (ref) 1.00  Q2 0  Q3 0  Q4 0  Q5 (highest intake) 0  Dietary fibre (change in intake)  Q1 (ref) 1.00  Q2 **─**  Q3 ─  Q4 ─  Q5 (highest intake) **─**  P trend <0.0001 | + |
| Masaki 2003 | Japan | 5644 | 40-69 | 10 years | Stomach Cancer  Vegetable and fruit pattern Low 1.00  Middle 0  High 0  P trend 0.56  Western breakfast  Low 1.00  Middle 0 High 0  P trend 0.20 | Meat  Low 1.00  Middle 0  High 0 P trend 0.07   Rice/snacks Low 1.00  Middle 0  High 0 P trend 0 .05 | + |
| Miura 2004 | United States | 1710 | 40-55 years  (Mean 48.5) | 39 years | Systolic blood pressure (men) Vegetables (cups/month)  <14 ref 14–42 0 >42 0  Fruits (cups/month)  <14 ref 14–42  >42 0 14–42 ─  >42 0  Fish (120-g units/month)  None (ref) <4 0 4–8 0 >8 0  Beef-veal-lamb (120-g units/month)  8–20 + >20 +  Pork (120-g units/month)  4–8 + >8 0  Poultry (120-g units/month)  4–8 0 >8 0 | Diastolic blood pressure Vegetables (cups/month)  14–42 0  >42 0  Fruits (cups/month)  14–42 0  >42 0  Fish (120-g units/month)  <4 0 4–8 0 >8 0  Beef-veal-lamb (120-g units/month)  8–20 0 >20 +  Pork (120-g units/month)  4–8 0 >8 0  Poultry (120-g units/month)  4–8 + >8 + | + |
| Nakamura 2009 | Japan | 2316 | 47-60 years | 19 years | Impaired ADL  Meat (no. times in 2 days) <1/2 d ref 1.00  ≥1/2 days **─** (less impaired ADL) p = 0.36    Fish  <1/day 1.00  ≥1/day 0  p = 0.50 | Egg  <1/day 1.00 ≥1/day 0  p = 0.43  Mortality  Meat, fish, egg: **0** | + |
| Nooyens 2011  (Doetinchem Cohort Study) | Netherlands | 2613 | 43-70 years | 10 years | Change in cognitive function  Fruit and vegetables: 0  Fruit: 0  Vegetables: 0  Legumes: 0  Juices: 0 |  | + |
| Osler 2003 | Denmark | 7540 | 30-70 years | 36 years | All cause mortality  **Men (times)**  <1/month vs once/wk: **─**  **Men and women**  2/month vs once/wk: **─** | Among males, females and high-risk participants, there was a significant linear trend of increasing risk in all-cause mortality with greater intake of fish (trend test p-values=0.02 and 0.03, respectively) | + |
| Ross 2000 | US  Hawaii | 8004 | 45-68 years | 30 years | Parkinson’s Disease  Coffee (drinkers v non-drinkers): **─**  Caffeine:  **─** |  | + |
| Ruder 2011 | United States | 292797 | 40-61 years | 10 years | Risk of colorectal cancer  Calcium: **─**  Vitamin A: **─**  Vitamin C: **─**  Fruit: **─**  Milk: **─**  (lower risk of colon cancer)  Total fat: +  Red meat +  Processed meat: +  Those in the highest intake category for calcium (HR: 0.83; 95% CI: 0.73, 0.94), vitamin A (HR: 0.81; 95% CI: 0.71, 0.92), vitamin C (HR: 0.83; 95% CI: 0.72, 0.95), fruit (HR: 0.84; 95% CI: 0.73, 0.97), and milk (HR: 0.78; 95% CI: 0.67, 0.90) had a lower risk. A higher risk of colon cancer was observed for total fat (HR: 1.15 ; 95% CI: 1.01, 1.30), red meat (HR: 1.31; 95% CI: 1.12, 1.53), and processed meat (HR: 1.24; 95% CI: 1.06, 1.45) | Rectal cancer  Fibre 0  Milk **─**  For rectal cancer, milk was inversely associated (HR: 0.75; 95% CI: 0.58, 0.96) with risk | ++ |
| Ruusanen 2010 | Finland | 2232 | 42-60 years | 17.5 years | Depression  Coffee (ml/d)  None 1  Light (<375): **─**  Moderate(375-813): 0  Heavy: **─**  P value 0.035 | Tea (ml/d)  None 1  Light (<375): 0  P value 0.252  Caffeine  All categories: 0 | + |
| Sabia 2009 | UK | 5123 | 35-55 years | 17 years | Executive function:  Fruit & veg(servings/d)  >/= 2 vs <2: +  The odds for poor executive function were higher among those who consumed fruits and vegetables >=2 times per day compared to those who consumed less (OR=1.60, [1.36, 1.89]) | Memory 0  The odds for poor memory were higher among those who consumed fruits and vegetables (>=2 times) compared to those who consumed less (OR=1.35, [1.14, 1.59]) | + |
| Samieri 2013 | United States | 10670 | Upper 50s, lower 60s  (SD: 59) | 15.2 years | Healthy ageing  Healthy eating index diet:  Q1 low ref 1.00  Q2 0  Q3 0  Q4 +  Q5 +  P trend<0.001 | Med diet score  Q1 low ref 1.00  Q2 +  Q3 +  Q4 +  Q5 +  P trend =0.002 | + |
| Seccareccia 2003 | Italy | 1536 | 45-65 years | 30 years | Total mortality  Vegetable intake (for each increase of 20g/day): **─**  0.97 (0.94–0.99) |  | + |
| Song 2006 | United States | 28349 | 45+ years | 9.8 years | Hypertension  Highest vs lowest quintile  Magnesium: 0 | Total magnesium intake  P trend 0.03  Dietary magnesium intake  P trend 0.002 | + |
| Strandhagen 2000 | Sweden | 792 | Age 54 | 26 years | Total mortality  Fruit: **─**  Veg: 0  Cancer  Fruit: 0  Veg: 0 | Cancer mortality  Fruit: 0  Veg: 0  RR (95% CI) P  Fruit 0.92 (0.84 - 1.00) 0.051 | ++ |
| Tsugane 2004 | Japan | 39065 | 40-59 years | 11 years | Gastric cancer  Salt intake  **Men**: +  Highest vs lowest  P trend<0.001  **Women**: 0 |  | + |
| Walda 2002 | Finland, Italy and The Netherlands | 2917 | 50-69 years | 20 years | COPD mortality  Fruit: ─  Vit C: 0  Vit E: 0  Β-carotene 0  Veg: 0  Fish: ─ |  | + |
| Wang 2009 | United States | 38408 | 45+ years | 11.5 years | Total cancer  Total or individual flavonoids: 0 | Other cancers  Breast cancer, colorectal cancer, lung cancer, endometrial cancer, ovarian cancer: 0  Stomach, pancreatic, bladder, brain, thyroid, cervical cancer, lymphoma/leukemia): 0 | + |
| Wang 2012 | United States | 28082 | 39+ | 12.9 years | Incident hypertension  Fruit & veg: 0  Fruit: 0  Vegetables: 0 |  | + |
| Wang 2008 | United States | 28766 | 45+ years  (SD 53.8) | 10 years | Hypertension  Low fat dairy  Q1 ref 1.00  Q2 0  Q3 0  Q4 0  Q5 ─  Ptrend 0.001  High fat dairy  Q1 ref 1.00  Q2 0  Q3 0  Q4 0  Q5 0  Ptrend 0.17 | Total dairy  Q1 ref 1.00  Q2 0  Q3 ─  Q4 ─  Q5 ─  Ptrend 0.003 | + |
| Xu 2010 | Australia | 564 | 45-60 years | 5 years | Anxiety  Caffeine v non-caffeine: 0  Depression  Caffeine v non-caffeine: 0 | Psychological symptoms  Caffeine v non-caffeine: 0  SF-36 Mental Health  Caffeine vs non-caffeine: ─  (lower mental health in caffeine drinkers) | - |
| ***Smoking***  Note: A positive association (+) with smoking is a worst outcome | | | | | | | |
| Agahi 2013 | Sweden | 1060 | 30-50 | Up to 34yrs | Mobility impairment:  Non Smoker: 1   - Persist heavy: + - Former heavy: + - Former light: +   Persist non-smoker: 1   - All categories: +   Rate of increase in mobility impairment was steepest among persistent heavy smokers (coeff.=0.01, SE=0.004), and former heavy smokers (coeff.=0.01, SE=0.003) in comparison with non-smokers  Compared to non-smokers, former light smokers had statistically significantly steep progression of mobility problems (coeff.=0.006, SE=0.003) | Musculoskeletal pain: 0  Psychological distress:  Persist non-smoker: 1  Heavy smoker: + | - |
| Alonso 2009 | USA | 11,151 | 45-64 | Up to 10yrs | Incident dementia   - Never smoker: 1 - Current: +   HR=1.7, (1.2, 2.5) |  | + |
| Baba 2006 | Japan | 41,307 | 40-59yrs | 11yrs | Acute coronary events   - Never smoker: 1 - Current: + - Past smoker: + - Men: + - Women: +   Risk of (total) coronary heart disease and (total) myocardial infarction significantly higher in male current smokers compared to those who never smoked (RR=2.85, [1.98, 4.12] and RR=3.64, [2.27, 5.83], respectively) | **Males**: risk of total CHD and total myocardial infarction increased with the number of cigarettes smoked per day (trend test p-values: <0.001 and <0.001, respectively)  **Women**: risk of (total) CHD and (total) myocardial infarction greater for current compared to never smokers (RR=3.07, [1.48, 6.40], RR=2.90, (1.18, 7.18), respectively); the risk of (total) myocardial infarction was also greater for past compared to never smokers (RR=3.72, [1.10, 12.6]) | + |
| Blanco-Cedres 2002 | USA | 8,816 | 40-59yrs | 25yrs | CHD death, CVD death, all-cause mortality (per strata cholesterol levels)   - Non-smoker: 1 - CHD Current: + - CVD Current: + - All cause mort. current: + | RR for CHD death was greater for smokers compared to non-smokers and ranged from 1.50 [1.17, 1.88]) to 2.18 [1.54, 3.08]) across cholesterol levels  RR for CVD death was greater for smokers than non-smokers and ranged from 1.58 [1.17, 2.14]) to 1.95 [1.48, 2.57]) across cholesterol level  RR for all-cause mortality greater for smokers compared to non-smokers and ranged from 1.78 [1.54, 2.07]) to 2.19 [1.84, 2.61]) across cholesterol levels | + |
| Boudik 2006 | Prague | 926 men | Mean 46.1 (Middle aged men) | 21yrs | Atherosclerotic CVD mortality  <15cig/d = 1  >=15cig/d: +  HR=3, [2.0, 4.6] |  | - |
| Britton 2008 | England | 5823  (civil servant) | 35-55 | 17yrs | Successful aging   - Current smoker: 1 - Non-smoker: **+**   Less exposure: + (men & women) | Non-smokers compared to current smokers for men and women [OR= 2.7, (1.8, 4.1), OR=2.2 (1.3, 3.7), respectively] | + |
| Dubas 2007 | Sweden | 7388 | 47-55 | 28yrs  (‘70-‘98) | All AMI  Never smoker: 1  Former: +  1–14 g/day: +  15–24 g/day: +  >25 g/day: +  Smoking (1–5): +  OR age-adjusted 95% CI  Smoking (1–5) 1.51 1.43–1.60 | Coronary bypass  Never smoker: 1  Former: 0  1–14 g/day: 0  15–24 g/day: 0  >25 g/day: +  Smoking (1–5): +  OR age-adjusted 95% CI  Smoking (1–5) 0.78 0.66–0.92 | ++ |
| Englund 2013 | Sweden | 778 | 54±5.9 | 11.2±2.6 | Wrist fracture   - Never smoker: 1 - Former: 0 - Current: 0 | Active commuters were at significantly lower risk of sustaining a wrist fracture (OR 0.48; 95 % CI 0.27–0.88) compared with those who commuted by car or bus, in middle-aged women | + |
| Fogelholm 2000 | Finland | 1143 | 36-88 | 10 yrs (’85-‘95) | Weight change   - Never smoker: 1 - Smoking: + | Being a smoker (beta= -1.59, SE=0.48) was significantly (p<0.05) associated with weight loss | + |
| Gerber 2012 | Israel | 4633 | 50.1±6.5 | Median 26yrs (quartiles 1–3: 16–35) | All cause mortality   - Maintained: 1 - Increased: 0 - Reduced: - - Quit: -   P trend <0.001 | CVD mortality   - Maintained: 1 - Increased: 0 - Reduced: - - Quit: -   P trend 0.01 | ++ |
| Halperin 2008 | USA | 13,529 | 52.4±8.9 | Med 14.5yrs  Max 20.5yrs | Hypertension   - Never smoker: 1 - Past: + - <20cig/day: + - ≥20cig/day: - - Current: + | Changes in smoking status showed increased RR (95% CI) of developing hypertension for current and past smokers of 1.14 (1.00, 1.25) and 1.08 (1.01, 1.15) respectively, and an increased RR of developing hypertension for new smokers at 2 years and new quitters at 2 years of 1.21 (0.96, 1.52) and 1.35 (1.08, 1.68), respectively. | + |
| Hara 2002 | Japan | 41,484 | 40-59 | Never: 64,986 PA  Former: 42,798 PA  Current: 103,537 PA | Circulatory death   - Never smoked: 1 - Former (M & F): 0 - Current (M): - - Current (F): +   RR 2.72 (1.45 – 5.07) | All cause mortality   - Never smoked: 1 - Former (M & F): 0 - Current (M & F): +   RR 1.89 (1.36 – 2.62) | + |
| Harmsen 2006 | Sweden | 7457 | Middle-age men | 28yrs | Stroke   - Non-smoker: 1 - Smoking: +   HR 1.33 (1.15–1.53) |  | + |
| Holme 2007 | Norway | 6382(M) | 40-49 | 28yrs | Metabolic syndrome   - Never smoked: 1 - Current: +   The odds of metabolic syndrome were higher for current smokers compared to never smoked OR=1.29, [1.11, 1.51] | Diabetes   - Never smoked: 1 - Current: 0 | + |
| Holmberg 2006 | Sweden | 22444 (M) 10902(W) | Men: 27-61 yrs  Women: 28-58 yrs | 19yrs (M) 15yrs (W) | Incident low-energy fractures   - Non-smoker: 1   (vs smoker)  **Women:**   - Vert fracture: + - All other types: 0   Female smokers had a higher risk for vertebral fractures (RR=1.96, [1.47, 2.64]) than non-smokers | **Men:**   - Any fract: + - Forearm fract: 0 - Vert fracture: + - Prox humerus: + - Ankle: 0 - Hip: +   Male smokers had a greater risk for low energy fractures (RR=1.25, [1.11, 1.39]), vertebral fractures (RR=1.85, [1.41, 2.42]), proximal humerus fractures (RR=1.58, [1.08, 2.33]), and hip fractures (RR=2.14, [1.51, 3.01]) than non-smokers | + |
| Humphries 2001 | UK | 3052 men | 55.7±3.2 | 11yrs | Coronary hearth disease  Never smoked: 1  Ex-smokers   - E3/E3 : 0 - E2+ : 0 - E4+ : 0 | Smokers   - E3/E3 : 0 - E2+ : 0 - E4+ : ++   E4+ 2.79 (1.59–4.91) | + |
| Inoue 2004 | Japan | 92,792 | 40-69 (mean 53) | 10 yrs | Cancer  Never smoker: 1  (M & W)   - Former: + - Current: +   # daily cig (dose):   - Men: + - Women: -   Pack/year (dose):   - Men: + - Women: -   Age started smoke:   - Men: + - Women: - | Never smoker: 1  (M&W)   - Men former: + - Men Current: + - Women former: - - Women current: +   # daily cig (dose): -  Pack/year (dose): -  Age started smoke:  Men: +  Women: -  Male current smokers presented a significantly increased HR of subsequent cancer occurrence compared with never-smokers [HR 1.64, 95% confidence interval (95% CI) 1.48–1.82]. Female current smokers also represented a significant increase (HR 1.46, 95% CI 1.21–1.75). | + |
| Janzon 2004 | Sweden | 10619 | 49 yrs (28.3-57.6) | 14.0±4.5yrs (range 0.5–21.9 years) | **Never smoker**   - Normotension 0 - Hypertension + - Norm Chol 0 - High Chol: + - No diabetes 0 - Diabetes +   Normotension 1.0  Hypertension 2.4 (1.4–4.3)  Normal cholesterol 1.0  High cholesterol 1.8(1.02–3.2)  No diabetes 1.0  Diabetes 8.8 (4.4–17.4)  **Ex-smoker**   - Normotension 0 - Hypertension + - Norm Chol 0 - High chol: + - No diabetes + - Diabetes +   Normotension 1.8 (0.99–3.2)  Hypertension 2.7 (1.1–6.0)  Normal cholesterol 1.6 (0.9–2.9)  High cholesterol 2.4 (1.2–5.0)  No diabetes 1.7 (1.01–2.8)  Diabetes 7.8 (2.4–25.6) | **Current smoker**   - Normotension + - Hypertension + - Norm Chol + - High chol: + - No diabetes + - Diabetes +   Normotension 5.3 (3.3–8.1)  Hypertension 12.2 (7.5–19.8)  Normal cholesterol 5.6 (3.6–8.6)  High cholesterol 8.2 (5.2–12.9)  No diabetes 6.0 (4.1–8.6)  Diabetes 19.0 (10.2–35.4) | + |
| Khalili 2002 | Sweden | 22 444 – (not clear) | Mean 42.2 | 17yrs | **Non-smoker: 1**   - CVD Morbidity: + - CVD Morbidity (in BP drugs): +   RR (95%CI) smokers and non-smokers  Q1 1.9 (1.5–2.4)  Q2 2.1 (1.8–2.5)  Q3 2.3 (1.8–2.9)  Q4 1.8 (1.5–2.1)  Q5 1.7 (1.5–2.0)  tHTs 1.4 (1.1–1.8). | **Non-smoker: 1**   - Mortality: + - Mortality (in BP drugs): +   RR (95%CI) smokers and non-smokers  Q1 1.8 (1.4–2.3)  Q2 2.5 (2.1–3.0)  Q3 2.7 (2.0–3.6)  Q4 2.2 (1.9–2.7)  Q5 2.5 (2.1–2.9)  tHTs 1.8 (1.3–2.5) | + |
| Kimm 2011 | Korea | 3252 | Men 51.9 ±8.7  Women 53.6 ±9.9 | 14yrs | AD & VD  **Men**   - Former: 0 - Current: 0 - Never smoker: 1   HR (95% CI)  AD  Ex-smokers 1.0(0.8–1.2)  Current smokers 1.1(0.9–3.4)  VaD  Ex-smokers 0.9(0.7–1.3)  Current smokers 1.1(0.8–1.5)  **Women**   - Former: 0 - Current: + - Never smoker: 1   HR (95% CI)  AD  Ex-smokers 1.2(0.9–1.5)  Current smokers 1.3(1.1–1.5)  VaD  Ex-smokers 0.9(0.5–1.5)  Current smokers 1.5(1.1–2.1) | **Unspec – both sex**   - Former: 0 - Current: + - Never smoker: 1   HR (95% CI)  Men  Ex-smokers 0.9(0.7–1.2)  Current smokers 1.2(1.0–1.5)  Women  Ex-smokers 0.9(0.7–1.2)  Current smokers 1.2(1.0–1.5)  **All – both sex**   - Former: 0 - Current: + - Never smoker: 1   HR (95% CI)  Men  Ex-smokers 1.0(0.8–1.1)  Current smokers 1.2(1.0–1.3)  Woman  Ex-smokers 1.1(0.9–1.3)  Current smokers 1.3(1.1–1.5) | + |
| Lim 2013 | Singapore | 48,251 | 45-74 | 93-98 to 2009 | All-cause   - New quitters: - - LT quitters: - - Never: - - Current smoker: 1   Other than lung cancer mortality   - New quitters: 0 - LT quitters: - - Never: - - Current smoker: 1   Lung cancer   - New quitters: - - LT quitters: - - Never: - - Current smoker: 1   Other than lung cancer mortality   - New quitters: 0 - LT quitters: - - Never: - - Current smoker: 1   Compared with current smokers, risk for total mortality was lower for new quitters (HR=0.84, [0.76, 0.94], long-term quitters (HR=0.61, [0.56, 0.67]), long-term quitters and never-smokers (HR=0.49, [0.46, 0.53]). Compared with current smokers, the risk of lung cancer mortality was lower for new quitters (HR=0.76 [0.57, 1.00) and long-term quitters (HR=0.44, [0.35, 0.57]) | CHD mortality   - New quitters: 0 - LT quitters: - - Never: - - Current smoker: 1   Stroke mortality   - New quitters: 0 - LT quitters: 0 - Never: - - Current smoker: 1   COPD mortality   - New quitters: 0 - LT quitters: - - Never: - - Current smoker: 1   Compared with current smokers, the risk for coronary heart disease mortality was lower for long-term quitters (HR= 0.63, [0.52,0.77]) | - |
| Mannami 2004 | Japan | 19,782 men and 21,500 women | 40-59 | 90-92 to 01 (total of 461,761 person-year follow-up) | **Current smoker – Men:**   - Total stroke: + - Intraparenchymal haemorrhage: 0 - Subarachnoid haemorrhage: + - Ischemic stroke + - Lacunar infarct: + - Large-artery occlusive infart: + - Embolic infarct: 0   Never smoked: 1  Former - Men or Women:   - Range of CV outcomes: 0 | **Current smoker – Women:**   - Total stroke: + - Intraparenchymal haemorrhage: 0 - Subarachnoid haemorrhage: na - Ischemic stroke 0 - Lacunar infarct: + - Large-artery occlu. Infart: na - Embolic infarct: 0   Risks for current smokers compared with never-smokers were higher for total stroke (RR=1.27, [1.05, 1.54]), subarachnoid hemorrhage (RR=3.60, [1.62, 8.01]), ischemic stroke (1.66, [1.25, 2.20]); the respective RRs among women were 1.98 (1.42 to 2.77), 1.53 (0.86 to 4.25), 2.70 (1.45 to 5.02), and 1.57 (0.86 to 2.87) | ++ |
| Moayyeri 2009 | UK | 25,311 | W: 64.7 (8.4)  M: 61.9 (9.7)  (40-75yrs) | 11.3yrs (SD = 1.5; range 9.2–14.1) | Osteoporotic fractures   - Women current: 0 - Men current: 0 - Never smoker: 1 | Any incident fracture  HR (95% CI)  Women  Smoking status (current) 1.10 (0.85–1.43)  Men  Smoking status (current) 1.19 (0.84–1.68)  Incident hip fracture  HR (95% CI)  Women  Smoking status (current) 1.19 (0.77–1.83)  Men  Smoking status (current) 1.38 (0.74–2.56) | + |
| Nakayama 2000 | Japan | 998 | 40-64yrs | 20yrs | Stroke  Smoking: +  PAF: 14.9 (+) |  | + |
| Noborisaka 2013 | Japan | 6998 | Men 84.3% bt 30-59yrs  Women 86.3% bt 30-59yrs | 6 yrs | Chronic kidney condition   - Former: 0 - Smoking: + - Non-smoker: 1 | Proteinuria  Odds ratio (95 % CI) p  Smoking status (vs. non-smokers) 0.002  Ex-smokers 1.29 (0.48–3.42) 0.614  Continuous smokers 2.52 (1.50–4.25) 0.001  Low eGFR  Odds ratio (95 % CI) p  Smoking status (vs. non-smokers) 0.006  Ex-smokers 1.05 (0.78–1.41) 0.735  Continuous smokers 0.74 (0.60–0.90) 0.003 | + |
| Nooyens 2008 | Netherlands | 1964 | 56.0 (7.0) | 5yrs | Cognitive decline   - Mem function: + - Speed of cog processing: + - Cog Flexibility: + - Global cog Fx: 0 - Never Smoker: 1 | b P  Memory function –0.04 .03  Speed of cog proc –0.02 .03  Cognitive flexibility –0.03 .04  Global cogn function –0.02 .06 | + |
| Nafziger 2007 | Sweden | 82927 | 30-60 | 10 years | Maintaining weight  Snus user: -  Non-user: + | Snuff use  No 1.00  Yes 0.83 (0.74, 0.92) | ++ |
| Östenson 2012 | Sweden | 2382 | 47.2  (46.9–47.4) | 10 years | Type 2 diabetes  Never use SNUS: 1   - Former: 0 - 1-5 boxes/w: 0 - >5 boxes/w: + - Consistent smo.: 0   OR 95% CI  Consistent never snus use 1.0  Consistent snus use 1.1 0.6–2.0  Former snus use 0.5 0.2–1.2  Consistent never snus use 1.0  1-5 boxes/week 0.6 0.2–1.4  >5 boxes/week 3.3 1.4–8.1 | - Never smoker: 1 - 1–15 cig/day: 0 - > 15 cig/day +   OR 95% CI  Consistent never smoking 1.0  Consistent smoking 1.5 0.8–3.0  Former smoking 0.9 0.5–1.7  Consistent never smoking 1.0  1–15 cigarettes/day 0.8 0.3–2.1  >15 cigarettes/day 2.4 1.0–5.8 | + |
| Ostbye 2002 | US | 7,845 | 51-61 years | HRS: 6yrs | Disability   - Heavy: + - Light: + - Former <3yrs: + - Former 3-15yrs: + - Former 15+yrs: 0 - Never smoked: 1 | Compared never smoked, current smokers had highest odds for IADL dependence (OR=1.46, [1.21, 1.77]), difficulty climbing stairs (OR=1.67, [1.37, 2.03]), difficulty walking (OR=2.06, [1.69, 2.49]), poor health (OR=1.55, [1.29, 1.87]), hospitalization (OR=1.28, [1.08, 1.52]), nursing home placement (OR=1.68, [1.08, 2.63]) | - |
| Otani 2003 | Japan | 19,862 (cohort 1)  10,212  (cohort 2) | 48.9 (6.0)  (cohort 1)  53.4 (8.2)  (cohort 2) | 10yrs  (cohort 1)  7yrs  (cohort 2) | Colorectal   - Former: 0 - Current: + - Never smoker: 1   Pack-years   - <20: 0 - 20–29: 0 - 30–39: + - 40+: 0   Invasive Colorectal   - Former: + - Current: + - Never smoker: 1   Pack-years   - <20: 0 - 20–29: 0 - 30–39: 0 - 40+: + | Colon / rectal   - Former: 0 - Current: 0 - Never smoker: 1   Pack-years   - <20: 0 - 20–29: 0 - 30–39: + - 40+: 0 | + |
| Patja 2005 | Finland | 41 372 | 25–64 | Mean follow-up 21 years | Type 2 diabetes  **Men:**   - Never smoker: 1 - Former: 0 - Current <20cig/day: + - Current >=20cig/day: +   **Women:**   - Never smoker: 1 - Former: 0 - Current <20cig/day: + - Current >=20cig/day: + | **Men and women combined:**   - Never smoker: 1 - Former: 0 - Current <20cig/day: + - Current >=20cig/day: +   HRs  Never smoking 1.00  Ex-smoking 1.09 (0.96–1.24)  Current smoker <20 cig/day 1.30 (1.15–1.47)  Current smoker >20 cig/day 1.65 (1.45–1.89) | - |
| Pelkonen 2000 | Finland | 1582 | Not reported | 30yrs | Mortality  Smokers across the entire range of pulmonary function may increase their expectation of lifespan by giving up smoking |  | ++ |
| Qiao 2000 | Finland | 1673 | Not reported | 35yrs | Mortality  Men smoking persistently were most at risk, while those who persisted in quitting had no increased risk of death compared with non-smokers |  | + |
| Qiu 2003 | China | 50,069 | 55.3±11.8 | 6yrs | CVD death   - Non-smoker: 1 - Former: 0 - Current: 0 | Hazard ratios  Non-smoker 1.00  Ex-smoker 1.40 (0.98- 2.00)  Current smoker 1.08 (0.87- 1.34)  p for trend 0.59 | + |
| Räikkönen 2001 | USA | 541 | 48.0±1.5 | 9.2yrs; SD, 3.4 years | Hypertension   - Smoking (no/yes) 0 | Predictor, b, P, HRs (95% CI)  Smoking status (no/yes) 0.40, 0.19, 1.50 (0.83–2.72) | + |
| Riserus 2007 | Sweden | 770 | 50 | 20 yrs (70-73 to 91-95) | Insulin sensitivity   - Never smoker: 1 - Smoking: 0 |  | + |
| Rusanen 2011 | USA | 21,123 | 50-60 | 17yrs | Dementia  Current-pack/d:   - <0.5: 0 - 0.5-1: + - 1-2: + - >=2: + - Never smoker: 1   Compared to non-smokers, the risk of dementia was higher among those smoking: more than 2 packs per day (HR=2.14, [1.65, 2.78]), 1-2 packs per day (HR=1.44, [1.26, 1.64]), and 0.5-1 packs per day (HR=1.37, [1.23, 1.52]). There was no association between smoking and AD risk | AD   - Former: 0 - Never smoker: 1   Current, pack/d:   - <0.5: 0 - 0.5-1: 0 - 1-2: 0 - >=2: +   VD   - Former: 0 - Never smoker: 1   Current, pack/d:   - <0.5: 0 - 0.5-1: 0 - 1-2: +   >=2: 0 | + |
| Sabia 2008 | England | 5388 | 35-55 | 85-88 to 97-99 | Memory   - LT ex-sm.: **–** - Recent ex.: 0 - Current sm.: + - Never smoker: 1   Reasoning   - LT ex-sm.: 0 - Recent ex-sm.: 0 - Current sm.: 0 - Never smoker: 1   Compared to never smokers, current smokers (OR=1.40, [1.11, 1.75]) and recent ex-smokers (OR=1.38, [1.07, 1.77]) were more likely to show a decline in reasoning and a decline in memory (OR=1.37, [1.10, 1.73]) | Vocabulary   - LT ex-sm.: **–** - Recent ex-sm.: **–** - Current sm.: 0 - Never smoker: 1   Phonemic fluency   - LT ex-sm.: **–** - Recent ex-sm.: 0 - Current sm.: 0 - Never smoker: 1   Semantic fluency   - LT ex-sm.: **–** - Recent ex-sm.: **–** - Current sm.: 0 - Never smoker: 1   Compared to never smokers, recent ex-smokers were less likely to show a decline in vocabulary, phonemic fluency, and semantic fluency (OR=0.73, [0.60, 0.87]; OR=0.73, [0.61, 0.87]; and OR=0.75, [0.63, 0.89], respectively); similar findings were reported for recent ex-smokers | + |
| Sabia 2009 | England | 5123 | Mean 56yrs | 5yrs | Cognitive function   - Current smoking no: 1 - Current smoking yes: + | The odds for poor executive function were higher among current smokers compared to non-smokers (OR=1.30, [1.01, 1.67]) | + |
| Satoh 2006 | Japan | 2,764 | 35-44 | 10yrs | Coronary Artery Disease   - Non-smoker: 1 - Smokers: 0 | HR (95% CI) p-value  Smoking 2.47 (0.86-7.10) 0.09 | + |
| Sairenchi 2004 | Japan | 39,528  men and 88,613 women | 40-79  (sub group: 40-59) | 93-02 | Type 2 diabetes mellitus  **Men**   - Never smoker: 1 - Former: + - Current: + - < 20 cig/day: + - >=20 cig/day: +   Among those 40-59 years of age, current smokers had greater risk of type 2 diabetes compared to never smokers (RR=1.37, [1.18, 1.60]). Among those 60-79 years, current smokers had higher risk for type 2 diabetes compared to never smokers (RR=1.20, [1.08, 1.34]) | **Women**   - Never smoker: 1 - Former: 0 - Current: + - <20 cig/day: + - >=20 cig/day: +   Among those 40-59 years of age, current smokers had greater risk of type 2 diabetes compared to never smokers (RR=1.45, [1.18, 1.79]). Among those 60-79 years, current smokers had higher risk for type 2 diabetes compared to never smokers (RR=1.34, [1.09, 1.66]) | + |
| Shaper 2003 | Britain | 7735 | 40–59 | 22yrs  (’78-‘00) | Total mortality   - Never smoker: 1 - Pipe/cigar: + - Former: 0 - Current: + | Cancer   - Never smokers: 1 - Pipe/cigar: + - Former: + - Current: + | ++ |
| Sobue 2002 | Japan | 91,738 | 40–69 | 9yrs | Squamous cell small cell carcinoma   - Non-smoker: 0 - Former: + - Current: +   **Men**  Non-smoker 1.0  Former smoker 5.1 (1.8–14.6)  Current smoker 12.7 (4.7–34.7)  **Women**  Non-smoker 1.0  Former smoker 10.8 (1.2–94.4)  Current smoker 17.5 (4.9–62.1) | Adenocarcinoma   - Non-smoker: 0 - Former: - - Current: +   **Men**  Non-smoker 1.0  Former smoker 1.3 (0.7–2.5)  Current smoker 2.8 (1.6–4.9)  **Women**  Non-smoker 1.0  Former smoker 4.3 (1.3–13.8)  Current smoker 2.0 (0.8–5.0) | ++ |
| Stevens 2009 | England Scotland | 1.3 million | 50-60  (women) | 5-9yrs | Cancers   - Never smoker: 0 - Former: - - Current (<15): + |  | ++ |
| Strand 2013 | Norway | 48,793 | 35–50 | 35yrs | Dementia death  Non-smokers: 0  Current <15: 0  Current 15+: 0 |  | ++ |
| Strandberg 2008 | Finland | 1658 | 40-55 | 26yrs | Mortality   - Never smoker: **+** - Smoking: **–**   Amount of daily cigarettes predicted mortality in a graded manner (P<.001). | Bodily pain, general health, Mental health/emotional wellbeing, role limitations owing to mental problems or to physical health, Social functioning, Energy vitality, Physical functioning   - Never smokers: **+** - Smoking: **0**   The physical component summary score showed a graded deterioration of HRQoL with an increasing number of cigarettes smoked daily (global P=.01) | + |
| Szoeke 2006 | Australia | 438 | 46-52 | 11 years | Osteoarthritis  Never smokers: 1  Smoking: + | Hand OA  RR (95.0% CI)  Smoking ever 1.01 (1.0–5.7)  P 0.35  Knee OA  RR (95.0% CI)  Smoking ever 0.9 (0.8–1.0)  P 0.05 | + |
| Tyas 2003 | Hawaii | 3734 | (mid-life) | (‘65–‘71) and  (‘91–’96) | Vascular dementia  Smoking: +  VaD  Never 1.0  Former 0.82 (0.43–1.52)  Current 1.14 (0.60–2.13) | AD  Never 1.0  Former 0.93 (0.58–1.50)  Current 1.17 (0.69–1.98)  AD ± CVD  Never 1.0  Former 0.88 (0.58–1.33)  Current 1.31 (0.85–2.01)  All dementia  Never 1.0  Former 0.80 (0.58–1.10)  Current 1.11 (0.79–1.55) | + |
| Whitmer 2005 | USA | 8,845 | 40-44 | 27yrs  (‘64-‘03) | Dementia  Never smoker: 1  Smoking: + | HR (95% CI)  1.26 (1.08–1.47) | + |
| Wilcox 2006 | USA | 5820  Japanese American men | Mean 54yrs (45-68) | Up to 40yrs | Non-survival vs survival at 85yr   - Ever smoker: +   OR (95% CI) P Value  Ever smoker 1.94 (1.72-2.18) .001 | Usual vs Exceptional survival   - Ever smoker: + *(borderline association)*   OR (95% CI) P Value  Ever smoker 1.23 (1.01-1.50) .04 | ++ |
| Wannamethe 2001 | UK | 7735 | 40-59 | 16.8 yrs | Type 2 diabetes  Never smoker: 1  Smoking: + |  | + |
| ***Alcohol***  Note: A positive association (+) with alcohol is the worst outcome | | | | | | | |
| Anttila 2004 | Finland | 632 women and 386 men | Mean age 48.3 yrs | 1972-77 to 1998 | Dementia:  Never: 0  Infrequent: 1.00  Frequent: 0  Among carriers of the APOE4, the risk of dementia was greater for frequent drinkers compared to non-drinkers (OR=7.07, [1.37, 36.60]) | Mild cognitive impairment:  Never: +  Infrequent: 1.00  Frequent: +  Odds for MCI higher for those who never drank and those who drank frequently compared to infrequent drinkers (OR=2.15 [1.01, 4.59] and OR=2.57 [1.19, 5.52], respectively) | + |
| Beulens 2007 | Netherlands | 1,417 | 49-70 yrs | 1993-97 to 2005 | CVD risk  U-shaped relationship between alcohol-intake and CVD risk |  | ++ |
| Elwood 2013 | Caerphilly, UK | 1,320 men | 45–59 | 1979-04 | Diabetes: 0  Vascular disease: 0  Cancer: 0  Any impairment: 0  Dementia: 0  Death: 0 |  | + |
| Emberson 2005 | England,Wales, and Scotland | 7,735 | 40–59 | 1978/1980 to 1998/2000 | Cardiovascular morbidity  Alcohol level  None 1.00  Occasional 1.00  Light : -  Moderate *0*  Heavy +  Major coronary heart disease  Hazard ratio 95% CI  None 0.91 (0.72, 1.15)  Occasional 1.00  Light 0.74 (0.63, 0.87)  Moderate 1.01 (0.84, 1.21)  Heavy 1.74 (1.31, 2.33)  Stroke  Hazard ratio 95% CI  None 1.08 (0.73, 1.60)  Occasional 1.00  Light 0.93 (0.71, 1.22)  Moderate 1.45 (1.08, 1.96)  Heavy 2.33 (1.46, 3.71) | All-cause mortality  Alcohol level  None: 0  Occasional 1.00  Light: -  Moderate: 0  Heavy: +  All-cause mortality  Hazard ratio 95% CI  None 0.93 (0.77, 1.12)  Occasional 1.00  Light 0.82 (0.72, 0.93)  Moderate 1.32 (1.15, 1.52)  Heavy 2.27 (1.84, 2.81) | ++ |
| Englund 2013 | Sweden | 778 | 49-61 | 85-08 | Wrist fracture  Alcohol level  None 1.00  User: 0 |  | + |
| Flood 2008 | USA | 49238 | Older than 50 y | 1995–1996 to 2000 | Colorectal cancer  Alcohol level  None 1.00  User: 0 |  | ++ |
| Iso 2004 | Japan | 19 544 | 40-59 | 1990-2000 | Stroke  Alcohol level  <450g ethanol pw: 0  >450 g ethanol pw: + |  | + |
| Lin 2005 | Japan | 110,792 | 40 to 79 years | 1988–1990 to 1999 | All-cause mortality  Alcohol level  <23 g/d 0  >23 g/d + | RR (95%CI)  Nondrinkers 1.00  Ex-drinkers 1.58 (1.44–1.74)  0-22.9 g/day: 0.80 (0.72-0.88)  23-45.9 g/day: 0.90 (0.82-0.98  46-68.9 g/day: 0.95 (0.86-1.04  > 69.0 g/day: 1.32 (1.18-1.48) | ++ |
| Moayyeri 2009 | UK | 25311 | 40-75 years | 1993–1997 to 2007 | Osteoporotic fractures  **Men**  None 1.00  User: +    HR (95% CI)  Any incident fracture 1.01 (1.01–1.02)  Incident hip fracture 1.01 (0.99–1.03) | **Women**  None 1.00  User: 0  HR (95% CI)  Any incident fracture 0.98 (0.97-1.00)  Incident hip fracture 0.99 (0.97-1.02) | + |
| Ostbye 2002 | US | HRS study: 7,845 people | HRS study: ages 51-61 yrs | HRS: 92-98 | Disability, health and health care utilization  Up to 2 drinks/day (ref: never drinking)  ADL dependence: -  Difficulty climbing stairs: -  Difficulty walking: -  Poor health: -  Hospitalized: -  2+ drinks/day (ref: never drink.)  ADL dependence: 0  Difficulty climbing stairs: 0  Difficulty walking: 0  Poor health: -  Hospitalized: 0 | Past drinking problem (ref: never drink.)  ADL dependence: +  Difficulty climbing stairs: +  Difficulty walking: +  Poor health: +  Hospitalized: +  Compared to those who never drink, those with a past drinking problem had the highest odds for ill health in terms of difficulty climbing stairs (OR=1.37, [1.07, 1.75]), hospitalization (OR=1.38, [1.13, 1.68]) | - |
| Otani 2003 | Japan | 90004 | 40–59 Cohort 1  40–69  Cohort 2 | Cohort I  After January 1, 1990-1999  Cohort II  January 1, 1993–1994-1999 | Colorectal cancer  **Men**  None 1.00  User: +  **Women**  None 1.00  User: 0 | Alcohol consumption and smoking were associated with colorectal cancer in men. Regular ethanol consumption was not associated with colorectal cancer in women. | + |
| Qiu 2003 | China | 50069 | 40-80+ | 1994/1996-2000 | CVD death  Non-drinker 1.00  Ex-drinker: 0  Current drinker: 0 | HR (95% CI)  Non-drinker 1.00  Ex-drinker 1.55 (1.04- 2.31)  Current 1.12 (0.93-1.34)  p for trend 0.23 | + |
| Sabia 2009 | England | 5,123 | Mean age 56 yrs | 97-99 to 02-04 | Cognitive function  Alcohol consumption (units/week):  0: +  1-14: 1.00  >=15: 0 | The odds for poor memory were higher among non-drinkers compared to those who consumed 1-14 units/week (OR=1.34, [1.08, 1.66]), those who consumed fruits and vegetables >=2 times per day compared to those who consumed less than this amount (OR=1.35, [1.14, 1.59]) | + |
| Sabia 2011 | France | 4073 men | Ages 40–50  for men and 35–50 for women | 10 yrs (1992 to 2002-04) | Cognitive performance  0 drinks/wk: 0/0/0  1-3 d/wk: 0/0/0  4-14 d/wk: 1.00  15-21 d/wk: 0/0/0  >21 d/wk: -/0/0 | Consumption, great increase and decrease in alcohol consumption were both associated with lower DSST score only in the low occupational group (−3.9 points (95% CI: −6.1, −1.7) and −3.5 points (95% CI: −6.2, −0.7) respectively for high increase and decrease compared to stable alcohol consumption); p for interaction=0.003 | + |
| Stevens 2009 | England and Scotland | 1.29 million women |  | 96-01 to 05-07  Mean yrs of follow-up: 7.2 for cancer incidence; 8.9 for mortality | Incident and fatal pancreatic cancer  None: + / +  1-2: 0 / 0  3-6: - / -  7-13: 0 / 0  14+: 0 / 0 |  | + |
| Sun 2011 | US | 13,894 | 70+ | 84-00 | Successful ageing  Days of alcohol use/week:  Nondrinker: 1.00  1-2: 0  3-4: +  5-7: + | Odds of successful aging to age 70+ higher among those consuming 5.1-15.0 g/day and 15.1-30.0 g/day alcohol compared to non-drinkers (OR=1.19, [1.01, 1.40], and OR=1.28, [1.03, 1.58], respectively) | ++ |
| Tabak 2001 | Finland, Italy, Netherlands | 2,953 men (Finland: 1,186 men; Italy: 1,183; Netherlands: 667) | 40-59 | 20 yrs (1965-70 to 1990) | Chronic obstructive pulmonary disease mortality  Nondrinkers: 1.00  Light drinkers: 0  Higher alcohol consumption: 0  e.g., >9 drinks /day: 0 |  | - |
| Virta 2010 | Finland | 1,486 | Mean age in 1981: 51.7 yrs (SD: 6.1) | 1975-81 to 1999-07 (mean follow-up: 22.8 yrs.) | Cognitive function  Abstainer: +  Light drinker: 1.00  Moderate: 0  Heavy: +  Binge drinking: no: 1.00  Yes: +  Number of pass-outs:  0: 1.00  1: 0  >2: + | Alcohol consumption in 1981  OR (95% CI)  Abstainer 1.51 (1.04–2.18)  Light 1.00  Moderate 0.87 (0.58–1.32)  Heavy 2.03 (1.17–3.54)  Binge drinking in 1976 and 1981  Neither 1976 nor 1981 1.00  Only 1976 or 1981 1.87 (1.04–3.38)  Both 1976 and 1981 1.98 (1.05–3.72)  Number of pass-outs in 1981  0 1.00  1–2 0.94 (0.50–1.78)  > 2 4.10 (1.54–10.94) | ++ |
| Wannamethee 2002 | England,Wales, and Scotland | 7157 | 40-59yrs | (’78–’80)-(2000) | Major coronary heart disease events   - Non-drinkers: 1.0 - Occasional drinkers who took up regular drinking: + - Continuing regular drinkers: + - Stable occasional drinkers: 0   TT (95% CI)  CHD mortality 1.71 (0.92 to 3.15)  CVD mortality 1.62 (0.93 to 2.82)  Total mortality 1.31 (0.82 to 2.10)  New occ  CHD mortality 1.05 (0.62 to 1.79)  CVD mortality 1.07 (0.66 to 1.74)  Total mortality 1.00 (0.67 to 1.51)  Reg  CHD mortality 1.33 (0.88 to 2.00)  CVD mortality 1.33 (0.92 to 1.93)  Total mortality 1.25 (0.92 to 1.69) | Ex  CHD mortality 1.56 (0.89 to 2.73)  CVD mortality 1.49 (0.89 to 2.49)  Total mortality 1.46 (0.96 to 2.22)  Stable occ  CHD mortality 1.00  CVD mortality 1.00  Total mortality 1.00  New reg  CHD mortality 1.19 (0.60 to 2.34)  CVD mortality 1.30 (0.72 to 2.37)  Total mortality 1.23 (0.75 to 2.04) | + |
| Waki 2005 | Japan | 28,893 | 40-59 yrs | 10 yrs (baseline: 1990) | Incident type 2 diabetes  **Men**   - Non-drinkers and eth. intake on <=3 days/month: 1.00 - 0 <ethanol <=4.9: 0 - 4.9 < ethanol <=11.5: + - >11.5 ethanol: 0   Odds of developing diabetes higher among men drinking between 23-46g ethanol/day than ‘non-drinkers and infrequent occasional drinkers’ (OR=1.26, [1.02-1.56]) | **Women**   - Non-drinkers and eth. intake on <=3 days/month: 1.00 - 0 <ethanol <=4.9: 0 - 4.9 < ethanol <=11.5: 0 - >11.5 ethanol: 0 | + |
| Wannamethee 2003 | England, Wales, Scotland | 7,608 men | 40-59 yrs | 1978-80 to 1983-85 | BMI  Stable intake:  None-occasional: 1.00  Light-moderate: 0  Heavy: +  Odds of weight gain >=4% over 5 years greater for stable heavy drinkers compared to stable none-occasional drinkers (OR=1.29 [1.10, 1.51]). Odds of weight gain >=4% over 5 years were greater for new heavy drinkers compared to the stable none-occasional group (OR=1.45, [1.09, 1.92]) | Changed intake:  Light-moderate (*at baseline*) to none-occasional (*at f/u*): 0  None-occasional to light-moderate: 0  Ex-heavy: 0  New heavy: + | + |
| Wang 2010 | US | 19,220 | 38-89 yrs | 12.9 yrs. follow-up (baseline: 1992-95) | Overweight  Total alcohol intake (g/day)  0: 1.00  >0- <5: 0  5- <15: -  15- <30: -  >= 30: -  Increasing levels of alcohol consumed contributed to decreasing incidence of overweight or obesity (BMI >=25); this trend was observed for ‘all alcohol’, beer, red wine, white wine (trend test p-values: <0.0001, 0.04, < 0.0001, and <0.0001, respectively) | Obesity  Total alcohol intake (g/day)  0: 1.00  >0- <5: -  5- <15: -  15- <30: -  >= 30: -  Increasing levels of alcohol consumed contributed to decreasing incidence of obesity (BMI >=30); this trend was observed for ‘all alcohol’, beer, red wine, white wine, liquor (trend test p-values: <0.0001, 0.02, 0.004, 0.0003, 0.005) | + |
| Willcox 2006 | island of Oahu | 5820 | 45-68yrs | Not reported | Overall survival  Alcohol level  <3 1.00  >3 – | Age-Adjusted ORs  Nonsurvival vs Survival  OR (95% CI) P Value  High (>3 drinks/d)  1.97 (1.68-2.31) .001  Usual Survival vs Exceptional Survival  High (>3 drinks/d)  1.84 (1.29-2.62) .001  Stepwise Logistic Regression  Nonsurvival vs Survival  High (>3 drinks/d)  1.58 (1.34-1.88) .001  Usual Survival vs Exceptional Survival  High (>3 drinks/d)  1.61 (1.11-2.34) .01 | ++ |
| Xu 2010 | South East Queensland, Australia | 564 | 45-60 yrs | 2001-06 | Anxiety  Never: ref  Past drinker: -  Occasionally: 0  Regularly: 0  Depression  Never: ref  Past drinker: 0  Occasionally: 0  Regularly: 0 | Psychological symptoms  Never: ref  Past drinker: 0  Occasionally: 0  Regularly: 0  SF-36 mental health  Never: ref  Past drinker: 0  Occasionally: 0  Regularly: 0 | - |
| ***Weight Change/Weight Cycling*** | | | | | | | |
| Field 2009 | United States | 44842 | 30-55 | 16 years | Mortality  Mild: 0  Severe: 0 |  | + |
| Langlois 2001 | United States | 2180 | 50-74 | 22 years | Hip Fracture  50–64 years: +  65–74 years: + | Women (50-64 years) at BL with >=10% weight loss had a greater risk of hip fracture compared to women with <5% weight loss (RR=2.54, [1.10, 5.86])  Women (65-74 years) at BL with >=10% weight loss had a greater risk of hip fracture compared to women with <5% weight loss (RR=2.04, [1.37, 3.04])  Women with BMI <26.2 (kg/m2) at BL with >=10% weight loss had a greater risk of hip fracture compared to women with <5% weight loss (RR=2.37, [1.32, 4.27]) | ++ |
| Ravona-Springer 2013 | Israel | 10000 | 40-70 | 36 years | Dementia  Wt change I: 0  Wt change II: 0  Wt change III: +  Wt change IV: + | OR (95%CI) p  I 1.43 (0.75–2.71) NS  III 1.05 (0.79–1.40) NS  IV 1.25 (0.73–2.14) NS | + |
| Waring 2010 | United States | 1577 | 40-50 | 11 years | Diabetes  Weight loss: 0  Weight gain: 0  Weight cycling: 0 |  | ++ |
| ***Combined Lifestyles*** | | | | | | | |
| Agrigoroaei 2011 | United States | 4995 | 33-84 | 9-10 years | Episodic memory  Behavioural protective factors +  Executive functioning  Behavioural protective factors + | The number of behavioural protective factors were positively associated with memory (b = 0.03, p = 0.032) and executive functioning, (b = 0.06, p < 0.001), and a significant percent of model variance was explained by these factors over and above the confounders assessed (R2 change = 0.001, R2 change = 0.003, respectively) | ++ |
| Elwood 2013 | Caerphilly, UK | 1,320 men | 45–59 | 1979-04 | Diabetes: 0  Vascular disease: 0  Cancer: 0  Any impairment: 0  Dementia: 0  Death: 0 | Odds of diabetes were lower among those who regularly exercised (OR=0.63, [0.46, 0.85]). Odds of vascular disease lower among non-smoking (OR=0.70, [0.58, 0.84]); similar findings reported for cancer (OR=0.65, [0.54, 0.79]). Odds of cognitive impairment were lower for those who regularly exercised (OR=0.62, [0.41, 0.92]); similar findings reported for dementia. The odds of death were lower among those who did not smoke (OR=0.42, [0.35, 0.51]) | + |
| King 2007 | United States | 15708 | 45-64 | 11-13 years | Cardiovascular disease  1 healthy behaviour: 0 *(nsig)*  2 healthy behaviours: 0 *(nsig)*  3 healthy behaviours: - *(sig)*  4 healthy behaviours: - *(sig)*  OR (95% CI)  Switched from Unhealthy to Healthy Lifestyle 0.65 (0.52-0.81)  Persistently Unhealthy (<4 Healthy Factors at Both Visits) 1.00 (reference) | Mortality  1 healthy behaviour: 0 *(nsig)*  2 healthy behaviours: - *(sig)*  3 healthy behaviours: - *(sig)*  4 healthy behaviours: - *(sig)*  OR (95% CI)  Switched from Unhealthy to Healthy Lifestyle 0.60 (0.39-0.92)  Persistently Unhealthy (<4 Healthy Factors at Both Visits) 1.00 (reference) | ++ |
| ***Leisure/Cognitive Activity/Social Networks*** | | | | | | | |
| Bielak 2012 | Australia | 7152 | 20-24: 2404  40-44: 2530  60:64: 2551 | 7 years | Perceptual speed  Activity Engagement  20: +  40: +  60: +  Short-term memory  Activity Engagement  20: +  40: +  60: +  Working memory  Activity Engagement  20: +  40: +  60: + | Episodic memory  Activity Engagement  20: +  40: +  60: +  Vocabulary  Activity Engagement  20: +  40: +  60: +  Activity-between X Age Group Estimate (SE)  60 vs. 40  Symbol Digit -0.06 (.02)  CVLT-Immediate -0.07 (.03)  Digit Backwards -0.002 (.03)  CVLT-Delayed -0.02 (.03)  Spot-the-word -0.03 (.03) | ++ |
| Britton 2008 | UK (England) | 5823 | 35-55 | 17 years | Successful aging  Low: 1  Medium 0  High 0 |  | + |
| Friedland 2001 | United States | 193 cases/358 controls  (for total study, not reported for 40-59 year olds) | 40-59 | >12  (not fully reported) | Alzheimer’s Disease  Intellectual activity: -  Physical activity: -  Passive activity: - | Comparisons between case- and control-group members on diversity scores (adjusted means)  Passive diversity F(1, 544) =19.25; Intellectual diversity F(1, 544)= 33.33; Physical diversity F(1, 544)= 29.24  Comparisons between case- and control-group members on ‘‘intensity’’ scores (adjusted means)  Passive diversity F(1, 544) = 0.16; Intellectual diversity F(1, 544)= 3.82; Physical diversity F(1, 544)= 0.96 | - |
| Holtzman 2004 | United States | 354 | 50+ | 12.4 years | Cognition  Risk of low MMSE score: - | A linear effect was observed for baseline network size (p=0.006, effect size=0.06); also, less increase - more decrease in network size is associated with decreased wave 3 MMSE (p=0.03, effect size=-0.06). Top tertiles of interpersonal activity and emotional support were significantly related to MMSE scores (activity betas=0.13 and 0.12, p <=0.04; support betas 0.16 and 0.17, p<=0.01) | ++ |
| Kareholt 2011 | Sweden | 1643 | 57.4 | 20+ years | Cognition  Political activity: +  Mental activity: +  Socio-cultural activity: +  Social activity: 0  Organisational activity: 0  Physical activity: 0 | β p-Value  Political 0.17 0.004  Mental 0.11 0.047  Socio-cultural0.04 0.415  Social 0.01 0.904  Organizational -0.03 0.628  Physical, all 0.21 0.05 0.375  Men -0.06 0.477  Women 0.14 0.055 | ++ |
| Raikkonen 2001 | United States | 541 | 42-50 | 9.2 years | Hypertension  Women: 0 |  | ++ |
