# Supplementary material for: Behavioural Risk Factors in Mid-Life Associated with Successful Ageing, Disability, Dementia and Frailty in Later Life: A Rapid Systematic Review
Source: PLoS One. 2016 Feb 4;11(2):e0144405. doi: 10.1371/journal.pone.0144405 (PMC4742275; doi:10.1371/journal.pone.0144405)
Supplement: S4 Table — (DOCX) [file pone.0144405.s004.docx]

**Full Data Extraction**

| **Study Details: authors, year, citations, country of study, aim of study, study design, quality score [++, +, or -], applicability [++, +, or -]** | | | | | | | | | | | | | |
| --- | --- | --- | --- | --- | --- | --- | --- | --- | --- | --- | --- | --- | --- |
| **Source population** | **Study (eligible and selected) population** | | | | **Exposures at midlife** | | | | **Outcomes at 55y or over** | | **Analysis** | | **Results, Limitations, Source of Funding** |
| **Authors**: Agahi N, Shaw BA **Year:** 2013 **Citation:** Prev Med. 2013;57(2):107-112 **Country of study:** Sweden **Aim:** To assess smoking trajectories from midlife to old age and the development of non-life-threatening health problems in a 34-year time span **Study design:** Longitudinal **Quality score:** -  **Applicability:** | | | | | | | | | | | | | |
| **Source population:**  *data for this study originated from the Swedish Level of Living Survey, a nationally representative study of Swedish people ages 18-75 years, and the Swedish Panel Study of Living Conditions of the Oldest Old, which comprised participants from the first study over the age of 75 years | **Study population:**  *data from the 1968, 1981, 1991 and 2000 phases of first study merged with data from 2002 phase of second study *up to 34 years follow-up of individuals that were 30-50 years of age at baseline in 1968 *final sample: **1060** people (52% of original sample)  *Follow-up:* *of the people meeting the inclusion criteria, 655 (32%) died during follow-up  *those who died during follow-up were older, less well educated, more likely to smoke, had more mobility impairment and psychological distress at baseline compared with those included in study  **Exclusion:**  -  **Attrition:**  *of 2051 people meeting the inclusion criteria, 336 (16%) people did not participate in study phases or had missing variable values (and 655 or 32% died) | | **Exposures:**  >smoking was assessed using structured participant interviews  >smoking status trajectories assessed in 1968, 1981, 1991, 2000/2002  *smoking categories: current non-smoking, light smoking (< 10 cigarettes/day), heavy smoking (10+ cigarettes/day)  *persistent heavy smokers (n=81): those who smoked throughout the period, with heavy smoking reported for at least 3 study waves *persistent light smokers (n=63): those who smoked throughout the follow-up period, but with two or fewer episodes of heavy smoking  *former smokers: smoking in the first and/or second study waves * former heavy smokers (n=107): those who reported mostly heavy smoking *former light smokers (n=176): light smokers  *persistent non-smokers (n=633): never smoked | | | | | **Outcomes:**  >3 outcomes assessed using structured interviews: mobility impairment, musculoskeletal pain, psychological distress >mobility impairment: index measuring ability to walk, run, go up and downstairs without difficulties (0=no mobility problems; 3=mobility in all 3 domains) >index of musculoskeletal pain and index of psychological distress based on summary score of health problems in past 12 months ranging from 0 (no pain) to 6 points (severe pain) and from 0 (no symptoms) to 8 points (severe problems in all domains assessed), respectively  *musculoskeletal pain index assessed perceived pain in hands, elbows, legs or knees, shoulders, back, hips, sciatica *psychological distress index assessed anxiety, nervousness, anguish, general fatigue, sleeping problems, depression | | **Analysis:**  *multivariate, multinomial regression analysis was used to assess smoking trajectories from midlife to old age and the development of non-life-threatening health problems   **Confounders:**  *age, education, sex | | **Results:**  *rate of increase in mobility impairment was steepest among persistent heavy smokers (coeff.=0.01, SE=0.004), and former heavy smokers (coeff.=0.01, SE=0.003) in comparison with non-smokers over 34 years of follow-up  *compared to non-smokers, former light smokers had statistically significantly steep progression of mobility problems over 34 years of follow-up (coeff.=0.006, SE=0.003)  *compared to the persistent non-smoking group, faster increases in mobility impairment were observed for all smoking trajectory groups  *heavy smokers had higher levels of psychological distress at baseline compared to persistent non-smokers, and this difference did not change over the follow-up period  **Limitations:**  *selective survival, leaving healthier individuals in sample *shorter period for observing the development of health problems for younger segments (follow-up until 2000) than for older segments (until 2002) in study; thus number of health problems may be underestimated in younger participants  **Source of Funding:**  *none reported | |
| **Authors**: Agrigoroaei S, Lachman ME **Year:** 2011 **Citation:** J Gerontol B Psychol Sci Soc Sci. 2011;66:Suppl 1 **Country of study:** United States **Aim:** To examine combined effect of psychological, social, and physical factors on cognitive functioning (above and beyond the effects of sodiodemographics, risk factors, cognitive activities **Study design:** Longitudinal **Quality score:** +  **Applicability:** | | | | | | | | | | | | | |
| **Source population:**  *data used from 1995-96 and 2004-05 national survey called Midlife in the United States (MIDUS), as well as a survey subsample, the Boston Longitudinal Study (BOLOS) *BOLOS measurements taken one year after MIDUS measurements *overall purpose: are behavioral, social, psychological, biological, neurological factors assessed in MIDUS associated with cognitive performance measured in MIDUS, as well as with subsequent cognitive functioning measured over 10 year-period in BOLOS | | **Study population:**  Specific study aims: *MIDUS Time 2 (2004-05): cross-sectional assessment of influence of psychosocial and behavioural factors on cognitive performance *influence of changes in the number of factors from Time 1 (1995-96) to Time 2 (2004-05) on cognition at Time 2  *national probability sample of 4,238 non-institutionalized adults from 48 states selected through random-digit dialing  >included 949 siblings of main respondents *1,913 twins selected from national sample of 50,000 households *Time 1: **7,100** participants ages 24-75 years (mean=46.40, SD=13.00) *Time 2: **4,900** participants ages 32-84 years (mean=55.45, SD=12.44) remaining (75% of people from Time1) *overall response rate: 70%   *1 year after MIDUS Time 1, **302** people ages 24-74 years (mean=47.89, SD=13.74), living in Boston recruited for BOLOS *1 year after MIDUS Time 2, **151** people who participated in first BOLOS wave participated in second BOLOS wave (68% participation rate); participants ages 34-84 years (mean=59.99, SD=12.81)  **Exclusion:**  -  **Attrition:**  *participants at Time 2 indicated positive selection on variables compared to those who dropped out *participants of BOLOS Time 2 were more education compared to non-participants of this wave | | **Exposures:**  *control beliefs: perceived control over life outcomes assessed using MIDUS sense of control scale, with scores ranging from 1-7 (higher values = higher sense of control) *quality of social support: 12 items assessed social strain in relationships, with scores ranging from 1-4 (higher values = higher quality of social support) *physical exercise: frequency of engaging in vigorous physical activities, with scores ranging from 1-6 (higher values = more frequent physical exercise) *psychosocial and behavioural protective composite score of above variables created (scores of aforementioned variables summed and higher values represent greater number of factors present at higher level) *Exposures assessed:* *influence of MIDUS protective composite  *interaction of MIDUS composite with age and education *influence of Time 1 MIDUS protective composite score and difference in MIDUS protective composite scores between Time 2 and Time 1 | | | **Outcomes:**  *7 cognitive domains assessed over telephone at Time 2 in MIDUS using the Brief Test of Adult Cognition by Telephone; cognitive factors grouped into episodic memory and executive functioning  *cognitive factors, such as short-term memory, speed of processing, reasoning, and vocabulary, assessed in person at Time 1 and Time 2 in BOLOS | | | **Analysis:**  *hierarchical multiple regression  **Confounders:**  *frequency of engaging in cognitive activities *age sex, level of education, race, waist circumference, smoking, alcohol or drug problems  *health status including history of diabetes, stroke, lupus, HIV or AIDS, MS, epilepsy or other neurological disorders, cancer, heart disease | | **Results:**  *MIDUS:* *the number of behavioural protective factors were positively associated with memory (b = 0.03, p = 0.032) and executive functioning, (b = 0.06, p < 0.001), and a significant percent of model variance was explained by these factors over and above the confounders assessed (R2 change = 0.001, R2 change = 0.003, respectively) *the association between education and cognition was reduced by the number of protective factors for episodic memory (b = –0.04, p = 0.015)  *BOLUS:* *Time 1 protective composite positively associated with change in reasoning (b = .10, p = .045) *the number of protective factors reduced the association between education and reasoning abilities when the interactions of the protective composite with age and education were entered in the model (b = –.09, p = .045)  **Limitations:**  *small sample size of BOLUS *approach used to compute protective composite cannot provide universal guidelines (method of dividing participants into high and low categories may not be clinically meaningful) *optimization of self-reported measures through use of multiple indicators *residual confounding from unexplored variables such as level of stress, personality profiles, nutrition  **Source of Funding:**  *National Institute on Aging and the John D. and Catherine T. MacArthur Foundation Research Network on Successful Midlife Development | |
| **Authors**: Akbaraly T, Sabia S, Hagger-Johnson G, Tabak AG, Shipley MJ, et al. **Year:** 2013 **Citation:** Am J Med. 2013;126(5):411-419 **Country of study:** England **Aim:** The association between diet at midlife assessed using dietary patterns and adherence to the Alternative Healthy Eating Index (AHEI) and overall health at older ages **Study design:** Longitudinal **Quality score:** +  **Applicability:** | | | | | | | | | | | | | |
| **Source population:**  *London-based office staff ages 35-55 years and working in civil service departments; recruited for Whitehall II study *baseline screening of 10,308 participants in 1985-1988 | | **Study population:**  *baseline: phase 3 of study took place 1991-1996 and 8815 people included *study comprised 5350 people at least 60 years of age at end of follow-up in phase 9 (2007-2009) ***3775** (70.6%) men and **1575** (29.4%) women included  *Follow-up:* *16-years  **Exclusion:**  *participants with no history of stroke, myocardial infarction, or cancer (n=7032) in 1991-1996 at phase 3 *for this particular study, 1682 excluded (more likely to be women, younger, less likely to have a higher AHEI score)  **Attrition:**  *included participants were less likely to be younger, to have a higher AHEI score, and better health outcomes compared to excluded participants | | | | **Exposures:**  >at phase 3, participants completed a semi-quantitative food-frequency questionnaire (hence, self-report)  *nutrient intakes of food items were computed by multiplying the consumption frequency for each food by its nutrient content and then summing the nutrient contributions from all foods  *validity and reliability of questionnaire has been established  *Dietary variables (exposure) for each participant included:  1. dietary patterns: “healthy-foods” diet versus “Western-type” diet (see analysis) 2. AHEI score based on intake of vegetables, fruits, nuts and soy, ratio of white meat (seafood and poultry) to red meat, total fiber, trans fat, ratio of polyunsaturated fat to saturated fat, long-term multivitamin use, alcohol consumption; higher AHEI scores represent healthier diet | | **Outcomes:**  >5 outcomes ascertained from 3 follow-up screenings in 1997-99, 2002-04, 2008-09: ideal aging, nonfatal cardiovascular disease at follow-up, cardiovascular death, noncardiovascular death, natural or normal aging *records from national health registers (e.g. national cancer registry), self-reported questionnaires and medical records used for case ascertainment  *deaths identified through National Health Services Central Registry  >ideal aging at age 60 and older defined as: *being alive *absence of chronic diseases, such as CHD, stroke, cancer (identified through cancer registry), diabetes (identified through self-reported doctor diagnosis, use of anti-diabetic medication, oral glucose tolerance test) *absence of mental health problems (>42 in mental health scale of the Short Form General Health Survey) *Good cardiometabolic functioning (based on systolic blood pressure and fasting glucose), respiratory functioning (forced expiratory volume at phase 9), musculoskeletal (walking speed), and cognitive functioning (5 cognitive tests at phase 9) | | **Analysis:**  *principal component analysis of the 127 food-frequency questionnaire items was performed and two dietary patterns were derived: 'healthy foods patterns' (had high loadings for intake of vegetables, fruit, fish) and the 'Western-type diet' (high loadings for items such as fried food, processed and red meat, pies, etc.); for each dietary pattern: factor scores were divided into tertiles and participants were categorized into the appropriate tertile based on their score *logistic regression was used to assess the association between dietary variables and each dichotomous aging outcome  **Confounders:**  *age, sex, total energy intake, smoking, physical activity | | **Results:**  *4% of participants met ideal aging definition, 12.7% developed a nonfatal cardiovascular disease, 2.8% died from cardiovascular disease, 7.3% died from noncardiovascular causes over 16 year follow-up; 73.2% showed natural aging *the odds for ideal aging were lower for participants in the top tertile of the Western-type diet (OR=0.58, [0.36, 0.93]) compared to the bottom tertile *high adherence to the AEHI recommendations was associated with lower odds of CVD and non-CVD deaths (OR=0.60, [0.39, 0.92]; OR=0.75, [0.57, 0.98], respectively)  **Limitations:**  *possibly lack of statistical power *generalizability issues as participants are mainly white, office-based civil servants *somewhat imprecise method of assessing dietary intake using semi-quantitative food-frequency questionnaire *residual confounding  **Source of Funding:**  *none reported | |
| **Authors**: Alonso A, Mosley TH Jr, Gottesman RF, Catellier D, Sharrett AR, et al. **Year:** 2009 **Citation:** J Neurol Neurosurg Psychiatry. 2009;80(11):1194-1201 **Country of study:** US **Aim:** To study the association between cardiovascular risk factors and incidence for dementia among Caucasians and African American people **Study design:** Longitudinal **Quality score:** +  **Applicability:** | | | | | | | | | | | | | |
| **Source population:**  *population-based cohort of 15,792 participants ages 45-64 recruited in 1987-9 from Forsyth County, North Carolina; Jackson, Mississippi; Washington County, Maryland; suburbs of Minneapolis, Minnesota in United States for Atherosclerosis Risk in Communities (ARIC) study *participants were examined at baseline in 1987-9 and every three years until 1996-1998 | | **Study population:**  *analysis restricted to white individuals from Minnesota, Washington County and Forsyth County communities and African Americans from Jackson and Forsyth County (**n=11,151**) *response rate: 86 %  *Follow-up:* *Follow-up from 1990-92 to occurrence of hospitalisation with dementia, death, loss to follow-up, or 31 December 2004, whichever occurred earlier  **Exclusion:**  -  **Attrition:**  *no details provided | | | | **Exposures:**  *lifestyles (e.g. smoking) assessed in 1990-92 | | **Outcomes:**  *incident dementia identified through participant or proxy report and chart abstraction of hospital discharge codes between 1990-92 and Dec. 31 2004 *dementia cases ascertained at annual follow-up of participants  *cognitive function assessed via interviews using 3 neuropsychological tests measuring memory, sustained attention and psychomotor speed, flexibility of verbal thought processes | | **Analysis:**  *Cox proportional hazard models to estimate hazard ratios of dementia by presence of cardiovascular risk factors at baseline   **Confounders:**  *sex, race, educational level, occupation, study centre, scores in cognitive assessment at baseline, presence of cardiovascular factors (hypercholesterolemia, BMI, hypertension, diabetes), APOE 4 -age assessed as confounder and as effect modifier | | **Results:**  *203 dementia cases identified during 142,625 person-years of follow-up *current smokers were more likely to develop dementia compared to those who had never smoked [HR=1.7, (1.2, 2.5)]; no differences by race, sex, or APOE4 genotype categories (when baseline cognitive scores were not controlled for) *stratification by age at examination: among those <60 years of age, current smokers were more likely to develop dementia than those who had never smoked [HR=2.2, (1.2, 4.1)]  **Limitations:**  *hospital discharge diagnoses used to ascertain dementia cases likely underestimate disease burden; dementia may be undetected in subgroups with high prevalence of comorbidities (e.g. smokers) *subgroups (e.g. smokers) have higher risk of hospitalisation, therefore dementia more likely to be detected in these groups  **Source of Funding:**  *National Heart, Lung and Blood Institute | |
| **Authors**: Anttila T, Helkala EL, Viitanen M, Kåreholt I, Fratiglioni L, et al. **Year:** 2004 **Citation:** BMJ. 2004;329(7465):539 **Country of study:** Finland **Aim:** Association between midlife alcohol consumption and subsequent mild cognitive impairment and dementia in old age **Study design:** Longitudinal **Quality score:** +  **Applicability:** | | | | | | | | | | | | | |
| **Source population:**  *participants selected from eastern Finland during 1971-1987 as part of Cardiovascular Risk Factors, Aging and Dementia (CAIDE) Study  *study response rates between 82% and 90% | | **Study population:**  ***1018** out of 1464 people (70%) ages 65-79 years invited for re-examination in 1998 participated *632 women and 386 men with mean age of 48.3 years at midlife examination in 1972/1977, and 71.7 years at follow-up examination in 1998  *Follow-up:* *follow-up from 1972-77 to 1998  **Exclusion:**  -  **Attrition:**  *Non-participants at the follow up visit in 1998 were, at the midlife assessment, older than the participants; had spent less time in education, and had dementia in old age more often than the participants  *40 cases of dementia did not participate | | | | **Exposures:**  *frequency of alcohol consumption assessed using self-administered questionnaire administered at midlife in 1972 and 1977, as well as in the follow-up examination in 1998 *frequency of alcohol consumption categorized as: never drank, drank infrequently (less than once a month), drank frequently (several times a month) | | **Outcomes:**  *cognitive function assessed in 1998 using MMSE, with scores <=24 on MMSE selected for further examination *mild cognitive impairment diagnosed according to Mayo Clinic Alzheimer Disease Research Center; diagnostic criteria; dementia diagnosis based on DSM-IV, Alzheimer’s disease diagnosed according to National Institute of Neurological and Communicative Disorders and Stroke and the Alzheimer’s Disease and Related Disorders Association | | **Analysis:**  *logistic regression used to investigate association between midlife alcohol consumption and subsequent mild cognitive impairment and dementia *effect modification by APOE4 assessed  **Confounders:**  *age, sex, education, midlife BMI, total cholesterol concentration, smoking status, follow up time, midlife systolic and diastolic blood pressure, history of myocardial infarction and stroke at follow up | | **Results:**  *61 (5.8%) participants had mild cognitive impairment, 48 (4.6%) had dementia of whom 37 (77%) had Alzheimer’s disease *the odds for mild cognitive impairment were higher for those who never drank and those who drank frequently compared to infrequent drinkers (OR=2.15 [1.01, 4.59] and OR=2.57 [1.19, 5.52], respectively) *among carriers of the APOE4, the risk of dementia was greater for frequent drinkers compared to non-drinkers (OR=7.07, [1.37, 36.60]) *e4 carriers who drank infrequently and e4 carriers who drank frequently were 2.3 and 3.6 times more likely to develop dementia, respectively, in comparison with participants who never drank and did not carry e4; similar results were observed when dementia and mild cognitive impairment were assessed together as one outcome  *sex stratification: an increased risk of mild cognitive impairment was observed for frequent male drinkers compared to infrequent male drinkers [OR=5.03, p=0.02]  **Limitations:**  *recall bias with respect to self-reported alcohol consumption  *selective survival related to APOE4 (heavy drinkers may be more likely to develop vascular morbidity, and presence of APOE4 can increase mortality) – this can underestimate relationship between alcohol drinking and dementia  **Source of Funding:** *Aging Program of the Academy of Finland, EVO-grants of Kuopio University Hospital and Academy of Finland grants (Insamligsstiftelsen för Alzheimer- och Demensforskning), and the Gamla Tjänarinnor Foundation | |
| **Authors**: Ascherio A, Zhang SM, Hernán MA, Kawachi I, Colditz GA, et al. **Year:** 2001 **Citation:** Ann Neurol. 2001;50(1):56-63 **Country of study:** US **Aim:** To examine the relationship of coffee and caffeine consumption to the risk of Parkinson’s disease among health professionals and nurses **Study design:** Longitudinal **Quality score:** ++  **Applicability:** | | | | | | | | | | | | | |
| **Source population:**  *51,529 male health professionals (mostly white, of European ancestry), ages 40-75 years, were recruited in 1986 to participate in HPFS study  *121,700 female nurses ages 30-55 years, living in 11 states (mostly white, of European ancestry), recruited in 1976 to participate in NHS study  *follow-up every 2 years | | **Study population:**  *avg. follow-up of 9.2 years for **47,351** men and 15.5 years for **88,565** women  *Follow-up:* *< 3% loss to follow-up for men and < 2% for women *follow-up for deaths more than 98% complete *follow-up: from 1986 (for men part of the HPFS) or 1980 (for women part of the NHS) to the occurrence of Parkinson’s, death, or end of follow-up in June 1996   **Exclusion:**  *participants diagnosed with Parkinson’s disease, stroke, or cancer before they answered baseline questionnaire  *men and women with extreme daily caloric intakes or incomplete food-frequency questionnaires at baseline  **Attrition:**  - | | | | **Exposures:**  *caffeine intake and dietary information assessed every 2-4 years using semi-quantitative food-frequency questionnaire (SFFQ); first administered in 1980 (NHS); assesses consumption of coffee, tea, chocolate, decaffeinated coffee, soft drinks with or without caffeine  *questionnaires assessed consumption (of 1 cup of coffee, 1 cup of tea, 1 glass of soft drink, 1 ounce of chocolate) during previous 12 months and allowed for 9 response categories ranging from never to 6 or more per day *intakes of nutrients and caffeine calculated based on US Department of Agriculture assuming that caffeine content was 137 mg per cup of coffee, 47 mg per cup of tea, 46 mg per can or bottle of cola, 7 mg per serving of chocolate candy *reproducibility and validity of SFFQ evaluated within the NHS and HPFS; has good validity and reproducibility (when self-reported coffee intake was compared with dietary records, correlation coefficient was 0.78-0.93) *for women, 1980 SFFQ and slightly more refined 1984 version was used to calculate caffeine consumption as well as cumulative average of caffeine consumption from all the available questionnaires prior to the beginning of each 2-year period | | **Outcomes:**  *Parkinson’s disease cases  *Measurement of outcomes:*  *lifetime occurrence of Parkinson’s disease included in 1988 (HPFS) and 1994 (NHS) questionnaires; Parkinson’s disease diagnosis within last 2 years assessed in subsequent questionnaires  *medical records and/or neurologist/internist/GP confirmed new patient self-reported diagnoses of Parkinson’s and certainty of diagnosis (definite, probable, possible)  *deaths were reported by next of kin, co-workers, postal authorities, or the National Death Index *when Parkinson’s listed as cause of death on death certificate, same process of outcome ascertainment was followed as for non-fatal cases | | **Analysis:**  *pooled logistic regression with 2-year intervals to assess the relationship between  caffeine intake from different sources and the risk of Parkinson’s disease in men and women, separately  **Confounders:**  *age, smoking, BMI, alcohol consumption, physical activity, niacin intake, use of HRT | | **Results:**  *157 cases of Parkinson’s disease in men and 131 in women (*in men and women, coffee consumption was strongly associated with smoking and weakly associated with alcohol use) *Among men, after adjustment for age and smoking, the relative risk of Parkinson’s disease was 0.42 (95% CI: 0.23–0.78; p for trend < 0.001) for participants consuming >6 cups/day compared to those consuming 0 cups/day *Men: significant inverse association observed between: coffee consumption and risk of Parkinson's (p for trend= 0.004), caffeine from non-coffee sources and risk of Parkinson's (p for trend < 0.001), as well as, tea and Parkinson's (p for trend= 0.02)  *Women: the relationship between caffeine intake and risk of Parkinson’s disease was U-shaped, with the lowest risk observed for those reporting 1–3 cups of coffee/day compared to those reporting 0 cups/day  **Limitations:**  *possible non-differential misclassification of caffeine *chance or interaction with other factors could be plausible explanations for associations observed  *participants were mostly white, of European ancestry, so limited generalizability  **Source of Funding:**  *National Institutes of Health | |
| **Authors**: Baba S, Iso H, Mannami T, Sasaki S, Okada K, et al. **Year:** 2006 **Citation:** Eur J Cardiovasc Prev Rehabil. 2006;13(2):207-213 **Country of study:** Japan **Aim:** To determine the sex-specific relationships of smoking with the risk of CHD **Study design:** Longitudinal **Quality score:** +  **Applicability:** | | | | | | | | | | | | | |
| **Source population:**  *27,063 men and 27,435 women, ages 40-59 years, and born between 1930-1949, and registered in 14 administrative districts supervised by public health centre areas on Jan. 1 1990 | | **Study population:**  ***19,794** men and **21,513** women (registered, non-institutional residents)  *Follow-up:* *88% follow-up *11 years follow-up from 1990 to 2001 (from collection of baseline questionnaire to first endpoint, death, or Jan. 1 2002)  **Exclusion:**  *participants with history of MI, angina pectoris, stroke, cancer  **Attrition:** - | | | | **Exposures:**  *self-administered lifestyle questionnaire distributed in 1990 and completed between Jan. 1990 and May 1992 *smoking and drinking habits, diet and other lifestyles, including leisure time sports and sleeping hours *smoking categorized as: never, ex, and current smoker (additional sub-categories for male ‘current smokers’: 1-14, 15-34, 35 per day or more) *drinking categorized as: never, ex, current drinkers who drink more than once a month (frequency and kinds of alcoholic beverages, as well as average quantity per day) *daily food intake: frequency of weekly intake asked for 27 food items and categorized as: rarely, 1-2 days per week, 3-4 days per week, almost every day; food items assessed included rice, miso soup, fruit, vegetables, fish | | **Outcomes:**  *acute coronary events [MI, sudden cardiac death, other fatal coronary events] that occurred between 1990 and Jan. 1 2002  *medical records reviewed from hospitals with cardiology departments *MI confirmed according to criteria of the MONICA project (electrocardiograms, cardiac enzymes, autopsy – if this work-up was not performed, probably diagnosis was made) *deaths occurring within 1 hour of symptom onset labelled as sudden cardiac deaths *death certificate also reviewed for evidence of CHD and acute heart failure [ICD-10]  *other fatal coronary events were those in which medical records could not be found for cases identified through death certificates, or coronary events identified through death certificates did not match study criteria for MI or sudden cardiac death *total MI includes both definite and probable cases | | **Analysis:**  *Cox proportional hazards model to assess the sex-specific relationships of smoking with the risk of CHD  **Confounders:**  -age, alcohol intake, history of hypertension and diabetes, treated hyperlipidemia, food intake (fruit, vegetable, fish servings), education years, public health centre | | **Results:**  461,761 person-years of follow-up:  *men: 260 CHD cases of which 174 were MI, 63 were sudden cardiac deaths, 23 were other fatal coronary events  *women: 66 CHD cases of which 43 were MI, 16 were sudden cardiac deaths, 7 were other fatal coronary events  *risk of (total) coronary heart disease and (total) myocardial infarction significantly higher in male current smokers compared to those who never smoked (RR=2.85, [1.98, 4.12] and RR=3.64, [2.27, 5.83], respectively)  *males: the risk of total coronary heart disease and total myocardial infarction increased with the number of cigarettes smoked per day (trend test p-values: <0.001 and <0.001, respectively) *women: the risk of (total) coronary heart disease and (total) myocardial infarction greater for current smokers compared to never smokers (RR=3.07, [1.48, 6.40], RR=2.90, (1.18, 7.18), respectively); the risk of (total) myocardial infarction was also greater for past smokers compared to never smokers (RR=3.72, [1.10, 12.6]) *population-attributable risk percent (95% CI) of CHD was 46% (34, 55) in men and 9% (0, 18) in women  **Limitations:**  *none reported  **Source of Funding:**  *none reported | |
| **Authors**: Beulens JW, de Bruijne LM, Stolk RP, Peeters PH, Bots ML, et al. **Year:** 2007 **Citation:** J Am Coll Cardiol. 2007;50(1):14-21 **Country of study:** The Netherlands **Aim:** Explore the association between dietary glycemic load and glycemic index with CVD; assess whether this association is modified by BMI **Study design:** Longitudinal **Quality score:** ++  **Applicability:** | | | | | | | | | | | | | |
| **Source population:**  *17,357 women (breast cancer screening participants part of the Prospect-European Prospective Investigation into Cancer and Nutrition [EPIC] cohort) ages 49-70 recruited between 1993-1997 | | **Study population:**  *10% random sample drawn from 15,714 women of original study, exclusion criteria applied to yield final cohort of **1,417** Dutch women *time period: 1993-1997 to Jan. 1, 2005  *Follow-up:* *follow-up from date of return of exposure assessment questionnaire until date of outcome of interest (CHD or CVA), participant date of death, or Jan. 1 2005  **Exclusion:**  *women who did not consent to linkage with vital status registries *women with missing questionnaires, who reported an energy intake of <500 kcal/day or >6000 kcal/day *women with history of CHD or cerebrovascular disease before baseline, or with established diabetes *study censoring: mortality due to noncardiovascular causes (n=549), loss to follow-up due to emigration (n=60) and withdrawn alive (14,306)  **Attrition:**  - | | | | **Exposures:**  *average exposure to glycemic index and glycemic load in the previous year (before completing questionnaire) *Food frequency questionnaire (validated – Spearman correlations between 0.56-0.78) used to assess average daily consumption of 178 foods; food glycemic index obtained  *glycemic load obtained by multiplication of glycemic index with carbohydrate content of food item and with frequency of consumption of food item – values over all food items summed *unit of dietary glycemic load is 1g carbohydrate from glucose *glycemic index (per gram of carbohydrate): glycemic load divided by total carbohydrate consumed | | **Outcomes:**  *Outcomes of interest: cardiovascular disease (coronary heart disease (CHD), cerebrovascular accidents (CVA), cardiovascular disease (CVD)) *hospital discharge diagnoses (ICD-9 codes) obtained from the Dutch Centre for Health Care Information register *follow-up until Jan. 1, 2005 *vital status information obtained from municipal administration registries; cause of death obtained from GPs | | **Analysis:**  *Cox regression to estimate hazard ratios  **Confounders:**  *age, hypertension, cholesterolemia, smoking, BMI, mean systolic blood pressure, total physical activity, menopausal status, HRT, oral contraceptive use, alcohol intake, total energy intake, energy-adjusted intake of vitamin E, protein, dietary fiber, folate, energy-adjusted intake of saturated fat, poly- and monounsaturated fat | | **Results:**  *during 141,633 person-years of follow-up: 556 incident cases of fatal or nonfatal CHD and 243 incident cases of fata or nonfatal CVA *the HR between the highest and lowest quartile of glycemic load was 1.47 (HR=1.47 [1.04, 2.09]) *the higher the quartile of energy-adjusted glycemic load, the greater the risk for cardiovascular disease (p-value for trend: 0.033) *the HR between the highest and lowest quartile of glycemic index was 1.33 (HR=1.33 [1.07, 1.67]) *the higher the quartile of energy-adjusted glycemic index, the greater the risk for cardiovascular disease (p-value for trend: 0.02) *among women with high BMI (>25 kg/m2), there was an increased risk of CHD for both higher levels of glycemic load and glycemic index (p-values for trend: 0.04, 0.06, respectively)  **Limitations:**  *residual confounding by unknown risk factors *misclassification of dietary exposure  **Source of Funding:**  *none reported | |
| **Authors**: Beulens JW, Rimm EB, Hu FB, Hendriks HF, Mukamal KJ **Year:** 2008 **Citation:** Diabetes Care. 2008;31(10):2050-2055 **Country of study:** US **Aim:** To determine whether the association between alcohol consumption and diabetes development is mediated by adiponectin concentrations and biomarkers of inflammation, endothelial dysfunction, and insulin resistance **Study design:** Nested case-control study **Quality score:** +  **Applicability:** | | | | | | | | | | | | | |
| **Source population:**  *121,700 female nurses aged 30-55 years initially took part in Nurses’ Health Study in 1976 | | **Study population:**  ***705** women free of diabetes in 1989-90 and with a confirmed diagnosis of type 2 diabetes by year 2000 constituted the cases;  *cases matched to two controls on the basis of year of birth, date of blood draw, race, and fasting status at blood draw (one of the two controls was additionally matched to the case on the basis of BMI) **787** controls matched to **705** cases  *Follow-up:* *1990-2000  **Exclusion:**  *women with missing information for alcohol consumption and markers of inflammation and endothelial dysfunction *women providing blood in 1989-90 were free of diagnosed diabetes, coronary heart disease, stroke, or cancer at baseline  **Attrition:**  *participants had a higher prevalence of obesity and family history of diabetes and a lower prevalence of current smoking than non-participants (those who did not provide blood) | | | | **Exposures:**  >self-reported alcohol intake >1990 semiquantitative food frequency questionnaire used to assess alcohol intake (among women who provided blood in 1989-90) *standard portion defined as a glass, bottle, or can of beer; 4-ounce glass of wine; shot of liquor *participant’s average consumption over past year multiplied by alcohol content of portion size (12.8g for beer, 11g for wine, and 14g for liquor) and then summing across beverages *high validity of alcohol consumption (Spearman correlation coefficient: 0.90) >biennial self-administered questionnaire | | **Outcomes:**  *type 2 diabetes *diabetes self-reported and confirmed through validated supplementary questionnaire detailing symptoms, diagnostic laboratory test results, and treatment *validity of self-reported diabetes confirmed through medical record review in 62 participants | | **Analysis:**  *logistic regression was used to determine if adiponectin concentrations and biomarkers of inflammation, endothelial dysfunction, and insulin resistance mediate the association between alcohol consumption and diabetes  **Confounders:**  *BMI, physical activity, smoking, family history of diabetes, postmenopausal HRT, energy intake, energy-adjusted intake of saturated fat, trans fatty acids, polyunsaturated fat, dietary fiber, glycemic load, coffee consumption | | **Results:**  *By year 2000, 714 women had type 2 diabetes diagnosis *the odds for type 2 diabetes were significantly lower for those who consumed alcohol with an OR of 0.67 (OR=0.67, [0.56-0.79]) per 12.5 g increment of alcohol intake (p<0.001) *25% of the association between alcohol intake and type 2 diabetes development was explained by adiponectin  **Limitations:**  *possible selection bias through use of slightly different subgroups for each group of biomarkers *only included women who provided blood samples (these women had higher prevalence of obesity and family history of diabetes in comparison with women who did not provide blood) – may limit generalizability to women with lower diabetes risk *more robust markers of insulin sensitivity may be needed  **Source of Funding:**  National Institutes of Health grants, a travel grant from the Dutch Heart Association, and a research exchange award from European Research Advisory Board | |
| **Authors**: Blanco-Cedres L, Daviglus ML, Garside DB, Liu K, Pirzada A, et al. **Year:** 2002 **Citation:** Am J Epidemiol. 2002;155(4):354-360 **Country of study:** United States **Aim:** To determine the association between smoking and cardiovascular disease (CVD), coronary heart disease (CHD), and all-cause mortality among men with various levels of serum total cholestero**l** **Study design:** Longitudinal **Quality score:** +  **Applicability:** | | | | | | | | | | | | | |
| **Source population:**  *39,572 out of 70,000 men and women ages 18 years and older, employed by 84 Chicago-area companies agreed to participate in Chicago Heart Association Detection Project in Industry (CHA) study (55% response rate) | | **Study population:**  *this study included **8,816** men aged 40-59 years at baseline  *Sociodemographics:* *study sample of men had mean age of 48.5 years, 5.3% were African Americans  *Follow-up:* *25 years follow-up with screening of men between 1967-1973  **Exclusion:**  *men and women 18-39 years and women 40-59 years at baseline *of the men aged 40-59 years screened at baseline: excluded if they had missing data at baseline or follow-up (n=451), baseline evidence of prior myocardial infarction (n=62), previous diagnosis of diabetes mellitus (n=169)  **Attrition:**  - | | | | **Exposures:**  *past and present smoking status ascertained by self-reported questionnaire | | **Outcomes:**  *CHD death, CVD death, all-cause mortality *deaths ascertained from Social Security Administration and National Death Index records | | **Analysis:**  *Cox multivariate proportional hazards regression used to assess influence of baseline current smoking on cause-specific and all-cause mortality for men within four strata of serum cholesterol: <180, 180-199, 200-239, >=240  **Confounders:**  *age, race, education, BMI, systolic blood pressure, presence of ECG abnormalities | | **Results:**  *of 8,816 men, 32% and 45.7% of men died of CHD and CVD, respectively *the relative risk for CHD death was greater for smokers compared to non-smokers and ranged from 1.50 (RR=1.50; [1.17, 1.88]) to 2.18 (RR=2.18, [1.54, 3.08]) across cholesterol levels *the relative risk for CVD death was greater for smokers than non-smokers and ranged from 1.58 (RR=1.58, [1.17, 2.14]) to 1.95 (RR=1.95, [1.48, 2.57]) across cholesterol levels *the relative risk for all-cause mortality greater for smokers compared to non-smokers and ranged from 1.78 (RR=1.78, [1.54, 2.07]) to 2.19 (RR=2.19, [1.84, 2.61]) across cholesterol levels *interactions between current smoking and cholesterol level were not significant for CHD, CVD, and all-cause mortality  **Limitations:**  *regression dilution bias (potentially underestimated results due to misclassification of cholesterol measurement)  **Source of Funding:**  *American Heart Association and its Chicago and Illinois affiliates; the Illinois Regional Medical Program; the National Heart, Lung, and Blood Institute; the Chicago Health Research Foundation; and private donors | |
| **Authors**: Boudík F, Reissigová J, Hrach K, Tomecková M, Bultas J, et al. **Year:** 2006 **Citation:** Atherosclerosis. 2006;184(1):86-93 **Country of study:** **Aim:** To evaluate the relationship between health risk factors and atherosclerotic CVD death **Study design:** Longitudinal **Quality score:** -  **Applicability:** | | | | | | | | | | | | | |
| **Source population:**  *50% of 2370 middle-aged men living in Prague identified through electoral register | | **Study population:**  *1390 out of 2370 men responded to and underwent screening examination in 1975-78 *mean age at study entry was 46.1 years *analysis restricted to **926** men  *Follow-up:* *1979 to 1999-01  **Exclusion:**  *diabetic patients at baseline  **Attrition:**  - | | | | **Exposures:**  *smoking was assessed through self-administered questionnaire in 1975-79 *smoking categories: >=15 cigarettes daily; non-smokers; ex-smokers for less than a year and who previously smoked >=15 cigarettes per day | | **Outcomes:**  *atherosclerotic cardiovascular disease (CVD) mortality ascertained in 1999-2001 *data on atherosclerotic CVD mortality and survival ascertained from outpatient departments, postal questionnaires, and registry offices (Institute of Health Information and Statistics of the Czech Republic) | | **Analysis:**  >cox proportional hazards model used to assess influence of smoking on CVD mortality among ‘risk group men’ *risk group men defined as those with one or more atherosclerosis risk factors and without apparent atherosclerotic CVD, diabetes mellitus, and other serious disease at baseline  **Confounders:**  *age, education, blood pressure, total cholesterol | | **Results:**  *the hazard rate of death from atherosclerotic CVD was 3 for participants reporting >=15 cigarettes daily compared to those reporting <15 cigarettes daily (HR=3, [2.0, 4.6])  **Limitations:**  *lack of a true control group (ethical reasons) and risk factor profile of participants may have varied from that of general population   **Source of Funding:**  *Ministry of Education of the Czech Republic | |
| **Authors**: Britton A, Shipley M, Singh-Manoux A, Marmot MG **Year:** 2008 **Citation:** J Am Geriatr Soc. 2008;56(6):1098-105 **Country of study:** England **Aim:** The influence of early- and midlife predictors on successful aging **Study design:** Longitudinal **Quality score:** +  **Applicability:** | | | | | | | | | | | | | |
| **Source population:**  *10,308 civil servants (6,895 men and 3,413 women) ages 35-55 in 20 London-based departments in England eligible for Phase 1 (1985-1988) of Whitehall II study *73% response rate | | **Study population:**  *analysis restricted to **5,823** participants (4,140 men and 1,683 women) ages 35-55, free of disease at Phase 1 and who had attended at least 5 phases of follow-up/data collection until Phase 7 (2002-2004), and with measures of functioning at Phase 7   *Follow-up:* *follow-up from Phase 1 to Phase 7  *535 people died during follow-up *mortality was greater in those of lower social position  **Exclusion:**  *those with prevalent disease at Phase 1, attended fewer than 5 phases of follow-up, did not attend Phase 7, had missing data on functioning at Phase 7, had unknown metabolic syndrome during study period *4,485 people excluded from the analyses; they tended to be older (mean age at Phase 1 45.1 vs 43.9 years), were more often female (39% vs 29%), and were from the lowest socio-economic position groups (33% vs 15%) than those included  **Attrition:**  - | | | | **Exposures:**  *smoking: never-smoker, ex-smoker, current smoker  *alcohol: 0, 1-14, 15 units/week with 1unit=8g ethanol *poor diet (yes/no): summary index of poor diet was defined if two or three of the following applied: most frequently used bread was white, consumption of whole milk, fruit or vegetables eaten less often than daily  *physical activity: frequency and number of hours per week spent on activities: grouped as vigorous or moderate (performed 1 or more hours per week of these); none or mild *self-reported questionnaire used to ascertain exposures | | **Outcomes:**  >successful aging: free from major disease (coronary heart disease, stroke, cancer, diabetes mellitus, depression, metabolic syndrome) from Phase 1 up to Phase 7 and with good physical and mental functioning at Phase 7 *physical and mental functioning based on  walking speed, lung function, Alice Heim 4-I cognitive test, physical component score of the 36-item Short Form General Health Survey >self-reported questionnaires, medication use, clinical examinations, evidence from GPs and hospitals used to ascertain outcome | | **Analysis:**  *logistic regression used to assess association between health behaviours (smoking, alcohol, diet, exercise) at Phase 1 and successful aging at Phase 7 for men and women, separately  **Confounders:**  *age at Phase 1, number of phases attended, SES, early-life factors (father’s social class, education, height), psychosocial factors, job demands, work support, network index | | **Results:**  *548/4,140 men and 246/1,683 women were aging successfully by Phase 7  >the odds of successful aging were higher in:  *non-smokers compared to current smokers for men and women [OR= 2.7, (1.8, 4.1), OR=2.2 (1.3, 3.7), respectively];  *men who did not have a poor diet compared to those who did [OR=1.4, (1.1,1.7)];  *men and women with higher levels of physical activity [OR=1.9, (1.2, 3.1); OR=1.7 (1.1, 2.6), respectively];   >the odds of successful aging were lower for women who did not drink alcohol versus those who had 15 units/week [OR=0.5 (0.3, 0.9)]  >an increasing trend in the odds of successful aging occurred with: *less exposure to cigarette smoking for men (p<0.001) and women (p=0.006); *greater levels of physical activity for men (p<0.001) and women (p=0.03);  *fewer units of alcohol consumed per week for women (p=0.01)  **Limitations:**  *potentially imprecise definition of successful aging *limited generalizability as study consisted of relatively homogeneous group (London-based office workers) *ethnicity not examined  **Source of Funding:**  *British Medical Research Council; British Economic and Social Research Council; British Heart Foundation; UK Health and Safety Executive; UK Department of Health; National Heart Lung and Blood Institute, US National Institute of Health, National Institute on Aging, US National Institutes of Health, Agency for Health Care Policy Research; and the John D. and Catherine T. MacArthur Foundation Research Networks on Successful Midlife Development and Socioeconomic Status and Health | |
| **Authors**: Carlson MC, Helms MJ, Steffens DC, Burke JR, Potter GG, et al. **Year:** 2008 **Citation:** Alzheimers Dement. 2008;4(5):324-331 **Country of study:** United States **Aim:** To determine whether midlife cognitive and physical leisure activities are associated with delayed onset or reduced risk of dementia within older male twin pairs (World War II veterans)  **Study design:** Longitudinal **Quality score:** ++  **Applicability:** | | | | | | | | | | | | | |
| **Source population**  *15,942 white male twin pairs born 1917-1927 in 42 US states made up the NAS-NRC Twin registry of male World War II veterans  *registry created through linkage of birth certificates with files of Department of Veterans Affairs | | **Study population:**  *exposure assessment questionnaire administered to twins at around 45 years of age (84% response rate [n=7400 twin pairs]) *analysis restricted to **147 twin pairs** (at least one twin received a dementia diagnosis and the other twin remained non-demented for at least 3 years after the onset of dementia in the first twin)  *Participants more educated than non-participants   **Follow-up:* 1967 to 1990-2005  **Exclusion:**  -  **Attrition:**  - | | | | **Exposures:**  *4 physical exercise and leisure activities assessed using 1967 self-reported questionnaire that focused on: outdoor activities; sports; gardening and home improvement; physical exercise after age 35 *for each participant, the number of activities was tallied to yield maximum activity score of four | | **Outcomes:**  *Dementia assessment and cognitive screening conducted from 1990 to 2005  *50-point Telephone Interview for Cognitive Status-modified (TICS-m) used for cognitive screening  *When participants could not complete phone interview, proxy was interviewed using Informant Questionnaire on Cognitive Decline in the Elderly or another interview whereby a physician or psychologist reviewed participant's answers *If suspected impairment on TICS-m or proxy instruments, Dementia Questionnaire (DQ) administered *If possible dementia identified through DQ, participant underwent neurologic examination, neuropsychological testing, blood or buccal DNA collection, history of cognitive symptoms and medical history assessed | | **Analysis:**  *Dependent proportional hazard Cox ratios used to model the elapsed time from date of leisure activity assessment to either dementia diagnosis or censoring age   **Confounders:**  *Occupational history, age at date of activity assessment, education, natural matching (e.g., genes)  Effect modification: APOE4 allele | | **Results:**  *dementia mean age of onset: 72.7 years  *Physical activity did not predict dementia risk reduction *Risk for dementia by physical activity scores in discordant twin-pairs: OR=0.99 (0.73-1.33) *Risk for dementia among monozygotic twin pairs with and without APOE 4 allele [OR=0.82, (0.48-1.41) and OR=0.94 (0.48-1.87), respectively]  **Limitations:**  *Possibly underpowered study and restricted measurement sensitivity   **Source of Funding:**  *National Institute on Aging | |
| **Authors**: Chang M, Jonsson PV, Snaedal J, Bjornsson S, Saczynski JS, et al. **Year:** 2010 **Citation:** J Gerontol A Biol Sci Med Sci. 2010;65(12):1369-1374 **Country of study:** Iceland **Aim:** To evaluate the association between mid-life physical activity and late-life cognitive performance and dementia  **Study design:** Longitudinal **Quality score:** -  **Applicability:** | | | | | | | | | | | | | |
| **Source population:**  *men and women born in 1907-1935 and living in Reykjavik, Iceland part of Reykjavik Study that was initiated in 1967 | | **Study population:**  *in 2002, cohort members re-invited to participate  ***4,761** participants (2,006 women and 2,755 men)   *Sociodemographics:* *mean age of 51 years of participants at midlife examination and 76 years at life-life  *Follow-up:* *on average, 26 years elapsed between mid-life and late-life examinations   **Exclusion:**  *participants with APOE e2/4 (n=32)  *people with missing data from cognitive performance tests (n=819)  *184 people with prevalent dementia at baseline  **Attrition:**  - | | | | **Exposures:**  >midlife physical activity assessed through interview  *participants who had ever participated in sports or exercise during adults life then reported the number of hours per week of exercise during winter and summer time and were subsequently categorized as follows: none (0 hours/week); <=5 hours/week; >5 hours/week | | **Outcomes:**  *cognitive function assessed using cognitive tests measuring speed of processing, memory, executive function *DSM-IV dementia diagnosed according to 3 step protocol; those with low scores on MMSE or digit symbol substitution test were administered second diagnostic cognitive test battery; last step involved neurological test and a proxy interview regarding medical history, social, cognitive and daily functioning changes of participant | | **Analysis:**  *logistic regression used to assess midlife physical activity on late-life dementia  **Confounders:**  *demographic and health factors, including age at time of examination, blood pressure, BMI, serum cholesterol, smoking status, resting heart rate, depressive symptoms  *Stratification by APOE: APOE allele genotyping on subsample of 2,113; participants categorized as carriers vs. non-carriers | | **Results:**  *those who exercised <=5 hours/week and >5 hours/week at midlife had significantly faster speed of processing (p<0.0001), better memory (p<0.0001), and higher executive function (p<0.0001) compared to those who never exercised at midlife *the odds of dementia were lower among those who exercised <=5 hours/week compared to those who never exercised (OR=0.59, [0.40, 0.88])  Stratification by APOE4: *APOE e4 non-carriers who reported midlife physical activity had very low risk for late-life dementia compared to those who reported no midlife physical activity and were APOE e4 carriers (OR=0.18, [0.07, 0.45])  *APOE e4 non-carriers who reported no midlife activity also had reduced risk for dementia (OR=0.59, [0.36, 0.98])  **Limitations:**  *limited detail by which physical activity is characterized  **Source of Funding:**  *National Institutes of Health contract N01-AG-12100, the National Institute on Aging Intramural Research Program, the Icelandic Heart Association, the Icelandic Parliament, and the Icelandic Center for Research | |
| **Authors**: Chang M, Saczynski JS, Snaedal J, Bjornsson S, Einarsson B, et al. **Year:** 2013 **Citation:** J Am Geriatr Soc. 2013;61(2):237-242 **Country of study:** Iceland **Aim:** To evaluate the association between mid-life physical activity and late-life lower extremity function (LEF) in older adults **Study design:** Longitudinal **Quality score:** -  **Applicability:** | | | | | | | | | | | | | |
| **Source population:**  *men and women born in 1907-1935 and living in Reykjavik, Iceland part of Reykjavik Study that was initiated in 1967 | | **Study population:**  >in 2002, cohort members re-invited to participate  ***4,753** participants included in primary analysis of physical activity (PA) on LEF ***4,359** participants with complete cognitive data selected for secondary analysis of PA on LEF, adjusting for cognitive function   *Follow-up:* *average of 25 years  *Sociodemographics:* *2,011 men and 2,742 women included in primary analysis  **Exclusion:**  >out of 5,764, people excluded from primary analysis because of: *missing data on LEF tests (n=797) *had diagnosed dementia (n=214) -those excluded were older, more sick than participants  **Attrition:**  - | | | | **Exposures:**  >midlife physical activity was assessed through interviews on the number of hours per week that people participated in sports or exercise in the summer and winter; participants were categorized as:  *active if they reported any physical activity hours in summer or winter, or *inactive, if they reported no physical activity | | **Outcomes:**  *lower extremity function (LEF) was measured in late life using reliable gait speed test, walk test (TUG test for assessing balance problems in older people and ADL decline), and knee extension strength (KES) | | **Analysis:**  **primary analysis:* linear regression used to assess influence of midlife PA on gait speed, TUG, and KES tests while adjusting for all confounders, except cognitive function **secondary analysis:* same as above, with additional adjustment for cognitive function  **Confounders:**  *mid-life variables including education, blood pressure, height, weight, serum cholesterol, smoking, serum cholesterol *late-life variables including diabetes mellitus, coronary events (history of myocardial infarction, coronary bypass surgery, heart bypass surgery, angioplasty, or others), stroke, depression, MMSE (only in primary analysis) or cognitive function (only in secondary analysis) | | **Results:**  *those who were active at midlife had significantly better LEF **primary analysis:* the active group had 0.049-m/s faster gait speed (95% CI = 0.038, 0.059, P < .001), completed the TUG test 0.53 seconds faster (95% CI =0.71, 0.36, P < .001), and had 1.34-kg greater KES (95% CI = 0.83, 1.86, P < 0.001) than the inactive group **secondary analysis:* the active group had 0.037-m/s faster gait speed (95% CI = 0.026, 0.048, P < .001), completed the TUG test 0.34 seconds faster (95% CI = 0.52, 0.16, P < .001), and had 0.87-kg greater KES (95% CI = 0.34, 1.42, P < .001) than the inactive group  **Limitations:**  *none reported by the study authors  **Source of Funding:**  *Intramural Research Program of the National Institutes of Health, the National Institute on Aging, the Icelandic Heart Association, Landspitali University Hospital, and the Icelandic Parliament | |
| **Authors**: Christensen U, Støvring N, Schultz-Larsen K, Schroll M, Avlund K **Year:** 2006 **Citation:** Scand J Med Sci Sports. 2006;16(4):245-251 **Country of study:** Denmark **Aim:** Determine the influence of physical inactivity from middle-age to early old-age on disability at age 75 **Study design:** Longitudinal **Quality score:** -  **Applicability:** | | | | | | | | | | | | | |
| **Source population:**  *cohort follow-up of 802 people selected for baseline study in 1964 from Copenhagen, Denmark (participation rate 84%) | | **Study population:**  ***387** people born in 1914 who participated in at least one of the three study phases in 1964, 1974, or 1984 and at the 25-year follow-up in 1989  *Sociodemographics:* *study had more women (54%) than men, 76% of participants had 7 years of school education and 84% were married or cohabiting  *Follow-up:* *25 years  **Exclusion:**  *people who died, moved, or refused to participate (75% participation rate, n=666; and 74% participation rate, n=537 at 10-year and 20-year follow-up, respectively)  *at 20- and 25-year follow-up, non-participants were more likely to be men, have 7 years of basic school education, to be cohabiting and smoke in comparison with participants  **Attrition:**  - | | | | **Exposures:**  >self-reported physical activity in leisure time assessed in 1964, 1974, and 1984  *participants grouped as ‘mainly sedentary’ or ‘mainly active’ (e.g. light physical activity 1-4 h/week or moderate activity 1-3 h/week or vigorous activity more than 4h/week or in competitive sports several times/week) | | **Outcomes:**  >functional ability assessed by interviewer-administered Mob-T scale that measures tiredness after performing mobility activities with scores ranging from 0 to 6 *categorized participants into those with good function (score=6) and those with poorer function (score<6) *cumulative physical activity measures formed for ages of 50+60, 60+70, and 50+60+70 – indicates average physical activity value during that time period *construct validity, criterion-related validity and reliability of Mob-T scale described in different study | | **Analysis:**  *logistic regression used to assess influence of physical inactivity (accumulated and separately at each point in time) on disability   **Confounders:**  Smoking, household composition at baseline  Stratification: by education | | **Results:**  *physical inactivity at age 60 to 70 was related to disability for the sub-group with more than 7 years of education (OR=8.62, [1.08, 68.54])  **Limitations:**  *“healthy participant effect” *if people who dropped out of study were more likely to be physically inactive, then effects of inactivity on functional ability may have been underestimated *reporting bias and social desirability bias when participants self-reported sedentary lifestyle *small sample size  **Source of Funding:**  *none reported | |
| **Authors:** Debette S, Seshadri S, Beiser A, Au R, Himali J, et al.  **Year:** 2011  **Citation:** Neurology. 2011;77:461–468  **Country of study:** USA  **Aim of study:** Test the association of vascular risk factor exposure in midlife with progression of MRI markers of brain aging and measures of cognitive decline  **Study design:** Cohort  **Quality score: (++, + or -):**  **External validity score: (++, + or -):** | | | | | | | | | | | | | |
| **Number of people**  5,124  **Demographics** Only report on includes at baseline Age, y, mean±SD 54±9 Women, n (%) 718 (53.1) High school graduate, n (%) 1,319 (97.6) | | **Number of people** 1,352  **Characteristics**  Age 61 ± 9 Women, n (%) 718 (53.1) High school graduate, n (%) 1,319 (97.6)  **Location**  Framingham  **Recruitment strategy** The present study includes participants from the offspring cohort of Framingham HS | | | | **Relevant exposures** Smokering habits, educational achievement, BMI  **Time** 1991–1995  M**easurement of exposure** Body mass index (BMI) was defined as weight (kg) divided by the square of height (m). Standing waist circumference was measured at the level of the umbilicus; hip circumference at the level of the trochanter major. Waist-to-hip ratio was calculated as the ratio of waist to hip circumferences. Educational achievement was studied as a 4-class variable (no high school degree; high school degree, no college; some college; college degree). | | **Outcomes** white matter hyperintensity volume, total brain volume, and temporal horn volume of the lateral ventricles, verbal memory, visuospatial memory, and executive function.  **Outcome measurement** Brain MRI techniques, The delayed recall component of the Logical Memory subtest from the Wechsler Memory Scale provides a savings measure of retention for verbal memory. The delayed recall component of the Visual Reproductions test assesses visuospatial memory. The difference between the score on Trail-Making Tests B and A is a marker of executive function.  **Time**  Not reported | | **Analysis strategy** used multivariable linear regression to relate each vascular risk factor to continuous measures of change and multivariable logistic regression for dichotomous measures of change  secondary analysis tested whether the associations were similar when additionally adjusting for the baseline measure of the examined outcome variable. the association of hypertension and systolic blood pressure with WMHV progression was also adjusted for interim stroke. Also tested for interaction with APOE 4 carrier status.  **Confounders** All analyses were adjusted for sex, age at the first NP/MRI assessment, time interval between the risk factor assessment and the first NP/MRI assessment, and education for cognitive outcomes. For the dichotomous measure of white matter hyperintensity volume change, we also adjusted for the time interval between the first and last NP/MRI evaluation | | **Number** See cell C  **Effect estimates** **Smoking** **Estimate± SE p.** White matter hyperintensity volume -0.03±0.07 p.0.694 Total brain volume -0.15±0.07 p.0.025 Temporal horn volume -0.19±0.07 p.0.008 Logical Memory, delayed recall -0.08±0.08 p.0.316 Delayed recall component of the Visual Reproductions test -0.14±0.08 p.0.070 Trail-Making Test -0.04± 0.08 p.0.563   **Significant trends** *Vascular risk factors and structural brain aging.* Current smoking in midlife was associated with: greater annual increase in temporal horn volume and decrease in total brain volume and also predicted an increased risk of prominent change in temporal horn volume, Total Brain volume, and white matter hyperintensity volume.   **Reported limitations**  persons included are not representative of the general population   vascular risk factors are highly correlated with each other, making it difficult to tease out the individual effects of each  did not perform any correction for multiple testing as we considered our study as exploratory  community sample of relatively young subjects, excluding persons with clinical dementia, thus leading to limited variability in cognitive performance  longitudinal differences in brain structure may reflect an earlier effect of exposure to vascular risk than changes in cognition  measures of change in brain structure are assessed using automated procedures. lack of a direct measure for longitudinal change in hippocampal volume  measures of cognitive decline are subject to a learning effect  **Source of funding** Framingham Heart Study’s National Heart, Lung, and Blood Institute contract (N01-HC-25195) and by grants from the National Institute of Neurological Disorders and Stroke (R01 NS17950) and from the National Institute on Aging (R01 AG16495; AG08122; AG033193; AG031287). Dr. Debette was supported by a Fulbright grant and received an award from the Bettencourt-Schueller Foundation | |
| **Authors:** Dudas KA, Wilhelmsen L, Rosengren A  **Year:** 2007  **Citation:** Eur J Cardiovasc Prev Rehabil. 2007;14:122–127  **Country of study:** Sweden  **Aim of study:** Assess risk factors for future coronary bypass grafting as a first coronary event, and to compare them with riskfactors for a first acute myocardial infarction  **Study design:** Prospective cohort study  **Quality score: (++, + or -):**  **External validity score: (++, + or -):** | | | | | | | | | | | | | |
| **Number of people**  7434  **Demographics** Not reported | | **Number of people** 7388 **Characteristics**  **No CHD.** n=5578: Age, mean; SD (years) 51.4;2.3: BMI, mean; SD (kg/m2) 25.4;3.2: Family history, 1013 (18%): Diabetes, n 73 (1%): Current smoker, n 2653 (47%): Sedentary physical activity, n 1379 (25%): Permanent stress, n 1112 (20%) 375   **All MI.** n=1664: Age, mean; SD (years) 52.1;2.2: BMI, mean; SD (kg/m2) 26.0;3.4: Family history, 401 (24%): Diabetes, n 68 (4%): Current smoker, n 969 (58%): Sedentary physical activity, n 487 (29%): Permanent stress, n 375 (23%)  **Cases CABG.** without previous MI n=146: Age, mean; SD (years) 51.0;2.2: BMI, mean; SD (kg/m2) 25.8;3.2: Family history, n 48 (33%): Diabetes, n 6 (4%): Current smoker, n 60 (41%): Sedentary physical activity, n 28 (19%): Permanent stress, n 33 (23%)  **Location**  Goteborg  **Recruitment strategy** All men in the city who were born between 1915 and 1925 (n=30 000), except those born in 1923, were randomized into three groups of 10 000 men each  **Length of follow-up**  28 years  **Response rate and loss to follow-up** Not reported  **Eligible population** All men in the city who were born between 1915 and 1925   **Excluded populations**  Women excluded. Forty-six men undergoing CABG in connection with an operation for aortic stenosis were excluded | | | | **Relevant exposures** smoking habits, physical activity during leisure time, psychological stress  **Time** 1970-1998  M**easurment of exposure** Smoking habits coded as never smoked, former smoker of more than 1 month’s duration, smoking 1–14 g of tobacco per day, smoking 15–24 g and smoking 25 g or more per day. One cigarette was considered to contain 1 g of tobacco, a cigarillo 2 g and a cigar 5 g of tobacco. Physical activity during leisure time was categorized into four levels with 1 representing sedentary activity, 2 moderate activity such as walking or light gardening during at least 4 h per week, and 3 regular, strenuous, or 4 very strenuous activities. Because there were few men in category 4, the two highest categories were combined.  Psychological stress was assessed by way of one single question in the postal questionnaire and rated from 1 to 6, with 5 and 6 defined as permanent stress during the last year, or the last 5 years, before the examination | | **Outcomes** coronary bypass  **outcome measurement** AMI was defined as a discharge, or death with International Classification of Diseases. To identify cases of aorto-coronary bypass operations classification codes 3066, 3067 and 3091 were used prior to 1997 and FNA and FNC during 1997 and 1998. Codes for coronary angioplasty were not registered for the purpose of the present study  **Time**  Not reported | | **Analysis strategy** A multiple logistic regression analysis for the two diagnoses, AMI and CABG, was used in a generalized logit model. According to this we modeled the logits of three category’s response variables (no risk, AMI and CABG) against the risk factors. In this model, age-adjusted odds ratios are obtained for the two separate outcomes, and then compared to see difference.  C**onfounders** Age, family history of AMI, BMI, cholesterol, systolic blood pressure, treatment for hypertension, diabetes, smoking, physical activity, stress | | **Number** 128 men  **Effect estimates** **All AMI** *Hazard ratio (95% CI) age adjusted* Never smoking 1.00 Former smoker 1.19 (1.02–1.38) 1–14 g/day 1.70 (1.50–1.94) 15–24 g/day 1.90 (1.64–2.21) 25 g/day or more 1.89 (1.45–2.46)  Physical activity 1 1.00 2 0.86 (0.76–0.96) 3 0.80 (0.68–0.95)  Stress No 1.00 Yes 1.16 (1.04–1.31)  *Odds ratio age-adjusted 95% CI* Age (years) 1.31 1.24–1.39 Smoking (1–5) 1.51 1.43–1.60 Physical activity (1–3) 0.83 0.76–0.90 Stress(yes/no) 1.16 0.99–1.35  **CABG without prior AMI** *Hazard ratio (95% CI) age adjusted* Never smoking 1.00 Former smoker 1.29 (0.84–1.98) 1–14 g/day 0.96 (0.62–1.51) 15–24 g/day 1.11 (0.65–1.90) 25 g/day or more 2.19 (1.02–4.66)  Physical activity 1 1.00 2 1.23 (0.80–1.89) 3 1.66 (0.98–2.81)  Stress No 1.00 Yes 1.28 (0.86–1.89)  *Odds ratio age-adjusted 95% CI* Age (years) 0.85 0.72–1.00 Smoking (1–5) 0.78 0.66–0.92 Physical activity (1–3) 1.27 0.98–1.64 Stress(yes/no) 1.03 0.64–1.65  **Significant trends** high BMI, low physical activity and psychological stress were significantly associated only with AMI. Even light to moderate smoking (1–14 g/day) was associated with increased risk of AMI, hazard ratio 1.70 (1.50–1.94); whereas only very heavy smokers were more likely to undergo CABG, hazard ratio for 25 g/day or more 2.19 (1.02–4.66). Moderate smoking was not associated with coronary bypass grafting.  **Reported limitations**  only men of a comparatively limited age span were studied. Did not have angiographic data in any patient group. Patients with mild angina who did not undergo CABG were not studied  **Source of funding** This study was supported by the Heart and Lung Foundation, the Swedish Research Council and FoU-radet in Goteborg and Sodra Bohuslan, Vastra Gotalandsregionen | |
| **Authors:** Ekelund U, Brage S, Franks PW, Hennings S, Emms S, at al.  **Year:** 2005  **Citation:** Diabetes Care. 2005;28:1195–1200  **Country of study:**  **Aim of study:** Examine the prospective associations between physical activity energy expenditure, aerobic fitness, obesity, and the progression toward the metabolic syndrome  **Study design:** Population-based cohort  **Quality score: (++, + or -):**  **External validity score: (++, + or -):** | | | | | | | | | | | | | |
| **Number of people**  Not reported  **Demographics** Men (n = 246)  Baseline  Age (years) 53.3 ± 10.5  Height (cm) 175.3 ± 6.8  Weight (kg) 80.6 ± 10.6  BMI (kg/m2) 26.2 ± 2.9   Women (n = 359) Baseline  Age (years) 53.1 ±10.1  Height (cm) 162.3 ± 6.1 Weight (kg) 68.0 ± 12.6  BMI (kg/m2) 25.8 ± 4.4 | | **Number of people** Men (n = 246)  Women (n = 359)  **Characteristics**  Men  Age (years) 58.9 ± 10.6 Height (cm) 175.0 ± 6.7  Weight (kg) 81.2 ± 12.2 BMI (kg/m2) 26.5 ± 3.5  Women Age (years) 58.7 ± 10.2 Height (cm) 162.0 ± 6.2 Weight (kg) 69.5 ± 14.7 BMI (kg/m2) 26.4 ± 6.2  **Location**  Ely, UK  **Recruitment strategy** Randomly selected  **Length of follow-up**  median ± SD follow-up period of 5.6±0.30 years  **Response rate and loss to follow-up** Not reported  **Eligible population** Not reported  **Excluded populations** Not reported | | | | **Relevant exposures** Physical activity  **Time** Not reported  **Measurement of exposure** Height and weight were measured using a rigid stadiometer and calibrated scales in light clothing. Body circumference was measured in duplicate using a metal tape. Resistance was assessed using a standard bioimpedance technique  physical activity energy expenditure measured using the flex heart rate method  Total body water and fat-free mass were calculated using the impedance index   Fat mass was calculated as body weight minus fat-free mass. Percentage of body fat was calculated as fat mass/body weight x 100.  Blood pressure was measured in seated position using an Accutorr automatic sphygmomanometer. Systolic and diastolic blood pressures were measured in triplicate at minute intervals, and the mean of these measurements was used in analyses. | | **Outcomes** Metabolic syndrome  **Outcome measurement** Blood samples  **Time**  2001–2003 | | **Analysis strategy** generalized linear models  **Confounders** included all subcomponents of the metabolic syndrome and was adjusted for baseline zMS, sex, age, smoking, SES, and follow-up time | | **Number** 84 subjects (46 male)  **Effect estimates** Outcome (SD)  PAEE -coefficients (95% CI)  Insulin 0.002 ( 0.0037 to 0.00053) BMI 0.00004 ( 0.00076 to 0.00084) WHR 0.00058 ( 0.0006 to 0.0017) 2-h glucose 0.00008 ( 0.0016 to 0.0015) DBP 0.00086 ( 0.0024 to 0.0007) SBP 0.002 ( 0.0037 to 0.00064) Triglycerides 0.000088 ( 0.0015 to 0.0015) HDL 0.00074 ( 0.0005 to 0.002) zMS 0.00085 ( 0.00177 to 0.000068) zMS-Ob 0.0011 ( 0.0021 to 0.0006974)  **Significant trends** Baseline PAEE significantly predicted fasting insulin at follow-up after adjustment for baseline age, sex, smoking, SES, fasting insulin, aerobic fitness, and duration of follow-up (standardized β 0.0012, P 0.01). This association was not affected by further adjustment for baseline BMI (standardized β 0.0013, P 0.005), baseline WHR (standardized β 0.0012, P 0.007), or baseline percentage body fat (standardized β 0.0012, P 0.006).  **Reported limitations  Author** Not powered to explore the possibility of nonlinearity. Measure of aerobic fitness is less precise than a true maximal test  **Source of funding** The Medical Research Council | |
| **Authors**: [Elwood P](http://www.ncbi.nlm.nih.gov/pubmed?term=Elwood%20P%5BAuthor%5D&cauthor=true&cauthor_uid=24349147), [Galante J](http://www.ncbi.nlm.nih.gov/pubmed?term=Galante%20J%5BAuthor%5D&cauthor=true&cauthor_uid=24349147), [Pickering J](http://www.ncbi.nlm.nih.gov/pubmed?term=Pickering%20J%5BAuthor%5D&cauthor=true&cauthor_uid=24349147), [Palmer S](http://www.ncbi.nlm.nih.gov/pubmed?term=Palmer%20S%5BAuthor%5D&cauthor=true&cauthor_uid=24349147), [Bayer A](http://www.ncbi.nlm.nih.gov/pubmed?term=Bayer%20A%5BAuthor%5D&cauthor=true&cauthor_uid=24349147), et al. **Year:** 2013 **Citation:** [PLoS One.](http://www.ncbi.nlm.nih.gov/pubmed/24349147) 2013;8(12):e81877  **Country of study:**  South Wales (Caerphilly) **Aim:** To assess influence of lifestyle on cognitive function **Study design:** Longitudinal **Quality score: +**  **Applicability: ++** | | | | | | | | | | | | | |
| **Source population:**  *cohort of men ages 45-59 years in small South Wales UK town  *after 1979 baseline survey, men were re-examined every 5 years | | **Study population:**  *analysis restricted to **2,235** men (89% of defined population) examined at baseline  **Exclusion:**  *****men with evidence of disease at baseline (diabetes, a history of angina, chest pain, clinical or ECG evidence of infarction, stroke, high blood pressure  *men with evidence of cognitive impairment at baseline omitted  **Attrition:**  - | | | | **Exposures:**  *self-reported smoking history, physical activity and alcohol consumption captured through food frequency questionnaire at baseline in 1979  **uptake of health behaviours assessed:*  health behaviour:  >not smoking including ex-smokers;  >diet: 3+ portions of fruit and/or vegetables a day and 30% less calories from fat;  >physical activity: walking 2+ miles or cycling 10+ miles each day, or vigorous exercise;  >alcohol: 3 or fewer units per day | | **Outcomes:**  *****incidence of diabetes, vascular disease, cancer, all-cause mortality, cognitive impairment and dementia ascertained through self-report, primary care and hospital records, CT scans and ECG, Office of National Statistics (for deaths and cancer registrations)  *in 2004, cognitive impairment and dementia assessed in late-life in participants ages 70-85 years using CAMDEX, CAMCOG, Frontal Assessment Battery, the Clinical Dementia Rating, and the Informant Questionnaire on Cognitive Decline in the Elderly | | **Analysis:**  *logistic regression used to assess influence of lifestyle (number of healthy behaviours practiced) on cognitive function  **Confounders:**  *age, social class | | **Results:**  *the odds of diabetes were lower among those who regularly exercised (OR=0.63, [0.46, 0.85])  *the odds of vascular disease were lower among those who were classified as non-smoking (OR=0.70, [0.58, 0.84]); similar findings reported for cancer (OR=0.65, [0.54, 0.79])  *the odds of cognitive impairment were lower for those who regularly exercised (OR=0.62, [0.41, 0.92]); similar findings reported for dementia  *the odds of death were lower among those who did not smoke (OR=0.42, [0.35, 0.51])  *generally, the greater the number of healthy lifestyle behaviours the participants adopted, the lower the odds of diabetes, vascular disease, cognitive impairment, dementia, all-cause mortality  **Limitations:**  *impact of healthy lifestyles underestimated due to small number of men adhering to all healthy behaviours (small cell sizes)  *residual confounding  **Source of funding:**  *none reported | |
| **Authors:** Emberson JR, Shaper AG, Wannamethee SG, Morris RW, Whincup PH  **Year:** 2005  **Citation:** Am J Epidemiol. 2005;161:856–863  **Country of study:** UK  **Aim of study:** Examine associations between alcohol intake and the 20-year risk of coronary heart disease, stroke, and all-cause mortality  **Study design:** Cohort  **Quality score: (++, + or -):**  **External validity score: (++, + or -):** | | | | | | | | | | | | | |
| **Number of people**  7,735 men aged 40–59 years  **Demographics** Not reported | | **Number of people** 6,544  **Characteristics**  middle-aged British men  **Location**  Throughout Britain  **Recruitment strategy** general practice  **Length of follow-up**  over 20 years from 1978/1980 to 1998/2000  **Response rate and loss to follow-up** 77%  **Eligible population** one general practice in each of 24 British towns  **Excluded populations**   Women and men with evidence of cardiovascular disease. 1,186 men with baseline evidence of CVD. five had incomplete baseline data | | | | **Relevant exposures** Alcohol  **Time** Between 1998 and 2000   time until censoring or first cardiovascular event, whichever is lowest  **Measurment of exposure** A five-point scale from zero (none) to four (heavy) was used to denote the alcohol intake level at the baseline assessment and at each of the follow-up assessments. Categorised as 1) nondrinkers; 2) occasional drinkers (1–2 times/month or on special occasions); 3) light drinkers (1–2 drinks/day or ‘‘weekend only’’ drinkers (1–6 drinks/day)); 4) moderate drinkers (3–6 drinks/day or weekend only drinkers (>6 drinks/day)); and 5) heavy drinkers (>6 drinks every day). From these average exposure levels, each individual was reclassified on the original scale (an average exposure of <0.5 was defined as ‘‘none,’’ 0.5–1.49 was defined as ‘‘occasional," 1.5–2.49 was defined as ‘‘light,’’ 2.5–3.49 was defined as ‘‘moderate,’’ and 3.5 was defined as ‘‘heavy’’). | | **Outcomes** cardiovascular morbidity and all-cause mortality  **Outcome measurement** Information on incident mortality was collected through the established ‘‘tagging’’ procedures provided by the National Health Service central registers.  **Time**  Between 1998 and 2000 | | **Analysis strategy** Cox proportional hazards regression. hazard ratios were calculated as ‘‘floating absolute risks’’ and inverse variance-weighted quadratic curves were fitted through the values. The ‘‘relative informativeness’’ of baseline versus average alcohol intake was evaluated by examining the contributions made by each measure to the X^2^ likelihood ratio statistic in the Cox regression model  **Confounders** adjustment for cigarette smoking, physical activity, and body mass index. adjustment for intake variation | | **Number** 6,544 men  **Effect estimates** Major coronary heart disease  Baseline exposure  Hazard ratio (95% CI) None 1.00 (0.75, 1.34)  Occasional 1.00  Light 0.81 (0.68, 0.96)  Moderate 0.94 (0.78, 1.12)  Heavy 1.08 (0.86, 1.35)   Usual exposure Hazard ratio 95% CI None 0.91 (0.72, 1.15)  Occasional 1.00  Light 0.74 (0.63, 0.87)  Moderate 1.01 (0.84, 1.21)  Heavy 1.74 (1.31, 2.33)   Stroke  Baseline exposure  Hazard ratio 95% CI None 1.58 (1.02, 2.44)  Occasional 1.00  Light 0.97 (0.72, 1.31)  Moderate 1.19 (0.88, 1.61) Heavy 1.54 (1.06, 2.22)   Usual exposure Hazard ratio 95% CI None 1.08 (0.73, 1.60)  Occasional 1.00  Light 0.93 (0.71, 1.22)  Moderate 1.45 (1.08, 1.96)  Heavy 2.33 (1.46, 3.71)   All-cause mortality Baseline exposure  Hazard ratio 95% CI None 1.22 (0.98, 1.52)  Occasional 1.00  Light 0.88 (0.77, 1.01)  Moderate 1.12 (0.98, 1.29) Heavy 1.44 (1.21, 1.72)  Usual exposure Hazard ratio 95% CI  None 0.93 (0.77, 1.12) Occasional 1.00 Light 0.82 (0.72, 0.93) Moderate 1.32 (1.15, 1.52) Heavy 2.27 (1.84, 2.81)  **Significant trends** After adjustment for variation in alcohol intake risks among heavy drinkers were respectively, 32%, 86% and 70% higher than for occasional drinkers. After adjustment for intake, regular light alcohol consumption was associated with a statistically sig 26% reduced risk of CHD and 18% reduced risk of all-cause mortality, as well as a statistically insignificant 7% reduced risk of stroke. Moderate drinking and heavy drinking associated with substantially increased risks of stroke, all-cause mortality, and (to a lesser degree) major CHD  **Reported limitations**  No limitations reported by author  **Reviewer** misclassification of consumption  misclassification of outcome  **Source of funding** Not reported | |
| **Authors:** Englund U, Nordström P, Nilsson J, Hallmans G, Svensson O, et al.  **Year:** 2013  **Citation:** Osteoporos Int. 2013;24:533–540  **Country of study:** Sweden  **Aim of study:** Investigate whether a physically active lifestyle in middle-aged women was associated with a reduced risk of later sustaining a low-trauma wrist fracture  **Study design:** Population-based nested case–control study  **Quality score: (++, + or -):**  **External validity score: (++, + or -):** | | | | | | | | | | | | | |
| **Number of people** ~35,000 subjects **Demographics** Mean age at baseline was 54.3±5.8 years, and mean age at fracture was 60.3±5.8 years | | **Number of people** 778 **Characteristics** Means (±SD) **Cases (n=376).** Age (year) 54.3±5.9: Height (cm) 164.2±5.7: Weight (kg) 67.4±10.5: BMI 24.8±3.7: Follow-up time (year) 11.2±2.6: Distance to work (km) 12.5±15.2: Low active 18.1±16.1: Moderate active 6.9±11.4: High active 2.9±6.9: Current smokers 21.1: Never smokers 53.4: Alcohol users 79.3   **Controls (n=402).** Age (year) 54.3±5.8: Height (cm) 164.0±5.8: Weight (kg) 69.0±12.0: BMI 25.6±4.3: Follow-up time (year) 11.1±2.5: Distance to work (km) 11.9±17.3: Low active 21.5±20.2: Moderate active 6.5±12.6: High active 2.7±5.4: Current smokers 25.0: Never smokers 50.2: Alcohol users 76.5   **Location** Sweden, county of Västerbotten **Recruitment strategy** All inhabitants 40, 50, or 60 years old are invited to a health survey and are also asked to donate a blood sample **Length of follow-up** Cases Follow-up time (year) 11.2±2.6   Controls Follow-up time (year) 11.1±2.5  **Response rate and loss to follow-up** Not reported **Eligible population** inclusion in contains three population-based subcohorts. fracture case was compared with at least one control drawn from the same cohort and matched for age and week of reporting data **Excluded populations** Subjects who had a wrist fracture before they were recruited into the VIP cohort were subsequently excluded. Another exclusion criterion was if the fracture had occurred before the age of 45 years or was the result of a major trauma | | | | **Relevant exposures** commuting activities, occupational physical activity, exercise, leisure time activities, walking and bicycling activities, smoking habits, alcohol habits  **Time** 1985  M**easurement of exposure** self-assessment questionnaire includes questions relating to the subject’s level of occupational activity, commuting activity (type of traveling to and from work), and different leisure time activities, if subject has performed any exercise wearing training clothes in the last 3 months and to training habits in youth Commuting activities for each of the four seasons were defined in three categories (Car-bus, bicycling, and walking).  Occupational physical activity  divided into three categories: low, moderate, and high physically demanding work.   Exercise in training clothes two groups: performed or not performed  Physical activity in youth was defined in three groups: physical training at school only (low activity), training and/or competing at an amateur level (moderate activity), and competing at an elite level (high activity).   Frequency of leisure time activities was based on seven different regular activities: walking and/or bicycling (besides the commuting activity), dancing, snow shoveling, gardening, hunting/fishing, and berry/mushroom picking  The frequencies of walking and bicycling activities defined as low (<one to two times/month) moderate (three to four times/month), and high (two - three times/week or more). The remaining leisure time activities were categorized as not performed or performed (if performed at least every month during the season)  Smoking habits were coded as “never smoker”, “former smoker” and “current smoker”  Alcohol habits coded as “teetotaller” or “alcohol user” | | **Outcomes** Wrist fracture **outcome measurement** identified from a prospective injury-fracture database at the Umeå University Hospital **Time** 31 December 2008 | | **Analysis strategy** Conditional logistic regression analysis   Subgroup analyses performed in women with data on HRT, alcohol use, calcium and vitamin D intake  **Confounders** adjustments for height, body mass index, smoking, and menopausal status, dietary habits and other physical activity variables  Subgroup analyses were also performed in women with available data on HRT and alcohol use and also on women with data on calcium and vitamin D intake | | **Number** 376  **Effect estimates** Risk factor Odds ratio (95% CI) Height 1.00 (0.96–1.030 BMI 0.94 (0.89–0.99) Smoking Never smoker 1.00 Former smoker 1.10 (0.66–1.83) Current smoker 0.84 (0.51–1.38) Commuting activity Low 1.00 Moderate 0.98 (0.60–1.59) High 0.48 (0.27–0.88) Occupational activity Low 1.00 Moderate 0.80 (0.53–1.19) High 0.96 (0.39–2.38) Training activity 1.14 (0.75–1.74) Bicycling Low 1.00 Moderate 1.01 (0.54–1.92) High 1.13 (0.70–1.82) Dancing 0.42 (0.22–0.81) Snow shoveling 0.50 (0.32–0.79)  **Significant trends** subjects with active commuting (especially walking) were at significantly lower risk of sustaining a wrist fracture (OR 0.48; 95 % CI 0.27–0.88) compared with those who commuted by car or bus, in middle-aged women   **Reported limitations**  no data on bone mineral density, neuromuscular functions or Vitamin D or increased muscle strength or balance. No estimation on the intensity or duration of the different activities or the loading characteristics. Differences in the level of outdoor activities and exposure to sunlight. All information about physical activity, health, and other lifestyle variables were self-estimated. Questionnaire was not validated. Assessments of the different physical variables were crude. Physical activity and other variables were only assessed at baseline  **Reviewer** Case-control  **Source of funding** Swedish Research Council (K2006-72X-20155013), Swedish Sports Research Council (87/06), Swedish Society of Medicine, Medical Faculty of Umeå, and by project grants from the Erik and Anne-Marie Detlof Foundation and the J C Kempe Foundation | |
| **Authors:** Englund U, Nordström P, Nilsson J, Bucht G, Björnstig U, et al.  **Year:** 2011  **Citation:** Osteoporos Int. 2011;22:499–505  **Country of study:** Sweden  **Aim of study:** investigate whether physical activity is associated with a decreased risk of later sustaining a hip fracture  **Study design:** population-based case-control study  **Quality score: (++, + or -):**  **External validity score: (++, + or -):** | | | | | | | | | | | | | |
| **Number of people**  70,000  **Demographics** Not reported | | **Number of people**  **Characteristics**  Means (±SD) **Cases (n=81).** Age (year) 57.2±5.0: Height (cm) 165.6±6.2: Weight (kg) 68.2±12.9: BMI 24.8±4.2: Percent (%) Smokers 33.8, Former smokers 6.8, Never smokers 59.5, Alcohol users 78.3   **Controls(n=156).** Age (year) 57.2±5.0: Height (cm) 162.1±6.9: Weight (kg) 68.8±11.9: BMI 26.2±4.8: Percent (%) Smokers 20.4, Former smokers 16.3, Never smokers 63.3, Alcohol users 72.7   **Location**  Sweden  **Recruitment strategy** All inhabitants 40-60 years old were invited to a health survey and asked to donate a blood sample  **Length of follow-up**  Case follow up time (year) 11.0±3.2  Control follow up time (year) 11.0±3.2   **Response rate and loss to follow-up** Not reported  **Eligible population** Involved in Västerbotten Intervention Programme  **Excluded populations**  Subjects who had a hip fracture or another fragility fracture before they were recruited into the VIP cohort. Women taking other medications known to affect bone metabolism were excluded from the study cohort | | | | **Relevant exposures** physical activity  **Time** Not reported  **Measurement of exposure** self-assessment questionnaire includes questions relating to the subject’s level of occupational activity, commuting activity (type of traveling to and from work), and different leisure time activities, if subject has performed any exercise wearing training clothes in the last 3 months and to training habits in youth  Commuting activities for each of the four seasons were defined in three categories (Car-bus, bicycling, and walking).  Occupational physical activity divided into three categories: low, moderate, and high physically demanding work.   Exercise in training clothes two groups: performed or not performed Frequency of leisure time activities was based on seven different regular activities: walking and/or bicycling (besides the commuting activity), dancing, snow shoveling, gardening, hunting/fishing, and berry/mushroom picking  The frequencies of walking and bicycling activities defined as low (<one to two times/month) moderate (three to four times/month), and high (two - three times/week or more). The remaining leisure time activities were categorized as not performed or performed (if performed at least every month during the season)  Smoking habits were coded as “never smoker”, “former smoker” and “current smoker”  Alcohol habits coded as “teetotaller” or “alcohol user” | | **Outcomes** Hip fracture  **outcome measurement** Fracture cases were identified from a prospective injury fracture database at the Umeå University Hospital  **Time**  Time from baseline to when the hip fracture occurred was 8.4±3.8 years and the mean age at fracture was 65.4±6.4 years | | **Analysis strategy** Conditional logistic regression analysis  multiple-adjusted associations were calculated with adjustments made for height, weight, smoking habits, and menopausal status.   Subgroup analyses were also performed using HRT or alcohol habits as potential confounding variables.  **Confounders** adjustments for height, weight, smoking, and menopausal status | | **Number** 202  **Effect estimates** Independent predictors of hip fracture risk Adjusted odds ratio (95% CI) Height 1.26 (1.14–1.40) Weight 0.94 (0.91–0.98) Smoking Never smoker 1 Former smoker 0.34 (0.08–1.50) Smoker 2.37 (0.81–6.92) Menopause 0.57 (0.03–11.4) Walking Never 1 1 time/week 0.14 (0.04–0.53) ≥2 times/week 0.33 (0.10–1.01) Spare time activity Low 1 Moderate 0.19 (0.08–0.46) High 0.17 (0.05–0.64)  **Significant trends** walking and seasonal-dependent physical spare time activities were associated with a significant hip fracture risk reduction in middle-aged women. Significantly reduced hip fracture risk in women walking once a week only. Lower odds ratio for women with higher frequency of walking.  **Reported limitations  Author** no data on bone mineral density, neuromuscular functions or Vitamin D. Information about lifestyle variables were self-reported. Questionnaire was not validated. Assessments of the different physical variables were crude. Physical activity and other variables were only assessed at baseline. Number of hip fracture cases in this cohort was also rather low. Adequately powered  **Reviewer** Cross-sectional  **Source of funding** Swedish Research Council (K2006-72X-20155013), Swedish Sports Research Council (87/06, 169/07), Swedish Society of Medicine, Medical Faculty of Umeå, and by project grants from the Erik and Anne-Marie Detlof Foundation and the J C Kempe Foundation. | |
| **Authors:** Eskelinen, Ngandu T, Helkala EL, Tuomilehto J, Nissinen A. et al.  **Year:** 2008  **Citation:** Int J Geriatr Psychiatry. 2008;23:741–747.  **Country of study:** Finland  **Aim of study:** Investigate the association of midlife dietary fat intake to cognitive performance, and to the occurrence of clinical mild cognitive impairment  **Study design:** Longitudinal population-based study  **Quality score: (++, + or -):**  **External validity score: (++, + or -):** | | | | | | | | | | | | | |
| **Number of people**  2000  **Demographics** 835 women (62.3%) and 506 men (37.7%) had a mean age at midlife of 50.2 (6.0) years, and | | **Number of people** 1449  **Characteristics**  mean age at midlife of 71.1 (4.0) years at the follow-up.  **Location**  Kuopio and Joensuu, Eastern Finland  **Recruitment strategy** random sample  **Length of follow-up**  21 (SD 4.9) years  **Response rate and loss to follow-up** 72%  **Eligible population** study came from four separate, independent population-based random samples studied within the North Karelia Project and the FINMONICA study in 1972, 1977, 1982 or 1987  **Excluded populations**  individuals who had dementia (n=68) were excluded. Those individuals who had dementia (n=68) were excluded, and 40 individuals did not complete all evaluations | | | | **Relevant exposures** Diet  **Time** 1998  **Measurement of exposure** Dietary habits investigated using a semiquantitative food frequency questionnaire consisting of mostly qualitative or frequency-based questions. Consumption of milk, sour milk and spreads assessed quantitatively | | **Outcomes** MCI, global cognitive and executive functions, episodic, semantic and prospective memory and psychomotor speed  **outcome measurement** Mayo Clinic AD Research Center criteria; Mini Mental State Examination; Category Fluency Test; Purdue Peg Board task; letter digit substitution test; the Stroop test; memory task by Einstein  **Time**  1998 | | **Analysis strategy** logistic regression analyses were used to calculate Odds Ratios with 95% Confidence Intervals  2 models reported but 3rd model undertaken controlling additionally for midlife leisure-time physical activity, alcohol consumption, or vascular disorders at late-life including myocardial infarction, stroke, and diabetes, the results remained unchanged or virtually the same  **Confounders** adjusted for midlife age, sex, education, follow-up time and other subtypes of fats, ApoE e4 carrier status, smoking, systolic blood pressure, cholesterol, and BMI | | **Number** 1,341  **Effect estimates** Association of midlife fat intake with clinical MCI in late-life Total fat (from milk products and spreads) Low (0–38.0 g) 1 High (>38.0 g) 1.69 (1.00–2.87) saturated fatty acids (from milk products and spreads) Low (0–21.6 g) 1 High (>21.6 g) 2.36 (1.17–4.74) polyunsaturated fatty acids (from milk products and spreads) Low (0–2.1 g) 1 High (>2.1 g) 0.94 (0.45–1.96) PUFA-SFA ratio (milk products and spreads) Low (0–0.05) 1 High (>0.05) 0.91 (0.53–1.56) monounsaturated fatty acids (from milk products and spreads) Low (0–11.3 g) 1  High (>11.3 g) 1.81 (0.87–3.80) Association of midlife fat intake with Global cognitive function (MMSE) Total fat from milk and spreads Low (0–38.0 g) 26.2 (0.1)  High (>38.0 g) 25.9 (0.1)  p-value 0.05  SFA from milk and spreads Low (0–21.6 g) 26.2 (0.1)  High (>21.6 g) 25.8 (0.1)  p-value 0.03  PUFA from milk and spreads Low (0–2.1 g) 26.0 (0.1)  High (>2.1 g) 26.1 (0.1)  PUFA–SFA ratio (milk and spreads) Low (0–0.05) 26.0 (0.1)  High (>0.05) 26.1 (0.1)  MUFA from milk and spreads Low (0–11.3 g) 26.1 (0.1)  High (>11.3 g) 26.0 (0.1)  Association of midlife fat intake with Episodic memory Total fat from milk and spreads Low (0–38.0 g) 5.0 (0.04)  High (>38.0 g) 4.9 (0.1)  SFA from milk and spreads Low (0–21.6 g) 5.0 (0.05)  High (>21.6 g) 4.9 (0.1)  PUFA from milk and spreads Low (0–2.1 g) 5.0 (0.05)  High (>2.1 g) 4.9 (0.1)  PUFA–SFA ratio (milk and spreads) Low (0–0.05) 5.0 (0.04)  High (>0.05) 4.9 (0.1)  MUFA from milk and spreads Low (0–11.3 g) 5.0 (0.05)  High (>11.3 g) 4.9 (0.1)  Association of midlife fat intake with Semantic memory Total fat from milk and spreads Low (0–38.0 g) 20.3 (0.2) High (>38.0 g) 20.0 (0.3)  SFA from milk and spreads Low (0–21.6 g) 20.4 (0.2)  High (>21.6 g) 19.7 (0.4)  PUFA from milk and spreads Low (0–2.1 g) 19.9 (0.2)  High (>2.1 g) 20.8 (0.3)  PUFA–SFA ratio (milk and spreads) Low (0–0.05) 20.1 (0.2)  High (>0.05) 20.3 (0.3)  MUFA from milk and spreads Low (0–11.3 g) 20.4 (0.2)  High (>11.3 g) 19.7 (0.4)  Association of midlife fat intake with Psychomotor speed Total fat from milk and spreads Low (0–38.0 g) 0.10 (0.03)  High (>38.0 g) -0.02 (0.04)  p-value 0.02 SFA from milk and spreads Low (0–21.6 g) 0.08 (0.03)  High (>21.6 g) 0.02 (0.05)  PUFA from milk and spreads Low (0–2.1 g) 0.04 (0.03)  High (>2.1 g) 0.1 (0.04) PUFA–SFA ratio (milk and spreads) Low (0–0.05) 0.04 (0.03)  High (>0.05) 0.1 (0.04)  MUFA from milk and spreads Low (0–11.3 g) 0.09 (0.03)  High (>11.3 g) 0.01 (0.05)  Association of midlife fat intake with Executive function (Stroop) Total fat from milk and spreads Low (0–38.0 g) 39.8 (0.8)  High (>38.0 g) 41.0 (1.1)  SFA from milk and spreads Low (0–21.6 g) 39.3 (0.9)  High (>21.6 g) 42.0 (1.5)  PUFA from milk and spreads Low (0–2.1 g) 39.9 (0.9)  High (>2.1 g) 40.8 (1.3)  PUFA–SFA ratio (milk and spreads) Low (0–0.05) 40.9 (0.8)  High (>0.05) 38.6 (1.1)  MUFA from milk and spreads Low (0–11.3 g) 39.7 (0.9)  High (>11.3 g) 41.2 (1.5)  Association of midlife fat intake with Prospective memory Total fat from milk and spreads Low (0–38.0 g) 2.7 (0.03) High (>38.0 g) 2.6 (0.04) SFA from milk and spreads Low (0–21.6 g) 2.7 (0.04) High (>21.6 g) 2.6 (0.1) p-value 0.05 PUFA from milk and spreads Low (0–2.1 g) 2.7 (0.04) High (>2.1 g) 2.7 (0.05) PUFA–SFA ratio (milk and spreads) Low (0–0.05) 2.7 (0.03) High (>0.05) 2.7 (0.04) MUFA from milk and spreads Low (0–11.3 g) 2.7 (0.04) High (>11.3 g) 2.7 (0.1)  **Significant trends** midlife dietary fat intake was related to cognitive performance, especially in domains of global cognitive function, semantic memory and psychomotor speed, and to the occurrence of MCI later in life  **Reported limitations  Author** dietary data was available from limited sources. Results of stratified analyses may be inconclusive due to insufficient power  **Reviewer** Authors undertake 3 models but show results from two. In conclusion refer to the three models. Selective reporting bias. Revisit when we have time to go through thoroughly.  **Source of funding** The study was supported by EVO-grants of Kuopio University Hospital (5772708, 5772720), Academy of Finland grants 103334, 120676 and 206951, EU grant QLK-2002-172, The Swedish Council for Working Life and Social Research, the Finnish Cultural Foundation, The Foundation of Juho Vainio, the Gamla Tjanarinnor Foundation, and Uulo Arhio foundation | |
| **Authors**: Eskelinen MH, Ngandu T, Tuomilehto J, Soininen H, Kivipelto M **Year:** 2009 **Citation:** J Alzheimers Dis. 2009;16(1):85-91 **Country of study:** Finland **Aim:** To evaluate the association between mid-life coffee and tea drinking and the risk of late-life dementia **Study design:** Longitudinal **Quality score:** +  **Applicability:** | | | | | | | | | | | | | |
| **Source population:**  *a population-based random sample of 2000 people aged 65-79 years and living in Joensuu or Kuopio, Eastern Finland in 1997 were invited to participate in 1998 *response rate of 71% (1409), of which 875 (62%) were women and 534 (38%) were men | | **Study population:**  *see ‘Source population’  *Sociodemographics:* *at midlife examination, **875** women and **534** men had mean age of 50.4 years, while at late-life examination, mean age was 71.3 years   *Follow-up:* *mean follow-up time of 21 years  *response rate of 71%  **Exclusion:**  -  **Attrition:**  - | | | | **Exposures:**  *coffee and tea consumption examined at midlife using validated semi-quantitative food-frequency questionnaire *coffee drinking categorized as: 0-2 cups/day (low), 3-5 cups/day (moderate), >5 cups/day (high) *Tea consumption categorized as: none (0 cups/day), drinking tea (>=1 cups/day) | | **Outcomes:**  *cognitive status assessing through screening, clinical and differential diagnosis *participants with score <=24 on MMSE addressed for further examination where dementia diagnosis may have been made | | **Analysis:**  *logistic regression was used to determine the influence of coffee or tea on the risk of developing dementia and AD   **Confounders:**  *midlife age, education, follow-up time and community of residence, midlife smoking, SBP, serum total cholesterol, BMI, physical activity, myocardial infarction, stroke, diabetes mellitus, depression; sex and APOE 4 assessed as effect modifiers | | **Results:**  *61 people had dementia, of which 48 had AD;  *moderate coffee drinkers had a 65-70% decreased risk of dementia and a 62-64% decreased risk of AD compared with low coffee consumers  *odds of developing dementia were lower for those consuming moderate amounts of coffee (3-5 cups) compared to low amounts (0-2 cups) [OR=0.30, (0.10, 0.93)]  *APOE4 stratification:  **for APOE4 carriers, odds of developing dementia were lower among moderate coffee consumers compared to low coffee consumers [OR=0.32, (0.11, 0.92)]  S*ex stratification:* *for males: lower odds for dementia were reported among moderate and high coffee consumers compared to low coffee consumers [OR=0.27, (0.08, 0.89); OR=0.36, (0.13, 0.97)]  **Limitations:**  *residual confounding due to measurement error  *sample may have been too small to detect significant differences in interaction analyses and dose-response effects   Bias: *self-reported data, residual confounding due to measurement error in the assessment of confounding factors  **Source of Funding:**  *EVO-grant of Kuopio University Hospital, Academy of Finland grants, EU grant, the Swedish Council for Working Life and Social Research, the Finnish Cultural Foundation, the Foundation of Juho Vainio, the Gamla Tjanarinnor Foundation, the Helsingin Sanomain 100-vuotissaatio, and the Yrjo Jahnsson Foundation | |
| **Authors:** Field AE, Malspeis S, Willett WC  **Year:** 2009  **Citation:** Arch Intern Med. 2009;169(9):881-886  **Country of study:** USA  **Aim of study:** aAssess the independent association of weight cycling with mortality  **Study design:** Prospective  **Quality score: (++, + or -):**  **External validity score: (++, + or -):** | | | | | | | | | | | | | |
| **Number of people**  121 701  **Demographics** Not reported | | **Number of people** 44 882 **Characteristics**  **Noncyclers.** Age in 1992, mean (SD), y 57.7 (7.1); BMI in 1992, mean (SD) 25.0 (4.3); BMI categories in 1992 17.0-20.9 1.0, 21.0-24.9 53.4, 25.0-29.9 28.6, >30.0 11.4; Smoking status Never 45.4  Past 38.7, Current 15.8; Alcohol intake g/day, mean (SD), 5.4 (9.8); Quintiles of MET hours of activity per week, % 1 (0.2-3.1 METs) 18.0, 2 (3.2-8.3 METs) 20.3, 3 (8.4-11.1 METs) 20.6, 4 (16.0-21.9 METs) 20.0, 5 (30.0-53.1 METs) 20.8  **Mild Cyclers.** Age in 1992, mean (SD), y 55.6 (6.7); BMI in 1992, mean (SD) 28.7 (4.8); BMI categories in 1992 17.0-20.9 0.1, 21.0-24.9 20.1, 25.0-29.42.1, >30.0 31.9; Smoking status Never 44.1, Past 45.1, Current 10.5; Alcohol intake g/day, mean (SD),4.3 (8.4); Quintiles of MET hours ofactivity per week, % 1 (0.2-3.1 METs) 18.4, 2 (3.2-8.3 METs) 20.5, 3 (8.4-11.1 METs) 19.8, 4 (16.0-21.9 METs) 21.6, 5 (30.0-53.1 METs) 19.3   **Severe Cyclers.** Age in 1992, mean (SD), y 55.2 (6.5); BMI in 1992, mean (SD) 32.6 (6.2); BMI categories in 1992 17.0-20.9 0.0, 21.0-24.9 7.8, 25.0-29.9 28.0, >30.0 58.7; Smoking status Never 41.6 Past 47.4; Current 10.7; Alcohol intake g/day, mean (SD), 3.1 (7.4); Quintiles of MET hours of activity per week, % 1 (0.2-3.1 METs) 23.6: 2 (3.2-8.3 METs) 21.7: 3 (8.4-11.1 METs) 18.3: 4 (16.0-21.9 METs) 18.2: 5 (30.0-53.1 METs) 18.0  **Location**  USA  **Recruitment strategy** postal questionnaire  **Length of follow-up**  Variable from 1976 to 1992 or 2004  **Response rate and loss to follow-up** Women who did not answer the 1988 or 1992 questionnaire (n=23 914), were diagnosed as having cancer (other than nonmelanoma skin cancer) or heart disease (n=9557), did not complete all of the intentional weight loss questions (n=34 560), reported no losses in the past 20 years but reported losses in the past 4 years (n=19), did not report their weight at age 18 years (n=5807)  **Eligible population** female registered nurses aged 30 to 55 years  **Excluded populations**  Women who had a body mass index (BMI), calculated as weight in kilograms divided by height in meters squared, of less than 17 (n=286), reported bypass surgery (in 1992) (n=108), or died in the period of 1988 to 1994 (n=551) were excluded from the analysis | | | | **Relevant exposures** Height and weight  **Time** Height and weight were ascertained in 1976, and current weight was assessed on each follow-up questionnaire  M**easurment of exposure** Body mass index was calculated from self-reported information on weight and height  Net weight change, irrespective of intentionality, was assessed by calculating the difference in weight reported at 2 time points  “Within the last 20 years, how many times did you lose each of the following amounts of weight on purpose (excluding illness or pregnancy)?” and “Within the last 4 years, how many times did you lose each of the following amounts of weight on purpose (excluding illness or pregnancy)?” The responses were 0, 1 to 2, 3 to 4, 5 to 6, or 7 or more times for each of the magnitudes of weight loss (2.3-4.1 kg, 4.5-8.6 kg, 9.1-22.2 kg, and 22.7 kg) | | **Outcomes** Deaths  **outcome measurement** Deaths were reported by next of kin, the postal service, or ascertained by the National Death Index  **Time**  1992 or 2004 | | **Analysis strategy** Multivariate Cox proportional hazards models, stratified by age in months and calendar time and that controlled for other potential confounders  **Confounders** age, body mass index at age 18 years, weight change from age 18 years to start of the cycling period (1976 for models assessing cycling during the past 20 years or 1988 for models assessing cycling during the past 4 years), smoking status with number of cigarettes currently smoked per day (never; past; current, 1-4; current, 5-24; current, 25-34; current, 35-45; current, number unknown), menopausal status, postmenopausal hormone therapy (premenopausal, never, past, or current), alcohol, activity level, and change in activity level, net weight change from the start of the cycling period (1976 for models assessing cycling during the past 20 years or 1988 for models assessing cycling during the past 4 years) until 1992 (ie, end of the cycling period). net weight change from the start of the cycling period (1976 for models assessing cycling during the past 20 years or 1988 for models assessing cycling during the past 4 years) until 2004 or the end of the follow-up period, instead of net weight change during the cycling period | | **Number** Cycling Between 1972 and 1992  Noncycler (n=32 836) Mild Cycler (n=8452) Severe Cycler (n=3594) Cycling Between 1988 and 1992 Noncycler (n=41 045) Mild Cycler (n=3142) Severe Cycler (n=695)  **Effect estimates** Cycling Between 1972 and 1992  Noncycler Deaths, No. 319 Person-years 315 836  HR (95% CI) 1 [Reference]  Mild Cycler Deaths, No. 65  Person-years 81 874  HR (95% CI) 0.89 (0.67-1.18)  Severe Cycler Deaths, No. 41  Person-years 34 663 HR (95% CI) 1.08 (0.75-1.56)  Cycling Between 1988 and 1992 Noncycler Deaths, No. 384  Person-years 395 312  HR (95% CI) 1 [Reference]  Mild Cycler Deaths, No. 29 Person-years 12  HR (95% CI) 1.11 (0.75-1.64)  Severe Cycler Deaths, No. 12 Person-years 6641 HR (95% CI) 1.65 (0.89-3.05)  **Significant trends** women who were severe cyclers from 1988 to 1992 were almost 3 times more likely than noncyclers to die from cardiovascular events during the follow-up period   **Reported limitations** self-reported information **Source of funding** | |
| **Authors:** [Fogelholm M](http://www.ncbi.nlm.nih.gov/pubmed?term=Fogelholm%20M%5BAuthor%5D&cauthor=true&cauthor_uid=10968728), [Kujala U](http://www.ncbi.nlm.nih.gov/pubmed?term=Kujala%20U%5BAuthor%5D&cauthor=true&cauthor_uid=10968728), [Kaprio J](http://www.ncbi.nlm.nih.gov/pubmed?term=Kaprio%20J%5BAuthor%5D&cauthor=true&cauthor_uid=10968728), [Sarna S](http://www.ncbi.nlm.nih.gov/pubmed?term=Sarna%20S%5BAuthor%5D&cauthor=true&cauthor_uid=10968728) **Year:** 2000 **Citation:** [Obes Res.](http://www.ncbi.nlm.nih.gov/pubmed/10968728) 2000;8(5):367-373  **Country of study:** Finland **Aim:** To assess influence of lifestyle on 10-year weight change **Study design:** Longitudinal **Quality score: +**  **Applicability: +** | | | | | | | | | | | | | |
| **Source population:**  *large cohort originally comprising former top male athletes (n=2675) and untrained referents  *2062 out of 2535 (81%) surviving subjects completed baseline questionnaire in 1985 and 1670 out of 2114 completed follow-up questionnaire in 1995 | | **Study population:**  *analysis restricted to **1143** Finnish participants who completed baseline and follow-up questionnaires and with complete data  *participants were ages 36-88 years at baseline  *Follow-up:*  *10 yrs (1985-95)  **Exclusion:**  *analysis restricted to subjects without malignant cancer or diabetes  **Attrition:**  - | | | | **Exposures:**  *****self-reportedsmoking, alcohol use,  dietary habits, leisure physical activity  *smoking categories: smoking, non-smoking, quit or started smoking  *alcohol consumption frequencies assessed; alcohol content of beer, wine, and spirits assumed to be 4, 9.5, and 32g/litre, respectively (subjects classified into intake quartiles ranging from low to high alcohol use)  *frequency of fruit and vegetable consumption assessed (healthy diet defined by presence of at least 2 of the following: fruit and vegetables intake at least 3 times/day; use of margarine; use of skimmed milk)  *frequency, duration, and intensity of physical activity assessed, with categories ranging from low to high | | **Outcomes:**  *self-administered questionnaire captured 10-year weight change | | **Analysis:**  *step-wise linear regression was used to assess influence of lifestyle on 10-year weight change  **Confounders:**  *****age, chronic diseases, occupation, present  occupational activity, living conditions, former athletic status | | **Results:**  *****being a smoker (beta= -1.59, SE=0.48), and increased physical activity (beta=-1.27, SE=0.54) were significantly (*p<*0.05) associated with weight loss  **Limitations:**  *residual confounding  *self-reported body weight (may be underestimated) and physician-diagnosed diseases  *cohort was not a random, representative population sample  **Source of funding:**  *Academy of Finland | |
| **Authors:** Gerber Y, Myers V, Goldbourt U  **Year:** 2012  **Citation:** Am J Epidemiol. 2012;175(10):1006–1012  **Country of study:** Israel  **Aim of study:** Assess survival and life expectancy according to changes in smoking intensity  **Study design:** Prospective cohort study  **Quality score: (++, + or -):**  **External validity score: (++, + or -):** | | | | | | | | | | | | | |
| **Number of people**  10,059  **Demographics** Not reported | | **Number of people** 4,633 **Characteristics**  No.% Mean (SD)  **Increased Smoking Intensity.** Age, years 50.1 (6.5); Lowest SES category 126 35; Systolic blood pressure, mm Hg 134 (20); Diastolic blood pressure, mm Hg 85 (12); Cholesterol, mg/dL 207 (42); Body mass index 25.2 (3.4); Leisure-time physical activity; None 235 67%; Light 48 14%; Light daily 45 13%; Heavy 22 6%; Diabetes 23 6%; Known coronary heart disease 20 6%; Intermittent claudication 8 2%  **Maintained Smoking Intensity.** Age, years 50.6 (6.7); Lowest SES category 780 26; Systolic blood pressure, mm Hg 136 (21); Diastolic blood pressure, mm Hg 86 (12); Cholesterol, mg/dL 209 (39); Body mass index 25.6 (3.5); Leisure-time physical activity; None 1,876 63; Light 417 14; Light daily 487 16; Heavy 204 7; iabetes 178 6 ; Known coronary heart disease 249 8 ; Intermittent claudication 117 4  **Reduced Smoking Intensity.** Age, years 50.2 (6.4); Lowest SES category 255 33; Systolic blood pressure, mm Hg 136 (21); Diastolic blood pressure, mm Hg 85 (12); Cholesterol, mg/dL 210 (38); Body mass index 25.3 (3.4); Leisure-time physical activity; None 490 63; Light 120 15; Light daily 115 15; Heavy 57 7; Diabetes 52 7; Known coronary heart disease 68 9; Intermittent claudication 45 6  **Quit.** Age, years 51.9 (7.2); Lowest SES category 104 23; Systolic blood pressure, mm Hg 139 (22); Diastolic blood pressure, mm Hg 87 (12); Cholesterol, mg/dL 210 (40); Body mass index 26.4 (3.2); Leisure-time physical activity; None 287 62; Light 75 16; Light daily 74 16; Heavy 26 6; Diabetes 36 8; Known coronary heart disease 71 15; Intermittent claudication 26 6  **P Value.** Age, years <0.001; Lowest SES category <0.001; Systolic blood pressure, mm Hg 0.02; Diastolic blood pressure, mm Hg 0.004; Cholesterol, mg/dL 0.63; Body mass index <0.001; Leisure-time physical activity 0.59; Diabetes 0.50; Known coronary heart disease <0.001; Intermittent claudication 0.01  **Location**  Tel Aviv, Haifa, and Jerusalem, Israel  **Recruitment strategy** Stratified sampling  **Length of follow-up**  median follow-up of 26 (quartiles 1–3: 16–35) years  **Response rate and loss to follow-up** 87% of participants died  **Eligible population** Israeli working men; civil servants and municipal employees  **Excluded populations**  excluding 96 participants who stopped cigarette smoking and switched to cigar or pipe smoking. 87% of participants died | | | | **Relevant exposures** Smoking behaviour, physical activity **Time** 1963 **Measurement of exposure** Smoking behavior was self-reported. participants were asked to choose one of 5 smoking status groups: never smoker, past smoker, 1–10, 11–20, or more than 20 cigarettes per day.  Physical activity during leisure time was determined via personal interview, with subjects reporting their physical activity outside working hours | | **Outcomes** Outcomes were time to 1) all-cause death and 2) cause-specific death, dichotomized into CVD and non-CVD.  **outcome measurement** Information on death was derived from the Israeli Mortality Registry  **Time**  all-cause death (1965–2005) and cause-specific death (1965–1997) | | **Analysis strategy** Cox proportional hazards regression models  **Confounders** age, socioeconomic status, and smoking intensity in 1963, systolic blood pressure, blood cholesterol, body mass index, leisure-time physical activity, diabetes, known coronary heart disease, and intermittent claudication | | **Number** Increased (n=358; %=8) Maintained (n=3,061; %=65) Reduced (n=787; %=17) Quit (n=472; %=10)  **Effect estimates** All-cause mortality  HR 95% CI Increased 1.14 0.99, 1.32  Maintained 1 Referent  Reduced 0.85 0.77, 0.95  Quit 0.78 0.69, 0.89  Ptrend <0.001  CVD mortality Increased 1.14 0.92, 1.41  Maintained 1 Referent  Reduced 0.77 0.66, 0.94  Quit 0.84 0.70, 1.05  Ptrend 0.01  Non-CVD mortality Increased 1.05 0.88, 1.25  Maintained 1 Referent  Reduced 0.98 0.87, 1.10  Quit 0.90 0.77, 1.05  Ptrend 0.19  **Significant trends** survival benefit associated with smoking reduction was mostly evident among heavy smokers and for cardiovascular disease mortality  **Reported limitations  Author** no information is available on smoking habits throughout follow-up. smoking was self-reported. Couldn’t control for dietary and physical activity patterns. male-only cohort  **Reviewer** Difference in time for outcome  **Source of funding** supported by PL 480 counterpart funds, research agreement no. 375106. The Fund for Basic Research from the Israeli Academy of Sciences supported the mortality follow-up from 1970 to 1978. | |
| **Authors:** Guallar-Castillon P, Rodríguez-Artalejo F, Tormo MJ, Sánchez MJ, Rodríguez L, et al.  **Year:** 2012  **Citation:** Nutr Metab Cardiovasc Dis. 2012;22:192e199  **Country of study:** Spain  **Aim of study:** Assess the association between major dietary patterns and the risk of coronary heart disease  **Study design:** Multi-centre prospective cohort study  **Quality score: (++, + or -):**  **External validity score: (++, + or -):** | | | | | | | | | | | | | |
| **Number of people**  519,978 (366,521 women and 153,457 men)  **Demographics** Not reported | | **Number of people** 40,757 persons **Characteristics**  **Westernized pattern**  **Quintile 1 (lowest).** Age (years) 50.9; Body mass index (kg/m2) 28.6; Waist circumference (cm) 92.3; No formal education (%) 38.3; Current smokers (%) 18.2; Sedentary at work (%) 24.2; Physical activity at home (METs h/wk) 68.2; Physical activity in leisure time (METs h/wk) 31.1  **Quintile 3.** Age (years) 48.6; Body mass index (kg/m2) 28.2; Waist circumference (cm) 91.6; No formal education (%) 33.9; Current smokers (%) 22.7; Sedentary at work (%) 20.9 17.5; Physical activity at home (METs h/wk) 69.9; Physical activity in leisure time (METs h/wk) 28.3  **Quintile 5 (highest).** Age (years) 47.0; Body mass index (kg/m2) 28.1; Waist circumference (cm) 92.1; No formal education (%) 31.9; Current smokers (%) 28.5; Sedentary at work (%) 17.5; Physical activity at home (METs h/wk) 69.9; Physical activity in leisure time (METs h/wk) 25.9 **Evolved Mediterranean pattern Quintile 1 (lowest).** Age (years) 48.0; Body mass index (kg/m2) 28.0; Waist circumference (cm) 91.0; No formal education (%) 33.6; Current smokers (%) 28.8; Sedentary at work (%) 19.4; Physical activity at home (METs h/wk) 69.2; Physical activity in leisure time (METs h/wk) 26.7 **Quintile 3.** Age (years) 48.7; Body mass index (kg/m2) 28.3; Waist circumference (cm) 91.8; No formal education (%) 34.7; Current smokers (%) 23.1; Sedentary at work (%) 22.0; Physical activity at home (METs h/wk) 69.4; Physical activity in leisure time (METs h/wk) 28.7  **Quintile 5 (highest).** Age (years) 49.6; Body mass index (kg/m2) 28.4; Waist circumference (cm) 92.6; No formal education (%) 34.3; Current smokers (%) 18.1; Sedentary at work (%) 21.0; Physical activity at home (METs h/wk) 68.0; Physical activity in leisure time (METs h/wk) 30.6  **Location**  Spain. 5 regions: Asturias, Gipuzkoa, Navarra, Granada and Murcia  **Recruitment strategy** invited to participate either by mail or in person  **Length of follow-up**  median follow-up of 11 years  **Response rate and loss to follow-up** followed-up until December 31, 2004.  **Eligible population** Participant eligibility within each cohort was based on geographic or administrative boundaries. Used only the data from the Spanish cohort of the EPIC study  **Excluded populations**  193 for having CHD at baseline; 167 for an implausibly high or low dietary consumption, defined as 3 standard deviations from the mean of the cohort (<788 kcal/day or >5710 kcal/day); and 321 for lack of information on important variables such as date of CHD event (12), hypercholesterolemia (197), diabetes (71), hypertension (60) and smoking (22). | | | | **Relevant exposures** dietary pattern, educational level, smoking, and physical activity  **Time** subject’s age at recruitment and exit time as the age at the occurrence of a CHD event, death, or December 31, 2004 **Measurement of exposure** self-administrated questionnaires and interview | | **Outcomes** myocardial infarction and mortality  **outcome measurement** hospital discharge registers  CHD events were classified on the basis of symptoms, signs, biomarkers, and electrocardiogram   **Time**  between 1992 and 1996 | | **Analysis strategy** Cox regression models  **Confounders** age, sex, BMI, waist circumference, educational level, smoking, physical activity at work, physical activity at home, physical activity in leisure time, diabetes, hypertension, hypercholesterolemia, cancer, oral contraceptives, menopausal status, hormone replacement therapy, and total energy intake | | **Number** 606 definite CHD events (466 myocardial infarctions and 140 anginas requiring revascularization).  **Effect estimates** **Definite CHD events** **Westernized Pattern** Quintile 1) 1 Ref. Quintile 2) 0.96 (0.75-1.24) Quintile 3) 0.81 (0.61-1.09) Quintile 4) 0.98 (0.72-1.34) Quintile 5) 0.86 (0.60-1.24) P for trend 0.51 **Evolved Mediterranean Pattern** Quintile 1) 1 Ref. Quintile 2) 0.77 (0.61-0.98) Quintile 3) 0.64 (0.50-0.83) Quintile 4) 0.56 (0.43-0.73) Quintile 5) 0.73 (0.57-0.94) P for trend 0.0013 **Definite, possible and probable CHD events Westernized Pattern** Quintile 1) 1 Ref. Quintile 2) 1.02 (0.81-1.29) Quintile 3) 0.88 (0.67-1.15) Quintile 4) 1.04 (0.78-1.38) Quintile 5) 0.87 (0.62-1.22) P for trend 0.55 **Evolved Mediterranean Pattern** Quintile 1) 1 Ref. Quintile 2) 0.76 (0.61-0.95) Quintile 3) 0.64 (0.51-0.81) Quintile 4) 0.58 (0.46-0.74) Quintile 5) 0.72 (0.57-0.91) P for trend <0.001  **Significant trends** No association was found between the Westernized dietry pattern and CHD risk. A Mediterranean diet, as consumed in this study population, was associatedwith a lower risk of CHD.  **Reported limitations  Author** Interpretation of the dietary patterns obtained by factor analysis is subjective. Patterns derived depend on the number of foods included in the diet measurement instrument. Percentage of the variance explained by the two major dietary patterns was only modest. Diet was measured only at baseline. Residual confounding may persist  **Source of funding** Spanish Ministry of Health PI04-0257, PI06-0366, PI04-2342, PI04-1822, PI04-1821 and PI04-2188 from the “Instituto de Salud Carlos III”; RETIC (RD06/0020) of ISCIII Spanish Regional Governments of Andalusia, Asturias, Basque Country, Murcia and Navarra and the Catalan Institute of Oncology; EL-G has a “Ramo´n y Cajal” contract from the Ministry of Education. | |
| **Authors:** Haapanen-Niemi N, Miilunpalo S, Pasanen M, Vuori I, Oja P, et al.  **Year:** 2000  **Citation:** Int J Obes. 2000;24:1465-1474  **Country of study:** Finland  **Aim of study:** To investigate the independent associations and the possible interaction of BMI, leisure time physical activity and perceived physical fitness and functional capability with the risk of mortality.  **Study design:** Prospective 16 year follow-up study  **Quality score: (++, + or -):**  **External validity score: (++, + or -):** | | | | | | | | | | | | | |
| **Number of people**  6787  **Demographics** Not reported | | **Number of people** 1090 men and 1122 women   **Characteristics**  Men  Alive (n.882) %  Age (y) 35-44 48.1  45-54 37.0  55-63 15.0  Socioeconomic status Upper-level employee 19.7  Lower-level employee 13.2  Manual worker 54.2  Farmer or other own-account worker 11.6  Other 0.8  Housewife -  Missing data 0.6  Employment status Participant in work life 83.3  Not participant in work life 15.6  Housewife - Missing data 1.0  Marital status Married 82.2  Single 12.8  Widowed 1.1  Divorced, separated 3.6  Missing data 0.2  Perceived health Good or fairly good 44.0  Average 38.9  Poor or rather poor 16.9  Missing data 0.2  Smoking status Never smoked 27.9  Past smoker 37.2  Current smoker 32.5  Missing data 2.4  Alcohol consumption (g=day) 0 16.1  0.1-12 54.1  >12 29.4  Missing data 0.5   Deceased (n.208) % Age (y) 35-44 20.7  45-54 35.6  55-63 43.8  Socioeconomic status Upper-level employee 12.5  Lower-level employee 8.2  Manual worker 60.6  Farmer or other own-account worker 16.3  Other 01.9  Housewife - Missing data 0.5  Employment status Participant in work life 50.5  Not participant in work life 47.1  Housewife - Missing data 2.4  Marital status Married 80.8  Single 14.4 Widowed - Divorced, separated 4.8 Missing data - Perceived health Good or fairly good 24.0  Average 34.6  Poor or rather poor 41.3  Missing data - Smoking status Never smoked 13.5  Past smoker 33.2  Current smoker 47.1  Missing data 6.3  Alcohol consumption (g=day) 0 15.9  0.1-12 47.1  >12 35.1  Missing data 1.9   Women Alive (n.1035) %  Age (y) 35-44 44.2  45-54 35.7  55-63 20.2  Socioeconomic status Upper-level employee 9.3  Lower-level employee 34.9  Manual worker 30.7  Farmer or other own-account worker 15.7  Other 1.0  Housewife 8.3  Missing data 0.2  Employment status Participant in work life 61.8  Not participant in work life 18.9  Housewife 18.6  Missing data 0.6  Marital status Married 78.1  Single 8.1  Widowed 8.5  Divorced, separated 5.0  Missing data 0.3 Perceived health Good or fairly good 43.5  Average 37.4  Poor or rather poor 19.0  Missing data 0.1  Smoking status Never smoked 75.9  Past smoker 9.1  Current smoker 13.6  Missing data 1.4  Alcohol consumption (g=day) 0 54.9  0.1-12 41.5  >12 2.4  Missing data 1.2   Deceased (n.87) % Age (y) 35-44 12.6 45-54 32.2 55-63 55.2 Socioeconomic status Upper-level employee 5.7 Lower-level employee 27.6 Manual worker 34.5 Farmer or other own-account worker 11.5 Other 5.7 Housewife 13.8 Missing data 1.1 Employment status Participant in work life 34.5 Not participant in work life 44.8 Housewife 18.4 Missing data 2.3 Marital status Married 63.2 Single 13.8 Widowed 17.2 Divorced, separated 5.7 Missing data - Perceived health Good or fairly good 23.0 Average 37.9 Poor or rather poor 39.1 Missing data - Smoking status Never smoked 63.2 Past smoker 10.3 Current smoker 23.0 Missing data 3.4 Alcohol consumption (g=day) 0 69.0 0.1-12 24.1 >12 5.7 Missing data 1.1  **Location**  medium-size industrial town and two rural municipalities in northeastern Finland  **Recruitment strategy** census data  **Length of follow-up**  16 y  **Response rate and loss to follow-up** 77.5% (nˆ=5259) 1y 88% 5y 84% 10y 85% 16 y 85%  **Eligible population** subjects aged 35 y and older (men, n=ˆ1340; women, nˆ=1500), who were 51 - 79 y of age at the end of the follow-up  **Excluded populations**  those having an initial BMI of less than 20.0 and those suffering from a disease or symptoms that totally prevented participation in LTPA | | | | **Relevant exposures** living habits, health behaviour, health status, functional capacity and sociodemographic background  **Time** 1980 **Measurment of exposure** self-administered questionnaire  LTPA was assessed with 23 questions concerning conditioning exercise, sports, physical recreation, different leisure time and household chores and active commuting to and from work  subjects were divided into high, moderate and low physical activity groups according to the index of total physical activity. For the men, the classes were designated as 0-1000, 1000.1-1900 and>1900 kcal per week, and for the women the respective categories were 0-800, 800.1-1500 and>1500 kcal per week  Physical fitness was assessed by three measures indicating perceived fitness and functional status | | **Outcomes** mortality  **outcome measurement** national census data  **Time**  September 1996 | | **Analysis strategy** Cox proportional hazards model  C**onfounders** age, marital and employment status, perceived health status, smoking and alcohol consumption | | **Number** 208 men and 87 women   **Effect estimates** Relative risks for all-cause-, CVD and CHD mortality for the men in 1980 - 1996 All causes  RR 95% CI P BMI 20.0-24.9 1.00  25.0-29.9 0.87 0.64 - 1.19 0.379 >30.0 1.06 0.67 - 1.69 0.796  LRa.1.1, df.2, P.0.577  Total LTPA energy expenditure index (kcal=week) High 1.00  Moderate 0.82 0.55 - 1.24 0.350  Low 1.20 0.82 - 1.76 0.349  LR.4.2, df.2, P.0.120  Single-item self-assessment of LTPA Vigorous activity at least once a week and some light activity 1.00  No or light intensity activity weekly 1.26 0.89 - 1.77 0.193 LR.1.7, df.1, P.0.187  Perceived physical fitness compared with age-mates Better 1.00  Similar 1.93 1.15 - 3.24 0.013  Worse 3.29 1.80 - 6.02 <0.001  LR.16.9, df.2, P<0.001  2 km walking ability No difficulties 1.00  At least some difficulties 1.62 1.05 - 2.50 0.028  LR.4.7, df.1, P.0.030  Climbing several flights of stairs No difficulties 1.00  At least some difficulties 1.47 0.97 - 2.23 0.070  LR.3.3, df.1, P.0.070  CVD RR 95% CI P BMI 20.0-24.9 1.00  25.0-29.9 0.87 0.56 - 1.35 0.541  >30.0 1.44 0.80 - 2.56 0.223  LR.2.6, df.2, P.0.274  Total LTPA energy expenditure index (kcal=week) High 1.00  Moderate 0.94 0.52 - 1.71 0.846  Low 1.69 0.97 - 2.93 0.063 LR.6.5, df.2, P.0.039 Single-item self-assessment of LTPA Vigorous activity at least once a week and some light activity 1.00  No or light intensity activity weekly 1.61 0.98 - 2.64 0.058  LR.3.8, df.1, P.0.050  Perceived physical fitness compared with age-mates Better 1.00  Similar 2.39 1.09 - 5.22 0.029  Worse 34.37 1.80 - 10.62 0.001  LR.12.5, df.2, P.0.002 2 km walking ability No difficulties 1.00  At least some difficulties 1.25 0.71 - 2.22 0.438  LR.0.6, df.1, P.0.441  Climbing several flights of stairs No difficulties 1.00  At least some difficulties 1.85 1.04 - 3.30 0.036  LR.4.5, df.1, P.0.034  CHD RR 95% CI P BMI 20.0-24.9 1.00  25.0-29.9 0.94 0.57 - 1.56 0.813 >30.0 1.23 0.61 - 2.50 0.564 LR.0.5, df.2, P.0.767 Total LTPA energy expenditure index (kcal=week) High 1.00  Moderate 0.88 0.44 - 1.76 0.709 Low 1.70 0.90 - 3.21 0.105 LR.5.8, df.2, P.0.056 Single-item self-assessment of LTPA Vigorous activity at least once a week and some light activity 1.00  No or light intensity activity 1.66 0.92 - 2.99 0.090 LR.3.1, df.1, P.0.079 Perceived physical fitness compared with age-mates Better 1.00  Similar 2.82 1.06 - 7.46 0.037 Worse 4.64 1.56 - 13.84 0.006 LR.9.1, df.2, P.0.011 2 km walking ability No difficulties 1.00  At least some difficulties 1.03 0.51 - 2.05 0.941 LR.0.005, df.1, P.0.941 Climbing several flights of stairs No difficulties 1.00  At least some difficulties 1.61 0.82 - 3.16 0.167 LR.1.9, df.1, P.0.164  Relative risks for all-cause- and CVD mortality for the women in 1980 - 1996 All causes  RR 95% CI P BMI 20.0-24.9 1.00  25.0-29.9 0.87 0.52 - 1.46 0.599 >30.0 1.35 0.76 - 2.41 0.310  LR.2.2, df.2, P.0.341  Total LTPA energy expenditure index (kcal=week) High 1.00  Moderate 0.59 0.30 - 1.18 0.136  Low 1.27 0.69 - 2.34 0.440  LR.6.8, df.2, P.0.033  Single-item self-assessment of LTPA Vigorous activity at least once a week and some light activity 1.00 No or light intensity activity weekly 1.61 0.89 - 2.92 0.114  LR.2.7, df.1, P.0.101  Perceived physical fitness compared with age-mates Better 1.00  Similar 0.82 0.41 - 1.65 0.582  Worse 1.71 0.72 - 4.05 0.221  LR.5.9, df.2, P.0.054  2 km walking ability No difficulties 1.00  At least some difficulties 1.45 0.78 - 2.70 0.237  LR.1.4, df.1, P.0.243  Climbing several flights of stairs No difficulties 1.00  At least some difficulties 2.39 1.25 - 4.60 0.009  LR.7.2, df.1, P.0.007  CVD RR 95% CI P BMI 20.0-24.9 1.00  25.0-29.9 0.85 0.42 - 1.74 0.664 >30.0 1.36 0.60 - 3.07 0.459 LR.1.3, df.2, P.0.527 Total LTPA energy expenditure index (kcal=week) High 1.00  Moderate 0.43 0.16 - 1.16 0.093 Low 1.17 0.51 - 2.68 0.717 LR.6.2, df.2, P.0.046 Single-item self-assessment of LTPA Vigorous activity at least once a week and some light activity 1.00 No or light intensity activity weekly 4.68 1.41 - 15.57 0.012 LR.9.4, df.1, P.0.002 Perceived physical fitness compared with age-mates Better 1.00  Similar 0.82 0.32 - 2.16 0.693 Worse 1.89 0.57 - 6.27 0.299 LR.3.7, df.2, P.0.154 2 km walking ability No difficulties 1.00  At least some difficulties 1.25 0.53 - 2.90 0.611 LR.0.3, df.1, P.0.614 Climbing several flights of stairs No difficulties 1.00  At least some difficulties 3.38 1.22 - 9.41 0.020 LR.6.2, df.1, P.0.013  **Significant trends** BMI was not associated with the risk of death among men or women  **Reported limitations  Author** self-reported information about physical fitness and functional capability, LTPA and BMI  **Source of funding** Juho Vainio Foundation, The Yrjo Jahnsson Foundation, the Finnish Ministry of Education and partially from the Emil Aaltonen Foundation | |
| **Authors:** Halperin RO, Gaziano JM, Sesso HD  **Year:** 2008  **Citation:** Am J Hypertens. 2008;21:148-152  **Country of study:** USA  **Aim of study:** Evaluate the relationship between smoking status and incident hypertension  **Study design:** Randomized, double blind, placebo-controlled trial  **Quality score: (++, + or -):**  **External validity score: (++, + or -):** | | | | | | | | | | | | | |
| **Number of people**  22,071  **Demographics** 52.4 years (s.d. 8.9) 51.9% never smokered, 37.6% as past smokers, 6.6% as current smokers of <20 cigarettes/day, and 3.9% as current smokers of ≥20 cigarettes/day. | | **Number of people** 13,529 **Characteristics**  Not reported  **Location**  USA  **Recruitment strategy** Not reported  **Length of follow-up**  Median follow-up was 14.5 years, with a maximum follow-up of 20.5 years.  **Response rate and loss to follow-up** >99%  **Eligible population** male physicians  **Excluded populations**  Excluded participants who reported any past or current history of hypertension, or a SBP of ≥140 mm Hg, or a DBP of ≥90 mm Hg. Excluded all participants with missing information on either baseline hypertension status or smoking status | | | | **Relevant exposures** age, body mass index, alcohol consumption, exercise  **Time** Not reported  **Measurement of exposure** Categorised as never smoker, past smoker, or current smoker, and those currently smoking were also asked to provide the number of cigarettes smoked per day  alcohol consumption (rarely/never, monthly, weekly, daily)  exercise to sweat ≥ once per week (yes, no) | | **Outcomes** Hypertension  **outcome measurement** The diagnosis of hypertension was based on self-reported BP and/or the initiation of antihypertensive treatment.  **Time**  Not reported | | **Analysis strategy** Cox proportional hazards models  **Confounders** Adjusted for age, BMI, diabetes, any history of total cholesterol ≥240 mg/dl, alcohol use, exercise, parental history of myocardial infarction | | **Number** 4,904  **Effect estimates** Relative risks (RR) and 95% confidence intervals (CIs) of developing hypertension according to smoking status in 13,529 men Never 1.00 (ref)  Past 1.08 (1.01, 1.15) <20 cigarettes/day1.18 (1.01, 1.38)  ≥20 cigarettes/day 1.12 (0.99, 1.27)  All current smokers 1.15 (1.03, 1.27) P value 0.006  **Significant trends** The multivariate models that considered changes in smoking status showed increased RR (95% CI) of developing hypertension for current and past smokers of 1.14 (1.00, 1.25) and 1.08 (1.01, 1.15) respectively, and an increased RR of developing hypertension for new smokers at 2 years and new quitters at 2 years of 1.21 (0.96, 1.52) and 1.35 (1.08, 1.68), respectively.  **Reported limitations**  Not reported  **Source of funding** Supported by research grants CA 34944, CA 40360, and CA 97193 from the National Cancer Institute, and grants HL 26490 and HL 34595 from the National Heart Lung, and Blood Institute, National Institutes of Health, Bethesda, MD. Dr Halperin supported in part by a VA Special Ambulatory Fellowship award. | |
| **Authors:** Hamer M, Lavoie KL, Bacon SL  **Year:** 2013  **Citation:** Br J Sports Med. 2013:doi:10.1136/bjsports-2013-092993  **Country of study:** England  **Aim of study:** Examine the association between physical activity and healthy ageing  **Study design:** Prospective study  **Quality score: (++, + or -):**  **External validity score: (++, + or -):** | | | | | | | | | | | | | |
| **Number of people**  11,391  **Demographics** aged 63.7±8.9 years at baseline. Other details not reported | | **Number of people** 1,953 **Characteristics**  **Healthy ageing.** Age (years) 67.0±4.2; Men 300 (45.1); Smoking Never 307 (46.2), Previous 301 (45.3), Current 57 (8.6); Alcohol intake Daily 217 (32.6), At least once per week (but not daily) 220 (33.1), Rarely 186 (28.0), ever 42 (6.3); Physical activity Inactive 55 (8.3), Moderate (at least once per week) 345 (51.9), Vigorous (at least once per week) 265 (39.8); Marital status Married 479 (72.0), Single, never married 25 (3.8), Separated/divorced 56 (8.4), Widowed 105 (15.8); Wealth quintile 1 (lowest) 45 (6.8), 2 103 (15.5), 3 135 (20.3), 4 172 (25.9), 5 (highest) 210 (31.6)   **Unhealthy ageing.** Age (years) 62.9±9.5; Men 1169 (41.9); Smoking Never 1010 (36.2), Previous) 1264 (45.3), Current 515 (18.5), Alcohol intake daily 804 (28.8), At least once per week (but not daily) 891 (31.9), Rarely 822 (29.5), Never 272 (9.8); physical activity Inactive 598 (21.4), Moderate (at least once per week) 1347 (48.3), Vigorous (at least once per week) 844 (30.3); Marital status Married 1890 (67.8), Single, never married 141 (5.1), Separated/divorced 341 (12.2), Widowed 417 (15.0); Wealth quintile 1 (lowest) 458 (16.4), 2 538 (19.3), 3 601 (21.5), 4 586 (21.0), 5 (highest) 606 (21.7)  **Location**  England  **Recruitment strategy** multistage-stratified probability sampling with postcode sectors selected at the first stage and household addresses selected at the second stage  **Length of follow-up**  8 years of follow-up  **Response rate and loss to follow-up** 70% at the household level and 67% at the individual level  **Eligible population** Not reported  **Excluded populations**  Any participants with existing chronic disease at baseline and wave 2 were excluded. Between wave 1 and 5 2,158 died, 142 moved from UK, 109 institutiuonalised, 2809 non-response at wave 5. After wave 5 1586 had missing data and 1,133 had disease at baseline | | | | **Relevant exposures** Baseline demographics, cigarette smoking, current frequency of alcohol intake, marital status, wealth, physical activity,   **Time** 2010-2011  **Measurement of exposure** Self-reported physical activity, participants wore a GeneActiv device on their wrist for seven consecutive days | | **Outcomes** Healthy aging  **outcome measurement** defined as those participants who survived without developing major chronic disease, depressive symptoms, physical or cognitive impairment  Disease status was measured using self-reported physician diagnosis of major chronic diseases  Cognitive function was assessed objectively using a battery of widely used neuropsychological tests  Depressive symptoms were assessed using the eight-item Centre of Epidemiological Studies Depression scale  disability based on participants’ responses to questions on perceived difficulties in basic and instrumental activities of daily living  Physical functioning was objectively assessed using walking speed measured over an 8-foot long course  **Time**  2010-2011 | | **Analysis strategy** Multiple logistic regressions  **Confounders** adjustment for age, sex, smoking (never; previous; current), alcohol (daily; at least weekly; rarely; never), marital status (married; always single; separated; widowed), wealth quintile. | | **Number** 19.3% of the sample.  38.4% of the sample had developed a chronic illness, 17.6% reported depressive symptoms, 32% reported disability, 19.2% had cognitive impairment and 17.7% had inadequate gait speed  **Effect estimates** **OR (95% CI) for the association of physical activity and different components of healthy ageing over 8 years follow-up** Chronic disease Inactive 1.00 Moderate physical activity 0.78 (0.64 to 0.95) Vigorous physical activity 0.67 (0.54 to 0.84) p-trend 0.001 Depressive symptoms (CES-D>3) Inactive 1.00 Moderate physical activity 0.67 (0.53 to 0.85) Vigorous physical activity 0.51 (0.39 to 0.67) p-trend 0.001 Cognitive impairment Inactive 1.00  Moderate physical activity 0.88 (0.69 to 1.13) Vigorous physical activity 0.64 (0.48 to 0.85) p-trend 0.005 ADL/IADL Inactive 1.00 Moderate physical activity 0.57 (0.46 to 0.70) Vigorous physical activity 0.41 (0.33 to 0.52) p-trend <0.001  Impaired gait speed (<0.6 m/s) Inactive 1.00  Moderate physical activity 0.54 (0.40 to 0.72) Vigorous physical activity 0.41 (0.29 to 0.58) p-trend <0.001  **OR (95% CI) for the association of baseline physical activity and healthy ageing over 8 years follow-up (N=3454)** Inactive 1.00 Moderate physical activity 2.67 (1.95 to 3.64) Vigorous physical activity 3.53 (2.54 to 4.89) P trend <0.001 **OR (95% CI) for the association of physical activity change over wave 1–3 and healthy ageing at follow-up (N=3051)** Remained inactive 12/273 1.00 Became inactive 37/363 2.36 (1.19 to 4.68) Became active 34/275 3.37 (1.67 to 6.78) Remained active 521/2140 7.68 (4.18 to 14.09) p-trend <0.001 **Odds ratio (95% confidence interval) for the association of baseline physical activity and healthy ageing over 8 years follow up in clinical sub-sample.** OR (95% CI) Inactive 1.00  Moderate physical activity 2.13 (1.45 – 3.13)  Vigorous physical activity 2.85 (1.91 – 4.23)  **Significant trends** Becoming active (multivariate adjusted, 3.37, 1.67 to 6.78) or remaining active (7.68, 4.18 to 14.09) was associated with healthy ageing in comparison with remaining inactive over follow-up. Among the covariates, wealth and smoking predicted healthy ageing; compared with participants in the poorest quintile, those in the richest were more likely to be healthy agers (multivariate adjusted OR=2.81, 95% CI 1.93 to 4.10).  **Reported limitations  Author** assessment of physical activity change was crude. Chronic disease was based on self-report of physician diagnosis  **Source of funding** The funding is provided by the National Institute on Aging in theUSA (grants 2RO1AG7644-01A1 and 2RO1AG017644) and a consortium of UK government departments co-ordinated by the Office for National Statistics | |
| **Authors:** Happonen P, Voutilainen S, Salonen JT.  **Year:** 2004  **Citation:** J. Nutr. 2004;134:2381–2386  **Country of study:** Finland  **Aim of study:** Study the effect of coffee consumption on the incidence of nonfatal acute myocardial infarction or coronary death  **Study design:** Population-based cohort study  **Quality score: (++, + or -):**  **External validity score: (++, + or -):** | | | | | | | | | | | | | |
| **Number of people**  2682  **Demographics** Not reported | | **Number of people** 1971 **Characteristics**  Median coffee consumption, mL 556; Age, y 52.5 ± 5.3; Current smoker, % 30; Packyears among smokers, y 28.4±18.3; Physical activity, kJ/d 581±707; Ischemia in exercise electrocardiogram, %18; Family history of CHD, % 46; Diabetes, % 5.2; Income, thousand Finnish Marks 130±74; Alcohol intake, g/wk 73±115; Total fat intake, % energy 39.7±5.9; Saturated fat intake, % energy 19.4±4.1; Daily total energy intake, MJ 10.8±2.7; Daily tea consumption, mL 110±194; Daily total water intake, L 2.35±0.60; BMI, kg/m2 26.7±3.5  **Location**  Finland  **Recruitment strategy** Not reported  **Length of follow-up**  mean follow-up of 14 y  **Response rate and loss to follow-up** There were no losses to follow-up  **Eligible population** Not reported  **Excluded populations**  677 men with prevalent CHD at baseline | | | | **Relevant exposures** Coffee and diet, smoking and alcohol **Time** Not reported **Measurement of exposure** Consumption of foods and beverages was assessed with an instructed and interview-checked 4-d food recording by household measures  participant defined as a current smoker if he had ever smoked on a regular basis and had smoked within the past 30 d  Alcohol intake was measured with a recall of the frequency and usual amounts of alcoholic beverages consumed in the past 12 mo  Mean daily coffee intake was divided into 4 categories: 0 (nondrinkers), 1 to 375 mL (light drinkers), 376 to 813 mL (moderate drinkers), and 814 mL and over (heavy drinkers). | | **Outcomes** acute coronary events  **outcome measurement** obtained by computer linkage to the national hospital discharge registry; diagnostic information was collected from the hospitals and classified using identical diagnostic criteria  **Time**  Not reported | | **Analysis strategy** Cox proportional hazards regression  **Confounders** age, smoking, exercise ischemia, diabetes, income, serum insulin concentration, time, serum HDL and LDL cholesterol concentration, diastolic blood pressure, maximal oxygen uptake, and waist-hip ratio | | **Number** 269  **Effect estimates** Coffee intake category None (n _ 77) Number of events 1  Person-time, y 379  RR (95% CI) 0.66 (0.09–4.91)   Light (n _ 456) Number of events 24  Person-time, y 2187  RR (95% CI) 1.93 (1.12–3.32)   Moderate (n _ 1087) Number of events 29  Person-time, y 5316  RR (95% CI) 1.00  Heavy (n _ 351) Number of events 21 Person-time, y 1672 RR (95% CI) 2.15 (1.20–3.83)  **Significant trends** heavy coffee consumption increases the short-term risk of acute myocardial infarction or coronary death, independent of the brewing method or currently recognized risk factors for CHD. The hazard rate of acute coronary events was 43% (95% CI, 5 to 94%) higher in heavy coffee drinkers compared with moderate drinkers  **Reported limitations  Author** in Finland the consumption of decaffeinated coffee is minimal and our findings are confined to caffeine-containing coffee  **Source of funding** Academy of Finland (201688 and 80185, S.V.), the Ministry of Education of Finland, the city of Kuopio, and the National Heart, Lung, and Blood Institute of the United States. Additional support was provided by the Juho Vainio Foundation, the Finnish Cultural Fund/North Savo Fund, the Yrjo Jahnsson Foundation, and the Finnish Foundation for Medical Science | |
| **Authors:** Hara M, Sobue T, Sasaki S, Tsugane S  **Year:** 2002  **Citation:** Jpn. J. Cancer Res. 2002;93:6–14  **Country of study:** Japan  **Aim of study:** Update the evidence on the association between smoking and mortality  **Study design:** Population-based prospective study  **Quality score: (++, + or -):**  **External validity score: (++, + or -):** | | | | | | | | | | | | | |
| **Number of people**  54 498 subjects (27 063 men and 27 435 women)  **Demographics** Not reported | | **Number of people** 41 484 (19 950 men and 21 534 women) **Characteristics**  **Men never smokers.** Age (SD) 49.6 (5.6); Alcohol drinking, at least once per week (%) 59.1; Education, College or more (%) 15.2; Past history of Hypertension 15.1; Any drug for medication 20.9; Physical activity, 1+/week (%) 19.3; Body mass index (SD) 23.9 (2.9); Diet (daily, %) Fruit 30.0; Green vegetables 31.0; Yellow vegetables 16.6; White vegetables 34.0; Pickled vegetables 39.2; Soy products 37.1; Fresh fish 15.5; Red meat 26.2  **Men past smokers.** Age (SD) 50.2 (6.0); Alcohol drinking, at least once per week (%) 69.9; Education, College or more (%) 17.1; Past history of Hypertension 18.6; Any drug for medication 27.2; Physical activity, 1+/week (%) 21.3; Body mass index (SD) 23.9 (2.9); Diet (daily, %) Fruit 27.3; Green vegetables 30.3; Yellow vegetables 16.7; White vegetables 35.5; Pickled vegetables 41.7; Soy products 36.3; Fresh fish 14.7; Red meat 27.7  Men current smokers. Age (SD) 49.0 (6.0); Alcohol drinking, at least once per week (%) 71.7; Education, College or more (%) 13.4; Past history of Hypertension 13.1; Any drug for medication 19.8; Physical activity, 1+/week (%) 15.2; Body mass index (SD) 23.2 (2.9); Diet (daily, %) Fruit 20.8; Green vegetables 26.4; Yellow vegetables 12.3; White vegetables 32.6; Pickled vegetables 46.9; Soy products 34.5; Fresh fish 15.0; Red meat 28.9  **Women never smokers.** Age (SD) 49.6 (5.8); Alcohol drinking, at least once per week (%) 20.8; Education, College or more (%) 11.9; Past history of Hypertension 13.4; Any drug for medication 24.2; Physical activity, 1+/week (%) 14.5; Body mass index (SD) 23.6 (3.2); Diet (daily, %) Fruit 45.7; Green vegetables 36.7; Yellow vegetables 24.3; White vegetables 45.4; Pickled vegetables 53.1; Soy products 46.3; Fresh fish 19.8; Red meat 26.3  **Women past smokers**. Age (SD) 49.0 (6.3); Alcohol drinking, at least once per week (%) 41.3; Education, College or more (%) 19.5; Past history of Hypertension 19.3; Any drug for medication 33.8; physical activity, 1+/week (%) 18.2; Body mass index (SD) 24.0 (3.4); Diet (daily, %); fruit 37.1; Green vegetables 30.3; Yellow vegetables 19.3; White vegetables 35.9; Pickled vegetables 46.6; Soy products 35.6; Fresh fish 15.4; Red meat 31.6  **Women current smokers.** Age (SD) 48.6 (5.9); Alcohol drinking, at least once per week (%) 44.6; Education, College or more (%) 11.7; Past history of Hypertension 12.2; Any drug for medication 24.7; Physical activity, 1+/week (%) 13.1; Body mass index (SD) 23.2 (2.9); Diet (daily, %) Fruit 30.1; Green vegetables 31.6; Yellow vegetables 18.4; White vegetables 35.5; Pickled vegetables 46.3; Soy products 36.8; Fresh fish 14.9; red meat 30.7 **Location**  5 Public Health Center areas (Ninohe PHC in Iwate Prefecture, Yokote PHC in Akita Prefecture, Saku PHC in Nagano Prefecture, Ishikawa PHC in Okinawa Prefecture and Katsushika PHC in Tokyo Metropolitan area).  **Recruitment strategy** population registries  **Length of follow-up**  Until the date of death of the deceased, the last date when the survival status had been confirmed for censored cases, and the end of the study period (December 31, 1999  **Response rate and loss to follow-up** men (76.5%) and women (82.1%)  **Eligible population** Not reported  **Excluded populations**  Not reported | | | | **Relevant exposures** Smoking and alcohol  **Time** Not reported  **Measurement of exposure** Smoking status was initially classified as current, past, or never smoker  Usual alcohol intake was first reported as frequency of consumption in six categories: <1 day/month, 1–3 days/month, 1–2 days/week, 3–4 days/week, 5–6 days/week, and every day. | | **Outcomes** Death  **outcome measurement** death certificates  **Time**  Not reported | | **Analysis strategy** Cox’s proportional hazard model  **Confounders** adjusted for age and area, educational background, medication, past history of hypertension, sports in leisure time, dietary habits, alcohol habit and quintiled BMI | | **Number** 1014 men and 500 women died  **Effect estimates** **Risk Ratios According to Status of Cigarette Smoking for Selected Causes of Death** Men Past  All causes 1.02 (0.82 – 1.28) All Cancer 1.09 (0.77 – 1.54) All circulatory system disease 0.99 (0.67 – 1.43) Noncancer-noncirculatory system disease 0.97 (0.67 – 1.43) Current All causes1.55 (1.29 – 1.86) All Cancer 1.61 (1.20 – 2.15) All circulatory system disease 1.41 (0.97 – 2.03) Noncancer-noncirculatory system disease 1.61 (1.17 – 2.19) Women Past  All causes 1.27 (0.65 – 2.48) All Cancer 0.89 (0.28 – 2.81) All circulatory system disease2.51 (0.90 – 6.99) Noncancer-noncirculatory system disease 0.90 (0.22 – 3.68) Current All causes 1.89 (1.36 – 2.62) All Cancer 1.83 (1.14 – 2.95) All circulatory system disease 2.72 (1.45 – 5.07) Noncancer-noncirculatory system disease 1.39 (0.71 – 2.73)  **Effect from Cumulative Dose as Indicated by Pack-years among Current Smokers Compared with Never Smokers** RR (95%CI) Men Never smoker 1.00 Pack-year – 19 All causes 1.44 (1.12 – 1.84) All cancer 1.33 (0.88–2.00) All circulatory system disease 1.02 (0.60–1.73) Noncancer-noncirculatory system disease 1.89 (1.28–2.79) 20 – 29 All causes 1.56 (1.23 – 1.99)  All cancer 1.41 (0.94–2.10) All circulatory system disease 1.44 (0.90–2.31) Noncancer-noncirculatory system disease 1.82 (1.23–2.68) 30 + All causes 1.57 (1.28 – 1.93)  All cancer 1.83 (1.34–2.51) All circulatory system disease 1.41 (0.95–2.12) Noncancer-noncirculatory system disease 1.37 (0.96–1.95) Women Never smoker 1.00 Pack-year – 9 All causes 1.64 (0.98–2.72)  All cancer 1.03 (0.42–2.52) All circulatory system disease 3.37 (1.52–7.47) Noncancer-noncirculatory system disease 1.30 (0.47–3.57) 10 – 19 All causes 1.52 (0.80–2.88)  All cancer 0.64 (0.16–2.61) All circulatory system disease 2.12 (0.65–6.95) Noncancer-noncirculatory system disease 2.58 (1.10–6.02) 20 + All causes 2.61 (1.52–4.47)  All cancer 4.51 (2.45–8.30) All circulatory system disease 1.51 (0.35–6.57) Noncancer-noncirculatory system disease 0  **Effect from Dose for Number of Cigarettes and Age at Start of Smoking in Current Smokers** Number of cigarettes Men 1–19 All causes 1.00  All cancer 1.00 All circulatory system disease 1.00 Noncancer-noncirculatory system disease 1.00 20–29 All causes 0.95 (0.78–1.16)  All cancer 1.21 (0.89–1.64) All circulatory system disease 0.93 (0.61–1.41) Noncancer-noncirculatory system disease 0.73 (0.53–1.02) 30> All causes 0.96 (0.76–1.21)  All cancer 1.00 (0.68–1.47) All circulatory system disease 1.20 (0.76–1.88) Noncancer-noncirculatory system disease 0.78 (0.53–1.14) Women Number of cigarettes 1–19 All causes 1.00  All cancer 1.00 All circulatory system disease 1.00 Noncancer-noncirculatory system disease 1.00 20–29 All causes 1.27 (0.63–2.57)  All cancer 1.77 (0.60–5.17) All circulatory system disease 0.15 (0.01–1.56) Noncancer-noncirculatory system disease 1.28 (0.23–7.12) 30> All causes 2.20 (0.75–6.44)  All cancer 6.03 (1.36–26.64) All circulatory system disease 1.25 (0.11–13.76)  Noncancer-noncirculatory system disease 0  **Age at start of smoking** Men –19 All causes 1.00 All cancer 1.00 All circulatory system disease 1.00 Noncancer-noncirculatory system disease 1.00 20–24 All causes 0.81 (0.67–0.98) All cancer 0.86 (0.63–1.17) All circulatory system disease 0.80 (0.54–1.16) Noncancer-noncirculatory system disease 0.74 (0.54–1.03) 25+ All causes 0.69 (0.52–0.92) All cancer 0.77 (0.49–1.19) All circulatory system disease 0.51 (0.27–0.97) Noncancer-noncirculatory system disease 0.71 (0.44–1.14) Women –24 All causes 1.00 All cancer 1.00 All circulatory system disease 1.00 Noncancer-noncirculatory system disease 1.00 25+ All causes 1.00 (0.50–2.02) All cancer 0.63 (0.20–1.92) All circulatory system disease 1.00 (0.21–4.66) Noncancer-noncirculatory system disease 1.62 (0.23–11.61)  **Significant trends** Twenty-two percent of death from all causes, 25% of all cancer, and 17% of all circulatory system disease deaths, could be attributed to cigarette smoking in males, and 5%, 4%, and 11% in females, respectively.  **Reported limitations  Author** smoking status was determined only once at baseline  **Source of funding** Grants-in-Aid for Cancer Research and for the 2nd Term Comprehensive 10-Year Strategy for Cancer Control from the Ministry of Health, Labour and Welfare of Japan. Megumi Hara was an Awardee of a Research Resident Fellowship from the Foundation for Promotion of Cancer Research in Japan. | |
| **Authors:** Harmsen P, Lappas G, Rosengren A, Wilhelmsen L  **Year:** 2006  **Citation:** Stroke. 2006;37:1663-1667  **Country of study:** Sweden  **Aim of study:** To estimate the predictive value of risk factors for stroke measured in midlife  **Study design:** Intervention trial  **Quality score: (++, + or -):**  **External validity score: (++, + or -):** | | | | | | | | | | | | | |
| **Number of people**  7494 men  **Demographics** Not reported | | **Number of people** 7457 **Characteristics**  Not reported  **Location**  Göteborg, Sweden  **Recruitment strategy** Responders to a postal questionnaire  **Length of follow-up**  28 years  **Response rate and loss to follow-up** 75% of the sample  **Eligible population** Middle-Aged Men  **Excluded populations**  Women | | | | **Relevant exposures** psychological stress, smoking, high body mass, physical activity  **Time** 1970 to 1973.  **Measurement of exposure** Psychological stress was assessed by using 1 question on self-perceived stress and rated from 1 through 6  Physical leisure time activity was coded as: (1) sedentary, (2) moderate, or (3) strenuous and regular.  Socioeconomic class was coded according to the Swedish socioeconomic classification system: (1) unskilled and semiskilled workers, (2) skilled workers, (3) foremen in industrial production and assistant nonmanual employees, (4) intermediate nonmanual employees, and (5) employed and self-employed professionals, higher civil servants, and executives | | **Outcomes** Stroke  **outcome measurement** End points of first-ever stroke in participants free of previous stroke were registered from several sources.  **Time**  1998 | | **Analysis strategy** multiple Cox regression analyses  **Confounders** Not reported. Paper says model adjusted for all other factors | | **Number** 1019  **Effect estimates** Hazard Ratio (95% CI) systolic blood pressure quintile 5 vs quintile 2.04 (1.61–2.59) HypMed 1.56 (1.21–2.02) Previous transient ischemic attacks 1.74 (1.14–2.64) atrial fibrillation 3.43 (1.60–7.32) Stroke in either parent 1.09 (0.93–1.28) History of diabetes 3.21 (2.19–4.72) Coronary events in parent 1.07 (0.90–1.27) Smoking 1.33 (1.15–1.53) History of chest pain 1.24 (1.01–1.52) Psychological stress 1.25 (1.03–1.51) BMI quintile 5 vs quintile 1.26 (1.00–1.60) Low physical activity 1.11 (0.90–1.36) S-Chol quintile 5 vs quintile 1.08 (0.86–1.35) Social class low, 5 vs 1.02 (0.79–1.31) Age 1.08 (1.04–1.11)   **Significant trends** Age, diabetes, and high blood pressure were independently associated with increased risk of stroke  **Reported limitations**  Not reported  **Source of funding** Bank of Sweden Tercentenary Fund, the Swedish Research Council, and the Swedish Heart Lung Foundation | |
| **Authors**: He K, Hu FB, Colditz GA, Manson JE, Willett WC, et al. **Year:** 2004 **Citation:** Int J Obes Relat Metab Disord. 2004;28(12):1569-1574 **Country of study:** US **Aim:** To determine the association between intake of fruit and vegetables and the risk of obesity and long-term weight gain among middle-aged women **Study design:** Longitudinal **Quality score:** + **Applicability:** | | | | | | | | | | | | | |
| **Source population:**  *121,700 female registered nurses ages 30-55 years from 11 US states responded to a questionnaire in 1976 | | **Study population:**  *analysis restricted to **74,063** women*Follow-up:* *12 years **Exclusion:**  women with: *history of cardiovascular disease, cancer, diabetes *incomplete or implausible information  **Attrition:**  - | | | | **Exposures:**  *a semiquantitative food frequency questionnaire (FFQ) was used to assess the intake of standard units of *fruit and vegetable* items in the previous year (responses ranging from ‘never’ to ‘six or more times per day’); these were subsequently converted to average daily intake of food items for each participant  *changes in fruit and vegetable intake between 1984 (baseline) and 1994 were also computed *certain vegetables were grouped as cruciferous vegetables; dark and yellow vegetables; green leafy vegetables; other vegetables *validity of FFQ established with correlation coefficients ranging from 0.69-0.84  *vegetable/fruit consumption was divided into quintiles with the first quintile indicating the largest decrease in intake and the fifth quintile the largest increase in intake during follow-up  *median values of quintiles of changes in fruit and vegetable intake were used in linear trend tests | | **Outcomes:**  *self-reported body weight captured through questionnaire every other year; when self-reported weight was compared with measured weight, correlation was 0.96  *obesity defined as BMI >=30 kg/m2 and major weight gain as weight gain of 25 kg or more during follow-up | | **Analysis:**  *logistic regression was used to assess the association between changes in fruit and vegetable intake (divided into quintiles) during follow-up *general linear models with least-square means were used to estimate the mean difference of changes in BMI during follow-up  **Confounders:**  *age, year of follow-up, change in physical activity, change in cigarette smoking status, changes in alcohol consumption and caffeine intake, change in use of HRT, changes in energy-adjusted intake of saturated fat, polyunsaturated fat, monounsaturated fat, trans-unsaturated fatty acid, protein, and total energy and baseline BMI | | **Results:**  *of 65,294 non-obese participants at baseline, 6,530 became obese during follow-up  *669 women reported major weight gain during follow-up *women with the largest increase in intake of fruits (median change=+1.86 servings/day) had the lowest risk for obesity compared to women with the largest decrease in intake of fruits (OR=0.76, [0.68, 0.84]; trend test p-value: 0.0007) *women with the largest increase in intake of vegetables (median change=+2.80 servings/day) had the lowest risk for obesity compared to women with the largest decrease in intake of vegetables (OR=0.84, [0.75, 0.93]; trend test p-value= 0.0002) *women with the largest increase in intake of fruits and vegetables combined had the lowest risk for obesity and major weight gain compared to women with the largest decrease in intake of fruits and vegetables combined (OR=0.76, [0.69, 0.86], trend test p-value<0.0001; and OR=0.72, [0.55, 0.93], trend test –value=0.01, respectively)  *there appeared to be a decreasing trend in risk for obesity with increasing intake of vegetables (trend test p-value: 0.0002); same observation noted for major weight gain in relation to fruits and vegetables combined (trend test p-value: 0.01)   Baseline BMI stratification: *among overweight women, those reporting the greatest change in fruit and vegetable intake gained 0.76 kg less weight than women reporting the largest decrease in fruit and vegetable intake (among normal weight women at baseline, a change of 0.52 kg was observed)   Stratification by chronic disease presence: *among women with incident chronic disease (e.g. cancer, CVD, diabetes), those reporting the largest increase in intake of fruits and vegetables gained 0.77 kg less weight than did women with the largest decrease in intake (among women without chronic diseases, a difference of 0.57 kg was observed)  **Limitations:**  *if participants perceive themselves to be overweight, eating habits may change in accordance with dietary recommendations of past decades; this may result in an underestimation of association between intake of fruits and vegetables and weight gain * issues of reliability due to self-reported weight information  *residual confounding **Source of Funding:**  *National Institutes of Health | |
| **Authors**: Holmberg AH, Johnell O, Nilsson PM, Nilsson J, Berglund G, et al. **Year:** 2006 **Citation:** Osteoporos Int. 2006;17(7):1065-1077 **Country of study:** Sweden **Aim:** To investigate the risk factors for fractures among men and women in middle age **Study design:** Longitudinal **Quality score:** + **Applicability:** | | | | | | | | | | | | | |
| **Source population:**  *72% of invited population recruited for Malmo Preventive Project  *22,444 men ages 27-61 years and 10,902 women ages 28-58 years recruited during 1974-84 and 1977-92, respectively *mean response rate to core questions of baseline questionnaire was 98% for women and 72% for men, and approximately 100% for questions added during recruitment period | | **Study population:**  *regression analysis restricted to **11** women and **10** men  *women and men had 11-year and 16-year follow-up, respectively*Sociodemographics:**Baseline mean age of 44 years for men and 48 years for women*Follow-up:**men and women followed up for average of 19 and 15 years, respectively (men: 1974-1984 until 1999; women: 1977-1992 until 1999) **Exclusion:**  *missing data for 6,368 men *high-energy fractures and fractures caused by cancer or other bone diseases  **Attrition:**  - | | | | **Exposures:**  *self-reported smoking behaviour | | **Outcomes:**  **incident low-energy fractures and mortality* data was obtained through data linkage with hospital medical and radiological files using personal identification numbers *fractures were categorized as those of the forearm, vertebral, proximal humerus, or ankle  *outcomes restricted to fractures that were classified as *low-energy* or those resulting from falling from standing height or less (high-energy fractures or those caused by high-energy trauma were included in the non-fracture population)  *individuals were still classified under low-energy fracture even if they had previous or subsequent high-energy fracture  *the first fracture of each fracture type was assessed | | **Analysis:**  *Cox proportional hazard regression was used to assess risk factors for fragility fracture in middle age  **Confounders:**  *age, height, weight, >10 kg weight gain since age 30, skinfold, forced vital capacity, SBP, DBP, resting pulse, diabetes, triglycerides, cholesterol, y-glutamyl transferase, serum phosphate, serum creatinine, serum uric acid, blood hemoglobin, sedimentation rate, sick leave at present, chest pressure, poor self-rated health, poor appetite, premature awakening, hospitalization for mental disorder, physical activity, HRT, history of previous fracture | | **Results:**  *1,257 out of 1,292 women and 1,262 out of 1,505 men were affected by incident low-energy fractures  *among women and men, the total number of low-energy fracture were: 622 and 330 for forearm, 155 and 123 for proximal humerus, 160 and 168 were vertebral, 233 and 259 ankle, and 141 and 174 hip  *smoking was more common among men with vertebral, proximal humerus, and hip fractures *among women, smokers had a higher risk for vertebral fractures (RR=1.96, [1.47, 2.64]) than non-smokers *among men, smokers had a greater risk for low energy fractures (RR=1.25, [1.11, 1.39]), vertebral fractures (RR=1.85, [1.41, 2.42]), proximal humerus fractures (RR=1.58, [1.08, 2.33]), and hip fractures (RR=2.14, [1.51, 3.01]) than non-smokers  **Limitations:**  *healthy volunteer effect: participants may have fewer fractures than non-participants *incomplete data sets *residual confounding **Source of Funding:**  *The Swedish Research Council Project, The Kock Foundation, The Herman Järnhardt Foundation, Malmö University Hospital Funds, and regional research grants supported this study | |
| **Authors**: Holme I, Tonstad S, Sogaard AJ, Larsen PG, Haheim LL **Year:** 2007 **Citation:** BMC Public Health. 2007;12:7:154 **Country of study:** Norway **Aim:** To assess association between smoking or physical activity and metabolic syndrome  **Study design:** Longitudinal **Quality score:** + **Applicability:** | | | | | | | | | | | | | |
| **Source population:**  *16,209 men ages 40-49 years, born in 1923-32 and living in Oslo were invited and attended screening examination (represented 63% of this age group) | | **Study population:**  *men living in Oslo in 2000-01 were surveyed and men originally invited to participate in study in 1972-73 and also living in Oslo or Akershus were invited for re-examination  *of 10,328 eligible participants, 6410 (62%) men attended the baseline and follow-up *analysis restricted to **6382** men without baseline diabetes and with blood pressure measurements at baseline and follow-up   *Follow-up:* *28 years **Exclusion:**  men who: *were dead or had emigrated (n=1655) *lived outside Oslo and Akershus (n=1278) *had unknown addresses (n=2944)  **Attrition:**  *participants who answered question on LTPA smoked less and had a greater level of education *participants who attended in 2000 had lower body weight, height, systolic and diastolic blood pressure, total cholesterol, triglycerides and glucose values, and smoked less at baseline than non-participants | | | | **Exposures:**  *self-reported physical activity at work and leisure, and smoking habits measured in 1972-73  *leisure-time physical activity (LTPA) categorized as light (e.g., reading, watching TV); moderate (e.g., walking, bicycling – totally at least 4 hours/week); moderately vigorous (e.g., exercise, sports, heavy gardening – totalling at least 4 hours/week); vigorous (e.g., hard training several times/week) *reliability and validity of LTPA question has been established | | **Outcomes:**  *At baseline, physical measurements, blood pressure, and a blood sample were taken: *metabolic syndrome was defined as presence of at least 3 out of the following 5 criteria: 1) triglycerides >=1.7 mmol/l adjusted for the last meal, 2) glucose >= 6.1 mmol/l adjusted for the last meal, 3) BMI >=30 kg/m2, 4) blood pressure >= 135/85 mmHg, and 5) HDL cholesterol <1.03 mmol/l *smoking was categorized into ‘never’, ‘previous’, and ‘current’ smoker groups *diabetes definition included self-reported diabetes, antidiabetic medication, insulin use or non-fasting glucose >=11.1 mmol/l | | **Analysis:**  *logistic regression was used to assess association between smoking or physical activity and metabolic syndrome or diabetes **Confounders:**  *age, education | | **Results:**  *the odds of metabolic syndrome were higher for current smokers compared to those who never smoked (OR=1.29, [1.11, 1.51]) *the odds of metabolic syndrome were lower for those who reported vigorous, moderately vigorous, or moderate levels of LTPA in comparison with those who reported sedentary/light levels (OR=0.46, [0.28, 0.74]; OR=0.65, [0.54, 0.80]; OR=0.83, [0.71, 0.98]) *the odds of diabetes were lower for those who reported vigorous, moderately vigorous, or moderate levels of LTPA in comparison with those who reported sedentary/light levels (OR=0.28, [0.11, 0.71]; OR=0.68, [0.52, 0.91]; OR=0.75, [0.60, 0.94])  **Limitations:**  *if diseases started before year 2000, then biological, anthropometric indices and lifestyle factors may have been affected *imperfect measurement/definition of metabolic syndrome leading to possible underestimation of its prevalence *participants with metabolic syndrome at baseline were not excluded *residual confounding, such as, dietary habits and alcohol consumption **Source of Funding:**  *Norwegian Council for Cardiovascular Diseases of the Norwegian National Association for Public Health | |
| **Authors**: Holtermann A, Mortensen OS, Burr H, Søgaard K, Gyntelberg F, et al. **Year:** 2009 **Citation:** Scand J Work Environ Health. 2009;35(6):466-474 **Country of study:** Denmark **Aim:** To determine the association between physical activity and risk of ischemic heart disease and all-cause mortality **Study design:** Longitudinal **Quality score:** + **Applicability:** | | | | | | | | | | | | | |
| **Source population:**  *5249 Copenhagen male employees ages 40-59 years were invited and agreed to participate (87% response rate) and undergo an interview, a clinical examination, and complete a questionnaire in 1970-71 | | **Study population:**  *analysis restricted to **4952** men  *Follow-up:* *from 1970-71 to 2001 **Exclusion:**  men with: *history of myocardial infarction (n=74), angina pectoris (n=165), intermittent claudication (n=105) at baseline *missing answers (n=9)  *men who had emigrated during follow-up (n=14)  **Attrition:**  - | | | | **Exposures:**  *Physical activity at work* was assessed using questionnaire and participants were classified as: a) ‘low’: mainly sedentary/not walking around much at workplace, b) ‘moderate’: walking around quite a bit but not having to carry heavy items, c) ‘high’: walking around most of the time or undertaking heavy/strenuous physical work  *strenuous work was assessed with answer options of ‘seldom or never’, ‘occasionally’, and ‘often’  *a dichotomous variable was created to assess presence of physically demanding work  *another variable combined information on physical activity and strenuous work to assess physical work demands, with possible scores ranging from 2-6 (higher scores representing higher demands)  *Physical activity during leisure time:* *self-reported physical activity level during leisure time categorized as ‘low’ (mainly sedentary), ‘moderate’ (light activities for at least 4 hours/week), ‘high’ (active for at least 3 hours/week or taking part in high intensity activities several times/week) | | **Outcomes:**  *IHD mortality diagnoses between 1970-71 and 2001 obtained from national registers | | **Analysis:**  *Cox proportional hazard regression used to assess influence of physical activity on ischemic heart disease (IHD) and all-cause mortality **Confounders:**  *age, BMI, SBP, DBP, treatment of diabetes or hypertension, alcohol use, smoking, social class | | **Results:**  *591 died from IHD during study period  *Among men with moderate physical work demands:*  *the risk of IHD mortality was significantly lower for those with a high level of physical activity during leisure time (HR=0.37, [0.19, 0.70]) compared to those with a low level of physical activity during leisure  *the risk for all-cause mortality was significantly lower for those with a moderate and high level of physical activity during leisure time (HR=0.82, [0.71, 0.94]; and HR=0.64, [0.50, 0.81], respectively) compared to those with a low level of physical activity during leisure  *the risk for all-cause mortality was significantly lower for those with moderate and high (combined) physical activity during leisure time (HR=0.80, [0.70, 0.92]) compared to those with low levels of physical activity reported during leisure time  **Limitations:**  *self-reported information resulting in possible misclassification *misclassification of exposure due to lack of continuous exposure data and repeated measures of exposure during follow-up *unknown if results also apply to women, people of different races, ages, with varying levels of physical fitness, or from rural areas (generalizability issues) **Source of Funding:**  *none reported | |
| **Authors**: Holtzman RE, Rebok GW, Saczynski JS, Kouzis AC, Wilcox Doyle K, et al. **Year:** 2004 **Citation:** J Gerontol B Psychol Sci Soc Sci. 2004;59(6):278-284 **Country of study:** US **Aim:** To determine the influence of social networks on cognitive change **Study design:** Longitudinal **Quality score:** ++ **Applicability:** | | | | | | | | | | | | | |
| **Source population:**  *4,238 participants were recruited for wave 1 in 1981 using probability sampling methods  *of 4,238 people, 3,481 were interviewed at wave 1 and 1,920 (73% of survivors) were re-interviewed in 1993-96 at wave 3 | | **Study population:**  *analysis restricted to **354** out of 881 participants with age >= 50 years and MMSE >=28 at wave 1  **Exclusion:**  *107 with missing longitudinal data  *88 had missing MMSE data at wave 3  *5.4% of 354 people with missing cross-sectional data at wave 3  *people with MMSE <28  **Attrition:**  *420 lost to follow-up *non-assessed participants were older, had fewer years of formal education, were less likely to be female, and had lower baseline MMSE scores compared to assessed participants | | | | **Exposures:**  *social network included network size, frequency of contact, and emotional support  *Network size at waves*  *1 and 3:*  *the number of relatives, family members, friends and neighbours with whom the respondent kept in touch with via phone or visits, with corresponding scores assigned that were subsequently summed (range of possible scores of 0-10)  *Frequency of contact at wave 3*  *frequency of contact by phone or getting together with relatives or family and friends or neighbours with the range of possible scores of 0-10  *Emotional support at wave 3*  *support from partners, relatives, and friends was assessed; scores were subsequently assigned and summed with a possible range of 0-27 | | **Outcomes:**  *cognitive change between wave 3 and wave 1 was assessed using MMSE | | **Analysis:**  *simultaneous linear or logistic regression was used to determine whether interactions in larger networks at wave 1 are associated with MMSE change between wave 1 and wave 3  **Confounders:**  *cerebrovascular disease or risk, age, education, depression, race, gender, physical disability, alcohol use disorder | | **Results:**  *mean MMSE at wave 3 was 26.5 *a linear effect was observed for baseline network size (p=0.006, effect size=0.06); also, less increase - more decrease in network size is associated with decreased wave 3 MMSE (p=0.03, effect size=-0.06) *top tertiles of interpersonal activity and emotional support (paired as categorical variables) were significantly related to MMSE scores (activity betas=0.13 and 0.12, p <=0.04; support betas 0.16 and 0.17, p<=0.01)  *more frequent contact in larger networks and higher levels of emotional support positively influence cognitive change  **Limitations:**  *cannot assess whether decline in MMSE is due to regression to the mean *if follow-up period is too short, directionality confound may exist (particularly given the long prodromal period before clinical symptoms in Alzheimer’s begin to manifest) *generalizability may be restricted as assessed participants were healthier, better educated, younger, and with larger networks than non-assessed individuals  **Source of Funding:**  *National Institutes of Mental Health | |
| **Authors:** Hu G, Qiao Q, Silventoinen K, Eriksson JG, Jousilahti P, et al.  **Year:** 2003  **Citation:** Diabetologia. 2003;46:322–329  **Country of study:** Finland  **Aim of study:** Examine the relationship of occupational, commuting and leisure-time physical activity with the incidence of Type 2 diabetes  **Study design:** Prospective cohort  **Quality score: (++, + or -):**  **External validity score: (++, + or -):** | | | | | | | | | | | | | |
| **Number of people**  21 630  **Demographics** Not reported | | **Number of people** 6898 men and 7392 women **Characteristics**  Men Physical activity  Light  Participants n 2688  Age at baseline 50.9±8.8  BMI 27.0±3.9  Education 10.2±4.5  Obesity 20.3  Smoking 31.8  Moderate  Participants n 1563  Age at baseline 46.5±7.9  BMI 26.9±3.7  Education 10.1±3.7  Obesity 18.1  Smoking 23.6  Active  Participants n 2647  Age at baseline 47.5±8.0  BMI 26.9±3.6 Education 7.8±2.2  Obesity 17.8  Smoking 33.6  p value for trend  Age at baseline <0.001 BMI >0.2 Education <0.001 Obesity 0.056 Smoking <0.001  Women Physical activity  Light  Participants n 3255  Age at baseline 51.0±9.2  BMI 26.5±5.1  Education 9.8±3.9  Obesity 21.6  Smoking 13.6  Moderate  Participants n 2357  Age at baseline 47.2±8.0  BMI 26.1±4.2  Education 10.1±3.8  Obesity 16.0  Smoking 12.9  Active  Participants n 1780  Age at baseline 47.9±7.9  BMI 27.0±4.7  Education 8.4±2.6  Obesity 24.2  Smoking12.9  p value for trend  Age at baseline <0.001 BMI <0.001 Education <0.001 Obesity <0.001 Smoking >0.2  **Location**  North Karelia and Kuopio, and the Turku-Loimaa region in southwestern Finland  **Recruitment strategy** Not reported  **Length of follow-up**  mean follow-up of 12 years  **Response rate and loss to follow-up** The participation rate varied by year from 74% to 88%  **Eligible population** General population 25 to 64 years of age  **Excluded populations** subjects diagnosed with coronary heart disease or stroke (n=590), subjects with known diabetes (n=435) at baseline, and subjects with incomplete data on all required factors or on physical activity (n=1355) | | | | **Relevant exposures** physical activity and smoking habits **Time** through the end of 1998 or until death  **Measurement of exposure** Occupational physical activity according to the following three categories: (i) ‘light’ was physically very easy, sitting office work, e.g. secretary; (ii) ‘moderate’ was work including standing and walking, e.g. store assistant; (iii) ‘active’ was work including walking and lifting, or heavy manual labour. subjects asked whether they walked, rode a bicycle, or used motorized transportation to and from work as well as the daily duration of this activity | | **Outcomes** Type 2 diabetes  **outcome measurement** from the National Hospital Discharge Register and the National Social Insurance Institution’s Register  **Time**  Not reported | | **Analysis strategy** Cox proportional hazards model  **Confounders** age, study year, education, systolic blood pressure, smoking, the other two types of physical activity, BMI, and sex | | **Number** 373  **Effect estimates** Occupational physical activity  No. of new cases Person- years Adjusted hazards ratios (95% confidence intervals) Men  Light 97 29216 1.00 Moderate 32 18874 0.67 (0.44–1.01) Active 71 32955 0.73 (0.52–1.02) p value for trend 0.075 Women  Light 102 38034 1.00 Moderate 31 29310 0.72 (0.46–1.12) Active 40 22740 0.78 (0.52–1.18) p value for trend 0.267 Men and women combined  Light 199 67250 1.00 Moderate 63 48184 0.70 (0.52–0.96) Active 111 55695 0.74 (0.57–0.95) p value for trend 0.020  Commuting physical activity Adjusted hazards ratios (95% confidence intervals)  Men  0 min 1.00 1–29 min 1.00 (0.71–1.42) ≥30 min 0.75 (0.46–1.23) p value for trend 0.501 Women  0 min 1.00 1–29 min 0.94 (0.63–1.42) ≥30 min 0.57 (0.34–0.96) p value for trend 0.105 Men and women combined  0 min 1.00 1–29 min 0.96 (0.74–1.25) ≥30 min 0.64 (0.45–0.92) p value for trend 0.048  Leisure-time physical activity  Adjusted hazards ratios (95% confidence intervals) Men  Low 1.00 Moderate 0.78 (0.57–1.06) High 0.84 (0.52–1.37) p value for trend 0.282 Women  Low 1.00 Moderate 0.81 (0.58–1.15) High 0.85 (0.43–1.66) p value for trend 0.49 Men and women combinedb  Low 1.00 Moderate 0.81 (0.64–1.02) High 0.84 (0.57–1.25) p value for trend 0.186  **Significant trends** The multivariate-adjusted hazards ratios of diabetes with none, 1 to 29, and more than 30 min of walking or cycling to and from work were 1.00, 0.96, and 0.64 (p=0.048 for trend). The multivariate-adjusted hazards ratios of diabetes for low, moderate, high levels of leisure-time physical activity were 1.00, 0.67, and 0.61 (p=0.001 for trend); after additional adjustment for BMI, the hazards ratio was no longer significant  **Reported limitations  Author** Self-report of physical activity. Did not carry out a glucose tolerance test in the baseline and follow-up  **Source of funding** Finnish Academy (grants 38387, 46558, 52342, 53585, 76502, 77618) | |
| **Authors:** Hu G, Bidel S, Jousilahti P, Antikainen R, Tuomilehto J  **Year:** 2007  **Citation:** Mov Disord. 2000;22(15):2242–2248  **Country of study:** Finland  **Aim of study:** Examine the association of coffee and tea consumption with the risk of incident Parkinson’s disease  **Study design:** Independent cross-sectional population surveys  **Quality score: (++, + or -):**  **External validity score: (++, + or -):** | | | | | | | | | | | | | |
| **Number of people**  29,890  **Demographics** Not reported | | **Number of people** 29,335 **Characteristics**  Coffee consumption Men 0  Participants (n) 891 Age (yr) 44.8 (13.0) Body mass index (kg/m2) 26.2 (3.9) Education (yr) 10.9 (4.4) Low leisure time physical activity (%) 26.4 Tea drinker (%) 66.0 Current smoker (%) 18.3 Alcohol drinker (%) 56.5  1–4  Participants (n) 5,583 Age (yr) 47.6 (12.8) Body mass index (kg/m2) 26.6 (3.8) Education (yr) 10.6 (4.1) Low leisure time physical activity (%) 23.2 Tea drinker (%) 50.3 Current smoker (%) 26.9 Alcohol drinker (%) 67.5  5 Participants (n) 7,819 Age (yr) 45.4 (11.4) Body mass index (kg/m2) 26.8 (3.9) Education (yr) 9.7 (3.7) Low leisure time physical activity (%) 29.5 Tea drinker (%) 19.7 Current smoker (%) 46.3 Alcohol drinker (%) 61.2  P-trend Age (yr) <:0.001 Body mass index (kg/m2) <:0.001 Education (yr) <:0.001 Low leisure time physical activity (%) <:0.001 Tea drinker (%) <:0.001 Current smoker (%) <:0.001 Alcohol drinker (%) <:0.001   Women 0  Participants (n) 1,022  Age (yr) 40.4 (12.8) Body mass index (kg/m2) 25.6 (4.8) Education (yr) 11.1 (4.1) Low leisure time physical activity (%) 30.7 Tea drinker (%) 70.8 Current smoker (%) 11.2 Alcohol drinker (%) 37.3  1–4  Participants (n) 7,439  Age (yr) 46.4 (12.4) Body mass index (kg/m2) 25.8 (4.9) Education (yr) 11.0 (4.0) Low leisure time physical activity (%) 29.0 Tea drinker (%) 50.3 Current smoker (%) 14.7 Alcohol drinker (%) 44.7  5 Participants (n) 6,581 Age (yr) 45.4 (10.8) Body mass index (kg/m2) 26.3 (4.9) Education (yr) 10.1 (3.6) Low leisure time physical activity (%) 34.6 Tea drinker (%) 22.5 Current smoker (%) 27.3 Alcohol drinker (%) 39.4  P trend Age (yr) <:0.001 Body mass index (kg/m2) <:0.001 Education (yr) <:0.001 Low leisure time physical activity (%) <:0.001 Tea drinker (%) <:0.001 Current smoker (%) <:0.001 Alcohol drinker (%) <:0.001  **Location**  North Karelia and Kuopio, and the Turku-Loimaa region in southwestern Finland  **Recruitment strategy** Not reported  **Length of follow-up**  mean follow-up of 12.9 years  **Response rate and loss to follow-up** participation rate varied by year from 74% to 88%  **Eligible population** 25 to 74 years  **Excluded populations** 91 subjects due to prevalent PD at the baseline and 464 subjects due to incomplete data. Exclusion of subjects with stroke or those who used neuroleptic drugs | | | | **Relevant exposures** physical activity, smoking habits, and alcohol, coffee and tea consumption, leisure-time physical activity **Time** 1982, 1987, 1992, and 1997 **Measurement of exposure** self-administered questionnaire.   leisure-time physical activity was classified into three categories: low, moderate, or high  participants were classified as never, ex-, and current smokers. Current smokers then categorised according to the amount of cigarettes smoked daily  participants were asked, “How many cups of coffee or tea do you drink per day?” Coffee consumption categorised: none, 1 to 4 cups, and 5 cups per day. Tea consumption was categorised: none, 1 to 2 cups, and 3 cups per day  Alcohol consumption was categorised: none, 1 to 100, >100 g per week. | | **Outcomes** Parkinson’s disease  **outcome measurement** National Social Insurance Institution’s Register on special reimbursement for drug costs  **Time**  1964 through December 31, 2002 | | **Analysis strategy** Cox proportional hazards models  **Confounders** age, body mass index, systolic blood pressure, total cholesterol, education, leisure-time physical activity, smoking, alcohol and tea consumption, and history of diabetes | | **Number** 102 men and 98 women  **Effect estimates** HR (CI 95%) volume of coffee consumption Men 0 1.00  1–4 0.55 (0.26–1.15)  5 0.41 (0.19–0.88)  P-trend 0.063 Women 0 1.00  1–4 0.50 (0.22–1.12)  5 0.39 (0.17–0.89)  P trend 0.073 Men and Women 0 1.00  1-4 0.53 (0.31–0.92)  5 0.40 (0.23–0.71)  P trend 0.005  Volume of tea consumption Men 0 1.00  1–4 1.06 (0.67–1.69)  5 0.55 (0.24–1.25) P-trend 0.31  Women 0 1.00  1–4 0.97 (0.62–1.52)  5 0.21 (0.05–0.90) P trend 0.11  Men and Women 0 1.00  1-4 1.02 (0.74–1.41)  5 0.41 (0.20–0.83)  P trend 0.038  **Significant trends** coffee drinking is associated with a lower risk of PD. More tea drinking is associated with a lower risk of PD  **Reported limitations  Author** self-report for data on coffee drinking. no data on possible changes of coffee drinking during the follow-up. lack of information about other caffeine sources in participants’ diet  **Source of funding** The Finnish Parkinson Foundation and Special Research Funds of the Social Welfare and Health Board, City of Oulu | |
| **Authors:** Hu G, Tuomilehto J, Silventoinen K, Barengo NC, Peltonen M, et al.  **Year:** 2005  **Citation:** Int J Obes. 2005;29:894–902  **Country of study:** Finland  **Aim of study:** Examine the association of physical activity and body mass index, and their combined effect, with the risk of total, cardiovascular disease and cancer mortality  **Study design:** Prospective follow-up study  **Quality score: (++, + or -):**  **External validity score: (++, + or -):** | | | | | | | | | | | | | |
| **Number of people**  52 058  **Demographics** Not reported | | **Number of people** 47212 **Characteristics**  Physical activity (men)  Low  Number of participants 2132  Age (y) 45.6  BMI (kg/m2) 26.9  Total cholesterol (mmol/l) 6.3  Education level (y) 9.5  Current smoking (%) 52.3  BMI (%) <18.5 0.9  18.5–24.9 35.5  25–29.9 40.6  >30.0 23.0   Moderate  Number of participants 9659  Age (y) 44.3  BMI (kg/m2) 26.3  Total cholesterol (mmol/l) 6.2  Education level (y) 9.5  Current smoking (%) 43.4  BMI (%) <18.5 0.3  18.5–24.9 39.2  25–29.9 45.9  >30.0 14.6   High  Number of participants 10 737  Age (y) 41.3  BMI (kg/m2) 25.9  Total cholesterol (mmol/l) 6.2  Education level (y) 9.2  Current smoking (%) 41.2  BMI (%) <18.5 0.2  18.5–24.9 42.7  25–29.9 46.1  >30.0 11.0    Physical activity (women) Low  Number of participants 3613  Age (y) 45.7  BMI (kg/m2) 27.1  Total cholesterol (mmol/l) 6.2  Education level (y) 9.3  Current smoking (%) 21.5  BMI (%) <18.5 2.0  18.5–24.9 39.1 25–29.9 31.8  >30.0 27.1   Moderate  Number of participants 11 782  Age (y) 44.2  BMI (kg/m2) 25.9  Total cholesterol (mmol/l) 6.1  Education level (y) 9.5  Current smoking (%) 17.9  BMI (%) <18.5 1.2  18.5–24.9 47.4  25–29.9 33.7  >30.0 17.7   High  Number of participants 9289 Age (y) 42.4 BMI (kg/m2) 25.3 Total cholesterol (mmol/l) 6.0 Education level (y) 10.0 Current smoking (%) 16.1 BMI (%) <18.5 1.1 18.5–24.9 52.2 25–29.9 33.5 >30.0 13.2  **Location**  Kuopio and North Karelia provinces, Turku-Loimaa, Oulu  **Recruitment strategy** Random selection  **Length of follow-up**  mean follow-up of 17.7 y  **Response rate and loss to follow-up** participation rate varied by year from 74 to 88%  **Eligible population** Born between 1913 and 1947  **Excluded populations** Subjects previously diagnosed with coronary heart disease (n=1252), stroke (n=423), heart failure (n=1732) and cancer (n=138) at baseline, and subjects with incomplete data on any required factors (n=1301) | | | | **Relevant exposures** Physical activity and smoking **Time** 1972, 1977, 1982, 1987, 1992 and 1997 **Measurement of exposure** self-administered questionnaire. Questions on physical activity included both occupational and leisure time physical activity  occupational physical activity three categories: ‘light’; ‘moderate’; and ‘active’  participants were classified as never, ex-, and current smokers. Current smokers then categorised according to the amount of cigarettes smoked daily | | **Outcomes** cardiovascular disease and cancer mortality  **outcome measurement** Computerized register linkage  Mortality data were obtained from Statistics Finland  **Time**  until the end of 2001 | | **Analysis strategy** Cox proportional hazards model  **Confounders** age, study year, education, smoking, systolic blood pressure, total cholesterol, diabetes and physical activity or BMI | | **Number** 7394 deaths  **Effect estimates** Hazard ratios for total, cardiovascular and cancer mortality according to different levels of physical activity among Finnish men and women Men Total mortality Low 1.00 Moderate 0.74 (0.68–0.81) High 0.63 (0.58–0.70) P-value for trend <0.001 Cardiovascular mortality Low 1.00 Moderate 0.82 (0.72–0.93) High 0.71 (0.62–0.82) P-value for trend <0.001 Cancer mortality Low 1.00 Moderate 0.83 (0.69–1.00) High 0.79 (0.65–0.96) P-value for trend 0.05  Women Total mortality Low 1.00 Moderate 0.64 (0.58–0.70) High 0.58 (0.52–0.64) P-value for trend <0.001 Cardiovascular mortality Low 1.00 Moderate 0.62 (0.54–0.71) High 0.55 (0.47–0.65) P-value for trend <0.001 Cancer mortality Low 1.00 Moderate 0.85 (0.71–1.01) High 0.73 (0.60–0.88) P-value for trend 0.005  Hazard ratios for total mortality according to different levels of BMI and smoking status among Finnish men and women Never smoker BMI <18.5 1.39 (0.34–5.62) 18.5–24.9 1.00 25–29.9 1.03 (0.88–1.20) >30.0 1.78 (1.47–2.15) P-value for trend <0.001  Ex-smoker BMI <18.5 1.87 (0.59–5.92) 18.5–24.9 1.00 25–29.9 1.03 (0.88–1.21) >30.0 1.48 (1.23–1.78) P-value for trend <0.001  Current smoker BMI <18.5 3.17 (1.99–5.06) 18.5–24.9 1.00 25–29.9 0.98 (0.90–1.06) >30.0 1.14 (1.01–1.28) P-value for trend <0.001  **Significant trends** Total mortality was increased both among lean and obese subjects. Statistically significant increased total mortality was observed among the obese men in all smoking categories. Regular physical activity and normal weight are both important indicators for a decreased risk of mortality from all causes, CVD and cancer  **Reported limitations  Author** self-report of physical activity. Changes to physical activity behaviours among the cohort members over time causes misclassification. standardised alcohol drinking data was not available across the survey.   **Source of funding** Finnish Academy (grant numbers 46 558, 53 585, 204 274 and 205 657). | |
| **Authors:** Hu G, Tuomilehto J, Silventoinen K, Barengo N, Jousilahti P  **Year:** 2004  **Citation:** Eur Heart J. 2004;25:2212–2219  **Country of study:** Finland  **Aim of study:** Assess joint associations of physical activity and different indicators of obesity with the risk of cardiovascular disease  **Study design:** Prospective follow-up study  **Quality score: (++, + or -):**  **External validity score: (++, + or -):** | | | | | | | | | | | | | |
| **Number of people**  20,547  **Demographics** Not reported | | **Number of people** 18,892 **Characteristics**  Baseline characteristics according to physical activity levels among the Finnish population by sex Physical activity (men)  Low  Age (year) 47.9  Body mass index 27.3  Total cholesterol (mmol/l) 5.8  Education (year) 11.3  Current smoking (%) 44.6  Obesity (%) 26.7   Moderate  Age (year) 48.0  Body mass index 26.6  Total cholesterol (mmol/l) 5.7  Education (year) 11.3  Current smoking (%) 33.5  Obesity (%) 17.9   High  Age (year) 42.9  Body mass index 26.6  Total cholesterol (mmol/l) 5.7  Education (year) 10.3  Current smoking (%) 34.3  Obesity (%) 14.1   p-Value  Age (year) <0.001  Body mass index <0.001  Total cholesterol (mmol/l) 0.002  Education (year) <0.001  Current smoking (%) <0.001  Obesity (%) <0.001   Physical activity (women)  Low  Age (year) 46.7  Body mass index 27.0  Total cholesterol (mmol/l) 5.6  Education (year) 11.3  Current smoking (%) 25.4  Obesity (%) 28.5   Moderate  Age (year) 46.4  Body mass index 25.9  Total cholesterol (mmol/l) 5.6  Education (year) 11.3  Current smoking (%) 20.3  Obesity (%) 18.8   High  Age (year) 43.3  Body mass index 25.7  Total cholesterol (mmol/l) 5.6  Education (year) 11.2  Current smoking (%) 19.6  Obesity (%) 13.8   p-Value Age (year) <0.001 Body mass index <0.001 Total cholesterol (mmol/l) 0.4 Education (year) 0.04 Current smoking (%) <0.001 Obesity (%) <0.001  **Location** Kuopio and North Karelia provinces, Turku-Loimaa, Oul**u  Recruitment strategy** random sample, stratified by area, gender and 10-year age group **Length of follow-up** median follow-up time was 9.8 years **Response rate and loss to follow-up** participation rate varied by year from 74 to 88% **Eligible population** Born between 1913 and 1947s **Excluded populations** previously diagnosed with coronary heart disease (CHD) (n = 672), stroke (n = 390) and heart failure (n = 408), incomplete data on any required factors (n=185) | | | | **Relevant exposures** Physical activity and smoking **Time** 1972, 1977, 1982, 1987, 1992 and 1997 **Measurment of exposure** self-administered questionnaire. Questions on physical activity included both occupational and leisure time physical activity  occupational physical activity three categories: ‘light’; ‘moderate’; and ‘active’  participants were classified as never, ex-, and current smokers. Current smokers then categorised according to the amount of cigarettes smoked daily | | **Outcomes** cardiovascular disease and cancer mortality  **outcome measurement** Finnish hospital discharge register for non-fatal outcomes (hospitalised myocardial infarction and stroke) and the mortality register by the Statistics Finland for fatal outcomes (cardiovascular death)  **Time**  until the end of 2001 | | **Analysis strategy** Cox proportional hazards model  **Confounders** age, study year, education, smoking, systolic blood pressure, total and HDL cholesterol, diabetes at baseline, and body mass index | | **Number** 818  **Effect estimates** Hazard ratios for risk of cardiovascular disease according to different levels of physical activity by sex Men Physical activity Low 1.00 Moderate 0.72 (0.57–0.91) High 0.68 (0.52–0.88) P trend 0.007  Women Physical activity Low 1.00 Moderate 0.73 (0.55–0.97) High 0.64 (0.45–0.89) P trend 0.02  **Significant trends** Both regular physical activity and normal weight can reduce the risk of CVD  **Reported limitations  Author** self-report of physical activity. Residual confounding. Data on several risk factors, such as triglycerides and apolipoprotein B, are not available.   **Source of funding** Finnish Academy (Grants 46558, 53585, 204274, and 205657). | |
| **Authors:** Humphries SE, Talmud PJ, Hawe E, Bolla M, Day I, et al.  **Year:** 2001  **Citation:** Lancet. 2001;358:115–119  **Country of study:** UK  **Aim of study:** Investigated whether the effect of smoking on coronary heart disease risk is affected by APOE genotype  **Study design:**  **Quality score: (++, + or -):**  **External validity score: (++, + or -):** | | | | | | | | | | | | | |
| **Number of people**  3984  **Demographics** Not reported | | **Number of people** 3052 **Characteristics**  No CHD event (n=2122)  Age (years) 55·7 (3·2)  Body-mass index (kg/m2) 26·2 (3·4) Current smokers (number [%]) 573 (27%)  CHD event (n=136) Age (years) 56·34 (3·4) Body-mass index (kg/m2) 27·0 (3·4) Current smokers (number [%]) 52 (38%)  **Location**  UK  **Recruitment strategy** Not reported. Pts registered with nine general medical practices  **Length of follow-up**  11 years  **Response rate and loss to follow-up** RR (77%)  **Eligible population** middle-aged men  **Excluded populations** Women | | | | **Relevant exposures** smoking **Time** Not reported **Measurement of exposure** smoking habit divided into groups: smokers (any man who had smoked at least one cigarette daily on average for a year or more), ex-smokers (those who had smoked less than one cigarette a day), and never-smokers | | **Outcomes** Coronary heart disease  **outcome measurement** ECG  **Time**  Not reported | | **Analysis strategy** Cox’s proportional hazards model  **Confounders** practice and conventional risk factors by including age, BMI, cholesterol, triglyceride, fibrinogen, and systolic blood pressure | | **Number** 96 men had an acute myocardial infarction, 26 needed coronary artery surgery, and 14 had silent myocardial infarctions  **Effect estimates** Adjusted hazard ratio (95% CI) Never-smokers 1.00 Ex-smokers E3/E3 1.49 (0.93–2.37) E2+ 0.47 (0.11–1•94) E4+ 0.74 (0.35–1.55) Smokers  E3/E3 1.47 (0.87–2.51) E2+ 0.85 (0.30–2.43) E4+ 2.79 (1.59–4.91)  **Significant trends** Smoking increases the risk of coronary heart disease in men of all genotypes but particularly in men carrying the E4 allele  **Reported limitations  Reviewer** CIs for the risk estimates are large  **Source of funding** British Medical Research Council, the US National Institute of Health (NHLBI 33014), and DuPont Pharma, Wilmington, USA. SEH, PJT, MB, and DMW are supported by the British Heart Foundation, and INMD is a Lister Institute fellow | |
| **Authors:** Inoue M, Hanaoka T, Sasazuki S, Sobue T, Tsugane S, et al.  **Year:** 2004  **Citation:** Prev Med. 2004;38:516-522  **Country of study:** Japan  **Aim of study:** Obtain a relevant epidemiological index of the impact of tobacco smoking on the subsequent risk of cancer in Japan  **Study design:** Population-based prospective study  **Quality score: (++, + or -):**  **External validity score: (++, + or -):** | | | | | | | | | | | | | |
| **Number of people**  116,896  **Demographics** Not reported | | **Number of people** 92,792 subjects (44,521 men and 48,271 women) **Characteristics**  Men Number of subjects 44,521 Proportion (%) Age (years) ± SD 52.9 ± 7.9 Alcohol drinking status (%)  none 23.1  occasional 9.4 <150 g/week 22.2 150–299 g/week 19.9 300–449 g/week 13.2 z450 g/week 12.2 Body mass index (%)  <18.9 4.1 19.0 – 20.9 14.5 21.0 – 22.9 25.7 23.0 – 24.9 27.9 25.0 – 26.9 16.6 27.0 – 29.9 9.0 >30.0 2.2 Green vegetable intake (%)  less 75.8 everyday 24.2 Women Number of subjects 48,271 Proportion (%) Age (years) ±F SD 53.3 ± 8.0 Alcohol drinking status (%)  none 79.4  monthly 9.8 <100 g/week 7.3 >100 g/week 3.5 <18.9 5.3 Body mass index (%) 19.0 – 20.9 15.2 21.0 – 22.9 26.0 23.0 – 24.9 24.6 25.0 – 26.9 15.5 27.0 – 29.9 10.1 >30.0 3.3 Green vegetable intake (%) Less 68.7 Everyday 31.3  **Location**  Ninohe City and Karumai Town in the Ninohe PHC area of Iwate Prefecture, Yokote City and Omonogawa town in the Yokote PHC area of Akita, 8 districts of Minami-Saku County in the Saku PHC area of Nagano, Gushikawa City and Onna Village in the Ishikawa PHC area of Okinawa  **Recruitment strategy** self-administered questionnaire was distributed to all registered noninstitutional residents in 1990  **Length of follow-up**  10yr, up to 31st December, 2001  **Response rate and loss to follow-up** response rate of 82% to the baseline questionnaire. proportion of losses to follow-up (0.05%)  **Eligible population** General population  **Excluded populations**  Two metropolitan areas. 210 subjects were found to be ineligible for the study and excluded because of non-Japanese nationality (n = 51), late reports of out-migration before the start of the follow-up (n = 156) and age ineligibility due to wrong birth date (n = 3). | | | | **Relevant exposures** Alcohol **Time** 2001 **Measurement of exposure** Alcohol consumption was represented in the questionnaire by the frequency of consumption during the past month and categorized into 6 classes: 1 day/month, 1 to 3 days/month, 1 to 2 days/week, 3 to 4 days/week, 5 to 6 days/week, and every day | | **Outcomes** Cancer  **outcome measurement** Occurrence of cancer was identified by active patients’ notification from local major hospitals in the study area and data linkage with population-based cancer registries with permission. Death certificate information was used as a supplementary information source  **Time**  Not reported | | **Analysis strategy** Cox proportional hazards model  **Confounders** age at baseline, study area, weekly ethanol intake, body mass index and green vegetable intake | | **Number** 4,922 newly diagnosed cancer cases (2,969 men and 1,953 women) and 2,132 cases of cancer deaths (1,411 men and 721 women)  **Effect estimates** Hazard ratios of cancer incidence and death according to smoking status in men Total cancer incidence (n = 2,969)  Number of cases Hazard ratio 95% CI  Smoking status Never 488 1.00 (reference)  Former 777 1.37 (1.22 – 1.54)  Current 1704 1.64 (1.48 – 1.82)  Daily cigarette consumption <19 483 1.48 (1.29 – 1.68) 20 – 29 796 1.71 (1.52 – 1.93) >30 425 1.72 (1.51 – 1.98) trend P < 0.05  Pack-years  <19 190 1.26 (1.06 – 1.49) 20 – 29 307 1.54 (1.33 – 1.79) 30 – 39 474 1.76 (1.54 – 2.08) >40 732 1.76 (1.56 – 1.98) trend P < 0.001  Age started smoking >25 283 1.50 (1.28 – 1.74)  20 – 24 1001 1.62 (1.45 – 1.82)  <19 420 1.81 (1.58 – 2.08)  trend P < 0.05   Total cancer death (n = 1,411) Number of cases Hazard ratio 95% CI  Smoking status Never 223 1.00 (reference) Former 351 1.35 (1.13 – 1.53) Current 837 1.78 (1.53 – 2.09) Daily cigarette consumption <19 244 1.64 (1.35 – 1.98) 20 – 29 391 1.86 (1.56 – 2.21) >30 202 1.84 (1.51 – 2.25) trend n.s  Pack-years  <19 96 1.49 (1.16 – 1.91) 20 – 29 153 1.75 (1.41 – 2.17) 30 – 39 220 1.86 (1.53 – 2.26) >40 367 1.86 (1.56 – 2.22) trend n.s  Age started smoking >25 142 1.65 (1.32 – 2.06) 20 – 24 473 1.71 (1.45 – 2.03) <19 222 2.11 (1.73 – 2.57) trend P < 0.05   Hazard ratios and of cancer incidence and death according to smoking status in women Total cancer incidence (n = 1,953)  Number of cases Hazard ratio 95% CI  Smoking status  Never 1779 1.00 (reference)  Former 37 1.47 (1.05 – 2.05)  Current 137 1.46 (1.21 – 1.75)  Daily cigarette consumption <19 90 1.45 (1.16 – 1.81)  20 – 29 32 1.42 (0.99 – 2.03)  >30 15 1.63 (0.98 – 2.72)  trend n.s.  Pack-years  <19 80 1.34 (1.06 – 1.69)  20 – 29 30 1.78 (1.20 – 2.63)  30 – 39 10 1.32 (0.71 – 2.47)  >40 17 1.83 (1.13 – 2.96)  Trend n.s.  Age started smoking >25 92 1.39 (1.12 – 1.73)  20 – 24 40 1.73 (1.24 – 2.41)  <19 5 1.10 (0.45 – 2.66)  Trend n.s.   Total cancer death (n = 721) Number of cases Hazard ratio 95% CI  Smoking status  Never 656 1.00 (reference) Former 10 1.03 (0.53 – 1.99) Current 55 1.58 (1.18 – 2.12) Daily cigarette consumption <19 32 1.36 (0.93 – 2.00) 20 – 29 16 1.99 (1.20 – 3.31) >30 7 1.96 (0.93 – 4.15) Trend n.s.  Pack-years  <19 23 1.08 (0.69 – 1.67) 20 – 29 20 3.37 (2.09 – 5.44) 30 – 39 7 2.18 (1.03 – 4.62) >40 5 1.26 (0.52 – 3.06) Trend n.s.  Age started smoking >25 35 1.41 (0.99 – 2.00) 20 – 24 18 2.22 (1.34 – 3.70) <19 2 1.36 (0.34 – 5.51) Trend n.s.   **Significant trends** From the baseline questionnaire, 52.2% of men were current smokers and they presented a significantly increased HR of subsequent cancer occurrence compared with never-smokers [HR 1.64, 95% confidence interval (95% CI) 1.48–1.82]. Only 5.6% of women were current smokers and their HR also represented a significant increase (HR 1.46, 95% CI 1.21–1.75).  **Reported limitations  Author** could not fully evaluate the effect of passive smoking due to the lack of detailed information. The proportion of female current smokers was 12–20% in two metropolitan areas. This is very high compared with the 4–10% proportion included in the analysis  **Source of funding** Grant-in-Aid for Cancer Research and for the Second Term Comprehensive 10-year Strategy for Cancer Control from the Ministry of Health, Labor and Welfare, Japan. | |
| **Authors:** Iso H, Baba S, Mannami T, Sasaki S, Okada K, et al.  **Year:** 2004  **Citation:** Stroke. 2004;35:1124-1129  **Country of study:** Japan  **Aim of study:** Examine impact of light-to-moderate alcohol consumption on risk of stroke  **Study design:** Prospective Study  **Quality score: (++, + or -):**  **External validity score: (++, + or -):** | | | | | | | | | | | | | |
| **Number of people**  27 063 men and 27 435 women  **Demographics** Not reported | | **Number of people** 19 544 **Characteristics**  Nondrinker N at risk 4063 Age, year 50.0 Smoking history, %  Never 30 Past 22 Current 48 Mean body mass index, kg/m2 23.5 History of hypertension, % 11 History of diabetes, % 6 College or higher education, % 13 Sport at leisure time 2:1 d/wk, % 15 Diet, n of frequencies  Fruit, d/wk 3.2 Green vegetables, d/wk 3.5 Yellow vegetables, d/wk 2.8 Other vegetables, d/wk 3.7 Fresh fish, d/wk 2.5 Dried fish, d/wk 1.7  Occasional Drinker N at risk 2133 Age, year 48.9 Smoking history, %  Never 33 Past 20 Current 46 Mean body mass index, kg/m2 24.0 History of hypertension, % 11 History of diabetes, % 5 College or higher education, % 17 Sport at leisure time 2:1 d/wk, % 19 Diet, n of frequencies  Fruit, d/wk 3.1 Green vegetables, d/wk 3.6 Yellow vegetables, d/wk 2.8 Other vegetables, d/wk 3.5 Fresh fish, d/wk 2.4 Dried fish, d/wk 1.4  **Location**  Ninohe City and Karumai Town in the Ninohe PHC area of Iwate Prefecture, Yokote City and Omonogawa town in the Yokote PHC area of Akita, 8 districts of Minami-Saku County in the Saku PHC area of Nagano, Gushikawa City and Onna Village in the Ishikawa PHC area of Okinawa  **Recruitment strategy** self-administered questionnaire was distributed to all registered noninstitutional residents in 1990  **Length of follow-up**  10-year follow-up questionnaire (88% followed-up)  **Response rate and loss to follow-up** 20 665 men (76%) initially. 11.0 years of follow-up from 1990 to the end of 2001. 10-year follow-up questionnaire (88% followed-up)  **Eligible population** Men aged 40-59  **Excluded populations**  data for women are not presented because of the small number of moderate-to-heavy drinkers in this group. Excluded men who reported stroke, myocardial infarction, angina pectoris, or cancer at baseline | | | | **Relevant exposures** Alcohol consumption, diet **Time** 1990 **Measurement of exposure** Alcohol consumption was represented in the questionnaire by the frequency of consumption during the past month and categorized into 6 classes: 1 day/month, 1 to 3 days/month, 1 to 2 days/week, 3 to 4 days/week, 5 to 6 days/week, and every day  frequency of weekly intake of 27 food items was reported under 4 categories: rarely, 1 to 2 days/week, 3 to 4 days/week, and almost every day | | **Outcomes** stroke  **outcome measurement** registered a total of 25 hospitals facilitated by computer tomographic scan and/or magnetic resonance images in the 4 PHC areas. Medical records were reviewed by registered hospital workers or PHC physicians, blinded to the lifestyle data. systematic search for death certificates was also undertaken.  **Time**  Stroke events were registered if they occurred after the date of return of the baseline questionnaire and before January 1, 2002. | | **Analysis strategy** Cox proportional hazards models  C**onfounders** age, smoking status, body mass index, history of diabetes, education level, sports at leisure time, and dietary intake categories of fruits, total vegetables, and fish. History of hypertension as well as the confounding variables to examine the residual or independent effect of alcohol consumption on risk of stroke | | **Number** 694  **Effect estimates** Nondrinker Person-years 44 379  Total stroke N of cases 133  RR (95% CI) Further adjusted for hypertension 1.09 (0.80–1.48)  Definite total stroke N of cases 106  Further adjusted for hypertension 0.95 (0.68–1.33)  Hemorrhagic stroke N of cases 48  Further adjusted for hypertension 1.48 (0.84–2.62)  Intraparenchymal hemorrhage N of cases 38  Further adjusted for hypertension 1.35 (0.73–2.51)  Subarachnoid hemorrhage N of cases 10  Further adjusted for hypertension 2.40 (0.52–11.0)  Ischemic stroke  N of cases 58  Further adjusted for hypertension 0.72 (0.47–1.08)  Lacunar infarction  N of cases 27  Further adjusted for hypertension 0.64 (0.36–1.16)  Large-artery occlusive infarction  N of cases 11  Further adjusted for hypertension 0.73 (0.28–1.89)  Embolic infarction  N of cases 10  Further adjusted for hypertension 0.58 (0.23–1.47)   Occasional drinker Person-years 23,532 Total stroke N of cases 59 Further adjusted for hypertension 1.0 Definite total stroke N of cases 53 Further adjusted for hypertension 1.0 Hemorrhagic stroke N of cases 16 Further adjusted for hypertension 1.0 Intraparenchymal hemorrhage N of cases 14 Further adjusted for hypertension 1.0 Subarachnoid hemorrhage N of cases 2 Further adjusted for hypertension 1.0 Ischemic stroke  N of cases 37 Further adjusted for hypertension 1.0 Lacunar infarction  N of cases 19 Further adjusted for hypertension 1.0 Large-artery occlusive infarction  N of cases 7 Further adjusted for hypertension 1.0 Embolic infarction  N of cases 8 Further adjusted for hypertension 1.0  **Significant trends** There was a lower risk of ischemic stroke, more specifically lacunar infarction, a higher risk of hemorrhagic stroke, and no excess risk of total stroke among drinkers of 1 to 149 g ethanol per week compared with occasional drinkers; the respective multivariate RR (95% CI) was 0.59 (0.37 to 0.93), 0.43 (0.22 to 0.87), 1.73 (0.98 to 3.07), and 0.98 (0.71 to 1.36).  **Reported limitations  Author** residual uncontrolled confounding of the association between alcohol consumption and risk of stroke. data on hypertension and diabetes were self-reported. generalizability of findings to women  **Source of funding** Cancer Research and for the Second Term Comprehensive Ten-Year Strategy for Cancer Control from the Ministry of Health, Labor, and Welfare of Japan | |
| **Authors:** Janzon E, Hedblad B, Berglund G, Engström, G  **Year:** 2004  **Citation:** J Intern Med. 2004;256:111–118  **Country of study:** Sweden  **Aim of study:** Explored how the risk of myocardial infarction in current and former smokers is modified by other cardiovascular risk factors  **Study design:** Cohort study  **Quality score: (++, + or -):**  **External validity score: (++, + or -):** | | | | | | | | | | | | | |
| **Number of people**  10 902  **Demographics** Not reported | | **Number of people** 10619 **Characteristics**  Never-smokers (n = 4848) Age (year) 50.5 ± 6.6 (28.3–57.6) BMI (kg m)2) 25 ± 4  Occupation, low (%) 77.5 Civil status, married (%) 77.4  Ex-smokers (n = 2035) Age (year) 49.8 ± 7.3 (28.2–57.2) BMI (kg m)2) 24 ± 4 Occupation, low (%) 72.1 Civil status, married (%) 73.2  Current smokers (n = 3738) Age (year) 48.3 ± 8.2 (28.2–56.9) BMI (kg m)2) 23 ± 4  Occupation, low (%) 81.4 Civil status, married (%) 64.6  **Location**  Malmo, Sweden  **Recruitment strategy** Not reported  **Length of follow-up**  Mean follow-up was 14.0 ± 4.5 years (range 0.5–21.9 years)  **Response rate and loss to follow-up** overall attendance rate was 71  **Eligible population** women 28–58 years old  **Excluded populations** exclude women with a history of MI or stroke (n=176) | | | | **Relevant exposures** Smoking **Time** Not reported **Measurement of exposure** self-administered questionnaire. Women asked ‘Are you a smoker?’ or ‘Are you a daily smoker?’ Women who reported that they had stopped smoking were considered to be ex-smokers. Women who did not report any history of smoking were never-smokers  Tobacco consumption amongst daily smokers was classified as low consumption (<10 cigarettes day), medium consumption (>10, <20 cigarettes day) and high consumption (>20 cigarettes day). | | **Outcomes** myocardial infarction  **outcome measurement** cases of nonfatal MI were retrieved from the Malmo Myocardial Infarction register and from the Swedish Myocardial Infarction register  **Time**  All subjects were followed from the baseline examination until death, cardiac event, emigration out of Sweden, or to 31 December 1998 | | **Analysis strategy** Cox proportional hazards model  C**onfounders** systolic blood pressure, age, BMI, cholesterol, diabetes, occupation, marital status and HRT | | **Number** 228  **Effect estimates** RR (CI 95%) Never-smoker Normotension 1.0  Hypertension 2.4 (1.4–4.3) Normal cholesterol 1.0 High cholesterol 1.8(1.02–3.2) No diabetes 1.0 Diabetes 8.8 (4.4–17.4)  Ex-smoker Normotension 1.8 (0.99–3.2) Hypertension 2.7 (1.1–6.0) Normal cholesterol 1.6 (0.9–2.9) High cholesterol 2.4 (1.2–5.0) No diabetes 1.7 (1.01–2.8) Diabetes 7.8 (2.4–25.6)  Current smoker Normotension 5.3 (3.3–8.1) Hypertension 12.2 (7.5–19.8) Normal cholesterol 5.6 (3.6–8.6) High cholesterol 8.2 (5.2–12.9) No diabetes 6.0 (4.1–8.6) Diabetes 19.0 (10.2–35.4)  **Significant trends** smoking is a major risk factor for MI, however the risk varies widely between women with similar tobacco consumption. There was a statistically significant interaction between smoking and hypertension for the risk of cardiac events  **Reported limitations  Author** crude categorisation of occupation. No question about occasional smoking. misclassification of occasional smokers as never-smokers. No information on age of initiation of smoking. No information on smoking cessation during follow-up  **Reviewer** Self-reported exposure  **Source of funding** Swedish Council for Work Life and Social Research | |
| **Authors:** Johnsen NF, Christensen J, Thomsen BL, Olsen A, Loft S, et al.  **Year:** 2006  **Citation:** Eur J Epidemiol. 2006;21:877–884  **Country of study:** Denmark  **Aim of study:** Investigate the effects of occupational activity and leisure time activity on incident colon cancer risk  **Study design:** Prospective cohort  **Quality score: (++, + or -):**  **External validity score: (++, + or -):** | | | | | | | | | | | | | |
| **Number of people**  160,725  **Demographics** Not reported | | **Number of people** 57,053 **Characteristics**  Women Age (years) 56 (50–64)  BMI 25 (20–34) Education (years) < 7 8751 (31)  8–10 14,296 (50)  >10 5309  Smoking Never 12,460 (44)  Former 6716 (24)  Present 9180 (32)  Alcohol (g/day) 27,764 (97)  Total number of Leisure time physical activity 28,356 (100)  Occupational physical activity Sitting 9248 (33)  Standing 4964 (18)  Manual 6219 (22)  Not working 7925 (28)   Men Age (years) 56 (50–64)  BMI 26 (21–33) < 7 9000 (34) 8–10 10,894 (42)  >10 6228 (24)  Smoking Never 6764 (26)  Former 9104 (35)  Present 10,254 (39)  Alcohol (g/day) 25,787 (98)  Total number of Leisure time physical activity 26,064 (100) Occupational physical activity Sitting 10,369 (40)  Standing 4417 (17)  Manual) 7282 (28)  Not working 4054 (16)   **Location**  Copenhagen and Aarhus, Denmark  **Recruitment strategy** Not reported  **Length of follow-up**  mean follow-up of 7.6 years  **Response rate and loss to follow-up** Less than 0.8% of the participants were completely lost to follow-up  **Eligible population** Danish middle-aged population  **Excluded populations** | | | | **Relevant exposures** smoking, daily intake of dietary fiber, red meat, alcohol and dietary fat **Time** 1993-1997 **Measurement of exposure** self-report questionnaire | | **Outcomes** colon cancer  **outcome measurement** Danish Cancer Registry  **Time**  date of diagnosis of any cancer (except for non-melanoma skin cancer), date of death or emigration, or December 31, 2003, whichever came first | | **Analysis strategy** Cox proportional hazards model  **Confounders** Model 1) participation in each of the six activities, occupational physical activity (four categories), BMI, education, NSAID, present use of HRT, smoking and intake of total energy, fat, dietary fibre, red meat and alcohol.  Model 2) occupational activity, BMI, education, NSAID, present use of HRT, smoking and intake of total energy, fat, dietary fibre, red meat and alcohol | | **Number** 140 women and 157 men were diagnosed with colon cancer  **Effect estimates** 1) Incidence rate ratios for colon cancer per additional hour/week of six types of leisure time physical activity IRR (95% CI)  Women  MET-score (per 10 units) 1.00 (0.96–1.04 Sports (per one hour) 1.03 (0.93–1.14)  Cycling (per one hour) 1.00 (0.94–1.06)  Walking (per one hour) 0.99 (0.95–1.03)  Gardening (per one hour) 0.95 (0.87–1.03)  Housework (per one hour) 1.01 (0.98–1.03)  Do-it-yourself (per one hour) 1.00 (0.91–1.11)   Men (cases = 157) MET-score (per 10 units) 0.97 (0.93–1.01) Sports (per one hour) 1.00 (0.91–1.10)  Cycling (per one hour) 1.00 (0.94–1.07)  Walking (per one hour) 1.01 (0.98–1.04)  Gardening (per one hour) 0.98 (0.92–1.04)  Housework (per one hour) 0.95 (0.89–1.02)  Do-it-yourself (per one hour) 0.97 (0.92–1.02)  2) Incidence rate ratios for colon cancer for active compared to non-active for six types of leisure time physical activity IRR (95% CI) Women  Total number of activities (per extra activity) 0.89 (0.77–1.03)  Sports 0.85 (0.60–1.20)  Cycling 0.89 (0.62–1.28)  Walking 0.94 (0.48–1.86 Gardening 0.96 (0.67–1.39)  Housework *d*  Do-it-yourself 0.86 (0.60–1.23)   Men Total number of activities (per extra activity) 0.90 (0.79–1.03) Sports 0.90 (0.64–1.25)  Cycling 0.92 (0.66–1.28)  Walking 1.19 (0.65–2.16)  Gardening 1.12 (0.74–1.69)  Housework 0.78 (0.51–1.19)  Do-it-yourself 0.69 (0.45–1.06)  **Significant trends** No associations were found between risk of colon cancer and occupational activity, MET-hours per week of total leisure time activity, residuals from a regression of each activity on the total MET-hours or the time spent on any of the six types of leisure time activities  **Reported limitations  Author** Not all the aspects of activity (type, frequency, duration and intensity) were measured. Self-administered questionnaires. Study is based on persons of a relatively high age. Adding random variation by applying an assumed intensity to the included activities  **Source of funding** Danish Medical Research Council and the Danish Cancer Society. | |
| **Authors:** Kareholt I, Lennartsson C, Gatz M, Parker MG  **Year:** 2011  **Citation:** Int J Geriatr Psychiatry 2011; 26: 65–74.  **Country of study:** Sweden  **Aim of study:** Association between different types of leisure time activity at baseline and cognition at follow-up  **Study design:**  **Quality score: (++, + or -):**  **External validity score: (++, + or -):** | | | | | | | | | | | | | |
| **Number of people**  7483  **Demographics** Not reported | | **Number of people** 1643 **Characteristics**  Age Range 46–75 years  mean 57.4  Age at FU Range 69–98 Mean 80.2  Sex % Women 58.7  Men 41.3 Mobility problems % 0 70.8 1 13.8 2 8.8  3 6.6  Employment status % Employed 70.1 Unemployed 29.9 Years of education Range 0–29 Mean 8.4  Adult socioeconomic status  % Blue-collar (unskilled) 33.4  Blue-collar (skilled) 21.1 Lower white-collar 19.5 Intermediate and upper white-collar 26.0  Childhood socioeconomic status  % Blue-collar (unskilled) 24.7  Blue-collar (skilled) 35.1 Lower white-collar 28.4 Intermediate and upper white-collar 11.0 Missing and unclassifiable 0.9  Smoking % No 74.6 Yes, 1–10 cigarettes 12.8 Yes >10 cigarettes 12.5 Alcohol drinking % No 24.4 Moderate 44.9 >Moderate 30.7 Total 1643  **Location**  Sweden  **Recruitment strategy** Randomised recruitment  **Length of follow-up**  from 1968 to 1992 was 24 years, from 1981 to 2002 was 21 years, and from 1981 to 2004 was 23 years (average 22.8 years)  **Response rate and loss to follow-up** 'has a low nonresponse rate'. Number not provided  **Eligible population** Not reported  **Excluded populations** 1871 persons aged 56–75 were interviewed in 1968. Of these, 534 were still alive in 1992 and cognitive data could be collected for 430 individuals. From the 1981 baseline survey, cognitive data could be collected for 416 individuals in 2002 and for 797 individuals in 2004. | | | | **Relevant exposures** smoking and alcohol, political, mental, and socio-cultural activities **Time** Baseline **Measurement of exposure** Smoking: Non-smoker; smoking 10 cigarettes (corresponding to 20 pipes or 5 cigars); >10 cigarettes.  Alcohol drinking: Non drinker; moderate drinker; drinking more than moderately. Moderate drinking is less than two times per month and normally not more than 1–2 glassesPolitical activities: ever having appealed a decision made by a public authority, delivered a speech at a meeting, written an article or a letter to the editor, or participated in a demonstration during the past year  Organizational activities: being an active trade union member (at least attending meetings), member of a political party, sports, temperance, religious, or other organization.  Mental activities: reading books, playing an instrument/ singing, and doing hobby activities.   Socio-cultural activities: going to the movies, the theatre, and attending study circles.   Social activities: four questions concerning visiting and/or being visited by friends and/or relatives.  Physical activities: doing sports, gardening, and dancing | | **Outcomes** Cognition  **Outcome measurement** MMSE  **Time**  1992, 2002, or 2004 | | **Analysis strategy** ordered logistic regressions  **Confounders** age, age-square, sex, follow-up-time, mobility problems, symptoms of mental distress, employment status, education, adult and childhood socioeconomic status, income, smoking, and drinking | | **Number** Not reported  **Effect estimates** β p-Value Political 0.17 0.004 Mental 0.11 0.047 Socio-cultural0.04 0.415 Social 0.01 0.904 Organizational -0.03 0.628 Physical, all 0.21 0.05 0.375 Men -0.06 0.477 Women 0.14 0.055  **Significant trends** significant association between later cognition and earlier political, mental, and socio-cultural activities. Physical activities had a significant association with cognition only among women.  **Reported limitations  Author** low level for moderate drinking. absence of a baseline measure of cognition  **Reviewer** Unclear reporting of study design and process. Do not report how many people developed cognitive issues  **Source of funding** Swedish Research Council (2007-1947) obtained by Marti G. Parker and by the Zenith award from the Alzheimer’s Association (ZEN-02-3895) to Margaret Gatz | |
| **Authors:** Kato M, Noda M, Inoue M, Kadowaki T, Tsugane S  **Year:** 2009  **Citation:** Endocr J. 2009;56(3):459-68  **Country of study:** Japan  **Aim of study:** Assess association between psychological factors and the onset of diabetes  **Study design:** Community-based, prospective cohort study  **Quality score: (++, + or -):**  **External validity score: (++, + or -):** | | | | | | | | | | | | | |
| **Number of people**  95,373  **Demographics** Not reported | | **Number of people** 55,826 **Characteristics**  J and K cells  **Location**  Ninohe, Yokote, Saku, Chubu, and Katsushika. Mito, Nagaoka, Chuohigashi, Kamigoto, Miyako and Suita.  **Recruitment strategy** Not reported  **Length of follow-up**  10 years  **Response rate and loss to follow-up** 75%, 32,369 men and 39,344 women  **Eligible population** middle-aged Japanese adults 40-69 years  **Excluded populations** individuals who had; cardiovascular disease, chronic liver disease, kidney disease or any type of cancer (n=4,515), also excluded any subjects with diabetes at baseline (n=3,092). Individuals who had missing baseline data for any of the exposure parameters (n=9,256). Individuals with a body mass index of less than 14 or more than 40 (n=741 ). | | | | **Relevant exposures** Behaviours, diet **Time** 1990 - 1993 **Measurement of exposure** self-administered questionnaire, food frequency questionnaire  Mental stress was assessed based on three levels of response (low, medium and high) to the question, 'How much stress do you feel in your daily life?'  type A behavioral patterns: competitive drive, speed and impatience, aggressiveness and irritability | | **Outcomes** diagnosed diabetes  **outcome measurement** 94% of the self-report cases of diagnosed diabetes were confirmed by medical records  **Time**  5- or 10-years after wave 1 | | **Analysis strategy** logistic regression  **Confounders** age, body mass index, smoking status, alcohol intake, family history of diabetes, physical activity, history of hypertension, and coffee consumption | | **Number** 1,601 incident cases ( 6.4%) of diabetes among men and 1,093 cases (3.5%) among women  **Effect estimates** Odds ratios for the 1 0-year incidences of diabetes mellitus according to perceived mental stress Men Low Perceived Mental Stress (reference)  Medium Perceived Mental Stress 1.19 (1.01-1.40)  High Perceived Mental Stress 1.36 (1.13-1.63)  p for trend 0.001  Women Low Perceived Mental Stress (reference)  Medium Perceived Mental Stress 1.12 (0.94-1.34)  High Perceived Mental Stress 1.22 (0.98-1.51)  p for trend 0.080  Odds ratios for the 1 0-year incidences of diabetes mellitus according to levels of Type A behaviour pattern index Men 4 (reference)  3 1.06 (0.91-1.22) 2 1.00 (0.84-1.20)  1 1.09 (0.94-1.27)  P for trend 0.381  Women 4 (reference)  3 0.93 (0.79-1.09)  2 1.03 (0.85-1.26)  1 1.22 (1.01-1.47)  P for trend 0.031  Odds ratios (95%CI) for the 10-year incidences of diabetes mellitus according to levels of constituent items of Type A behaviour pattern index Men Low (reference) Impatience (reference) Irritability (reference)  Aggressiveness (reference) Competitiveness (reference)  Medium Impatience 1.01 (0.85-1.18)  Irritability 1.08 (0.92-1.28)  Aggressiveness 1.05 (0.89-1.25)  Competitiveness 0.87 (0.73-1.03)   High Impatience 1.02 (0.86-1.22) Irritability 1.14 (0.95-1.37)  Aggressiveness 1.12 (0.92-1.36) Competitiveness 0.90 (0.74-1.09)  Women Low Impatience (reference) Irritability (reference)  Aggressiveness (reference) Competitiveness (reference)  Medium Impatience 1.05 (0.88-1.26)  Irritability 0.99 (0.80-1.23)  Aggressiveness 0.96 (0.80-1.15)  Competitiveness 0.99 (0.83-1.19)   High Impatience 1.23 (1.00-1.51) Irritability 1.16 (0.91-1.48)  Aggressiveness 1.08 (0.87-1.36) Competitiveness 1.01 (0.80-1.29)  Odds ratios (95% CI) for the 1 0-year incidences of diabetes mellitus according to coffee consumption Men almost never (reference) 1  1-2 days per week 0.93 (0.80-1.08) 3-4 days per week 0.84 (0.71-1.01) 1-2 cups/day 0.84 (0.73-0.97) 3-4 cups/day 0.83 (0.68-1.02) >5 cup/day 0.82 (0.60-1.112 p for trend 0.006   Women almost never (reference) 1  1-2 days per week 0.90 (0.76-1.06) 3-4 days per week 0.95 (0.77-1.17) 1-2 cups/day 0.81 (0.69-0.96) 3-4 cups/day 0.62 (0.45-0.84)  >5 cup/day 0.40 (0.20-0.78)  p for trend <0.001  **Significant trends** The risk of diabetes increased with an increasing stress level, especially among men. This association remained almost unchanged after adjustments for known risk factors of diabetes, type A and hours of sleep.  **Reported limitations  Author** assessment of diabetes mellitus was based on the results of a self-reported questionnaire. Perceived mental stress was assessed based on a single simple question  **Source of funding** Cancer Research H16-S2 and for the Third Term Comprehensive Ten-Year Strategy for Cancer Control H16-010, and Health Sciences Research grants (Medical Frontier Strategy Research H13-008, Clinical Research for Evidence-based Medicine H14-008, H15-006, Comprehensive Research on Cardiovascular Diseases H16-019, H17-019, H18-028, H19-019) from the Ministry of Health, Labour and Welfare of Japan. | |
| **Authors:** Kesse-Guyot E, Andreeva VA, Jeandel C, Ferry M, Hercberg S, et al.  **Year:** 2012  **Citation:** J Nutr. 2012;142: 909–915  **Country of study:** France  **Aim of study:** Evaluated association between empirically derived dietary patterns in midlife and cognitive performance  **Study design:** Randomized, double-blind, placebo-controlled, primary prevention trial  **Quality score: (++, + or -):**  **External validity score: (++, + or -):** | | | | | | | | | | | | | |
| **Number of people**  12,741  **Demographics** Not reported | | **Number of people** 3054 **Characteristics**  Cells J & K  **Location**  France  **Recruitment strategy** Not reported. At end of trial phase, the participants were invited for an additional 2-y follow-up for the SU.VI.MAX 2 observational study  **Length of follow-up**  13.4 ± 0.7 y.  **Response rate and loss to follow-up** Not reported  **Eligible population** Not reported  **Excluded populations** younger than 45 y at baseline (n = 1267), those with missing neuropsychological test scores (n = 1136), those with fewer than 3 dietary records during the first 2 y of follow-up (n = 1085), or those with missing data on any of the covariates (n = 308). dietary records that reported <100 or >6000 kcal/d were excluded; men reporting <800 kcal/d and women reporting <500 kcal/d in 60% or more of their dietary records were also excluded. | | | | **Relevant exposures** Diet **Time** 1995–1996 **Measurment of exposure** 24-h dietary records. Food and nutrient intakes were based on the reported mean intakes across all 24-h records | | **Outcomes** Cognitive performance  **outcome measurement** neuropsychological tests  **Time**  2007–2009 | | **Analysis strategy** ANCOVA  C**onfounders** age, gender, intervention group, education, alcohol and energy intake, number of dietary records, physical activity, BMI, tobacco use, self-reported memory troubles, diabetes, hypertension, follow-up time, history of cardiovascular disease, and depressive symptoms and, for women, menopausal status and hormone therapy use | | **Number** 3054  **Effect estimates** Healthy pattern  Global cognitive function  Q1 48.9 ± 0.7 Q2 49.4 ± 0.7 Q3 50.8 ± 0.7 Q4 50.1 ± 0.7  P2 0.001   Verbal memory Q1 49.1 ± 0.7 Q2 49.5 ± 0.7 Q3 50.6 ± 0.7 Q4 50.3 ± 0.7 P2 0.01  Executive functioning  Q1 49.3 ± 0.7 Q2 49.6 ± 0.7 Q3 50.6 ± 0.7 Q4 49.8 ± 0.7 P2 0.13  Traditional pattern Global cognitive function Q1 50.1 ± 0.7 Q2 49.5 ± 0.7 Q3 49.6 ± 0.7 Q4 49.9 ± 0.7 P2 0.68  Verbal memory Q1 50.2 ± 0.7 Q2 49.8 ± 0.7 Q3 49.6 ± 0.8 Q4 49.7 ± 0.7 P2 0.32  Executive functioning Q1 49.9 ± 0.7 Q2 49.5 ± 0.7 Q3 49.7 ± 0.7 Q4 50.1 ± 0.7 P2 0.60  **Significant trends** A healthy and a traditional DP were identified. In the multivariate model, the healthy pattern was associated with better global cognitive function (50.1 ± 0.7 vs. 48.9 ± 0.7; P-trend = 0.001) and verbal memory (49.7 ± 0.4 vs. 48.7 ± 0.4; P-trend = 0.01).  **Reported limitations  Author** cognitive evaluation was not available at baseline. Empirically derived DP showed some limitations regarding food groupings, factor selection, and labeling. Sample likely included the more compliant or health-conscious participants. Residual confounding  **Reviewer** Unclear reporting. From intervention study but little detail of impact of intervention on groups  **Source of funding** French National Research Agency (no. ANR-05-PNRA-010), the French Ministry of Health, Mederic, Sodexo, Ipsen, MGEN, and Pierre Fabre | |
| **Authors:** Khalili P, Nilsson PM, Nilsson JÅ, Berglund G  **Year:**  2002  **Citation:** J Hypertens. 2002;20:1759–1764  **Country of study:** Sweden  **Aim of study:** Examine to what degree smoking habits modulate the relationship between systolic blood pressure and risk for cardiovascular morbidity and mortality  **Study design:** Population-based screening study  **Quality score: (++, + or -):**  **External validity score: (++, + or -):** | | | | | | | | | | | | | |
| **Number of people**  22 444  **Demographics** Not reported | | **Number of people** Not reported **Characteristics**  Q1  n 3249 Age (years) 42.2 Smoking habits* (%) 59.0/41.0 BMI (kg/m2 ) 23 (2.8)  Q2 n 6846 Age (years) 42.6 Smoking habits* (%) 51.9/48.1 BMI (kg/m2 ) 24 (2.9)  Q3 n 2272 Age (years) 43.0 Smoking habits* (%) 50.0/50.0 BMI (kg/m2 ) 25 (3.2)  Q4  n 4947 Age (years) 44.0 Smoking habits* (%) 45.9/54.1 BMI (kg/m2 ) 25 (3.1)  Q5 n 4215 Age (years) 46.0 Smoking habits* (%) 43.9/56.1 BMI (kg/m2 ) 26 (3.6)  treated hypertensives n 915 Age (years) 48.4 Smoking habits* (%) 38.9/61.1 BMI (kg/m2 ) 27 (3.9)  **Location**  Malmo, Sweden  **Recruitment strategy** Not reported  **Length of follow-up**  Averaged a mean of 17 years  **Response rate and loss to follow-up** mean attendance rate 71%  **Eligible population** middle-aged men  **Excluded populations** | | | | **Relevant exposures** smoking **Time** Not reported **Measurement of exposure** self-reported questionnaire | | **Outcomes** cardiovascular events  **outcome measurement** local and national registers. A first cardiovascular event was defined as the first recorded cardiovascular event during follow-up, including fatal and non-fatal cases of ischaemic heart disease and cerebrovascular disease  **Time**  end of 1996 | | **Analysis strategy** calculated using direct standardization and expressed as risk ratios with 95% confidence intervals  **Confounders** age, systolic blood pressure, diastolic blood pressure, body mass index, cholesterol, triglycerides, or history of diabetes | | **Number** Not reported  **Effect estimates** Morbidity RR (95%CI) between smokers and non-smokers Q1 1.9 (1.5–2.4) Q2 2.1 (1.8–2.5) Q3 2.3 (1.8–2.9) Q4 1.8 (1.5–2.1) Q5 1.7 (1.5–2.0) tHTs 1.4 (1.1–1.8).  Mortality RR (95%CI) between smokers and non-smokers Q1 1.8 (1.4–2.3) Q2 2.5 (2.1–3.0) Q3 2.7 (2.0–3.6) Q4 2.2 (1.9–2.7) Q5 2.5 (2.1–2.9) tHTs 1.8 (1.3–2.5)  **Significant trends** Increasing systolic blood pressure levels is associated with an increasing risk of future cardiovascular events and mortality, an association modified by smoking habits. Treated hypertensive patients were at increased risk in spite of antihypertensive drugs  **Reported limitations  Author** Unclear reporting.   **Reviewer** Not reported  **Source of funding** Swedish Society of Medicine | |
| **Authors:** Kimm H, Lee PH, Shin YJ, Park KS, Jo J, et al.  **Year:** 2011  **Citation:** Arch Gerontol Geriatr. 2011;52;e117–e122  **Country of study:** Republic of Korea  **Aim of study:** Determine the effects of vascular risk factors, such as blood pressure, diabetes and smoking in the mid-life or the late-life on dementia risk  **Study design:** Prospective cohort study  **Quality score: (++, + or -):**  **External validity score: (++, + or -):** | | | | | | | | | | | | | |
| **Number of people**  1,329,525  **Demographics** Not reported | | **Number of people** 848,505 **Characteristics**  Men Number 490,445  Age at enrollment (year) 51.9 ±8.7 BMI (kg/m2) 23.3± 2.3 Alcohol drinking (g/day) 15.4 ± 32.2 Diabetes 6.8 Hypertension 42.8 Smoking status Never smokers 21.4 Ex-smokers 22.6 Current smokers 56.0 Any alcohol use 73.1  Women Number 358,060  Age at enrollment (year) 53.6 ±9.9 BMI (kg/m2) 23.8± 3.1 Alcohol drinking (g/day) 0.1 ± 1.4 Categorical variables (%)  Diabetes 5.1 Hypertension 35.2 Never smokers 92.4 Ex-smokers 2.5 Current smokers 5.1 Any alcohol use 13.6  **Location**  Korea  **Recruitment strategy** participated in at least one biennial National Health Insurance Corporation (NHIC) medical evaluation between 1992 and 1995  **Length of follow-up**  14 years  **Response rate and loss to follow-up** 784,870 (59.0%) enrolled in 1992; 367,903 (27.7%) in 1993; 98,417 (7.4%) in 1994; and 78,335 (5.9%) in 1995  **Eligible population** Koreans aged 40–95 years insured by the National Health Insurance Corporation who had a biennialmedical evaluation during 1992–1995  **Excluded populations** 904 participants who died before January 1, 1993, were excluded, as were 75,807 participants who reported having cardiovascular disease, cancer, liver disease, or a respiratory disease at or prior to their initial visit, and 17,933 participants with missing information on body mass index or alcohol consumption, or with extremely low levels of BMI (<16 kg/m2) or height ( 1.30 m). | | | | **Relevant exposures** alcohol drinking, and smoking **Time** 1992-1995 **Measurement of exposure** Self-report. participants were classified into ‘‘current’’ smoker if they smoked currently for at least 1 year, ‘nonsmokers’ if they never smoked, and ex-smokers if they had quit smoking | | **Outcomes** Dementia events  **outcome measurement** The category included dementia in AD (ICD-10 code: F00), VaD (F01), and unspecified dementia (F03). Diagnostic and statistical manual of mental disorder (DSM-IV, 4th edition), along with historical, physical, neurological, neuropsychological, laboratory and imaging evaluation  **Time**  January 1993 to December 2006 | | **Analysis strategy** Cox proportional hazard model  **Confounders** age, hypertension, total cholesterol, alcohol drinking, and smoking | | **Number** 3252  **Effect estimates** HR (95% CI) Men AD  Diabetes 1.6(1.3–2.0) Ex-smokers 1.0(0.8–1.2) Current smokers 1.1(0.9–3.4) Pre-hypertension 1.1(0.9–1.4) Stage 1 hypertension 1.3(1.0–1.6) Stage 2 hypertension 1.4(1.1–1.8) Borderline cholesterol. 1.2(1.0–1.4) High cholesterol 1.2(1.0–1.5)  VaD  Diabetes 2.0(1.5–2.8) Ex-smokers 0.9(0.7–1.3) Current smokers 1.1(0.8–1.5) Pre-hypertension 1.0(0.7–1.5) Stage 1 hypertension 1.7(1.2–2.5) Stage 2 hypertension 2.6(1.7–3.8) Borderline cholesterol. 1.3(1.0–1.6) High cholesterol 1.1(0.8–1.6)  Unspec.  Diabetes 1.3(1.0–1.7) Ex-smokers 0.9(0.7–1.2) Current smokers 1.2(1.0–1.5) Pre-hypertension 1.0(0.8–1.3) Stage 1 hypertension 1.2(0.9–1.5) Stage 2 hypertension 1.5(1.1–2.0) Borderline cholesterol. 0.9(0.7–1.0) High cholesterol 1.0(0.8–1.3)  All Diabetes 1.6(1.3–1.8) Ex-smokers 1.0(0.8–1.1) Current smokers 1.2(1.0–1.3) Pre-hypertension 1.0(0.9–1.2) Stage 1 hypertension 1.3(1.1–1.5) Stage 2 hypertension 1.6 (1.4.–1.9) Borderline cholesterol. 1.0(0.9–1.2) High cholesterol 1.1(0.9–1.3)  Women AD  Diabetes 1.4(1.1–1.7)  Ex-smokers 1.2(0.9–1.5)  Current smokers 1.3(1.1–1.5)  Pre-hypertension 1.1(0.9–1.3)  Stage 1 hypertension 1.1(0.9–1.3)  Stage 2 hypertension 1.2(0.9–1.4)  Borderline cholesterol 1.0(0.9–1.2)  High cholesterol 1.1(0.9–1.3)   VaD  Diabetes 2.8(2.0–3.9) Ex-smokers 0.9(0.5–1.5) Current smokers 1.5(1.1–2.1) Pre-hypertension 1.2(0.8–1.7) Stage 1 hypertension 1.5(1.0–2.2) Stage 2 hypertension 2.3(1.6–3.3) Borderline cholesterol 1.1(0.8–1.4) High cholesterol 0.9(0.7–1.3)  Unspec.  Diabetes 1.4(1.1–1.9)  Ex-smokers 0.9(0.7–1.2) Current smokers 1.2(1.0–1.5) Pre-hypertension 1.1(0.9–1.4) Stage 1 hypertension 1.2(1.0–1.5) Stage 2 hypertension 1.4(1.1–1.7) Borderline cholesterol 1.0(0.8–1.2) High cholesterol 1.1(0.9–1.4)  All Diabetes 1.6(1.4–1.9) Ex-smokers 1.1(0.9–1.3) Current smokers 1.3(1.1–1.5) Pre-hypertension 1.1(1.0–1.3) Stage 1 hypertension 1.1(1.0–1.3) Stage 2 hypertension 1.3(1.2–1.6) Borderline cholesterol 1.0(0.9–1.1) High cholesterol 1.0(0.9–1.2)  **Significant trends** diabetes increased the risk of either dementia in Alzheimer’s disease or vascular dementia in men and women  **Reported limitations  Author** accuracy of dementia data used from hospitalization of NHIC has not been validated as for an outcome measurement  **Source of funding** Seoul City R&BD program [10526] | |
| **Authors:** King DE, Mainous AG, Geesey ME  **Year:**  2007  **Citation:** Am J Med. 2007;120:598-603  **Country of study:** USA  **Aim of study:** Determine the frequency of adopting a healthy lifestyle in a middle-aged cohort, and determine the subsequent rates of cardiovascular disease and mortality among those who adopt a healthy lifestyle  **Study design:** Cohort study  **Quality score: (++, + or -):**  **External validity score: (++, + or -):** | | | | | | | | | | | | | |
| **Number of people**  15,792  **Demographics** not reported | | **Number of people** 15,708  **Characteristics**  (n = 15,708) % Age 45-54 52.7 55-64 47.3  Gender Male 44.8 Female 55.2  Race Other than African American 73.0 African American 27.0  Education <High school 24.0 High school or trade school 40.7 College 35.3  Family Income <$35,000/yr 57.8 >$35,000/yr 42.2  Hypertension Yes 47.0 No 53.0  Diabetes Yes 7.6 No 92.4  High Cholesterol Yes 64.3 No 35.7  Coronary Heart Disease Yes 4.9 No 95.1  **Location**  four communities across the United States  **Recruitment strategy** probability sampling then selection involves sampling age-eligible persons from listings and then identifying their households  **Length of follow-up**  6 years  **Response rate and loss to follow-up** Not reported  **Eligible population** adults age 45-64  **Excluded populations** Not reported | | | | **Relevant exposures** Diet, smoking, physical activity **Time** 1987 to 1989 **Measurement of exposure** The ARIC dietary questionnaire consisted of items regarding the frequency of consumption of various foods over the previous year. A healthy lifestyle was characterized by having all 4 of the following lifestyle characteristics: eating at least 5 fruits and vegetables daily; exercising minimum of 2.5 hours per week; BMI maintained between 18.5 and 30 kg/m2; and not smoking  For each physical activity the hours per week and the months per year are reported, and these values then were used to calculate the average number of hours per week spent on that activity over the course of the year  Current smokers identified by questionnaire during each visit. | | **Outcomes** all-cause mortality and fatal or non-fatal cardiovascular disease  **outcome measurement** state death certificates  identified participants who developed fatal or non-fatal cardiovascular disease from those whose underlying cause of death was coded as cardiovascular disease, or who had an MI, a silent MI, diagnosed coronary heart disease, a coronary heart disease procedure, or a definite or probable stroke  **Time**  end of the year 1998 | | **Analysis strategy** multiple logistic regression  **Confounders** age group, gender, race, education, family income, and histories of hypertension, diabetes, elevated cholesterol, and previous coronary heart disease | | **Number** Not reported  **Effect estimates** Switchers to Healthy Lifestyle and Persistently Healthy Compared to Persistently Unhealthy Individuals  Cardiovascular Disease Event OR (95% CI) Switched from Unhealthy to Healthy Lifestyle 0.65 (0.52-0.81)  Persistently Unhealthy (<4 Healthy Factors at Both Visits) 1.00 (reference)  Death OR (95% CI) Switched from Unhealthy to Healthy Lifestyle 0.60 (0.39-0.92) Persistently Unhealthy (<4 Healthy Factors at Both Visits) 1.00 (reference)  **Significant trends** individuals who are older, female, with a college education, with family annual incomes greater than $35,000, or with no history of hypertension are more likely to have switched than others  **Reported limitations  Author** misclassification via exaggerated exercise frequency or intake of fruits and vegetables  **Reviewer** Unclear no. of deaths  **Source of funding** Not reported | |
| **Authors**: [Knopman D](http://www.ncbi.nlm.nih.gov/pubmed?term=Knopman%20D%5BAuthor%5D&cauthor=true&cauthor_uid=11148234), [Boland LL](http://www.ncbi.nlm.nih.gov/pubmed?term=Boland%20LL%5BAuthor%5D&cauthor=true&cauthor_uid=11148234), [Mosley T](http://www.ncbi.nlm.nih.gov/pubmed?term=Mosley%20T%5BAuthor%5D&cauthor=true&cauthor_uid=11148234), [Howard G](http://www.ncbi.nlm.nih.gov/pubmed?term=Howard%20G%5BAuthor%5D&cauthor=true&cauthor_uid=11148234), [Liao D](http://www.ncbi.nlm.nih.gov/pubmed?term=Liao%20D%5BAuthor%5D&cauthor=true&cauthor_uid=11148234), et al. **Year:** 2001 **Citation:** [Neurology.](http://www.ncbi.nlm.nih.gov/pubmed/11148234) 2001;56(1):42-48  **Country of study:** US **Aim:** To determine the influence of smoking on cognitive change **Study design:** Longitudinal **Quality score: +**  **Applicability: +** | | | | | | | | | | | | | |
| **Source population:**  *15,792 men and women ages 45-64 years recruited from Forsyth County, NC; Jackson, MS; suburban Minneapolis, MN; and Washington County, MD in 1987-89 using probability samples  *clinical examination administered in 1987-89 with response rate of 46% in Jackson, and 65% in the other communities  *13% of Forsyth County sample was black; the other sites consisted of predominantly white individuals | | **Study population:**  *in 1990-92 (baseline for this study), 14,348 participants underwent cognitive evaluation with follow-up 6 years later, during which 10,963 respondents (76%) were re-tested  *Sociodemographics:*  *****8,729 white individuals and 2,234 black individuals  *6% of subjects had less than 9^th^ grade education  *Follow-up:*  *6 years follow-up since 1990-92 (baseline)  **Exclusion:**  *individuals with history of stroke or TIA (n=300)  **Attrition:**  *****participants who dropped out or died were more impaired than those who returned for follow-up | | | | **Exposures:**  *self-reported smoking (never, former, current categories) | | **Outcomes:**  *cognitive change: change in follow-up scores minus baseline scores for cognitive testing using: Delayed Word Recall (DWR) test, the Digit Symbol Subtest (DSS) of the Wechsler Adult Intelligence Scale-Revised, First Letter World Fluency (WF) test | | **Analysis:**  *linear regression was used to determine the influence of smoking on cognitive change  **Confounders:**  *****age, gender, race-center, education level, and use of  CNS medications | | **Results:**  *smoking was not associated withdeclines on any of the cognitive tests  **Limitations:**  *limited cognitive battery  **Source of funding:**  *none reported | |
| **Authors**: Lahti J, Laaksonen M, Lahelma E, Rahkonen O **Year:** 2010 **Citation:** Prev Med. 2010;50(5-6):246-250 **Country of study:** Finland **Aim:** To determine the impact of physical activity on physical health functioning  **Study design:** Longitudinal **Quality score:** + **Applicability:** | | | | | | | | | | | | | |
| **Source population:**  *baseline questionnaire was administered to 13,346 employees ages 40, 45, 50, and 55 in Helsinki during 2000, 2001, and 2002 (response rate: 67%) *at follow-up, in 2007, 7330 completed the survey (response rate: 83%) | | **Study population:**  *analysis restricted to **5,437** women and **1,257** men  *Sociodemographics:* *mean ages of women and men were 49 and 51, respectively **Exclusion:**  *participants with missing information (n=636)  **Attrition:**  - | | | | **Exposures:**  *the average weekly hours of physical activity during leisure time or community during the last 12 months were used to calculate the volume of physical activity  *time spent in physical activity and intensity were used to create the following participant categories: inactive, active moderate, active vigorous or vigorous activity only, very active moderate, very active vigorous, conditioning (moderate and vigorous activity or vigorous activity only) | | **Outcomes:**  *physical health functioning was measured by the Short-Form 36 (SF-36) *high scores on the SF-36 are related to better health *the SF-36 demonstrates good construct validity, has high internal consistency and test-retest reliability | | **Analysis:**  *sex-specific proportions were calculated to determine the percentage of participants with poor or good physical health functioning at follow-up by baseline physical activity *means were also computed **Confounders:**  *age, baseline physical health functioning and limiting longstanding illness, working conditions, working overtime and BMI, smoking and alcohol consumption | | **Results:**  *the more vigorous the physical activity, the greater the physical functioning mean scores appeared to be for both sexes *among women, the lowest percentage of poor physical health functioning at follow-up was reported for the active vigorous group (22%, [95% CI: 19%, 25]) *among men, the lowest percentage of poor physical health functioning was reported for the active moderate and active vigorous group (22%, [17%, 27%], and 22%, [16%, 28%], respectively)  *among women, the highest percentage of good physical health functioning at follow-up was reported for the very active moderate group (27%, [24%, 30%]), while for men it was reported for the conditioning group (29%, [24%, 35%])  **Limitations:**  *limited generalizability as sample consisted predominantly of relatively healthy, middle-aged, public sector female employees *reverse causality may be possible as sicker participants may be less able to engage in physical activity  *changes in levels or intensity of physical activity may have occurred during follow-up *self-reported data **Source of Funding:**  *Ministry of Education, the Yrjö Jahnsson Foundation, the Juho Vainio Foundation, and the Academy of Finland | |
| **Authors**: Laitala VS, Kaprio J, Koskenvuo M, Räihä I, Rinne JO, et al. **Year:** 2009 **Citation:** Am J Clin Nutr. 2009;90(3):640-646 **Country of study:** Finland **Aim:** To determine whether coffee consumption protects against cognitive decline in a sample of Finnish twins **Study design:** Longitudinal **Quality score:** ++ **Applicability:** | | | | | | | | | | | | | |
| **Source population:**  *13,888 same sex twin pairs born before 1958 in Finland were recruited and completed a self-administered questionnaire in 1975 and 1981 (response rates: 89% and 84%, respectively) *twins ages 65 years or greater had their cognitive status assessed through telephone interview: monozygotic twins with both twins alive interviewed between 1999-01, same sex dizygotic twins and twins of uncertain zygosity between 2003-07 | | **Study population:**  ***2483 twins** of known zygosity (703 monozygotic twins and 1780 dizygotic twins) and **123** twins of uncertain zygosity  *overall response rate: 79% *Sociodemographics:* *mean age of respondents of 46 years in 1975 and 52 years in 1981   *Follow-up:* *median follow-up of 28 years **Exclusion:**  *participants not reached by phone (n=127) *declined to participate (n=412) *died (n=32) *not contacted or with incomplete interviews (n=133)  **Attrition:**  *participants and non-participants comparable in terms of sex, education, and alcohol use patterns | | | | **Exposures:**  *daily coffee drinking was assessed in 1975 and 1981 *if coffee consumption was reported at both times, the mean intake was calculated  *participants were categorized into the following groups of intake: 0–3, 3.5–8, and >8 cups/d | | **Outcomes:**  *potential dementia cases identified through TELE screening and the Telephone Interview for Cognitive Status (TICS) *based on these screening tests, scores of cognitive function were assigned (higher scores indicate better cognitive function) and participants were classified as demented, cognitively declined, and healthy *TELE and TICS and sensitive and specific and correlate well with the MMSE | | **Analysis:**  *multinomial logistic regression was used to assess the association between coffee intake and mild cognitive impairment or dementia, with twins being treated as individuals, not pairs (clustering taken into account) *the association between cognitive performance and coffee intake was also assessed for discordant twin pairs **Confounders:**  *education, age at the interview, sex, BMI, binge drinking, smoking, life satisfaction, cardiovascular disease, hypercholesterolemia, hypertension, and diabetes | | **Results:**  *The prevalence of dementia according to the TELE and TICS was: 9.8% and 7.8% (respectively) for those ages 65-69 years; 11.9% and 7.9% for those ages 70-74 years; 19.1% and 15.6% for those ages 75-79 years; 31.7% and 25.3% for those ages 80-84 years; and 53.0% and 47.0% for those older than 84 years  *coffee consumption was not associated with dementia or mild cognitive impairment *the twin-pair analysis of twins discordant for coffee consumption and cognitive score showed that the correlation between cognitive function and coffee drinking was not statistically significant  **Limitations:**  *unable to assess if rate of coffee consumption affects rate of cognitive decline  **Source of Funding:**  *none reported | |
| **Authors**: Laitinen MH, Ngandu T, Rovio S, Helkala EL, Uusitalo U, et al. **Year:** 2006 **Citation:** Dement Geriatr Cogn Disord. 2006;22(1):99-107 **Country of study:** Finland **Aim:** To determine the association between fat intake at midlife and risk of dementia and Alzheimer’s disease **Study design:** Longitudinal **Quality score:** ++  **Applicability:** | | | | | | | | | | | | | |
| **Source population:**  *randomly selected participants from the survivors of population-based samples from North Karelia and Kuopio provinces; samples drawn from the national population register *at least 250 subjects of each sex and 10-year age group chosen *62.1% (n=900) and 37.9% (549) of participants were women and men, respectively | | **Study population:**  *a random sample of **1449** participants examined once in 1972, 1977, 1982, or 1987 were re-examined in 1998 (ages 65-79 years)  *Sociodemographics:* *mean age at midlife examination was 50.4 years and 71.3 years at follow-up  *participation rate: 77%-96%  *Follow-up:* *mean length of follow-up 21 years  **Exclusion:**  -  **Attrition:** | | | | **Exposures:**  *dietary habits assessed through self-administered questionnaire *most questions were qualitative or frequency-based; consumption of milk, sour milk, eggs, coffee, tea, sugar in tea/coffee assessed quantitatively *daily number of bread slices and type of spread used and amount of spread per slice (i.e. 0, 2.5, 5, 10, 15 g) *fat intake from milk, sour milk and spreads calculated – served as indicator of total fat intake *calculated polyunsaturated [PUFA], monounsaturated [MUFA], saturated fatty acids [SFA] derived from spreads | | **Outcomes:**  *Cognitive status assessed using MMSE; if score <=24, invited to clinical phase for dementia diagnosis | | **Analysis:**  Logistic regression used to assess influence of fat intake and dementia and AD development  **Confounders:**  *midlife age, gender, education, follow-up time, milk fat and other subtypes of fats from spreads, midlife vascular risk factors including smoking status, systolic blood pressure, cholesterol and BMI, history of vascular disorders including MI, stroke, and diabetes; - effect modification of APOE4 by fat intake assessed | | **Results:**  *117 diagnosed with dementia, of which 76 had AD *proportion of demented persons was higher among those having used very little or no fat (from milk and spreads) compared to moderate users (31.6% [n=37] and 24.6% [n=458], respectively; p-value=0.02) *28.2% [n=33] and 24.8% [n=462] of demented and non-demented people consumed large amounts of fats  *odds of developing dementia were lower for those consuming moderate amounts of polyunsaturated fats from spreads compared to those consuming low amounts [OR=0.40, 95% CI: 0.17-0.94; and 2] *odds of developing dementia and AD were higher for those consuming moderate amounts of saturated fats from spreads in comparison with those consuming low amounts [OR=2.45, (1.10-5.47), and OR=3.82, (1.48-9.87), respectively] *Among APOE4 carriers, the odds of developing dementia was lower for those with moderate PUFA intake compared to those with low PUFA intake [OR=0.29, 95% CI: 0.09-0.89]; the reverse was observed for SFA  **Limitations:**  *limited reliability of self-reported dietary data *residual confounding, i.e. intake of fat from other sources and other nutrients may influence association with dementia/AD *long-term fat intake may be more likely to influence disease risk  *selective survival: those with high SFA intake may have died from vascular disease – may differ from survivors *misclassification of dementia diagnosis by using medical records (under-diagnosis)  **Source of Funding:**  *EVO grants from Kuopio University Hospital, Academy of Finland, Alzheimer Association, EU, Finnish Cultural Foundation of Northern Savo, the Foundation of Juho Vainio, and the Gamla Tjanarinnor Foundation, the Swedish Council for Working Life and Social Research | |
| **Authors**: Lajous M, Willett WC, Robins J, Young JG, Rimm E, et al. **Year:** 2013 **Citation:** Am J Epidemiol. 2013;178(3):382-391 **Country of study:** US **Aim:** To determine whether changes in fish consumption in midlife affect the risk of coronary heart disease  **Study design:** Longitudinal **Quality score:** ++ **Applicability:** | | | | | | | | | | | | | |
| **Source population:**  *51,529 US male health professionals ages 40-75 years enrolled in 1986 (Health Professionals Follow-up Study)  *121,701 US female nurses ages 30-55 years enrolled in 1976 (Nurses’ Health Study) | | **Study population:**  *see 'Source population'*Follow-up - Health Professionals Follow-up Study:* *1990 until CHD diagnosis, death, censoring, or June 2008, whichever came first  *Follow-up – Nurses’ Health Study:* *time of return of 1986 participant questionnaire to CHD diagnosis, death, censoring, or December 2008, whichever came first  **Exclusion:**  Health professionals who **(male [n], female [n])**: *did not respond or provided insufficient information for survey (n=14,485, 55,328) *died (n=779, 92) *had MI (n=432, 6) *had CVD, diabetes, cancer (n=6,300, 9,653) *had missing variable data or implausible energy intake (n=4,726, 2,850)  **Attrition:** - | | | | **Exposures:**  *fish intake and red meat intake were assessed using a semiquantitative food frequency questionnaire that was first administered to males in 1986 and females in 1984 and 1986, and both sexes every 4 years thereafter  *validated questionnaire evaluated average consumption of specific portion sizes of food items in the past year, with response options ranging from ‘never or less than once a month’ to ‘6 or more per day’ *food item intake was summed and daily nutrient intakes were based on nutrient content of specified portion and frequency of food item intake *total nutrient and energy intake was calculated and based on the sum of nutrient intakes from different foods *total alcohol intake was also calculated | | **Outcomes:**  *total, nonfatal, and fatal CHD were assessed *nonfatal myocardial infarctions occurring during follow-up were identified using medical records, while deaths were identified through relatives, postal authorities, or the National Death Index (medical records, death certificates, autopsies used to identify cause of death) | | **Analysis:**  *Hypothetical interventions:* *a randomized trial of fish intake on 18-year risks of CHD outcomes in men and 22-year risks of CHD outcomes in women was emulated using observational data *the effect of the following hypothetical ‘threshold’ interventions was assessed: intake of fish (measured in servings/week) of: 1) 0; 2) at least 1; 3) at least 2; 4) at least 3; or 4) at least 5 (servings/week) *at each 4-year interval, fish intake is increased to the threshold for those who eat fewer fish servings than the threshold (e.g., the threshold for intervention 3) is at least 2 servings/week); otherwise, it remains unchanged *‘isocaloric’ interventions were also considered in which red meat intake was replaced by fish intake to achieve the following serving amounts: 6) at least 1 (serving/week); 7) at least 2; 8) at least 3; 9) at least 5  *increase to the threshold was not undertaken if neither fish nor red meat consumption was reported  *Analysis:* *the standardized risk of CHD outcomes under hypothetical interventions was assessed using the parametric g-formula *pooled logistic and linear regression models were used to estimate probability density functions *Monte Carlo simulations were conducted  **Confounders:** *age, parental history of myocardial infarction, oral contraceptive use, body mass index, smoking, menopausal status, hormone replacement therapy, physical activity, aspirin use, vitamin E supplement use, multivitamin supplement use, high blood pressure, high cholesterol, diabetes, angina or coronary artery bypass grafting, stroke, and intakes of calories, trans-fats, alcohol, cereal fiber, red meat, and fish | | **Results:**  *no significant associations were reported for males  Females: Meat replaced with fish: *the lowest risk for *total coronary heart disease* was reported when meat was replaced with fish to attain >=3 servings/week (Risk ratio=0.81, [0.68, 0.95])  *the lowest risk for *fatal coronary heart disease* was reported when: meat was replaced with fish to attain >=5 servings/week (Risk ratio=0.68, [0.37, 0.99]); and when >=5 fish servings/week were consumed (Risk ratio=0.66, [0.36, 0.98])  **Limitations:**  *measurement error when attempting to quantify dietary change *potential residual confounding; particularly in men, as they may have been more focused on preventing CVD and making dietary changes in presence of slightly elevated BP or glucose/lipid levels **Source of Funding:**  *National Institutes of Health | |
| **Authors**: Lang IA, Guralnik JM, Melzer D **Year:** 2007 **Citation:** J Am Geriatr Soc. 2007;55(11):1836-1841 **Country of study:** US **Aim:** To assess whether body mass index and level of physical activity are associated with impaired physical function **Study design:** Longitudinal **Quality score:** + **Applicability:** | | | | | | | | | | | | | |
| **Source population:**  *Health and Retirement Study(HRS):* *10,086 US participants ages 50-59 years at baseline in 1998 and still alive in 2004 *after baseline face to face interviews, phone interviews were conducted every even-numbered year   *English Longitudinal Study of Aging (ELSA):* *19,924 English participants born on or before February 29, 1952 were identified for cross-sectional survey in 1998, 1999, 2001 *11,392 individuals recruited for ELSA study (eligible respondents 65.7%) | | **Study population:**  *HRS:* *analysis restricted to **8,702** participants  *Sociodemographics:*  *4569 were women and 1,031 had 0-9 years of full-time education, baseline mean age of 60.2 years   *ELSA:*  *analysis restricted to **1,507** out of 3,335 respondents ages 50-69 years with baseline exposure information measured in 1998-99, who participated in second ELSA wave in 2004, and had recommended weight or above and were nondisabled at baseline  *Sociodemographics:*  *837 were women and 134 had 0-9 years of full-time education, baseline mean age of 58 years   *Median length of follow-up:*  *HRS: 72 months *ELSA: 73 months **Exclusion:**  *HRS:* *BMI <20 (353) *participants with one or more mobility problems (n=1,370)   *ELSA:* *participants who died or were ineligible for follow-up (n=2,596) *BMI <20 (n=64) *participants with long-term conditions (n=1,764)  **Attrition:** - | | | | **Exposures:**  *BMI:* *in HRS, self-reported weight and height at baseline and follow-up used to calculate BMI, which was classified as: recommended weight (20-24.9), overweight (25-29.9), or obese (>=30) *in ELSA, weight and height measured by clinicians at baseline and follow-up, and same BMI categories used as for HRS   *Physical activity:* *In HRS, participants asked about participation in vigorous physical activity (e.g., sports) at least 3 times/week over last 12 months, with yes/no answer options  *In ELSA, respondents asked about the number of days/week that they undertook activities, such as housework or gardening, manual labour, participating in vigorous sports for 30 minutes or longer, and a summary measure was subsequently created *ELSA participants were also categorized into whether or not they participated in vigorous activity at least 3 days/week | | **Outcomes:**  *Physical function* *mobility impairment was ascertained for HRS participants through questions on difficulty with walking or climbing stairs *measured physical performance impairment in ELSA respondents was measured by clinician using a modified version of the Short Physical Performance Battery that assessed balance, chair stands, and grip strength; a physical performance score out of 12 was allocated to each participant (with 7 or less representing impairment) | | **Analysis:**  *logistic regression was used to assess the influence of physical activity on incident impaired physical function (ELSA) and self-reported mobility impairment (HRS) by baseline BMI category  **Confounders:**  *age, sex, health behaviors, socioeconomic status, and baseline functional limitations, smoking, drinking | | **Results:**  *participants who reported being active at least 3 times per week had a lower incidence of physical impairment than those with lower levels of reported physical activity for each weight category in both HRS and ELSA  **Limitations:**  *self-reported physical activity **Source of Funding:**  *National Institutes of Health Award, Intramural Research Program, National Institute on Aging, NIH | |
| **Authors**: Langlois JA, Mussolino ME, Visser M, Looker AC, Harris T, et al. **Year:** 2001 **Citation:** Osteoporos Int. 2001;12(9):763-768 **Country of study:** US **Aim:** To determine the influence of weight loss in middle-aged and older women and risk for hip fracture  **Study design:** Longitudinal **Quality score:** + **Applicability:** | | | | | | | | | | | | | |
| **Source population:**  *US women surveyed on nutrition and weight history in 1971-75 (baseline) *14,407 individuals ages 25-74 years participated in study follow-up waves in 1982-84, 1986, 1987, and 1992 | | **Study population:**  *study restricted to **2180** out of 2410 community-dwelling white women ages 50–74 years who met inclusion criteria  *Follow-up:* *33,174 person-years of observation during 22 years *time from date of examination to date of hip fracture, or date of last follow-up interview, or date of death for those without a hip fracture **Exclusion:**  women with: *a history of hip fracture at baseline (n=51) *loss to follow (n=139) *missing data (n=40)  **Attrition:**  *139 women lost to follow-up *at baseline, excluded women were older, less physically active, and weighed less than participants | | | | **Exposures:**  *body weight and weight history self-reported at baseline in 1971-75 *self-reported maximum lifetime weight was ascertained and the percent weight loss was calculated ([maximum weight-baseline weight / maximum weight]*100) *proportional weight loss categorized into: 1) <5%; 5% to <10% and >=10%; and 2) <10% and >=10% | | **Outcomes:**  *hip fractures identified through death certificates and hospital records | | **Analysis:**  *cox proportional hazards regression was used to determine the influence of weight loss on hip fracture risk  **Confounders:**  *age at baseline, body mass index at maximum weight, smoking, alcohol consumption in the past year, history of chronic conditions and level of non-recreational physical activity  Stratification: *BMI, age | | **Results:**  *171 hip fractures identified *mean age at hip fracture was 71.7 years for women ages 50-64 years, and 81.1 years for women ages 65- 74 years *incidence rates of hip fracture appeared to increase with increasing age and weight loss  women aged 50-64 years at baseline: *women with >=10% weight loss had a greater risk of hip fracture compared to women with <5% weight loss (RR=2.54, [1.10, 5.86])  women aged 65-74 years at baseline: *women with >=10% weight loss had a greater risk of hip fracture compared to women with <5% weight loss (RR=2.04, [1.37, 3.04]) *when <10% weight loss was used as the reference category, the relative risks changed minimally for both age groups  women with BMI <26.2 (kg/m2) at baseline: *women with >=10% weight loss had a greater risk of hip fracture compared to women with <5% weight loss (RR=2.37, [1.32, 4.27])  **Limitations:**  *cannot generalize to other age groups, races, or older men *self-reported weight (potentially bias association towards the null) *weight fluctuation was not taken into account **Source of Funding:**  *none reported | |
| **Authors**: Laurin D, Masaki KH, Foley DJ, White LR, Launer LJ **Year:** 2004 **Citation:** Am J Epidemiol. 2004;159(10):959-967 **Country of study:** US **Aim:** To determine the influence of midlife dietary intake of antioxidants on risk of late-life incident dementia **Study design:** Longitudinal **Quality score:** + **Applicability:** | | | | | | | | | | | | | |
| **Source population:**  *8,006 Japanese-American men, born between 1900-19, and residing in Oahu, Hawaii in 1965 were clinically evaluated and interviewed in 1965-68 when they were aged 45-68 years (examination 1), with follow-up examinations/interviews in 1968-70 (examination 2) and 1971-74 (examination 3)  *3,734 people (80% of those eligible) ages 71-93 years participated in 1991-93 survey on neurodegenerative disease (examination 4) with 2 re-examinations for dementia after assessments in 1994-96 (examination 5) and 1997-99 (examination 6) | | **Study population:**  *analysis restricted to **2,459** participants free of dementia in 1991-93 and who underwent follow-up examinations until 1999*Follow-up:* *30.2 years  *Sociodemographics:* *mean ages at examinations 1 and 4 were 51.2 and 76.3 years *median number of years of education was 12  **Exclusion:**  Participants with: *dementia (n=226) *atypical diet (n=226) *died during follow-up (n=516) *nonresponse at examination 5 (n=307)  **Attrition:**  *excluded individuals with atypical diet were similar to participants with respect to age, education, and BMI at exam. 1 and intake of supplemental vitamins *those who died during follow-up were, at exam. 1, older, less educated, reported lower energy intake and were less likely to use supplements *non-responders at examination 5 were older at exam. 1, had lower intakes of beta-carotene and vitamin C, and were less likely to use supplements | | | | **Exposures:**  *24-hour dietary recall assessed through interview in 1965-68 *participants also queried on frequency of consumption of 26 food and drink items at examinations 1 and 3 *flavonoid intake was assessed through mean intake of tea assessed at examinations 1 and 3 *intakes of beta-carotene, vitamin C, vitamin E, and flavonoids were divided into quartiles; the median energy-adjusted intakes for the first, second, third, and fourth quartiles, respectively, were: 16, 71, 296, and 1,101 μg/day for beta-carotene; 23, 69, 128, and 219 mg/day for vitamin C, 3.8, 10.7, 18.0, and 29.9 mg/day for vitamin E; and 2.0, 2.9, 4.5, and 8.2 mg/day for flavonoids | | **Outcomes:**  *dementia was ascertained through screening using the Cognitive Abilities Screening Instrument and evaluated through a neurologic examination, neuropsychological testing, and an informant interview *subjects with dementia underwent blood tests and brain imaging | | **Analysis:**  *Cox proportional hazards regression was used to determine influence of midlife intake of antioxidants and risk of late-life cognitive impairment  **Confounders:**  *age, education, smoking status, alcohol intake, body mass index, physical activity, systolic and diastolic blood pressures, year of birth, total energy intake, cholesterol concentration, history of cardiovascular disease, supplemental vitamin intake, and apolipoprotein E e4 | | **Results:**  *235 participants developed dementia over 30.2 years of follow-up (102 Alzheimer’s disease cases, 38 cases of Alzheimer’s disease with cerebrovascular disease, 44 vascular dementia cases, 51 cases due to other causes)  Vitamin E intake: *compared to the first quartile of vit. E intake, the risk for dementia and Alzheimer’s disease was greater for those in the second quartile of vit. E intake (dementia RR=1.47, [1.01, 2.14] and Alzheimer’s disease RR=1.84, [1.04, 3.25], respectively) *compared to the first quartile of vit. E intake, the risk for Alzheimer’s disease with and without cerebrovascular disease was greater for those in the second and fourth quartiles of vit. E intake (RR=1.92, [1.16, 3.18], RR=1.78, [1.06, 2.98], respectively)   *there was a significant association between high intake of antioxidants and risk for Alzheimer’s disease with and without contributing cerebrovascular disease (RR=1.82, [1.04, 3.21])  **Limitations:**  *high nonparticipation at first follow-up and death rates *the short 24-hour recall period of food intake may be inaccurate in determining average intake of antioxidants  **Source of Funding:**  *National Institutes of Health, the National Institute on Aging, and the National Heart, Lung, and Blood Institute | |
| **Authors:** Lehto SM, Ruusunen A, Tolmunen T, Voutilainen S, Tuomainen T, et al.  **Year:** 2013  **Citation:** J Affect Disord. 2013;150:682–685  **Country of study:** Finland  **Aim of study:** Examine the association between dietary zinc intake and depression  **Study design:** Population-based prospective study  **Quality score: (++, + or -):**  **External validity score: (++, + or -):** | | | | | | | | | | | | | |
| **Number of people**  Not reported  **Demographics** Not reported | | **Number of people** 2682 **Characteristics**  Discharge diagnosis of depressive disorder  Age (years) 54.3(48.5–54.4)  HPL depression scores 2(0–3)  Living alone, n (%) 8(13.3) Length of education (years) 8(6–10) Smoking (cigarette-years) 0(0–287)  Alcohol consumption (g/week) 41.1(7.6–116.1) Energy expenditure (kcal/d) 85.8(32.1–179.6) Regular use of vitamins and trace elements, yes (%)7(11.7) Body mass index (kg/m2) 26.8(25.0–28.5)  Total energy intake (kJ/d) 10,451(8708–12,780) Zinc RDIe met (≥9 mg/d), n (%) 60(100.0) Dietary zinc (energy-adjusted)(mg)14.4(12.7–16.4)  No depression  Age (years) 54.3(48.9–54.5)  HPL depression scores 1(0–2)  Living alone, n (%) 282(12.5)  Length of education (years) 8(6–10)  Smoking (cigarette-years) 0(0–173)  Alcohol consumption (g/week) 30.7(6.2–88.5)  Energy expenditure (kcal/d) 88.2(31.8–193.4)  Regular use of vitamins and trace elements, yes (%) 165(7.3)  Body mass index (kg/m2) 26.4(24.5–28.9)  Total energy intake (kJ/d) 9621 (8122–11,289)  Zinc RDIe met (≥9 mg/d), n (%) 2129 (94.3)  Dietary zinc (energy-adjusted)(mg) 14.3 (12.9–16.0)   Test statistics and P value Age Z=−2.2 and 0.026 HPL depression scores Z=−2.7 and 0.006 Living alone χ2=0.0 and 0.846 Length of education Z=0.0 and 0.968 Smoking Z=−0.5 and 0.597 Alcohol consumption Z=−0.7 and 0.467 Energy expenditure Z=0.0 and 0.993 Regular use of vitamins and trace elements, yes χ2=1.60 and .204 Body mass index Z=−0.7 and 0.476 Total energy intake Z=−2.7 and 0.008 Zinc RDIe met – 0.076c Dietary zinc Z=−0.3 and 0.795  **Location**  Kuopio region of eastern Finland  **Recruitment strategy** Not reported  **Length of follow-up**  20 years  **Response rate and loss to follow-up** Not reported  **Eligible population** Finnish men  **Excluded populations** Individuals who at baseline had elevated depressive symptoms. Data were incomplete for 82 participants | | | | **Relevant exposures** Diet, smoking, physical activity **Time** March 1984 and December 1989 **Measurement of exposure** 4-day food recording during baseline examination  The current number of cigarettes, cigars and pipefuls of tobacco smoked daily and the duration of regular smoking in years were recorded using a self-administered questionnaire  Physical activity was assessed using the 12-month Physical Activity Questionnaire | | **Outcomes** Depressive symptoms  **outcome measurement** depression was defined as having received a hospital  discharge diagnosis of unipolar depressive disorder  **Time**  Not reported | | **Analysis strategy** Cox proportional hazards model  **Confounders** age, baseline depression severity, smoking, alcohol use, physical exercise and the use of dietary supplements | | **Number** 60 (2.7%)  **Effect estimates** receiving diagnosis of depression and energy- adjusted dietary zinc intake RR 1.02, 95%CI 0.96– 1.08, p=0.595  lowest tertile of energy-adjusted zinc intake and a diagnosis of depression RR 1.06, 95%CI 0.59– 1.90, p=0.856  future hospital discharge diagnosis of depression and baseline HPL depressions core RR 1.39, 95% CI 1.15–1.67, p=0.001  **Significant trends** No association was observable between the risk of receiving a hospital discharge diagnosis of depression and the energy- adjusted dietary zinc intake as a continuous measure. no association was observed between belonging to the lowest tertile of energy-adjusted zinc intake and a future hospital discharge diagnosis of depression  **Reported limitations  Author** study comprised exclusively of men. the number of individuals who received a hospital discharge diagnosis of depression was fairly small. May not have detected the cases with milder depression  **Source of funding** The authors conducted this study as part of their work, without external funding | |
| **Authors**: Leosdottir M, Nilsson PM, Nilsson JA, Berglund G **Year:** 2007 **Citation:** Eur J Cardiovasc Prev Rehabil. 2007;14(5):701-706 **Country of study:** Sweden **Aim:** To determine whether total fat intake, saturated, monounsaturated, or polyunsaturated fat intake are risk factor for cardiovascular events **Study design:** Longitudinal **Quality score:** ++ **Applicability:** | | | | | | | | | | | | | |
| **Source population:**  *74,138 individuals, comprising men born between 1923-45 and women born between 1923-50 were recruited from 1991 through 1996 | | **Study population:**  ***28,098** enrolled individuals (60.6% were women) *40% participation rate  *Follow-up:* *Date of study entry until occurrence of cardiovascular event or end of follow in year 2002  *mean follow-up of 8.4 years **Exclusion:**  *individuals with history of acute coronary event or ischemic stroke (women: n=168, men: n=536)  **Attrition:**  *100 women and 64 men lost to follow-up mainly due to out-of-country emigration *mortality higher in non-participants | | | | **Exposures:**  *dietary intake was assessed through 7-day menu diary, questionnaire assessing meal patterns, food item consumption frequencies and portion sizes, as well as interviews *intake of fat, protein, carbohydrates, and alcohol recorded in grams/day, and relative fat intake measured as percentage of non-alcohol energy from fat *reproducibility and validity of dietary measurement methods established *intake divided into quartiles of total fat intake, saturated, monounsaturated, and polyunsaturated fat intake; the ratio between unsaturated and saturated fat intake was also obtained | | **Outcomes:**  *fatal and nonfatal CVD event cases identified through local and national registries | | **Analysis:**  *Cox proportional hazards regression used to assess influence of fat intake on risk for cardiovascular events in total, as well as risk for acute coronary events or ischemic stroke   **Confounders:**  *age, smoking habits, alcohol consumption, socioeconomic status, marital status, physical activity, body mass index, fibre intake, and blood pressure; adjustments were also made for total fat intake for the ratio between unsaturated and saturated fats | | **Results:**  *1556 fatal and non-fatal events (908 acute coronary events and 648 ischemic strokes) occurred   *cardiovascular events:* *compared to the first quartile of monounsaturated/sat. fat intake ratio, the risk for cardiovascular events was lower for women in the second quartile of monounsaturated/sat. fat intake ratio (HR=0.77, [0.61, 0.98]); similar findings were observed among women for polyunsaturated/sat. fat intake ratio (HR=0.77, [0.61, 0.97]) *compared to the first quartile of polyunsaturated/sat. fat intake ratio, the risk for cardiovascular events was lower for women in the third quartile of polyunsaturated/sat. fat intake ratio (HR=0.78, [0.62, 0.99]);  *acute coronary events:*  *compared to the first quartile of monounsaturated/sat. fat intake ratio, the risk for acute coronary events was lower for women in the second quartile of monounsaturated/sat. fat intake ratio (HR=0.71, [0.51, 0.99]); similar findings were observed among women for saturated fat intake (HR=0.67, [0.45, 0.99]  **Limitations:**  *residual confounding (e.g., trans-fatty acids not measured) *assessment of diet at only one point in time raises issue of reliability of dietary assessment  *self-reported dietary assessment may lead to underreporting of energy and fat intake *low participation rate *possible healthy-cohort effect  **Source of Funding:**  *The Swedish Scientific Council, The Swedish Cancer Foundation, Anna Jonssons Memorial Fund, the Swedish Heart and Lung Foundation, The European Commission, and The Region of Skane, Sweden | |
| **Authors**: [Lim SH](http://www.ncbi.nlm.nih.gov/pubmed?term=Lim%20SH%5BAuthor%5D&cauthor=true&cauthor_uid=22170337), [Tai BC](http://www.ncbi.nlm.nih.gov/pubmed?term=Tai%20BC%5BAuthor%5D&cauthor=true&cauthor_uid=22170337), [Yuan JM](http://www.ncbi.nlm.nih.gov/pubmed?term=Yuan%20JM%5BAuthor%5D&cauthor=true&cauthor_uid=22170337), [Yu MC](http://www.ncbi.nlm.nih.gov/pubmed?term=Yu%20MC%5BAuthor%5D&cauthor=true&cauthor_uid=22170337), [Koh WP](http://www.ncbi.nlm.nih.gov/pubmed?term=Koh%20WP%5BAuthor%5D&cauthor=true&cauthor_uid=22170337) **Year:** 2013 **Citation:** [Tob Control.](http://www.ncbi.nlm.nih.gov/pubmed/22170337) 2013;22(4):235-240  **Country of study:** Singapore **Aim:** To assess influence of smoking (cessation) on all-cause and cause-specific mortality **Study design:** Longitudinal **Quality score: -**  **Applicability: +** | | | | | | | | | | | | | |
| **Source population:**  *cohort of Hokkiens and Cantonese permanent residents or citizens of Singapore ages 45-74 years recruited between April 1993 and Dec. 1998 (baseline)  *response rate: 85%  *surviving members re-interviewed at follow-up between 1999 and 2004 | | **Study population:**  *63,257 people from Fujian Province and Guangdong Province interviewed at baseline, and 52,322 participants re-contacted at follow-up  *analysis restricted to **48,251** people  *Follow-up:*  *1993-98 to 09  *date of the follow-up interview to the date of death or Dec. 31, 2009, whichever came first  **Exclusion:**  *participants who started smoking after baseline interview (n=2564) and those whose responses were inconsistent (n=1507)  **Attrition:**  *loss to follow-up due to death, physical disability, or participants could no longer be contacted  *41 lost to follow-up due to migration or other reasons | | | | **Exposures:**  *self-reported smoking categorized as:  current smokers (smoking at baseline and f/u interview), long-term quitters (quitter at baseline and f/u interview), new quitters (baseline smoking and quitter at f/u interview), never smokers | | **Outcomes:**  *mortality: all-cause, lung cancer, other cancers, coronary heart disease, stroke, chronic obstructive pulmonary disease identified using nationwide death registry  *mortality data available through Dec. 31 2009 | | **Analysis:**  *Cox proportional hazards model used to assess influence of smoking (cessation) on all-cause mortality  *the association between smoking and cause-specific mortality assessed using competing risks regression  **Confounders:**  *age, year, BMI, sex, dialect, education, alcohol intake, diagnosis of physical activity, hypertension, diabetes mellitus, stroke and cardiovascular  disease, and cancer | | **Results:**  *compared with current smokers, the risk for total  mortality was lower for new quitters (HR=0.84, [0.76, 0.94], long-term quitters (HR=0.61, [0.56, 0.67]), long-term quitters and never-smokers (HR=0.49, [0.46, 0.53]  *compared with current smokers, the risk of lung cancer mortality was lower for new quitters (HR=0.76 [0.57, 1.00) and long-term quitters (HR=0.44, [0.35, 0.57])  *compared with current smokers, the risk for coronary heart  disease mortality was lower for long-term quitters (HR= 0.63, [0.52,0.77])  **Limitations:**  *****nondifferential  misclassification of smoking status  *small sample sizes for various sub-groups  *no validation of self-reported smoking  **Source of funding:**  *National Institutes of Health | |
| **Authors**: Liu S, Willett WC, Manson JE, Hu FB, Rosner B, et al. **Year:** 2003 **Citation:** Am J Clin Nutr. 2003;78(5):920-927 **Country of study:** US **Aim:** To determine the influence of intake of whole grains on body weight and weight changes **Study design:** Longitudinal **Quality score:** + **Applicability:** | | | | | | | | | | | | | |
| **Source population:**  *121,700 US female registered nurses ages 30-55 years recruited in 1976 and followed-up every two years as part of the Nurses’ Health Study | | **Study population:**  ***74,091** women that were ages 38-63 years in 1984 **Exclusion:**  *participants with daily energy intakes outside the 2514 – 14665 KF range in 1980 *respondents with diabetes (n=2518), cardiovascular disease (n=690), and cancer (n=4458)  **Attrition:**  - | | | | **Exposures:**  *semiquantitative food frequency questionnaire (FFQ) administered in 1984, 1986, 1990, and 1994 to measure *intake of whole-grain and refined-grain foods*  *participants were asked about the mean frequency of consumption of one unit of each food item of interest over the past year, with response options ranging from ‘never’ to ‘>=6 times per day’  *average grain intake in servings/day was calculated for each participant *reproducibility and validity of FFQ has been established; when FFQ was compared to diet records, the correlation coefficients for grain products ranged from 0.71-0.77  *in the analysis, grain intake was divided into quintiles; the first quintile represented the smallest change in intake | | **Outcomes:**  **body weight, BMI, and weight changes* assessed*body weight was self-reported every 2 years from 1984 to 1996, height was reported in 1976 *weight change was calculated as the difference in weight or BMI between: 1) 1984 and 1986, 2) 1986 and 1990, 3) and 1990 and 1994; weight change between 1984 and 1996 was also calculated *when self-reported and measured weights were compared in a sample of participants, correlation was 0.96 | | **Analysis:**  *generalized estimating equations used to determine the influence of changes in intake of whole or refined grains on weight changes  **Confounders:**  *age, changes in exercise, change in smoking status, change in hormone replacement therapy status, changes in intakes of alcohol, caffeine, and total energy, changes in intakes of saturated fat, polyunsaturated fat, monounsaturated fat, trans fat, and protein, and BMI at baseline | | **Results:**  *average BMI increased over time regardless of grain intake; 6400 women became obese (BMI >=30) and 657 had a major weight gain (>=25 kg) over 12 years  *average changes in BMI or in weight in 2-4 years during follow-up:* *the average change in *BMI* increased (meaning, greater weight gain) with increasing intake of refined-grains (linear trend test p-value <0.0001)  *the average change in *BMI* decreased (less weight gain) with increasing intake of dietary fiber (trend p-value: 0.0001)  *the average change in *BMI* appeared to decrease (less weight gain) with increasing intake of whole grains (trend test p<0.0001) *same results as above were reported when the average change in *weight* in 2-4 years was the outcome  *average weight gain during follow-up:* *average weight gain became greater with increasing change in intake of refined grains (trend test p-value <0.0001), while it became lower with increasing intake of dietary fiber (trend test p-value <0.0001)  *Odds for obesity or weight gain during follow-up:* *there appeared to be a decreasing trend in obesity (BMI >=30) and major weight gain (>=25 kg) with larger intakes of whole grains (trend test p-values=0.0002 and 0.03, respectively); intake of refined grains appeared to be inversely related to obesity and major weight gain (trend test p-values: <0.0001 and 0.04, respectively)  *there appeared to be a decreasing trend in major weight gain with larger intake of dietary fiber (trend test p-value <0.0001)  *women with the highest increase in intake of dietary fiber had a significantly lower risk of major weight gain compared to women with the smallest change in intake of fiber (OR=0.51, [0.39, 0.67])  **Limitations:**  *those with recent weight gain may attempt to lose weight by increasing consumption of grain products (difficult to ascertain whether increases in intake of grains prevented weight gain) *participants with greater intake of whole grains and caloric restriction may be more health conscious *misclassification of dietary intake may be dependent on body weight (e.g., obese people may underreport intake) *residual confounding from other dietary factors  **Source of Funding:**  *none reported | |
| **Authors**: Liu Y, Sobue T, Otani T, Tsugane S **Year:** 2004 **Citation:** Cancer Causes Control. 2004;15(4):349-357 **Country of study:** Japan **Aim:** To determine the influence of fruit and vegetables on lung cancer **Study design:** Longitudinal **Quality score:** + **Applicability:** | | | | | | | | | | | | | |
| **Source population:**  *two population-based cohorts of 57,591 men and 59,103 women were established through residence registries maintained by local governments and public health centres of administrative districts *cohort I comprised inhabitants ages 40-59 years in 1990 and cohort II consisted of inhabitants ages 40-69 years in 1993 *questionnaires completed by 45,452 men (response rate: 79%) and 49,924 women (response rate: 84%) from both cohorts | | **Study population:**  ***44,774** men and **48,566** women   *Follow-up:* *study start date (Jan. 1, 1990 for Cohort I and Jan. 1, 1993 for Cohort II) until date of diagnosis of lung cancer, date of migration out of study area, date of death or end of follow-up Dec. 31, 1999  **Exclusion:**  *men (n=680) and women (n=1358) with a history of cancer  **Attrition:**  - | | | | **Exposures:**  *dietary habits were assessed in 1990 for Cohort I and 1993 for Cohort II using self-administered questionnaire  *food frequency questionnaires administered to both cohorts that assessed average consumption of food items (vegetables, fruit, fruit and vegetable juices) during past month, with the following response options ‘<1 day/week’, ‘1-2 days/week’, ‘3-4 days/week’, and ‘almost daily’ (with the additional category of ‘never use’ for cohort II) *participants reporting daily drinking of juice, the cups/day were further assessed  *the portion size and content of food items were taken into account when calculating amount of vegetable and fruit intakes, which was subsequently divided into and analysed as tertiles (‘low’, ‘medium’, ‘high’) *when the questionnaire for Cohort I was compared to dietary records, correlation coefficients ranged from 0.26 to 0.52 | | **Outcomes:**  *incident cases of lung cancer (adenocarcinoma, squamous cell carcinoma, small cell carcinoma, large cell carcinoma, other histological types) identified through hospital records, population-based cancer registries, and death certificates | | **Analysis:**  *Cox proportional hazards model used to assess influence of dietary intake on lung cancer risk *relative risks of Cohorts I and II were also pooled using inverse-variance weighting (cohorts combined)  **Confounders:**  *age, gender, area, sports, frequency of alcohol intake, body mass index, vitamin supplement use, salted fish and meat, and pickled vegetables, smoking status, smoking duration, and number of cigarettes per day among ever smokers | | **Results:**  *428 lung cancer cases (177 cases from Cohort I reported during 401,382 person-years of follow-up, and 251 cases from Cohort II during 330,588 person-years)  *among Cohort I participants, the risk of adenocarcinoma was greater for those in the middle consumption category of fruit intake compared to the low consumption category of fruit intake (RR=2.06, [1.20, 3.54]) *overall, there were no significant associations between vegetable and fruit consumption and incidence of lung cancer (except for the above result)  **Limitations:**  *residual confounding *misclassification of intake of fruit and vegetables through use of a simple food frequency questionnaire  **Source of Funding:**  *Ministry of Health and Welfare of Japan | |
| **Authors:** Malmberg JJ, Miilunpalo SI, Vuori IM, Pasanen ME, Oja P, et al.  **Year:** 2006  **Citation:** J Ag Phy Act 14 133-163  **Country of study:** Finland  **Aim of study:** Associations of the ammount, frequency and intesity, and type of leisure-time physical acitivity with the risk of self-reported difficulty in walking and stair climbing  **Study design:** Population-based cohort  **Quality score: (++, + or -):**  **External validity score: (++, + or -):** | | | | | | | | | | | | | |
| **Number of people**  6,787  **Demographics** Not reported | | **Number of people** 1791 **Characteristics**  Men Age in 1981, years 40-47 98 (234/238)  48-55 92 (147/160)  56-64 80(59/74)  Marital status Married 94 (369/394)  Single 92 (55/60)  Divorced, separated, or widowed 88 (14/16)  Living community Urban 95 (253/267)  Rural 91 (186/204)  Education Higher education 100 (21/21)  Secondary education 98 (119/121)  Vocational training 94 (176/187)  No education 86 (110/128)  BMI, kg/m2 <25.0 93 (175/188)  25.0-29.9 94 (219/233) >30 89(33/37)  Smoking status Never smoked 96 (150/157) Past smoker 91 (179/196)  Current smoker 93 (102/110) Alcohol use. g/cby 0 88 (67/76). 0.1-12.0 95 (250/263)  >12.0 92 (122/132)  Participant in working life Yes 95 (404/425)  No 73 (30/41)  Occupational activity Sitting 95 (112/118)  Light or moderate movement 97 (176/182) Heavy or very heavy movement 93 (116/125) Did not work 68 (19/28)  Disease or symptoms Did not prevent participation in PA 96 (335/348)  Somewhat prevented participation in PA 85 (105/124)   Women Age in 1981, years 40-47 93 (252/270) 48-55 91 (177/194) 56-64 77(76/99) Marital status Married 90 (392/434) Single 90(44/49) Divorced, separated, or widowed 87(67/77) Living community Urban 90 (310/345) Rural 90(195/217) Education Higher education 97 (33/34) Secondary education 93 (124/134) Vocational training 93 (185/198) No education 83 (157/189) BMI, kg/m2 <25.0 94 (244/261) 25.0-29.9 89(199/223) >30 73 (43/59) Smoking status Never smoked 89 (388/434) Past smoker 94(43/46) Current smoker 90(71/79) Alcohol use. g/cby 0 86 (242/282) 0.1-12.0 94 (246/262) >12.0 100 (15/15) Participant in working life Yes 91 (357/391) No 86(146/169) Occupational activity Sitting 91 (122/134) Light or moderate movement 92 (267/289) Heavy or very heavy movement 85 (44/52) Did not work 80 (48/60) Disease or symptoms Did not prevent participation in PA 93 (396/428) Somewhat prevented participation in PA 81 (109/135)  **Location**  Oja, Miilunpalo, Vuori, Pasanen, Urponen. Finland  **Recruitment strategy** Not reported  **Length of follow-up**  16 years  **Response rate and loss to follow-up** 77.5%  **Eligible population** all respondents who were 40-64 years old (in 1981) and had no self-reponed difficulty in walking (n=1,198) or stair climbing (n=1,000).  **Excluded populations** excluded all individuals who reponed (in 1980) that they were substantially or totally unable to participate in physical activity because of their health status or who failed to respond to 5 or more of the 13 questions concerning physical activity | | | | **Relevant exposures** Leisure-Time Physical Activity,  **Time** 1980 - 1981 **Measurement of exposure** three different sets of L TPA questions, two sets in 1980 and one in 1981  used two LTPA indexes-fitness activity and commuting-to test recent evidence suggesting that energy expenditure during physical activity  The single-item self-assessment of global LTPA covered the intensity, frequency, and duration of respondents' exercise sessions as follows: .. Which of the following categories best describes your physical activity during the past 2 months?  the single-item self-assessment of global LTPA reflects the behaviour, and LTPA energy-expenditure index, the energy cost of this behaviour | | **Outcomes** Mobility Difficulties  **outcome measurement** self-reported estimate of their ability to walk 2 km and climb several flights of stairs without rest  **Time**  1981, 1990, and 1996 | | **Analysis strategy** Cox proportional hazards model  C**onfounders** age, body-mass Index, disease or symptoms, and education, marital, living community, employment, occupational activity, smoking, and alcohol- consumption status | | **Number** Depending upon outcome and variable:  Walking Men 113-118 Women 130-146  Stair Men 116-127 Women 176-198  **Effect estimates** Relative Risks (95% CI) for Self-Reported Difficulty In Walking and Stair Climbing According to LTPA  Men Difficulty In Walking  Global LPTA  High 1.00 Moderate 1.49 (0.77-2.88) Low 1.60 (0.88-2.91) p = .298  LTPA energy expenditure index  High 1.00 Moderate 1.27 0.77-2.09 Low 0.98 (0.585-1.75) P= .531  LTPA frequency intensity Vigorous 1.00 Moderate 1.13 0.63-2.03 Light 0.93 (0.51-1.72) No activity 1.23 (0.67-2.28) P=.826  Fitness activity >3x a week 1.00 Twice 2.90 (1.09-7.70) Once a week 4.48 (1.70-11.79) <1 per week 3.74 (1.47-9.54) No activity 4.15 (1.62-10.63) P= .029  Commuting At least once a week 1.00 No activity 0.86 (0.54-1.37) P= .532  Difficulty in Stair Climbing Global LPTA  High 1.00 Moderate 1.86 (0.99-3.52) Low 2.30 (1.27-4.16) p = .023  LTPA energy expenditure index  High 1.00 Moderate 1.04 (0.66-1.66) Low 1.26 (0.77-2.05) P= .633  LTPA frequency intensity Vigorous 1.00 Moderate 0.88 (0.51-0.51) Light 0.98 (0.56-1.73) No activity 1.42 (0.81-2.49) P= .393  Fitness activity >3x a week 1.00 Twice 0.91 (0.46-1.82) Once a week 1.38 (0.10-2.71) <1 per week 1.34 (0.69-2.62) No activity 1.81 (0.96-3.43) P= .221  Commuting  At least once a week 1.00 No activity 1.08 (0.71-1.65) P= .725   Women Difficulty In Walking  Global LPTA  High 1.00 Moderate 0.69 (0.37-1.29) Low 1.00 (0.61-1 .65) p = .371  LTPA energy expenditure index  High 1.00 Moderate 0.95 (0.61-1.47) Low 1.04 (0.65-1.66) P=.935  LTPA frequency intensity Vigorous 1.00 Moderate 1.01 (0.61-1.66) Light 1.19 (0.73-1.94) No activity 1.01 (0.57-1.76) P= .873  Fitness activity >3x a week 1.00 Twice 0.86 (0.49-1.52) Once a week 0.82 (0.46-1.45) <1 per week 0.68 (0.38-1.21) No activity 0.71 (0.41-1.25) P=.690  Commuting At least once a week 1.00 No activity 0.79 (0.48-1.31) P= .363  Difficulty in Stair Climbing Difficulty In Walking  Global LPTA  High 1.00 Moderate 0.93 (0.56-1.53) Low 1.49 (0.99-2.24) p = 0.34  LTPA energy expenditure index  High 1.00 Moderate 0.92 (0.65-1.30) Low 0.99 (0.64-1.51) P= .869  LTPA frequency intensity Vigorous 1.00 Moderate 0.95 (0.62-1.47) Light 1.81 (1.20-2.73) No activity 1.16 (0.73-1.85) P= .009  Fitness activity >3x a week 1.00 Twice 0.68 (0.42-1.12) Once a week 0.97 (0.60-1.56) <1 per week 0.98 (0.60-1.62) No activity 0.78 (0.48-1.26) P= .439  Commuting At least once a week 1.00 No activity 0.92 (0.64-1.33) P=.659  **Significant trends** Men  Rates for difficulty in walking and stair climbing were highest among the oldest; the obese; those not working; those who suffered from disease or symptoms that prevented them from participating in physical activity.  Women Rates for difficulty in walking and stair climbing were highest among the oldest; the overweight or obese; those who lived in rural communities; who suffered from disease or symptoms that prevented them from participating in physical activity.  **Reported limitations  Reviewer** self-reported estimates  **Source of funding** Finnish Ministry of Education, the Finnish Ministry of Social Affairs and Health, and the Yrjo Jahnsson Foundation | |
| **Authors**: [Mannami T](http://www.ncbi.nlm.nih.gov/pubmed?term=Mannami%20T%5BAuthor%5D&cauthor=true&cauthor_uid=15118170), [Iso H](http://www.ncbi.nlm.nih.gov/pubmed?term=Iso%20H%5BAuthor%5D&cauthor=true&cauthor_uid=15118170), [Baba S](http://www.ncbi.nlm.nih.gov/pubmed?term=Baba%20S%5BAuthor%5D&cauthor=true&cauthor_uid=15118170), [Sasaki S](http://www.ncbi.nlm.nih.gov/pubmed?term=Sasaki%20S%5BAuthor%5D&cauthor=true&cauthor_uid=15118170), [Okada K](http://www.ncbi.nlm.nih.gov/pubmed?term=Okada%20K%5BAuthor%5D&cauthor=true&cauthor_uid=15118170), et al.  **Year:** 2004 **Citation:** [Stroke.](http://www.ncbi.nlm.nih.gov/pubmed/15118170) 2004;35(6):1248-1253  **Country of study:** Japan **Aim:** To assess the influence of smoking on fatal and nonfatal strokes **Study design:** Longitudinal **Quality score:** ++  **Applicability: +** | | | | | | | | | | | | | |
| **Source population:**  *population-based cohort of 27,063 men and 27,435 women ages 40-59, and born between 1930 and 1949, registered in administrative districts and supervised by public health centre areas in Jan. 1, 1990  *cohort from Ninohe, Yokote, Nagano, Ishikawa | | **Study population:**  *self-administered questionnaire (capturing smoking, drinking, diet) returned by 20,665 men (76%) and 22,484 women (82%) in 1990/1992  *analysis restricted to **19,782** men and **21,500** women  *Follow-up:*  *11 years from 1990 to 2001  *person-months calculated from date of return of the  baseline questionnaire to the first end point, death, or January 1, 2002, whichever was first  **Exclusion:**  *individuals with stroke, myocardial infarction, angina pectoris, cancer at baseline (n=667 men and 883 women)  *those with incomplete data (n=196 men and 85 women)  *loss to follow-up (n=14 men and 14 women)  **Attrition:**  *14 men and 14 women lost to follow-up | | | | **Exposures:**  *self-reported smoking  for both sexes categorized asnever-smokers, ex-smokers, and current smokers; smoking habit for men categorized as: never-smoker, ex-smoker, current smoker, and number of cigarettes 1 to 19/d, 20 to 39/d, and >=40/d | | **Outcomes:**  *fatal and nonfatal strokes assessed with medical records, death certificates | | **Analysis:**  *the Cox proportional hazards model was used to assess the influence of smoking on fatal and nonfatal strokes  **Confounders:**  *****age, alcohol intake, body mass index, history of diabetes (yes), education level, sports at leisure, frequency of fruit, vegetable, and fish servings , and public health centers | | **Results:**  *risks for current smokers compared with never-smokers were higher for total stroke (RR=1.27, [1.05, 1.54]), subarachnoid hemorrhage (RR=3.60, [1.62, 8.01]), ischemic stroke (1.66, [1.25, 2.20]); the respective relative risks among women were 1.98 (1.42 to 2.77), 1.53 (0.86 to 4.25), 2.70 (1.45 to 5.02),  and 1.57 (0.86 to 2.87)  **Limitations:**  *****self-reported data  *limited generalizability of results to women and older men  **Source of funding:**  *****Ministry of Health, Labor, and Welfare of Japan | |
| **Authors:** Masaki M, Sugimori H, Nakamura KI, Tadera M  **Year:** 2003  **Citation:** Asian Pacific J Cancer Prev. 2003;4:61-66  **Country of study:** Japan  **Aim of study:** Identify dietary patterns that may change stomach cancer risk  **Study design:** Cohort  **Quality score: (++, + or -):**  **External validity score: (++, + or -):** | | | | | | | | | | | | | |
| **Number of people**  200,000  **Demographics** | | **Number of people** 5,644 **Characteristics**  Vegetable and fruit  Low  Entire cohort (%) 33.4 Mean age (years) 50.1 Mean BMI 23.1 Education (% university) 58.9 History of peptic ulcer (% yes) 17.9 Family history of stomach cancer (% yes) 10.3 Cigarette smoking (%)  Never 18.8 Past 21.2 Current 60.1 Alcohol drinking (%) No 17.2 Light 32.4  Heavy 50.4   Middle  Entire cohort (%) 34.5  Mean age (years) 51.7 Mean BMI 23.2 Education (% university) 62.3 History of peptic ulcer (% yes) 20.1 Family history of stomach cancer (% yes) 8.3 Cigarette smoking (%)  Never 21.1 Past 29.3 Current 49.7 Alcohol drinking (%) No 18.3 Light 36.3  Heavy 45.4  High Entire cohort (%) 32.1  Mean age (years) 53.2  Mean BMI 23.2 Education (% university) 64.4  History of peptic ulcer (% yes) 22.3 Family history of stomach cancer (% yes) 10.9 Cigarette smoking (%)  Never 21.1 Past 34.3 Current 44.6  Alcohol drinking (%) No 19.6 Light 38.3 Heavy 42.1   Western breakfast  Low  Entire cohort (%) 32.6  Mean age (years) 51.9 Mean BMI 23.4 Education (% university) 54.6 History of peptic ulcer (% yes) 20.5 Family history of stomach cancer (% yes) 10.7 Cigarette smoking (%)  Never 17.8 Past 28.6 Current 53.7 Alcohol drinking (%) No 12.1 Light 27.9 Heavy 60.0  Middle  Entire cohort (%) 32.9 Mean age (years) 50.8 Mean BMI 23.2 Education (% university) 62.0 History of peptic ulcer (% yes) 18.0 Family history of stomach cancer (% yes) 8.6 Cigarette smoking (%)  Never 21.6 Past 26.0 Current 52.4 Alcohol drinking (%) No 18.9 Light 34.7 Heavy 46.4  High Entire cohort (%) 34.5  Mean age (years) 52.0 Mean BMI 23.0 Education (% university) 68.5 History of peptic ulcer (% yes) 21.6 Family history of stomach cancer (% yes) 10.0 Cigarette smoking (%)  Never 21.6 Past 29.9 Current 48.6 Alcohol drinking (%) No 23.7 Light 43.9  Heavy 32.5  Meat  Low  Entire cohort (%) 33.7 Mean age (years) 53.4 Mean BMI 23.2 Education (% university) 63.9 History of peptic ulcer (% yes) 21.5 Family history of stomach cancer (% yes) 10.3 Cigarette smoking (%)  Never 22.4 Past 33.8 Current 43.8 Alcohol drinking (%) No 23.2 Light 38.8  Heavy 38.0  Middle  Entire cohort (%) 35.5 Mean age (years) 51.1 Mean BMI 23.2 Education (% university) 60.0 History of peptic ulcer (% yes) 20.3 Family history of stomach cancer (% yes) 8.9 Cigarette smoking (%)  Never 19.2 Past 26.8 Current 54.0 Alcohol drinking (%) No 15.9 Light 37.0  Heavy 47.1  High Entire cohort (%) 30.8 Mean age (years) 50.1 Mean BMI 23.2 Education (% university) 61.7 History of peptic ulcer (% yes) 18.2 Family history of stomach cancer (% yes) 10.2 Cigarette smoking (%)  Never 19.4 Past 23.6 Current 57.1 Alcohol drinking (%) No 15.8 Light 30.6  Heavy 53.6  Rice/snacks Low Entire cohort (%) 33.6  Mean age (years) 51.4 Mean BMI 23.1 Education (% university) 60.3 History of peptic ulcer (% yes) 21.4 Family history of stomach cancer (% yes) 10.0 Cigarette smoking (%)  Never 16.0 Past 27.5 Current 56.4 Alcohol drinking (%) No 13.2 Light 33.1  Heavy 53.8  Middle  Entire cohort (%) 32.9 Mean age (years) 51.1 Mean BMI 23.2 Education (% university) 63.0 History of peptic ulcer (% yes) 18.8 Family history of stomach cancer (% yes) 9.4 Cigarette smoking (%)  Never 18.9 Past 27.4 Current 53.7 Alcohol drinking (%) No 15.8 Light 36.5 Heavy 47.7  High Entire cohort (%) 33.5 Mean age (years) 52.4 Mean BMI 23.2 Education (% university) 62.4 History of peptic ulcer (% yes) 20.0 Family history of stomach cancer (% yes) 10.0 Cigarette smoking (%)  Never 26.1 Past 29.6 Current 44.4 Alcohol drinking (%) No 26.0 Light 37.4 Heavy 36.6  **Location**  Tokoyo, Japan  **Recruitment strategy** Not reported  **Length of follow-up**  10 years of follow-up  **Response rate and loss to follow-up** subjects who had retired from their firms before the start of the study (n=8), having a past history of cancer (n=11), incomplete description of food consumption questionnaire (n=102), those identified within first year follow-up (n=71)  **Eligible population** members of the Health Insurance Society of Tokyo Stockbrokerage  **Excluded populations** | | | | **Relevant exposures** frequency of consumption of selected foods, health condition, medical history, smoking and drinking habits, exercise and leisure time, places of birth, and working situation **Time** 1988 **Measurement of exposure** Self-report questionnaire | | **Outcomes** stomach cancer  **outcome measurement** detailed statements of medical care (performed for insured persons) by medical care facilities  **Time**  until the dates of the events or the end of follow-up (August 31, 1998) whichever occurred first | | **Analysis strategy** Cox proportional hazard regression model  **Confounders** age (10 years age groups), cigarette smoking (never, former current), alcohol drinking (no or current drinker), and history of peptic ulcer and family history of stomach cancer (yes or no). | | **Number** 86 incident cases of stomach cancer  **Effect estimates** Rate Ratios for Stomach Cancer According to Tertiles of Dietary Pattern RR 95% CI Vegetable and fruit  Low 1.00  Middle 1.06 (0.61-1.87)  High 0.78 (0.42-1.44) P trend .56  Western breakfast  Low 1.00  Middle 0.59 (0.33-1.08) High 0.71 (0.40-1.24) P trend .20  Meat  Low 1.00  Middle 0.55 (0.29-1.01) High 1.10 (0.64-1.89) P trend .07   Rice/snacks Low 1.00  Middle 0.52 (0.27-1.01) High 1.19 (0.71-2.02) P trend .05  **Significant trends** There were no clear associations between the four major dietary patterns and stomach cancer risk  **Reported limitations  Author** relatively small size of cohort. food consumption data in summer only. case ascertainment only through medical records provided by various clinical sites. no data on histological classification of stomach cancer. portion size and energy intake were not available.   **Source of funding** Grant-in-Aid for Scientific Research from the Ministry of Education, Science, Sports and Culture of Japan: 61010076, 62010074, 63010074, 1010068, 2151065, 3151064, 4151063, 5151069, 6279102 | |
| **Authors:** Menotti A, Lanti M, Puddu PE  **Year:** 2000  **Citation:** Ital Heart J. 2000; I(II):749-757  **Country of study:** Italy  **Aim of study:** To describe the comprehensive disease burden related to cardiovascular diseases of atherosclerotic-hypertensive origin in a population sample of middle-aged men  **Study design:** Prospective cohort  **Quality score: (++, + or -):**  **External validity score: (++, + or -):** | | | | | | | | | | | | | |
| **Number of people**  Not reported  **Demographics** Not reported | | **Number of people** 1712 **Characteristics**  Age (years) 49.8 ± 5.1 Cigarettes (n/day) 8.7 ± 9.5 Diabetes(%) 4.8 Corneal arcus(%) 13.9 Vital capacity (dl) 45.7 ± 8.0 Cholesterol (mmol/1) 5.21 ± 1.06 Systolic blood pressure (mmHg) 143.6 ± 21.O  **Location**  Crevalcore in Northern Italy and Montegiorgio in Central Italy  **Recruitment strategy** Not reported  **Length of follow-up**  2S-year follow-up  **Response rate and loss to follow-up** 98.8%  **Eligible population** Italian middle-aged men  **Excluded populations** subjects with cardiovascular diseases at entry examination | | | | **Relevant exposures** Smoking habits **Time** 1960 **Measurement of exposure** elicited from a questionnaire allowing us to estimate the daily average consumption of cigarettes (nlday) | | **Outcomes** morbidity and mortality  **outcome measurement** Causes of death were allocated reviewing and combining together information from several sources such as death cenificlnes, hospital and medical records, interviews with physicians, relatives of the deceased and any other witness of the fatal event.  morbidity data obtained via a) interim quinquennial; b) information obtained in relation to causes of death; c) periodic visits to local physicians and hospitals for identification of new cases; d) home visits to subjects suspected of having developed a new CV event; e) postal questionnaire and postal clinical records in a few cases  **Time**  Not reported | | **Analysis strategy** log-linear model incorporating the Weibull distribution  **Confounders** Age, Cigarettes, diabetes, corneal arcus, vital capacity, cholesterol and systolic blood pressure | | **Number** 126 for CHD, 62 for stroke, 8 for PAD, 9 for other heart diseases, 24 for lung cancer, 126 for other cancer locations, 21 for chronic bronchitis, 8 for infectious diseases, 25 for violence, and 75 for all other causes  **Effect estimates** CHD-H  Coefficient T value Age -0.0308 -3.69  Cigarettes -0.0101 -2.60   CHD-A  Coefficient T value Age -0.0281 -3.63  Cigarettes -0.0070 -1.89   STR-H  Coefficient T value Age -0.0471 -5.17  Cigarettes -0.0038 -0.89   STR-A  Coefficient T value Age -0.0471 -.5.55  Cigarettes 0.0041 -1.02   PAD  Coefficient T value Age -0.0450 -3.27  Cigarettes 0.0276 -4.45  CVD Coefficient T value Age -0.0401 -6.42 Cigarettes -0.0115 -3.95  Delta for Hazard ratio, Hazard ratio, (95% Cl) Age (years) 5, 1.29, (1.19-1.39) Cigarettes (n/day) 10, 1.16 (1.08-1.24)  **Significant trends** Incidence of cardiovascular disease is higher than CHD  **Reported limitations  Author** Not reported  **Source of funding** personal grant to the senior author (AM) from the Association for Cardiac Research. Rome, Italy, and by a grant of the Martinson Clinical Foundation, Wayzata. Minnesota, USA. | |
| **Authors:** Menotti A, Lanti M, Maiani G, Kromhout D  **Year:** 2006  **Citation:** Aging Clin Exp Res. 2006;18(5):394-406  **Country of study:** Italy  **Aim of study:** Survival and allcause mortality in two cohorts of middle-aged men followed for 40 years  **Study design:**  **Quality score: (++, + or -):**  **External validity score: (++, + or -):** | | | | | | | | | | | | | |
| **Number of people**  Not reported  **Demographics** Not reported | | **Number of people** 1712 **Characteristics**  Not reported  **Location**  Crevalcore in Northern ltaly and Montegiorgio in Central Italy  **Recruitment strategy** Not reported  **Length of follow-up**  40 years  **Response rate and loss to follow-up** 98.8%   **Eligible population** Middle-aged men  **Excluded populations** Not reported | | | | **Relevant exposures** physical activity, cigarette smoking, diet **Time** 1960 **Measurement of exposure** questionnaire | | **Outcomes** Causes of death and morbidity  **outcome measurement** death certificates, hospital and medical records, interviews with physicians and relatives of the deceased, and any other witnesses of fatal events.  **Time**  Not reported | | **Analysis strategy** proportional hazard model  **Confounders** age; father history, mother history, family history of cardiovascular diseases; marital status children; job related physical activity, cigarette smoking, diet of any type followed by subjects; height, weight, body mass index (BMI), trunk/height ratio, biacromial diameter, bicrystal diameter, shoulder/pelvis shape. laterality/linearity index, tricipital skinfold thickness. subscapular skinfold thickness, arm circumference; systolic blood pressure. diastolic blood pressure, rnean blood pressure, heart rate: vital capacity, forced expiratory volume; serum choles· terol, urine protein. urine glucose: baldness. corneal arcus. xanthelasma: diagnoses of Cardiovascular diseases, cancer. diabetes, chronic bronchitis; history of lung tuberculosis, bronchial asthma, peptic ulcer, intestinal diseases. liver diseases, gall bladder diseases. kidney stones. other genito-urinary diseases; thyroid disease; minor ECG abnormalities at rest. exercise ECG abnormalities | | **Number** 1434 deaths (83.8%)  **Effect estimates** Risk factor Coefficient and (SE) Hazard ratio (95% confidence) Standardized Coefficient and (rank) Age 0.1021 (0.0064) 1.67 (1 56-1.77) 0.5152 (1) Father history.0.1692 (0.0679) 1.18 (1.04-135) 0.0689 (10) Mother history 0.2166 (0.0695) 1.24 (1.08-1.42) 0.0875 (7) Physical activity -0.0986 (0.0446) 0.91 (0.83-0.09) -0.0636 (13) Cigarette smoking 0.0194 (0.0029) 1.21 (1.15-1.29) 0.1841 (3) Body mass index (linear) -0.1425 (0.0721) 0.57 (0.33-1.00) --- Body mass index (quadratic) 0.0025 (0.0013) 1.65 (0.99-2.74) --- Mid-arm circumference -0.0036 (0.0017) 0.91 (0.84-0.99) -0.0840(8} Mean blood pressure 0.0205 (0 0024) 1.36 (1.27-1.46) 0.2764 (2) Forced expiratory volume in 3/4 sec -0.5770 (0.1256) 0 87 (0.81-0.92) -0.1439(4} Serum cholesterol 0.0851 (0.0271) 1.09 (1.03-1.15) 0.0908 (6) Corneal arcus 0.1838 (0.0795) 1.20 (1.03-1.40) 0.0643 (12) Xanthelasma 0 5550 (0.21421) 1.74 (1.14-2.65) 0.0685 (11) Diagnosis of cardiovascular disease 0.3714 (0.1315) 1.45 (1.12-1.88) 0.0761 (9) Diagnosis of cancer 2.1717 (0.4554) 8.77 (3.59-21 42) 0.1232(5) Diagnosis of diabetes 0.2193 (0.1103) 1.25 (1.00-1.55) 0.0549 (14)  **Significant trends** During a 40-year period 15 mainly cardiovascular risk factors were highly predictive of all cause mortality and survival in middle-aged men  **Reported limitations  Author** Not reported  **Source of funding** The company Medrisk of Roma, Italy, contributed financially to this analysis | |
| **Authors**: Menotti A, Alberti-Fidanza A, Fidanza F **Year:** 2012 **Citation:** Nutr Metab Cardiovasc Dis. 2012;22(4):369-375 **Country of study:** Italy **Aim:** To test the adequacy of the Mediterranean Adequacy Index (MAI) as a predictor of CHD mortality **Study design:** Longitudinal **Quality score:** - **Applicability:** | | | | | | | | | | | | | |
| **Source population:**  *men ages 40-59 years of two rural cohorts living in Crevalcore and Montegiorgio, Italy | | **Study population:**  *1712 men (98% of the roster) were examined in 1960; of these, 1286 men were re-examined in 1965 *study restricted to **1139** men free from CHD at study entry and with complete data   *Sociodemographics:* *mean age of 34.5 years at baseline **Exclusion:**  *men who died between 1960 and 1965 (n=90) *men with missing data (n=278) *men with CHD  **Attrition:**  - | | | | **Exposures:**  *the adequacy of the Mediterranean Adequacy Index (MAI), which captures the healthiness of a diet, was tested as a predictor of CHD mortality  *Mediterranean Adequacy Index (MAI)* *in 1960, participant diet was captured through weighted-record method for 1 week during 3 seasons from a subsample of 28 men from Crevalcore and 34 from Montegiorgio *in 1965, food intake of all participants was assessed through dietary-history method *dietary intake data was used to create the MAI: *the MAI measures the similarity of various diets to the Reference Mediterranean Dietary Pattern, and an MAI score ranging from 0 to 100 is obtained by dividing the sum of percentages of dietary energy of healthy Mediterranean diet food groups (e.g., cereals, legumes, vegetables) by the sum of the percentages of dietary energy of other food groups not characteristic of a Mediterranean diet (e.g., milk, cheese, meat, animal fats, cakes, pies, sugar) | | **Outcomes:**  *CHD deaths occurring during 40 years after 1965 examination ascertained through death certificates, medical records, physical interviews, interviews with relatives or other witnesses of fatal events | | **Analysis:**  *reduced rank regression was used to derive dietary patterns with C-reactive protein, IL-6 and IL-18 as responses; partial least squares and principal components regression was also used  **Confounders:**  *age, survey, BMI, place of residence (urban/rural), actual hypertension (yes/no), education level (low/ high), self-reported diagnosis of diabetes, physical activity (active/not active), energy intake (kcal/day), ratio of total cholesterol and HDL cholesterol, smoking status | | **Results:**  *79 CHD fatal events in 20 years, and 162 in 40 years *the coefficient of lnMAI was negative, and thus, had a protective effect against CHD mortality at 20 and 40 years (hazard ratios for 1 unit of lnMAI: HR=0.74, [0.55, 0.99], and HR=0.79, [0.64, 0.97] for each follow-up time period, respectively) *hazard ratio of 1 unit of lnMAI (2.7 units of MAI) was associated with a CHD mortality reduction of 26% and 21% at 20 and 40 years of follow-up, respectively  **Limitations:**  *small sample size  **Source of Funding:**  *none reported | |
| **Authors**: Meyer J, Döring A, Herder C, Roden M, Koenig W, et al.  **Year:** 2011 **Citation:** Eur J Clin Nutr. 2011;65(7):800-807 **Country of study:** Germany **Aim:** To determine the association between dietary patterns, subclinical inflammation, incident coronary heart disease and mortality **Study design:** Longitudinal **Quality score:** + **Applicability:** | | | | | | | | | | | | | |
| **Source population:**  *3 cross-sectional surveys were conducted during 1984-1995 in Southern Germany | | **Study population:**  ***981** males ages 45-64 years with complete data for survey 1 (n=594/899) and survey 3 (n=387/430) conducted in 1984-85 and 1994-95, respectively  *participants were on average 54.9 years at time of exposure assessment  *Follow-up:* 1984-1995 to 2002 for CHD case development or 2007 for CHD mortality *median follow-up time until occurrence of acute coronary event was 16.7 and 7.9 years for CHD survey 1 and survey 3, respectively *median follow-up time until death was 22.8 and 12.9 years for survey 1 and survey 3, respectively **Exclusion:**  -  **Attrition:** - | | | | **Exposures:**  *7-day dietary record completed by participants through surveys *food consumed assessed through weighing techniques and household measures | | **Outcomes:**  *incidence of fatal or non-fatal myocardial infarction and all-cause mortality were study end points *deaths were identified through population registries and the underlying cause of death ascertained through death certificates, while myocardial infarctions occurring before 75 years were identified through population-based coronary event registry and questionnaires *self-reported non-fatal incident myocardial infarction cases were validated using hospital records/physicians  *proinflammatory markers CRP, IL-6 and IL-18 measured through blood samples at the time the surveys were administered | | **Analysis:**  *reduced rank regression was used to derive dietary patterns with C-reactive protein, IL-6 and IL-18 as responses; partial least squares and principal components regression was also used  **Confounders:**  *age, survey, BMI, place of residence (urban/rural), actual hypertension (yes/no), education level (low/ high), self-reported diagnosis of diabetes, physical activity (active/not active), energy intake (kcal/day), ratio of total cholesterol and HDL cholesterol, smoking status | | **Results:**  *101 participants had an acute coronary event, and 292 died (88 died from CHD) during follow-up  *high intakes of meat, soft drinks and beer and low intakes of vegetables, fresh fruit, chocolates, cake, pastries, wholemeal bread, cereals, muesli, curd, condensed milk, cream, butter, nuts, sweet bread spread and tea was associated with a *high score* of the RRR-derived pattern  *a *high score* was associated with a high risk for all-cause mortality (HR=1.16, [1.00, 1.33], respectively)  **Limitations:**  *cannot generalize to women *limited study power *participants may have changed dietary behaviour over time, which may attenuate association between dietary behaviour at baseline and end points  **Source of Funding:**  *Helmholtz Zentrum Munchen, the Federal Ministry of Education and Research, Berlin, German Research Foundation, Bonn, University of Ulm, the German Diabetes Center, Dusseldorf, the Federal Ministry of Health, the Ministry of Innovation, Science, Research and Technology of the state North Rhine Westphalia | |
| **Authors:** Miura K, Greenland P, Stamler J, Liu K, Daviglus ML, et al.  **Year:** 2004  **Citation:** Am J Epidemiol. 2004;159:572–580  **Country of study:** USA  **Aim of study:** Associations between food group intake and subsequent blood pressure change  **Study design:** Prospective population study  **Quality score: (++, + or -):**  **External validity score: (++, + or -):** | | | | | | | | | | | | | |
| **Number of people**  5,397  **Demographics** Not reported | | **Number of people** 2,080 **Characteristics**  Mean (SD) Age (years) 48.5 (4.4)  Height (cm) 174.6 (6.4)  Weight (kg) 78.1 (11.0)  Body mass index (weight (kg)/height (m)2) 25.6 (3.2)  Obesity (body mass index ≥30) (%) 8.5 Current smoker (%) 53.0 No. of cigarettes per day: all 10.2 (11.0)  No. of cigarettes per day: smokers 17.8 (8.8) Current drinker (%) 85.8 Current alcohol consumption: all (ml/day) 16.2 (20.3)  Current alcohol consumption: drinkers (ml/day) 118.8 (20.7)  Education (no. of years) 11.2 (2.5)  Total energy (kcal) 3,124 (777)  Total fat (% of kcal) 42.8 (4.2)  **Location**  Chicago, Illinois  **Recruitment strategy** Random selection  **Length of follow-up**  7 year  **Response rate and loss to follow-up** 67.1 percent  **Eligible population** men aged 40–55 years. Employed at the Hawthorne Works of the Western Electric Company in Chicago  **Excluded populations** missing baseline dietary assessments (n = 191); blood pressure (n = 72); educational attainment (n = 223); previously diagnosed diabetes mellitus (n = 31); prior myocardial infarction (n = 44), and/or fewer than three follow-up examinations between 1960 and 1966 (n = 184). | | | | **Relevant exposures** Nutrient intake **Time** 1958 - 1959 **Measurment of exposure** two nutritionists using standardized interviews and questionnaires based on Burke’s comprehensive dietary history method | | **Outcomes** systolic blood pressure or diastolic blood pressure  **Outcome measurement** standard mercury sphygmomanometers  **Time**  1966 | | **Analysis strategy** generalized estimating equation models  **Confounders** baseline age, weight at each year, height, education, smoking, alcohol consumption, daily intake of 12 nutrients | | **Number** Not reported  **Effect estimates** *Relation between baseline food intake and adjusted average annual change in men’s systolic blood pressure* Change/ year (mmHg) p value Vegetables (cups/month)  14–42 –0.29 0.096 >42 –0.08 0.801  Fruits (cups/month)  14–42 –0.29 0.043 >42 –0.22 0.307  Fish (120-g units/month)  <4 –0.34 0.095 4–8 –0.22 0.286 >8 –0.41 0.085  Beef-veal-lamb (120-g units/month)  8–20 0.76 0.026 >20 0.80 0.022  Pork (120-g units/month)  4–8 0.55 0.002 >8 0.33 0.060  Poultry (120-g units/month)  4–8 0.21 0.072 >8 0.49 0.012  *Relation between baseline food intake and adjusted average annual change in men’s diastolic blood pressure* Change/ year (mmHg) p value Vegetables (cups/month)  14–42 –0.11 0.269 >42 –0.06 0.713  Fruits (cups/month)  14–42 –0.13 0.115 >42 –0.19 0.119  Fish (120-g units/month)  <4 –0.11 0.282 4–8 –0.03 0.811 >8 –0.17 0.190  Beef-veal-lamb (120-g units/month)  8–20 0.29 0.089 >20 0.41 0.022  Pork (120-g units/month)  4–8 0.06 0.592 >8 0.04 0.735  Poultry (120-g units/month)  4–8 0.18 0.006 >8 0.25 0.031  **Significant trends** Higher intakes of vegetables and of fruits were related to less of an increase in SBP and DBP over time. men with a higher intake of red meat had a significantly greater increase in blood pressure. men with a higher poultry intake had a significantly greater annual increase in blood pressure. men with a higher fish intake tended to have less of an increase in blood pressure.  **Reported limitations  Author** data collection for this study took place over 40 years ago. no information on dietary sodium chloride, potassium, magnesium, or fiber intakes. misclassification of intake for each food. unmeasured nondietary factors. findings may (or may not) be generalizable beyond middle-aged, non-Hispanic White males.   **Reviewer** no control for medication use  **Source of funding** American Heart Association and its Chicago and Illinois affiliates; the National Heart, Lung, and Blood Institute (HL 15174, HL 21010, and HL 03387); the Chicago Health Research Foundation; the Otho S. Sprague Foundation; the Research and Education Committee of the Presbyterian-St. Luke’s Hospital; the Illinois Foundation; and private donors | |
| **Authors:** Moayyeri A, Kaptoge S, Luben RN, Wareham NJ, Bingham S, et al.  **Year:** 2009  **Citation:** Eur J Epidemiol. 2009;24:259–266  **Country of study:** UK  **Aim of study:** Estimates of fracture risk  **Study design:** Population-based cohort study  **Quality score: (++, + or -):**  **External validity score: (++, + or -):** | | | | | | | | | | | | | |
| **Number of people**  25,639  **Demographics** Not reported | | **Number of people** 25,311 **Characteristics**  Women  Fracture n = 649 Age (years) 64.7 (8.4) History of fracture 117 (11.1%) Height (cm) 160.2 (6.3) Weight (kg) 67.4 (12.4) Body mass index (kg/m2) 26.2 (4.5) Current smoking 64 (9.9%) Alcohol intake (units/week) 1.5 (0.5–4.5)  No fracture n = 13,186 Age (years) 58.1 (9.2) History of fracture 936 (7.1%) Height (cm) 161.0 (6.2) Weight (kg) 68.0 (11.8) Body mass index (kg/m2) 26.2 (4.3) Current smoking 1,508 (11.4) Alcohol intake (units/week) 2.5 (0.5–6.5)  P value Age (years) \0.001 History of fracture \0.001 Height (cm) 0.002 Weight (kg) 0.2 Body mass index (kg/m2) 0.9 Current smoking 0.2 Alcohol intake (units/week) \0.001  Men Fracture n = 276 Age (years) 61.9 (9.7) History of fracture 25 (9.1%) Height (cm) 174.4 (6.4) Weight (kg) 80.7 (11.9) Body mass index (kg/m2) 26.5 (3.4) Current smoking 38 (13.8%) Alcohol intake (units/week) 7 (2–16.5)  No fracture n = 11,200 Age (years) 59.0 (9.3) History of fracture 654 (5.8%) Height (cm) 174.0 (6.6) Weight (kg) 80.4 (11.5) Body mass index (kg/m2) 26.5 (3.3) Current smoking 1,362 (12.2%) Alcohol intake (units/week) 6 (2–14)  P value Age (years) \0.001 History of fracture 0.02 Height (cm) 0.3 Weight (kg) 0.6 Body mass index (kg/m2) 0.9 Current smoking 0.4 Alcohol intake (units/week) 0.2  **Location**  Norfolk, East Anglia  **Recruitment strategy** age and sex registers of general practices  **Length of follow-up**  11.3 years (SD = 1.5; range 9.2–14.1  **Response rate and loss to follow-up** Not reported  **Eligible population** Not reported  **Excluded populations** Not reported | | | | **Relevant exposures** Smoking and alcohol **Time** 1993–1997 **Measurement of exposure** Smoking status was derived from responses to the questions ‘‘Have you ever smoked as much as one cigarette a day for as long as a year?’’ and ‘‘Do you smoke cigarettes now?’’   Alcohol consumption derived from a question ‘‘How many alcoholic drinks do you have each week?’’ with four separate categories of drinks.   Total alcohol consumption was estimated as the total units of drinks consumed in a week | | **Outcomes** Osteoporotic fractures  **outcome measurement** death certificates  **Time**  March 2007 | | **Analysis strategy** Cox proportional hazards models  **Confounders** age, sex, history of fractures, body mass index, smoking, and alcohol intake | | **Number** 925 incident fractures; 334 (36%) hip fractures; 154 (17%) clinical spinal fractures; 219 (24%) wrist fractures  **Effect estimates** Any incident fracture HR (95% CI)  Women (649 cases)  Age (years)1.08 (1.07–1.09)  History of fracture 1.92 (1.57–2.36) Body mass index (kg/m2) 0.99 (0.97–1.01)  Smoking status (current) 1.10 (0.85–1.43)  Alcohol intake (units/week) 0.98 (0.97–1.00)  C-index (95% CI)  Derivation dataset 0.70 (0.67–0.72)  Validation dataset 0.72 (0.67–0.76)   Men (276 cases)  Age (years) 1.04 (1.02–1.05)  History of fracture 1.53 (1.01–2.31) Body mass index (kg/m2) 0.99 (0.96–1.03)  Smoking status (current) 1.19 (0.84–1.68)  Alcohol intake (units/week) 1.01 (1.01–1.02)  C-index (95% CI)  Derivation dataset 0.60 (0.55–0.64)  Validation dataset 0.63 (0.56–0.70)   Incident hip fracture HR (95% CI) Women (245 cases)  Age (years) 1.14 (1.12–1.16) History of fracture 1.59 (1.14–2.20) Body mass index (kg/m2) 0.96 (0.93–0.99) Smoking status (current) 1.19 (0.77–1.83) Alcohol intake (units/week) 0.99 (0.97–1.02) C-index (95% CI)  Derivation dataset 0.78 (0.75–0.81) Validation dataset 0.82 (0.78–0.87)  Men (89 cases) Age (years) 1.15 (1.11–1.18) History of fracture 1.73 (0.87–3.45) Body mass index (kg/m2) 0.95 (0.89–1.01) Smoking status (current) 1.38 (0.74–2.56) Alcohol intake (units/week) 1.01 (0.99–1.03) C-index (95% CI)  Derivation dataset 0.79 (0.74–0.85) Validation dataset 0.79 (0.72–0.86)  **Significant trends** Statistically significant differences between men and women. The 10-year probability of fracture was approximately 1% in both men and women aged 40–45 years rising to about 17% for women and 5% for men aged 75 years with a previous history of fracture.  **Reported limitations  Author** lack of BMD assessment at the beginning of follow-up. potential for under-registration of fracture outcomes in the cohort population. participants are likely to be healthier and have lower fracture rates  **Source of funding** EPIC-Norfolk is supported by program grants from the Medical Research Council and Cancer Research UK with additional support from the Stroke Association, Research into Ageing, the Academy of Medical Sciences, British Heart Foundation, Department of Health, and the Wellcome Trust | |
| **Authors**: Morgan GS, Gallacher J, Bayer A, Fish M, Ebrahim S, et al. **Year:** 2012 **Citation:** J Alzheimers Dis. 2012;31(3):569-580 **Country of study:** Wales **Aim:** To determine the association between physical activity in mid-life and dementia in late-life **Study design:** Longitudinal **Quality score:** +  **Applicability:** | | | | | | | | | | | | | |
| **Source population:**  *2,512 mostly manual class men ages 45-59 and resident in this region during 1979-1983 were identified through electoral register and recruited (89% of eligible population) | | **Study population:**  *analysis restricted to **1005** men who took part in phase 2 (1984-1988) and phase 5 (2002-2004) of study and with complete variable data  *Follow-up:* *16 years *between phase 2 and phase 5, 125 people could not be traced  **Exclusion:**  -  **Attrition:**  *85% of subjects participating in phase 2 and 5 included (between these phases: participants had died [n=109], moved [n=86], did not wish to take part [n=295], could not be traced [n=125] or had incomplete exposure/covariate or outcome data) | | | | **Exposures:**  *physical activity self-reported questionnaire data assessed: *work-related physical activity: combined score created that is indicative of occupational time spent sitting, walking, lifting, and the total number of hours spent at work  *leisure-time physical activity: total duration and frequency of participation in over thirty different leisure-time activities assessed over the past year, with each activity allocated an intensity score; finally, type, frequency, and duration of leisure-time physical activity were combined into a composite score for each participant | | **Outcomes:**  *cognitive function screening using CAMCOG  *clinical assessment for case ascertainment  included use of death certificates, community mental health team for older people used for those institutionalized (those clinically assessed underwent further tests and clinical exams) *outcome categories:  1.cognitive impairment not dementia (CIND) [further broken down into vascular vs. non-vascular CIND] 2.dementia [vascular dementia vs. non-vascular dementia] | | **Analysis:**  *logistic regression used to assess whether work-related and leisure-related physical activity in mid-life are related to dementia or cognitive impairment  **Confounders:**  *age, social class, National Adult Reading Test score, smoking status, marital status, self-reported history of vascular disease, alcohol consumption, body mass index, common mental disorder, anxiety | | **Results:**  *no association between leisure-time activity and vascular or non-vascular subtypes *no association between work-related or leisure-related activity with dementia risk  **Limitations:**  *residual confounding, such as social networking and engagement *self-report can lead to non-differential misclassification and underestimation of true associations *possible that study was underpowered *associations may reflect healthy survivor effect  **Source of Funding:**  *Medical Research Council, Alzheimer’s Society | |
| **Authors:** Mursu J, Voutilainen S, Nurmi T, Tuomainen TP, Kurl S, et al.  **Year:** 2008  **Citation:** Br J Nutr. 2008;100:890–895  **Country of study:** Finland  **Aim of study:** Investigate risk factors for CVD, atherosclerosis and related outcomes in middle-aged men from eastern Finland  **Study design:**  **Quality score: (++, + or -):**  **External validity score: (++, + or -):** | | | | | | | | | | | | | |
| **Number of people** 2682 **Demographics** Not reported | | **Number of people** 1950 **Characteristics**  Quartiles of flavonoid intake (mg/d) Mean SD 1 (lowest)  Age (years) 51.9 5.4 BMI (kg/m2) 26.7 3.4 Leisure-time physical activity (kJ/d) 482.8 636.8 Leisure-time physical activity (kcal/d) 115.4 152.2 Smokers (%) 38.2  Alcohol intake (g/week) 87.1 136.1 Total fat intake (% of total energy) 40.2 6.4  2  Age (years) 53.2 5.3  BMI (kg/m2) 26.8 3.5  Leisure-time physical activity (kJ/d) 558.6 638.9  Leisure-time physical activity (kcal/d) 133.5 152.7  Smokers (%) 32.4  Alcohol intake (g/week) 72.3 106.0  Total fat intake (% of total energy) 38.6 6.4   3  Age (years) 52.4 5.3  BMI (kg/m2) 26.7 3.5  Leisure-time physical activity (kJ/d) 670.7 825.5  Leisure-time physical activity (kcal/d) 1603 197.3  Smokers (%) 28.9  Alcohol intake (g/week) 69.7 115.7  Total fat intake (% of total energy) 37.5 6.1   4 (highest) Age (years) 52.2 5.3 BMI (kg/m2) 26.5 3.5 Leisure-time physical activity (kJ/d) 605.4 677.4 Leisure-time physical activity (kcal/d) 144.7 161.9 Smokers (%) 19.9  Alcohol intake (g/week) 60.5 95.2 Total fat intake (% of total energy) 38.1 5.6  P values Age (years) 0.001 BMI (kg/m2) 0.506 Leisure-time physical activity (kJ/d) , 0.001 Leisure-time physical activity (kcal/d) , 0.001 Smokers (%) , 0.001 Alcohol intake (g/week) 0.003 Total fat intake (% of total energy) , 0.001  **Location**  Finland  **Recruitment strategy** Not reported  **Length of follow-up**  average follow-up time of 15·2 years  **Response rate and loss to follow-up** 82·9% of those eligible. There were no losses to follow-up  **Eligible population** Not reported  **Excluded populations** Men who had history of CHD or stroke were excluded from the study | | | | **Relevant exposures** smoking and alcohol **Time** March 1984 - December 1989 **Measurment of exposure** The number of cigarettes, cigars and pipefuls of tobacco currently smoked daily, duration of regular smoking in years, alcohol consumption,  recorded with a self-administered questionnaire  consumption of foods was assessed with an instructed 4 d food recording by household measures | | **Outcomes** ischaemic strokes and CVD deaths, atherosclerosis and related outcomes  **outcome measurement** 1984 and 1992 information of strokes were collected prospectively  1993 post obtained by computer linkage to the national hospital discharge and death registers  CVD deaths were ascertained by computer linkage to the national death registry using the Finnish social security number  **Time**  1984 - 1992 and 1993  CVD deaths from study entry to 31 December 2004 | | **Analysis strategy** Cox proportional hazards model  **Confounders** age and examination years, BMI, systolic blood pressure, hypertension medication, serum HDL- and LDL-cholesterol, serum TAG, maximal oxygen uptake, smoking, family history of CVD, diabetes, alcohol intake, energy-adjusted intake of folate, vitamin E, total fat and saturated fat intake (percentage of energy) | | **Number** 102 ischaemic strokes and 153 CVD deaths  **Effect estimates** Quartiles of flavonoid intake (mg/d) 1 Ischaemic stroke Flavonols 1 Flavones 1 Flavanones 1 Flavan-3-ols 1 Anthocyanidins 1 Total sum of flavonoids 1  CVD mortality Flavonols 1 Flavones 1 Flavanones 1 Flavan-3-ols 1 Anthocyanidins 1 Total sum of flavonoids 1  2  RR 95 % CI Ischaemic stroke  Flavonols 0.68 0.40, 1.14 Flavones 1.12 0.60, 2.11 Flavanones 0.83 0.47, 1.47 Flavan-3-ols 1.24 0.73, 2.10 Anthocyanidins 0.89 0.48, 1.63 Total sum of flavonoids 1.65 0.98, 2.79  CVD mortality  Flavonols 1.22 0.77, 1.94 Flavones 0.64 0.40, 1.05 Flavanones 0.60 0.37, 0.98 Flavan-3-ols 1.29 0.82, 2.04 Anthocyanidins 0.51 0.30, 0.87 Total sum of flavonoids 1.85 1.18, 2.90   3  RR 95 % CI Ischaemic stroke  Flavonols 0.54 0.30, 0.95 Flavones 2.05 1.15, 3.65 Flavanones 0.97 0.56, 1.71 Flavan-3-ols 1.02 0.58, 1.80 Anthocyanidins 1.58 0.91, 2.71 Total sum of flavonoids 1.00 0.55, 1.81  CVD mortality  Flavonols 1.42 0.88, 2.28 Flavones 0.64 0.40, 1.02 Flavanones 0.97 0.62, 1.50 Flavan-3-ols 1.03 0.64, 1.65 Anthocyanidins 1.17 0.74, 1.86 Total sum of flavonoids 1.05 0.63, 1.74  4 (highest) RR 95 % CI Ischaemic stroke  Flavonols 0.55 0.31, 0.99 Flavones 1.30 0.69, 2.47 Flavanones 0.89 0.49, 1.63 Flavan-3-ols 0.59 0.30, 1.14 Anthocyanidins 0.88 0.47, 1.62 Total sum of flavonoids 0.71 0.37, 1.37  CVD mortality  Flavonols 1.26 0.75, 2.14 Flavones 0.65 0.40, 1.05 Flavanones 0.54 0.32, 0.92 Flavan-3-ols 1.06 0.64, 1.65 Anthocyanidins 0.99 0.62, 1.85 Total sum of flavonoids 1.25 0.74, 2.11  P trend Ischaemic stroke Flavonols 0.027 Flavones 0.181 Flavanones 0.870 Flavan-3-ols 0.102 Anthocyanidins 0.813 Total sum of flavonoids 0.137  CVD mortality  Flavonols 0.622 Flavones 0.333 Flavanones 0.266 Flavan-3-ols 0.694 Anthocyanidins 0.193 Total sum of flavonoids 0.730  **Significant trends** a high intake of flavonoids decreases the risk of ischaemic stroke and possibly CVD mortality  **Reported limitations  Author** could not study the main sources of flavonoids. Seasonal variation in the 4d food recording  **Source of funding** Juho Vainio Foundation and Finnish Cultural Foundation, North-Savo Foundation | |
| **Authors:** Nafziger AN, Lindvall K, Norberg M, Stenlund H, Wall S  **Year:** 2007  **Citation:** BMC Public Health. 2007;7:108  **Country of study**: Sweden  **Aim of study:** Characterize who is not gaining weight during a 10 year period in Sweden  **Study design:** Longitudinal survey  **Quality score: (++, + or -):**  **External validity score: (++, + or -):** | | | | | | | | | | | | | |
| **Number of people**  23,863  **Demographics** Non-participants (%) Age (years)  30 29.9% 40 39.3% 50 30.8% Sex Male 50.4% Female 49.6% Education Low 21.8% Medium 52.6% High 25.6% Smoker 30.4% Snuff use 28.7% Physically inactive 42.9% | | **Number of people** 14,867 **Characteristics**  Age (years) 3023.4% 40 39.7% 50 36.9% Sex Male 46.6% Female 53.4% Education Low 22.8% Medium 53.9% High 23.4% Smoker 24.8% Snuff use 25.6% Physically inactive 41.5%  **Location**  Västerbotten, Sweden  **Recruitment strategy** Not reported  **Length of follow-up**  10 years  **Response rate and loss to follow-up** The overall follow-up rate was 68.1%; the response rate among the eligible was 74%  **Eligible population** men and women aged 30, 40 or 50 years at baseline  **Excluded populations** Participants who lacked a BMI were excluded. Participants with an initial BMI <18.5 or ≥30 kg/m2 were excluded. 1062 participants who moved out of the county, 503 individuals who died, and 7 who could not be located because of assignment of an anonymous civil number | | | | **Relevant exposures** Physical activity, smoking and snuff use **Time** 1990 - 1994 **Measurement of exposure** Participants completed a questionnaire that included questions use of tobacco products, physical activity. Participants were classified as smokers (yes/no) and snuff users (Swedish moist snuff (snus); yes/no) | | **Outcomes** Weight gain  **outcome measurement** BMI  **Time**  2000-2004 | | **Analysis strategy** multivariate logistic regression model  **Confounders** Not reported | | **Number** 9625 categorised as gainers  **Effect estimates** Odds Ratio (95% CI) Men  30 yr 1.00 40 yr 1.13 (0.99, 1.30) 50 yr 2.24 (1.96, 2.56) Women 30 yr 1.14 (0.98, 1.32) 40 yr 1.17 (1.03, 1.35) 50 yr 1.50 (1.16, 1.34) Body mass index (kg/m2) 18.5–24.9 1.00 25–29.9 1.25 (1.16, 1.34) Year of initial survey 1990 1.00 1991 1.19 (1.04, 1.36) 1992 1.38 (1.21, 1.56) 1993 1.43 (1.26, 1.62) 1994 1.74 (1.53, 1.98) Glucose metabolism Normal 1.00 Glucose intolerance 1.15 (0.94, 1.40) Type 2 diabetes 1.47 (1.08, 1.99) Snuff use No 1.00 Yes 0.83 (0.74, 0.92)  **Significant trends** Older age, being female, classified as overweight by baseline BMI, later survey year and baseline diagnosis of diabetes increased the chances of not gaining weight. Those who did not use snuff also were more likely to be non-gainers.  **Reported limitations  Author** participation rates were not optimal. Participants in this study were more likely to be of older age, women, lower education, lower baseline BMI and less likely to have cardiovascular risk factors - differences between participants and non-participants should have resulted in more conservative odds ratios  **Reviewer** Fail to report confounders in analysis  **Source of funding** AFA-Insurance Sweden | |
| **Authors:** Nakamura Y, Hozawa A, Turin TC, Takashima N, Okamura T  **Year:** 2009  **Citation:** Gerontology. 2009;55:707–713  **Country of study:** Japan  **Aim of study:** Examine the association of meat, fish and egg intake with risk of subsequent mortality and/or future decline in activities of daily living  **Study design:** Cohort  **Quality score: (++, + or -):**  **External validity score: (++, + or -):** | | | | | | | | | | | | | |
| **Number of people**  3,227  **Demographics** Not reported | | **Number of people** 1,889 **Characteristics**  Men Meat <1/2 days  Number 375  Age, years 53.2±3.6  BMI 22.4±2.9  Smoking, % 69.9  Daily drinking, % 49.1  Professional work, % 33.7  Urban residence, % 25.3   ≥1/2 days  Number 667  Age, years 52.9±3.5  BMI 22.7±2.7  Smoking, % 65.4  Daily drinking, % 52.5  Professional work, % 40.7  Urban residence, % 31.0   Fish <1/day  Number 555  Age, years 52.9±3.5  BMI 22.5±2.9  Smoking, % 68.5  Daily drinking, % 47.9  Professional work, % 41.1  Urban residence, % 31.2   ≥1/day  Number 487  Age, years 53.1±3.5  BMI 22.7±2.7  Smoking, % 65.3  Daily drinking, % 55.0  Professional work, % 34.9  Urban residence, % 26.5   Egg  <1/day  Number 599  Age, years 53.1±3.5  BMI 22.7±2.8  Smoking, % 66.9  Daily drinking, % 51.6  Professional work, % 38.7  Urban residence, % 30.4   ≥1/day  Number 443 Age, years 52.9±3.5 BMI 22.4±2.9 Smoking, % 67.0 Daily drinking, % 50.8 Professional work, % 37.5 Urban residence, % 27.1  P Meat Age, years 0.27 BMI 0.15 Smoking, 0.13  Daily drinking, 0.29 Professional work, 0.03 Urban residence, 0.05  Fish Age, years 0.52 BMI 0.14 Smoking, 0.28 Daily drinking, 0.02 Professional work, 0.04 Urban residence, 0.10  Egg Age, years 0.51 BMI 0.048 Smoking, 0.97 Daily drinking, 0.80 Professional work, 0.69 Urban residence, 0.25  Women Meat <1/2 days  Number 573  Age, years 53.6±3.8  BMI 23.3±3.6  Smoking, % 9.8  Daily drinking, % 1.8  Professional work, % 15.4  Urban residence, % 28.5     ≥1/2 days  Number 701  Age, years 53.1±3.8  BMI 23.3±3.2  Smoking, % 6.0  Daily drinking, % 2.0  Professional work, % 22.0  Urban residence, % 32.0    Fish <1/day  Number 750  Age, years 53.1±3.7  BMI 23.4±3.4  Smoking, % 8.8  Daily drinking, % 2.3  Professional work, % 17.6  Urban residence, % 32.0    ≥1/day  Number 524  Age, years 53.5±3.9  BMI 23.2±3.4  Smoking, % 6.1  Daily drinking, % 1.3  Professional work, % 21.0  Urban residence, % 28.1   Egg  <1/day  Number 877  Age, years 53.3±3.8 BMI 23.4±3.4 Smoking, % 7.8  Daily drinking, % 1.6  Professional work, % 18.5  Urban residence, % 29.8    ≥1/day  Number 397 Age, years 53.3±3.7  BMI 23.2±3.3  Smoking, % 7.6  Daily drinking, % 2.5  Professional work, % 20.2  Urban residence, % 31.7   P Meat Age, years 0.02 BMI 0.98 Smoking, % 0.01 Daily drinking, % 0.74 Professional work, % 0.003 Urban residence, % 0.18   Fish Age, years 0.10  BMI 0.39  Smoking, % 0.08  Daily drinking, % 0.23  Professional work, % 0.13  Urban residence, % 0.13  Egg Age, years 0.997 BMI 0.39 Smoking, % 0.90 Daily drinking, % 0.26 Professional work, % 0.47 Urban residence, % 0.48  **Location**  300 districts  **Recruitment strategy** Participants from 300 randomly selected districts in 1980  **Length of follow-up**  19 years  **Response rate and loss to follow-up** 75%   **Eligible population** aged 47–59 years  **Excluded populations** 286 participants who had a history of coronary heart disease (CHD) or stroke (n = 39), had missing information in the baseline survey (n = 54) or were lost to follow-up (n = 193), 427 died | | | | **Relevant exposures** Diet **Time** 1980 **Measurement of exposure** A lifestyle survey was also carried out using a self-administered questionnaire which included the daily consumption of meat, eggs and fish. | | **Outcomes** activities of daily living  **outcome measurement** Participants were asked about 5 basic ADL items (modified from Katz et al.)  **Time**  1999 | | **Analysis strategy** multiple adjusted logistic regression model  **Confounders** age, BMI and cigarette smoking, alcohol drinking, hypertension and diabetes, serum albumin and total cholesterol concentrations, job type and urban residence | | **Number** 75 participants became dependent due to impaired ADL  **Effect estimates** Associations of impaired ADL or death and food intake in the meat, fish and egg intake groups Odds ratio (CI 95%) Meat  <1/2 days 1  ≥1/2 days 0.91 (0.73–1.12)  P 0.36  Fish  <1/day 1  ≥1/day 1.08 (0.87–1.33)  p 0.50  Egg  <1/day 1 ≥1/day 1.09 (0.88–1.35)  P 0.43  Associations of death and food intake in in the meat, fish and egg intake groups Odds ratio (CI 95%) Meat  <1/2 days 1  ≥1/2 days 1.00 (0.80–1.25)  P 0.99  Fish  <1/day 1  ≥1/day 1.06 (0.84–1.32)  P 0.65  Egg  <1/day 1  ≥1/day 1.13 (0.90–1.42)  P 0.29  Associations of impaired ADL and food intake in the meat, fish and egg intake groups Odds ratio (CI 95%) Meat  <1/2 days 1  ≥1/2 days 0.61 (0.38–0.99)  P 0.04  Fish  <1/day 1  ≥1/day 1.25 (0.76–1.95)  P 0.42  Egg  <1/day 1  ≥1/days 0.90 (0.54–1.49)  P 0.68  **Significant trends** higher intake of meat was associated with a statistically significant decrease in impaired ADL occurrence. Fish and egg intake were not associated with any difference in impaired ADL occurrence. None of the 3 foods were associated with any changes in mortality  **Reported limitations  Author** Did not assess the baseline ADL condition. No information on SES other than professional work and urban residence. Diet may have changed during 19 years  **Source of funding** Grant-in-Aid from the Ministry of Health and Welfare under the auspices of the Japanese Association for Cerebro-Cardiovascular Disease Control, a Research Grant for Cardiovascular Diseases (7A-2) from the Ministry of Health, Labor and Welfare and a Health and Labor Sciences Research Grant, Japan (Comprehensive Research on Aging and Health: H11-chouju-046, H14-chouju-003, H17-chouju-012 and H19-chouju-014). | |
| **Authors:** Nakayama T, Yokoyama T, Yoshiike N, Zaman MM, Tanaka H, et al.  **Year:** 2000  **Citation:** Neuroepidemiology. 2000;19:217–226  **Country of study:** Japan  **Aim of study:** Determined the population attributable fraction of stroke due to hypertension, atrial fibrillation and smoking - to quantify the proportion of stroke that might be prevented  **Study design:**  **Quality score: (++, + or -):**  **External validity score: (++, + or -):** | | | | | | | | | | | | | |
| **Number of people**  2,302  **Demographics** Not reported | | **Number of people** 998 **Characteristics**  Not reported  **Location**  Shibata, Japan  **Recruitment strategy** Not reported  **Length of follow-up**  20 years  **Response rate and loss to follow-up** Response rate 69%  **Eligible population** All residents aged 40 years and over  **Excluded populations** Those with a previous history of stroke. Nonrespondents in the initial survey included 184 men and 109 women, of whom 28 men and 29 women were hospitalized or were treated at clinics and 14 men and 14 women were being cared for at home | | | | **Relevant exposures** Smoking **Time** 1977 **Measurment of exposure** Not reported | | **Outcomes** Stroke  **outcome measurement** Laboratory and diagnostic imaging examined by clinicians  **Time**  Follow-up examination conducted annually | | **Analysis strategy** Cox regression model  PAF was calculated by pd x ((RR – 1)/RR) where pd = proportion of cases exposed to the risk factor.  C**onfounders** BP status, Af smoking and physical activity, diabetes mellitus, obesity, drinking and age at baseline | | **Number** 213  **Effect estimates** Adjusted RRs and PAFs of stroke incidence due to hypertension, smoking and atrial fibrillation RR 95% CI Men hypertension  controlled 0.96 (0.40–2.29) untreated 3.94 (1.79–8.68) uncontrolled 4.92 (2.19–11.05) smoking 1.77 (0.96–3.27) Af 1.94 (0.24–15.90)  Women hypertension  controlled 1.35 (0.60–3.04) untreated 3.31 (1.24–8.83) uncontrolled 3.39 (0.99–11.64) smoking 0.00 (0.00–0.00) Af 92.23 (25.34–335.62)  All hypertension  controlled 1.07 (0.59–1.92) untreated 3.61 (2.01–6.50) uncontrolled 3.69 (1.89–7.19) smoking 1.84 (1.00–3.40) Af 11.24 (3.72–33.97)  PAF, % (90% CI) Men hypertension  controlled – –  untreated 16.6 (5.7–26.2) uncontrolled 13.3 (4.0–21.6) smoking 26.6 (0.7–45.7) Af 0.9 (–2.3–4.0)  Women  hypertension  controlled 4.3 (–6.9–14.4) untreated 10.2 (0.6–18.8) uncontrolled 4.4 (–1.7–10.1) smoking – Af 6.2 (0.2–11.8)  All hypertension  controlled 0.9 (–6.5–7.8) untreated 13.5 (6.3–20.1) uncontrolled 8.6 (2.9–13.9) smoking 14.9 (2.3–26.0) Af 3.6 (0.3–6.7)  **Significant trends** control of smoking in Japan is the most substantial single factor for reducing the incidence of stroke in middleaged men. ack of treatment and control of hypertension is responsible for approximately one third of strokes in the middle-aged population of Shibata.  **Reported limitations  Author** misclassification and change of exposure. Smoking was self-reported. limited sample size  **Reviewer** No or little reporting of patient characteristics or study methodology  **Source of funding** Ministry of Health and Welfare, Japan: Nervous and Mental Disorders (grant 3A-3) from the National Center of Neurology and Psychiatry; Cardiovascular Diseases (grant 3C-2) from the National Cardiovascular Center, and a grant from the Japan Foundation for Aging and Health | |
| **Authors:** Noborisaka Y, Ishizaki M, Yamada Y, Honda R, Yokoyama H, et al.  **Year:** 2013  **Citation:** Environ Health Prev Med. 2013;18:24–32  **Country of study:** Japan  **Aim of study:** Examine association between smoking and the development of chronic kidney disease  **Study design:** Retrospective 6-year observational study  **Quality score: (++, + or -):**  **External validity score: (++, + or -):** | | | | | | | | | | | | | |
| **Number of people**  20,782  **Demographics** Not reported | | **Number of people** 6,998 **Characteristics**  Men (n = 4,121) Age (years) <29 587 (14.2)  30–59 3,473 (84.3)  >60 61 (1.5)  BMI (kg/m2) <18.4 161 (3.9)  18.5–24.9 2,920 (70.9)  25.0–29.9 928 (22.5)  >30.0 112 (2.7)  Cigarettes Never smoked 1,028 (24.9)  Ex-smoker 904 (21.9)  Smoke up to >1 pack/day 1,540 (37.4)   Smoke >1 pack/day 649 (15.7)  Alcohol Usually not drinking 1,246 (30.2)  Up to 69 mL/week of ethanol 1,474 (35.8)  70–209 mL/week 1,256 (30.5)  >70–209 mL/week 145 (3.5)  Occupation Clerks 1,000 (24.3 %)  Managers/professionals 1,144 (27.8 %)  Operators/drivers 1,223 (29.7 %)  Service/sales 636 (15.4 %)  Others 118 (2.9 %)   Women (n = 2,877) Age (years) <29 344 (12.0) 30–59 2,484 (86.3) >60 49 (1.7)  BMI (kg/m2) <18.4 356 (12.4) 18.5–24.9 2,122 (73.8) 25.0–29.9 348 (12.1) >30.0 51 (1.8) Cigarettes Never smoked 2,539 (88.3)  Ex-smoker 57 (2.0)  Smoke up to >1 pack/day 273 (9.5)  Smoke >1 pack/day 8 (0.3) Alcohol Usually not drinking 2,088 (72.6)  Up to 69 mL/week of ethanol 673 (23.4) 70–209 mL/week 108 (3.8)  >70–209 mL/week 8 (0.3)  Occupation Clerks 1,129 (39.2 %)  Managers/professionals 600 (20.9 %)  Operators/drivers 342 (11.9 %)  Service/sales 656 (22.8 %)  Others 150 (5.2 %)  **Location**  Ishikawa, Japan  **Recruitment strategy** recruited from workplaces  **Length of follow-up**  6-year  **Response rate and loss to follow-up** 87.9 %  **Eligible population** people working in 447 various kinds of workplaces  **Excluded populations** those whose data for body weight (37 men, 50 women), urinalysis (2 men, 114 women), or PG (7 men) were not available were excluded; 13 men and five women who declared a past history of primary kidney disease; 252 men and 50 women with high PG consistent DM; 28 men and 12 women who showed severe hypertension in 2003; 4,298 men and 3,096 women showing CKD signs in 2003; five men and 88 women who did not undergo urinalysis in 2009 | | | | **Relevant exposures** cigarette and alcohol consumption **Time** either 2009 or 2003 **Measurment of exposure** Data on cigarette and alcohol consumption were obtained by interview  Smoking habits were classified into four categories, namely, lifelong non-smokers, exsmokers, current smokers consuming up to one pack per day, and smokers consuming more than one pack per day  alcohol consumption was categorized into four levels, ‘‘non-drinkers’’, ‘‘mild drinkers’’, ‘‘moderate drinkers’’ and ‘‘heavy drinkers’’ | | **Outcomes** chronic kidney disease  **outcome measurement** categorisation in subjects based on single measurements of proteinuria and eGFR  **Time**  2009 | | **Analysis strategy** Multiple logistic regression  **Confounders** sex, age, BMI, BP levels, alcohol consumption, the presence of IGR and dyslipidemia, and occupation | | **Number** Sixty men (1.5 %) and 21 women (0.7 %) developed proteinuria  **Effect estimates** Proteinuria  Odds ratio (95 % CI) p Sex (women/men) 1.06 (0.57–1.98) 0.859 Age (year) 1.02 (0.99–1.05) 0.134 BMI levels (1–4) 1.82 (1.27–2.59) 0.001 Blood pressure levels (1–5) 1.33 (1.10–1.62) 0.004 IGR 2.35 (1.19–4.65) 0.014 hChol 1.46 (0.90–2.36) 0.130 lHDLc 1.33 (0.64–2.79) 0.449 hTG 1.20 (0.68–2.13) 0.533 hGFR 1.35 (0.52–3.45) 0.538 Smoking status (vs. non-smokers) 0.002 Ex-smokers 1.29 (0.48–3.42) 0.614 Continuous smokers 2.52 (1.50–4.25) 0.001 Alcohol consumption levels (1–4) 0.87 (0.66–1.16) 0.349 Occupations (1–5) 1.01 (0.84–1.21) 0.941  Low eGFR  Odds ratio (95 % CI) p Sex (women/men) 1.02 (0.84–1.24) 0.866 Age (year) 1.08 (1.07–1.09) <0.001 BMI levels (1–4) 1.37 (1.19–1.57) <0.001 Blood pressure levels (1–5) 0.97 (0.90–1.04) 0.347 IGR 0.84 (0.59–1.18) 0.312 hChol 1.06 (0.90–1.27) 0.480 lHDLc 0.92 (0.65–1.29) 0.625 hTG 1.44 (1.14–1.80) 0.002 hGFR 0.05 (0.01–0.22) <0.001 Smoking status (vs. non-smokers) 0.006 Ex-smokers 1.05 (0.78–1.41) 0.735 Continuous smokers 0.74 (0.60–0.90) 0.003 Alcohol consumption levels (1–4) 0.87 (0.78–0.97) 0.009 Occupations (1–5) 0.98 (0.92–1.04) 0.433  **Significant trends** Continuing smokers showed a twofold or more higher risk of developing proteinuria. Discontinuation of smoking substantially reduced the risk  **Reported limitations  Reviewer** the clinical definition of CKD requires CKD signs to be detectable for >3 months.  **Source of funding** KAKENHI, a Grant-in-Aid for Scientific Research (C), 2010, from the Japan Society for the Promotion of Science | |
| **Authors**: Nokes NR, Tucker LA **Year:** 2012 **Citation:** Am J Health Promot. 2012;26(6):341-347 **Country of study:** US **Aim:** To determine the influence of physical activity volume and intensity on bone mineral density (BMD) of the hip **Study design:** Longitudinal  **Quality score:** + **Applicability:** | | | | | | | | | | | | | |
| **Source population:**  *268 women ages 35-45 years recruited at baseline using newspaper advertisements and flyers circulated in workplaces and churches in at least 20 cities in the Mountain West | | **Study population:**  ***244** participants presented to follow-up assessment 6 years later  *Follow-up:* *6 years   *Sociodemographics:* *sample 90% white **Exclusion:**  *smokers *women who were ill according to physical activity readiness questionnaire  **Attrition:**  *9% attrition rate *women who did not present to follow-up assessment were not significantly different from those who completed baseline and follow-up exams in terms of age, baseline hip BMD, baseline weight or objectively measured PA | | | | **Exposures:**  *physical activity (PA) volume (sum of all activity counts or total volume of movement) and physical activity intensity (variations of activity within a specified time period) measured over 7 days after baseline assessment using valid and reliable accelerometers *PA volume divided into ‘low’, ‘moderate’, and ‘high’ *accelerometer activity counts within a specific time period (of 10 min.) reflected physical activity intensity  *as per the 2007 American Heart Association and American College of Sports medicine guidelines, participants were required to participate in physical activity, and were categorized into the following PA intensity ranges: 1) ‘vigorous’: for those with at least 50,000 PA counts during at least 15 bouts (150 min.); 2) ‘moderate’: at least 30,000 counts during a minimum of 15 bouts; 3) ‘low’: at least 30,000 counts in fewer than 15 bouts | | **Outcomes:**  *at baseline and at 6 year follow-up, participants had their hip scanned on the valid and reliable bone densitometer to determine bone mineral density (BMD) (predicts risk of hip fracture) *BMD change was calculated by subtracting follow-up BMD score from baseline score or value of the total hip with scores divided into quartiles and participants categorized into the following groups: ‘ BMD loss’ (<25th percentile); ‘minimal BMD change’ (25-75th percentile); ‘BMD gain’ (>75th percentile) | | **Analysis:**  *power analysis determined that 155 participants needed for effect size of 0.25 with power of 0.80 *Poisson regression was used to determine influence of PA on BMD  **Confounders:**  *age, baseline hip BMD, baseline body weight, weight change, time in study, menopause status, maternal history of osteoporosis, calcium intake, vitamin D intake | | **Results:**  *59% of participants lost BMD at the hip during follow-up *over 6 years, women with moderate to high PA volume levels were more likely to have hip BMD gains compared to women in the low PA volume group (RR=2.01, [1.05, 3.81]) *over 6 years, women with moderate PA volume levels were more likely to gain BMD at the hip than women with low PA volume (RR=1.97, [1.02, 3.79])  *physical activity intensity was not significant in in predicting changes in BMD  **Limitations:**  *limited generalizability due to homogeneous sample *not known whether PA status may have changed during follow-up and if this may have influenced outcome  **Source of Funding:**  *none reported | |
| **Authors:** Nooyens AC, Bueno-de-Mesquita HB, van Boxtel MP, van Gelder BM, Verhagen H, et al.  **Year:** 2011  **Citation:** Br J Nutr. 2011;106:752–761  **Country of study:** Netherlands  **Aim of study:** Habitual fruit and vegetable intake was studied in association with cognitive function and cognitive decline  **Study design:** Cohort  **Quality score: (++, + or -):**  **External validity score: (++, + or -):** | | | | | | | | | | | | | |
| **Number of people**  7769  **Demographics** Not reported | | **Number of people** 2613 **Characteristics**  Quartile 1 Mean SD N 522  Range of total fruit and vegetable intake (g) 50 – 199  Age (years) 54.3 6.6 Sex (% women) 31.2  Highly educated† (%) 48.3  Cigarette smoker at baseline (%) 35.3  Number of pack years smoked in life 22.8 15.8  Excessive consumption of alcohol (%) 24.3  Inactive (%) 32.8  Vitality 67 17  Mental health 77 16  Quartile2 N 523 Range of total fruit and vegetable intake (g) 199 – 265 Age (years) 54.7 6.7 Sex (% women) 46.9  Highly educated† (%) 50.9  Cigarette smoker at baseline (%) 22.9  Number of pack years smoked in life 18.3 15.5  Excessive consumption of alcohol (%) 13.6  Inactive (%) 26.4  Vitality 67 17  Mental health 77 15   Quartile3 N 523 Range of total fruit and vegetable intake (g) 265 – 334 Age (years) 55.4 7.0 Sex (% women) 54.1  Highly educated† (%) 49.1  Cigarette smoker at baseline (%) 19.9  Number of pack years smoked in life 16.7 16.0  Excessive consumption of alcohol (%) 14.2  Inactive (%)22.6  Vitality 68 18  Mental health 77 14   Quartile4 N 523 Range of total fruit and vegetable intake (g) 334 – 415 Age (years) 55.8 7.3 Sex (% women) 57.0  Highly educated† (%) 51.1  Cigarette smoker at baseline (%) 16.3  Number of pack years smoked in life 15.8 13.8  Excessive consumption of alcohol (%) 14.9  Inactive (%) 23.7  Vitality 68 17  Mental health 77 14   Quartile5 N 522 Range of total fruit and vegetable intake (g) 415 – 1131 Age (years) 56.0 6.8 Sex (% women) 64.4  Highly educated† (%) 55.0  Cigarette smoker at baseline (%) 15.5  Number of pack years smoked in life 14.5 13.3  Excessive consumption of alcohol (%) 12.1  Inactive (%) 19.4  Vitality 68 16 Mental health 77 14  P trends (P,0.05) Age  Sex  Highly educated Cigarette smoker at baseline  Number of pack years smoked in life Excessive consumption of alcohol Inactive  **Location**  Doetinchem  **Recruitment strategy** From Doetinchem Cohort Study  **Length of follow-up**  5-year  **Response rate and loss to follow-up** 80%  **Eligible population** 1995–7, a random sample of one-third of participants aged 45 years and older was enrolled in the study on cognitive functioning  **Excluded populations** Participants who reported having experienced a stroke (n 77) | | | | **Relevant exposures** Diet, smoking and physical activity **Time** 1995–2002 **Measurement of exposure** self-administered semi-quantitative FFQ was used to assess the habitual consumption of 178 food items during the previous year.Averaged reported intakes at baseline and follow-up  smoking and physical activity recorded in self-report questionnaire. Smoking status defined as 'non-smoker' or 'current smoker'   Physical activity level was assessed by the EPIC questionnaire on physical activity | | **Outcomes** Cognitive decline  **outcome measurement** neuropsychological test battery  **Time**  2003–7 | | **Analysis strategy** multivariate linear regression analyses  **Confounders** age, sex, level of education, total energy intake, intake of other fruits, vegetables, legumes and juices, and the baseline level of cognitive function, physical activity, smoking, systolic blood pressure, use of blood pressure-lowering medication, serum HDL-cholesterol, waist circumference, coffee consumption, vitality and mental health | | **Number** Not reported  **Effect estimates** Average change in cognitive function domains: -0.14 (SD 0.77) for memory -0.13 (SD 0.45) for information processing speed -0.08 (SD 0.66) for cognitive flexibility -0.10 (SD 0.42) for global cognitive function  Baseline  β P-trend * P,0.05, ** P,0.01. Fruits and vegetables  Memory 0.03  Speed - 0.01  Flexibility - 0.01  Global 0.03  Fruits  Memory 0.01  Speed 0.01  Flexibility 0.02  Global 0.03  Vegetables  Memory 0.03  Speed - 0.04 * Flexibility - 0.04 * Global 0.00  Legumes  Memory - 0.02  Speed 0.00  Flexibility 0.00  Global 0.00  Juices  Memory 0.00  Speed - 0.01  Flexibility - 0.02  Global - 0.02   Change Fruits and vegetables  Memory 0.00  Speed 0.03  Flexibility 0.00  Global 0.02  Fruits  Memory 0.00  Speed 0.01  Flexibility - 0.01  Global 0.01  Vegetables  Memory 0.03  Speed 0.07 ** Flexibility 0.03 Global 0.05 ** Legumes  Memory 0.03  Speed - 0.02  Flexibility 0.01  Global 0.03  Juices  Memory 0.01  Speed - 0.03  Flexibility - 0.01  Global 0.00  **Significant trends** total intake of fruits and vegetables was not or inconsistently associated with cognitive function and cognitive decline. Nuts had statistically significant association between fruit consumption and cognitive decline  **Reported limitations  Author** baseline characteristics were different. unfavourable effect of allium is not explained  **Reviewer** crude measure of habitual intake   **Source of funding** Ministry of Public Health, Welfare and Sport of The Netherlands and the National Institute for Public Health and the Environment. The data up to and including 1997, including the dietary assessment method, were additionally financially supported by the Europe against Cancer programme of the European Commission | |
| **Authors:** Nooyens AC, van Gelder BM, Verschuren WM  **Year:** 2008  **Citation:** Am J Public Health. 2008;98:2244–2250  **Country of study:** Netherlands  **Aim of study:** Studied the effect of smoking on cognitive decline  **Study design:** Cohort Study  **Quality score: (++, + or -):**  **External validity score: (++, + or -):** | | | | | | | | | | | | | |
| **Number of people**  2434  **Demographics** Not reported | | **Number of people** 1964 **Characteristics**  Age, y, mean (SD) 56.0 (7.0) Men, % 48.5 Married, % Lifelong cigarette smoking, pack-years, % 85.1 0 36.4 0–20 38.9 > 20 24.8 Level of education, %  Primary school 7.6 Lower vocational 26.4 Intermediate secondary 17.5 Intermediate vocational/higher secondary 24.3 Higher vocational/university 24.2 Cardiovascular risk factors Total cholesterol, mmol/L, mean (SD) 5.9 (1.0) Elevated serum cholesterol level, % 30.0 HDL cholesterol, mmol/L, mean (SD) 1.38 (0.39) Systolic blood pressure, mm Hg, mean (SD) 131.1 (17.8) Hypertension, % 35.6 Body mass index, kg/m2, mean (SD) 26.3 (3.8) Self-reported diabetes or cardiovascular disease, % 4.1 Physically active, % 56.2 Alcohol consumption, %  No alcohol use 30.7 0–1 glass/day 27.9 1–2 glasses/day 20.0 2–4 glasses/day 16.4 > 4 glasses/day 5.0 Total energy intake, MJ/day 8.9 (2.3)  **Location**  Doetinchem  **Recruitment strategy** From Doetinchem Cohort Study  **Length of follow-up**  5 years  **Response rate and loss to follow-up** 71% of the eligible population took part in the cognitive testing at baseline  **Eligible population** all participants of the Doetinchem Cohort Study aged 45 years and older  **Excluded populations** Participants who reported a cerebrovascular accident (n=60) | | | | **Relevant exposures** smoking, diet, alcohol consumption, and physical activity **Time** 1993–1997, 1998–2002, and 2003–2007 **Measurement of exposure** Smoking status categorised as persistent nonsmoker; ex-smoker; persistent smoker, or recent quitter, resumed smoking  Alcohol consumption classified: (1) no alcohol use, (2) 0 to 1 glass per day, (3) 1 to 2 glasses per day, (4) 2 to 4 glasses per day, and (5) more than 4 glasses per day  physical activity was dichotomized as less versus more than half an hour per day of at least moderate-intensity physical activities  food-frequency questionnaire was used to assess the habitual consumption of 178 food items during the previous year | | **Outcomes** Cognitive decline  **outcome measurement** neuropsychological test battery  15-Word Verbal Learning Test  Stroop Color–Word Test  Animal Naming Verbal Fluency Test  **Time**  1995 - January 2000 | | **Analysis strategy** multivariate linear regression analyses  **Confounders** age, gender, level of education, alcohol consumption, hypertension, serum total and high-density lipoprotein cholesterol, body mass index, diabetes or cardiovascular disease, and physical activity), energy intake, total fat intake, coffee consumption, fish consumption, antioxidant intake, estrogen use, and marital status | | **Number** Not reported  **Effect estimates** Effect of the Number of Cigarettes Smoked on Change in Cognitive Functions b P Memory function (n = 1162) –0.04 .03 Speed of cognitive processes (n = 1161) –0.02 .03 Cognitive flexibility (n = 1165) –0.03 .04 Global cognitive function (n = 1146) –0.02 .06  **Significant trends** no difference between smokers and never smokers in rates of decline in the speed of cognitive processes during follow-up. decline among smokers was 1.9 times greater for memory function, 2.4 times greater for cognitive flexibility, and 1.7 times greater for global cognitive function than among never smokers  **Reported limitations  Reviewer** No figure given for numbers of participants with cognitive decline  **Source of funding** Ministry of Health, Welfare and Sport of the Netherlands and the National Institute for Public Health and the Environment. | |
| **Authors**: Osler M, Andreasen AH, Hoidrup S **Year:** 2003 **Citation:** [J Clin Epidemiol.](http://www.ncbi.nlm.nih.gov/pubmed/12725883) 2003;56(3):274-249  **Country of study:** Denmark **Aim:** To determine the association between fish consumption and risk of all-cause mortality, and fatal and nonfatal coronary heart disease  **Study design:** Longitudinal **Quality score:** + **Applicability:** | | | | | | | | | | | | | |
| **Source population:**  *5 population studies conducted at Copenhagen County Centre for Preventive Medicine *birth cohorts of Western suburbs of Copenhagen followed since 1964 *411 men and 391 women born in 1914 were examined in 1984; 436 men born in 1936 were examined in 1987; 1955 men and 1873 women sampled from 4 birth cohorts (1922, 1932, 1942, 1952) were examined in 1982; 731 men and 739 women (born 1927, 1937, 1947, and 1957) were examined in 1987; and 980 men and 981 women (born in 1922, 1932, 1942, 1952, and 1962) were examined in 1992 | | **Study population:**  ***4,007** out of 4,513 men and **3,533** out of 3,984 women *baseline response rate was 78%  *Follow-up:* *from baseline until Sept. 27, 2000 for death from all causes or until Dec. 31, 1997 for fatal and nonfatal incident CHD **Exclusion:**  *participants with CHD diagnosed during the 5 years prior to enrollment (n=109) *participants with incomplete data  **Attrition:**  - | | | | **Exposures:**  *cardiovascular risk factors assessed through clinical examinations and questionnaires (5 surveys) *dietary variables assessed through questionnaire, whereby participants were asked about frequency of consumption of fish food items, with response options ranging from ‘never’, ‘once a month or less’, ‘twice a month’, ‘once a week’, ‘two to three times a week’, ‘once a day’, ‘two to three times a day’, and ‘four times or more daily’ *questionnaire previously compared with dietary history – adequate in identifying levels of fish intake | | **Outcomes:**  *data on all-cause mortality, CHD mortality, incident CHD obtained from National Board of Healths Register of Cause of Death and the National Patient Register | | **Analysis:**  *Cox proportional hazards regression used to assess the influence of cardiovascular risk factors on fatal and nonfatal CHD outcome, or all-cause mortality *analysis was repeated on a high-risk subgroup of 981 men 50+ years and 622 women 60+ years who were current smokers or current non-smokers, with serum cholesterol over 6-7 mmol/L  **Confounders:**  *familial predisposition, smoking status, physical activity, alcohol, educational status, healthy diet score, total cholesterol, BMI | | **Results:**  *at end of follow-up, death occurred in 826 men and 503 women *among men, the risk of all-cause mortality was lower for those consuming fish one time a month or less compared to those consuming fish once a week (HR=0.80, [0.65, 0.90]) *among males and females combined, the risk of all-cause mortality was lower for those consuming fish two times a month compared to those consuming it once a week (HR=0.84, [0.73, 0.96])  *among males and females combined, as well s the subgroup of high-risk participants, there was a significant linear trend of increasing risk in all-cause mortality with greater intake of fish (trend test p-values=0.02 and 0.03, respectively)   *among males and females combined with serum cholesterol less than 5 mmol/L, CHD risk was lowest for those consuming fish once per week; men with cholesterol levels over 7mmol/L, the lowest CHD risk was identified for those consuming fish once per week *among women with lower educational levels, frequent fish intake was associated with lower risk for CHD  **Limitations:**  *low statistical power *measurement bias due to self-reported dietary intake *residual confounding  **Source of Funding:**  *Danish Medical Research Council | |
| **Authors**: Østbye T, Taylor DH, Jung SH **Year:** 2002 **Citation:** Prev Med. 2002;34(3):334-345 **Country of study:**  **Aim:** To determine the impact of smoking and other modifiable risk factors on ill health in middle-aged and older people **Study design:** Longitudinal **Quality score:** - **Applicability:** | | | | | | | | | | | | | |
| **Source population:**  *at least 12,600 people born in 1931-41 were recruited for Health and Retirement Study (HRS) and interviewed along with their spouses as part of baseline survey in 1992 (wave 1), with follow-up surveys in 1994 (wave 2), 1996 (wave 3), and 1998 (wave 4)  *respondents ages 70 and older and their spouses comprised the baseline sample (wave 1, 1993) were recruited for Asset and Health Dynamics Among the Oldest Old (AHEAD), with follow-up telephone interviews conducted in 1995 (wave 2) and 1998 (wave 3) | | **Study population:**  *HRS: **7,845** people ages 51-61 years  *AHEAD: **5,037** people age 70+ at baseline  **Exclusion:**  HRS participants who: *were outside the age range (n=2,357) *died (n=796) *were lost to follow-up (n=1,958)   AHEAD participants who: *were outside the age range (n=725) *died (n=1,853) *were lost to follow-up (n=607)  **Attrition:**  - | | | | **Exposures:**  *smoking, exercise, and alcohol consumption measured at baseline for HRS and AHEAD *HRS smokers categorized as heavy (at least a pack of cigarettes/day) or light (less than one pack/day), with former smokers categorized as quit < 3 years prior to baseline, quit 3-15 years ago, or more than 15 years ago; AHEAD smokers categorized as current, former, and never smokers *HRS physical activity assessed through questions on 1. participation in light physical activity (3+ times/week, 1-2 times/week, 1-3 times/months, less than once a month, never); and 2. Participation in vigorous physical activity (same categories as for light activity)  >based on these questions, participants were classified into ‘sedentary’ (no exercise), ‘heavy exercise’ (heavy physical activity 3+ times/week), ‘moderate exercise’ (heavy physical activity 1-2 times/week or light physical activity 3+ times/week), ‘light exercise’ (other) groups *alcohol consumption divided into light to moderate drinking (up to 2 drinks/day), heavy drinking (2+ drinks/day), none; self-reported history of drinking problems ascertained | | **Outcomes:**  *ill health outcomes: disability, impaired mobility, self-reported health, and health care utilization, were measured at waves 2-4 for HRS and 2-3 for AHEAD  *Disability:* *disability was defined as having an impairment that limits the amount of paid work that can be accomplished; ADL or impairment in activities necessary for survival (with yes/no answer options for activities assessed), and IADL or impairment in activities necessary to manage in today’s society (with yes/no answer options for activities assessed), were identified  *Impaired mobility:* *ability to walk or climb stairs (with yes/no answer options)  *Self-reported health:* *self-perception of health status measured with answer options of excellent, very good, good, fair or poor  *Health care use:* *proxy for ill health *hospitalization in the past year or admission to nursing home with yes/no options | | **Analysis:**  *data for respondents who completed all waves for each study were entered into multivariate logistic regression models; each 'ill health' outcome (e.g. disability, impaired mobility, etc.) was assessed separately *models with no impairment at baseline were also built   **Confounders:**  *gender, race, marital status, age, education | | **Results:**  *AHEAD (70+ years):* *compared to those who never smoked, current smokers had the highest odds for ill health in terms of IADL dependence (OR=1.46, [1.21, 1.77]), difficulty climbing stairs (OR=1.67, [1.37, 2.03]), difficulty walking (OR=2.06, [1.69, 2.49]), poor health (OR=1.55, [1.29, 1.87]), hospitalization (OR=1.28, [1.08, 1.52]), nursing home placement (OR=1.68, [1.08, 2.63]); further, the odds for ill health were greater for current smokers compared to former smokers *compared to those with BMI 18.5-30, those with BMI 30 or greater had the highest odds for ill health in terms of ADL dependence (OR=1.76, [1.51, 2.06]), IADL dependence (OR=1.23, [1.06, 1.43]), difficulty climbing stairs (OR=2.08, [1.77, 2.46]), difficulty walking (OR=2.27, [1.94, 2.65]), poor health (OR=1.43, [1.24, 1.65]), hospitalization (OR=1.27, [1.12, 1.46]); further, the odds for ill health were generally greater for people with BMI 30 or greater compared to those with BMI less than 18.5 *compared to those who never drink, those with a past drinking problem had the highest odds for ill health in terms of difficulty climbing stairs (OR=1.37, [1.07, 1.75]), hospitalization (OR=1.38, [1.13, 1.68])  *HRS (51-64 at baseline):* *compared to those who never smoked, heavy smokers (one pack or more) and former smokers (quit less than 3 years), had the highest odds for ill health in terms of ADL dependence (OR=1.52, [1.27, 1.82]; OR=1.71, [1.29, 2.26], respectively), disability (OR=2.23, [1.84, 2.71]; OR=2.45, [1.81, 3.33], respectively), difficulty climbing stairs (OR=2.10, [1.86, 2.37]; OR=1.72, [1.41, 2.10], respectively), difficulty walking (OR=2.37, [2.05, 2.74]; OR=2.08, [1.65, 2.62], respectively), poor health (OR=2.06, [1.80, 2.36]; OR=1.99, [1.60, 2.48], respectively), hospitalization (OR=1.41, [1.24, 1.59]; OR=1.46, [1.20, 1.78], respectively); further, the odds for ill health were greater for light smokers (less than one pack), former smokers (quit 3-15 years), former smokers (quite more than 15 years) compared to non-smokers *exercise had a beneficial effect on the odds of ill health  * compared to those with BMI 18.5-30, those with BMI 30 or greater generally had the highest odds for ill health in terms of ADL dependence (OR=1.66, [1.45, 1.89]), disability (OR=1.48, [1.28, 1.72]), difficulty climbing stairs (OR=2.37, [2.16, 2.60]), difficulty walking (OR=2.10, [1.89, 2.34]), poor health (OR=1.70, [1.53, 1.88]), hospitalization (OR=1.38, [1.26, 1.51]); further, the odds for ill health were generally greater for people with BMI less than 18.5 *compared to those who never drink, those with a past drinking problem had the highest odds for ill health in terms of ADL (OR=1.49, [1.20, 1.84]), disability OR=1.43, [1.15, 1.79], difficulty climbing stairs (OR=1.33, [1.13, 1.57]), difficulty walking (OR=1.32, [1.10, 1.60]), poor health (OR=1.29, [1.08, 1.53]), and hospitalization (OR=1.20, [1.02, 1.41])  **Limitations:**  *residual confounding (e.g., detailed data on nutritional and occupational information) *construct overlap – for example, exercise may not be completely distinct from dependent ill health outcomes, such as those reflecting physical function (in AHEAD, ere was some difficulty in identifying adequate controls when measuring exercise levels) **Source of Funding:**  *none reported | |
| **Authors:** Östenson CG, Hilding A, Grill V, Efendic S  **Year:** 2012  **Citation:** Scand J Public Health. 2012;40:730–737  **Country of study:** Sweden  **Aim of study:** Snus use predicts the risk of Type 2 Diabetes incidence  **Study design:** Prospective population-based study  **Quality score: (++, + or -):**  **External validity score: (++, + or -):** | | | | | | | | | | | | | |
| **Number of people**  12,952  **Demographics** Not reported | | **Number of people** 2,383 **Characteristics**  Consistent never snus use N 1,431 Age: mean (95% CI) 47.2 (46.9–47.4) BmI: mean (95% CI) 25.7 (25.5–25.8) Physical activity during leisure time:  % sedentary (95% CI) 10.6 (9.1–12.3) Alcohol consumption:  % highest tertile (95% CI) 28.1 (25.7–30.5 ) Socioeconomic position:  % low (95% CI) 27.0 (24.7–29.4) Current smokersh among snus users/current snus users among smokers: n (% )246 (17.2)  Consistent snus use N 301 Age: mean (95% CI) 44.8 (44.2–45.3) BmI: mean (95% CI) 26.4 (26.0–26.8) Physical activity during leisure time:  % sedentary (95% CI) 9.3 (6.5–13.1) Alcohol consumption:  % highest tertile (95% CI) 47.5 (41.9–53.2) Socioeconomic position:  % low (95% CI) 41.3 (35.8–46.9) Current smokersh among snus users/current snus users among smokers: n (%) 36 (12.0)  former snus use n 213 Age: mean (95% CI) 45.8 (45.2–46.4) BmI: mean (95% CI) 25.8 (25.4–26.2) Physical activity during leisure time:  % sedentary (95% CI) 8.0 (5.0–12.4) Alcohol consumption:  % highest tertile (95% CI) 39.2 (32.9–46.0) Socioeconomic position:  % low (95% CI) 41.8 (35.3–48.6) Current smokersh among snus users/current snus users among smokers: n (%) 16 (7.6)  Consistent never-smoking N 835 Age: mean (95% CI) 46.2 (45.9–46.6) BmI: mean (95% CI) 25.6 (25.4–25.8) Physical activity during leisure time:  % sedentary (95% CI) 9.1 (7.3–11.2) Alcohol consumption:  % highest tertile (95% CI) 21.7 (19.0–24.7) Socioeconomic position:  % low (95% CI) 22.7 (20.0–25.7) Current smokersh among snus users/current snus users among smokers: n (%) 74 (8.9)  Consistent smokinge N 287 Age: mean (95% CI) 46.7 (46.1–47.3) BmI: mean (95% CI) 25.2 (24.8–25.6) Physical activity during leisure time: % sedentary (95% CI) 18.5 (14.5–23.4) Alcohol consumption: % highest tertile (95% CI) 42.0 (36.3–47.9) Socioeconomic position: % low (95% CI) 41.3 (35.8–47.2) Current smokersh among snus users/ current snus users among smokers: n (%) 48 (16.8)  former smoking n 740 Age: mean (95% CI) 46.8 (46.4–47.1) BmI: mean (95% CI) 26.1 (25.8–26.3) Physical activity during leisure time: % sedentary (95% CI) 8.1 (6.4–10.3) Alcohol consumption: % highest tertile (95% CI) 40.9 (37.3–44.5) Socioeconomic position: % low (95% CI) 36.5 (33.1–40.1) Current smokersh among snus users/current snus users among smokers: n (%) 232 (31.4)  **Location**  four municipalities within Stockholm County  **Recruitment strategy** Not reported  **Length of follow-up**  10 years  **Response rate and loss to follow-up** 87% were reinvestigated with anthropometric measurements  **Eligible population** middle-aged Swedish men  **Excluded populations** 246 control subjects and nine subjects with newly diagnosed T2D | | | | **Relevant exposures** Snus use **Time** 1992–94 **Measurement of exposure** Subjects asked if they had ever been daily users of snus, and if so, if they were current daily users  Subjects asked about daily cigarette smoking and categorised into never, former or current smokers | | **Outcomes** Type 2 diabetes  **outcome measurement** oral glucose tolerance test  homeostasis model assessment  **Time**  2002-2004 | | **Analysis strategy** multiple regression analysis  C**onfounders** age, BMI, glucose tolerance at baseline, physical activity, alcohol consumption, socioeconomic position, family history of diabetes and smoking | | **Number** 99  **Effect estimates** Cases: type 2 diabetes; newly diagnosed OR 95% CI Consistent never snus use 1.0 Consistent snus use 1.1 0.6–2.0 Former snus use 0.5 0.2–1.2 Consistent never snus use 1.0 1-5 boxes/week 0.6 0.2–1.4 >5 boxes/week 3.3 1.4–8.1 Consistent never smoking 1.0 Consistent smoking 1.5 0.8–3.0 Former smoking 0.9 0.5–1.7 Consistent never smoking 1.0 1–15 cigarettes/day 0.8 0.3–2.1 >15 cigarettes/day 2.4 1.0–5.8  **Significant trends** men smoking at baseline and still smoking at follow-up had an increased risk of diabetes compared with never smokers  **Reported limitations  Author** small number of cases developing diabetes  **Source of funding** Stockholm County Council, the Swedish Research Council, the Swedish Council for Working Life and Social Research, and Novo Nordisk Scandinavia. | |
| **Authors:** Otani T, Iwasaki M, Yamamoto S, Sobue T, Hanaoka T, et al.  **Year:** 2003  **Citation:** Cancer Epidemiol Biomarkers Prev. 2003;12:1492-1500  **Country of study:** Japan  **Aim of study:** Examine the association of alcohol consumption and cigarette smoking with colorectal cancer  **Study design:** Prospective cohort  **Quality score: (++, + or -):**  **External validity score: (++, + or -):** | | | | | | | | | | | | | |
| **Number of people**  90,004  **Demographics** Not reported | | **Number of people** Not reported **Characteristics**  J - M cells  **Location**  Iwate, Akita, Nagano, Okinawa, and Tokyo (Cohort I)   Ibaraki, Niigata, Kochi, Nagasaki, Okinawa, and Osaka (Cohort II)  **Recruitment strategy** Not reported  **Length of follow-up**  10-year (cohort I)  7-year (cohort II)  **Response rate and loss to follow-up** Loss to follow-up 0.04%  **Eligible population** middle-aged and elderly Japanese men and women  **Excluded populations** non-Japanese (29 men and 20 women), those who had already moved away at baseline (94 men and 57 women), and those outside of the 40–59 age parameters in cohort I (2 women). self- reported medical history of cancer and with a diagnosis of colorectal cancer before the survey began (687 men and 1,363 women); incomplete alcohol and/or smoking items (2,225 men and 1,097 women) | | | | **Relevant exposures** smoking, alcohol consumption, dietary habits, and other lifestyle factors **Time** Cohort I  After January 1, 1990  Cohort II January 1, 1993–1994 **Measurement of exposure** Cohort I average frequency of alcohol consumption reported by : “less than 1 day/month,” “1–3 days/month,” “1–2 days/week,” “3–4 days/week,” “5–6 days/week,” and “everyday.” Subjects consuming alcoholic beverages at least once a week were also asked about types of drinks and average consumption.   Cohort II  asked about drinking status, i.e., never-, ex-, or current drinkers. Ex- and current drinkers provided information on average frequency, types of drinks, and average consumption per day  smoking habits included current and former smoking status, age at initiation of smoking, and average number of cigarettes smoked per day | | **Outcomes** Colorectal Cancer  **outcome measurement** Cases of colorectal cancer were extracted from the JPHC cancer registry based on site codes  mortality data from the Ministry of Health, Labor, and Welfare  **Time**  until the date of diagnosis of colorectal cancer, the date of a subject’s death, the date of moving from a PHC area, or December 31, 1999 | | **Analysis strategy** Cox proportional hazards model  **Confounders** age, family history of colorectal cancer, body mass index, physical exercise, smoking status, alcohol consumption, and PHC area | | **Number** 716  **Effect estimates** N-Q cells  **Significant trends** alcohol consumption and smoking were associated with colorectal cancer in men. regular ethanol consumption was not associated with colorectal cancer in women  **Reported limitations  Author** None reported  **Source of funding** Grant-in-Aid for Cancer Research and for the 2nd Term Comprehensive 10-Year-Strategy for Cancer Control from the Ministry of Health, Labor and Welfare of Japan. | |
| **Authors:** Patel KV, Coppin AK, Manini TM, Lauretani F, Bandinelli S, et al.  **Year:** 2006  **Citation:** Am J Prev Med. 2006;31(3): 217–224  **Country of study:** Italy  **Aim of study:** Test associations of past physical activity levels in midlife with objective measures of mobility in old age  **Study design:** Cohort  **Quality score: (++, + or -):**  **External validity score: (++, + or -):** | | | | | | | | | | | | | |
| **Number of people**  1155  **Demographics** Not reported | | **Number of people** 1001 **Characteristics**  Age, M (SD) 74.8 (7.3) Education, M (SD) 5.4 (3.3) Cigarette smoking  Ever (vs never), % 40.2 Total pack-years among smokers, M (SD) 29.9 (22.0) Body mass index (kg/m2), M (SD) 27.5 (4.1) PHYSICAL ACTIVITY  20–40 years of age, %  Sedentary/minimal 0.7 Light 13.7 Moderate 55.6 Moderate/intense 16.2 Intense/strenuous 13.8 M (SD) 3.3 (0.9)  40–60 years of age, %  Sedentary/minimal 1.5 Light 22.9 Moderate 50.7 Moderate/intense 15.0 Intense/strenuous 10.0 M (SD) 3.1 (0.9)  In the past year, %  Sedentary/minimal 20.9 Light 42.5 Moderate 32.3 Moderate/intense 3.8 Intense/strenuous 0.6 M (SD) 2.2 (0.9)  Lifetime index to age 60, %  Level I 23.6 Level II 51.1 Level III 25.4  Functional outcomes  Short Physical Performance Battery, M (SD) 9.7 (3.3) Unable to walk 400 meters, % 15.0  **Location**  Greve in Chianti and Bagno a Ripoli, Italy  **Recruitment strategy** probability sample  **Length of follow-up**  5-8 years  **Response rate and loss to follow-up** 91.6%  **Eligible population** Not reported  **Excluded populations** | | | | **Relevant exposures** Physical activity and smoking **Time** September 1998 and March 2000 **Measurement of exposure** interviewer administered questionnaire; participants asked to indicate their average level of physical activity during three age periods in life: 20 to 40 years, 40 to 60 years, and the past year  Mini-Mental State Examination was used to measure cognitive impairment | | **Outcomes** Mobility  **outcome measurement** Short Physical Performance Battery   Subjects asked to walk a distance of four meters at their usual pace. The quickest time out of two trials was analyzed.   Subjects were asked to rise from a chair and return to the seated position five times as quickly as possible while keeping their arms folded over their chest  Subjects were asked to walk a standard 400-meter course  **Time**  2005 and 2006 | | **Analysis strategy** Linear and logistic regression models  C**onfounders** age, education, smoking behavior, BMI, total number of medical conditions, Mini-Mental State Examination, nerve conduction velocity, leg muscle power, range of motion of hip and ankle, ankle-brachial index, and serum hemoglobin | | **Number** Not reported  **Effect estimates** Lifetime physical activity to age 60  Short Physical Performance Battery  Men b weight (SE) Level I Reference Level II 0.03 (0.24) Level III 0.40 (0.26) p for trend p = 0.042  Women b weight (SE) Level I Reference Level II 0.27 (0.19) Level III 0.90 (0.27) p for trend p = 0.002  Unable to walk 400 meters Men Odds ratio (95% CI) Level I Reference Level II 0.76 (0.18–3.29) Level III 0.22 (0.04–1.19) p for trend p = 0.047  Women Odds ratio (95% CI) Level I Reference Level II 1.16 (0.40–3.30) Level III 0.52 (0.13–2.08) p for trend p = 0.434  **Significant trends** Older adults who in higher levels of physical activity in midlife were significantly more likely to perform better than individuals who were less physically active  **Reported limitations  Author** Misclassification of previous physical activity  **Reviewer** Participants retrospectively recalled their physical activity levels in midlife  **Source of funding** Intramural Research Program of the National Institutes of Health, National Institute on Aging | |
| **Authors:** Pelkonen M, Tukiainen H, Tervahauta M, Notkola IL, Kivelä SL  **Year:** 2000  **Citation:** Thorax. 2000;55:746–750  **Country of study:** Finland  **Aim of study:** Study the impact of smoking cessation on mortality over range of baseline pulmonary function  **Study design: Cohort**  **Quality score: (++, + or -):**  **External validity score: (++, + or -):** | | | | | | | | | | | | | |
| **Number of people**  Not reported  **Demographics** Not reported | | **Number of people** 1582 **Characteristics**  Not reported  **Location**  Ilomantsi, Pöytyä and Mellilä in Finland  **Recruitment strategy** Not reported  **Length of follow-up**  30 year   **Response rate and loss to follow-up** (97.5% in 1959) and in subsequent re-examinations (90–97.7%)  **Eligible population** Finnish participants in the Seven Countries Study  **Excluded populations** Not reported | | | | **Relevant exposures** Smoking **Time** Re-examinations were performed in 1964, 1969, 1974, 1984, and 1989. **Measurement of exposure** Smoking habits were recorded at the baseline and in subsequent reexaminations by a trained nurse according to a standard questionnaire developed for the Seven Countries Study | | **Outcomes** Mortality  **outcome measurement** Death certificates were collected and causes of death coded  **Time**  1959 and 1989 | | **Analysis strategy** Cox’s proportional hazards regression model  **Confounders** age, BMI, diastolic blood pressure, and total cholesterol and smoking | | **Number** 1086  **Effect estimates** All cause mortality during 1959–89 by tertile of FEV0.75 at baseline Hazard ratio (95% CI) p value  Tertile of FEV0.75 Low 1.56 (1.35 to 1.81) <0.001 Middle 1.08 (0.93 to 1.26) 0.295 High 1  All cause and cause specific mortality during 1964–89 among those who quit between 1959–84 compared with continuous smokers  Hazard ratio (95% CI) p value All cause 0.71 (0.50 to 1.00) 0.049 Cardiovascular disease 0.60 (0.37 to 0.98) 0.043 Cancer 0.58 (0.30 to 1.12) 0.105 Lung cancer 0.50 (0.14 to 1.77) 0.281 Other cancer 0.62 (0.28 to 1.33) 0.220 Respiratory diseases† 2.51 (0.65 to 9.70) 0.181 Other causes 0.91 (0.38 to 2.18) 0.833  **Significant trends** smokers across the entire range of pulmonary function may increase their expectation of lifespan by giving up smoking  **Reported limitations  Author** data set was too small for confident results on mortality from causes other than cardiovascular diseases  **Reviewer** Does not describe sample  **Source of funding** Finnish Academy, the Finnish Lung Health Association, the Finnish Anti-Tuberculosis Association Foundation, and the National Institute on Aging, USA (grant EDC-1 1 RO1 AGO8762-01A1) | |
| **Authors:** Pitsavos C, Panagiotakos DB, Chrysohoou C, Kokkinos P, Menotti A, et al  **Year:** 2004  **Citation:** J Hum Hypertens. 2004;18:495–501  **Country of study:** Greece  **Aim of study:** Investigate the interaction between physical activity and left ventricular hypertrophy on stroke mortality  **Study design:** Cohort  **Quality score: (++, + or -):**  **External validity score: (++, + or -):** | | | | | | | | | | | | | |
| **Number of people**  12 763  **Demographics** Not reported | | **Number of people** 529  **Characteristics**  Presence of LVH  Age at baseline (years) 49 ±6  Current smoking (total) 30 (76%)  <10 cigarettes/day 14 (36%)  10–20 cigarettes/day 8 (20%)  >20 cigarettes/day 8 (20%)  Physical activity  Sedentary life 12 (30%)  Moderate physical activity 12 (30%)  Hard physical activity 16 (40%)  Body mass index (kg/m2) 23 ±4   Absence of LVH Age at baseline (years) 51 ±6  Current smoking (total) 308 (63%)  <10 cigarettes/day 62 (13%) 10–20 cigarettes/day (29%) >20 cigarettes/day 104 (21%) Physical activity  Sedentary life 155 (32%)  Moderate physical activity 185 (38%) Hard physical activity 149 (30%) Body mass index (kg/m2) 23 ±5   P-value Age at baseline (years) 0.156 Current smoking (total) 0.070 Physical activity 0.423 Body mass index (kg/m2) 0.562  **Location**  Corfu, Greece  **Recruitment strategy** Not reported  **Length of follow-up**  40-year  **Response rate and loss to follow-up**  95.3%  **Eligible population** Corfu cohort from the Seven Countries Study  **Excluded populations** | | | | **Relevant exposures**  Physical activity and smoking **Time** Since 1961 periodic visits every 5 years **Measurement of exposure** Physical activity levels were assessed by sel freports of habitual, occupational and leisure-time activities  daily cigarette smoking identified by a positive response on a standardized questionnaire | | **Outcomes** stroke mortality  **outcome measurement** causes of death were obtained from: previous clinical records filled out by the study’s research group, or by hospital records, or by necroscopy records, or by information from family or hospital doctors, other specialists, family or relatives, friends and any other witnesses, or from the police, in case of violent causes or death occurred suddenly in public places or un-witnessed  **Time**  2001 | | **Analysis strategy** Cox proportional hazard model  C**onfounders** age, body mass index, total cholesterol, glucose, smoking and blood pressure levels | | **Number** 74 deaths  **Effect estimates** Estimates from a backward stepwise Cox model predicting 40-year stroke mortality as a function of baseline risk factors, by LVH level HR (95% CI) Presence of LVH  Physical activity status  Sedentary (reference group) 1.00 — Moderate 0.70 (0.50–0.97) Hard 0.75 (0.52–1.09) Age (per 1 year) 1.12 (0.94–1.33) Systolic blood pressure (per 10 mmHg) 1.01 (0.73–1.62) Total serum cholesterol (per 1 mmol/l) 0.86 (0.69–1.07)  Absence of LVH Physical activity status  Sedentary (reference group) 1.00 — Moderate 0.64 (0.45–0.91) Hard 0.72 (0.51–1.02) Age (per 1 year) 1.12 (1.07–1.180 Systolic blood pressure (per 10 mmHg) 1.21 (1.10–1.480 Total serum cholesterol (per 1 mmol/l) 0.80 (0.47–1.22)  **Significant trends** Physical activity was associated with a lower risk of stroke. LVH had 5.8-fold the risk of stroke among sedentary and 4.5-fold the risk among physically active men. Moderate physical activity decreased the risk of stroke by 49% in men with LVH as compared to sedentary without LVH. Hard exercise did not confer any significant reduction in stroke risk  **Reported limitations  Author** mortality data were available from this specific cohort - could not provide predictors for stroke incidence. the imbalance of the sample sizes between men with and without LVH. inability to segregate thrombotic from haemorrhagic strokes. physical activity level of the active group at baseline is likely to decline with advancing age. the physically inactive group at baseline could either stay physically inactive or increase their physical activity level.   **Source of funding** National Heart, Lung and Blood Institute Grants HE 04697, HE 6090 and HE00278 | |
| **Authors:** Preis SR, Stampfer MJ, Spiegelman D, Willett WC, Rimm EB  **Year:** 2010  **Citation:** Am J Clin Nutr 2010;92:1265–72  **Country of study:** USA  **Aim of study:** Examine the association between dietary protein and risk of ischemic heart disease  **Study design:** Prospective study  **Quality score: (++, + or -):**  **External validity score: (++, + or -):** | | | | | | | | | | | | | |
| **Number of people**  51,529  **Demographics** Not reported | | **Number of people** 43,960  **Characteristics**  Q1 Age (y) 53 ± 10 Smoking status [n (%)]  Never 3993 (45.2) Past 3405 (38.6) 1–14 cigarettes/d 262 (3.0) 15–24 cigarettes/d 334 (3.8) 2:25 cigarettes/d 390 (4.4) Unknown no. of cigarettes/d 105 (1.2) Missing 337 (3.8) Exercise (METs) 20.9 ± 30.1 BMI (kg/m2) 25.1 ± 3.2 Calories (kcal/d) 2137 ± 664 Alcohol (% of energy) 5.9 ± 7.4 Alcohol (g/d) 17.5 ± 22.0  Q3 Age (y) 53 ±6 9 Smoking status [n (%)]  Never 4007 (47.5) Past 3336 (39.5) 1–14 cigarettes/d 216 (2.6) 15–24 cigarettes/d 294 (3.5) 2:25 cigarettes/d 214 (2.5) Unknown no. of cigarettes/d 72 (0.9) Missing 301 (3.6) Exercise (METs) 20.8 ± 27.4 BMI (kg/m2) 25.5 ± 3.3 Calories (kcal/d) 2024 ± 597 Alcohol (% of energy) 3.9 ± 4.8 Alcohol (g/d) 10.9 ± 13.5  Q5 Age (y) 55 ± 9 Smoking status [n (%)]  Never 4409 (44.9) Past 4126 (42.1) 1–14 cigarettes/d 251 (2.6) 15–24 cigarettes/d 276 (2.8) 2:25 cigarettes/d 209 (2.1) Unknown no. of cigarettes/d 96 (1.0) Missing 446 (4.5) Exercise (METs) 21.1 ± 29.1 BMI (kg/m2) 25.9 ± 3.6 Calories (kcal/d) 1780 ± 574 Alcohol (% of energy) 2.7 ± 3.6 Alcohol (g/d) 6.8 ± 9.1  P for linear trend Age (y) <0.0001 Smoking status [n (%)]  Never Referent Past 0.009 1–14 cigarettes/d 0.07 15–24 cigarettes/d <0.0001 2:25 cigarettes/d <0.0001 Unknown no. of cigarettes/d 0.14 Missing 0.03 Exercise (METs) 0.89 BMI (kg/m2) <0.0001 Calories (kcal/d) <0.0001 Alcohol (% of energy) <0.0001 Alcohol (g/d) <0.0001  **Location**  Not reported  **Recruitment strategy** Not reported  **Length of follow-up**  18 y  **Response rate and loss to follow-up**  Not reported  **Eligible population** men aged 40–75 y at baseline in 1986  **Excluded populations** those who reported a history of myocardial infarction, angina, coronary artery bypass graft, other heart conditions, stroke, pulmonary embolism, or cancer on the baseline questionnaire. those who had an implausible caloric intake derived from the baseline or had >70 missing responses to food items | | | | **Relevant exposures**  smoking status, diet and physical activity **Time** 1986 and in 1990, 1994, 1998, and 2002 **Measurement of exposure** Every 4 y, participants are sent a food-frequency questionnaire to assess their diet composition | | **Outcomes** nonfatal myocardial infarction and fatal ischemic heart disease  **outcome measurement** Nonfatal MI was assessed biennially with a mailed questionnaire  Deaths ascertained by contact with family members or through the National Death Index. Fatal IHD was confirmed from the medical records or autopsy reports or if IHD was listed as the cause of death on the death certificate and there was evidence of previous IHD in the records  **Time**  1986 and 31 January 2004 | | **Analysis strategy** Cox proportional hazards regression model  C**onfounders** Age and quintiles of percentage of energy from saturated fat, monounsaturated fat, polyunsaturated fat, trans fat, and calories, fibre, folate, vitamin C, magnesium, total omega-3 fatty acids, glycemic index, physical activity, family history of myocardial infarction, BMI, cigarette smoking, alcohol use, and multivitamin use, status of hypertension, hypercholesterolemia and diabetes | | **Number** 2959  **Effect estimates** Relative risks (RRs) and 95% CIs for total ischemic heart disease according to quintile of percentage of energy from protein  Q1 Total protein 1.00 (referent) Animal protein 1.00 (referent) Vegetable protein 1.00 (referent)  Q2 Total protein 1.03 (0.91, 1.16) Animal protein 1.05 (0.93, 1.16) Vegetable protein 0.96 (0.85, 1.08)   Q3 Total protein 1.07 (0.94, 1.21) Animal protein 1.02 (0.90, 1.16) Vegetable protein 0.94 (0.82, 1.08)  Q4 Total protein 1.03 (0.90, 1.16) Animal protein 1.03 (0.90, 1.17) Vegetable protein 0.94 (0.80, 1.09)  Q5 Total protein 1.08 (0.95, 1.23) Animal protein 1.11 (0.97, 1.28) Vegetable protein 0.93 (0.78, 1.12)   P for trend Total protein 0.30 Animal protein 0.18 Vegetable protein 0.49  Relative risks (RRs) and 95% CIs for nonfatal myocardial infarction and fatal ischemic heart disease according to quintile   Q1 Total protein  Non-fatal MI 1.00 (referent) Fatal IHD 1.00 (referent)   Animal protein  Nonfatal MI 1.00 (referent) Fatal IHD 1.00 (referent)   Vegetable protein  Non-fatal MI 1.00 (referent) Fatal IHD 1.00 (referent)   Q2 Total protein  Non-fatal MI 1.01 (0.86, 1.18) Fatal IHD 1.05 (0.86, 1.28)   Animal protein  Nonfatal MI 1.01 (0.86, 1.18) Fatal IHD 1.12 (0.91, 1.37)   Vegetable protein  Non-fatal MI 1.07 (0.91, 1.25) Fatal IHD 0.84 (0.69, 1.02)   Q3 Total protein Non-fatal MI 1.05 (0.90, 1.23) Fatal IHD 1.08 (0.88, 1.33)   Animal protein  Nonfatal MI 0.99 (0.84, 1.16) Fatal IHD 1.08 (0.87, 1.33)   Vegetable protein  Non-fatal MI 1.03 (0.86, 1.24) Fatal IHD 0.84 (0.68, 1.05)   Q4 Total protein  Non-fatal MI 1.02 (0.87, 1.20) Fatal IHD 1.02 (0.83, 1.25)   Animal protein  Nonfatal MI 1.05 (0.89, 1.23) Fatal IHD 0.98 (0.79, 1.21)   Vegetable protein  Non-fatal MI 1.09 (0.89, 1.33) Fatal IHD 0.76 (0.59, 0.96)   Q5 Total protein  Non-fatal MI 1.10 (0.92, 1.30) Fatal IHD 1.05 (0.85, 1.30)   Animal protein  Nonfatal MI 1.12 (0.94, 1.33) Fatal IHD 1.10 (0.88, 1.37)   Vegetable protein  Non-fatal MI 1.18 (0.93, 1.48) Fatal IHD 0.66 (0.49, 0.88)   P for trend Total protein  Non-fatal MI 0.30 Fatal IHD 0.79   Animal protein  Nonfatal MI 0.18 Fatal IHD 0.71   Vegetable protein  Non-fatal MI 0.18 Fatal IHD 0.005  **Significant trends** no association between dietary protein and risk of total IHD in this group of men  **Reported limitations  Author** self-administered questionnaires. study population consisted of white male health professionals  **Source of funding** National Institutes of Health (HL35464 and CA55075) and by the Kirschstein-NRSA Aging Training Grant (AG000158) | |
| **Authors:** Qiao Q, Tervahauta M, Nissinen A, Tuomilehto J  **Year:** 2000  **Citation:** Eur Heart J. 2000;21:1621–1626  **Country of study:** Finland  **Aim of study:** Risk of early and late death in relation to smoking and ex-smoking  **Study design:** Cohort  **Quality score: (++, + or -):**  **External validity score: (++, + or -):** | | | | | | | | | | | | | |
| **Number of people**  1711  **Demographics** Not reported | | **Number of people** 1673  **Characteristics**  Not reported  **Location**  Finland  **Recruitment strategy** Not reported  **Length of follow-up**  35-year  **Response rate and loss to follow-up**  not reported  **Eligible population** men born between 1900 and 1919  **Excluded populations** Subjects known to have coronary heart disease at baseline | | | | **Relevant exposures**  Smoking **Time** 1959 to 1989 **Measurement of exposure** smoking habits was collected using a standardized questionnaire developed for the Seven Countries Study | | **Outcomes** Mortality  **outcome measurement** review of death certificates, collection of medical information and clinical records from hospitals, and interviews with physicians and relatives of the deceased or any other witnesses of fatal events. After the 15th year of follow-up, only the reviewing and coding of official death certificates was performed.  **Time**  end of 1994 | | **Analysis strategy** Cox proportional hazards model  C**onfounders** age, area of residence, body mass index, systolic blood pressure, serum cholesterol at baseline | | **Number** CHD mortality (n = 1463) Total mortality (n = 1673)  **Effect estimates** Smoking status at baseline Total mortality 10-year mortality Non-smoker 1 Ex-smoker 1.74 (1.07–2.83) Current smoker 2.16 (1.44–3.25)  35-year mortality Non-smoker 1 Ex-smoker 1.13 (0.93–1.36) Current smoker 1.62 (1.40–1.88)  CHD mortality 10-year mortality Non-smoker 1 Ex-smoker 7.37 (1.66–32.70) Current smoker 6.80 (1.64–28.22)  35-year mortality Non-smoker 1 Ex-smoker 1.39 (1.00–1.94) Current smoker 1.63 (1.24–2.13)  Baseline cigarettes consumption (number per day) Total mortality 10-year mortality Non-smoker 1 Ex-smoker 1–9 1.94 (1.01–3.74) 10–19 2.05 (1.10–3.80) >20 1.45 (0.77–2.74)  Current smoker 1–9 1.74 (1.02–2.98) 10–19 2.26 (1.45–3.52) >20 2.56 (1.62–4.06)  35-year mortality Non-smoker 1 Ex-smoker 1–9 0.98 (0.72–1.33) 10–19 1.09 (0.82–1.44) >20 1.29 (1.01–1.65)  Current smoker 1–9 1.15 (0.93–1.42) 10–19 1.76 (1.48–2.09) >20 1.98 (1.65–2.37)  CHD mortality 10-year mortality Non-smoker 1 Ex-smoker 1–9 8.18 (1.48–45.09) 10–19 7.32 (1.34–40.08) >20 7.91 (1.59–39.41)  Current smoker 1–9 7.13 (1.51–33.69) 10–19 8.28 (1.92–35.76) >20 7.16 (1.60–31.95)   35-year mortality Non-smoker 1 Ex-smoker 1–9 1.21 (0.73–2.03) 10–19 1.47 (0.93–2.32) >20 1.60 (1.06–2.41)  Current smoker 1–9 1.13 (0.77–1.66) 10–19 1.82 (1.33–2.48) >20 1.96 (1.42–2.72)  Hazard ratios (95% CI) of deaths from all-cause and coronary heart disease in relation to the changes in smoking status between 1959 and 1969 Total mortality  Non-smoker 1 Persistent former smoker 0.97 (0.76–1.22) Smoked in one of three examinations 1.13 (0.87–1.47) Smoked in two of three examinations 1.18 (0.93–1.51) Persistent current smoker 1.84 (1.54–2.19)  CHD mortality  Non-smoker 1 Persistent former smoker 1.12 (0.77–1.65) Smoked in one of three examinations 1.07 (0.67–1.70) Smoked in two of three examinations 1.08 (0.71–1.65) Persistent current smoker 1.84 (1.36–2.48)  **Significant trends** Men smoking persistently were most at risk, while those who persisted in quitting had no increased risk of death compared with non-smokers  **Reported limitations  Author** None reported  **Reviewer** Not reporting demographic characteristics of participants   **Source of funding** Yrjo Jahnsson’s Foundation, Academy of Finland, the Sandoz Foundation for Gerontological Research and the National Institute of Health (AG-08762) | |
| **Authors:** Qiu D, Mei J, Tanihata T, Kawaminami K, Minowa, M  **Year:** 2003  **Citation:** J Epidemiol 2003;13;149-156.  **Country of study:** China  **Aim of study:** Clarify the risk factors of CVD deaths in rural areas  **Study design:** Cohort study  **Quality score: (++, + or -):**  **External validity score: (++, + or -):** | | | | | | | | | | | | | |
| **Number of people**  50,252  **Demographics** Not reported | | **Number of people** 50,069  **Characteristics**  No. of subjects 50252 Age group(%) 40-49 years 41.1  50-59 23.0  60-69 20.8  70-79 12.3  80+ 2.8  Mean age (years) ± SD 55.3 ±11.8 Cigarette smoking status(%)  Non-smoker 57.1  Ex-smoker 3.0  Current smoker 39.9  Alcohol drinking status(%)  Non- drinker 57.2  Ex-drinker 2.0  Current drinker 40.8  Mean body mass index (kg/m') ± SD 20.1 ± 2.5  **Location**  Sixi, Lixi, Luoping, Putian, Luxi, Shinao, Meizhuang, and Ertang Townships in Shanggao, Wuning, Jinxian, and Gaoan Counties in Jiangxi Province, China.  Area(%)  Wuning 39.7  Shanggao 19.7  Gaoan 19.6  Jinxian 21.0   **Recruitment strategy** door-to-door  **Length of follow-up**  6 years  **Response rate and loss to follow-up**  99.6%  **Eligible population** door-to-door  **Excluded populations** 183 cases with a previous history of CVD, 632 CVD deaths. Inpatients were excluded from the study. | | | | **Relevant exposures**  frequency of food intake, liking for fatty foods and salty foods, cigarette smoking, alcohol drinking **Time** September 1, 1994 and June 30, 1996 **Measurement of exposure** door-to-door survey | | **Outcomes** CVD death  **outcome measurement** Village physicians who lived in the same village as cohort subjects filled out follow-up reports and submitted them to the township hospitals every 10 days  **Time**  death from causes other than CVD, or December 31, 2000, whichever came first | | **Analysis strategy** Cox proportional hazard model  C**onfounders** sex, age group, area, cigarette smoking status, alcohol drinking status, blood pressure, BMI, marital status, fatty foods, salty foods, frequency of Chinese pickles intake, frequency of meat intake, sleeping hours per day | | **Number** 671  **Effect estimates** Hazard ratios of cerebrovascular disease mortality HR (95% CI) (Age group) 40-49 years 1.00 50-59 2.14 (1.52- 3.01) 60-69 5.13 (3.79- 6.94) 70-79 11.38 (8.34- 15.54) 80+ 19.85 (13.65- 28.87) p for trend <0.01  (Cigarette smoking status) Non-smoker 1.00 Ex-smoker 1.40 (0.98- 2.00) Current smoker 1.08 (0.87- 1.34)  p for trend 0.59  (Alcohol drinking status) Non-drinker 1.00 Ex-drinker 1.55 (1.04- 2.31)  Current drinker 1.12 (0.93- 1.34) p for trend 0.23  (Blood pressure) Normal 1.00 High-normal 1.38 (1.09- 1.74)  Hypertension 2.06 (1.72- 2.47)  p for trend <0.01  (Body mass index) <18.5 1.00 18.5-23.9 1.12 (0.94- 1.33) >24.0 1.03 (0.68- 1.58) p for trend 0.33  (Marital status) Married 1.00 Never married 1.25 (0.59- 2.65)  Divorced 0.95 (0.42- 2.13)  Widowed 1.16 (0.96-1.41)  p for trend -  (Fatty foods) Dislike 1.00 Normal 1.24 (0.72- 2.15)  Like 1.33 (0.78- 2.29)  p for trend 0.23  (Salty foods) Dislike 1.00 Normal 1.40 (1.13- 1.73)  Like 1.46 (1.10- 1.95)  p for trend <0.01   (Frequency of Chinese pickles intake) Never or seldom 1.00 Once or twice per month 0.91 (0.74- 1.13) More than once per week 0.79 (0.63- 0.98) p for trend 0.03  (Frequency of meat intake) Never or seldom 1.00 Once or twice per month 10.75 (0.62- 0.91)  More than once per week  p for trend 0.13  Sleeping hours per day 6 hours or less 1.00 7 to 8 hours 0.86 (0.68- 1.09) 9 hours or more 1.01 (0.78- 1.31) p for trend 0.65  **Significant trends** CVD mortality significantly increased in parallel with age, blood pressure and salty foods  **Reported limitations  Author** None reported  **Reviewer** misclassification and change of exposure. behaviours are self-reported  **Source of funding** Not reported | |
| **Authors:** Räikkönen K, Matthews KA, Kuller LH  **Year:** 2001  **Citation:** Hypertension. 2001;38:798-802  **Country of study:** USA  **Aim of study:** Test the hypotheses that the trajectory of psychological risk increases the risk for the development of hypertension and that blood pressure levels fluctuate with psychological changes  **Study design:** Began as a prospective study  **Quality score: (++, + or -):**  **External validity score: (++, + or -):** | | | | | | | | | | | | | |
| **Number of people**  Not reported  **Demographics** Not reported | | **Number of people** 541  **Characteristics**  Hypertensives  Mean±SD (coefficient of variation) Age at baseline, y 48.0±1.5  Age at the time of menopause, y 52.6±3.1  BMI, kg/m2 26.8±5.2 (5.6) Physical activity, kJ/wk 4847±5010 (262)  Alcohol consumption, g/d 9.0±12.6 (88.1) Current smoking (yes), n (%) 24 (32)  Cigarettes among smokers, n/d 20.5±11.6 (77.6)  Normotensives Age at baseline, y 47.6±1.6  Age at the time of menopause, y 52.6±2.6  BMI, kg/m2 24.4±4.3 (5.2) Physical activity, kJ/wk 6217±7276 (229) Alcohol consumption, g/d 8.4±10.0 (92.2) Current smoking (yes), n (%) 123 (28.5)  Cigarettes among smokers, n/d 19.3±12.0 (74.9)  **Location**  USA  **Recruitment strategy** driver’s license list  **Length of follow-up**  9.2 years; SD, 3.4 years; range, 1 to 14 years  **Response rate and loss to follow-up**   **Eligible population** middle-aged women  **Excluded populations** | | | | **Relevant exposures**  Physical activity, alcohol use, and cigarette smoking **Time** 1983-1984 **Measurement of exposure** Cigarette smoking defined as the number of cigarettes smoked per day  Alcohol intake defined as the amount of current alcohol intake per day converted into grams of absolute alcohol | | **Outcomes** Hypertensive  **outcome measurement** use of antihypertensive medication and/or had elevated systolic BP or diastolic BP on 2 consecutive exams  **Time**  Not reported | | **Analysis strategy** Cox proportional hazards  **Confounders** age, race, years of education, parental history of hypertension, baseline blood pressure, body mass index, physical activity, alcohol use, and cigarette smoking | | **Number** 75  **Effect estimates** Biological and Health Behaviour Predictors of Hypertension Incidence Predictor, b, P, Hazards Ratio (95% CI) Race (white/African American) 0.16, 0.67, 1.18 (0.57–2.44) Parental history (no/yes) 0.76, 0.005, 2.15 (1.26–3.66) Age at baseline (y) 0.12, 0.14, 1.12 (0.96–1.31) Education (y) -0.04, 0.13, 0.96 (0.78–1.19) BMI (kg/m2) 0.05, 0.04, 1.05 (1.01–1.10) Physical activity (kJ/wk) -0.45, 0.12, 0.64 (0.36–1.13) Alcohol consumption (g/d) 0.84, 0.17, 2.31 (0.71–7.54) Smoking status (no/yes) 0.40, 0.19, 1.50 (0.83–2.72)  **Significant trends** Increasing levels of anger, decreasing levels of social support, and high anxiety increase the likelihood of women’s development of hypertension in midlife  **Reported limitations  Author** bias due to unavailability of follow-up, missing data for BP or for prescription of antihypertensive medication, and psychological changes occurring between the available data points. participants had a different number of evaluations (range, 2 to 8 visits) across the follow-up  **Source of funding** National Institutes of Health grant HL-28266, the Pittsburgh Mind-Body Center (HL-65111 and HL-65112), and the John D. and Catherine T. MacArthur Foundation Research Network on Socioeconomic Status and Health | |
| **Authors**: [Risérus U](http://www.ncbi.nlm.nih.gov/pubmed?term=Ris%C3%A9rus%20U%5BAuthor%5D&cauthor=true&cauthor_uid=17644620), [Arnlöv J](http://www.ncbi.nlm.nih.gov/pubmed?term=Arnl%C3%B6v%20J%5BAuthor%5D&cauthor=true&cauthor_uid=17644620), [Berglund L](http://www.ncbi.nlm.nih.gov/pubmed?term=Berglund%20L%5BAuthor%5D&cauthor=true&cauthor_uid=17644620) **Year:** 2007 **Citation:** [Diabetes Care.](http://www.ncbi.nlm.nih.gov/pubmed/17644620) 2007 Nov;30(11):2928-33  **Country of study:** Sweden **Aim:** To identify predictors of insulin sensitivity  **Study design:** Longitudinal **Quality score: +**  **Applicability: +** | | | | | | | | | | | | | |
| **Source population:**  *2,322 men age 50 years from Uppsala, Sweden were invited and participated in baseline investigation in 1970-73  *1,221 out of 2,322 men (73% of survivors) participated at re-examination in 1991-95 | | **Study population:**  *analysis restricted to **770** men with complete data  *Follow-up:*  *20 yrs (1970-73 to 1991-95)  **Exclusion:**  *124 subjects taking glucose-lowering medication or who had diabetes  *subjects treated with drugs for cardiovascular disease (n=251)  *non-participants (n=460)  *subjects with incomplete data (n=76)  **Attrition:**  *422 men died and 219 moved | | | | **Exposures:**  *self-reported smoking and physical activity  *smoking categorized as smoking versus non-smoking  *physical activity assessed using validated questionnaire and participants categorized into ‘sedentary’, ‘moderate’, ‘regular’, and ‘athletic’ groups | | **Outcomes:**  *insulin sensitivity of men at age 70 was calculated as glucose infusion rate using hyperinsulinemic – euglycemic clamp | | **Analysis:**  *multivariate regression was used to identify independent predictors of insulin sensitivity  **Confounders:**  *****BMI, triglycerides, HDL cholesterol, glucose, blood pressure, physical activity, saturated fat biomarkers, and socioeconomic status, baseline fasting insulin | | **Results:**  *smoking did not significantly predict insulin sensitivity  *physical activity significantly predicted insulin sensitivity (beta=0.25, [0.08, 0.42])  **Limitations:**  *lack of clamp measurements at baseline  *survival bias with unhealthy men (high insulin resistance) more likely to die follow-up  *residual confounding (e.g. abdominal obesity)  *cannot generalize to younger age groups, women, ethnicities other than Caucasians  **Source of funding:**  *The Swedish Society for Medical Research | |
| **Authors**: Ruder EH, Thiébaut AC, Thompson FE, Potischman N, Subar AF, et al.  **Year:** 2011 **Citation:** Am J Clin Nutr. 2011;94(6):1607-1619 **Country of study:** United States **Aim:** To assess the influence of midlife (and adolescent) diet on colorectal cancer risk **Study design:** Longitudinal **Quality score:** ++  **Applicability:** | | | | | | | | | | | | | |
| **Source population:**  *prospective cohort study of men and women ages 50-71 years living in California, Florida, Louisiana, New Jersey, North Carolina, Pennsylvania, Texas, Arizona, Atlanta (GA), Detroit (MI)  *baseline questionnaire completed in 1995-1996 by 617,119 people | | **Study population:**  *cohort for this study included **292,797** participants (171,171 men and 121,626 women) *92.8% and 58.5% of participants were white and male *mean age of 62.8 years at administration of risk factor questionnaire  *Follow-up:* *follow-up from receipt of risk factor questionnaire (1995-1996) until censoring at the end of 2006 or when the participant moved out of the cancer registry areas, had a cancer diagnosis, or died, whichever came first *5% lost to follow-up as a result of moving out of 10 states  **Exclusion:** Excluded people for whom either the baseline [n=6959] or the risk factor questionnaire [n=3424] was completed by proxy respondents, those with prevalent cancer at the administration of the baseline [14,565] or risk factor [n=4297] questionnaires, those who had a death only report for any cancer [n=983], those with 0 PY of follow-up [n=19], and those in the extremes of energy intake for diet in the previous 12 months [n=2334]  **Attrition:**  - | | | | **Exposures:**  *Baseline questionnaire completed in 1995-1996  *6 months later, risk factor questionnaire assessing dietary intake 10 years previously (when participants were around 40-61 years) was administered - comprised 37-item food-frequency questionnaire (FFQ)  *databases used to capture information on energy, carbohydrate, protein, calcium >9 categories of frequency of consumption ranging from ‘never’ to ‘2 or more times per day’ *diet 10 years before baseline: portion size estimated using the median sex-specific portion size from NHANES III; energy and nutrient intakes (carbohydrate, total fat protein, calcium, vitamins A and C) based on NHANES III *exposures assessed: carbohydrate, total fat, protein, fiber, calcium, vitamin A, vitamin C, grains, vegetables, fruit, milk, red meat, processed meat, solid fat, sweet baked goods | | **Outcomes:**  *colorectal cancer (primary diagnoses of adenocarcinoma identified through state cancer registries) | | **Analysis:**  *Cox proportional hazards regression used to estimate the influence of midlife diet on colorectal cancer risk  **Confounders for assessment of intake of energy and nutrients:** *Energy at ages 12-13 years, energy in recent adulthood, nutrient of interest in recent adulthood, age at completion of risk-factor questionnaire, sex, BMI, race, education, physical activity, alcohol consumption, smoking, use of nonsteroidal anti-inflammatory drugs, use of HRT, self-report of a first-degree relative with a history of colon cancer **Confounders for assessment of food group intake:**  *same as above with the addition of use of aspirin and ibuprofen | | **Results:**  *3773/292,797 people had colorectal cancer  *Those in the highest intake category 10 y previously for calcium (HR: 0.83; 95% CI: 0.73, 0.94), vitamin A (HR: 0.81; 95% CI: 0.71, 0.92), vitamin C (HR: 0.83; 95% CI: 0.72, 0.95), fruit (HR: 0.84; 95% CI: 0.73, 0.97), and milk (HR: 0.78; 95% CI: 0.67, 0.90) had a lower risk of colon cancer, but a higher risk of colon cancer was observed for total fat (HR: 1.15 ; 95% CI: 1.01, 1.30), red meat (HR: 1.31; 95% CI: 1.12, 1.53), and processed meat (HR: 1.24; 95% CI: 1.06, 1.45). For rectal cancer, milk was inversely associated (HR: 0.75; 95% CI: 0.58, 0.96) with risk  **Limitations:**  *self-reported data leading to possible misclassification *fiber values from NHANES 1999-2000 possible source of error  **Source of Funding:**  *none reported | |
| **Authors:** [Rusanen M](http://www.ncbi.nlm.nih.gov/pubmed?term=Rusanen%20M%5BAuthor%5D&cauthor=true&cauthor_uid=20975015), [Kivipelto M](http://www.ncbi.nlm.nih.gov/pubmed?term=Kivipelto%20M%5BAuthor%5D&cauthor=true&cauthor_uid=20975015), [Quesenberry CP Jr](http://www.ncbi.nlm.nih.gov/pubmed?term=Quesenberry%20CP%20Jr%5BAuthor%5D&cauthor=true&cauthor_uid=20975015), [Zhou J](http://www.ncbi.nlm.nih.gov/pubmed?term=Zhou%20J%5BAuthor%5D&cauthor=true&cauthor_uid=20975015), [Whitmer RA](http://www.ncbi.nlm.nih.gov/pubmed?term=Whitmer%20RA%5BAuthor%5D&cauthor=true&cauthor_uid=20975015) **Year:** 2011 **Citation:** [Arch Intern Med.](http://www.ncbi.nlm.nih.gov/pubmed/20975015) 2011;171(4):333-339  **Country of study:** US **Aim:** To assess influence of midlife smoking on dementia, AD, and VaD **Study design:** longitudinal **Quality score: +**  **Applicability: +** | | | | | | | | | | | | | |
| **Source population:**  *33,108 members ages 50-60 years of Kaiser Permanente Medical Care Program of Northern California participated in health examination in 1978-85 | | **Study population:**  *analysis restricted to **21,123** surviving participants who were members of health plan in 1994  *mean age in 1994 (at onset of outcome follow-up) of participants was 71.6 years  *Follow-up:*  *for cases, person-years calculated since Jan. 1, 1994  *censoring at dementia diagnosis, date of death, date of end of Kaiser membership, or end of follow-up in July 31, 2008  **Exclusion:**  *people with missing data (n=1045)  *individuals with dementia diagnosis other than outcomes assessed  **Attrition:**  - | | | | **Exposures:**  *self-reported smoking assessed using interview  *participants categorized as never smokers; former smokers; current smokers, which were further categorized into: less than 0.5 packs/day, 0.5-1 pack/day, 1-2 packs/day, 2+ packs/day | | **Outcomes:**  *dementia, Alzheimer’s disease (AD), vascular dementia (VaD)  assessed using electronic health records from Jan. 1, 1994 to July 31, 2008 | | **Analysis:**  *Cox proportional hazards model used to assess influence of midlife smoking on dementia, AD, and VaD  **Confounders:**  *****age, sex, education, race, marital status, midlife BMI, hyperlipidemia, diabetes,  hypertension, heart disease, and stroke during the follow-up, alcohol intake | | **Results:**  *compared to non-smokers, the risk of dementia was higher among those smoking: more than 2 packs per day (HR=2.14, [1.65, 2.78]), 1-2 packs per day (HR=1.44, [1.26, 1.64]), and 0.5-1 packs per day (HR=1.37, [1.23, 1.52])  *there was no association between smoking and AD risk  *those smoking more than 2 packs per day in midlife were almost 3 times (HR=2.72, [1.20, 6.18]) more likely to develop VaD later in life than the non-smoking individuals  **Limitations:**  *possible outcome misclassification (e.g. of dementia diagnosis)  *AD or VaD cases may have been missed in those who died prior to 1994 resulting in selective survival effect  *influence of smoking cessation on dementia risk was not assessed  **Source of funding:**  *****The National Graduate School of Clinical Investigation, EVO grants from Kuopio University Hospital, and grants from  the Juho Vainio Foundation and Maire Taponen Foundation; Kaiser Permanente Community Benefits Grant and National Institute of Health and Academy of Finland | |
| **Authors**: [Sabia S](http://www.ncbi.nlm.nih.gov/pubmed?term=Sabia%20S%5BAuthor%5D&cauthor=true&cauthor_uid=20840170), [Guéguen A](http://www.ncbi.nlm.nih.gov/pubmed?term=Gu%C3%A9guen%20A%5BAuthor%5D&cauthor=true&cauthor_uid=20840170), [Berr C](http://www.ncbi.nlm.nih.gov/pubmed?term=Berr%20C%5BAuthor%5D&cauthor=true&cauthor_uid=20840170), [Berkman L](http://www.ncbi.nlm.nih.gov/pubmed?term=Berkman%20L%5BAuthor%5D&cauthor=true&cauthor_uid=20840170), [Ankri J](http://www.ncbi.nlm.nih.gov/pubmed?term=Ankri%20J%5BAuthor%5D&cauthor=true&cauthor_uid=20840170), et al. **Year:** 2011 **Citation:** [Addiction.](http://www.ncbi.nlm.nih.gov/pubmed/20840170) 2011;106(1):93-101  **Country of study:** France **Aim:** To assess association between alcohol intake and cognitive function by occupational position **Study design:** Longitudinal **Quality score: +**  **Applicability: ++** | | | | | | | | | | | | | |
| **Source population:**  *cohort of 20,625 employees ages 40-50 years for men and 35-50 years for women of France’s national electricity and gas company established in 1989  *in 2002-04, 10,537 participants (9,399 men) over the age of 55 years were invited and were eligible for cognitive testing | | **Study population:**  *analysis restricted to **4,073** out of 4,525 men (denominator represents 48.2% of target population) who underwent cognitive testing and had complete data  *Follow-up:*  *approximately 10 years  **Exclusion:**  *participants with incomplete data (n=452)  **Attrition:**  - | | | | **Exposures:**  *self-reported alcohol intake assessed annually using validated questionnaire  *frequency and daily consumption of alcoholic beverages in the week preceding questionnaire administration were used to calculate units of alcoholic drinks consumed in a week  (with 1 unit=10-12 g alcohol)  *mean alcohol consumption and change in alcohol intake over 10 years prior to cognitive testing assessed *participants were classified into the following drinking categories: no alcohol consumption, 1–3 drinks/week (occasional drinkers), 4–14 drinks/week (light drinkers), 15–21 drinks/week (moderate drinkers), and more than 21 drinks/week (heavy drinkers)  *alcohol data was extracted from 1992-2001 for those who underwent cognitive screening in 2002, from 1993-2002 for those screened in 2003, and from 1994-2003 for those screened in 2004 | | **Outcomes:**  *cognitive performance assessed in 2002-04 using Digit Symbol Substitution Test (DSST), which measures psychomotor speed, sustained attention and logical reasoning | | **Analysis:**  *ANCOVA used to assess association between alcohol intake and cognitive performance by occupational position  **Confounders:**  *****age, screening centre, marital status, and smoking history | | **Results:**  *among those with low occupational position, participants consuming more than 21 drinks/week had a mean score on the DSST that was 2.1 points lower (95% CI: −3.9, −0.3) than those consuming 4–14 drinks/week  *associations between DSST and alcohol consumption were observed only in the lower educational group  *Compared to stable alcohol  consumption, great increase and decrease in alcohol consumption were both associated with lower DSST score only in the low occupational group (−3.9 points (95% CI: −6.1, −1.7)  and −3.5 points (95% CI: −6.2, −0.7) respectively for high increase and decrease compared to stable alcohol consumption); *p* for interaction=0.003  **Limitations:**  *unable to distinguish regular alcohol intake from binge drinking  *possible bias due to missing data  *cannot generalize to women and unemployed people  **Source of funding:**  *European Science Foundation, National Institute on Aging | |
| **Authors**: [Sabia S](http://www.ncbi.nlm.nih.gov/pubmed?term=Sabia%20S%5BAuthor%5D&cauthor=true&cauthor_uid=19574344), [Nabi H](http://www.ncbi.nlm.nih.gov/pubmed?term=Nabi%20H%5BAuthor%5D&cauthor=true&cauthor_uid=19574344), [Kivimaki M](http://www.ncbi.nlm.nih.gov/pubmed?term=Kivimaki%20M%5BAuthor%5D&cauthor=true&cauthor_uid=19574344), [Shipley MJ](http://www.ncbi.nlm.nih.gov/pubmed?term=Shipley%20MJ%5BAuthor%5D&cauthor=true&cauthor_uid=19574344), [Marmot MG](http://www.ncbi.nlm.nih.gov/pubmed?term=Marmot%20MG%5BAuthor%5D&cauthor=true&cauthor_uid=19574344), [et](http://www.ncbi.nlm.nih.gov/pubmed?term=Singh-Manoux%20A%5BAuthor%5D&cauthor=true&cauthor_uid=19574344) al. **Year:** 2009 **Citation:** [Am J Epidemiol.](http://www.ncbi.nlm.nih.gov/pubmed/19574344) 2009;15;170(4):428-437  **Country of study:** England **Aim:** To assess the influence of unhealthy or high risk behaviours on poor executive function and memory **Study design:** Longitudinal **Quality score: +**  **Applicability: ++** | | | | | | | | | | | | | |
| **Source population:**  *10,308 London-based office staff, ages 35-55 years initially examined in 1985-88 | | **Study population:**  *analysis restricted to **5,123** participants  *****mean age at baseline in 1997-99 was 56 years  Follow-up:  *1997-99 to 02-04  Exclusion:  **-**  **Attrition:**  *during the 17-year follow-up, 50% of original cohort lost to follow-up | | | | **Exposures:**  *Self-reported health behaviours at baseline (alcohol, smoking, physical activity, diet):*  *self-reported alcohol units in last 7 days, with 1unit=8 g ethanol; intake categorized as no alcohol, 1–14 units, and  15 or more units of alcohol per week (‘high risk’ behaviour defined as abstinence from alcohol)  *self-reported smoking (‘high risk’ behaviour defined as current smoker)  *self-reported frequency and duration of physical activity used to compute hours per week of activity intensity level (‘low risk’ behaviour defined as more than 2.5 hours per week of moderate physical activity or more than 1 hour per week of vigorous physical activity)  *self-reported dietary behaviour including frequency of fruit and vegetable intake (‘low risk’ behaviour defined as eating fruits and vegetables 2 or more times per day) | | **Outcomes:**  *****cognitive function tests used to measure executive function (reasoning, verbal fluency measures) during clinical examination in 2002-04 | | **Analysis:**  *logistic regression to assess the influence of unhealthy or high risk behaviours on poor executive function and memory  **Confounders:**  *age, sex, socio-economic status | | **Results:**  *the odds for poor executive function were higher among current smokers compared to non-smokers (OR=1.30, [1.01, 1.67]), non-drinkers compared to those who consumed 1-14 units/week (OR=1.71, [1.39, 2.10]), those with high levels of physical activity compared to low levels (OR=1.19, [1.01, 1.39]), those who consumed fruits and vegetables >=2 times per day compared to those who consumed less than this amount (OR=1.60, [1.36, 1.89])  *the odds for poor memory were higher among non-drinkers compared to those who consumed 1-14 units/week (OR=1.34, [1.08, 1.66]), those who consumed fruits and vegetables >=2 times per day compared to those who consumed less than this amount (OR=1.35, [1.14, 1.59])  *the odds of poor executive function were higher among those with all four unhealthy behaviours (in terms of diet, physical activity, alcohol, smoking) compared to those with no unhealthy behaviours (OR=5.12, [2.46, 10.70])  **Limitations:**  *limited generalizability  *high loss to follow-up  *frequency, and not amount, of fruit and vegetable intake assessed  **Source of funding:**  *****British Medical Research  Council; the British Heart Foundation; the British Health and Safety Executive; the British Department of Health;  the National Heart, Lung, and Blood Institute; the National Institute on Aging; the Agency for Health Care Policy and Research; and the John D. and Catherine T. MacArthur Foundation Research Networks on Successful Midlife  Development and Socioeconomic Status and Health | |
| **Authors** [Sabia](http://www.ncbi.nlm.nih.gov/pubmed/?term=Sabia%20S%5Bauth%5D) S, Marmot M, [Dufouil](http://www.ncbi.nlm.nih.gov/pubmed/?term=Dufouil%20C%5Bauth%5D) C, [Singh-Manoux](http://www.ncbi.nlm.nih.gov/pubmed/?term=Singh-Manoux%20A%5Bauth%5D) A **Year:** 2008 **Citation:** Arch Intern Med. 2008;168(11):1165–1173  **Country of study:** England **Aim:** Used to assess the association between smoking status and cognitive function **Study design:** Longitudinal **Quality score: +**  **Applicability: ++** | | | | | | | | | | | | | |
| **Source population:**  *10,308 London-based civil servants (6895 men and 3413 women) ages 35-55 years recruited in 1985  *73% response rate  *baseline examination undertaken in 1985-88 (phase 1); subsequent phases of data collection undertaken (e.g., phase 5 in 1997-99; phase 7 in 2002-04 | | **Study population:**  *analysis restricted to **5,388** participants ages 35-55 years  *Follow-up:*  *1985-88 to 97-99  *mean of 17.1 years of follow-up  **Exclusion:**  *non-responders (n=2204)  **Attrition:**  *individuals who had died (n=274) | | | | **Exposures:**  *self-reported smoking status, age at smoking initiation or stopping if ex-smoker, and mean number of cigarettes/cigars, ounces of tobacco smoked per week assessed using questionnaire  *smoking categories created: never smoker (those who never smoked), current smoker at phase 5, long-term ex-smoker (those who stopped before phase 1), and recent ex-smoker (stopped smoking between phase 1 and phase 5)  *grams of tobacco smoked per day (1 cigarette=1g and 1 cigar-3g) used to calculate smoking pack-years | | **Outcomes:**  *cognitive function at phase 7 assessed (memory, reasoning, vocabulary, semantic and phonemic fluency) using battery of tests: 20-word free recall test, Alice Heim AH4 Group Test of General Intelligence, Mill Hill Vocabulary Test, verbal fluency tests | | **Analysis:**  *Cox proportional hazards regression models used to assess the association between smoking status at phase 1 and cognitive function at phases 5 and 7  **Confounders:**  *****age, sex, marital status,  education, socioeconomic position, alcohol use, frequency of fruit and vegetable intake, physical activity | | **Results:**  *odds ratio of being in the lowest quintile of change in cognitive function between phase 5 (1997-99) and phase 7 (2002-04):*  *compared to never smokers, current smokers (OR=1.40, [1.11, 1.75]) and recent ex-smokers (OR=1.38, [1.07, 1.77]) were more likely to show a decline in reasoning  *odds ratio of being in the lowest Quintile of Cognitive Function at phase 5 as a function of smoking status (1997-1999):*  *compared to never smokers, current smokers were more likely to show a decline in memory (OR=1.37, [1.10, 1.73])  *compared to never smokers, recent ex-smokers were less likely to show a decline in vocabulary, phonemic fluency, and semantic fluency (OR=0.73, [0.60, 0.87]; OR=0.73, [0.61, 0.87]; and OR=0.75, [0.63, 0.89], respectively); similar findings were reported for recent ex-smokers  **Limitations:**  *****self-reported smoking habits (social desirability)  *limited generalizability due to homogeneity of cohort  *useful to include additional study phases to better study intraindividual change  **Source of funding:**  *****British Medical Research Council; the British Heart Foundation; the British Health and Safety Executive; the British Department of Health; the National Heart, Lung, and Blood Institute; the National Institute on Aging; the Agency for Health Care Policy and Research; and by the John D. and Catherine T.  MacArthur Foundation Research Networks on Successful Midlife Development and Socioeconomic Status and  Health | |
| **Authors**: [Sairenchi T](http://www.ncbi.nlm.nih.gov/pubmed?term=Sairenchi%20T%5BAuthor%5D&cauthor=true&cauthor_uid=15234937), [Iso H](http://www.ncbi.nlm.nih.gov/pubmed?term=Iso%20H%5BAuthor%5D&cauthor=true&cauthor_uid=15234937), [Nishimura A](http://www.ncbi.nlm.nih.gov/pubmed?term=Nishimura%20A%5BAuthor%5D&cauthor=true&cauthor_uid=15234937), [Hosoda T](http://www.ncbi.nlm.nih.gov/pubmed?term=Hosoda%20T%5BAuthor%5D&cauthor=true&cauthor_uid=15234937), [Irie F](http://www.ncbi.nlm.nih.gov/pubmed?term=Irie%20F%5BAuthor%5D&cauthor=true&cauthor_uid=15234937), et al. **Year:** 2004 **Citation:** [Am J Epidemiol.](http://www.ncbi.nlm.nih.gov/pubmed/15234937) 2004;15;160(2):158-162  **Country of study:** Japan **Aim:** To assess influence of smoking on the development of type 2 diabetes mellitus **Study design:** Longitudinal **Quality score: +**  **Applicability: +** | | | | | | | | | | | | | |
| **Source population:**  *192,125 Japanese individuals (63,379 men and 128,746 women) ages 40-79 years underwent health check-ups in 1993 | | **Study population:**  *analysis restricted to **39,528** men and **88,613** women  *Follow-up:*  *1994 to 2002  *annual follow-up examinations until diagnosis of diabetes mellitus or end of 2002  **Exclusion:**  *3,614 men and 3,645 women with fasting plasma glucose greater than 7 mmol/L or a nonfasting plasma flucose level greater than 11.1 mmol/L  *1,333 men and 1,646 women with history of diabetes mellitus at baseline  *non-participants of 1994 survey (18,904 men and 34,842 women)  **Attrition:**  *28% of participants lost to follow-up | | | | **Exposures:**  *self-reported never smokers, ex-smokers, current smokers of <20 cigarettes/day, or current smokers of >=20 cigarettes/day | | **Outcomes:**  *incident type 2 diabetes diagnosed when fasting plasma glucose level was greater than 7 mmol/L or a nonfasting plasma glucose level of greater than 11.1 mmol/L and/or person had begun to receive diabetes treatment | | **Analysis:**  *Cox proportional hazards regression was used to assess influence of smoking on the development of type 2 diabetes mellitus  **Confounders:**  *****age, alcohol intake, body mass index, systolic  blood pressure, antihypertensive medication use, fasting status, plasma glucose level, serum total cholesterol level, high density lipoprotein cholesterol level, and log-transformed triglyceride level | | **Results:**  *3,704 incident cases of type 2 diabetes mellitus among men and 4,286 cases among  women  *Men:*  *among those 40-59 years of age, current smokers had greater risk of type 2 diabetes compared to never smokers (RR=1.37, [1.18, 1.60])  *among those 60-79 years, current smokers had higher risk for type 2 diabetes compared to never smokers (RR=1.20, [1.08, 1.34])  *Women:*  *****among those 40-59 years of age, current smokers had greater risk of type 2 diabetes compared to never smokers (RR=1.45, [1.18, 1.79])  *among those 60-79 years, current smokers had higher risk for type 2 diabetes compared to never smokers (RR=1.34, [1.09, 1.66])  **Limitations:**  *incomplete follow-up  *residual confounding by physical activity and dietary habits  **Source of funding:**  *none reported | |
| **Authors**: [Stevens RJ](http://www.ncbi.nlm.nih.gov/pubmed?term=Stevens%20RJ%5BAuthor%5D&cauthor=true&cauthor_uid=19165860), [Roddam AW](http://www.ncbi.nlm.nih.gov/pubmed?term=Roddam%20AW%5BAuthor%5D&cauthor=true&cauthor_uid=19165860), [Spencer EA](http://www.ncbi.nlm.nih.gov/pubmed?term=Spencer%20EA%5BAuthor%5D&cauthor=true&cauthor_uid=19165860), [Pirie KL](http://www.ncbi.nlm.nih.gov/pubmed?term=Pirie%20KL%5BAuthor%5D&cauthor=true&cauthor_uid=19165860), [Reeves GK](http://www.ncbi.nlm.nih.gov/pubmed?term=Reeves%20GK%5BAuthor%5D&cauthor=true&cauthor_uid=19165860), et al. **Year:** 2009 **Citation:** [Int J Cancer.](http://www.ncbi.nlm.nih.gov/pubmed/19165860) 2009;15;124(10):2400-2405  **Country of study:** England and Scotland **Aim:** To assess influence of risk factors on incident or fatal pancreatic cancer **Study design:** Longitudinal **Quality score: +**  **Applicability: ++** | | | | | | | | | | | | | |
| **Source population:**  *13 million middle-aged women invited for breast cancer screening in England and Scotland were recruited for study in 1996-01 | | **Study population:**  ***1.29** million women  *Follow-up:*  *96-01 to 2005-07  *mean yrs of follow-up: 7.2 for cancer incidence; 8.9 for cancer mortality  *Incidence of pancreatic cancer: participants followed from date of recruitment to date of pancreatic cancer diagnosis, date of death, or last date of follow-up, whichever came first  *Pancreatic cancer mortality: follow-up from recruitment until death from pancreatic cancer, death from other cause, or study end  *Date of follow-up for cancer incidence was Dec. 31 2002- Dec. 31 2006, and for mortality Dec. 31, 2007  **Exclusion:**  *women with cancer other than non-melanoma skin cancer before recruitment  **Attrition:**  - | | | | **Exposures:**  *self-reported smoking (never, former, current categories), alcohol consumption in units/week, strenuous exercise (enough to cause sweating/rapid heartbeat), any exercise | | **Outcomes:**  *incident and fatal pancreatic cancer identified through National Health Service Central Register (deaths and cancer registrations) | | **Analysis:**  *Cox proportional hazards model used to assess influence of risk factors on incident or fatal pancreatic cancer  **Confounders:**  *****age, region, socioeconomic status, smoking, body mass index and height | | **Results:**  *****current smokers had a high risk of incident pancreatic cancer compared to never  smokers (RR 2.39, 95% CI 2.10–2.73)  *the incidence of pancreatic cancer increased with increasing BMI; the risk of incidence among obese women (BMI>= 30.0 kg/m2) was higher compared to women with BMI 22.5–25.0 kg/m2 (RR=1.34, [CI 1.13–1.57])  **Limitations:**  *self-reported exposure measurements  **Source of funding:**  *none reported | |
| **Authors**: [Sun Q](http://www.ncbi.nlm.nih.gov/pubmed?term=Sun%20Q%5BAuthor%5D&cauthor=true&cauthor_uid=21909248), [Townsend MK](http://www.ncbi.nlm.nih.gov/pubmed?term=Townsend%20MK%5BAuthor%5D&cauthor=true&cauthor_uid=21909248), [Okereke OI](http://www.ncbi.nlm.nih.gov/pubmed?term=Okereke%20OI%5BAuthor%5D&cauthor=true&cauthor_uid=21909248), [Rimm EB](http://www.ncbi.nlm.nih.gov/pubmed?term=Rimm%20EB%5BAuthor%5D&cauthor=true&cauthor_uid=21909248), [Hu FB](http://www.ncbi.nlm.nih.gov/pubmed?term=Hu%20FB%5BAuthor%5D&cauthor=true&cauthor_uid=21909248), et al. **Year:** 2011 **Citation:** [PLoS Med.](http://www.ncbi.nlm.nih.gov/pubmed/21909248) 2011;8(9):e1001090  **Country of study:** US **Aim:** To assess the influence of alcohol intake on the odds of successful aging **Study design:** Longitudinal **Quality score: ++**  **Applicability: +** | | | | | | | | | | | | | |
| **Source population:**  *121,700 female registered nurses recruited in 1976 for Nurses’ Health Study and followed every 2 years thereafter | | **Study population:**  *analysis restricted to **13,894** participants who survived to 70+ years of age through to year 2000  *Sociodemographics:*  *median age of 58 years at midlife for participants  *Follow-up:*  *1980-84 through to 2000  *biennial follow-up rate ranged from 99.2% in 1986 to 94.6% in 2000  **Exclusion:**  *heavy drinkers (>45g/day) at midlife (n=268)  *those diagnosed with chronic conditions included in the successful aging definition prior to baseline in 1984 (n=2,196)  *those with insufficient/ missing data (n=2253)  *individuals reporting previous diagnosis of alcohol dependence or chronic liver disease (n=130) or with past alcohol problems (n=674)  **Attrition:**  - | | | | **Exposures:**  *midlife alcohol use was identified using averaged reports from the 1980 and 1984 validated food frequency questionnaires (FFQ)  *self-reported average total alcohol intake in grams per day over a year was assessed with response options ranging from  ‘almost never’ to ‘6+ servings per day’  *alcohol consumption based on frequency of alcohol intake and alcohol content in beverages (e.g., 13.2 g alcohol for 1 bottle of beer, 10.8 g for 1 glass of wine, 15.1 g for 1 drink of liquor)  *high level of reproducibility and validity of alcohol measurement on the FFQ  *correlation coefficient between FFQ and diet records ranged from 0.84-0.90  *to assess drinking patterns of alcohol, 1986 FFQ was used in determining the number of drinking days/week | | **Outcomes:**  *successful aging defined as being free of 11 major chronic diseases and having no major cognitive impairment, physical impairment, or mental health limitations, and survival until at least age 70 years  *outcomes assessed using medical record review, pathology report review,  telephone interview, questionnaires  *Telephone Interview for Cognitive Status (TICS) used to assess cognitive function (1995-2001); TICS has high reliability (r=0.97) and validity  *SF-36 used to assess physical function and mental health | | **Analysis:**  *logistic regression used to assess the influence of alcohol intake on the odds of successful aging  **Confounders:**  *****age at baseline; body mass index; physical activity; smoking status; education; husband’s  education; marital status; postmenopausal hormone use; family history of heart disease; family history of diabetes; family history of cancer; history of hypertension; history of high cholesterol; use of aspirin; and intakes of fruits and vegetables, whole grains, fish, and red meat | | **Results:**  *1,491 participants aged successfully  *the odds of successful aging to age 70+ were higher among those consuming 5.1-15.0 g/day and 15.1-30.0 g/day alcohol compared to nondrinkers (OR=1.19, [1.01, 1.40], and OR=1.28, [1.03, 1.58], respectively)  *similar findings were observed when drinking pattern at midlife in 1986 was assessed in relation to odds of successful aging (e.g., higher odds of successful aging with greater alcohol use)  **Limitations:**  *cannot generalize to other ethnic groups (sample consisted predominantly of participants of European ancestry) and men  *high-risk participants may have died early (selection bias)  *residual confounding  **Source of funding:**  *none reported | |
| **Authors**: [Tabak C](http://www.ncbi.nlm.nih.gov/pubmed?term=Tabak%20C%5BAuthor%5D&cauthor=true&cauthor_uid=11246587), [Smit HA](http://www.ncbi.nlm.nih.gov/pubmed?term=Smit%20HA%5BAuthor%5D&cauthor=true&cauthor_uid=11246587), [Räsänen L](http://www.ncbi.nlm.nih.gov/pubmed?term=R%C3%A4s%C3%A4nen%20L%5BAuthor%5D&cauthor=true&cauthor_uid=11246587), [Fidanza F](http://www.ncbi.nlm.nih.gov/pubmed?term=Fidanza%20F%5BAuthor%5D&cauthor=true&cauthor_uid=11246587), [Menotti A](http://www.ncbi.nlm.nih.gov/pubmed?term=Menotti%20A%5BAuthor%5D&cauthor=true&cauthor_uid=11246587), et al **Year:** 2001 **Citation:** [Epidemiology.](http://www.ncbi.nlm.nih.gov/pubmed/11246587) 200;12(2):239-245  **Country of study:** Finland, Italy, Netherlands **Aim:** Used to assess association between alcohol consumption and 20-year COPD mortality **Study design:** Longitudinal **Quality score: -**  **Applicability: +** | | | | | | | | | | | | | |
| **Source population:**  *samples of men ages 40-59 years recruited in 1960 from seven countries and with vital status information during 30 years of follow-up | | **Study population:**  *analysis restricted to Finnish, Italian, Dutch cohorts comprising **2,953** men  *Follow-up:*  *approximately 20 years from 1965-70 to 1990  **Exclusion:**  **-**  **Attrition:**  *1,729 men died over 20 years of follow-up | | | | **Exposures:**  *alcohol consumption in 1965-1970 estimated using cross-check dietary history method  *participants categorized into: none, <=1 drink per week (occasional), >1 per week and <=3 per day (light), >3 and <=9 per day, and >9 per day (with 1 drink = 10 grams alcohol) | | **Outcomes:**  *chronic obstructive pulmonary disease (COPD) mortality assessed between 1970 and 1990 using clinical records, family doctors, specialists, relatives, other sources | | **Analysis:**  *Cox proportional hazards model used to assess association between baseline alcohol consumption and 20-year COPD mortality  **Confounders:**  *****age, BMI, energy intake, cigarette smoking, and  country | | **Results:**  *73 men died from COPD during follow-up  *U-shaped relation between alcohol use and COPD mortality  **Limitations:**  **-**  **Source of funding:**  *none reported | |
| **Authors**: [Virtaa JJ](http://www.ncbi.nlm.nih.gov/pubmed?term=Virtaa%20JJ%5BAuthor%5D&cauthor=true&cauthor_uid=20858964), [Järvenpää T](http://www.ncbi.nlm.nih.gov/pubmed?term=J%C3%A4rvenp%C3%A4%C3%A4%20T%5BAuthor%5D&cauthor=true&cauthor_uid=20858964), [Heikkilä K](http://www.ncbi.nlm.nih.gov/pubmed?term=Heikkil%C3%A4%20K%5BAuthor%5D&cauthor=true&cauthor_uid=20858964), [Perola M](http://www.ncbi.nlm.nih.gov/pubmed?term=Perola%20M%5BAuthor%5D&cauthor=true&cauthor_uid=20858964), [Koskenvuo M](http://www.ncbi.nlm.nih.gov/pubmed?term=Koskenvuo%20M%5BAuthor%5D&cauthor=true&cauthor_uid=20858964), et al. **Year:** 2010 **Citation:** [J Alzheimers Dis.](http://www.ncbi.nlm.nih.gov/pubmed/20858964) 2010;22(3):939-48  **Country of study:** Finland **Aim:** to assess the influence of alcohol intake on cognitive impairment **Study design:** longitudinal **Quality score: ++**  **Applicability: ++** | | | | | | | | | | | | | |
| **Source population:**  *3,310 individuals (same-sexed twin pairs) born in Finland before 1937 with both co-twins alive in 1967 contacted  *baseline questionnaires administered in 1975 and 1981 (response rates: 89% and 84%, resp.) | | **Study population:**  *analysis restricted to **1,486** Finnish participants comprised of same-sexed monozygotic or dizygotic twins  *Mean age in 1981: 51.7 years  *Follow-up:*  1975-81 to 1999-07 (mean follow-up: 22.8 years)  **Exclusion:**  *lack of data (n=534)  *those who died (n=21)  *participants with incomplete interview data or missing information (n=378)  *insufficient data captured at both interviews (n=1)  *daily alcohol intake outlier (n=1)  *those with mild cognitive impairment at baseline  **Attrition:**  *inadequate self-reported alcohol data mostly for women with lower levels of education | | | | **Exposures:**  *self-reported total weekly (for beer, wine) or monthly (for spirits) alcohol  intake  *1 drink=12 g ethanol  *participants categorized as abstainers, light drinkers (alcohol intake > 0 and <=  3 drinks /week), moderate drinkers (> 3 and <= 7 drinks for women, > 3 and <= 14 drinks /week for  men), and heavy drinkers (> 7 drinks for women, > 14 for men)  *number of pass-outs and at least monthly binge drinking assessed at baseline (1975-81) | | **Outcomes:**  *cognitive function assessed using TELE, a self-report  telephone interview  *monozygotic and dizygotic twins ages 65 years and older interviewed in 1999-01 and 2003-07 | | **Analysis:**  *logistic regression used to assess the influence of alcohol intake on cognitive impairment  **Confounders:**  *****gender, age, educational level | | **Results:**  *the odds for cognitive impairment were higher among abstainers and heavy drinkers compared to light drinkers (OR=1.51, [1.04, 2.18]; and 2.03, [1.17, 3.54], respectively)  *the odds for cognitive impairment were higher among binge drinkers compared to non-binge drinkers (odds ratios ranged from OR=1.87, [1.04, 3.38] to OR=1.98, [1.05, 3.72])  *the odds for cognitive impairment were higher among those who passed out more than 2 times due to excess drinking compared to those who did not report pass-outs (OR=4.10, [1.54, 10.94])  *among APOE 4 carriers: abstainers and heavy drinkers had higher odds for cognitive impairment compared to light drinkers (OR=1.96, [1.12, 3.41], OR=4.08, [1.85, 9.00], respectively)  **Limitations:**  *social desirability bias associated with self-reported alcohol intake  *telephone interview to assess cognitive function is less specific and sensitive than clinical evaluation  **Source of funding:**  *the Academy of Finland, the Sigrid Juselius Foundation and Clinical grants of Turku University Hospital (EVO) | |
| **Authors**: Waki K, Noda M, Sasaki S, Matsumura Y, Takahashi Y, et al. **Year:** 2005 **Citation:** Diabet Med. 2005;22(3):323-331 **Country of study:** Japan **Aim:** To assess the influence of alcohol consumption and other risk factors on self-reported diabetes among middle-aged Japanese people **Study design:** Longitudinal **Quality score:** +  **Applicability:** | | | | | | | | | | | | | |
| **Source population:**  *Japanese residents of 14 administrative districts supervised by 4 public health centres *43,149 residents ages 40-59 years living in Ninohe, Karumai, Yokote, Omonogawa, Minami-Saku County, Gushikawa, Onna completed the baseline questionnaire in 1990 (response rates: 76% for men and 82% for women) *32,126 participants completed 5- and 10-year follow-up questionnaires | | **Study population:**  *analysis restricted to **28,893** eligible participants with complete data and who responded to the baseline, 5- and 10-year follow-up questionnaires (response rates: 70.4% for men and 78.2% for women)   *Follow-up:* *10 years  **Exclusion:**  Participants with the following characteristics at baseline: *diabetes (n=1120) *cardiovascular disease (n=470) *chronic liver disease (n=311) *kidney disease (n=546) *cancer (n=689) *missing exposure data (n=298)  **Attrition:**  *no significant differences in terms of BMI and lifestyle characteristics between those who responded to both follow-up questionnaires and those who did not | | | | **Exposures:**  *smoking habits, alcohol intake, and physical activity assessed through self-administered questionnaires   *Smoking:* *smokers classified as ‘never smoked’, ‘former smokers’, ‘current smokers’  >‘current smokers’ further sub-divided into: smoked 1-19 or >=20 cigarettes/day  *Alcohol use:* *alcohol intake measured using data on type, frequency per week, and daily quantity consumed *total daily alcohol intake was calculated, using the alcohol content of the following beverages in calculations: 23g ethanol per 180ml of sake, 36g ethanol per 180 ml of sochu or awamori, 10g ethanol per 30ml of whisky/brandy, 6g ethanol per 60ml of wine, 23g ethanol per 633ml of beer  *participants classified as ‘non-drinkers and infrequent occasional drinkers’ (consuming alcohol on 3 or fewer days per month) or ‘drinkers’ who were further subdivided by tertiles of daily ethanol consumption *questionnaire measured alcohol use with high degree of validity  *Physical activity:* *physical activity assessed through questions on leisure time sports activities *if respondents participated in sports at least once a week, they were categorized as ‘active’; otherwise, they were considered ‘inactive’ | | **Outcomes:**  *men who reported diagnosis of incident type 2 diabetes at 5- and/or 10-year follow-up questionnaires *to determine the validity of self-reported diabetes diagnoses: >a sample of medical records was reviewed, and diabetes diagnosis confirmed for 82%-94% of participants >results from blood samples of volunteering participants were compared to self-reported diabetes diagnoses; sensitivity and specificity of study questionnaire for diabetic hyperglycemia were 46% and 98%, respectively | | **Analysis:**  *multiple logistic regression used to assess contributions of smoking, alcohol use, and physical activity on type 2 diabetes incidence over 10-year follow-up period **Confounders:**  *age, BMI (also assessed as effect modifier), family history of diabetes, hypertension  Effect modification: *sex, BMI | | **Results:**  *703 and 480 incident cases of diabetes among men and women, respectively, during 10-year follow-up period  Men: *the odds of developing diabetes over 10 years were higher among those smoking >=20 cigarettes/day and past smokers compared to never-smokers (OR=1.37, [1.11, 1.69]; OR=1.35, [1.08, 1.69], respectively) *the odds of developing diabetes were higher among men drinking between 23-46g ethanol/day than ‘non-drinkers and infrequent occasional drinkers’ (OR=1.26, [1.02-1.56])  Women: *the odds of developing diabetes over 10 years were higher among those smoking >=20 cigarettes/day and past smokers compared to never-smokers (OR=2.94, [1.57, 5.50]; OR=2.77, [1.67, 4.61], respectively)  Stratification by BMI among males: *compared to ‘non-drinkers and infrequent occasional male drinkers’, the odds of developing diabetes over 10 years were higher among males consuming 23-46g/day and over 46g/day of ethanol (OR=1.91, [1.05, 3.46]; OR=2.89, [1.63, 5.11], respectively)  **Limitations:**  *self-reported information leading to possible under-estimation of OR *possible follow-up bias between diabetic and non-diabetic categories (e.g. excess mortality among diabetic patients affecting response rate) **Source of Funding:**  *grant-in-aid for Cancer Research and for the Second Term Comprehensive Ten-Year Strategy for Cancer Control, and Health Sciences Research grants | |
| **Authors**: Wang L, Manson JE, Buring JE, Lee IM, Sesso HD **Year:** 2008 **Citation:** Hypertension. 2008;51(4):1073-1079 **Country of study:** US **Aim:** To determine the association between dietary intake of dairy products, calcium, and vitamin D and the risk of hypertension in middle-aged and older women **Study design:** Longitudinal **Quality score:** +  **Applicability:** | | | | | | | | | | | | | |
| **Source population:**  *39,876 US female health professionals ages 45 years and older and free of cardiovascular disease and cancer (except non-melanoma skin cancer) were randomized into the Women’s Health Study in 1992-95 (baseline)  *39,310 completed semiquantitative food frequency questionnaire (SFFQ) | | **Study population:**  ***28,886** women *mean age of 53.8 years  *Follow-up:* *10 years  **Exclusion:**  Women with: *insufficient completion of the SFFQ *implausible total energy intake *missing data on exposure *pre-randomization cardiovascular disease or cancer  *baseline hypertension  Censoring: *women who developed cardiovascular disease over study period censored on date of cardiovascular disease diagnosis  **Attrition:**  - | | | | **Exposures:**  *annual follow-up SFFQ used to assess portion size of each food item that participants consumed over the previous year with responses ranging from ‘never or less than once per month’ to ‘6+ per day’  *average daily intake calculated based on intake frequency and portion size of item *total dairy product intake summed dairy items (e.g., low-fat dairy items include skim or low-fat milk, sherbet, yogurt, and cottage/ricotta cheese, high-fat dairy items include whole milk, cream, sour cream, ice cream, cream cheese, and other cheese) *nutrient intake was calculated based on intake frequency of item as well as the nutrient content of the specified portion size  *Pearson correlation coefficients between SFFQ responses and dietary records ranged from 0.25-0.79 | | **Outcomes:**  * self-reported incident hypertension defined as meeting one of the following: new physician diagnosis of BP; newly-initiated BP treatment; self-reported systolic BP >=140 mmHg; self-reported diastolic BP >=90 mmHg *time of event was also captured  *high validity of self-reported BP | | **Analysis:**  *Cox regression model used to estimate the hazard ratio (presented as the relative risk of developing incident hypertension across quintiles of dairy intake  **Confounders:**  *age, race, total energy intake, randomized treatment, smoking, alcohol use, exercise, postmenopausal, multivitamin use, BMI, history of diabetes and hypercholesterolemia, intake of fruit and vegetable, whole grain, red meat *when dietary calcium and vitamin D were assessed: same confounders were included in the adjusted model as above, with the exception of: *intake of fruit and vegetable, whole grain, red meat; also, additional confounders included in adjusted model were dietary sodium, fiber, saturated fats, and cholesterol* | | **Results:**  *8,710 cases of incident hypertension identified during 10 years of follow-up *the risk of hypertension was smaller across increasing quintiles of low-fat dairy products and total dairy products (trend test p-values: 0.001 and 0.0003) *trend test p-value was significant (p=0.002) for risk of hypertension across quintiles of intake of skim milk, although no clear direction of trend was evident (same observation applies to hypertension risk and quintiles of intake of dietary vitamin D, trend test p-value=0.02) *risk for hypertension appears to decrease with increasing intake of dietary calcium (p<0.0001)  **Limitations:**  *exposure was measured at a single point in time (subject to random error leading to underestimation of true association) *self-reported outcome data *residual confounding *limited generalizability as cohort comprises predominantly white health professional women **Source of Funding:**  *National Institutes of Health, Bethesda, Md. | |
| **Authors**: Wang L, Lee IM, Zhang SM, Blumberg JB, Buring JE, et al **Year:** 2009 **Citation:** Am J Clin Nutr. 2009;89(3):905-912 **Country of study:** US **Aim:** To assess the association between baseline flavonoid intake and the risk of total and site-specific cancers **Study design:** Longitudinal **Quality score:** +  **Applicability:** | | | | | | | | | | | | | |
| **Source population:**  *39,876 female health professionals free of cardiovascular disease and cancer (except non-melanoma skin cancer) were assigned into the Women’s Health Study, a randomized double-blind, placebo-controlled trial *39,876 women completed a validated semi-quantitative food-frequency questionnaire (SFFQ), which has been shown to have reasonable validity and reproducibility | | **Study population:**  ***38,408** women  *Sociodemographics*: *predominantly white female health professionals  *Follow-up:*  *person-years of observation were calculated from time of randomization to cancer diagnosis, death, last day of follow-up, or 16 March 2007, whichever came first *average of 11.5 years of follow-up  **Exclusion:**  *insufficient completion of SFFQ (n=21) *women with implausible daily energy intake [n=829] *women with cardiovascular disease or cancer diagnosed before randomization but reported after randomization [n=59]  **Attrition:**  - | | | | **Exposures:**  *at baseline, participants were asked how often they had consumed specified unit of each food item listed in questionnaire, with responses ranging from ‘never or less than once per month’ to ‘6+ per day’ *average daily intake of each food item was calculated by multiplying intake frequency by portion size of items *nutrient intake was calculated by multiplying unit intake frequency by nutrient content of portion size  *total flavonoids were analysed and these included 3 flavonols (quercetin, kaempferol, myricetin) and 2 flavones (apigenin and luteolin); major sources of flavonoids analysed included apples, broccoli, onions, tofu, and tea | | **Outcomes:**  *confirmed cancer cases, except non-melanoma skin cancer *every six months and then annually thereafter, data on newly-diagnosed cancer was collected through questionnaires *deaths ascertained through family member reports, postal authorities, National Death index; cancer was identified through pathology or cytology reports, or rarely, through clinical and radiologic or laboratory marker evidence | | **Analysis:**  *Cox regression was used to assess the association between 1) intake of total and individual flavonoids, or the 2) intake of flavonoid-rich foods and incidence of total invasive cancer as well as site-specific cancers **Confounders:**  *age, race, total energy intake, randomized treatment assignment, BMI, smoking, alcohol use, physical activity, postmenopausal status, hormone replacement therapy use, multivitamin use, family history of cancer in a parent or sibling, and intake of fruit and vegetables, fiber, folate, and saturated fat | | **Results:**  *3234 cancer cases identified during average of 11.5 years of follow-up *incidence of total invasive cancer did not differ significantly across quintiles of total and individual quantified flavonoid intakes  *lack of an association between total quantified flavonoid intake and incidence of common site-specific cancers (e.g. breast cancer, colorectal cancer, lung cancer, endometrial cancer, ovarian cancer); tests for linear trends in incidence of site-specific cancers across quintiles for each individual flavonoid were also non-significant *no association between total and individual flavonoid intake and rare site-specific cancers (e.g. stomach, pancreatic, bladder, brain, thyroid, cervical cancer, lymphoma/leukemia) *similar lack of an association between intake of flavonoid-rich foods and total cancer, as well as site-specific common and rare cancers  **Limitations:**  *possible incomplete assessment of flavonoid intake due to missing information on certain flavonoid-containing food items *quantities of flavonoids in food may differ by species variety, growth condition, maturation, preparation, and food-processing methods; therefore, these factors can contribute to random errors in intake assessment and bias results towards null *random error of self-report (cumulative intake and dietary changes during follow-up not captured – can lead to underestimation of associations) *lack of associations with cancer may have been due to small number of cases *associations with cancer may have been present for different flavonoid intake amounts  **Source of Funding:**  *****none reported | |
| **Authors**: Wang L, Lee IM, Manson JE, Buring JE, Sesso HD **Year:** 2010 **Citation:** Arch Intern Med. 2010;170(5):453-461 **Country of study:** US **Aim:** To assess the association between alcohol consumption and risk of becoming overweight or obese in middle-aged and older women **Study design:** Longitudinal **Quality score:** +  **Applicability:** | | | | | | | | | | | | | |
| **Source population:**  *39,876 US female health professionals ages 38-89 years and free of cardiovascular disease and cancer (except non-melanoma skin cancer) were randomized into the Women’s Health Study in 1992-95 (baseline) | | **Study population:**  *of 19,563 women with a baseline BMI of 18.5-25 kg/m2, analysis was restricted to **19,220** women meeting inclusion criteria   *Sociodemographics:* *predominantly white female health professionals  *Follow-up:* *average 12.9 years of follow-up  **Exclusion:**  Women with: *missing data on baseline exposure [n=5] *no updated outcome information during study period [n=128] *baseline diabetes [n=194] *pre-randomization cardiovascular disease or cancer [n=17]  **Attrition:**  - | | | | **Exposures:**  *self-reported alcohol intake assessed using baseline, validated, semiquantitative food frequency questionnaire (SFFQ) that measured frequency of alcohol consumption over the past year with responses ranging from ‘never or less than once per month’ to ‘6+ per day’  *alcohol intake also took alcohol content of beverages into account, assuming ethanol of 13.2g for 360 ml beer, 10.8 g for 12. ml red or white wine, and 15.1 g for 45 ml liquor *total alcohol intake categorized as: 0, >0- <5, 5- <15, 15- <30, >= 30g/day *correlation coefficient comparing self-reported alcohol intake in questionnaire with diet records ranged from 0.81-0.90 *SFFQ has demonstrated reasonable validity and reproducibility | | **Outcomes:**  *incident cases of overweight or obesity assessed at 2-, 3-, 5-, 6-, and 9-year follow-up questionnaires for 19,220 women (16,322 of these women continued to have their weight updated at 11-, 12-, and 13-year follow-up time points) *BMI was calculated using self-reported weight at follow-up and height at baseline, and categorized as: normal (18.5 to <25 kg/m2); overweight (25 to 30 kg/m2); obese (>=30 kg/m2) *cases of ‘overweight or obesity’ comprised women with a normal BMI at baseline and a BMI >=25 kg/m2 at follow-up *cases of ‘obesity’ comprised women with a normal BMI at baseline and a BMI>=30 kg/m2 at follow-up  *Time of event and censoring:* *for cases, time of event was estimated from last-reported normal BMI to first reported BMI >=25 kg/m2  *for non-cases, time of censoring was the latest date of reported normal BMI  *women who developed diabetes during study period censored on date of diabetes diagnosis  *high correlation between self-reported weight and clinic-measured weight (r=0.97) | | **Analysis:**  *Cox regression model used to estimate the hazard ratio (presented as the relative risk of becoming a) overweight or obese and b) obese across categories of total alcohol intake) **Confounders:**  *race, baseline BMI, randomized treatment (vitamin E, aspirin, B-carotene, placebo), total non-alcohol energy intake, physical activity, smoking, post-menopausal status, post-menopausal hormone use, multivitamin use, history of hypercholesterolemia, hypertension, intake of fruit and vegetables, whole grains, refined grains, red meats and poultry, low-fat dairy products, high-fat dairy products, energy-adjusted total fat, carbohydrates, fiber | | **Results:**  *of 19,220 women with normal baseline BMI, 7,942 (41.3%) become ‘overweight or obese’ over 12.9 years of follow-up, and 732 (3.8%) became obese during this time *increasing levels of alcohol consumed contributed to decreasing incidence of overweight or obesity (BMI >=25); this trend was observed for ‘all alcohol’, beer, red wine, white wine (trend test p-values: <0.0001, 0.04, < 0.0001, and <0.0001, respectively) >For example, the risk of overweight or obesity was smallest among those consuming 15-<30 g/day of ‘total alcohol’ compared to non-drinkers (RR=0.70, [0.62, 0.79])  *increasing levels of alcohol consumed contributed to decreasing incidence of obesity (BMI >=30); this trend was observed for ‘all alcohol’, beer, red wine, white wine, liquor (trend test p-values: <0.0001, 0.02, 0.004, 0.0003, 0.005) >For example, the risk of obesity was smallest among those consuming >=30 g/day of ‘total alcohol’ compared to non-drinkers (RR=0.29, [0.15, 0.54])  *similar inverse association between total alcohol intake and risk of becoming overweight or obese was observed for women in different age strata (<50, 50-<60, >=60), smoking strata, physical activity groups, and baseline BMI groups  **Limitations:**  *self-reported weight resulting in non-differential misclassification *social desirability bias with respect to alcohol intake (under-report), and lack of information on change in alcohol drinking patterns over time *residual confounding *limited generalizability as participants were predominantly white female health professionals **Source of Funding:**  *National Institutes of Health, Bethesda, MD | |
| **Authors**: Wang L, Manson JE, Gaziano JM, Buring JE, Sesso HD **Year:** 2012 **Citation:** Am J Hypertens. 2012;25(2):180-189 **Country of study:** US **Aim:** To determine the association between intake of total, subgroup, and individual items of fruits and vegetables and the risk of hypertension **Study design:** Longitudinal **Quality score:** +  **Applicability:** | | | | | | | | | | | | | |
| **Source population:**  *39,876 US female health professionals ages 39-89 years, and free from cardiovascular disease and cancer (except non-melanoma skin cancer) were included in the Women’s Health Study, a randomized, double-blind, placebo-controlled trial, with follow-up questionnaires every 6 months for the first year and annually thereafter | | **Study population:**  *analysis restricted to **28,082** out of 39,310 women who completed semi-quantitative food frequency questionnaire (FFQ)   *Sociodemographics:* *predominantly white female health professionals  *Follow-up:* *average follow-up of 12.9 years *Follow-up from randomization at baseline to the date of incident hypertension (outcome), the last day in the study, or Feb. 2007, whichever came first  **Exclusion:**  Women with: *hypertension (systolic BP>=140 mmHg or diastolic BP >=90 mmHg or history of anti-hypertensive treatment) at baseline (n=10,751) *(self-reported) implausible total daily energy intake [n=829] *pre-randomization cardiovascular disease or cancer [n=41] *missing FFQ items [n=21] *incomplete outcome information [n=109]  Censoring: *women who developed cardiovascular disease over study period and whose treatment may influence BP  **Attrition:** - | | | | **Exposures:**  *self-reported fruit and vegetable consumption was assessed using validated FFQ that included 28 vegetable items and 16 fruit items *participants were asked how often they had consumed a unit of each food item over the past year with responses ranging from ‘never or less than once per month’ to ‘6+ times per day’  *average daily intake of individual fruit and vegetable items was summed to create measures of a) total fruit, b) total vegetable, and c) total fruit and vegetable intake *vegetables were also categorized into: green leafy vegetables; cruciferous vegetables; and dark and yellow vegetables *Pearson correlation coefficients comparing FFQ responses with dietary records ranged from 0.50-0.84 | | **Outcomes:**  *self-reported incident hypertension defined as meeting one of the following: physician diagnosis of BP; newly-initiated BP treatment; self-reported systolic BP >=140 mmHg; self-reported diastolic BP >=90 mmHg *time of event was self-reported (correlation coefficient comparing self-reported with measured BP among health professionals: 0.60-0.72) *high validity of self-reported BP | | **Analysis:**  *Cox models were used to assess influence of hypertension across levels of fruit and vegetable intake  Stratification by age, BMI, smoking status, baseline systolic/diastolic BP **Confounders:**  *age, race, total energy intake, randomized treatment (vitamin E, aspirin, B-carotene, placebo), smoking, alcohol use, exercise, postmenopausal status, postmenopausal hormone use, multivitamin supplement use, history of diabetes or hypercholesterolemia, intake of whole grains, red meats, low-fat dairy product, and nuts; if fruit intake was assessed, model was also adjusted for vegetable intake, and vice versa | | **Results:**  *during 12.9 years of follow-up, 13,633 women developed incident hypertension *there was a significant inverse association between baseline intake of apples, oranges, and raisins, and incidence of hypertension (trend test p-values: 0.03, 0.01, 0.0004);  *women consuming 1 serving/week and 2-4 servings/week of strawberries had a slight increased risk for hypertension compared to women never or rarely consuming strawberries (trend test p-value: 0.04) *compared to women in the lowest category of intake (<0.2 servings/day) of dark-yellow vegetables, the risk of hypertension was lowest among women in the fourth (0.6-<1.0 servings/day) and highest (>=1.0 servings/day) categories of intake (HR=0.93, [0.87, 0.99]; HR=0.88, [0.82, 0.95], respectively) *trend tests between intake of cruciferous vegetables, dark-yellow vegetables, legumes, onions, and incident hypertension were significant (p<0.05); however, no clear direction was evident for the trend *the risk of hypertension was lower among women consuming 0.6-<1.0 servings/day and >=1.0 servings/day of dark-yellow vegetables compared to women in the lowest intake category of <0.2 servings/day (HR=0.93, [0.87, 0.99]; HR=0.88, [0.82, 0.95], respectively)  Stratification by BP: *there was a significant interaction of baseline BP with fruit intake: >among women with baseline systolic/diastolic BP <120/80 mmHg, the risk of hypertension decreased with increasing total fruit intake (trend test p-value=0.003); no trend observed for BP >=120/80 mmHg  **Limitations:**  *fruit and vegetable intake measured at one point in time (baseline), thus introducing possible measurement error and non-differential misclassification *self-reported outcome data again leading to misclassification *residual confounding by unmeasured or imprecisely measured hypertension risk factors *limited study generalizability due to homogeneity of cohort in terms of race, education, and SES **Source of Funding:**  *National Institutes of Health, Bethesda, MD, along with an investigator-initiated grant from the California Strawberry Commission, Watsonville, CA | |
| **Authors**: Wannamethee SG, Shaper AG, Walker M **Year:** 2001 **Citation:** Br J Cancer. 2001;85(9):1311-1316 **Country of study:** England **Aim:** To assess physical activity and risk of cancer in middle-aged men **Study design:** Longitudinal **Quality score:** +  **Applicability:** | | | | | | | | | | | | | |
| **Source population:**  *prospective study of 7735 men ages 40-59 years selected from registers of general practices in 24 towns in England, Wales and Scotland; initially examined in 1978-80 | | **Study population:**  ***7630** men with available data   *Follow-up:* *follow-up of 18.8 years achieved for 99% of cohort  **Exclusion:**  *excluded men who were diagnosed with cancer prior to or in the same year as screening [n=42]  **Attrition:**  - | | | | **Exposures:**  *physical activity assessment: regular walking or cycling (weekday journeys including travel to and from work), recreational activity (gardening, pleasure walking, do-it-yourself jobs) and sporting (running, golf, swimming, tennis, sailing, digging) *total physical activity score calculated for each participant based on frequency and type (intensity) of activity >categories used: inactive and occasional; light; moderate; moderately vigorous; vigorous | | **Outcomes:**  *Cancer cases up to Dec. 1997 ascertained from death certificates, cancer registry, postal questionnaires mailed to surviving members in 1992, 1996, 1998 | | **Analysis:**  *Cox proportional hazards model used to assess effects of physical activity on risk of cancer **Confounders:**  *age, BMI, alcohol consumption, smoking, social class | | **Results:**  *969 cases out of 7588 men  *men who engaged in vigorous physical activity had a significantly reduced risk of total cancers compared to those who did not or engaged in occasional activity (RR=0.65, [0.44, 0.88]) *the greater the physical activity level, the lower the risk for total cancers (sig. trend, p<0.0001) *moderate-vigorous exercise was associated with a significantly reduced risk of combined upper digestive tract cancer (oral, esophagus, stomach cancer) (OR=0.37 [0.16, 0.86]) *vigorous exercise was associated with a  significantly increased risk of bladder cancer [OR=2.06, (1.08, 3.95)]  **Limitations:**  *residual confounding, ie. Diet  **Source of Funding:** *Department of Health, University College Medical School, London | |
| **Authors:** Wannamethee SG, Shaper AG  **Year:** 2002  **Citation:** Heart 2002;87:32–36  **Country of study:** UK  **Aim of study:** Examine effects of taking up regular drinking by middle aged non-drinkers and occasional drinkers on major coronary heart disease events and total mortality  **Study design:** Prospective cohort study  **Quality score: (++, + or -):**  **External validity score: (++, + or -):** | | | | | | | | | | | | | |
| **Number of people**  7735 men  **Demographics** Not reported | | **Number of people** 7157 men  **Characteristics**  **TT (292).** Mean age (years) 51.1: Never smoked 43.2: Current smoker (Q5) 28.1: Active (Q1) 38.3   **Ex (299).** Mean age (years) 51.2: Never smoked 16.1: Current smoker (Q5) 40.8: Active (Q1) 29.6  **Stable occ (1150).** Mean age (years) 49.7: Never smoked 30.9: Current smoker (Q5) 29.9: Active (Q1) 41.3  **New occ (782).** Mean age (years) 50.1: Never smoked 25.1: Current smoker (Q5) 33.7: Active (Q1) 38.0  **New reg (305).** Mean age (years) 49.4: Never smoked 24.5: Current smoker (Q5) 30.1: Active (Q1) 44.0  **Reg (3675).** Mean age (years) 49.6: Never smoked 22.8: Current smoker (Q5) 32.6: Active (Q1) 41.3  **Location**  England,Wales, and Scotland  **Recruitment strategy** men aged 40–59 years selected from the age–sex registers of general practices in each of 24 towns in England,Wales, and Scotland  **Length of follow-up**  16.8 years (CI) 15.5 to 18.0 years  **Response rate and loss to follow-up**  78% and 99%  **Eligible population** Men in Britain  **Excluded populations**  Women | | | | **Relevant exposures**  Smoking, drinking, physical activity  **Time** 1978–1980  M**easurment of exposure** never smoked, ex-smokers at both Q1 and Q5, ex-smokers at Q5 only, and two groups of current cigarette smokers at Q5 (1–19 and > 20/day).  A physical activity score was derived on the basis of frequency and type of activity: inactive, occasional, light, moderate, moderately vigorous, and vigorous.  Eight drinking categories: non-drinkers; occasional drinkers: < 2 units a month; light drinkers: weekend, three to six drinks a day; weekdays, one to two drinks a day; 1–15 units/week; moderate drinkers: weekend, more than six drinks a day; weekdays, three to six drinks a day; 15–42 units/week; heavy drinkers: more than six drinks a day; > 42 units/week.  In men without diagnosed coronary heart disease alcohol categories were classified into six groups: Teetotallers; Ex-drinkers; stable occasional; new occasional; new regular drinker and Stable regular drinkers | | **Outcomes** risk of mortality and major coronary heart disease events  **outcome measurement** All deaths and all major coronary heart disease events occurring in the period were recorded  Amajor coronary heart disease event includes non-fatal myocardial infarction, fatal myocardial infarction, and sudden cardiac death classified as caused by coronary heart disease  Information on non-fatal myocardial infarction was obtained from reports provided by general practitioners, supplemented by regular two yearly reviews of the general practice records and by self reported questionnaires  **Time**  Up to December 2000 | | **Analysis strategy** Cox proportional hazards model. Adjustments for risk factors were based on risk factors measured at Q1 and Q5. In the adjustment, age and body mass index were fitted as continuous variables, and physical activity (six levels), smoking (five levels), social class (seven levels), employment status (yes/no), self rated health and recall of stroke (yes/no), and diabetes (yes/no) were fitted as categorical variables  C**onfounders** Relative risk adjusted for age, social class, smoking, BMI, physical activity, employment status, pre-existing stroke, diabetes, regular drug treatment, and self assessed health status.  adjustment for physical activity is based on physical activity data at screening. | | **Number** 654 men  **Effect estimates** diagnosed coronary heart disease by alcohol group TT (43) 95% CI  CHD mortality Cases 17  Rate/1000 p-y 34.0  Adjusted RR 1.71 (0.92 to 3.15)   CVD mortality Cases 21  Rate/1000 p-y 42.0  Adjusted RR 1.62 (0.93 to 2.82)  Total mortality Cases 28  Rate/1000 p-y 56.0  Adjusted RR 1.31 (0.82 to 2.10)   Ex (59) 95% CI CHD mortality Cases 22  Rate/1000 p-y 37.1  Adjusted RR 1.56 (0.89 to 2.73)   CVD mortality Cases 26  Rate/1000 p-y 43.8  Adjusted RR 1.49 (0.89 to 2.49)   Total mortality Cases 39  Rate/1000 p-y 65.7  Adjusted RR 1.46 (0.96 to 2.22)   Stable occ (112) CHD mortality Cases 31  Rate/1000 p-y 22.8  Adjusted RR 1.00   CVD mortality Cases 38  Rate/1000 p-y 27.9  Adjusted RR 1.00   Total mortality Cases 57  Rate/1000 p-y 41.9  Adjusted RR 1.00   New occ (87)  CHD mortality Cases 27  Rate/1000 p-y 26.0  Adjusted RR 95% CI 1.05 (0.62 to 1.79)   CVD mortality Cases 33  Rate/1000 p-y 31.8  Adjusted RR 1.07 (0.66 to 1.74)  Total mortality Cases 46  Rate/1000 p-y 44.3  Adjusted RR 1.00 (0.67 to 1.51)   New reg (37)  CHD mortality Cases 12 Rate/1000 p-y 29.6  Adjusted RR 95% CI 1.19 (0.60 to 2.34)   CVD mortality Cases 16  Rate/1000 p-y 39.4  Adjusted RR 1.30 (0.72 to 2.37)  Total mortality Cases 23  Rate/1000 p-y 56.7  Adjusted RR 1.23 (0.75 to 2.04)   Reg (316) CHD mortality Cases 106 Rate/1000 p-y 28.3 Adjusted RR 95% CI 1.33 (0.88 to 2.00)  CVD mortality Cases 129 Rate/1000 p-y 34.4 Adjusted RR 1.33 (0.92 to 1.93)  Total mortality Cases 182 Rate/1000 p-y 48.6 Adjusted RR 1.25 (0.92 to 1.69)  **Significant trends** among men with a diagnosis of CHD the teetotallers and ex-drinkers had an increased adjusted risk of mortality from CHD and CVD compared with stable occasional drinkers. new regular drinkers showed no benefit for CHD mortality, and some increase (non-significant) in CVD and total mortality was observed compared with stable occasional drinkers  **Reported limitations**  No limitations reported by the author  **Source of funding** Not reported | |
| **Authors**: Wannamethee SG, Shaper AG **Year:** 2003 **Citation:** Am J Clin Nutr. 2003 May;77(5):1312-7 **Country of study:** England, Wales, Scotland **Aim:** To assess the influence of alcohol consumption patterns on body weight and weight gain **Study design:** Longitudinal **Quality score:** +  **Applicability:** | | | | | | | | | | | | | |
| **Source population:**  *7,735 men ages 40-59 years selected from registers of general practices of 24 towns in England, Wales and Scotland were examined from Jan. 1978 to July 1980 as part of the British Regional Heart Study *response rate: 78% *At baseline, questionnaires were administered to participants, physical measurements were made and blood samples drawn *7275 of the surviving participants completed a postal questionnaire in 1983-85 to assess changes in health behaviours and other risk factors | | **Study population:**  ***7,608** men  *Follow-up:* *5 years  **Exclusion:**  *men with diagnosed diabetes at baseline (n=118)  **Attrition:**  - | | | | **Exposures:**  *Baseline:* *frequency, quantity and type of alcohol measured at baseline and after 5 years, with the following categories used to classify participants: non-drinkers; occasional drinkers (<1 unit/week); light-moderate (1-20 units/week), including weekend drinkers of 1-2 or 3-6 units/day and daily drinkers of 1-2 units/day; heavy (21-42 units/week), including weekend drinkers of >6 units/day and daily drinkers of 3-6 units/day; very heavy (>42 units/week), comprising daily drinkers of >6 units/day  *1 unit of alcohol is approx. 10 g alcohol *exposure was validated using biochemical and hematologic measurements  *After 5 years:* *participants were asked about past and current drinking habits, and same drinking categories used as those at baseline  Men classified into stable or changed groups on the basis of intake reported at baseline and after 5 years: **stable groups:* stable non-occasional (non-drinkers or occasional drinkers at baseline who remained non-drinkers or occasional drinkers after 5 years); stable light-moderate (light-moderate drinkers at both waves); stable heavy (heavy or very heavy drinkers at both waves) **changed groups:* light-moderate to none-occasional (light-moderate drinkers at baseline who reported being non-drinkers or occasional drinkers after 5 years; none-occasional to light-moderate (non-drinkers or occasional drinkers at baseline who reported light-moderate drinking after 5 years); ex-heavy (heavy or very heavy drinkers at baseline who reported being non-drinkers, occasional drinkers, or light-moderate drinkers at after 5 years); new heavy (none-occasional and light-moderate drinkers at baseline who reported heavy or very heavy drinking after 5 years). | | **Outcomes:**  *BMI was measured at baseline and 5 years later using height and weight measurements (kg/m2) *high BMI: >=28 or upper quintile of BMI distribution of men at baseline  *Percentage change in body weight since baseline screening calculated:* *weight loss was defined as a loss of >=4% of body weight, weight gain as a gain of >=4% of body weight, while stable weight was weight gained or lost that was <4% of body weight *the following weight change categories were used: weight loss; stable (weight); gain of 4-10%; gain of >10% of body weight | | **Analysis:**  *logistic regression was used to assess the influence of alcohol consumption patterns on body weight and weight gain **Confounders:**  *age, social class, physical activity, cigarette smoking, initial BMI | | **Results:**  *the proportion of men with high BMI increased significantly with higher levels of alcohol intake at baseline *at baseline: stable heavy and ex-heavy drinkers had the highest proportion of men with BMI>=28 (20.8% and 23.5%, respectively) *5 years after baseline: stable (continuing) heavy drinkers and new heavy drinkers had the highest proportion of men with BMI >=28 (25.7% and 27.2%, respectively) *the odds of weight gain >=4% over 5 years were greater for stable heavy drinkers compared to stable none-occasional drinkers (OR=1.29 [1.10, 1.51])  *the odds of weight gain >=4% over 5 years were greater for new heavy drinkers compared to the stable none-occasional group (OR=1.45, [1.09, 1.92])  *Stratification by smoking:* *among non-smokers, the odds of weight gain >=4% were greater for stable heavy drinkers compared to stable none-occasional drinkers (OR=1.75, [1.19, 2.56]) *among non-smokers, the odds of weight gain >=4% were greater for ex-heavy drinkers compared to stable none-occasional drinkers (OR=1.55, [1.07, 2.25])  **Limitations:**  *findings cannot be generalized to women **Source of Funding:**  *none reported | |
| **Authors**: Waring ME, Eaton CB, Lasater TM, Lapane KL **Year:** 2010 **Citation:** Am J Epidemiol. 2010;171(5):550-6 **Country of study:** US **Aim:** To examine incident diabetes in relation to weight patterns during middle age **Study design:** Longitudinal **Quality score:** +  **Applicability:** | | | | | | | | | | | | | |
| **Source population:**  *5,209 men and women ages 28-62 years, living in Framingham, Massachusetts, and without clinically apparent cardiovascular disease were enrolled in Framingham Heart Study in 1948-52 *biennial study visits involved an interview, a clinical examination, and laboratory tests | | **Study population:**  ***1,476** participants  *Follow-up:* *from age 50 years to the first examination with diagnosis of incident diabetes or the last examination attended   **Exclusion:**  -of 5,079 who provided informed consent, the following were excluded: *enrolled in the FHS cohort after 40 years of age [n=3,071] *did not attend FHS examinations after age 50 [n=218] *those missing BMI measurements at several study waves [n=213]  -of 1,577 study participants, individuals excluded were those: *who were consistently underweight [n=20] *with diabetes before 50 years [n=23] *with missing data on covariates [n=58]  **Attrition:**  - | | | | **Exposures:**  *weight patterns during middle age (40-50 years) were assessed using principal component analysis *BMI from age 40 to 50 years was calculated and guided by standard BMI cut-offs (BMI<25, BMI 25-29.9, BMI>=30), participants were categorized as being normal weight, overweight, or obese *weight change was also measured and categorized as: weight loss, stable weight, and weight gain *weight cycling was also assessed | | **Outcomes:**  *type 2 diabetes was ascertained through assessment of non-fasting plasma glucose level and/or reported treatment with insulin or medication for diabetes | | **Analysis:**  *Cox proportional hazards regression was used to analyse the influence of weight patterns on incident diabetes  **Confounders:**  *gender, education, family history of diabetes, weight status at age 25 years, physical activity, smoking, alcohol use, hormone use, weight change, weight cycling | | **Results:**  *217 cases of type 2 diabetes diagnosed over 35,359 person-years of follow-up *diagnosis made at median age of 67.8 years *adults who were overweight during middle age had 2.9 times the rate of diabetes incidence (HR=2.9, [2.0, 4.1]) as those who were normal weight during middle age *adults who were obese had 7.7 times the rate of diabetes (HR=7.7, [4.9, 12.1]) as those who were normal weight  **Limitations:**  *fasting glucose measurements not available; study definition of diabetes may have been less sensitive than American Diabetes Association criteria *potential misclassification of diabetes diagnosis date (unlikely to explain results) *residual confounding due to measurement error and unmeasured variables (potential misclassification of smoking, alcohol, and hormone use at time of diagnosis; recall bias in recalled weight; information lacking on current or previous pregnancies/history of gestational diabetes) *biannual measurements limited ability to detect changes occurring more frequently than every 2 years  **Source of Funding:**  *****none reported | |
| **Authors**: Wiles NJ, Haase AM, Gallacher J, Lawlor DA, Lewis G **Year:** 2007 **Citation:** Am J Epidemiol. 2007;165(8):946-954 **Country of study:** Wales **Aim:** To determine the association between leisure-time and occupational physical activity on common mental disorder among middle-aged men **Study design:** Longitudinal **Quality score:** +  **Applicability:** | | | | | | | | | | | | | |
| **Source population:**  *2,512 men ages 45-59 years living in Caerphilly, South Wales took part in 1979-83 cohort study | | **Study population:**  ***2,398** men who participated in 1979-83 survey (phase I) and who had moved into the study area were re-contacted and invited to participate in 1984-88  *Follow-up:* *participants surveyed in 1984-88 (phase II – study baseline) followed-up until 1989-93 (phase III) and, again, until 1993-97 (phase IV)  *study population of men participating in phases II-III: n=1,158 *study population of men participating in phases II-IV: n=1,016  *Sociodemographics:* *53% of men employed at phase II, mean age of 57 years, 87.5% were married, two thirds were from lower social classes  **Exclusion:**  *participants with prevalent common mental disorder in phase II and non-cases taking antidepressants excluded  **Attrition:**  *of the 1,703 men who formed the total longitudinal analysis, 442 and 606 participants had missing outcome data in phases III and IV (possible loss to follow-up), respectively | | | | **Exposures:**  *phase II (baseline) leisure-time physical activity assessed using Minnesota Leisure Time Physical Activity questionnaire measuring type, frequency, and duration of activities in the last 12 months  >total energy expenditure on activities calculated and divided into low, medium, and high categories; percentage of heavy-intensity physical activity divided into none, low, and high *physical activity at current work or in last job held assessed using self-administered questionnaire (modified from the Health Insurance Plan questionnaire) focusing on time spent walking, sitting, and lifting/carrying, with scores ranging from 1 (least active) to 4 (most active) | | **Outcomes:**  *common mental disorders, comprising anxiety and depression, measured at phases II and IV using validated psychiatric disorders screening questionnaire  *incident cases based on: 1.meeting predefined cut-off score on screening questionnaire and report of antidepressant use in phase II or IV OR 2.report of antidepressant/ anxiolytic use in phase II or IV | | **Analysis:**  *logistic regression used to assess association between leisure-time or occupational physical activity and common mental disorder during phases II-III and phases II-IV, separately  **Confounders for 5-year follow-up analysis (phase II-III):**  *age, social class, marital status, employment status, smoking habits, alcohol consumption, social support, BMI, phase II common mental disorders score, phase II HDL cholesterol level, change in triglyceride level between phase II and phase III, phase II job demand variables **Confounders for 10-year follow-up analysis (phase II-IV):**  *age, social class, marital status, employment status, smoking habits, alcohol consumption, social support, BMI, phase II common mental disorder score, phase II HDL cholesterol level, change in triglyceride level between phase II and phase IV, and phase II job demand variables | | **Results:**  *5-year follow-up: >the odds of a common mental disorder at phase III were lower among those with low and high percentage of leisure time spent in heavy-intensity activity at phase II (OR=0.61, [0.40, 0.93], and OR=0.54, [0.35, 0.83], respectively)   *10-year follow-up: >the odds of a common mental disorder at phase IV were higher among those with increasing job class activity at phase II (significant results ranging from OR=1.85, [1.06, 3.23] to OR=2.41, [1.48, 3.92])  **Limitations:**  *possible exposure and outcome (nondifferential) misclassification >e.g. measuring physical activity at one point in time may cause misclassification; also, measuring disease at 2 time points may have missed relapsing cases that occurred between these two time points *residual confounding >should have included other measures of SES status, e.g. housing tenure >participants who were physically active in their leisure time may lead healthier lifestyles (thus; other unmeasured variables may account for observed association) *cannot generalize results to women **Source of Funding:**  *Medical Research Council (MRC) | |
| **Authors:** Willcox BJ, He Q, Chen R, Yano K, Masaki KH, et al.  **Year:** 2006  **Citation:** JAMA. 2006;296:2343-2350  **Country of study:** USA  **Aim of study:** Test whether midlife biological, lifestyle, and sociodemographic risk factors are associated with overall survival and exceptional survival  **Study design:** Prospective cohort study  **Quality score: (++, + or -):**  **External validity score: (++, + or -):** | | | | | | | | | | | | | |
| **Number of people**  8006  **Demographics** Japanese American middleaged men (mean age, 54 [range, 45-68] years) | | **Number of people** 5820  **Characteristics**  ***Exceptional.*** Age at baseline, y 55.5 (5.2): Height, cm 163.2 (5.4): Weight, kg 61.8 (7.8): BMI in youth 21.8 (1.9): Ever smoker, No. (%) 364 (56.4): Smoking, pack-years 14.0 (19.6): High alcohol consumption ( 3 drinks/d), No. (%) 42 (6.8) 76: Alcohol consumption, oz/mo 10.3 (16.3): Physical activity index 32.8 (4.3)  ***Usual, Diseased.*** Age at baseline, y 55.0 (5.6): Height, cm 162.9 (5.6): Weight, kg 63.1 (8.0): BMI in youth 22.0 (2.0): Ever smoker, No. (%) 469 (62.4): Smoking, pack-years 18.8 (22.4): High alcohol consumption ( 3 drinks/d), No. (%) 76 (10.3): Alcohol consumption, oz/mo 12.0 (20.9): Physical activity index 32.6 (4.1)   ***Usual, Disabled.*** Age at baseline, y 51.7 (3.4): Height, cm 162.6 (5.6): Weight, kg 63.3 (8.8): BMI in youth 22.1 (2.1): Ever smoker, No. (%) 663 (62.8): Smoking, pack-years 19.4 (22.0): High alcohol consumption ( 3 drinks/d), No. (%) 123 (11.4): Alcohol consumption, oz/mo 12.6 (24.8): Physical activity index 33.4 (4.8)   ***Nonsurvival.*** Age at baseline, y 54.1 (5.5): Height, cm 163.0 (5.7): Weight, kg 63.3 (9.8): BMI in youth 22.3 (2.3): Ever smoker, No. (%) 2561 (76.1): Smoking, pack-years 28.1 (25.5): High alcohol consumption ( 3 drinks/d), No. (%) 592 (17.6): Alcohol consumption, oz/mo 16.7 (26.9): Physical activity index 32.9 (4.5)  **Location**  island of Oahu  **Recruitment strategy** Not reported  **Length of follow-up**  up to 40 years (1965-2005)  **Response rate and loss to follow-up**  Not reported  **Eligible population** Island inhabitants  **Excluded populations**  Not reported | | | | **Relevant exposures**  smoking status, alcohol consumption, and physical activity  **Time** Not reported  M**easurment of exposure** High alcohol intake was dichotomized as 3 or more drinks/d (based on an increased risk of mortality in the HHP/HAAS cohort) or less than 3 drinks/d.   Smoking was dichotomized as ever or never. | | **Outcomes** Overall survival and exceptional survival  **outcome measurement** 1 of 4 phenotypes: (1) nonsurvivors — men who died before a specified age (75, 80, 85, or 90 years); (2) so-called “usual survivors but disabled”—men who survived until the specified age but with physical or cognitive disability and with or without a major chronic disease; (3) usual survivors with major chronic diseases but no disability; and (4) exceptional survivors  **Time**  Not reported | | **Analysis strategy** Continuous variables were dichotomized as high or low based on conventional cutoff points or median values. Odds ratios for mortality vs survival (for each specified age) and, among survivors, for having at least 1 morbid condition vs being free of these conditions were estimated using logistic regression models. Backward stepwise logistic regression was used to select a subset of variables in the final model (including variables significant at the .10 level).  C**onfounders** Age-Adjusted | | **Number** N = 5820  **Effect estimates** **Age-Adjusted ORs of Selected Risk Factors for Death (Nonsurvival) or Unhealthy Survival (Usual Survival) at Age 85** Nonsurvival vs Survival *OR (95% CI) P Value* Ever smoker  2.05 (1.83-2.29) .001  High alcohol consumption ( 3 drinks/d)  1.97 (1.68-2.31) .001  Usual Survival vs Exceptional Survival *OR (95% CI) P Value* Ever smoker  1.27 (1.06-1.53) .01 High alcohol consumption ( 3 drinks/d)  1.84 (1.29-2.62) .001  **Stepwise Logistic Regression Model of Risk of Death (Nonsurvival) or Unhealthy Survival (Usual Survival) at Age 85 Years** Nonsurvival† vs Survival *OR (95% CI) P Value* Lifestyle Ever smoker  1.94 (1.72-2.18) .001  High alcohol consumption ( 3 drinks/d)  1.58 (1.34-1.88) .001   Usual Survival‡ vs Exceptional Survival§ *OR (95% CI) P Value* Lifestyle Ever smoker  1.23 (1.01-1.50) .04 High alcohol consumption ( 3 drinks/d)  1.61 (1.11-2.34) .01  **Significant trends** ever smoking is associated with overall survival but has only a borderline association with exceptional survival.  **Reported limitations**  **Authors** study population consists of ethnic Japanese men which limits generalizability (applicability to women)  cohort effects  excluded men with chronic diseases at baseline  **Reviewers** Do not report findings for physical activity  **Source of funding** This study was supported by contract N01-HC-05102 from the National Heart, Lung, and Blood Institute, contract N01-AG-4-2149 and grants 5 U01 AG019349-05, R01 AG027060-01 (Hawaii Lifespan Study), and K08 AG22788-02 from the National Institute on Aging, and grant 2004-0463 from the Hawaii Community Foundation | |
| **Authors**: Yaffe K, Barnes D, Nevitt M, Lui LY, Covinsky K **Year:** 2001 **Citation:** Arch Intern Med. 2001;161(14):1703-1708 **Country of study:** US **Aim:** To determine whether physical activity is associated with the risk of developing cognitive decline **Study design:** Longitudinal **Quality score:** +  **Applicability:** | | | | | | | | | | | | | |
| **Source population:**  *9,704 predominantly white community-dwelling women 65 years of older recruited from Baltimore, Minneapolis, the Monongahela Valley near Pittsburgh, and Portland in the United States and examined in 1986-88 (baseline) *participants examined at baseline visit also underwent biennial clinic visits and completed annual questionnaires | | **Study population:**  *analysis restricted to **5,925** participants with cognitive test measurements at baseline and follow-up 6 and 8 years later   *Follow-up:* *mean of 7.5 years of follow-up  *Sociodemographics:* *the mean age of women across quartiles of expended energy was 70.2-71.7 years, and the mean number of years of education across energy quartiles were 12.3-13.4 years **Exclusion:** *black women *women unable to walk without assistance or who had bilateral hip replacements *women with baseline cognitive impairment [n=950], with missing baseline cognitive scores [n=53] with baseline physical limitations [n=939], with missing information on physical limitations [n=10], and women who did not complete the baseline physical activity assessments [n=51] *women who died [n=596], were unavailable for follow-up [n=238], did not have follow-up cognitive measurements [n=942]  **Attrition:** *women without cognitive follow-up measurements had lower baseline scores on cognitive testing and were less physically active compared to participants (p<0.001) | | | | **Exposures:**  *self-reported baseline physical activity was assessed using: 1. interview questions on walking and stair climbing, and 2. a reliable scale measuring the frequency and duration of participation per week during the past year in 33 different physical activities (used to calculate kilocalories expended per week) | | **Outcomes:**  *cognitive assessment using MMSE performed at baseline, and follow-up 6 and 8 years later | | **Analysis:**  *logistic regression was used to assess the influence of physical activity (blocks walked per week or total kilocalories expended per week) on cognitive function in all women, as well as in sub-groups of women ages <=70 or >70 years; women with educational level <12 or >=12 years; and among those with or without of co-morbidities  **Confounders:**  *baseline age, educational level, health status, functional limitation, depression score, stroke, diabetes, hypertension, myocardial infarction, smoking, estrogen use | | **Results:**  *1178 (20%) participants developed cognitive decline over 7.5 years of follow-up  **analysis of blocks walked per week (n=5,921):* >the odds of cognitive declined were lowest among the two highest physical activity quartiles compared to the lowest activity quartile (OR=0.63 [0.52, 0.77]; OR=0.66 [0.54, 0.82])   **analysis of total kilocalories expended per week (n=5,925):* > the odds of cognitive decline were lowest among the two highest physical activity quartiles compared to the lowest activity quartile (OR=0.78 [0.64, 0.96]; OR=0.74 [0.60, 0.90])  *the odds of cognitive decline were low among those in the highest physical activity quartile regardless of age, comorbidities, education, (same findings observed when physical activity was assessed as blocks walked per week or kilocalories expended per week)  **Limitations:**  *self-reported data (recall bias) *clinical assessment for dementia not performed, therefore cannot determine cause of cognitive decline *sample was not racially diverse **Source of Funding:**  *Public Health Service, Bethesda, Md. | |
| **Authors**: Yu S, Yarnell JW, Sweetnam PM, Murray L; Caerphilly study **Year:** 2003 **Citation:** Heart. 2003;89(5):502-506 **Country of study:** Wales **Aim:** To assess the influence of leisure or job physical activity on all-cause, CHD, or CVD mortality **Study design:** Longitudinal **Quality score:** +  **Applicability:** | | | | | | | | | | | | | |
| **Source population:**  *2,512 men ages 45-59 years were recruited in 1979-1983 from Caerphilly and its surrounding villages | | **Study population:**  *analysis restricted to **1975** men, including those re-examined in 1984-88 and men who had moved into the study area   *Follow-up:* *mean follow-up of 10.5 years *follow-up from date of examination in 1984-88 to date of death or Sept. 25, 1997  **Exclusion:**  *men with symptomatic evidence of CHD, history of myocardial infarction, grade 1 or grade 2 angina, probable ischemia [n=393] *men who died within 2 years of study inception [n=30]  *deaths from congenital anomalies or injury and poisoning were censored for analyses of all-cause mortality (n=7) *men with missing data on variables related to the job physical activity questionnaire (n=23)  **Attrition:**  - | | | | **Exposures:**  *physical activity was assessed using self-administered questionnaires   *Leisure physical activity* *validated baseline Minnesota Leisure Time Physical Activity questionnaire administered in 1984-1988 to assess type and duration of leisure activities during the previous 12 months and to estimate energy expenditure (expressed as an activity index [AI] in kcal/day)  >4 AI scores characterized each person, one for each class of intensity activity and their sum total AI   *Job physical activity* *job physical activity questionnaire (slightly modified Health Insurance Plan questionnaire) used to assess physical activity at work, or at last job with categories ranging from low to high occupational physical activity | | **Outcomes:**  *mortality from all causes, CVD, and cancer were ascertained using the National Health Service Central Registry | | **Analysis:**  *Cox proportional hazards regression was used to assess the influence of leisure or job physical activity on all-cause, CHD, or CVD mortality **Confounders (for analysis of total activity during leisure):**  *age, diastolic blood pressure, BMI, smoking status, social class (manual), family history of CHD among first degree relatives before age 55, history of diabetes mellitus in the past 5 years, job physical activity class  **Confounders (for analysis of heavy intensity activity during leisure):**  *confounders listed above, combined light and moderate intensity activity | | **Results:**  *252 total deaths over 10 years, with 111 deaths being caused by CVD, 82 by CHD, and 98 by cancer  *Analysis of total activity during leisure:* *the greater the kcal/day consumed through total activity during leisure, the smaller the risk of CHD death (trend p-value=0.039) *people expending 395.5-2747.2 kcal/day had a lower risk of CHD death than those expending 0.0-161.6 kcal/day (HR=0.55, [0.31, 0.98])  *Analysis of heavy intensity activity during leisure:* *the greater the energy expenditure of heavy intensity activity during leisure at baseline, the smaller the risk of all-cause death, CVD death, and CHD death during follow-up (trend p-values: 0.006, 0.001, and 0.009, respectively)  *people expending 23.9-2142.9 kcal/day had a lower risk of all-cause death, CVD death, and CHD death than those expending 0.0-0.6 kcal/day (HR=0.61, [0.43, 0.86]; HR=0.38, [0.21, 0.67]; HR=0.36, [0.18, 0.73], respectively)  **Limitations:**  *lack of information on changes of physical activity over time can cause misclassification  *men with low levels of leisure-time physical activity at baseline may have already been sick; thus, the illness may have been the cause rather than the result of lack of physical activity  *self-reported data resulting in potential misclassification  **Source of Funding:**  *British Heart Foundation and the Medical Research Council | |
| **Authors**: Xu Q, Anderson D, Courtney M **Year:** 2010 **Citation:** Health Care Women Int. 2010;31(12):1082-1096 **Country of study:** Australia **Aim:** To determine the influence of lifestyle factors on mental outcomes **Study design:** Longitudinal **Quality score:** -  **Applicability:** | | | | | | | | | | | | | |
| **Source population:**  *10,923 women from South East Queensland, Australia recruited from rural and urban areas | | **Study population:**  *886/1,500 women ages 45-60 years were recruited and participated in 2001 survey and of these participants, 564 agreed to participate again in 2006  *analysis restricted to **564** women  *2006 response rate: 37.6%   *Follow-up:* Non-response for 2006 survey due to: *migration out of study area [n=2] *death [n=3] *non-participation [n=28] *address change [n=104] *loss to follow-up [13] *other reasons for non-response [n=172]   *Sociodemographics:* *mean age of women was 55 years *78.1% (n=438) of women were married or in relationships; 83.1% (n=466) were born in Australia; 28.4% (n=159) were college-educated; 40.8% (n=219) did not have paid employment; 41.8% (n=236) had family annual income less <= $40,000  **Exclusion:**  -  **Attrition:**  *322/886 women dropped out between 2001 and 2006 *women lost to follow-up were slightly younger than those who remained (54.36 vs. 54.95 years, p=0.003) | | | | **Exposures:**  *physical activity measure assessed frequency of participation in exercise each week using the following response options:  >none >1-2 times/week >3-4 times/week >5-6 times/week *alcohol use was measured using the following options: >never >drank in past >occasionally >regularly *smoking status was categorized as >never smoker >past smoker >current smoker *caffeine consumption was dichotomized (yes/no) | | **Outcomes:**  *general mental well-being, and psychological symptoms including depression and anxiety were measured using SF-36 and the self-reported Greene Climacteric Scale (GCS) questionnaire, respectively *test-retest reliability of the GCS is 0.87 | | **Analysis:**  *logistic regression was used to determine the influence of lifestyle factors on mental outcomes **Confounders:**  *BMI, sociodemographics, menopausal status, mental health status in 2001 | | **Results:**  *women who exercise 5-6 times per week scored 1 point lower on the anxiety scale (p=0.013), 1 point lower on the depression scale (p=0.001), 2 points lower on the psychological scale (p=0.002), and 9 points higher on the mental well-being scale (p=0.001) compared to women who did not exercise *women who smoked had a 6.725 point lower score in mental well-being than non-smokers (p=0.006) *women who consumed caffeinated drinks regularly had a lower mental well-being by 5 points compared to those who did not drink caffeinated beverages *past alcohol drinkers reported lower anxiety scores compared to non-drinkers (p=0.040)  **Limitations:**  *physical activity measurement did not include intensity and duration *alcohol measurement may be imprecise; potentially useful to assess volume of alcohol consumption **Source of Funding:**  *none reported | |
