# Supplementary material for: Behavioural Risk Factors in Mid-Life Associated with Successful Ageing, Disability, Dementia and Frailty in Later Life: A Rapid Systematic Review
Source: PLoS One. 2016 Feb 4;11(2):e0144405. doi: 10.1371/journal.pone.0144405 (PMC4742275; doi:10.1371/journal.pone.0144405)
Supplement: S1 Text — (DOCX) [file pone.0144405.s006.docx]

**PROTOCOL**

**REVIEW 2 –** A review of behavioural risk factors in midlife associated with successful ageing and the primary prevention or delay of disability, dementia, frailty and non-communicable chronic conditions.

Cambridge Institute of Public Health

28th November 2013 (v2)

**Principal Investigator:**

Professor Carol Brayne

**Correspondence to:**

Dr Louise Lafortune

Institute of Public Health, Forvie Site
University of Cambridge School of Clinical Medicine
Box 113 Cambridge Biomedical Campus
Cambridge, CB2 0SR

[ll394@medschl.cam.ac.uk](mailto:ll394@medschl.cam.ac.uk)

1. Guidance title

Disability, dementia and frailty in later life – mid-life approaches to prevent or delay the onset of these conditions.

- 1. **Short title**

Disability, dementia and frailty in later life – mid-life approaches to prevention

- 1. **Review team**

| **Staff/resource description^a^** | **No. of days per staff/ resource (all 3 reviews)^b^** | **Review 2^b^**  **^(Time specific to review 2)^** | **Email address** |
| --- | --- | --- | --- |
| Systematic Reviewer (Sarah Kelly) | 220 days (1 FTE)  (1 Aug ’13 – 30 Nov ‘14) | 88 days  (Sept ’13 – March 2014) | sak65@medschl.cam.ac.uk |
| Systematic Reviewer  (Steven Martin) | 55 days (0.25 FTE)  (1 Aug ’13 – 30 Nov ‘14) | 22 days  (Sept ’13 – March 2014) | sm987@medschl.cam.ac.uk |
| Admin/Technical Support (Andy Cowan) | 110 days (0.5 FTE)  (Sept’13 – Feb ’14) | 44 days  ((Sept ’13 – March 2014) | ac798@medschl.cam.ac.uk |
| Scientific Coordinator / Project Manager  (Louise Lafortune) | 22 days (0.10 FTE)  (1 Aug ’13 – 30 Nov ‘14) | 9 days  (Sept ’13 – March 2014) | ll394@medschl.cam.ac.uk |

^a^A third systematic reviewer (Dr Nadja Smailagic) is available to provide a third view on inclusion/exclusion of studies or data

^b^Likely division of time between reviews: review 1 (30%), review 2 (40%), review 3 (30%)

1. **Summary of the scope**

This review is the second of three to be conducted to inform the guidance on which primary prevention behaviours to be adopted in mid-life are most effective and cost-effective to prevent and delay the onset of disability, dementia, frailty, and other non-communicable chronic conditions in later-life.

The full scope of the guidance is available in the final scope document (Final scope, NICE Public Health Guidance, Disability, dementia and frailty in later life – mid-life approaches to prevention) that incorporates stakeholder comments from a 4-week consultation 21 March to 18 April 2013. Further background information and details of the broad approach to the conduct of the three reviews to provide evidence to inform the guidance is detailed in the tender proposal submitted by Cambridge Institute of Public Health, 15^th^ July 2013 (invitation to tender reference DDER 42013). The key details of these documents have been incorporated into this protocol.

- 1. **Review title and main objectives**
  - This systematic review (**Review 2**) of the literature is entitled:

Behavioural risk factors in midlife associated with successful ageing and the primary prevention or delay of disability, dementia, frailty, and non-communicable chronic conditions.

It will address the following questions:

- - What behavioural risk factors in midlife are associated with successful ageing and the primary prevention or delay of disability, dementia, frailty, and non-communicable chronic conditions? How strong are the associations and how does this vary for different subpopulations?

The two other reviews (and study protocols) related to this one address the following questions:

- - **Review 1**: What are the key issues for people in midlife that prevent or limit, or which help or motivate them to take up and maintain healthy behaviours and to what extent do they have an effect? (Review 1 is underway at the date of submission of this protocol).
  - **Review 3**: What are the most effective and cost-effective midlife interventions for increasing the uptake and maintenance of healthy behaviours? To what extent do the different health behaviours prevent or delay dementia? To what extent do the different health behaviours prevent or delay disability and frailty related to modifiable behavioural risk factors? To what extent do the different health behaviours prevent or delay non-communicable chronic diseases?
  1. **Groups that will be covered**
- Studies in adults aged 40-64 years (‘midlife’) at baseline, with a particular focus on people at increased risk of disability, dementia, frailty, or other non-communicable chronic conditions due to health behaviour and behavioural risk factors with outcomes in adults aged 55 and over relating to disability, dementia, frailty or non-communicable chronic conditions.
- Studies in adults aged 39 and younger from disadvantaged populations (as they are at increased risk of ill health and more likely to develop multiple morbidities). Disadvantaged populations will include (but is not limited to) low socioeconomic status; ethnic minority groups; lesbian, gay, bisexual, transsexual (LGBT) groups; travellers, and other groups with protected characteristics under the equality and diversity legislation.
  1. **Groups that will not be covered**
- Adults with any type of dementia or pre-existing cognitive impairments in midlife.
- Adults who are receiving treatment for a non-communicable chronic condition or who have a disability associated with behavioural risk factors will not be included for that particular condition or disability.
  1. **Activities**

**2.4.1 Activities that will be covered**

Behavioural risk factors for people in midlife (aged 40 to 64) that are associated with successful ageing or the development and progression of: disability, dementia, frailty (including bone health) and common non-communicable chronic conditions (NCCC’s) in older age (age 55 and over). Examples of NCCC’s include cardiovascular diseases and stroke, type 2 diabetes, chronic obstructive pulmonary disease, renal disease, osteoporosis and bone health, visual and hearing conditions and some cancers that may be associated with lifestyle (these may be defined by the types of studies found).

Behavioural risk factors for younger adults (aged 18 to 39) from disadvantaged populations (as defined in section 2.2) that are associated with successful ageing or the development and progression of: disability, dementia, frailty (including bone health) and common NCCC’s in later life. As disability, frailty and NCCC’s may present earlier in people from disadvantaged populations, outcomes for this group would not be restricted to those in people aged 55 and over.

Behavioural risk factors by people in midlife (aged 40 to 64) that are associated with the development and progression of ‘preconditions’ for disability, dementia, frailty (including bone health) and common NCCC’s in later life (age 55 and over). Such preconditions include high blood pressure, impaired glucose intolerance, high cholesterol, overweight/obesity, impaired cognitive function, mood disorders, and functional limitations.

Behavioural risk factors for younger adults (aged 18 to 39) from disadvantaged populations (as defined in section 2.2) that are associated with the development and progression of preconditions for disability, dementia, frailty (including bone health) and common NCCC’s in later life.

The scope includes:

1. Behavioural risk factors including less sedentary behaviour, increased physical activity, improved diet or components of diet (e.g. fat intake, fruit and vegetable intake), weight loss or control, cessation or reduction of smoking, reduction or modification of alcohol consumption, to maintain sufficient levels of social activity and avoid loneliness (this may vary for individuals), avoidance of excessive exposure to noise and addressing hearing and/or sight loss, or to improve/modify multiple behavioural risk factors and health behaviours in general.
2. Behavioural risk factors at individual, family, community, subnational or national level (these may be targeted at specific groups, particularly those who are at increased risk, or who are from disadvantaged groups, or at healthcare professionals).
3. Behavioural risk factors in a range of settings including primary and secondary care, and workplace and community settings in the private, public, voluntary or commercial sectors.

**2.4.2 Activities that will not be covered**

1. Use of drugs to prevent or treat dementia and non-communicable chronic conditions;
2. Use of dietary supplements;
3. Diagnosis and care of disability, dementia, frailty and common non-communicable chronic conditions;
4. Management of existing disability, dementia, frailty and common non-communicable chronic conditions;
5. Recreational drug use;
6. Management of obesity, including medical and surgical interventions for obesity;
7. Organisational interventions, policies and laws.

**2.4.3 Other aspects of the scope**

Associations between health-related behaviours in midlife and ageing well outcomes and NCCC’s, and between health-related behaviours and ‘preconditions’ such as overweight or obesity, or hypertension or raised cholesterol are covered by the scope of the review and the guidance. However, associations in people with existing dementia, disability, frailty or NCCC’s are outside the scope of this review and the guidance. Associations between preconditions and dementia, disability, frailty or NCCC’s are outside the scope of this review.

1. **Reviews**

**3.1. Overview**

This review is the second of three to be conducted to inform the guidance on mid-life approaches to prevent or delay the onset of disability, dementia or frailty in later life.

This review (Review 2) aims to identify behavioural risk factors in midlife that are associated with successful ageing and the primary prevention or delay of disability, dementia, frailty and non-communicable chronic conditions (see Table 1 for full definitions of terms).

A conceptual overview of the 3 reviews and how Review 2 fits into the overall scheme is presented in Figure 1.

The model details behavioural risk factors in midlife, interventions to improve or maintain healthy behaviours, intermediate biological risk factors that can be influenced by healthy behaviours and preventable outcomes relating to disability, dementia, frailty or NCCC’s in later life. The model will be used to inform the searches and selection of studies for the review within the inclusion/exclusion criteria outlined above in section 2.

**Healthy behaviours**

- Physical activity / Sedentary behaviours
- Diet
- Tobacco smoking
- Alcohol consumption
- Cognitive activities
- Noise exposure
- Work / Social activities / Participation

**REVIEW 1**

**Uptake & maintenance of healthy behaviours in mid-life**

**Barriers**

**Facilitators**

- Personal factors (e.g. gender, SES, ethnicity, employment, family, previous experiences, expectations)
- Social factors (e.g. norms, support)
- Environmental factors (e.g. access to resources/interventions; residential & work environment)
- Organisational factors (e.g. design & delivery of intervention, resources)

**Effect on ageing Well Outcomes**

- Disability (ADL, IALD, independence, mobility)
- Dementia
- Frailty
- Healthy life span
- Quality of life
- Participation

**Effect on non-communicable conditions**

- Cardiovascular diseases& stroke
- Renal disease
- Life style related cancers
- COPD
- Type II diabetes
- Osteoporosis / Bone health
- Hearing & Sight Loss

**Primary prevention of preconditions**

- Impaired glucose intolerance
- High blood pressure
- High cholesterol
- Overweight / Obesity (weight loss or control)
- Impaired cognitive function (MCI)
- Mood disorders
- Functional limitations

**Other relevant outcomes**

- Resource use, costs, cost effectiveness

**Effect on healthy behaviours**

- Increase/maintain “good” levels of physical activity OR decrease sedentary life styles OR maintain balance, strength and weight-bearing functions
- Improve/maintain good diet & nutrition
- Reduce/prevent/stop tobacco consumption
- Decrease/prevent excessive alcohol consumption
- Maintain/increase cognitive and social activities, and participation
- Prevent / decrease excessive noise/ sun exposure
- Improve/modify multiple behavioural risk factors
- Remove barriers / facilitate uptake & maintenance of any life style behaviours WITH demonstration of impact.

**Intervention**

Effectiveness & cost effectiveness…

**REVIEW 2**

Association between behavioural risk factors and ageing well outcomes & NCCC

**REVIEW 3**

**Figure 1.**

**3.2 Review question**

**Review question 2 (from a series of 3 reviews)**

- - What behavioural risk factors in midlife are associated with successful ageing and the primary prevention or delay of disability, dementia, frailty and non-communicable chronic conditions? How strong are the associations and how does this vary for different subpopulations?

**3.3 Review outcomes**

Evidence for behavioural risk factors in midlife that are associated with successful ageing, and the primary prevention or delay of disability, dementia, frailty and non-communicable chronic conditions, namely quantitative evidence of associations.

These quantitative outcomes will include the extent of the association between the type, level and amount of behavioural risk factor and ageing well or morbid outcomes including dementia, disability, frailty and NCCC’s.

**4. Methods**

**4.1 Inclusion criteria – types of studies**

a) The first tier of evidence to be included in the review will be primary longitudinal cohort studies that provide information on the association between behavioural risk factors at midlife and ageing well or morbid outcomes including dementia, disability, frailty and NCCCs in later life.

Only cohort studies that have conducted multivariate analyses will be included in this review. Studies that conducted only univariate analysis will be excluded.

Cross-sectional studies will be excluded from the review as they would only show a cross-sectional association, and would not provide information on the impact of behavioural risk factors in later life. Any cross-sectional analyses reported in studies in addition to longitudinal analyses will be excluded also. Qualitative studies will be excluded from the review, as they would not provide any quantitative evidence of an association between behavioural risk factors and ageing well or morbid outcomes in later life.

Abstracts, letters and editorials will be excluded. Theses will be excluded, although we will seek relevant published peer-reviewed papers based on thesis data. If found these would be included if they meet the inclusion criteria for the review.

b) Where no primary longitudinal cohort studies in midlife populations are found to cover a potentially relevant topic or area of interest, systematic reviews or meta-analyses of quantitative longitudinal observational studies in adult populations in general may be searched for and included if appropriate.

**4.2 Inclusion criteria – Dates of studies to be included**

Primary studies and systematic reviews published from year 2000 onwards.

**4.3 Inclusion criteria – observational studies**

For the purposes of this review, observational studies to be included are longitudinal cohort studies.

Population:

Studies in adults at midlife (aged 40 to 64 years for the general population) with outcomes at follow-up in people aged 55 and over. A younger age cut point (i.e. 55 years as opposed to 60 or 65 years) was selected with recognition of the fact that disease processes can be accelerated in disadvantaged populations.

Studies in adults from disadvantaged populations (as defined in section 2.2) aged 18-39 with outcomes at follow-up in later life, even if not in people aged 55 and over.

Studies would not be excluded on basis of country of origin, however, where the study was conducted will be considered in the applicability ratings.

Exposure:

Behavioural risk factors in the populations described above including (but not limited to) increase/maintain physical activity or decrease sedentary lifestyles; maintain balance, strength and weight-bearing functions; improve/maintain good diet (or components of diet) and nutrition; smoking cessation or reduction or prevention of smoking; decrease/moderate alcohol consumption or prevent excessive consumption; improve/modify multiple behavioural risk factors; healthy behaviours in general, increase/maintain social activity or prevent loneliness; increase or maintain/address management of sight loss or hearing loss, body weight, avoid excessive exposure to noise.

Outcomes**:** Dementia, disability (activities of daily living (ADL), instrumental activities of daily living (IALD), independence, mobility), frailty, healthy life span, quality of life, participation, NCCC’s including cardiovascular diseases and stroke, renal disease, cancer, chronic obstructive pulmonary disease, type 2 diabetes, osteoporosis and bone health.

Timescale: Follow-up of 5 years or over (follow-up of less than 5 years is unlikely to be sufficient for the development and measurement of dementia, disability, frailty or pre-conditions associated with behavioural risk factors)

Language: English language studies only.

**4.4 Inclusion criteria – systematic reviews**

Population, exposure and outcomes to be included as for observational studies (section 4.3)

Study types to be searched for will be systematic reviews or meta-analyses of longitudinal cohort studies in adults that have reported multivariate analyses and have follow-up of 5 years or longer.

Systematic reviews will be included if they answer the review question. The process for using review level material is described in more detail in section 6.

Where there exist systematic reviews published from 2000 onwards that cover all or part of a topic area, if the systematic review was published more than 5 years ago, the evidence will be updated using primary evidence published since (if available).

**5. Searching**

To develop the search strategies, an iterative approach will be taken involving the whole team, which consists of the following steps:

1. Initial team discussions around research questions.
2. Initial drafting of search building at least (but not exclusively) on the final scope for this guidance, comments received from key stakeholders on the draft scope, high quality peer-review systematic reviews (when available) on same or similar topics for each key domains of the strategy, (e.g. health, preventative interventions, behaviours, etc.);
3. Testing of individual components and development of the review specific strategies in key databases;
4. Refining of specific review strategies upon discussion with information specialist;
5. Updating of search strategies based on reviewers comments;
6. Adaptation of strategies to individual databases (i.e. Mesh terms or filters in one database don’t usually apply to other databases);
7. Running of search and uploading of references in individual Endnote data bases (for specified time period, i.e. since 2000);
8. Create a combined Endnote database (master file); delete duplicate and prepare for title screening;
9. Identification of potential included studies; selection of full text for further assessment; identification of included and excluded studies.

A structured search strategy will be developed and conducted for this review (see Appendix B for draft Medline Ovid search with number of hits for key term combinations). Searches will be restricted to publications in English language.

Searching will be conducted in two stages: 1) searching for primary longitudinal cohort studies using an observational study search filter agreed with CPH), 2) where there are no primary studies covering a topic or area, targeted searches will then be conducted for relevant systematic reviews in adults in general as appropriate, using a systematic review search filter agreed with CPH.

5.1 Databases to be searched for peer-reviewed primary studies published since year 2000 (with host platform):

- MEDLINE (including MEDLINE – in-process) (Ovid)
- EMBASE (Ovid)
- PsycINFO (Ovid)
- CINAHL (EBSCO host)
- Health Management Information Consortium (Ovid)
- Social Science Citation Index (Web of Knowledge)

5.2 Additional databases to be searched for systematic reviews (if necessary) published since year 2000 (with host platform):

- The Cochrane Collaboration databases ([www.thecochranelibrary.com](http://www.thecochranelibrary.com))
  - Cochrane Database of Systematic reviews
  - Database of Abstracts of Reviews of Effectiveness
  - HTA database

5.2 Websites to be searched for grey literature and peer-reviewed studies published since year 2000 (for both primary studies and systematic reviews):

- NHS Evidence Search ([www.evidence.nhs.uk](http://www.evidence.nhs.uk))
- Open Grey ([www.opengrey.eu](http://www.opengrey.eu))
- Public Health Observatories ([www.apho.org.uk](http://www.apho.org.uk))
- Health Evidence Canada ([www.healthevidence.org](http://www.healthevidence.org))
- Alzheimer’s Society ([www.alzheimers.org.uk](http://www.alzheimers.org.uk))
- RNIB ([www.fightforsight.org.uk](http://www.fightforsight.org.uk))
- Fight for Sight ([www.fightforsight.org.uk](http://www.fightforsight.org.uk))
- Action on Hearing Loss ([www.actiononhearingloss.org.uk](http://www.actiononhearingloss.org.uk))
- Beth Johnson Foundation ([www.bjf.org.uk](http://www.bjf.org.uk))
- British Library (<http://www.bl.uk>)
- Campbell Collaboration (<http://www.campbellcollaboration.org>)
- Department of Health (<https://www.gov.uk/government/publications>)
- E-Print Network (<http://www.osti.gov/eprints/>)
- Google Scholar (<http://scholar.google.co.uk>)
- Grey Literature Report (<http://www.greylit.org>)
- Lenus (<http://www.lenus.ie/hse/>)
- OAIster (<http://www.oclc.org>)
- Public Health Europe (<http://ec.europa.eu/health/index_en.htm>)
- RAND Health (<http://www.rand.org/health.html>)
- Scirus (<http://www.scirus.com>)
- World Health Organisation (<http://www.who.int/en/>)

Records retrieved from the searches will be reported as in Appendix C, CPHE methods manual.

**6. Identification & selection of relevant studies (Section 5.2 CPHE methods manual)**

Titles and/or abstracts will be screened independently by 2 reviewers using the inclusion criteria detailed in the review protocol. Differences between reviewer’s results will be resolved by discussion and when necessary in consultation with a third reviewer. If after discussion, there is still doubt about a study’s relevance for the review it will be retained.

Full paper copies will be obtained for all reviews and studies identified by the title/abstract screening. A full paper screening tool with inclusion/exclusion criteria as defined in the review protocol will be developed for screening of the full papers. Full paper screening will be conducted independently by two people. Any differences of opinion about inclusion/exclusion will be resolved by discussion between the two reviewers or by consultation with a third reviewer.

Additionally, for systematic reviews, the process for using review level material as described in Appendix J of the CPHE methods manual will be followed. Systematic reviews will initially be screened using the review screening form in Appendix J of the CPHE methods manual to determine if the review is relevant to the guidance topic. Adaptations to the form will be jointly agreed with the CPH project team.

Additionally, experts in the field will be consulted to identify any further potentially relevant papers through contacts and links with the Institute of Public Health at University of Cambridge. Also, responses to the NICE call for evidence relating to this guidance conducted between 31/5/2013 and 28/6/2013 will be screened for potential inclusion in the review.

A flow chart will be used to summarise the number of papers included and excluded at each stage of the process. Primary studies or systematic reviews excluded at the full paper screening stage will be listed in the appendix of the review along with the reason for exclusion.

**7. Quality Assessment**

Two types of studies will be included in the review: cohort studies and systematic reviews of cohort studies. Study designs will be assigned using the glossary of study designs (appendix D, CPHE methods manual) and the algorithm for classifying study designs (appendix E, CPHE methods manual).

Cohort studies

Quality appraisal of cohort studies will be done using the relevant quality appraisal checklist in the NICE methods manual (Appendix D; CPHE methods manual).

Each full paper will be assessed by one reviewer and checked for accuracy by another. A minimum of 10% of the studies will be fully double assessed. Any discrepancy between reviewers would be resolved by discussion.

The composite inter-rater reliability studies will be reported as a kappa statistic noting if it is good (0.60 to 0.74) or excellent (above 0.75). If the inter-rater reliability score is below 0.60, the reasons for digression would be explored and a course of action agreed.

Systematic reviews

The methodological quality of each systematic review will be assessed using the AMSTAR tool (www.Amstar.ca). Each full review will be assessed by one reviewer and checked for accuracy by another. A minimum of 10% of the studies will be fully double assessed. Any discrepancy between reviewers would be resolved by discussion.

The quality of the evidence presented in the included systematic reviews would not be reassessed by the authors. The results would be extracted into the evidence tables and narrative summary and a narrative summary would be provided of the highest rated evidence presented in each of the systematic reviews, where this is different from the overall summary of the evidence provided in the review. This would be possible because all the included reviews would assess the quality of their primary studies (meeting AMSTAR criterion 7).

**8. Data extraction**

We will extract data on participant, exposure, study and health outcome characteristics, effect size. To ensure accurate reporting the data extraction pro-forma will be piloted against two included papers. Each included full paper will be assessed by one reviewer and checked for accuracy by another. A minimum of 10% of the studies will be fully double extracted (as above for quality assessment).

**9. Synthesis of evidence**

Only quantitative evidence will be included in this review. Findings will be narratively synthesised and presented to inform guidance. Data specific to health inequalities and vulnerable communities will be assessed and findings may be summarised separately if sufficient data is available.

Information about included studies will be presented in both narrative and evidence table sections of the review, and in sufficient detail, to ensure clear and transparent links between recommendations and evidence (Section 5, Appendix K, CPHE methods manual).

Narrative summaries of both primary quantitative studies and systematic reviews will be written as outlined in section 5.4.2. CPHE methods manual. Key themes based on analysis of the evidence tables across each topic area will be synthesised in a narrative format (where sufficient data is available to identify themes). Otherwise, a descriptive approach to the available evidence will be taken (see section 5.4.5 CPHE methods manual).

For each key question or issue an evidence statement will be generated which will provide an aggregated summary of all of the relevant studies (Sections 5.5.1 to 5.5.5 CPHE methods manual). Applicability ratings will be used to assess each evidence statement to judge how similar the population(s), setting(s), exposure(s) and outcome(s) of the included studies are to those outlined in the review question (Section 5.6 CPHE methods manual). Each evidence statement will be assessed as ‘directly applicable, partially applicable or not applicable’.

**10. Project management**

The proposed timelines for each review (Appendix C, CPHE methods manual) are aligned with the project timelines (and the PHAC committee meetings) set forth by NICE in appendix B of the invitation to tender.

**10.1 Quality assurance**

We will adhere to the NICE process and comply with the highest standard of quality.

**10.2 Data Protection and Freedom of Information Acts**

As full time researchers at the University of Cambridge everything we do falls under the Data Protection and Freedom of Information Acts. We will keep a record of all activities pertaining to this project and comply to NICE’s request shall the situation arise.

This project does not involve collecting nor analysing personal data - the main source of information (i.e. scientific evidence) is already in the public domain. All project specific correspondence, documents, etc. will be available to the NICE project team. The CPHE Methods Manual (namely chapters 3 through 7) lists the key steps of the evidence review and the project information will be organised accordingly.

Date and version of the protocol: 28th Nov 2013 (v2)

**Table 1 – Operational definitions**

| Successful ageing | Successful ageing is defined as survival to an advanced age while maintaining physical and cognitive function, functional independence and a full and active life. It means that morbidity and disability are compressed into a relatively short period before death, in line with the ‘compression of morbidity’ theory. |
| --- | --- |
| Disability | Disability will refer to any long-term restriction on the ability to perform an activity in the manner, or within the range, considered normal. |
| Dementia | Dementia will refer to a progressive, degenerative condition caused by diseases of the brain. Whether it occurs alone, in addition to, or as a combination of, chronic conditions, it is characterised by cognitive and non-cognitive symptoms of variable frequency and severity. |
| Frailty | Frailty will refer to a syndrome characterised by age-related declines in functional reserves where a small insult (e.g. infection, loss a partner) results in a striking and disproportionate change in health state. Frail older adults experience an increased risk of adverse health outcomes such as falls, fractures, comorbidity, disability, dependency, hospitalization, need for long-term care and mortality. |
| Non-communicable chronic conditions | Non-communicable chronic conditions will include cardiovascular diseases, diabetes, chronic obstructive pulmonary diseases, obesity, visual and hearing conditions, and some cancers that may be associated with behavioural risk factors. |
| Disadvantaged populations | Disadvantaged populations will include (but is not limited to) low socioeconomic status, ethnic minority groups, lesbians, gay, bisexual and transsexual (LGBT) community groups, travellers and other groups with protected characteristics under the equality and diversity legislation. |

**Appendix A – Draft project timescale**

| Task | Review 1 | Review 2 | Review 3 |
| --- | --- | --- | --- |
| Submission of draft evidence review to NICE team | 7 November 2013 | 19 December 2013 | 6 February 2014 |
| NICE provide comments on draft review | 15 November 2013 | 9 January 2014 | 14 February 2014 |
| Submission of revised draft review to NICE | 2 December 2013 | 22 January 2014 | 3 March 2014 |
| Draft review mailed to PHAC members | 6 December 2013 | 24 January 2014 | 6 March 2014 |
| Submission of final slides for presentation of review to PHAC | 6 December 2013 | 24 January 2014 | 6 March 2014 |
| Presentation of draft review at PHAC meeting | 18 December 2013 | 5 February 2014 | 19 March 2014 |
| Final amendments to be made to report post PHAC meeting | 9 January 2014 | 20 February 2014 | 3 April 2014 |

**Appendix C – Timelines review 2: A review of lifestyle factors in midlife associated with the promotion of successful ageing**

| **Project Activity** | **Actions** | **2013** | | | | | | **2014** | | | | | | | | | | |
| --- | --- | --- | --- | --- | --- | --- | --- | --- | --- | --- | --- | --- | --- | --- | --- | --- | --- | --- |
|  |  | **Jul** | **Aug** | **Sep** | **Oct** | **Nov** | **Dec** | **Jan** | **Feb** | **Mar** | **Apr** | **May** | **Jun** | **Jul** | **Aug** | **Sep** | **Oct** | **Nov** |
| **Protocol** | Initial discussions with the NICE team |  |  |  |  |  |  |  |  |  |  |  |  |  |  |  |  |  |
|  | Feedback from team incorporated into protocol & finalised |  |  |  |  |  |  |  |  |  |  |  |  |  |  |  |  |  |
|  | **Allocation of roles and responsibilities** |  |  |  |  |  |  |  |  |  |  |  |  |  |  |  |  |  |
| **Review** | Development of search strategy |  |  |  |  |  |  |  |  |  |  |  |  |  |  |  |  |  |
|  | Databases search & reference retrieval |  |  |  |  |  |  |  |  |  |  |  |  |  |  |  |  |  |
|  | Quality Assessment |  |  |  |  |  |  |  |  |  |  |  |  |  |  |  |  |  |
|  | Data Extraction |  |  |  |  |  |  |  |  |  |  |  |  |  |  |  |  |  |
|  | Missing information/data followed up |  |  |  |  |  |  |  |  |  |  |  |  |  |  |  |  |  |
|  | Analysis |  |  |  |  |  |  |  |  |  |  |  |  |  |  |  |  |  |
|  | Writing of report |  |  |  |  |  |  |  |  |  |  |  |  |  |  |  |  |  |
|  | Submission of draft evidence review to NICE team |  |  |  |  |  |  |  |  |  |  |  |  |  |  |  |  |  |
|  | Feedback from NICE team |  |  |  |  |  |  |  |  |  |  |  |  |  |  |  |  |  |
|  | Final amendments |  |  |  |  |  |  |  |  |  |  |  |  |  |  |  |  |  |
|  | Provide support for NICE guidance |  |  |  |  |  |  |  |  |  |  |  |  |  |  |  |  |  |
