# Supplementary material for: Behavioural Risk Factors in Mid-Life Associated with Successful Ageing, Disability, Dementia and Frailty in Later Life: A Rapid Systematic Review
Source: PLoS One. 2016 Feb 4;11(2):e0144405. doi: 10.1371/journal.pone.0144405 (PMC4742275; doi:10.1371/journal.pone.0144405)
Supplement: S2 Text — (DOCX) [file pone.0144405.s007.docx]

**Search Strategies**

**1. Sample search strategy used to identify systematic reviews**

Sample search: Ovid MEDLINE(R) In-Process & Other Non-Indexed Citations and Ovid MEDLINE(R) <1946 to Present>

Note: Searches terms were modified were necessary when searching other databases.

1 ((dement* or alzheimer* or disability* or disabled or diabet* or angina or stroke or copd or frail* or bronchiti* or melanoma* or carcinoma* or cancer* or neoplasm* or tumo?r* or blind* or deaf* of glaucoma*) adj3 (prevent* or control* or limit* or restric* or restrain* or obstruct* or inhibit* or imped* or delay* or constrain*)).ti,ab. (276946)

2 (lewy* adj2 bod* adj3 (prevent* or control* or limit* or restrict* or restrain* or obstruct* or inhibit* or imped* or delay* or constrain*)).ti,ab. (67)

3 ((coronary* or vascular* or cardiac or cardiovasc* or cardio vasc* or cerebrovasc* or heart*1 or myocardia*) adj3 (bypass* or graft* or disease* or event* or infarct* or re?vascular* or isch?emi* or peripheral* or complication* or disorder*) adj3 (prevent* or control* or limit* or restrict* or restrain* or imped* or obstruct* or inhibit* or delay* or constrain*)).ti,ab. (29741)

4 (((glucose adj3 (intoleran* or toleran*)) or (insulin adj3 resistan*)) adj3 (prevent* or control* or limit* or restrict* or restrain* or obstruct* or inhibit* or imped* or delay* or constrain*)).ti,ab. (2630)

5 (obstruct* adj3 (pulmonary or lung* or airway* or airflow* or bronch* or respirat*) adj3 (prevent* or control* or limit* or restrict* or restrain* or inhibit* or imped* or delay* or constrain*)).ti,ab. (1247)

6 (((visual adj3 impair*) or (vision* adj3 disorder*) or (macular adj3 degenerat*)) adj3 (prevent* or control* or limit* or restrict* or restrain* or obstruct* or inhibit* or imped* or delay* or constrain*)).ti,ab. (436)

7 (hear* adj3 (impair* or difficult* or hard* or disorder*) adj3 (prevent* or control* or limit* or restrict* or restrain* or obstruct* or inhibit* or imped* or delay* or constrain*)).ti,ab. (368)

8 ((cognition disorder* or cognitive impair*) adj3 (prevent* or control* or limit* or restrict* or restrain* or obstruct* or inhibit* or imped* or delay* or constrain*)).ti,ab. (950)

9 exp Dementia/pc (4213)

10 exp Wheelchairs/ (3567)

11 exp Cardiovascular Diseases/pc (156094)

12 exp Cardiovascular Deconditioning/ (244)

13 exp Cerebrovascular Disorders/pc (21793)

14 exp Diabetes Mellitus/pc (20043)

15 exp Pulmonary Disease, Chronic Obstructive/pc (596)

16 exp Lung Diseases, Obstructive/pc (6349)

17 exp Frail Elderly/ (6818)

18 Melanoma/pc (1547)

19 exp Blindness/pc (1957)

20 exp Vision Disorders/pc (3318)

21 exp Deaf-Blind Disorders/pc (3)

22 exp Hearing Disorders/pc (3446)

23 exp Glaucoma/pc (887)

24 exp Lung Neoplasms/pc (4337)

25 exp Skin Neoplasms/pc (4524)

26 exp Colorectal Neoplasms/pc (9228)

27 exp Colonic Neoplasms/pc (3678)

28 exp Intestinal Neoplasms/pc (9545)

29 exp Rectal Neoplasms/pc (754)

30 exp Stomach Neoplasms/pc (1463)

31 exp Mouth Neoplasms/pc (1249)

32 exp macular degeneration/pc (783)

33 exp cognition disorders/pc (2314)

34 ((ageing or aging) adj3 (well or success* or positive* or active* or healthy or unhealthy or unsuccess*)).ti,ab. (6519)

35 (compress* adj3 morbid*).ti,ab. (181)

36 or/1-35 (503786)

37 exp *Middle Aged/ (847)

38 (middle adj age*).ti. (9779)

39 (baby adj2 boomer*).ti. (221)

40 (midlife or "mid life" or "midlives" or "mid lives").ti. (1675)

41 or/37-40 (12244)

42 Epidemiologic studies/ (6282)

43 exp case control studies/ (668222)

44 exp cohort studies/ (1374003)

45 Case control.tw. (80438)

46 (cohort adj (study or studies)).tw. (90189)

47 Cohort analy*.tw. (3823)

48 (Follow up adj (study or studies)).tw. (38213)

49 (observational adj (study or studies)).tw. (46124)

50 Longitudinal.tw. (146429)

51 Retrospective.tw. (276650)

52 Cross sectional.tw. (175165)

53 Cross-sectional studies/ (181453)

54 or/42-53 (1929289)

55 exp Regression Analysis/ (308240)

56 ((multivariat* or regress* or varia* or bivariat*) adj3 analys*).tw. (337057)

57 exp multivariate analysis/ (86933)

58 or/54-57 (2266895)

59 letter/ (835895)

60 editorial/ (353835)

61 comment/ (583940)

62 animal/ (5513032)

63 human/ (13712336)

64 62 not (62 and 63) (3974352)

65 59 or 60 or 61 or 64 (5249555)

66 dement*.ti,ab. (71853)

67 alzheimer*.ti,ab. (94300)

68 (lewy* adj2 bod*).ti,ab. (6183)

69 (disabilit* or disabled).ti,ab. (123911)

70 (wheelchair* or walking aid* or walker*1).ti,ab. (14208)

71 cardiovascular*.ti,ab. (284550)

72 (coronary* adj3 (bypass* or graft* or disease* or event*)).ti,ab. (147435)

73 cerebrovascular*.ti,ab. (38161)

74 cardio?vasc*.ti,ab. (285073)

75 (myocardial* adj3 (infarct* or re?vascular* or isch?emi*)).ti,ab. (174190)

76 (vascular* adj3 (peripheral* or disease* or complication*)).ti,ab. (56118)

77 (angina* or stroke*).ti,ab. (196749)

78 (heart* adj3 (disease* or attack* or bypass*)).ti,ab. (138679)

79 diabet*.ti,ab. (421288)

80 ((glucose adj3 (intoleran* or toleran*)) or (insulin adj3 resistan*)).ti,ab. (83550)

81 copd.ti,ab. (27312)

82 (obstruct* adj3 (pulmonary or lung* or airway* or airflow* or bronch* or respirat*)).ti,ab. (61501)

83 (chronic* adj3 bronchiti*).ti,ab. (10432)

84 frail*.ti,ab. (9086)

85 (lung adj3 (cancer* or neoplasm* or tumo?r*)).ti,ab. (116128)

86 melanoma*.ti,ab. (82712)

87 ((bowel* or colorectal* or rect* or intestin* or colon*) adj3 (cancer* or neoplasm* or tum?or*)).ti,ab. (124255)

88 (stomach* adj3 (cancer* or neoplasm* or tum?or*)).ti,ab. (11225)

89 ((oral* or mouth*) adj3 (cancer* or neoplasm* or tum?or*)).ti,ab. (14878)

90 (blind* or (visual* adj3 impari*) or (vision* adj3 disorder*)).ti,ab. (220907)

91 (deaf* or (hear* adj3 (impair* or difficult* or hard* or disorder*))).ti,ab. (45625)

92 glaucoma*.ti,ab. (43418)

93 ((ageing or aging) adj3 (well or success* or positive* or active* or healthy or unhealthy or unsuccess*)).ti,ab. (6519)

94 maculopath*.ti,ab. (3012)

95 ((macul or retina* or choroid*) adj3 degener*).ti,ab. (7847)

96 (macula* adj2 lutea).ti,ab. (112)

97 (skin* adj3 (cancer or neoplasm* or tumo?r*)).ti,ab. (22468)

98 (cogniti* adj3 (disorder* or degenerat*)).ti,ab. (5517)

99 ((limit* or difficult*) adj3 (mobil* or walk* or ambulat*)).ti,ab. (5193)

100 osteoporo*.ti,ab. (52576)

101 osteopenia*.ti,ab. (7137)

102 (bone* adj3 (dens* or loss* or mass* or age* or defect* or mineral* or disease* or health*)).ti,ab. (104300)

103 ur?emi*.ti,ab. (25975)

104 ((kidney* or renal*) adj3 (transplant* or graft* or fail* or disease*)).ti,ab. (212407)

105 (hemodialysis or haemodialysis or dialysis or pre-dialysis or predialysis).ti,ab. (118000)

106 (CKD or CKF or CRD or CRF or ESKD or ESRD or ESKF or ESRF).ti,ab. (35716)

107 (obes* adj2 diabet*).ti,ab. (15199)

108 (mody or niddm).ti,ab. (7673)

109 (diabet* and (non insulin* depend$ or noninsulin* depend* or noninsulindepend* or non insulindepend*)).ti,ab. (11972)

110 ((typ* 2 or typ* II) adj diabet*).ti,ab. (79809)

111 ((ketoresist* or keto* resist* or nonketo* or non keto*) adj diabet*).ti,ab. (263)

112 ((adult* or matur* or late or slow or stabl*) adj diabet*).ti,ab. (1313)

113 ((plurimetabolic* or metabolic) adj syndrom*).ti,ab. (29347)

114 (insulin* defic* adj relativ*).ti,ab. (7)

115 hyperglyc?em*.ti,ab. (41987)

116 (compress* adj3 morbid*).ti,ab. (181)

117 exp dementia/ (122570)

118 exp Disabled Persons/ (46518)

119 exp Wheelchairs/ (3567)

120 exp Cardiovascular Deconditioning/ or exp Cardiovascular diseases/ (1885236)

121 exp Cerebrovascular Disorders/ (277649)

122 exp Diabetes Mellitus/ (320101)

123 exp Pulmonary Disease, Chronic Obstructive/ (24783)

124 exp Lung Diseases, Obstructive/ (167822)

125 exp Frail Elderly/ (6818)

126 exp Lung Neoplasms/ (179429)

127 exp Skin Neoplasms/ (97248)

128 exp melanoma/ (75062)

129 exp intestinal neoplasms/ or exp colorectal neoplasms/ or exp colonic neoplasms/ or exp rectal neoplasms/ (173781)

130 exp Stomach Neoplasms/ (74319)

131 exp Mouth Neoplasms/ (55023)

132 exp Blindness/ (20465)

133 exp Vision Disorders/ (58963)

134 exp Deaf-Blind Disorders/pc or exp hearing disorders/ or exp hearing impaired persons/ (70943)

135 exp glaucoma/ (42761)

136 exp macular degeneration/ (17379)

137 exp retinal degeneration/ (31530)

138 exp retinal neovascularization/ (2366)

139 exp choroidal neovascularization/ (4692)

140 exp macula lutea/ (10257)

141 exp cognition disorders/ (64141)

142 exp mobility limitation/ (2363)

143 exp "bone and bones"/ (484556)

144 exp bone density/ (41566)

145 exp osteoporosis/ (45428)

146 exp bone diseases, metabolic/ (63376)

147 exp uremia/ (22454)

148 exp renal insufficiency/ (124924)

149 exp kidney failure, chronic/ (78957)

150 exp renal dialysis/ (92574)

151 exp renal dialysis/ or exp dialysis/ (115058)

152 exp Diabetes mellitus, non insulin dependent/ (87707)

153 exp Insulin resistance/ (56125)

154 exp hyperglycemia/ (26892)

155 exp Diabetes Mellitus/ (320101)

156 or/66-155 (4784767)

157 exp health behavior/ (100801)

158 exp risk reduction behavior/ (7634)

159 exp Health promotion/ (55508)

160 exp primary prevention/ (115346)

161 exp preventive medicine/ (32449)

162 exp life style/ (65495)

163 exp food habits/ (21495)

164 exp food preferences/ (10186)

165 exp food preferences/ (10186)

166 exp vision tests/ (81401)

167 exp hearing tests/ (38265)

168 exp SMOKING/ (125355)

169 exp SMOKING CESSATION/ (21260)

170 exp "Tobacco Use Disorder"/ (8504)

171 exp "Tobacco Use Cessation"/ (21973)

172 exp Tobacco smoke pollution/ (10787)

173 exp ALCOHOL DRINKING/ (53408)

174 exp alcohol deterrents/ (4214)

175 exp drinking behavior/ (58899)

176 exp temperance/ (2647)

177 exp Loneliness/ (2183)

178 exp EXERCISE/ (115713)

179 exp Sports/ (114218)

180 exp exercise therapy/ (30462)

181 exp physical exertion/ (55019)

182 exp physical fitness/ (22555)

183 exp "Physical Education and Training"/ (13758)

184 exp exercise test/ (51288)

185 exp walking/ (20457)

186 exp running/ (13969)

187 exp jogging/ (702)

188 exp bicycling/ (7943)

189 exp swimming/ (19044)

190 exp dancing/ (1856)

191 exp gardening/ (475)

192 exp fitness centers/ (339)

193 exp sedentary lifestyle/ (2625)

194 (health* adj3 (behavior* or behaviour*)).ti,ab. (29899)

195 ((ageing or aging) adj3 (well or success* or positive* or active* or healthy)).ti,ab. (6481)

196 (food* adj3 choice*).ti,ab. (3151)

197 dieting.ti,ab. (2965)

198 (diet* adj3 (health* or balance* or fat* or salt* or sugar* or mediterranean or choice* or improv* or unhealthy or nutritious)).ti,ab. (60083)

199 ((fruit* or vegetable* or salt* or fat* or sugar*) adj3 (intake* or consum* or eat* or ate)).ti,ab. (33245)

200 (undernutrition or undernourish* or under-nutrition* or under-nourish*).ti,ab. (7463)

201 (multimicronutrient* or multi-micronutrient* or micronutrient* or micro-nutrient* or multinutrient* or multi-nutrient*).ti,ab. (8883)

202 ("five a day" or "5 a day").ti,ab. (179)

203 ("health check" or "check-up").ti,ab. (5509)

204 "health mot*".ti,ab. (285)

205 ((eye or eyesight or sight* or vision* or visual* or hearing) adj3 (test* or check* or screen*)).ti,ab. (20270)

206 (smok* or tobacco or cigar* or nicotine).ti,ab. (252522)

207 ((Alcohol* or Drunk* or Drink*) and (consum* or misuse or abus* or intoxicat* or harmful or excess* or binge or hazardous or heavy or temperan* or abstinen*)).ti,ab. (100162)

208 temperan*.ti,ab. (237)

209 teetotal*.ti,ab. (257)

210 (loneli* or lonely).ti,ab. (3656)

211 (socialis* or socializ*).ti,ab. (9480)

212 (social* adj3 (isolat* or network* or contact* or alien*)).ti,ab. (17204)

213 (sedentary or exercis* or sport*).ti,ab. (251538)

214 "physical condition*".ti,ab. (4515)

215 (balance* and (exercise* or retrain* or re-train* or reeducat* or re-educat*)).ti,ab. (6739)

216 inactiv*.ti,ab. (252817)

217 (walk* or run* or jog* or swim* or danc* or garden* or cycl* or bicycl* or bike* or recreation*).ti,ab. (1102662)

218 ("resistance trainiing" or "acquatic exercis*" or "wellness centre*" or "wellness center*").ti,ab. (154)

219 ("weight gain*" or "weight los*" or "overweight" or "over weight").ti,ab. (130192)

220 (obesity and "related behavio*").ti,ab. (510)

221 (overeat* or "over eat*").ti,ab. (1973)

222 (waist adj3 (circumference* or measur*)).ti,ab. (16292)

223 ((bmi or "body mass index") adj3 (gain* or loss* or lose* or lost or change*)).ti,ab. (4960)

224 (weight adj2 (cycling or reduc* or los* or maint* or decreas* or increas* or watch* or control*)).ti,ab. (106278)

225 "weight change*".ti,ab. (7524)

226 ((behavio?r or lifestyle or "life style") adj3 (change* or changing or modification or modify or modifying or therapy or therapies or program* or intervention* or counsel*)).ti,ab. (47776)

227 ((physical* or keep* or cardio* or aerobic or fitness) adj3 (fit* or activit* or train*)).ti,ab. (115603)
